# Low-Coordinate W–W Quadruple Bond as an Alkene Surrogate: Divergent [2 + 2 + 2] and [2 + 2 + 1] Cycloadditions with Alkynes

**DOI:** 10.1021/jacsau.6c01251

**Published:** 2026-09-24

**Authors:** Wen-Chi Chen, Cian-Wei Yang, Bo-An Liao, Chiao-Yun Chen, Hong-Zhang Chen, Wen-Chuan Hsu, Yi-Chou Tsai

**Affiliations:** Department of Chemistry, 34881 National Tsing Hua University, Hsinchu 30013, Taiwan

**Keywords:** tungsten, metal–metal bond, cycloaddition, metallacycle, regioselectivity

## Abstract

Although quadruply bonded metal–metal complexes constitute a large and well-established family, their cycloaddition chemistry remains underdeveloped because the quadruple bond is typically confined within a tetragonal, highly ligand-protected coordination environment. Herein, we demonstrate that a low-coordinate amidinate-supported W–W quadruple bond in W_2_(μ-Cl)­(μ-κ^2^-Cl_2_Li­(THF))­[μ-κ^2^-HC­(N-2,6-^*i*^Pr_2_C_6_H_3_)_2_]_2_ behaves as an alkene surrogate in cycloaddition chemistry. Depending on ligand coordination, this W–W quadruple bond engages alkynes in either formal [2 + 2 + 2] or [2 + 2 + 1] pathways. In the absence of ancillary donor ligands, direct reaction with two alkynes proceeds through tail-to-tail [2 + 2 + 2] cycloaddition to afford C_4_W_2_ metallacycles, W_2_Cl_2_[μ-η^2^:η^2^-1,4-R_2_C_4_H_2_]­[μ-κ^2^-HC­(N-2,6-^*i*^Pr_2_C_6_H_3_)_2_]_2_ (R = C_6_H_5_, 2-MeC_6_H_4_, 4-MeOC_6_H_4_, and *n*-C_3_H_7_), and a corresponding [2 + 2] intermediate, W_2_Cl_2_[μ-η^2^-2-MeC_6_H_4_CCH]­[μ-κ^2^-HC­(N-2,6-^*i*^Pr_2_C_6_H_3_)_2_]_2_, could be isolated and structurally characterized. In contrast, coordination of σ-donating ligands to the W–W core diverts the reaction pathway by stabilizing vinylidene intermediates, W_2_Cl_2_(C_5_H_5_N)­[μ-η^1^:η^2^-CC­(H)­R^1^]­[μ-κ^2^-HC­(N-2,6-^*i*^Pr_2_C_6_H_3_)_2_]_2_ (R^1^ = 2-MeC_6_H_4_, ^*t*^Bu, and SiMe_3_), which subsequently react with a second alkyne (R^2^CCH) to furnish C_3_W_2_ ring products of the type W_2_Cl_2_[μ-η^2^:η^2^-1-R^2^-4-R^1^H–C_4_H]­[μ-κ^2^-HC­(N-2,6-^*i*^Pr_2_C_6_H_3_)_2_]_2_ bearing an exocyclic alkene fragment. In both [2 + 2 + 2] and [2 + 2 + 1] manifolds, while the initial cycloadducts adopt nonplanar conformations, sequential reduction populates the W–W δ manifold and drives planarization via δ-π conjugation between the W_2_ unit and the organic fragment. Ultimately, the C_4_W_2_ systems are transformed into aromatic bimetallic metallabenzenes, [W_2_(μ-κ^2^-1,4-R_2_C_4_H_2_)­(μ-κ^2^-HC­(N-2,6-^*i*^Pr_2_C_6_H_3_)_2_)_2_] (R = C_6_H_5_, 2-MeC_6_H_4_, 3-MeC_6_H_4_, 4-MeC_6_H_4_, 4-MeOC_6_H_4_, 4-FC_6_H_4_, and *n*-C_3_H_7_), whereas the C_3_W_2_ system evolves into a bimetallic fulvene analogue, W_2_Cl­[μ-κ^2^-1-C_6_H_5_-4-^*t*^BuH-C_4_H]­[μ-κ^2^-HC­(N-2,6-^*i*^Pr_2_C_6_H_3_)_2_]_2_ and W_2_(μ-H)­[μ-κ^2^-1-C_6_H_5_-4-^*t*^BuH-C_4_H]­[μ-κ^3^-HC­(N-2,6-^*i*^Pr_2_C_6_H_3_)­(N-2-^*i*^Pr-6-(C_3_H_6_)­C_6_H_3_)]­[μ-κ^2^-HC­(N-2,6-^*i*^Pr_2_C_6_H_3_)_2_]. Taken together, these results expand the scope of cycloaddition partners to include a low-coordinate W–W quadruple bond.

## Graphical abstract

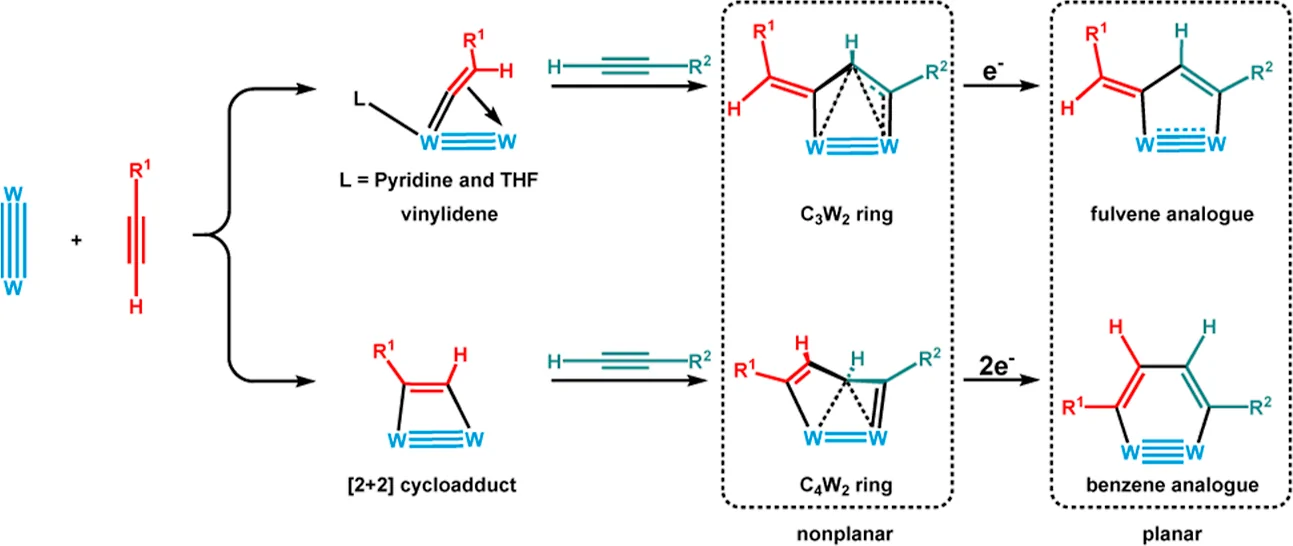

## Introduction

Cycloaddition reactions are among the most powerful methods for constructing cyclic scaffolds with high atom economy.^1^ Under transition-metal catalysis, unsaturated organic components can often be assembled efficiently into cyclic products, although achieving chemodivergence from identical starting materials remains a central challenge.^2,3^ Among these transformations, [2 + 2 + 2] cycloaddition has become one of the most versatile strategies for the single-step construction of six-membered and aromatic frameworks.^4–8^ This reaction typically combines three π components, most commonly alkynes, alkenes, or nitriles. For example, [2 + 2 + 2] cyclotrimerization of alkynes provides benzene derivatives (Figure 1a, top), whereas replacement of one alkyne by an alkene can furnish 1,3-cyclohexadiene derivatives through a related pathway. In contrast, formal [2 + 2 + 1] cycloaddition leading to fulvenes or alkylidenecyclopentenes remains far less common and generally requires more stringent substrate and reaction design, in part because skeletal reorganization, such as a 1,2-hydrogen shift, is often necessary.^9–12^ The Pauson–Khand reaction therefore remains the dominant archetype of [2 + 2 + 1] cycloaddition (Figure 1b).^13–15^

**Figure 1:**
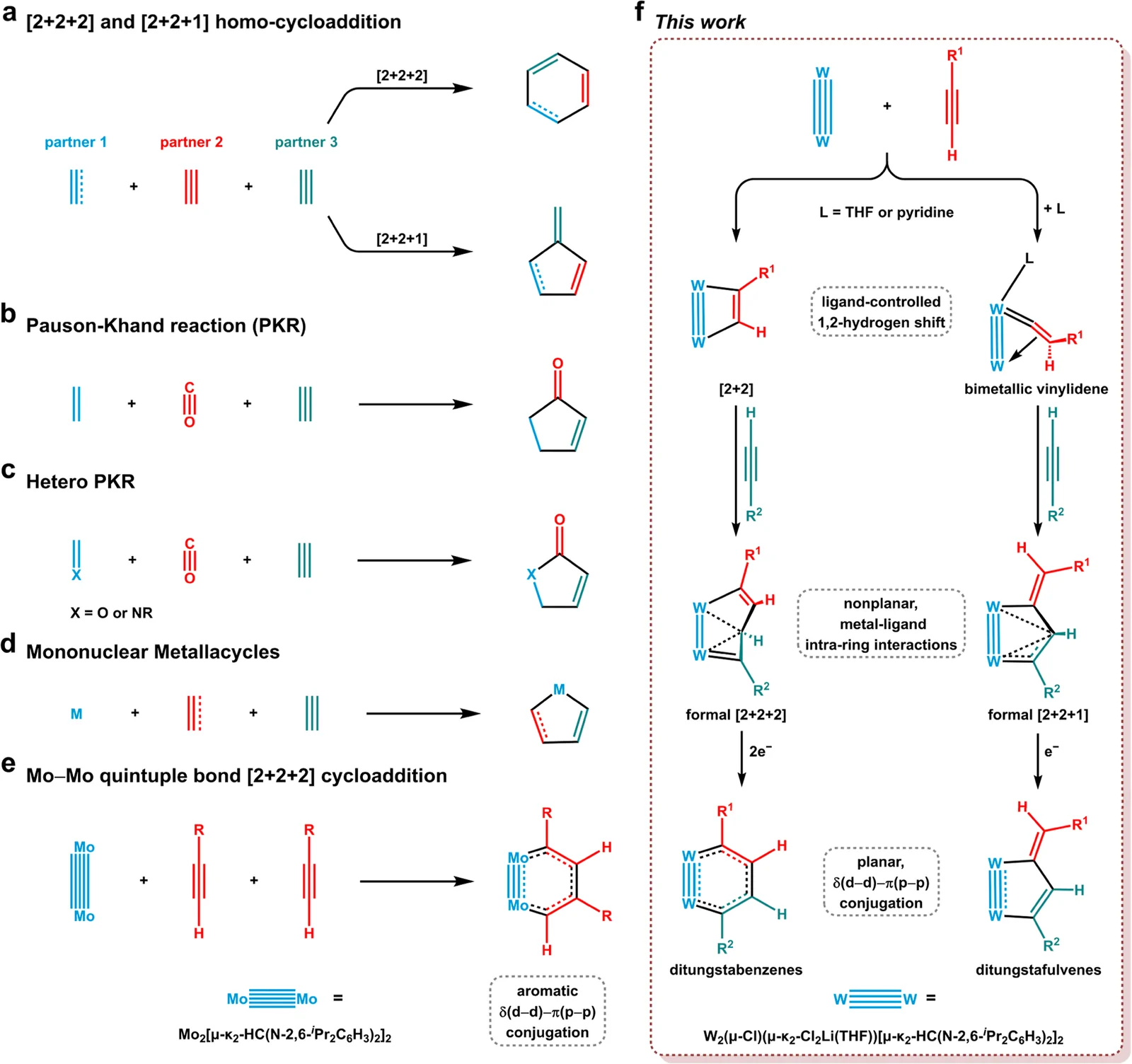
(a) [2 + 2 + 2] and [2 + 2 + 1] cycloaddition by unsaturated hydrocarbons. (b) Pauson–Khand reaction. (c) Hetero PKR. (d) Synthesis of mononuclear metallacycles (e) [2 + 2 + 2] cycloaddition by Mo–Mo quintuple bond. (f) [2 + 2 + 2] and [2 + 2 + 1] cycloaddition by W–W quadruple bonds (this work).

Beyond purely organic π fragments, transition-metal complexes can also participate directly in cycloaddition chemistry to generate metallacycles, processes that are fundamental to many catalytic transformations (Figure 1d). For example, oxidative coupling of two alkynes or alkenes at a metal center affords metallacyclopentadienes or metallacyclopentenes, respectively.^16^ In parallel, metallaaromatics constitute an important class of metallacyclic compounds in which aromaticity is delocalized over both metal and carbon atoms.^17–20^ However, transition-metal-based metallaaromatics remain largely confined to mononuclear systems, where aromatic stabilization typically arises from interactions between the d orbitals of a single metal center and the organic π framework, and a general synthetic strategy for their construction is still lacking.

We previously extended this concept by exploiting the δ orbitals of metal–metal multiple bonds rather than relying solely on mononuclear metal centers.^21^ In particular, we showed that an amidinate-stabilized Mo–Mo quintuple bond undergoes [2 + 2 + 2] cycloaddition with two molecules of terminal alkynes to furnish a new class of metalloaromatic compounds (Figure 1e), in which a multiply bonded Mo–Mo unit is embedded in a six-membered C_4_Mo_2_ aromatic ring. Remarkably, this transformation proceeds without an external catalyst, because the quintuply bonded Mo_2_ unit serves simultaneously as both the cycloaddition partner and the reactive center. The process is also completely regioselective, with the two alkyne molecules coupling in a head-to-tail manner across the Mo–Mo bond. These findings established that the Mo–Mo quintuple bond behaves as a close analogue of a C–C triple bond in cycloaddition chemistry, while the resulting δ­(d–d)–π­(p–p) conjugation actively participates in aromatic delocalization.

Given the striking analogy between the cycloaddition reactivity of the Mo–Mo quintuple bond and that of alkynes, we next asked whether a metal–metal quadruple bond could likewise function as a cycloaddition partner toward alkynes. This question is especially compelling because quadruply bonded dinuclear complexes constitute a large and well-established family, dating back to the first quadruply bonded dirhenium complex, [Re_2_Cl_8_]^2–^, reported by Cotton and co-workers in 1964,^22^ yet their chemistry has remained comparatively underdeveloped owing to the kinetic inertness imposed by their dominant tetragonal coordination environment. At the same time, a quadruply bonded dinuclear complex contains only a single δ component and might therefore display reactivity more akin to that of an alkene. Such behavior would be notable because although alkenes are established two-electron partners in cycloaddition chemistry, they are generally less competent than alkynes in [2 + 2 + 2] annulation. In this regard, the W–W quadruple bond appeared especially attractive, as its electronic structure differs fundamentally from that of the Mo–Mo quintuple bond owing to differences in metal–metal bonding interactions and oxidation state.^23,24^ These distinctions led us to anticipate divergent reaction pathways and regioselectivity. Such an expectation is consistent with the contrasting behavior already known for related systems: even within closely similar bonding and ligand frameworks, Mo–Mo triple bonds typically undergo coordination or mediate C–C coupling with alkynes, whereas W–W triple bonds are distinguished by their pronounced tendency toward alkyne metathesis, giving tungsten alkylidyne complexes.^25–27^

Despite these precedents, the corresponding cycloaddition chemistry of W–W quadruple bonds has remained largely unexplored.^27,28^ More broadly, quadruply bonded complexes across diverse transition-metal families have not been shown to engage in multicomponent cycloadditions analogous to those of organic unsaturated molecules; instead, their reactions with alkynes are generally restricted to simple addition events,^27,29^ with only limited examples displaying catalytic activity toward alkyne trimerization or polymerization.^30^ Although recent efforts have sought to access W–W quadruply and quintuply bonded complexes in more reactive conformations analogous to those known for chromium and molybdenum systems, these attempts have most often culminated in ligand activation or further reduction to alternative thermodynamically stable states.^31,32^

Because cycloaddition chemistry has traditionally been associated with classical organic unsaturated molecules, extending the scope of cycloaddition partners to a low-coordinate W–W quadruple bond would represent a significant conceptual advance. Herein, we report a strategy for accessing a series of amidinate-supported low-coordinate W–W multiply-bonded complexes featuring a maximum metal–metal bond order of 4 within a [W–W]^4+^ core. Reactivity studies reveal alkene-type cycloaddition behavior, in which the W–W quadruple bond mimics an alkene in both formal [2 + 2 + 2] and [2 + 2 + 1] cycloaddition pathways. In a notable departure from the Mo–Mo quintuple bond, the W–W quadruple bond undergoes [2 + 2 + 2]­cycloaddition with distinct tail-to-tail regioselectivity, allowing the direct [2 + 2]­cycloadduct intermediate to be isolated successfully. Alternatively, the reaction pathway can be diverted toward the stabilization of vinylidene intermediates, in which coordination of σ-donating solvents plays a crucial role. These vinylidene species subsequently react with alkynes along a formal [2 + 2 + 1] pathway, representing a rare example of controllable divergence between formal [2 + 2 + 2] and [2 + 2 + 1] cycloadditions. Importantly, sequential reduction of the resulting nonplanar C_4_W_2_ and C_3_W_2_ frameworks populates the W–W δ manifold and drives planarization through enhanced δ­(d–d)–π­(p–p) conjugation. This electronic reorganization ultimately furnishes a benzene-like ditungsten aromatic system and a bimetallic fulvene analogue, respectively.

## Result and Discussion

### Synthesis of Amidinate-Supported W–W Multiple Bonds

Based on our established studies of the quintuply bonded Cr and Mo systems,^23,24,33,34^ we continue to pursue the amidinate-stabilized ditungsten precursor as a robust platform for investigating W–W multiple-bond reactivity. As illustrated in Figure 2, the complex [Li­(THF)_4_]­[{WCl_2_(μ-κ^2^-HC­(N-2,6-^*i*^Pr_2_C_6_H_3_)_2_)}_2_] (**1**)^31^ was thus synthesized by treating an equimolar mixture of W_2_Cl_4_(μ-Cl)_2_(κ^2^-DME)_2_ ^35^ (DME = 1,2-dimethoxyethane) and KC_8_ in THF with the corresponding lithiated amidinate, affording cherry-pink powder **1** in 62% yield. Single-crystal X-ray diffraction (Figure S1) confirmed its formulation and revealed that the anion of **1** adopts a *D*_2*h*_-symmetric paddlewheel structure in which two tungsten atoms are bridged by two amidinate ligands, and each W atom bears two terminal equatorial chlorides. Accordingly, **1** contains a W_2_^5+^ core. The W–W bond length of 2.2535(4) Å falls within the range reported for W–W triple and quadruple bonds.^27,28,35–38^ Magnetic measurements indicate that **1** contains one unpaired electron and exhibits a solution magnetic moment of 2.17 μ_B_ (Evans method),^39,40^ consistent with a formal W–W bond order of 3.5.

**Figure 2:**
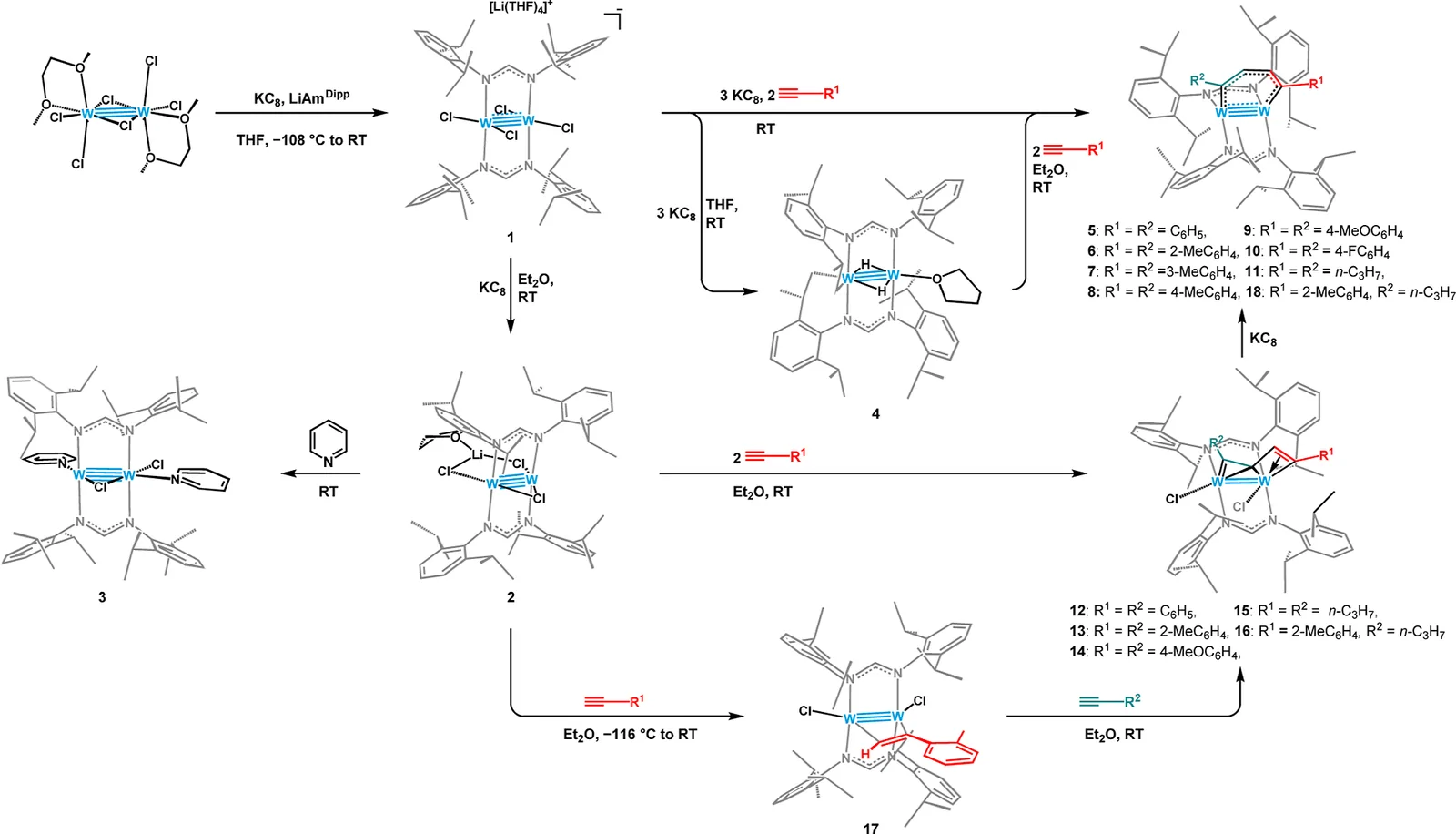
Synthesis of multiply bonded ditungsten amidinates and subsequent [2 + 2 + 2] cycloaddition reactions with alkynes.

Subsequent reduction of **1** with 2 equiv of KC_8_ in diethyl ether (Et_2_O) afforded the orange–yellow complex W_2_(μ-Cl)­(μ-κ^2^-Cl_2_Li­(THF))­[μ-κ^2^-HC­(N-2,6-^*i*^Pr_2_C_6_H_3_)_2_]_2_ (**2**) in 85% yield. The molecular structure of **2** was determined by single-crystal X-ray crystallography and NMR spectroscopy. As presented in Figure S2, 2 adopts a *C*_2*v*_-symmetric paddlewheel conformation, where the two tungsten atoms are bridged by two amidinate ligands, a single chloride ligand, and a μ-κ^2^-Cl-Li­(THF)-Cl unit, giving rise to a W_2_^4+^ core. The W–W bond length of 2.2017(3) Å, characteristic of a W–W quadruple bond and significantly shorter than that in **1**, is consistent with an increase in the formal W–W bond order to 4.^27,28,31,32^ The ^1^H NMR spectrum reveals that two amidinates are magnetically equivalent and that the complex contains one coordinated THF molecule.

The reactivity of **2** was subsequently examined. Addition of 2 equiv of pyridine to an ethereal solution of **2** elicited a quick color change to green. The (THF)­LiCl fragment was replaced by a pyridine molecule to afford the malachite–green complex W_2_Cl­(μ-Cl)­(NC_5_H_5_)_2_[μ-κ^2^-HC­(N-2,6-^*i*^Pr_2_C_6_H_3_)_2_]_2_ (**3**) in 71% yield. X-ray quality crystals of **3** were obtained from diethyl ether at −35 °C. Single-crystal X-ray diffraction revealed that **3** also adopts a paddlewheel structure, where one chloride and one pyridine ligand occupy one face of the W_2_[μ-κ^2^-HC­(N-2,6-^*i*^Pr_2_C_6_H_3_)_2_]_2_ fragment, while a bridging chloride spans two metal centers on the opposite face (Figure S3). Consequently, **3** contains a quadruply bonded W_2_^4+^ core. In addition, a pyridine ligand coordinates axially to one tungsten atom, giving **3** an overall *C*_1_-symmetric structure. The W–W bond length, 2.2393(2) Å is elongated relative to **2**, consistent with the axial coordination of pyridine. The unusually long W–N­(axial pyridine) bond length 2.442(3) Å, relative to the W–N­(equatorial pyridine) bond length of 2.132(3) Å,^41–43^ indicates weak axial coordination, which may point to potential lability of the axial pyridine ligand in solution.

In view of the recognition of amidinate-supported quintuply bonded dichromium and dimolybdenum complexes [M_2_(μ-κ^2^-HC­(N-2,6-^*i*^Pr_2_C_6_H_3_)_2_)_2_] (M = Cr, Mo),^23,24,33,34^ the most anticipated reactivity of **1** and **2** is their reduction to a ditungsten complex featuring a W–W quintuple bond, exemplified by [W_2_(μ-κ^2^-HC­(N-2,6-^*i*^Pr_2_C_6_H_3_)_2_)_2_]. In this context, slow addition of 3 equiv of KC_8_ to a THF solution of **1** at room temperature instead afforded the moss–green complex [W_2_(THF)­(μ-H)_2_(μ-κ^3^-HC­(N-2-CH­(CH_2_)­(CH_3_)-6-^*i*^PrC_6_H_3_)­(N-2,6-^*i*^Pr_2_C_6_H_3_))_2_] (**4**) in 91% yield, showing double intramolecular C–H activation. This outcome is consistent with the theoretical prediction that [W_2_(μ-κ^2^-HC­(N-2,6-^*i*^Pr_2_C_6_H_3_)_2_)_2_] is more reducing than its lighter Cr and Mo congeners.^44^ The formulation of **4** was established by single-crystal X-ray crystallography and NMR spectroscopy, and its molecular structure is depicted in Figure S4. Interestingly, each of both amidinato ligands undergoes cyclometalation with the W2 atom through C–H activation of one isopropyl substituent, resulting in C–H addition across the W–W bond. Consequently, the W2 atom is equatorially coordinated by two –CH_2_ groups in addition to the two N donors from the amidinate ligands. Two hydrido ligands also bridge two tungsten centers in the equatorial plane, as confirmed by both X-ray diffraction and, more definitively, NMR spectroscopy. In the ^1^H NMR spectrum, the resonance attributable to the 2 equiv bridging hydrides appears at −2.23 ppm with two sets of tungsten satellites (^1^ *J*_W–H_ = 216.5 Hz, ^1^ *J*_W–H_ = 52.0 Hz), consistent with their spanning two tungsten centers in an unsymmetrical fashion. Moreover, these data indicate that the equatorial THF ligand bound to W1 exhibits a fluxional behavior. Overall, a W_2_^6+^ core is present in **4** with a W–W bond length of 2.3285(3) Å, typical for a W–W triple bond.

The characterization of **4** suggests the involvement of the transient quintuply bonded ditungsten amidinate [W_2_(μ-κ^2^-HC­(N-2,6-^*i*^Pr_2_C_6_H_3_)_2_)_2_]. This inference is further supported by theoretical predictions that, compared to its lighter Cr and Mo congeners, this species is highly reducing due to pronounced relativistic effects.^44^ Moreover, facile polarization of the two δ orbitals in the transition state renders the metal–metal quintuple bond highly reactive toward H–H and C–H bond cleavage reactions.^45^

### [2 + 2 + 2] Cycloaddition Reactions of W–W Multiple Bonds

Reactions of **4** and terminal alkynes were first explored. This investigation was motivated by the efficient [2 + 2 + 2] cycloaddition previously observed in the dimolybdenum system Mo_2_[μ-κ^2^-HC­(N-2,6-^*i*^Pr_2_C_6_H_3_)_2_]_2_. By utilizing complex **4** as a surrogate for a quintuply bonded ditungsten species, we sought to evaluate if the ditungsten framework could likewise accommodate a [2 + 2 + 2] manifold while expecting its distinct electronic profile to drive a unique reaction outcome. The reaction of **4** with 2 equiv of phenylacetylene (PhC≡CH) in Et_2_O proved to be complex, yielding multiple products. Among them, the brown [2 + 2 + 2] cycloadduct W_2_(μ-κ^2^-1,4-Ph_2_C_4_H_2_)­[μ-κ^2^-HC­(N-2,6-^*i*^Pr_2_C_6_H_3_)_2_]_2_ (**5**) was the only identifiable species and was isolated in 17% yield. The ^1^H NMR spectrum of **5** revealed that this reaction displays regioselectivity distinct from that observed for [Mo_2_(μ-κ^2^-HC­(N-2,6-^*i*^Pr_2_C_6_H_3_)_2_)_2_], as **5** is *C*_2*v*_-symmetric, whereas the corresponding Mo_2_ products are at most *C*_*s*_-symmetric. The newly formed six-membered ditungstacycle C_4_W_2_ in **5** displays aromatic character, evidenced by a downfield-shifted singlet at 8.56 ppm (H4 and H5) in the ^1^H NMR spectrum and two singlets at 230.8 ppm (two α-carbon atoms) and 137.8 ppm (two β-carbon atoms) in the ^13^C NMR spectrum.

The regiochemistry around the newly formed C_4_W_2_ ring was unambiguously confirmed by X-ray crystallography (Figure 3a). Compound **5** crystallizes in the triclinic system (space group *P*1̅) and features two crystallographically independent molecules (**5A** and **5B**) in the unit cell. Both **5A** and **5B** feature three rings and thus exhibit a lantern geometry. The six-membered ditungstacycle C_4_W_2_ is constructed through tail-to-tail coupling of two phenylacetylene molecules across the W–W bond. Both C_4_W_2_ rings in **5A** and **5B** are essentially planar, as indicated by the sums of their internal bond angles, 718.8(4) and 719.9(5)°, respectively, which are close to the ideal 720° for a hexagon. Both W–W bonds in **5A** and **5B** measure 2.2377(3) and 2.2404(3) Å, respectively, that fall within the reported ranges for W–W quadruple bonds (2.155–2.375 Å) and triple bonds (2.233–2.480 Å), suggesting a formal bond order of approximately 3.5. The four C–W bonds span 1.989(4)–2.033(4) Å,^46–48^ consistent with distances intermediate between single and double bonds, in line with the downfield chemical shift observed at 230.8 ppm for the C–W carbons in the ^13^C NMR spectrum, indicating significant multiple-bond character. The six C–C bond lengths of the two C_4_W_2_ rings in **5A** and **5B** lie in the range of 1.336(8)–1.425(8) Å,^49^ also intermediate between canonical single and double bond values. Furthermore, the chemical shift of the two C–H carbons appeared at 137.8 ppm in the ^13^C NMR spectrum. Collectively, these structural and spectroscopic features provide compelling evidence for extensive delocalization within the C_4_W_2_ framework and support its assignment as an aromatic system.

**Figure 3:**
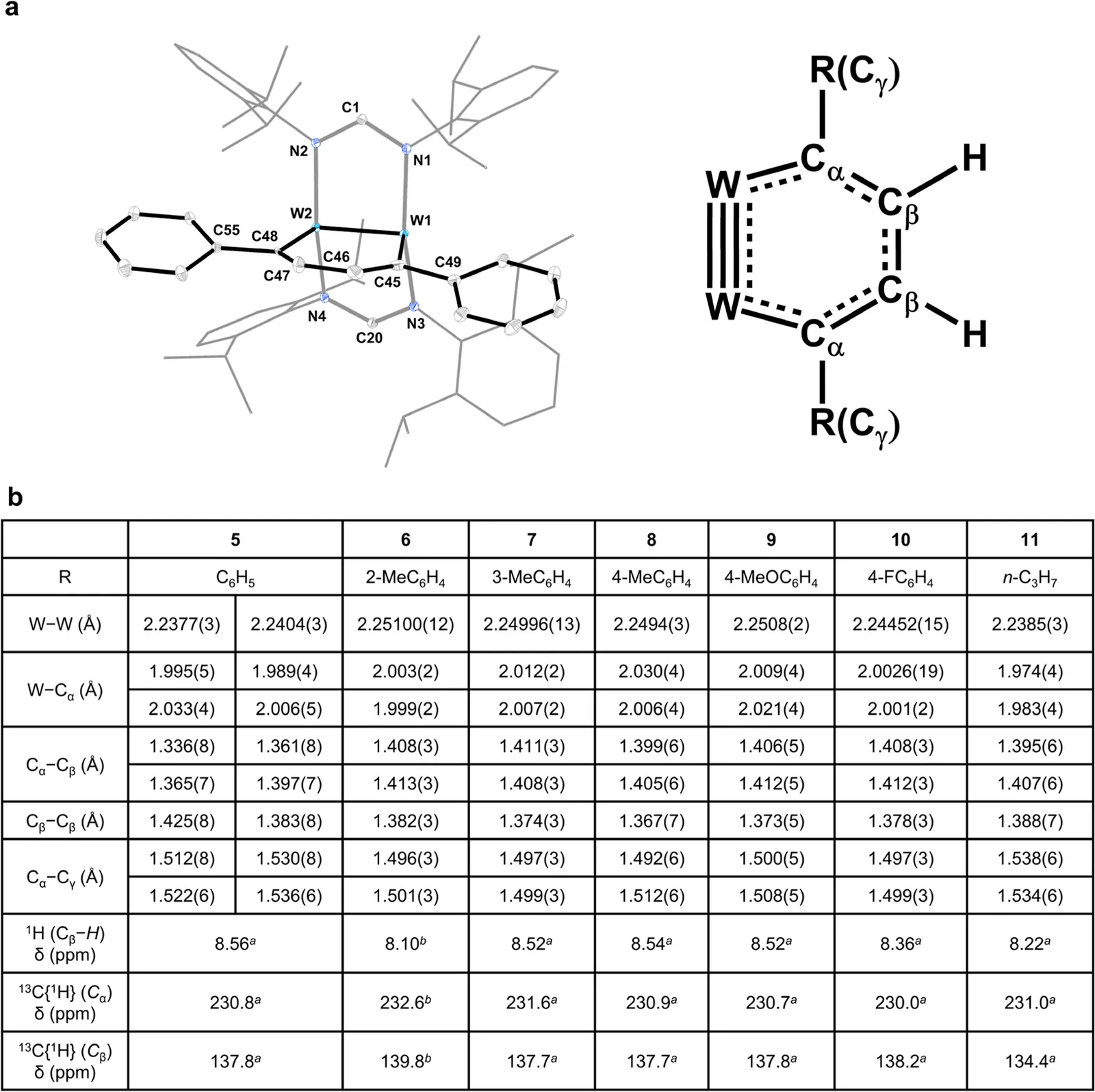
(a) The solid-state molecular structure of **5** (thermal ellipsoids at 30% probability. Hydrogen atoms are omitted for clarity). (b) Selected crystallographic data and NMR chemical shifts for **5**–**11** (C_6_D_6_, 500 MHz). ^a^298 K. ^b^333 K.

Compound **5** can also be obtained in a 63% yield by adding 2 equiv of PhCCH to a mixture of **1** and 4 equiv of KC_8_ in Et_2_O. This synthetic strategy also proved general, affording six additional derivatives of the type [W_2_(μ-κ^2^-1,4-R_2_C_4_H_2_)­(μ-κ^2^-HC­(N-2,6-^*i*^Pr_2_C_6_H_3_)_2_)_2_] in good to excellent yields (60–89%). This series includes R = 2-MeC_6_H_4_ (**6**), 3-MeC_6_H_4_ (**7**), 4-MeC_6_H_4_ (**8**), 4-MeOC_6_H_4_ (**9**), 4-FC_6_H_4_ (**10**), *n*-C_3_H_7_ (**11**). All compounds were successfully characterized by NMR spectroscopy and X-ray crystallography. The ^1^H and ^13^C chemical shifts and structural parameters within their planar C_4_W_2_ rings are summarized in Figure 3b, respectively. Notably, the C_4_W_2_ frameworks in **5**–**11** exhibit closely similar electronic structures despite variations in the steric and electronic properties of the appended substituents.

Considering the head-to-tail coupling characteristic of Mo–Mo quintuple bond congeners, the distinct tail-to-tail regioselectivity observed here, alongside the predicted instability of the highly reducing W–W quintuple bond,^44^ indicates that **5**–**11** do not likely arise from a direct reaction between the quintuply bonded ditungsten amidinate W_2_[μ-κ^2^-HC­(N-2,6-^*i*^Pr_2_C_6_H_3_)_2_]_2_ and 2 equiv of RCCH. Complementary to these findings, we performed the reaction of **2** with 2 equiv of phenylacetylene (PhCCH) in Et_2_O, which furnished an olive-green [2 + 2 + 2] cycloadduct, W_2_Cl_2_[μ-η^2^-η^2^-1,4-Ph_2_C_4_H_2_]­[μ-κ^2^-HC­(N-2,6-^*i*^Pr_2_C_6_H_3_)_2_]_2_ (**12**), isolated in 58% yield.

The molecular structure of **12** was deciphered by X-ray crystallography and is presented in Figure 4a. The structure reveals that two PhCCH molecules also couple in a tail-to-tail fashion across the W–W quadruple bond. In stark contrast to the planar C_4_W_2_ ring observed in **5**, the C_4_W_2_ core in **12** adopts a distinctly nonplanar conformation. The μ-η^2^-η^2^-1,4-Ph_2_C_4_H_2_ ligand binds the W_2_ unit in an unusual bis­(η^2^-vinyl) fashion. The C1–C2 bond bridges the two tungsten centers and lies nearly parallel to the W–W bond, whereas the C3–C4 bond coordinates exclusively to W2. The W1–C1 and W2–C4 bond lengths are 1.892(6) and 2.049(8) Å, respectively, with the former consistent with a localized W=C double bond and the latter intermediate between single- and double-bond character. The C1–C2 and C2–C3 bonds correspond to typical C–C single bonds at 1.504(8) and 1.497(8) Å, while the C3–C4 bond is significantly shorter at 1.344(10) Å, consistent with partial double-bond character.^49^ The W–W bond length of 2.4281(4) Å suggests a formal double-bond assignment. The unsymmetric coordination environments around the two tungsten centers are evident from the orientations of the chloride ligands. The Cl2 atom tilts toward W1 at a distance of 3.043(2) Å, giving a W1–W2–Cl2 bond angle of 78.59(4)°. This distortion in turn pushes Cl1 away from W2, as evidenced by the enlarged W2–W1–Cl1 angle of 139.16(4)°.

**Figure 4:**
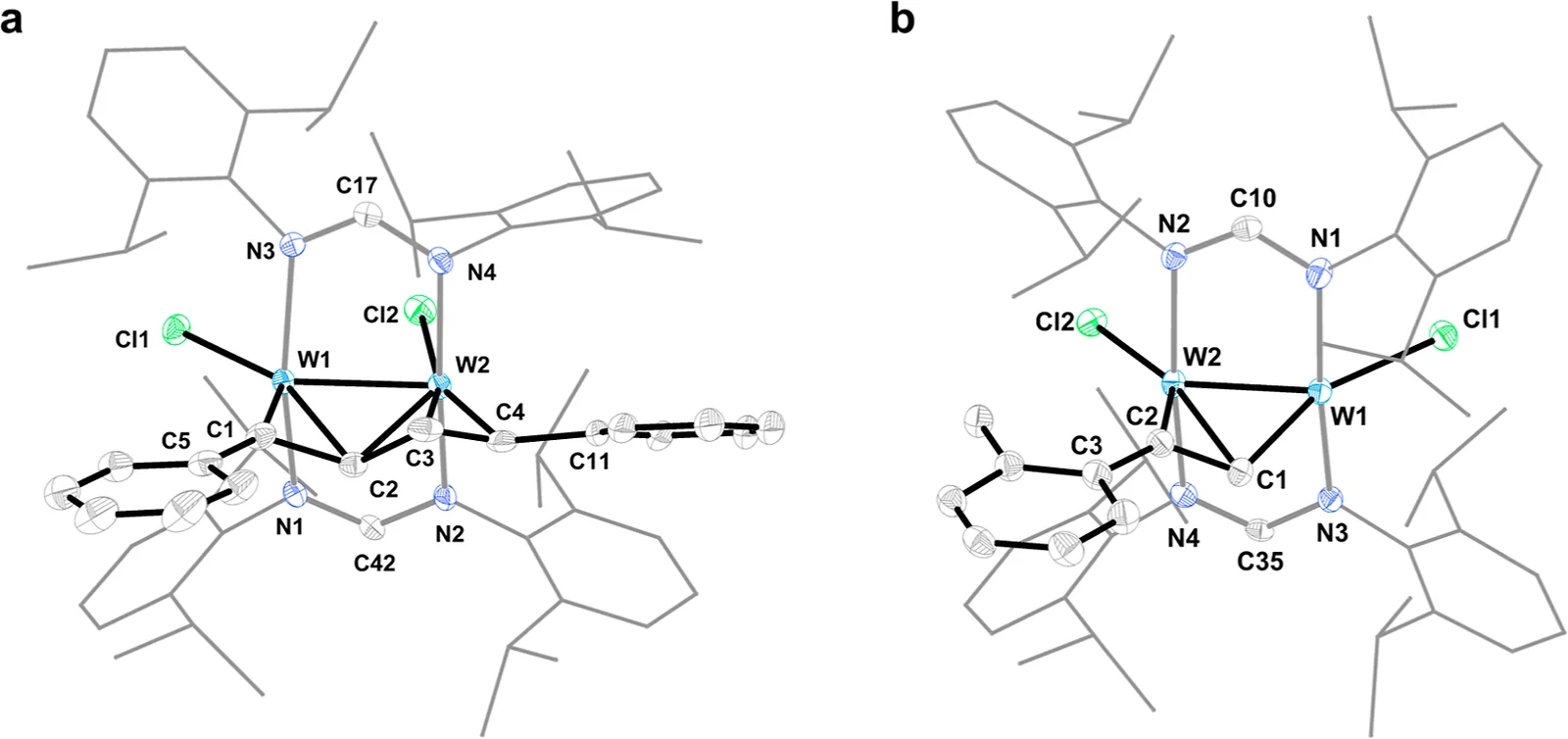
(a) The solid-state molecular structure of **12**. (b) The solid-state molecular structure of **17** (thermal ellipsoids at 30% probability. Hydrogen atoms are omitted for clarity).

Three structural analogues of **12**, W_2_Cl_2_[μ-η:^2^η^2^-1,4-R_2_C_4_H_2_]­[μ-κ^2^-HC­(N-2,6-^*i*^Pr_2_C_6_H_3_)_2_]_2_, were subsequently synthesized and isolated in good yields (51–74%) by the same method, where R = 2-MeC_6_H_4_ (**13**), 4-MeOC_6_H_4_ (**14**), and *n*-C_3_H_7_ (**15**). All these three complexes were structurally characterized by NMR spectroscopy and X-ray crystallography as well. Along with displaying similar spectral features, **13**–**15** (Figures S14–S16) are essentially isostructural with **12**. Subsequent reduction of **12**–**15** with 2 equiv of KC_8_ afforded the corresponding products **5**, **6**, **9**, and **11**, respectively. This outcome strongly suggests that aromatization provides the key thermodynamic driving force for the KC_8_-mediated transformations, stabilizing the reduced products through delocalization within the C_4_W_2_ framework. Moreover, these results provide pivotal mechanistic insights into the [2 + 2 + 2]­cycloaddition directly obtained from **4** with two alkynes. Specifically, a quadruple-bonded intermediate featuring a single reversed C–H activation likely serves as the crucial species governing the observed tail-to-tail selectivity rather than a transient quintuple-bonded intermediate arising from the complete reversal of both C–H activations. In this proposed pathway, the first alkyne coordinates to the metal center either prior to or following the first C–H bond reversal. After the second alkyne undergoes oxidative coupling with the first, the remaining C–H-activated moiety is subsequently reversed to furnish the final aromatic metallacycle framework.

A theoretical mechanistic study of the [2 + 2 + 2] cycloaddition between the Mo–Mo quintuple bond and two molecules of terminal alkyne has suggested that the reaction proceeds through sequential [2 + 2] and [4 + 2] cycloaddition steps.^50^ However, the [2 + 2] intermediates have not been experimentally realized. To gain further insight into the mechanism underlying the formation of **12**–**15**, we successfully prepared the unsymmetric cycloadduct W_2_Cl_2_[μ-η:^2^η^2^-1–2-MeC_6_H_4_-4-*n*-C_3_H_7_C_4_H_2_]­[μ-κ^2^-HC­(N-2,6-^*i*^Pr_2_C_6_H_3_)_2_]_2_ (**16**) through coupling of three different components: **2**, 2-MeC_6_H_4_CCH, and 1-pentyne. During this reaction, the intermediate [2 + 2] cycloadduct W_2_Cl_2_[μ-η^2^-2-MeC_6_H_4_CCH]­[μ-κ^2^-HC­(N-2,6-^*i*^Pr_2_C_6_H_3_)_2_]_2_ (**17**) was successfully isolated from the reaction of **2** with 1 equiv of the bulkier alkyne, 2-methylphenylacetylene. Compound **17** was recognized by ^1^H NMR spectroscopy, where the resonance for the terminal alkyne proton is significantly downfield-shifted to 16.0 ppm, indicative of coordination of the alkyne molecule to the W_2_ core. The solid-state molecular structure of **17** (Figure 4b) reveals that the alkyne bridges the W_2_ unit, as evidenced by W1–C1 and W2–C2 bond lengths of 2.136(9) and 2.005(7) Å, respectively. These metrics are consistent with W–C single bonds, while the C1–C2 bond length of 1.340(9) Å indicates localized double-bond character. The most salient structural feature is the near-parallel alignment of the C1–C2 and W1–W2 bonds, as reflected by the small C1–W1–W2–C2 dihedral angle of 16.3(3)°. In contrast to [2 + 2] cycloadducts formed across metal–metal bonds often display highly skewed geometries, with torsion angles ranging from 23° to 34°,^21,51–54^ complex **17** adopts a more nearly eclipsed conformation. The 2-methylphenyl substituent is likewise oriented nearly parallel to the W–W axis, as evidenced by W1–W2–C2–C3 dihedral angle of 178.1(8)°. This arrangement likely reflects steric repulsion between the bulky diisopropyl substituents of the amidinate ligands and the 2-methylphenyl group.

Subsequent addition of 1-pentyne to **17** afforded the unsymmetric [4 + 2] product **16**, formed exclusively in a tail-to-tail manner. The solid-state molecular structure of **16** provides direct insight into the coupling mechanism, revealing that the second incoming alkyne (1-pentyne) forms the W=C double bond at the W1 center. Specifically, the C1 atom bearing the *n*-propyl substituent displays a short W1–C1 bond length of 1.882(4) Å, while the adjacent C1–C2 bond is elongated to 1.514(4) Å, consistent with single-bond character. In contrast, the initially coordinated alkyne fragment derived from **17** retains pronounced C=C double-bond character, as evidenced by the C3–C4 bond length of 1.382(6) Å. These structural features support a sequence in which the first alkyne forms the [2 + 2] intermediate **17**, after which the second alkyne is incorporated selectively to furnish the six-membered ditungstacycle, C_4_W_2_. Notably, complex **16** is also redox-active; KC_8_ reduction affords the unsymmetric aromatic ditungsten system W_2_[μ-κ^2^-1–2-MeC_6_H_4_-4-*n*-C_3_H_7_C_4_H_2_]­[μ-κ^2^-HC­(N-2,6-^*i*^Pr_2_C_6_H_3_)_2_]_2_ (**18**). This transformation further demonstrates that redox-induced planarization is a general feature of these ditungsten metallacycles, regardless of the substituent pattern.

### [2 + 2 + 1] Cycloaddition Reactions of W–W Quadruple Bonds

In the course of synthesizing **16**, we observed that the reaction solvent can effectively alter the reaction pathway. Specifically, the presence of THF led to the observation of an alternative product distinct from **16**, yielding a 1:1 mixture of both species, as determined by ^1^H NMR spectroscopy. The ^1^H NMR spectrum of this new species exhibited a signal pattern markedly different from that of the [2 + 2 + 2] congener series. Subsequent isolation via recrystallization enabled single-crystal X-ray diffraction analysis, which elucidated this isomeric structure as W_2_Cl_2_[μ-η:^2^η^2^-1-*n*-C_3_H_7_-4–2-MeC_6_H_4_(H)–C_4_H]­[μ-κ^2^-HC­(N-2,6-^*i*^Pr_2_C_6_H_3_)_2_]_2_ (**19**) (Figure 5b).

**Figure 5:**
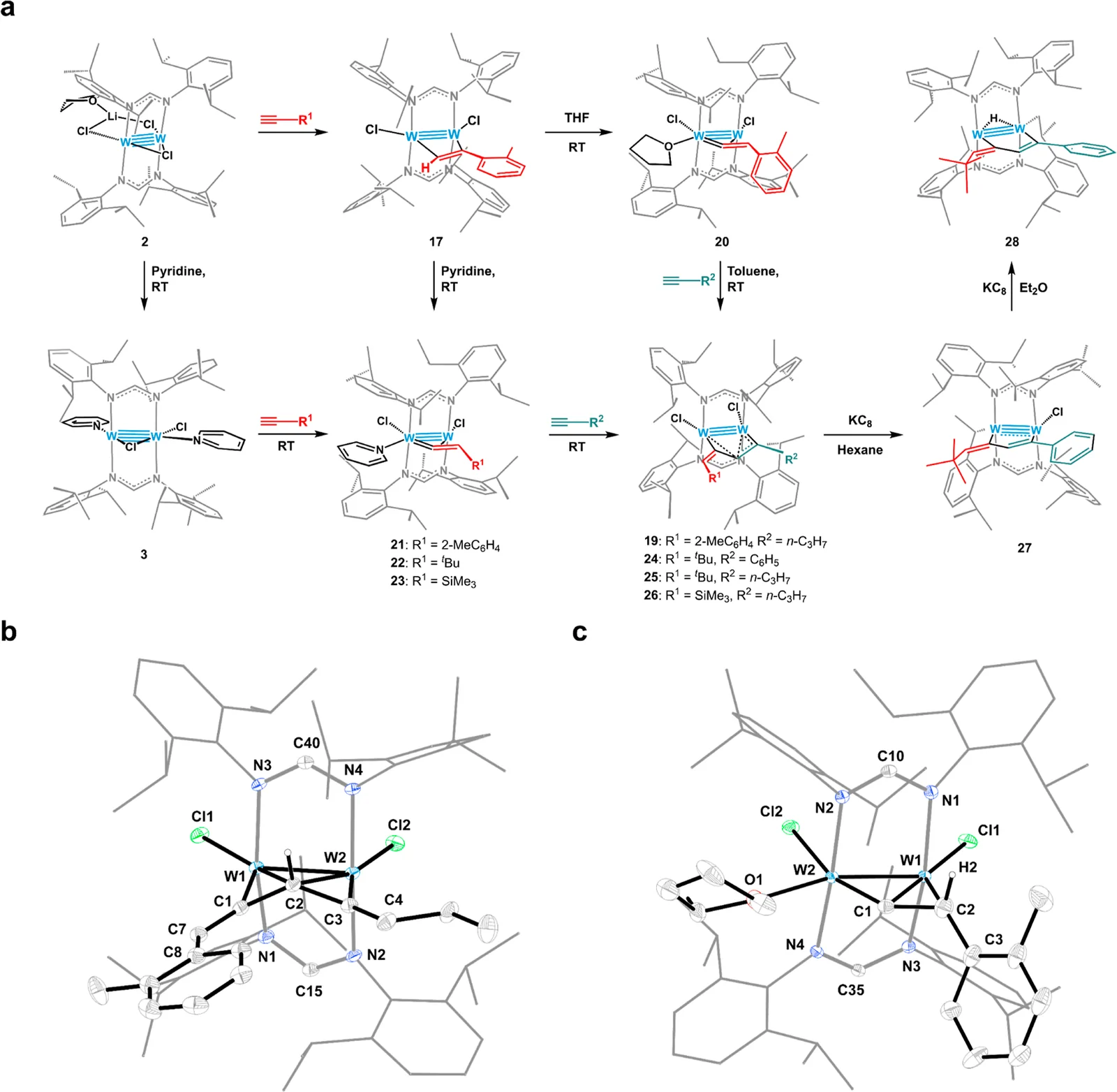
(a) Synthesis of **19**–**28**. (b) The solid-state molecular structure of **19**. (c) The solid-state molecular structure of **20** (thermal ellipsoids at 30% probability. Hydrogen atoms are omitted for clarity).

In complex **19**, a five-membered ditungstacycle, C_3_W_2_, is formed. The major difference compared to the [2 + 2 + 2] congener **16** is the W–W core; specifically, **19** exhibits a significantly shorter W–W bond length of 2.3533(5) Å, which is in line with a formal W≡W triple bond.^28^ This shortening is attributable to the minimal interaction between the exocyclic C1=C2 unit and the tungsten centers. While the C1=C2 moiety does not show notable interactions with the W–W center, C7 still displays an sp^2^-hybridized geometry with a C1–C7–C8 angle of 127.7(3)°, indicating the occurrence of a 1,2-hydrogen shift from C1 to C7. The cyclic C_3_W_2_ core adopts an envelope conformation, wherein the C1–W1–W2–C3 sequence is essentially planar (torsion angle = 4.7(1)°), while C2 lies out of this mean plane, as indicated by the W1–W2–C3–C2 torsion angle of 20.6(2)°. This puckered C2 atom engages in distinctive bridging interactions with both tungsten centers (W1–C2 = 2.426(3) Å; W2–C2 = 2.333(3) Å), even though the metric parameters already indicate a W1–C1 single bond (2.059(2) Å) and a W2=C3 double bond (1.927(2) Å).

To elucidate the crucial role of THF in this chemodivergent reactivity and to gain control over the formation of **19**, we evaluated both **16** and its [2 + 2] precursor **17**. While **16** remains stable in THF at room temperature, **17** reacts rapidly with THF, evidenced by an immediate color change from green to brownish-yellow, affording the vinylidene complex W_2_Cl_2_(THF)­[μ-η:^1^η^2^-CC­(H)-2-MeC_6_H_4_]­[μ-κ^2^-HC­(N-2,6-^*i*^Pr_2_C_6_H_3_)_2_]_2_ (**20**). The ^1^H NMR spectrum of **20** indicates a marked conformational change relative to the [2 + 2] complex **17**; most notably, the alkyne proton resonance shifts significantly upfield from 16.0 ppm in **17** to approximately 10.2 ppm in **20**.

The solid-state structure confirms this structural reorganization. As illustrated in Figure 5c, compound **20** features an unusual bent vinylidene moiety,^55^ in which the α-carbon (C1) is terminally bound to W2. The W2–C1 bond length of 1.911(4) Å is noticeably shorter than the corresponding W–C bonds in the [2 + 2] intermediate **17**, yet still falls within the range expected for bonds with mixed single- and double-bond character.^46–48^ This metric is associated with a 29.7(1)° tilt of the vinylidene ligand toward W1, which consequently diminishes the W2–C1 π-bonding interaction. Concomitantly, the C1–C2 bond (1.430(6) Å) coordinates side-on to W2, thereby providing additional stabilization to the vinylidene unit. Consistent with this binding mode, the W1–C2 bond (2.316(4) Å) is substantially longer than the W1–C1 bond (2.163(4) Å). A striking difference between **20** and **17** lies in the orientation of the 2-methylphenyl group: whereas it lies nearly parallel to the W–W axis in **17**, it adopts an almost perpendicular orientation in **20**. The W2–W1–C2–C3 dihedral angle of 122.9(8)° is consistent with an sp^3^-hybridized C2 center, further supporting the occurrence of a 1,2-hydrogen shift. Finally, the axial coordination of a weakly bound THF ligand to W2 (W2–O1 = 2.374(3) Å) is consistent with the enhanced electron-withdrawing character of the vinylidene group.

The isolation of complex **20** has rationalized the formation of **19**. In general, metal vinylidene complexes are renowned for their metallaallene-like character featuring an M=C=CR_2_ framework, wherein the M=C linkage of mononuclear vinylidenes is capable of undergoing cycloaddition reactions with unsaturated organic molecules to yield four-membered metallacycles.^56,57^ Analogous observations regarding the relationship between complexes **19** and **20**, indeed shed light on the feasible construction of five-membered bimetallacycles.

Subsequent treatment of complex **20** with 1-pentyne indeed triggered a [3 + 2] cycloaddition with W=C, yielding product **19** without any detectable formation of the [4 + 2] isomer **16**. This critical observation inspired us to conduct a deeper investigation into the [2 + 2 + 1] cycloadditions mediated by ditungsten moieties and their corresponding vinylidene complexes. While THF possesses sufficient electron-donating ability to transform **17** into the tungsten vinylidene **20**, the use of pyridine provided superior reactivity in these transformations, allowing for the accommodation of even stronger electron-donating groups on the vinylidene moiety. The addition of pyridine to the reaction of **2** with terminal alkynes RCCH (R = 2-MeC_6_H_4_, ^*t*^Bu, and SiMe_3_), or the direct reaction with the pyridine-coordinated species **3**, afforded exclusively the corresponding vinylidene complexes W_2_Cl_2_(C_5_H_5_N)­[μ-η:^1^η^2^-CC­(H)-R]­[μ-κ^2^-HC­(N-2,6-^*i*^Pr_2_C_6_H_3_)_2_]_2_ (R = 2-MeC_6_H_4_ (**21**), ^*t*^Bu (**22**), and SiMe_3_ (**23**)).

We also observed that, in the 2-methylphenyl-substituted system, the THF and pyridine ligands become mutually exchangeable at high concentrations. By contrast, the vinylidene complexes bearing ^*t*^Bu (**22**) or SiMe_3_ (**23**) substituents display remarkable stability and do not undergo ligand exchange, remaining intact even in THF solution. Additionally, to access the THF-ligated analogues of **22** and **23** while avoiding potential steric inhibition of ligand-exchange pathways by the bulky ^*t*^Bu and SiMe_3_ group, complex **2** was treated with RCCH (^*t*^Bu, SiMe_3_). Nonetheless, no signal corresponding to the formation of the target vinylidene complexes was detected. These observations regarding the easier access to vinylidene formation from 2-methylphenylacetylene are fully consistent with established vinylidene chemistry, in which the vinylidene moiety is best stabilized by stronger electron-withdrawing vinyl substituents in combination with strongly σ-donating ancillary ligands.^58,59^

Importantly, these stabilized vinylidene complexes can also undergo the [3 + 2] cycloaddition reaction upon further treatment with 1 equiv of terminal alkynes RCCH (R = C_6_H_5_ and *n*-C_3_H_7_) to furnish green [3 + 2] C_3_W_2_ products: **19**, derived from **21**; W_2_Cl_2_[μ-η:^2^η^2^-1-R-4-^*t*^BuH-C_4_H]­[μ-κ^2^-HC­(N-2,6-^*i*^Pr_2_C_6_H_3_)_2_]_2_ (R = C_6_H_5_ (**24**) and *n*-C_3_H_7_ (**25**)), derived from **22**; and W_2_Cl_2_[μ-η:^2^η^2^-1-*n*-C_3_H_7_-4-SiMe_3_H–C_4_H]­[μ-κ^2^-HC­(N-2,6-^*i*^Pr_2_C_6_H_3_)_2_]_2_ (**26**), derived from **23**. Their structures were established by single-crystal X-ray diffraction and NMR spectroscopy, revealing closely comparable metrical parameters across the series. Alternatively, in the presence of a coordinating solvent, these five-membered metallacycles can be accessed directly via in situ [2 + 2 + 1] cycloadditions by treating the quadruply bonded W–W species **2** or **3** sequentially with two different alkynes, although this one-pot procedure generally affords the desired [2 + 2 + 1] ditungsten cycloadducts in lower purity.

Notably, given that the W–W quadruple bond can serve as a reaction partner for [2 + 2 + 1] and [2 + 2 + 2] cycloadditions with alkynes, the catalytic potential of complexes **2** and **3** for alkyne trimerization was also evaluated by treating them with 10 equiv of alkyne. Nonetheless, no resulting benzene or fulvene derivatives were detected. Presumably, unlike the digonal paddlewheel conformation of M–M quintuply bonded amidinate complexes (M = Cr, Mo), which can serve as catalysts for alkyne cyclotrimerization,^21,60^ the two terminal chloride ligands on the W–W core sterically block the coordination sites required for the incoming third alkyne, thereby preventing the catalytic cycle from proceeding.

To determine whether [2 + 2 + 1] cycloadducts also display a distinctive redox behavior, we next examined the reduction of **24**. The reaction outcome proved to be highly selective. Treatment of **24** with 1 equiv of KC_8_ in *n*-hexane resulted in a one-electron reduction, affording W_2_Cl­[μ-κ^2^-1-C_6_H_5_-4-^*t*^BuH-C_4_H]­[μ-κ^2^-HC­(N-2,6-^*i*^Pr_2_C_6_H_3_)_2_]_2_ (**27**). In contrast, treatment of **24** with 2 equiv of KC_8_ or subsequent reduction of **27** in an ethereal solvent induced intramolecular C–H cleavage to furnish W_2_(μ-H)­[μ-κ^2^-1-C_6_H_5_-4-^*t*^BuH-C_4_H]­[μ-κ^3^-HC­(N-2,6-^*i*^Pr_2_C_6_H_3_)­(N-2-^*i*^Pr-6-(C_3_H_6_)­C_6_H_3_)]­[μ-κ^2^-HC­(N-2,6-^*i*^Pr_2_C_6_H_3_)_2_] (**28**). The structures of both **27** and **28** were confirmed by single-crystal X-ray diffraction. In **27** (Figure 6a), the W–W bond length contracts to 2.2387(4) Å from 2.3252(6) Å in **24**, consistent with occupation of a metal–metal bonding orbital and a 0.5 increase in W–W bond order. At the same time, the C_3_W_2_ dimetallacycle becomes essentially coplanar, and the C_3_ fragment adopts μ-η:^1^η^1^ alkyl coordination to the ditungsten center (W1–C3 = 2.136(3) Å; W2–C1 = 2.107(3) Å). The C2 atom no longer interacts with the distal tungsten center but instead forms a localized C2=C3 double bond (1.366(3) Å). Together with the exocyclic C=C unit (C1–C10 = 1.355(3) Å), these features establish **27** as a bimetallic fulvene analogue in which one endocyclic C=C bond is replaced by a W–W multiple bond.

**Figure 6:**
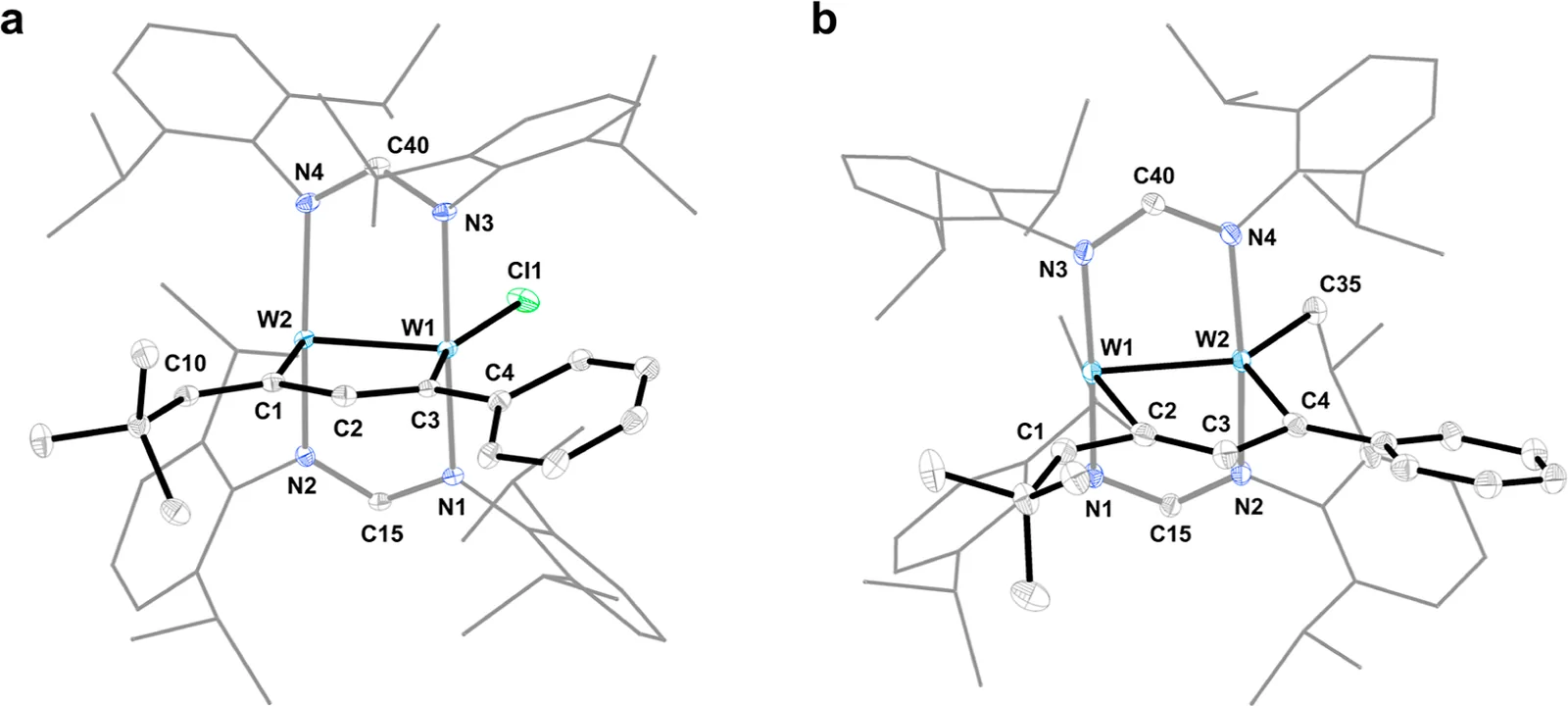
(a) The solid-state molecular structure of **27**. (b) The solid-state molecular structure of **28**. (Thermal ellipsoids at 30% probability. Hydrogen atoms are omitted for clarity).

Further one-electron reduction of **27** furnishes a chloride-free W_2_ unit; however, one methyl group of a diisopropyl substituent unexpectedly undergoes C–H activation to give W_2_(μ-H)­[μ-κ^2^-1-C_6_H_5_-4-^*t*^BuH-C_4_H]­[μ-κ^3^-HC­(N-2,6-^*i*^Pr_2_C_6_H_3_)­(N-2-^*i*^Pr-6-(C_3_H_6_)­C_6_H_3_)]­[μ-κ^2^-HC­(N-2,6-^*i*^Pr_2_C_6_H_3_)_2_] (**28**). This formulation is supported by a significantly downfield-shifted hydride resonance at 4.71 ppm in the ^1^H NMR spectrum, accompanied by two sets of tungsten satellites, as well as by a doublet of doublets at 3.08 ppm attributable to W–C*H*_2_– group. The two sets of tungsten satellites not only confirm the hydride’s proximity to the W_2_ unit but also point to pronounced magnetic anisotropy associated with the C_3_W_2_ ring system, especially in comparison with the dihydride complex **4**. Single-crystal X-ray diffraction corroborates the C–H activation event (Figure 6b), revealing a distinct W2–C35 bond of 2.151(4) Å. The W–W distance reverts to 2.3495(3) Å, consistent with a formal W≡W triple bond.^28^ Compound **28**, however, does not simply mirror the electronic structure of **24**: although the C_3_W_2_ ring remains essentially coplanar, the C_3_W_2_ pentagon is distinctly distorted with the exocyclic C=C positioned fragment drawn closer to tungsten center (W1···C1 = 2.452(4) Å).

### Computational Studies

To probe the effect of solvent coordination on vinylidene stability, the enthalpy of a model complex, lacking THF or pyridine (**20′**), was compared with those of **17**, **20** and **21** (Figure S126). In the absence of the auxiliary ligand, the vinylidene complex **20′** lies 2.89 kcal/mol higher in enthalpy than the [2 + 2] cycloadduct **17**, indicating that vinylidene formation is energetically disfavored. Upon coordination of a THF or pyridine ligand, complex **20** and **21** become 2.65 and 4.72 kcal·mol^–1^ lower in enthalpy than **17**. This reversal in relative stability demonstrates that auxiliary ligand provides substantial stabilization to the tungsten center. Such stabilization is particularly important because the vinylidene ligand is a stronger π-acceptor than the corresponding alkyne,^58,59^ thereby imposing a greater electronic demand on the metal center and requiring additional donor support from pyridine to achieve stability.

Although the [2 + 2] cycloadduct can persist in the absence of coordinating solvents and subsequently undergo further [4 + 2] cycloaddition, the calculated energy profile provides detailed insight into the transformation pathway (Figure 7). Starting from the model complex **17** ^**Ph**^, where the aryl groups are simplified to phenyl substituents, a second alkyne molecule initially coordinates to the axial site of the W–W bond to form intermediate **INT1a**. To facilitate the subsequent C–C bond-forming step, the coordinated alkyne molecule then rotates into an orientation perpendicular to the first alkyne, affording **INT2a**. This intermediate serves as the key precursor to the experimentally observed tail-to-tail coupling product. In **INT2a**, the relative orientation of the two coordinated alkynes minimizes steric repulsion while positioning the two less hindered H-substituted carbon atoms for selective coupling, ultimately leading to the tail-to-tail product **13** ^**Ph**^.

**Figure 7:**
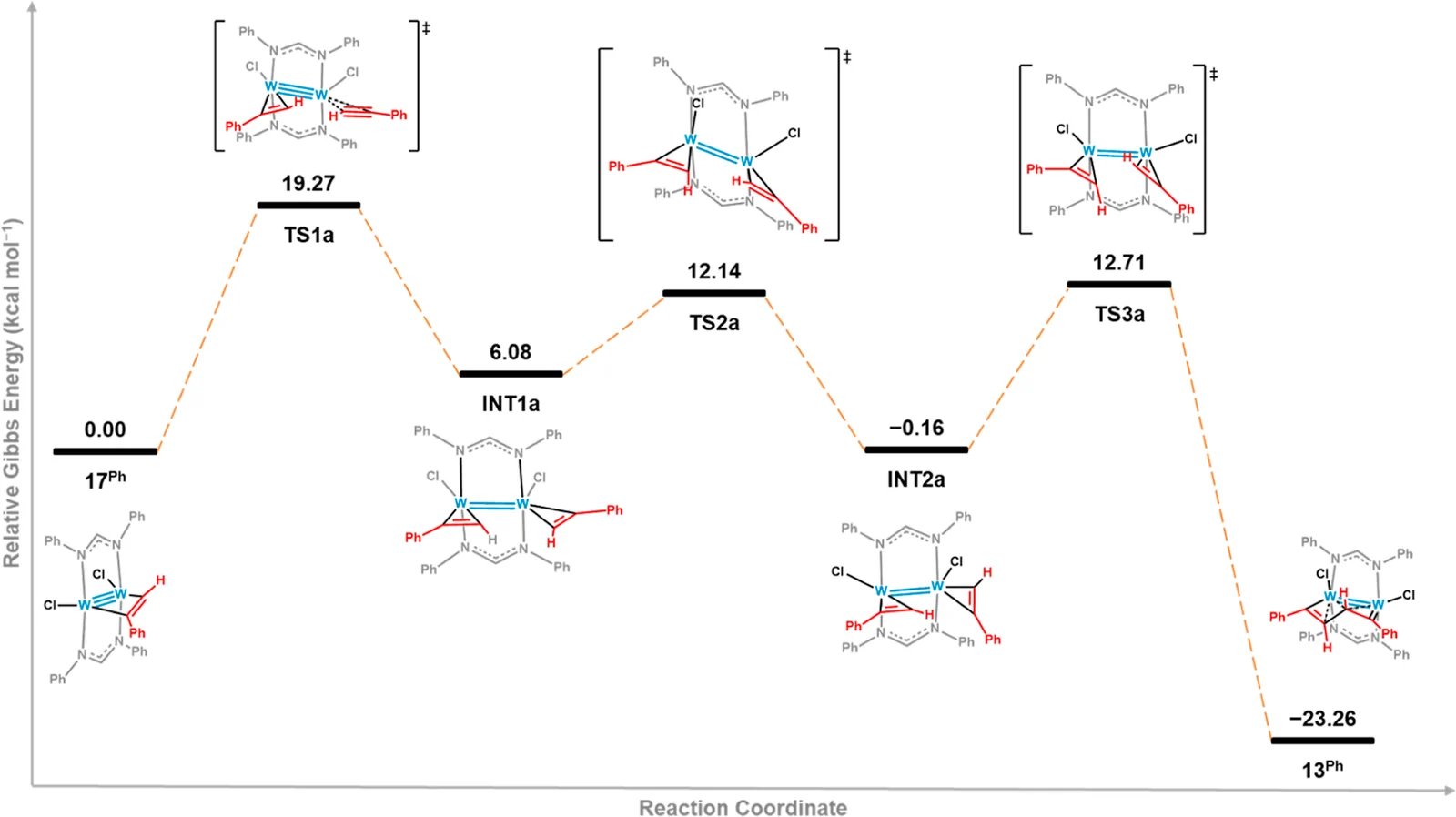
DFT-calculated energy profile for the [2 + 2 + 2] cycloaddition pathway from intermediate **17** to **13** (relative Gibbs free energies are given in kcal·mol^–1^).

For the [3 + 2] pathway (Figure S128), the stabilizing effect of pyridine suggests that the reaction most likely proceeds through an associative or interchange mechanism rather than a dissociative one. Since both chlorido ligands remain terminal throughout the process, the limited coordination space at the W_2_ core favors an interchange pathway. Starting from the model complex **22** ^**Ph**^, where all aryl substituents are simplified to phenyl groups, 1-pentyne coordinates to the W_2_ unit while displacing the pyridine ligand from the site opposite the vinylidene group, thereby generating intermediate **INT1b**. Intermediate **INT1b** then undergoes [3 + 2] cycloaddition with a remarkably low activation barrier of only 4.60 kcal/mol, ultimately affording the final product **25** ^**Ph**^.

To gain deeper insight into the electronic structures of these novel W_2_-containing species, DFT calculations were performed on **1**–**5**, **12**, **20**, **21**, **22**, **24**, **27**, and **28**, and the optimized geometries are in good agreement with their corresponding solid-state structures. For **1** (Figures S134 and S135), the α-spin manifold of the canonical molecular orbitals (MOs) contains four occupied W–W bonding orbitals, δ­(d_*xy*_ + d_*xy*_), π­(d_*xz*_ + d_*xz*_), π­(d_*yz*_ + d_*yz*_) and σ­(d_*z* ^2^_ + d_*z* ^2^_), located at the αHOMO, αHOMO – 1, αHOMO – 2 and αHOMO – 4 levels, respectively. In contrast, the β-spin manifold contains three occupied W–W bonding orbitals, δ­(d_*xy*_ + d_*xy*_), π­(d_*xz*_ + d_*xz*_), and π­(d_*yz*_ + d_*yz*_), corresponding to the βHOMO, βHOMO – 1, and βHOMO – 2, respectively. This electron-occupancy pattern is consistent with a W–W formal bond order (fBO) of 3.5 with a Mayer bond order (MBO) of 2.89.^61^

For **2** (Figure S136) and **3** (Figure S137), one additional electron resides in the W–W δ-bonding orbital relative to **1**, and the dominant W–W bonding contributions follow the same ordering δ­(d_*xy*_ + d_*xy*_) > π­(d_*xz*_ + d_*xz*_) > π­(d_*yz*_ + d_*yz*_) > σ­(d_*z* ^2^_ + d_*z* ^2^_). In **2**, these orbitals are located at the HOMO, HOMO – 2, HOMO – 3, and HOMO – 11, whereas in **3**, they appear at the HOMO, HOMO – 1, HOMO – 2, and HOMO – 3 levels. The corresponding W–W MBOs are 3.02 for **2** and 2.82 for **3**. Complex **4** can be viewed as a transient W–W unit preceding double C–H activation. Notably, its W–W formal triple bond is constructed by δ­(d_*xy*_ + d_*xy*_), π­(d_*yz*_ + d_*yz*_) and σ­(d_*z* ^2^_ + d_*z* ^2^_) orbitals, located at the HOMO, HOMO – 1 and HOMO – 2 levels, respectively (Figure S138). The resulting W–W MBO is 2.12.

The MOs of **5** (Figure 8a) exhibit a characteristic electronic pattern for a bimetallic aromatic system, closely paralleling that of its dimolybdenum analogue.^21^ The HOMO shows δ^2^-π^4^ conjugation delocalized over the C_4_W_2_ ring. In this orbital, the tungsten δ manifold mixes with the π system of the 1,4-butadienyl fragment, such that the two α carbons engage in π-bonding with the W centers. The HOMO – 1 corresponds to one-node π-conjugated orbital of butadienyl ligand, whereas the fully in-phase four-p_*y*_ π-bonding combination resides much lower in energy at the HOMO – 21. The limited W–W contribution to the HOMO – 1 and HOMO – 21 reflects a substantial energy mismatch between the W–W δ set and the butadienyl π orbitals. Meanwhile, the HOMO – 2, HOMO – 3 and HOMO – 4 are dominated by W–W π and σ bonding interactions, with only modest mixing into the butadienyl ligand. The W–W MBO is 2.79, essentially identical to that of **1**. Moreover, the NICS_*zz*_ line scan indicates a diatropic ring current for the C_4_W_2_ ring of **5** (Figure S153),^62–64^ and the normalized multicenter bond order (MCBO) for the C_4_W_2_ ring is 0.571, comparable to that of benzene (0.643).^65,66^ Together, these results support the assignment of **5** as a bimetallic aromatic species.

**Figure 8:**
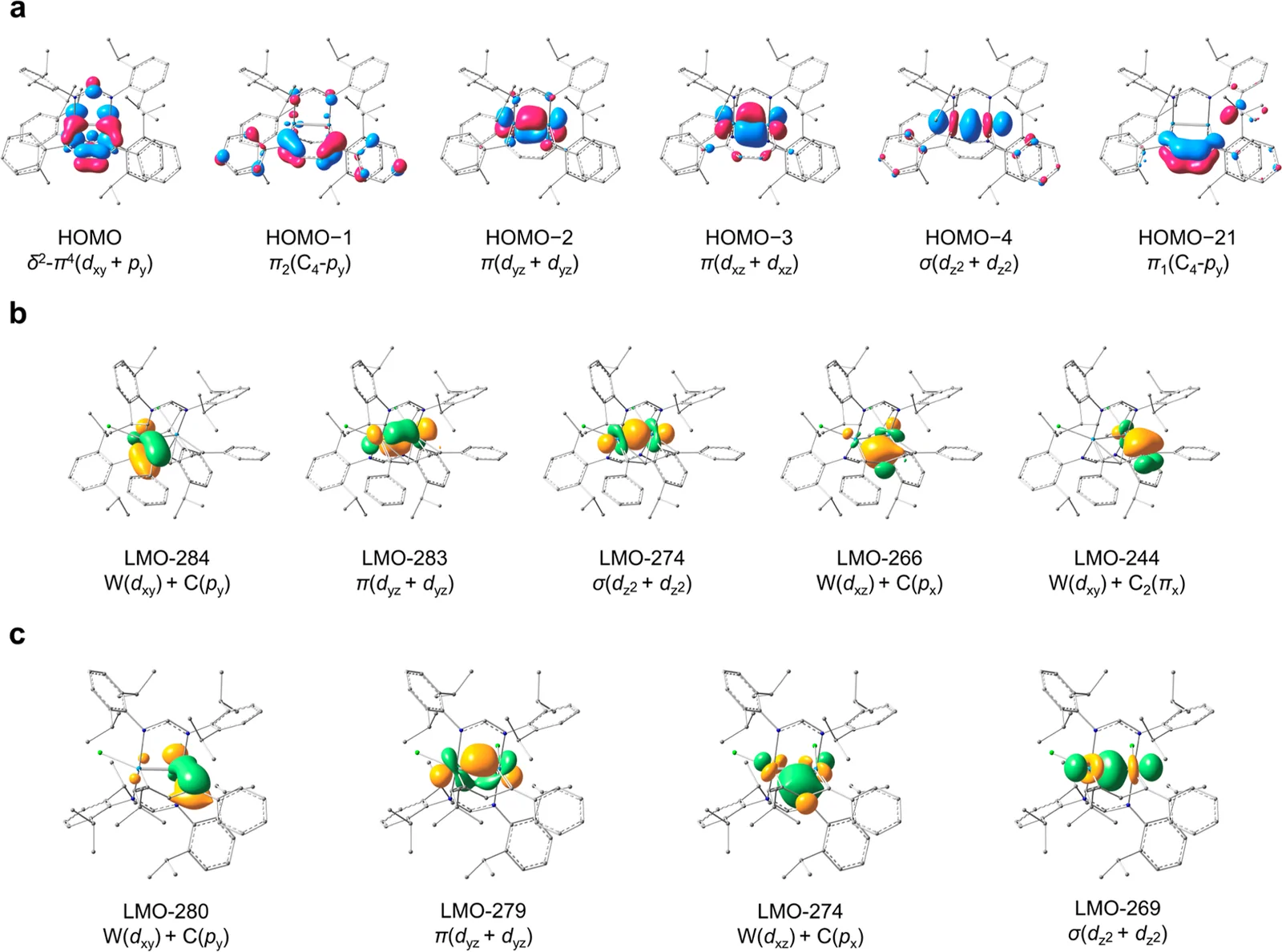
(a) Frontier MOs of **5** (isosurface = 0.05). (b) LMOs of **12** (isosurface = 0.06). (c) LMOs of **24** (isosurface = 0.06).

The MOs of **12** show extensive mixing within the C_4_W_2_ ring (Figure S140). Within the canonical MO set, only the W–W π- and σ-bonding combinations can be cleanly identified, appearing at the HOMO – 1 and HOMO – 2 levels, respectively. To further resolve the metal–ligand interactions within the ring, localized molecular orbitals (LMOs) were analyzed (Figure 8b).^67–69^ LMO-284 depicts an η^1^-alkylidene interaction between W1 and C1, whereas LMO-266 corresponds primarily to a bridging alkyl fragment centered at C2. In addition, LMO-244 depicts the C3–C4 unit as an η^2^-alkene coordinated to W2. Collectively, these results indicate that the C_4_ fragment binds the two metal centers through a distinctive η^1^-alkylidene-μ-η^1^-alkyl-η^2^-alkene motif. The W–W π and σ bonds are likewise evident in LMO-283 and LMO-274, respectively.

Distinctive bonding features are observed in the bent vinylidene complex **20** (Figure S141). The HOMO displays not only the conventional π­(d_*yz*_ + p_*y*_) interaction of the vinylidene ligand, but also additional π-conjugation with the W–W π­(d_*yz*_ + d_*yz*_) manifold; this delocalization is captured in LMO-281 (Figure S149). The HOMO – 1 likewise shows significant coupling between the W–W π­(d_*xz*_ + d_*xz*_) manifold and the C=CH_2_ fragment. Decomposition into LMOs further clarifies these interactions. LMO-276 depicts the C1 p_*y*_ orbital bridging into the W–W π­(d_*xz*_ + d_*xz*_) manifold, with a pronounced π interaction between C1 and W2. Meanwhile, LMO-280 and LMO-247 show the p_*z*_ orbital of C1 and C2 interacting with the tungsten centers, respectively.

A bonding pattern closely related to that in **12** is observed in **24** (Figure S144). The HOMO and HOMO – 1 correspond to η^1^-alkylidene interactions coupled with the W–W π­(d_*yz*_ + d_*yz*_) manifold, whereas the HOMO – 2 represents a μ-η^1^-alkyl interaction involving the W–W π­(d_*xz*_ + d_*xz*_) manifold. The exocyclic C=C bond shows no significant additional interaction with the ditungsten core. These interactions are likewise visualized in LMO-280 and LMO-274 (Figure 8c). The W–W MBO is 2.10, consistent with a formal W–W triple bond.

Compound **27** displays a W–W manifold closely resembling that of **1** (Figures S145 and S146): both possess a W–W fBO of 3.5, and **27** has a calculated MBO of 2.79, identical to that of **1**. Within the planar C_3_W_2_ ring of **27**, δ-π conjugation is evident. The αHOMO is a W–W δ orbital containing a non-negligible contribution from the p orbital of the vertex-carbon atom, whereas the αHOMO – 1 and βHOMO show π^4^ conjugation spanning both the exocyclic and endocyclic C=C bonds. Compound **28** exhibits the same δ-π conjugation pattern (Figure S147). Although it undergoes C–H activation, its HOMO remains a δ orbital, while only one π and one σ frontier orbital are retained at the HOMO – 2 and HOMO – 4 levels, respectively.

## Conclusions

In summary, we have demonstrated that a W–W quadruple bond serves as a versatile partner for the cycloaddition of terminal alkynes, exhibiting reactivity that extends beyond classical unsaturated π systems to specifically emulate that of alkenes. This unusual reactivity enables ligand-controlled divergence between formal [2 + 2 + 2] and [2 + 2 + 1] manifolds, together with highly selective tail-to-tail coupling in the [2 + 2 + 2] pathway. The resulting C_4_W_2_ rings in the initial cycloadducts **12**–**15** are nonplanar but undergo redox-induced planarization upon reduction to **5**–**11**, underscoring both the structural flexibility of these frameworks and the emergence of aromaticity upon population of the W–W δ manifold. Importantly, isolation of the direct [2 + 2] cycloadduct **17** from reaction with 2-methylphenylacetylene provides key mechanistic evidence for this stepwise assembly process, while subsequent reaction with 1-pentyne enables formation of the unsymmetrical [2 + 2 + 2] product **16**. Furthermore, ligand-controlled chemodivergence was revealed by the observation of the isomeric C_3_W_2_ product **19** in the presence of THF, indicating that treatment of the [2 + 2] precursor **17** with THF promotes its conversion into the THF-ligated bent vinylidene complex **20**. Introduction of pyridine significantly broadens the substrate scope of terminal alkynes, giving an expanded series of stabilized vinylidene complexes **21**–**23**. Subsequent reaction of these vinylidene complexes with a second alkyne furnishes formal [2 + 2 + 1]­cycloadducts featuring C_3_W_2_ rings with an exocyclic C=C fragment (**24**–**26**). Although these initial products adopt distorted conformations, one- or two-electron reduction affords the planarized species **27** and **28**. This transformation highlights the central role of δ bonding in mediating conjugation between the ditungsten core and the organic π system. Overall, the exclusive regioselectivity and tunable divergence between [2 + 2 + 2] benzene-like and [2 + 2 + 1] fulvene-like pathways, governed by ligand coordination and redox state, provide a useful framework for controlling complex cycloaddition chemistry at metal–metal multiple bonds.

## Supplementary Material



## Notes

Additional experimental/computational details: materials, methods, spectroscopic data, crystallographic data, and optimized Cartesian coordinates (PDF)
