# Supplementary material for: Low-Coordinate W–W Quadruple Bond as an Alkene Surrogate: Divergent [2 + 2 + 2] and [2 + 2 + 1] Cycloadditions with Alkynes
Source: JACS Au. 2026 Sep 24;6(10):5935–48. doi: 10.1021/jacsau.6c01251 (PMC13714859; doi:10.1021/jacsau.6c01251)
Supplement: Supplementary file 1 [file au6c01251_si_002.pdf]

Low-Coordinate W−W Quadruple Bond as an Alkene Surrogate: Divergent [2+2+2] and [2+2+1] Cycloadditions with Alkynes

Wen-Chi Chen,‡ Cian-Wei Yang,‡ Bo-An Liao,‡ Chiao-Yun Chen, Hong-Zhang Chen, Wen-Chuan Hsu, Yi-Chou Tsai*

Department of Chemistry, National Tsing Hua University, Hsinchu 30013, Taiwan.

Email: yictsai@mx.nthu.edu.tw

‡These authors contributed equally.

Table of contents

1 General consideration......1

2 Synthesis 2

2.1 $[Li(THF)_4][\{WCl_2(\mu\text{-}\kappa^2\text{-}HC(N\text{-}2,6\text{-}^iPr_2C_6H_3)_2)\}_2]$ (**1**) 2

2.2 $W_2(\mu\text{-}Cl)(\mu\text{-}\kappa^2\text{-}Cl_2Li(THF))[\mu\text{-}\kappa^2\text{-}HC(N\text{-}2,6\text{-}^iPr_2C_6H_3)_2]_2$ (**2**)......2

2.3 $W_2Cl(\mu\text{-}Cl)(NC_5H_5)_2[\mu\text{-}\kappa^2\text{-}HC(N\text{-}2,6\text{-}^iPr_2C_6H_3)_2]_2$ (**3**) 3

2.4 $W_2(THF)(\mu\text{-}H)_2[\mu\text{-}\kappa^3\text{-}HC(N\text{-}2\text{-}CH(CH_2)(CH_3)\text{-}6\text{-}^iPrC_6H_3)(N\text{-}2,6\text{-}^iPr_2C_6H_3)]_2$ (**4**) 4

2.5 $W_2(\mu\text{-}\kappa^2\text{-}1,4\text{-}Ph_2C_4H_2)[\mu\text{-}\kappa^2\text{-}HC(N\text{-}2,6\text{-}^iPr_2C_6H_3)_2]_2$ (**5**) 5

2.6 $W_2(\mu\text{-}\kappa^2\text{-}1,4\text{-}(2\text{-}MePh)_2C_4H_2)[\mu\text{-}\kappa^2\text{-}HC(N\text{-}2,6\text{-}^iPr_2C_6H_3)_2]_2$ (**6**) 6

2.7 $W_2(\mu\text{-}\kappa^2\text{-}1,4\text{-}(3\text{-}MePh)_2C_4H_2)[\mu\text{-}\kappa^2\text{-}HC(N\text{-}2,6\text{-}^iPr_2C_6H_3)_2]_2$ (**7**) 7

2.8 $W_2(\mu\text{-}\kappa^2\text{-}1,4\text{-}(4\text{-}MePh)_2C_4H_2)[\mu\text{-}\kappa^2\text{-}HC(N\text{-}2,6\text{-}^iPr_2C_6H_3)_2]_2$ (**8**) 8

2.9 $W_2(\mu\text{-}\kappa^2\text{-}1,4\text{-}(4\text{-}OMePh)_2C_4H_2)[\mu\text{-}\kappa^2\text{-}HC(N\text{-}2,6\text{-}^iPr_2C_6H_3)_2]_2$ (**9**) 9

2.10 $W_2(\mu\text{-}\kappa^2\text{-}1,4\text{-}(4\text{-}FPh)_2C_4H_2)[\mu\text{-}\kappa^2\text{-}HC(N\text{-}2,6\text{-}^iPr_2C_6H_3)_2]_2$ (**10**) 10

2.11 $W_2(\mu\text{-}\kappa^2\text{-}1,4\text{-}(n\text{-}C_3H_7)_2C_4H_2)[\mu\text{-}\kappa^2\text{-}HC(N\text{-}2,6\text{-}^iPr_2C_6H_3)_2]_2$ (**11**) 12

2.12 $W_2Cl_2[\mu\text{-}\eta^2\text{:}\eta^2\text{-}1,4\text{-}Ph_2C_4H_2][\mu\text{-}\kappa^2\text{-}HC(N\text{-}2,6\text{-}^iPr_2C_6H_3)_2]_2$ (**12**)......12

2.13 $W_2Cl_2[\mu\text{-}\eta^2\text{:}\eta^2\text{-}1,4\text{-}(2\text{-}MePh)_2C_4H_2][\mu\text{-}\kappa^2\text{-}HC(N\text{-}2,6\text{-}^iPr_2C_6H_3)_2]_2$ (**13**) 13

2.14 $W_2Cl_2[\mu\text{-}\eta^2\text{:}\eta^2\text{-}1,4\text{-}(4\text{-}OMePh)_2C_4H_2][\mu\text{-}\kappa^2\text{-}HC(N\text{-}2,6\text{-}^iPr_2C_6H_3)_2]_2$ (**14**) 14

2.15 $W_2Cl_2[\mu\text{-}\eta^2\text{:}\eta^2\text{-}1,4\text{-}(n\text{-}C_3H_7)_2C_4H_2][\mu\text{-}\kappa^2\text{-}HC(N\text{-}2,6\text{-}^iPr_2C_6H_3)_2]_2$ (**15**)......15

2.16 $W_2Cl_2[\mu\text{-}\eta^2\text{:}\eta^2\text{-}1\text{-}2\text{-}MeC_6H_4\text{-}4\text{-}n\text{-}C_3H_7C_4H_2][\mu\text{-}\kappa^2\text{-}HC(N\text{-}2,6\text{-}^iPr_2C_6H_3)_2]_2$ (**16**) 17

2.17 $W_2Cl_2[\mu\text{-}\eta^2\text{-}2\text{-}MeC_6H_4CCH][\mu\text{-}\kappa^2\text{-}HC(N\text{-}2,6\text{-}^iPr_2C_6H_3)_2]_2$ (**17**) 18

2.18 W_2[μ-κ^2-1-2-MeC_6H_4-4-*n*-$C_3H_7C_4H_2$][μ-κ^2-HC(N-2,6-^{i}Pr$_2C_6H_3$)$_2$]$_2$ (**18**)....19

2.19 W_2Cl_2[μ-η^2:η^2-1-*n*-C_3H_7-4-2-MeC_6H_4(H)-C_4H][μ-κ^2-HC(N-2,6-^{i}Pr$_2C_6H_3$)$_2$]$_2$ (**19**)....20

2.20 W_2Cl_2(THF)[μ-η^1:η^2-CC(H)-2-MeC_6H_4][μ-κ^2-HC(N-2,6-^{i}Pr$_2C_6H_3$)$_2$]$_2$ (**20**)....21

2.21 W_2Cl_2(C_5H_5N)[μ-η^1:η^2-CC(H)-2-MeC_6H_4][μ-κ^2-HC(N-2,6-^{i}Pr$_2C_6H_3$)$_2$]$_2$ (**21**)....22

2.22 W_2Cl_2(C_5H_5N)[μ-η^1:η^2-CC(H)-*t*-Bu][μ-κ^2-HC(N-2,6-^{i}Pr$_2C_6H_3$)$_2$]$_2$ (**22**)....23

2.23 W_2Cl_2(C_5H_5N)[μ-η^1:η^2-CC(H)-SiMe$_3$][μ-κ^2-HC(N-2,6-^{i}Pr$_2C_6H_3$)$_2$]$_2$ (**23**)....24

2.24 W_2Cl_2[μ-η^2:η^2-1-C_6H_5-4-^{t}BuH-C_4H][μ-κ^2-HC(N-2,6-^{i}Pr$_2C_6H_3$)$_2$]$_2$ (**24**)....24

2.25 W_2Cl_2[μ-η^2:η^2-1-*n*-C_3H_7-4-^{t}BuH-C_4H][μ-κ^2-HC(N-2,6-^{i}Pr$_2C_6H_3$)$_2$]$_2$ (**25**)....26

2.26 W_2Cl_2[μ-η^2:η^2-1-*n*-C_3H_7-4-SiMe$_3$H-C_4H][μ-κ^2-HC(N-2,6-^{i}Pr$_2C_6H_3$)$_2$]$_2$ (**26**)....27

2.27 W_2Cl[μ-κ^2-1-C_6H_5-4-^{t}BuH-C_4H][μ-κ^2-HC(N-2,6-^{i}Pr$_2C_6H_3$)$_2$]$_2$ (**27**)....28

2.28 W_2(μ-H)[μ-κ^2-1-C_6H_5-4-^{t}BuH-C_4H][μ-κ^3-HC(N-2,6-^{i}Pr$_2C_6H_3$)(N-2-^{i}Pr-6-(C_3H_6)C_6H_3)][μ-κ^2-HC(N-2,6-^{i}Pr$_2C_6H_3$)$_2$] (**28**)....29

3 X-ray crystallography....31

4 NMR Spectroscopy....71

4.1 [Li(THF)$_4$][{WCl$_2$(μ-κ^2-HC(N-2,6-^{i}Pr$_2C_6H_3$)$_2$)}$_2$] (**1**)....71

4.2 W_2(μ-Cl)(μ-κ^2-Cl$_2$Li(THF))[μ-κ^2-HC(N-2,6-^{i}Pr$_2C_6H_3$)$_2$]$_2$ (**2**)....72

4.3 W_2Cl(μ-Cl)(NC_5H_5)$_2$[μ-κ^2-HC(N-2,6-^{i}Pr$_2C_6H_3$)$_2$]$_2$ (**3**)....74

4.4 W_2(THF)(μ-H)$_2$[μ-κ^3-HC(N-2-CH(CH$_2$)(CH$_3$)-6-^{i}PrC_6H_3)(N-2,6-^{i}Pr$_2C_6H_3$)]$_2$ (**4**)....75

4.5 W_2(μ-κ^2-1,4-Ph$_2C_4H_2$)[μ-κ^2-HC(N-2,6-^{i}Pr$_2C_6H_3$)$_2$]$_2$ (**5**)....77

4.6 W_2(μ-κ^2-1,4-(2-MePh)$_2C_4H_2$)[μ-κ^2-HC(N-2,6-^{i}Pr$_2C_6H_3$)$_2$]$_2$ (**6**)....79

4.7 W_2(μ-κ^2-1,4-(3-MePh)$_2C_4H_2$)[μ-κ^2-HC(N-2,6-^{i}Pr$_2C_6H_3$)$_2$]$_2$ (**7**)....80

4.8 W_2(μ-κ^2-1,4-(4-MePh)$_2C_4H_2$)[μ-κ^2-HC(N-2,6-^{i}Pr$_2C_6H_3$)$_2$]$_2$ (**8**)....81

4.9 W_2(μ-κ^2-1,4-(4-OMePh)$_2C_4H_2$)[μ-κ^2-HC(N-2,6-^{i}Pr$_2C_6H_3$)$_2$]$_2$ (**9**)....82

4.10 W_2(μ-κ^2-1,4-(4-FPh)$_2C_4H_2$)[μ-κ^2-HC(N-2,6-^{i}Pr$_2C_6H_3$)$_2$]$_2$ (**10**)....83

4.11 W_2(μ-κ^2-1,4-(*n*-C_3H_7)$_2C_4H_2$)[μ-κ^2-HC(N-2,6-^{i}Pr$_2C_6H_3$)$_2$]$_2$ (**11**)....84

4.12 W_2Cl_2[μ-η^2:η^2-1,4-Ph$_2C_4H_2$][μ-κ^2-HC(N-2,6-^{i}Pr$_2C_6H_3$)$_2$]$_2$ (**12**)....86

4.13 W_2Cl_2[μ-η^2:η^2-1,4-(2-MePh)$_2C_4H_2$][μ-κ^2-HC(N-2,6-^{i}Pr$_2C_6H_3$)$_2$]$_2$ (**13**)....88

4.14 W_2Cl_2[μ-η^2:η^2-1,4-(4-OMePh)$_2C_4H_2$][μ-κ^2-HC(N-2,6-^{i}Pr$_2C_6H_3$)$_2$]$_2$ (**14**)....90

4.15 W_2Cl_2[μ-η^2:η^2-1,4-(*n*-C_3H_7)$_2C_4H_2$][μ-κ^2-HC(N-2,6-^{i}Pr$_2C_6H_3$)$_2$]$_2$ (**15**)....92

4.16 $W_2Cl_2[\mu\text{-}\eta^2{:}\eta^2\text{-1-2-MeC}_6H_4\text{-4-n-}C_3H_7C_4H_2][\mu\text{-}\kappa^2\text{-HC(N-2,6-}^i\text{Pr}_2C_6H_3)_2]_2$ (**16**) 94

4.17 $W_2Cl_2[\mu\text{-}\eta^2\text{-2-MeC}_6H_4\text{CCH}][\mu\text{-}\kappa^2\text{-HC(N-2,6-}^i\text{Pr}_2C_6H_3)_2]_2$ (**17**) 95

4.18 $W_2[\mu\text{-}\kappa^2\text{-1-2-MeC}_6H_4\text{-4-}n\text{-}C_3H_7C_4H_2][\mu\text{-}\kappa^2\text{-HC(N-2,6-}^i\text{Pr}_2C_6H_3)_2]_2$ (**18**) 96

4.19 $W_2Cl_2[\mu\text{-}\eta^2{:}\eta^2\text{-1-}n\text{-}C_3H_7\text{-4-2-MeC}_6H_4\text{(H)-}C_4H][\mu\text{-}\kappa^2\text{-HC(N-2,6-}^i\text{Pr}_2C_6H_3)_2]_2$ (**19**) 98

4.20 $W_2Cl_2\text{(THF)}[\mu\text{-}\eta^1{:}\eta^2\text{-CC(H)-2-MeC}_6H_4][\mu\text{-}\kappa^2\text{-HC(N-2,6-}^i\text{Pr}_2C_6H_3)_2]_2$ (**20**) 99

4.21 $W_2Cl_2(C_5H_5N)[\mu\text{-}\eta^1{:}\eta^2\text{-CC(H)-2-MeC}_6H_4][\mu\text{-}\kappa^2\text{-HC(N-2,6-}^i\text{Pr}_2C_6H_3)_2]_2$ (**21**) 100

4.22 $W_2Cl_2(C_5H_5N)[\mu\text{-}\eta^1{:}\eta^2\text{-CC(H)-}t\text{-Bu}][\mu\text{-}\kappa^2\text{-HC(N-2,6-}^i\text{Pr}_2C_6H_3)_2]_2$ (**22**) 101

4.23 $W_2Cl_2(C_5H_5N)[\mu\text{-}\eta^1{:}\eta^2\text{-CC(H)-SiMe}_3][\mu\text{-}\kappa^2\text{-HC(N-2,6-}^i\text{Pr}_2C_6H_3)_2]_2$ (**23**) 102

4.24 $W_2Cl_2[\mu\text{-}\eta^2{:}\eta^2\text{-1-}C_6H_5\text{-4-}^t\text{BuH-}C_4H][\mu\text{-}\kappa^2\text{-HC(N-2,6-}^i\text{Pr}_2C_6H_3)_2]_2$ (**24**) 103

4.25 $W_2Cl_2[\mu\text{-}\eta^2{:}\eta^2\text{-1-}n\text{-}C_3H_7\text{-4-}^t\text{BuH-}C_4H][\mu\text{-}\kappa^2\text{-HC(N-2,6-}^i\text{Pr}_2C_6H_3)_2]_2$ (**25**) 105

4.26 $W_2Cl_2[\mu\text{-}\eta^2{:}\eta^2\text{-1-}n\text{-}C_3H_7\text{-4-SiMe}_3\text{H-}C_4H][\mu\text{-}\kappa^2\text{-HC(N-2,6-}^i\text{Pr}_2C_6H_3)_2]_2$ (**26**) 106

4.27 $W_2Cl[\mu\text{-}\kappa^2\text{-1-}C_6H_5\text{-4-}^t\text{BuH-}C_4H][\mu\text{-}\kappa^2\text{-HC(N-2,6-}^i\text{Pr}_2C_6H_3)_2]_2$ (**27**) 107

4.28 $W_2(\mu\text{-H})[\mu\text{-}\kappa^2\text{-1-}C_6H_5\text{-4-}^t\text{BuH-}C_4H][\mu\text{-}\kappa^3\text{-HC(N-2,6-}^i\text{Pr}_2C_6H_3)(\text{N-2-}^i\text{Pr-6-}(C_3H_6)C_6H_3)][\mu\text{-}\kappa^2\text{-}$ $\text{HC(N-2,6-}^i\text{Pr}_2C_6H_3)_2]$ (**28**) 108

5 Evans method 110

6 Elemental analysis........................112

7 Details of computational studies 140

7.1 Computational methods 140

7.2 Comparison of the experimental and calculated structural metrics 141

7.3 Thermodynamic parameters and energy profiles 153

7.4 Canonical molecular orbital analysis 158

7.5 Localized molecular orbital analysis........................172

7.6 Nucleus-independent chemical shift (NICS) analysis........................177

7.7 Cartesian coordinates for the optimized geometries. 178

8 References 253

1 General consideration

All manipulations were carried out using standard Schlenk and glove box techniques under an atmosphere of high-purity nitrogen. The 4 Å sieves and *Celite* were dried in vacuo for one week at a temperature just above 200 °C. Diethyl ether (Et_2O) and tetrahydrofuran (THF) were distilled under nitrogen from purple sodium benzophenone ketyl. *n*-Pentane, *n*-hexane and toluene were passed through columns of solvent purification systems (Vigor VAPA-5) to remove oxygen and moisture. Hexamethyldisiloxane (TMS_2O) was refluxed over calcium hydride under nitrogen and distilled after 5 days. All distilled solvents were transferred under vacuum into vacuum-tight glass vessels before being transferred into a glove box and stored over activated molecular sieves. Benzene-d_6 (C_6D_6), toluene-d_8 (C_7D_8), THF-d_8 (C_4D_8O) were purchased from Aldrich Chemical and stored directly in gloveboxes. Phenylacetylene, 2-methylphenylacetylene, 3-methylphenylacetylene, 4-methylphenylacetylene, 4-fluorophenylacetylene, 4-methoxyphenylacetylene, trimethylsilylacetylene, *tert*-butylacetylene and 1-pentyne, fluorobenzene were degassed by freeze-pump-thaw and stored over molecular sieves. ^{1}H, $^{13}C\{^{1}H\}$ and $^{7}Li\{^{1}H\}$ solution NMR spectra were recorded using Varian Unity INOVA 500 MHz and Bruker AVANCE 500 MHz spectrometers. ^{1}H and $^{13}C\{^{1}H\}$ chemical shifts are reported referenced to the residual solvent resonances respectively.[1] Elemental analysis was carried out with a Heraeus CHN-O rapid elemental analyzer. $W_2Cl_6(DME)_2$,[2] KC_8[3] and lithiated amidinate Li[HC(N-2,6-$^{i}Pr_2C_6H_3)_2$] ($LiAm^{Dipp}$)[4–5] were synthesized following the documented methods.

2 Synthesis

2.1 [Li(THF)$_4$][{WCl$_2$(μ-κ^2-HC(N-2,6-^{i}Pr$_2$C$_6$H$_3$)$_2$)}$_2$] (1)

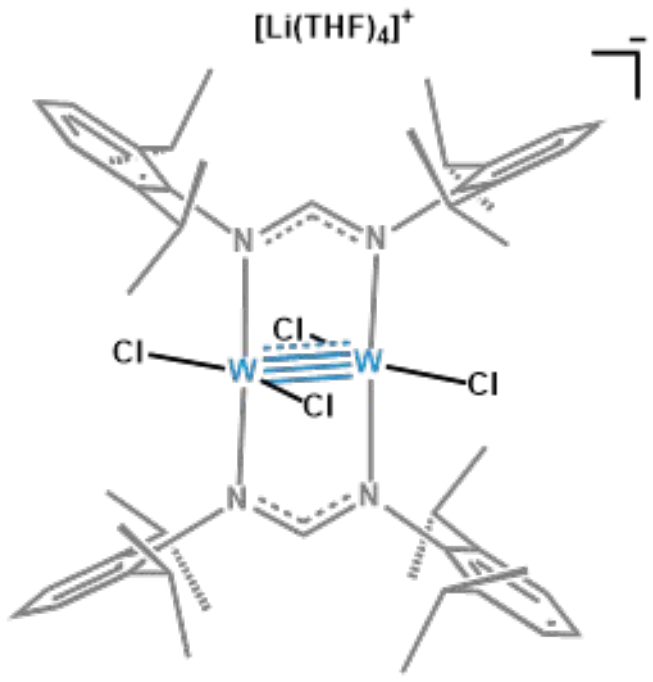

1

A 250 mL round-bottom flask was charged with $W_2Cl_6(DME)_2$ (6.0151 g, 7.9082 mmol) and THF (40 mL). The suspension was stirred for 5 min, then cooled in a liquid-nitrogen bath. KC_8 (1.0956 g, 8.1047 mmol) was added portion wise. The mixture was allowed to warm to room temperature and stirred for 3 h, during which it gradually turned dark green. The reaction was recooled (liquid-nitrogen bath) and $(LiAm^{Dipp})_2$ (39.6 mL of a 0.40 M solution in toluene) was added dropwise. The mixture was then allowed to warm to room temperature and stirred for 30 h, becoming red. The red mixture was passed through a pad of Celite, washing the residue with THF until the filtrate was colorless. The combined filtrate was concentrated under reduced pressure to a solid, which was collected on a frit and washed thoroughly with Et_2O until the washings were colorless. The solid was then redissolved in THF (40 mL) and recrystallized (1 day). The crystalline solid was collected on a frit and washed with Et_2O until the washings were colorless, then dried under vacuum to afford cherry-pink **1** (7.5007 g, 4.8961 mmol, 61.9%). Single crystals suitable for X-ray diffraction were obtained by recrystallization from THF at −35 °C.

^{1}H NMR (500 MHz, C_6D_6, 298 K): δ 8.17 (br. s), 4.40 (br. s), 3.59 (s, Li(THF)-CH_2(2,5)), 2.54(br, s), 1.41 (s, Li(THF)-CH_2(3.4)), 0.53 (br. s). **Evans method (500 MHz, 298 K):** $\mu_{\text{eff}} = 2.17\ \mu_{\text{B}}$. **Anal. Calcd. for $C_{66}H_{102}Cl_4LiN_4O_4W_2$:** N, 3.66. C, 51.74; H, 6.71; **Found:** N, 4.09. C, 52.43; H, 6.54.

Note: LiAm^{Dipp} is potential pyrophoric. Extreme caution must be exercised during the handling of both LiAm^{Dipp} and liquid nitrogen.

2.2 W$_2$(μ-Cl)(μ-κ^2-Cl$_2$Li(THF))[μ-κ^2-HC(N-2,6-^{i}Pr$_2$C$_6$H$_3$)$_2$]$_2$ (2)

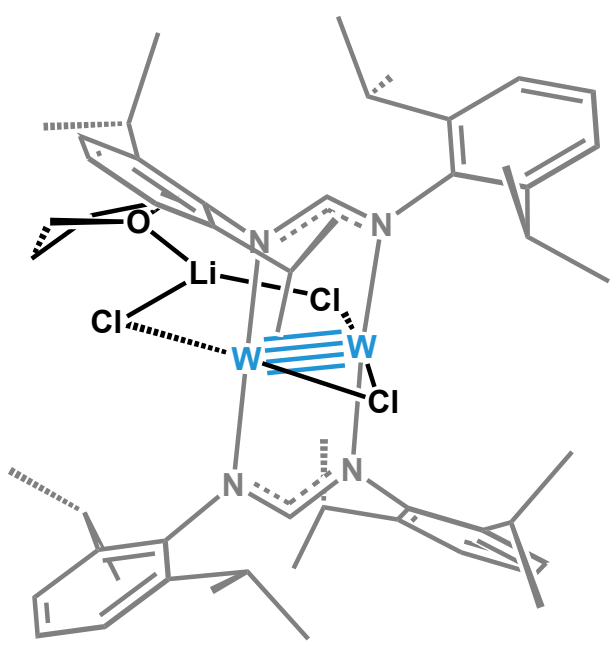

2

A 20-mL vial was charged with **1** (0.0545 g, 0.0356 mmol). Et_2O (6 mL) and KC_8 (0.0104 g, 0.0769 mmol) were added sequentially. The resulting solution was stirred for 30 h at room temperature, during which it gradually turned red to orange. The mixture was collected on a frit and washed thoroughly with Et_2O until the washings were colorless. The combined filtrate was concentrated under reduced pressure to a solid to

afford orange-yellow **2** (0.0387 g, 0.0302 mmol, 85.0%). Single crystals suitable for X-ray diffraction were obtained by recrystallization from *n*-hexane at −35 °C.

^{1}H NMR (500 MHz, C_6D_6, 298 K): δ 8.72 (s, 2H, NC*H*N), 7.19–7.17 (m, 4H, Dipp-Ar-*meta*), 7.12 (t, $^{3}J_{H–H}$ = 7.6 Hz, 4H, Dipp-Ar-*para*), 7.03 (d, $^{3}J_{H–H}$ = 7.8 Hz; 4H, Dipp-Ar-*meta*), 4.29 (sep, $^{3}J_{H–H}$ = 6.8 Hz, 4H, Dipp-C*H*(CH3)2), 3.87 (sep, $^{3}J_{H–H}$ = 6.8 Hz, 4H, Dipp-C*H*(CH_3)$_2$), 1.42 (d, $^{3}J_{H–H}$ = 6.7 Hz, 12H, Dipp-CH(C*H*$_3$)$_2$), 1.38 (d, $^{3}J_{H–H}$ = 6.7 Hz, 12H, Dipp-CH(C*H*$_3$)$_2$), 1.35 (d, $^{3}J_{H–H}$ = 6.7 Hz, 12H, Dipp-CH(C*H*$_3$)$_2$), 0.94 (d, $^{3}J_{H–H}$ = 6.7 Hz, 12H, Dipp-CH(C*H*$_3$)$_2$). **^{7}Li{^{1}H} NMR (194 MHz, C_6D_6, 298 K):** δ 1.56. **^{13}C NMR (126 MHz, C_6D_6, 298 K):** 166.8 (s, 2C, N*C*HN), 146.0 (s, 4C, Dipp-Ar-*C*), 145.0 (s, 4C, Dipp-Ar-*C*), 144.5 (s, 4C, Dipp-Ar-*C*), 126.7 (s, 4C, Dipp-Ar-*C*H), 124.6 (s, 4C, Dipp-Ar-*C*H), 124.1 (s, 4C, Dipp-Ar-*C*H), 29.1 (s, 4C, Dipp-*C*H(CH_3)$_2$), 28.9 (s, 4C, Dipp-*C*H(CH_3)$_2$), 28.7 (s, 4C, Dipp-CH(*C*H$_3$)$_2$), 26.6 (s, 4C, Dipp- CH(*C*H$_3$)$_2$), 25.3 (s, 4C, Dipp- CH(*C*H$_3$)$_2$), 25.2 (s, 4C, Dipp- CH(*C*H$_3$)$_2$). **Anal. Calcd. for $C_{54}H_{78}Cl_3LiN_4OW_2$:** N, 4.38; C, 50.66; H, 6.14; **Found:** N, 4.34; C, 51.00; H, 6.41.

Note: KC_8 is pyrophoric. Extreme caution must be exercised during its handling.

2.3 W_2Cl(μ-Cl)(NC_5H_5)$_2$[μ-κ^2-HC(N-2,6-$^{i}Pr_2C_6H_3$)$_2$]$_2$ (3)

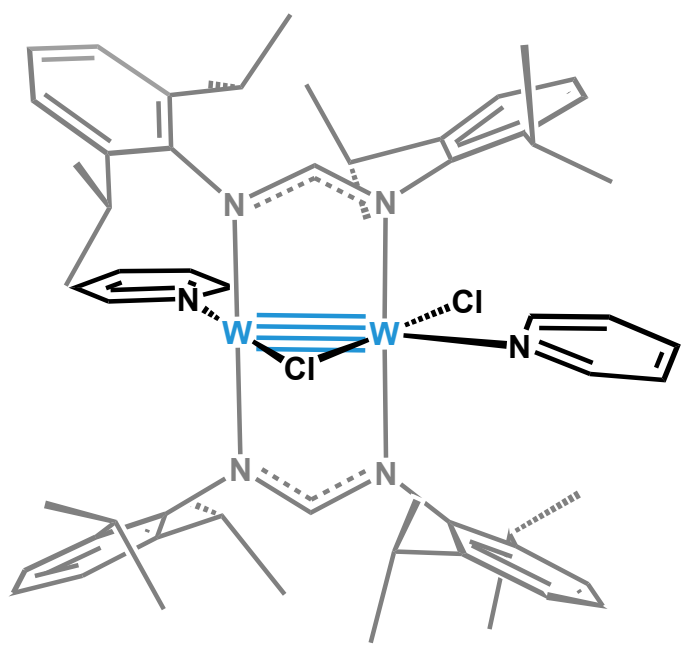

3

A 20 mL vial was charged with **2** (0.0540 g, 0.0352 mmol). Et_2O (6 mL) and pyridine (0.10 M, 0.70 mL, 0.0692 mmol) were added sequentially. The mixture was stirred at room temperature for 1.5 h, during which the rose-red suspension turned mint-green. Volatiles were removed under reduced pressure. *n*-Pentane (2 mL) was then added to the mixture and stirred 5 min at room temperature. The mint-green suspension was transferred onto a pad of *Celite*, the solid on the pad was washed with *n*-pentane until the filtrate was pale green. The residue was extracted with THF, and the insoluble materials were removed by filtration. The malachite-green filtrate was collected and concentrated under vacuum to give malachite-green **3** (0.0324 g, 0.0245 mmol, 70.7%). Single crystals suitable for X-ray diffraction were obtained by recrystallization from Et_2O at −35 °C.

^{1}H NMR (500 MHz, C_6D_6, 298 K): δ 8.94 (s, 2H, NC*H*N), 7.75 (br. s, 4H, Py-C*H*(2,6)), 7.09 (d, $^{3}J_{H–H}$ = 6.9 Hz, 4H, Dipp-Ar-*meta*), 7.04 (t, $^{3}J_{H–H}$ = 7.6 Hz, 4H, Dipp-Ar-*para*), 6.96 (d, $^{3}J_{H–H}$ = 7.4 Hz, 4H, Dipp-Ar-*meta*), 6.35 (br. s, 6H, Py-C*H*(3,4,5)), 4.11 (sep, $^{3}J_{H–H}$ = 6.7 Hz, 4H, Dipp-C*H*(CH_3)$_2$), 3.73 (sep, $^{3}J_{H–H}$ = 6.7 Hz, 4H, Dipp-C*H*(CH_3)$_2$), 1.35 (d, $^{3}J_{H–H}$ = 6.9 Hz, 12H, Dipp-CH(C*H*$_3$)$_2$), 1.30 (d, $^{3}J_{H–}$

$_{\mathrm{H}}$ = 6.7 Hz, 12H, Dipp-CH(CH_3)$_2$), 1.25 (d, $^3J_{\mathrm{H–H}}$ = 6.9 Hz, 12H, Dipp-CH(CH_3)$_2$), 0.65 (d, 3$J_{\mathrm{H–H}}$ = 6.9 Hz, 12H, Dipp-CH(CH_3)$_2$). **^{13}C{^{1}H} NMR (126 MHz, C_6D_6, 298 K):** δ 166.2 (s, 2C, N*C*HN), 148.9 (br. s, 4C, Py-*C*H(2,6)), 145.6 (s, 4C, Dipp-Ar-*C*), 144.9 (s, 4C, Dipp-Ar-*C*), 144.8 (s, 4C, Dipp-Ar-*C*), 136.7 (br. s, 2C, Py-*C*H(4)), 126.4 (s, 4C, Dipp-Ar-*C*H), 124.5 (s, 4C, Dipp-Ar-*C*H), 124.1 (s, 4C, Dipp-Ar-*C*H), 123.1 (br. s, 4C, Py-*C*H(3,5)), 29.2 (s, 4C, Dipp-*C*H(CH_3)$_2$), 28.4 (s, 4C, Dipp-*C*H(CH_3)$_2$), 27.9 (s, 4C, Dipp-CH(*C*H_3)$_2$), 27.7 (s, 4C, Dipp-CH(*C*H_3)$_2$), 24.8 (s, 4C, Dipp-CH(*C*H_3)$_2$), 23.7 (s, 4C, Dipp-CH(*C*H_3)$_2$). **Anal. Calcd. for $C_{60}H_{80}Cl_2N_6W_2$:** N, 6.35; C, 54.43; H, 6.09. **Found:** N, 5.98; C, 53.75; H, 5.73.

Note: No uncommon hazards are noted.

2.4 W_2(THF)(μ-H)$_2$[μ-κ^3-HC(N-2-CH(CH_2)(CH_3)-6-^{i}PrC_6H_3)(N-2,6-^{i}Pr$_2C_6H_3$)]$_2$ (4)

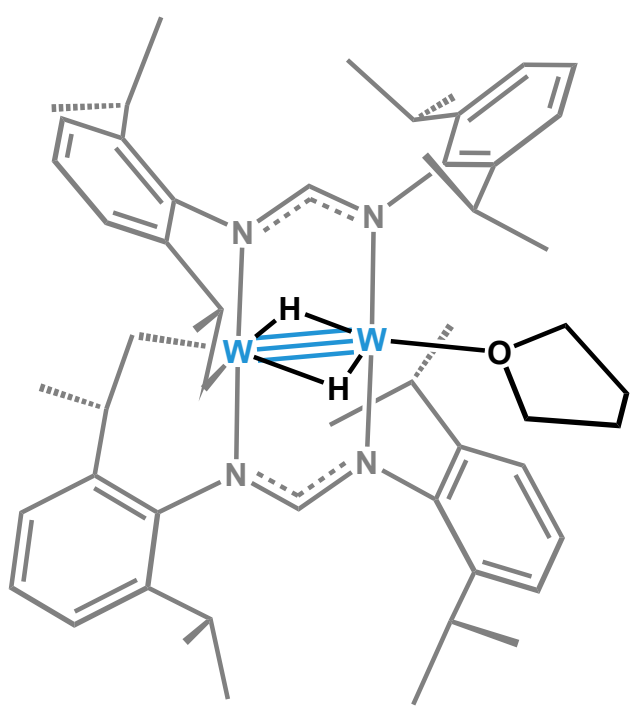

4

A 20 mL vial was charged with **1** (0.0509 g, 0.0332 mmol) and KC_8 (0.0173 g, 0.1280 mmol). THF (4 mL) was added and the mixture was stirred at room temperature for 1 h, during which the rose-red suspension turned green. The mixture was passed through a pad of *Celite* and the residue was washed with THF until the filtrate was colorless. The combined green filtrate was concentrated under reduced pressure to remove volatiles. *n*-Pentane (2 mL) was added and the green suspension was transferred onto a pad of *Celite*; the solid on the pad was washed with *n*-pentane until the filtrate was colorless. The residue was extracted with *n*-pentane:toluene 1:1 mixture and the insoluble materials were removed by filtration. The combined filtrate was concentrated under vacuum to give moss-green **4**·(0.0352 g, 0.0302 mmol, 90.8%). Single crystals suitable for X-ray diffraction were obtained by slow evaporation of *n*-hexane/Et_2O solution at room temperature.

^{1}H NMR (500 MHz, C_6D_6, 298K): δ 8.21 (s, 2H, NC*H*N), 7.26 (d, $^3J_{\mathrm{H–H}}$ = 7.4 Hz, 2H, Dipp-Ar-*meta*), 7.12–7.06 (m, 4H, Dipp-*Ar*), 7.03–6.95 (m, 6H, Dipp-*Ar*), 3.87 (sep, $^3J_{\mathrm{H–H}}$ = 7.5 Hz, 2H, Dipp-C*H*(CH_3)$_2$), 3.62–3.51 (m, 2H, Dipp-C*H*(CH_2)(CH_3)), 3.29 (sep, $^3J_{\mathrm{H–H}}$ = 7.4 Hz, 2H, Dipp-C*H*(CH_3)$_2$), 3.22 (sep, $^3J_{\mathrm{H–H}}$ = 7.3 Hz, 2H, Dipp-C*H*(CH_3)$_2$), 2.64 (dd, $^2J_{\mathrm{H–H}}$ = 13.6 Hz; $^3J_{\mathrm{H–H}}$ = 3.0 Hz, 2H, Dipp-CH(C$H_\alpha H_\beta$)(CH_3)), 2.58 (br. s, 4H, THF-CH_2(2,5)), 1.48 (d, $^3J_{\mathrm{H–H}}$ = 6.7 Hz, 6H, Dipp-CH(CH_3)$_2$), 1.43 (d, $^3J_{\mathrm{H–H}}$ = 6.9 Hz, 12H, Dipp-CH(CH_3)$_2$), 1.40 (d, $^3J_{\mathrm{H–H}}$ = 6.9 Hz, 6H, Dipp-CH(CH_3)$_2$), 1.35 (d, $^3J_{\mathrm{H–H}}$ = 6.7 Hz, 6H, Dipp-CH(CH_2)(CH_3)), 1.14 (d, $^3J_{\mathrm{H–H}}$ = 6.9 Hz, 6H, Dipp-CH(CH_3)$_2$), 1.06 (d, $^3J_{\mathrm{H–H}}$ = 6.9 Hz, 6H, Dipp-

CH(CH_3)$_2$), 0.87 (dd, $^2J_{H-H}$ = 13.6 Hz; $^3J_{H-H}$ = 12.6 Hz, 2H, Dipp-CH(CH$_\alpha$$H_\beta$)(CH$_3$)), 0.56 (br. s, 4H, THF-CH_2(3,4)), –2.23 (dd, $^1J_{W-H}$ = 216.5 Hz; $^1J_{W-H}$ = 52.0 Hz, 2H, W–H). **^{13}C{^{1}H} NMR (126 MHz, C$_6$D$_6$, 298K):** δ 168.2 (s, 2C, NCHN), 149.9 (s, 2C, Dipp-Ar-C), 146.1 (s, 2C, Dipp-Ar-C), 145.8 (s, 2C, Dipp-Ar-C), 143.6 (s, 2C, Dipp-Ar-C), 143.0 (s, 2C, Dipp-Ar-C), 140.9 (s, 2C, Dipp-Ar-C), 125.6 (s, 2C, Dipp-Ar-CH), 125.3 (s, 2C, Dipp-Ar-CH), 124.2 (s, 2C, Dipp-Ar-CH), 123.6 (s, 2C, Dipp-Ar-CH), 123.5 (s, 2C, Dipp-Ar-CH), 122.7 (s, 2C, Dipp-Ar-CH), 72.8 (br. s, 2C, THF-CH$_2$(2,5)), 62.1 (tt, $^1J_{W-C}$ = 81.9 Hz; $^2J_{W-C}$ = 26.7 Hz, 2C, Dipp-CH(CH$_2$)(CH$_3$)), 38.7 (t, $^2J_{W-C}$ = 77.5 Hz, 2C, Dipp-CH(CH$_2$)(CH$_3$)), 30.6 (s, 2C, Dipp-CH(CH$_3$)$_2$), 29.8 (s, 2C, Dipp-CH(CH$_3$)$_2$), 27.7 (s, 2C, Dipp-CH(CH$_2$)(CH$_3$)), 27.6 (s, 2C, Dipp-CH(CH$_3$)$_2$), 27.3 (s, 2C, Dipp-CH(CH$_3$)$_2$), 26.8 (s, 2C, Dipp-CH(CH$_3$)$_2$), 25.8 (s, 2C, Dipp-CH(CH$_3$)$_2$), 25.3 (s, 2C, THF-CH$_2$(3,4)), 25.1 (s, 2C, Dipp-CH(CH$_3$)$_2$), 24.2 (s, 2C, Dipp-CH(CH$_3$)$_2$), 23.4 (s, 2C, Dipp-CH(CH$_3$)$_2$). **Anal. Calcd. for $C_{54}H_{78}N_4OW_2$:** N, 4.80; C, 55.58; H, 6.74; **Found:** N, 4.54; C, 55.26; H, 7.15.

Note: KC$_8$ is pyrophoric. Extreme caution must be exercised during its handling.

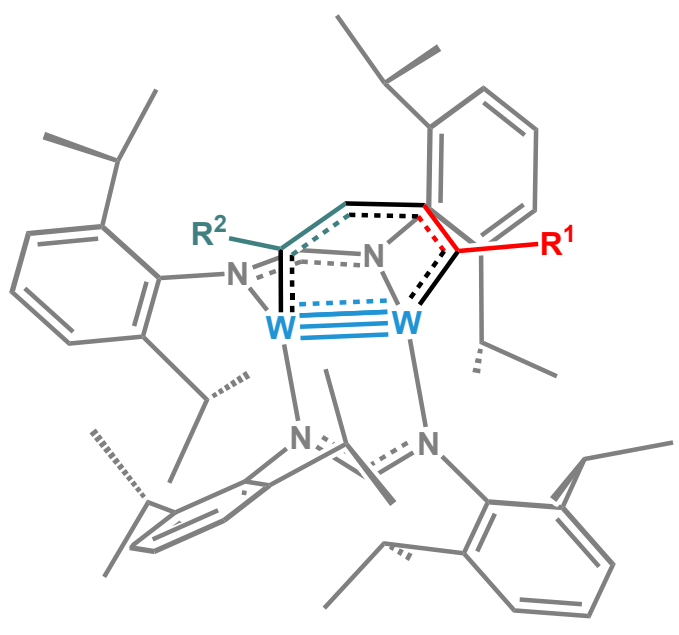

5: R^1 = R^2 = C$_6$H$_5$,

2.5 W$_2$(μ-κ^2-1,4-Ph$_2$C$_4$H$_2$)[μ-κ^2-HC(N-2,6-^{i}Pr$_2$C$_6$H$_3$)$_2$]$_2$ (5)

Method 1: A 20 mL vial was charged with **4** (0.0608 g, 0.0520 mmol). Et$_2$O (8 mL) and phenylacetylene (0.53 mL of a 0.20 M solution in toluene, 0.106 mmol) were added sequentially and the mixture was stirred at room temperature for 12 h, during which the dark green suspension turned brown. All volatiles were then removed under reduced pressure. The brown residue was washed with *n*-hexane, then extracted with Et$_2$O, and the insoluble materials were removed by filtration through a pad of Celite. The filtrate was concentrated under vacuum to afford **5** (0.0119 g, 0.0080 mmol, 16.9%). Single crystals suitable for X-ray diffraction were obtained by recrystallization from THF/toluene solution at −35 °C.

Method 2: A 20 mL vial was charged with **1** (0.0524 g, 0.0342 mmol). Et$_2$O (4 mL), phenylacetylene (0.66 mL of a 0.1 M solution in toluene, 0.0653 mmol) and KC$_8$ (0.0191 g, 0.1413 mmol) were added sequentially and the mixture was stirred at room temperature for 2 h, during which the rose-red suspension turned brown. All volatiles were then removed under reduced pressure. *n*-Hexane (4 mL) was added and the brown suspension was transferred onto a pad of *Celite*; The residue was washed with *n*-hexane until the filtrate was light-red. The residue was extracted with THF and the insoluble materials

were removed by filtration. The combined filtrate was concentrated under vacuum to give brown powder **5**·(0.0265 g, 0.0204 mmol, 62.5%).

^{1}H NMR (500 MHz, C_6D_6, 298 K): δ 8.57 (s, 2H, NC*H*N), 8.56 (s, 2H, $W_2(PhC)_2(CH_2)$), 7.01 (t, $^3J_{H–H}$ = 7.7 Hz, 4H, Dipp-Ar-*para*), 6.96 (d, $^3J_{H–H}$ = 7.8 Hz, 4H, Dipp-Ar-*meta*), 6.90 (dd, $^3J_{H–H}$ = 7.5 Hz; $^4J_{H–H}$ = 1.7 Hz, 4H, Dipp-Ar-*meta*), 6.85 (t, $^3J_{H–H}$ = 7.5 Hz, 4H, $W_2(PhC)_2(CH_2)$-Ar-*meta*), 6.72 (t, $^3J_{H–H}$ = 7.3 Hz, 2H, $W_2(PhC)_2(CH_2)$-Ar-*para*), 6.26 (d, $^3J_{H–H}$ = 7.7 Hz, 4H, $W_2(PhC)_2(CH_2)$-Ar-*ortho*), 3.80 (sep, $^3J_{H–H}$ = 6.8 Hz, 4H, Dipp-C*H*$(CH_3)_2$), 2.65 (sep, $^3J_{H–H}$ = 6.9 Hz, 4H, Dipp-C*H*$(CH_3)_2$), 1.31 (d, $^3J_{H–H}$ = 6.7 Hz, 12H, Dipp-CH$(CH_3)_2$), 1.01 (d, $^3J_{H–H}$ = 6.8 Hz, 12H, Dipp-CH$(CH_3)_2$), 0.86 (d, $^3J_{H–H}$ = 6.6 Hz, 12H, Dipp-CH$(CH_3)_2$), 0.71 (d, $^3J_{H–H}$ = 6.6 Hz, 12H, Dipp-CH$(CH_3)_2$). **^{13}C{^{1}H} NMR (126 MHz, C_6D_6, 298 K):** δ 230.8 (s, 2C, $W_2(PhC)_2(CH_2)$), 172.1 (s, 2C, N*C*HN), 146.2 (s, 4C, Dipp-Ar-*C*), 146.1 (s, 4C, $W_2(PhC)_2(CH_2)$-Ar-*C*), 142.7 (s, 4C, Dipp-Ar-*C*), 142.5 (s, 4C, Dipp-Ar-*C*), 137.8 (s, 2C, $W_2(PhC)_2(CH_2)$),129.0 (s, 4C, Dipp-Ar-*meta*), 127.0 (s, 4C, $W_2(PhC)_2(CH_2)$-Ar-*para*), 125.3 (s, 4C, $W_2(PhC)_2(CH_2)$-Ar-*ortho*), 124.7 (s, 4C, Dipp-Ar-*meta*), 123.7 (s, 2C, $W_2(PhC)_2(CH_2)$-Ar-*para*), 125.6 (s, 2C, Dipp-Ar-*C*H), 123.7 (s, 4C, $W_2(PhC)_2(CH_2)$-Ar-para), 123.5 (s, 4C, Dipp-Ar-CH), 30.6 (s, 4C, Dipp-*C*H$(CH_3)_2$), 27.6 (s, 4C, Dipp-*C*H$(CH_3)_2$), 27.2 (s, 4C, Dipp-CH$(CH_3)_2$), 26.8 (s, 4C, Dipp-CH$(CH_3)_2$), 24.6 (s, 4C, Dipp-CH$(CH_3)_2$), 22.5 (s, 4C, Dipp-CH$(CH_3)_2$). **Anal. Calcd. For $C_{66}H_{82}N_4W_2$:** N: 4.31, C: 61.02, H: 6.36. **Found:** N: 4.29, C: 60.97, H: 6.934.

Note: KC_8 is pyrophoric. Extreme caution must be exercised during its handling.

2.6 W_2(μ-κ^2-1,4-(2-MePh)$_2C_4H_2$)[μ-κ^2-HC(N-2,6-^{i}Pr$_2C_6H_3)_2$]$_2$ (6)

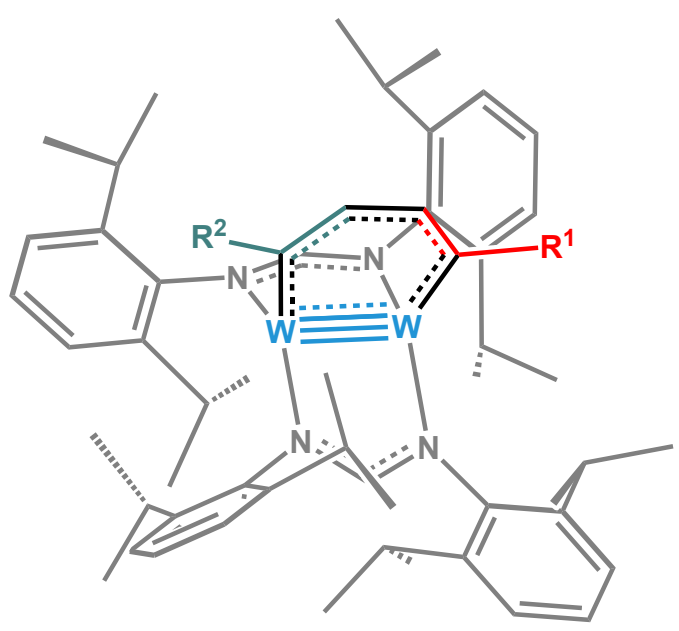

6: R^1 = R^2 = 2-MeC$_6$H$_4$

A 20 mL vial was charged with **1** (0.0470 g, 0.0307 mmol). Et_2O (6 mL) and KC_8 (0.0081 g, 0.0599 mmol) were added sequentially and the mixture was stirred at room temperature for 30 min, during which the red suspension turned orange. The suspension was transferred onto a pad of *Celite*; The residue was extracted with Et_2O until the filtrate was colorless and the insoluble materials were removed by filtration. 2-methylphenylacetylene (0.66 mL of a 0.1 M solution in toluene, 0.0653 mmol) was then added to the filtrate and the mixture was stirred at room temperature for 1.5 h during which the orange suspension turned olive green. KC_8 (0.0096 g, 0.0710 mmol) was then added, and stirring was continued for 3 h at room temperature, during which the green suspension turned green yellow. All volatiles were then

removed under reduced pressure. *n*-Hexane (1 mL) was added and the suspension was transferred onto a pad of *Celite*; The residue was washed with *n*-hexane (1 mL). The residue was extracted with THF and the insoluble materials were removed by filtration. The filtrate was concentrated under vacuum to give chrome-yellow powder **6**·(0.0363 g, 0.0274 mmol, 89.2%). Single crystals suitable for X-ray diffraction were obtained by recrystallization from *n*-hexane at −35 °C.

^{1}H NMR (500 MHz, C_6D_6, 333 K): δ 8.49 (s, 2H, NC*H*N), 8.10 (s, 2H, W_2(2-MePhC)$_2$(C*H*$_2$)), 7.30 (d, $^3J_{H–H}$ = 7.6 Hz, 4H, Dipp-Ar-*meta*), 7.06 (t, $^3J_{H–H}$ = 7.6 Hz, 2H, Dipp-Ar-*para*), 7.02 (d, $^3J_{H–H}$ = 6.1 Hz, 4H, Dipp-Ar-*meta*), 6.95 (d, $^3J_{H–H}$ = 7.4 Hz, 4H, Dipp-Ar-*meta*), 6.77 (t, $^3J_{H–H}$ = 7.4 Hz, 2H, W_2(2-MePhC)$_2$(CH_2)-Ar-C*H*), 6.36 (t, $^3J_{H–H}$ = 7.5 Hz, 2H, W_2(2-MePhC)$_2$(CH_2)-Ar-C*H*), 5.25 (d, $^3J_{H–H}$ = 7.6 Hz, 2H, W_2(MePhC)$_2$(CH_2)-Ar-*ortho*), 3.96–3.72 (m, 4H, Dipp-C*H*(CH_3)$_2$), 2.68–2.58 (m, 4H, Dipp-C*H*(CH_3)$_2$), 1.30 (d, $^3J_{H–H}$ = 6.7 Hz, 12H, Dipp-CH(C*H*$_3$)$_2$), 0.91 (d, $^3J_{H–H}$ = 6.7 Hz, 12H, Dipp-CH(C*H*$_3$)$_2$), 0.89–0.75 (m, 12H, Dipp-CH(C*H*$_3$)$_2$), 0.68 (d, $^3J_{H–H}$ = 6.7 Hz, 12H, Dipp-CH(C*H*$_3$)$_2$). **^{13}C{^{1}H} NMR (126 MHz, C_6D_6, 333 K):** δ 232.6 (s, 2C, W_2(2-MePh*C*)$_2$(CH_2)), 173.6 (s, 2C, N*C*HN), 146.5 (s, 2C, Dipp-Ar-*C*), 146.2 (s, 2C, W_2(2-MePh*C*)$_2$(CH_2)-Ar-*C*), 143.8 (s, 4C, Dipp-Ar-*ortho*), 143.2 (s, 4C, Dipp-Ar-*ortho*), 139.8 (s, 2C, W_2(2-MePhC)$_2$(*C*H$_2$)), 133.9 (s, 2C, W_2(MePhC)$_2$(CH_2)-Ar-*meta*), 133.3 (s, 2C, W_2(MePhC)$_2$(CH_2)-Ar- *ortho*), 130.5 (s, 2C, W_2(MePhC)$_2$(CH_2)-Ar-*meta*), 127.2 (s, 2C, W_2(MePhC)$_2$(CH_2)-Ar-*para*), 125.2 (s, 2C, W_2(MePhC)$_2$(CH_2)-Ar-*ortho*), 124.8 (s, 4C, Dipp-Ar-*meta*), 124.0 (s, 2C, W_2(MePhC)$_2$(CH_2)-Ar-*para*),123.9 (s, 4C, Dipp-Ar-*meta*), 30.5 (s, 4C, Dipp-*C*H(CH_3)$_2$), 27.5 (s, 4C, Dipp-*C*H(CH_3)$_2$), 26.8 (s, 4C, Dipp-CH(*C*H$_3$)$_2$), 26.6 (s, 4C, Dipp-CH(*C*H$_3$)$_2$), 25.7 (s, 4C, Dipp-CH(*C*H$_3$)$_2$), 23.1 (s, 2C, W_2(*Me*PhC)$_2$(CH)). **Anal. Calcd. for $C_{68}H_{86}N_4W_2$:** N, 4.22; C, 61.54; H, 6.53. **Found:** N, 3.72; C, 61.04; H, 6.28.

Note: KC_8 is pyrophoric. Extreme caution must be exercised during its handling.

2.7 W_2(μ-κ^2-1,4-(3-MePh)$_2C_4H_2$)[μ-κ^2-HC(N-2,6-^{i}Pr$_2C_6H_3$)$_2$]$_2$ (7)

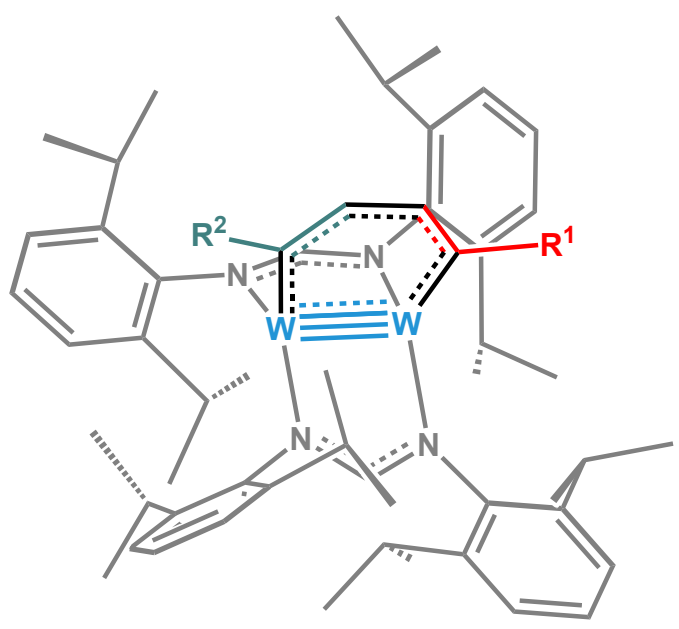

7: R^1 = R^2 =3-MeC$_6$H$_4$

A 20 mL vial was charged with **1** (0.0539 g, 0.0352 mmol). THF (4 mL), 3-methylphenylacetylene (0.66 mL of a 0.1 M solution in toluene, 0.0653 mmol) and KC_8 (0.0185 g, 0.1369 mmol) were added sequentially and the mixture was stirred at room temperature for 2 h, during which the rose-red suspension turned brown. All volatiles were then removed under reduced pressure. *n*-Hexane (4 mL) was added

and the brown suspension was transferred onto a pad of *Celite*; The residue was washed with *n*-hexane until the filtrate was light-red. The residue was extracted with THF and the insoluble materials were removed by filtration. The combined filtrate was concentrated under vacuum to give brown powder **7**·(0.0311 g, 0.0234 mmol, 71.8%). Single crystals suitable for X-ray diffraction were obtained by slow evaporation of THF solution at room temperature.

^{1}H NMR (500 MHz, C_6D_6, 298 K): δ 8.58 (s, 2H, NC*H*N), 8.52 (s, 2H, W_2(3-MePhC)$_2$(C*H*)$_2$), 7.02 (t, $^3J_{H–H}$ = 7.6 Hz, 4H, Dipp-Ar-*para*), 6.96 (dd, $^3J_{H–H}$ = 7.8 Hz; $^4J_{H–H}$ = 1.9 Hz, 4H, Dipp-Ar-*meta*), 6.92 (d, $^3J_{H–H}$ = 7.5 Hz; $^4J_{H–H}$ = 1.8 Hz, 4H, Dipp-Ar-*meta*), 6.88 (t, $^3J_{H–H}$ = 7.6 Hz, 2H, W_2(3-MePhC)$_2$(CH)$_2$-Ar-*meta*), 6.60 (d, $^3J_{H–H}$ = 7.8 Hz, 2H, W_2(3-MePhC)$_2$(CH)$_2$-Ar-*para*), 6.36 (d, $^3J_{H–H}$ = 7.6 Hz, 2H, W_2(3-MePhC)$_2$(CH)$_2$-Ar-*ortho*), 6.04 (s, 2H, W_2(3-MePhC)$_2$(CH)$_2$-Ar-*ortho*), 3.82 (sep, $^3J_{H–H}$ = 6.7 Hz, 4H, Dipp-*C*H(CH$_3$)$_2$), 2.65 (sep, $^3J_{H–H}$ = 6.8 Hz, 4H, Dipp-*C*H(CH$_3$)$_2$), 1.85 (s, 6H, W_2(3-*Me*PhC)$_2$(CH)$_2$), 1.32 (d, $^3J_{H–H}$ = 6.7 Hz, 12H, Dipp-CH(*C*H$_3$)$_2$), 1.01 (d, $^3J_{H–H}$ = 6.9 Hz, 12H, Dipp-CH(*C*H$_3$)$_2$), 0.87 (d, $^3J_{H–H}$ = 6.9 Hz, 12H, Dipp-CH(*C*H$_3$)$_2$), 0.75 (d, $^3J_{H–H}$ = 6.5 Hz, 12H, Dipp-CH(*C*H$_3$)$_2$). **^{13}C{^{1}H} NMR (126 MHz, C_6D_6, 298 K):** δ 231.6 (s, 2C, W_2(3-MePh*C*)$_2$(CH)$_2$), 172.1 (s, 2C, N*C*HN), 146.3 (s, 4C, Dipp-Ar-*C*), 146.2 (s, 2C, W_2(3-MePhC)$_2$(CH)$_2$-Ar-*C*), 143.1 (s, 4C, Dipp-Ar-*ortho*), 142.5 (s, 4C, Dipp-Ar-*ortho*), 138.0 (s, 2C, W_2(3-MePhC)$_2$(CH)$_2$-Ar-*meta*), 137.7 (s, 2C, W_2(3-MePhC)$_2$(*C*H)$_2$), 129.1 (s, 2C, W_2(3-MePhC)$_2$(CH)$_2$-Ar-*meta*), 127.0 (s, 4C, Dipp-Ar-*para*), 126.8 (s, 2C, W_2(3-MePhC)$_2$(CH)$_2$-Ar-*ortho*), 124.7 (s, 2C, W_2(3-MePhC)$_2$(CH)$_2$-Ar-*ortho*), 124.6 (s, 4C, Dipp-Ar-meta), 123.5 (s, 4C, Dipp-Ar-*meta*), 122.7 (s, 2C, W_2(3-MePhC)$_2$(CH)$_2$-Ar-*para*), 30.6 (s, 4C, Dipp-CH(*C*H$_3$)$_2$), 27.6 (s, 4C, Dipp-CH(*C*H$_3$)$_2$), 27.1 (s, 4C, Dipp-CH(*C*H$_3$)$_2$), 26.9 (s, 4C, Dipp-CH(CH$_3$)$_2$), 24.7 (s, 4C, Dipp-CH(*C*H$_3$)$_2$), 22.6 (s, 4C, Dipp-CH(CH$_3$)$_2$), 21.8 (s, 2C, W_2(3-*Me*PhC)$_2$(CH)$_2$). **Anal. Calcd. for $C_{68}H_{86}N_4W_2$:** N, 4.22; C, 61.54; H, 6.53. **Found:** N, 4.14; C, 60.96; H, 6.54.

Note: KC_8 is pyrophoric. Extreme caution must be exercised during its handling.

2.8 W_2(μ-κ^2-1,4-(4-MePh)$_2$C$_4$H$_2$)[μ-κ^2-HC(N-2,6-^{i}Pr$_2$C$_6$H$_3$)$_2$]$_2$ (8)

A 20 mL vial was charged with **1** (0.0539 g, 0.0352 mmol). THF (4 mL), 4-methylphenylacetylene (0.66 mL of a 0.1 M solution in toluene, 0.0653 mmol) and KC_8 (0.0188 g, 0.1391 mmol) were added sequentially and the mixture was stirred at room temperature for 2 h, during which the rose-red

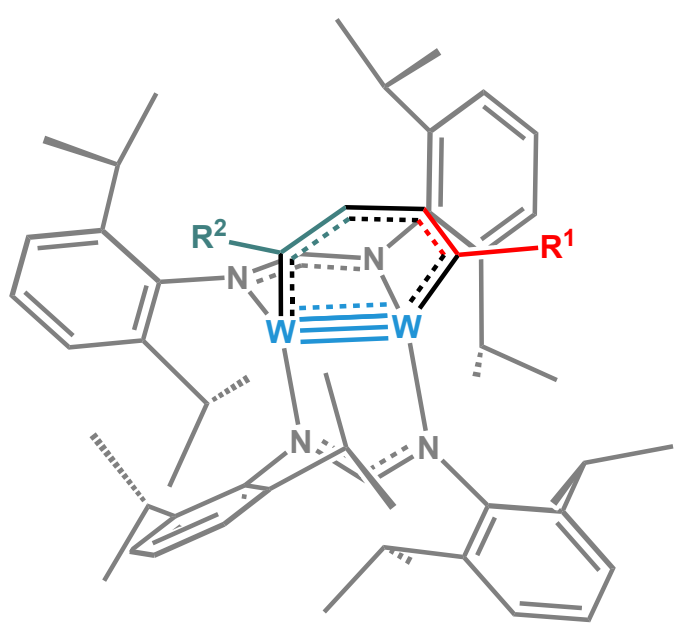

8: $R^1 = R^2 = 4\text{-}MeC_6H_4$

suspension turned brown. All volatiles were then removed under reduced pressure. *n*-Hexane (4 mL) was added and the brown suspension was transferred onto a pad of *Celite*; The residue was washed with *n*-hexane until the filtrate was colorless. The residue was extracted with THF and the insoluble materials were removed by filtration. The combined filtrate was concentrated under vacuum to give brown powder **8**·(0.0257 g, 0.0194 mmol, 59.9%). Single crystals suitable for X-ray diffraction were obtained by slow evaporation of Et_2O solution at room temperature.

^{1}H NMR (500 MHz, C_6D_6, 298 K): δ 8.59 (s, 2H, NC*H*N), 8.54 (s, 2H, W_2(4-MePhC)$_2$(C*H*)$_2$), 7.03 (t, $^3J_{H\text{–}H}$ = 7.6 Hz, 4H, Dipp-Ar-*para*), 6.98 (dd, $^3J_{H\text{–}H}$ = 7.8 Hz; $^4J_{H\text{–}H}$ = 1.9 Hz, 4H, Dipp-Ar-*meta*), 6.91 (dd, $^3J_{H\text{–}H}$ = 7.5 Hz; $^4J_{H\text{–}H}$ = 1.8 Hz, 4H, Dipp-Ar-*meta*), 6.70 (d, $^3J_{H\text{–}H}$ = 8.0 Hz, 4H, W_2(4-MePhC)$_2$(CH)$_2$-Ar-*meta*), 6.22 (d, $^3J_{H\text{–}H}$ = 8.2 Hz, 4H, W_2(4-MePhC)$_2$(CH)$_2$-Ar-*ortho*), 3.83 (sep, $^3J_{H\text{–}H}$ = 6.8 Hz, 4H, Dipp-C*H*(CH$_3$)$_2$), 2.67 (sep, $^3J_{H\text{–}H}$ = 6.6 Hz, 4H, Dipp-C*H*(CH$_3$)$_2$), 2.07 (s, 6H, W_2(4-*Me*PhC)$_2$(CH)$_2$), 1.32 (d, $^3J_{H\text{–}H}$ = 6.7 Hz, 12H, Dipp-CH(C*H*$_3$)$_2$), 1.03 (d, $^3J_{H\text{–}H}$ = 6.9 Hz, 12H, Dipp-CH(C*H*$_3$)$_2$), 0.87 (d, $^3J_{H\text{–}H}$ = 7.1 Hz, 12H, Dipp-CH(C*H*$_3$)$_2$), 0.75 (d, $^3J_{H\text{–}H}$ = 6.5 Hz, 12H, Dipp-CH(C*H*$_3$)$_2$). **^{13}C{^{1}H} NMR (126 MHz, C_6D_6, 298 K):** δ 230.9 (s, 2C, W_2(4-MePh*C*)$_2$(CH)$_2$), 171.9 (s, 2C, N*C*HN), 146.2 (s, 4C, Dipp-Ar-*C*), 143.1 (s, 2C, W_2(4-MePhC)$_2$(CH)$_2$-Ar-*C*), 143.0 (s, 4C, Dipp-Ar-*ortho*), 142.6 (s, 4C, Dipp-Ar-*ortho*), 137.7 (s, 2C, W_2(4-MePhC)$_2$(*C*H)$_2$), 132.6 (s, 2C, W_2(4-MePhC)$_2$(CH)$_2$-Ar-*para*), 129.8 (s, 4C, W_2(4-MePhC)$_2$(CH)$_2$-Ar-*meta*), 126.9 (s, 4C, Dipp-Ar-*para*), 125.4 (s, 4C, W_2(4-MePhC)$_2$(CH)$_2$-Ar-o*rtho*), 124.7 (s, 4C, Dipp-Ar-*meta*), 123.5 (s, 4C, Dipp-Ar-*meta*), 30.6 (s, 4C, Dipp-*C*H(CH$_3$)$_2$), 27.6 (s, 4C, Dipp-*C*H(CH$_3$)$_2$), 27.2 (s, 4C, Dipp-CH(*C*H$_3$)$_2$), 26.8 (s, 4C, Dipp-CH(*C*H$_3$)$_2$), 24.6 (s, 4C, Dipp-CH(*C*H$_3$)$_2$), 22.6 (s, 4C, Dipp-CH(*C*H$_3$)$_2$), 20.9 (s, 2C, W_2(4-*Me*PhC)$_2$(CH)$_2$). **Anal. Calcd. for $C_{68}H_{86}N_4W_2$:** N, 4.22; C, 61.54; H, 6.53. **Found:** N, 4.16; C, 61.08; H, 6.41.

Note: KC_8 is pyrophoric. Extreme caution must be exercised during its handling.

2.9 W_2(μ-κ^2-1,4-(4-OMePh)$_2C_4H_2$)[μ-κ^2-HC(N-2,6-^{i}Pr$_2C_6H_3$)$_2$]$_2$ (9)

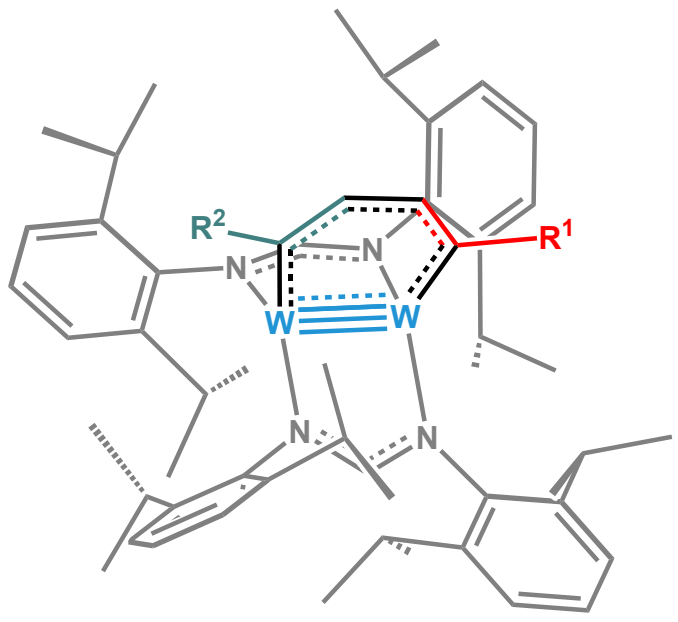

9: $R^1 = R^2 = 4\text{-}MeOC_6H_4$

A 20 mL vial was charged with **1** (0.0503 g, 0.0328 mmol). THF (4 mL), 4-methoxyphenylacetylene (0.66 mL of a 0.1 M solution in toluene, 0.0653 mmol) and KC_8 (0.0178 g, 0.1371 mmol) were added sequentially and the mixture was stirred at room temperature for 2 h, during which the rose-red suspension turned brown. All volatiles were then removed under reduced pressure. *n*-Hexane (4 mL) was added and the brown suspension was transferred onto a pad of *Celite*; The residue was washed with Et_2O until the filtrate was light green. The residue was extracted with THF and the insoluble materials were removed by filtration. The combined filtrate was concentrated under vacuum to give brown powder **9**·(0.0376 g, 0.0277 mmol, 84.8%). Single crystals suitable for X-ray diffraction were obtained by slow evaporation of THF/*n*-pentane solution at room temperature.

^{1}H NMR (500 MHz, C_6D_6, 298 K): δ 8.54 (s, 2H, NC*H*N), 8.52 (s, 2H, W_2(4-MeOPhC)$_2$(C*H*)$_2$), 7.02 (t, $^3J_{H–H}$ = 7.7 Hz, 4H, Dipp-Ar-*para*), 6.96 (dd, $^3J_{H–H}$ = 7.8 Hz; $^4J_{H–H}$ = 1.7 Hz, 4H, Dipp-Ar-*meta*), 6.92 (dd, $^3J_{H–H}$ = 7.5 Hz; $^4J_{H–H}$ = 1.8 Hz, 4H, Dipp-Ar-*meta*), 6.53 (d, $^3J_{H–H}$ = 8.8 Hz, 4H, W_2(4-MeOPhC)$_2$(CH)$_2$-Ar-*meta*), 6.25 (d, $^3J_{H–H}$ = 8.6 Hz, 4H, W_2(4-MeOPhC)$_2$(CH)$_2$-Ar-*ortho*), 3.82 (sep, $^3J_{H–H}$ = 6.5 Hz, 4H, Dipp-C*H*(CH$_3$)$_2$), 3.27 (s, 6H, W_2(4-*Me*OPhC)$_2$(CH)$_2$), 2.69 (sep, $^3J_{H–H}$ = 6.7 Hz, 4H, Dipp-C*H*(CH$_3$)$_2$), 1.31 (d, $^3J_{H–H}$ = 6.7 Hz, 12H, Dipp-CH(C*H*$_3$)$_2$), 1.01 (d, $^3J_{H–H}$ = 6.7 Hz, 12H, Dipp-CH(C*H*$_3$)$_2$), 0.89 (d, $^3J_{H–H}$ = 6.9 Hz, 12H, Dipp-CH(C*H*$_3$)$_2$), 0.80 (d, $^3J_{H–H}$ = 6.5 Hz, 12H, Dipp-CH(C*H*$_3$)$_2$). **^{13}C{^{1}H} NMR (126 MHz, C_6D_6, 298 K):** δ 230.7 (s, 2C, W_2(4-MeOPh*C*)$_2$(CH)$_2$), 171.6 (s, 2C, N*C*HN), 157.1 (s, 2C, W_2(4-MeOPhC)$_2$(CH)$_2$-Ar-*para*), 146.1 (s, 4C, Dipp-Ar-*C*), 143.0 (s, 4C, Dipp-Ar-*ortho*), 142.5 (s, 4C, Dipp-Ar-*ortho*), 138.7 (s, 2C, W_2(4-MeOPhC)$_2$(CH)$_2$-Ar-*C*), 137.8 (s, 2C, W_2(4-MeOPhC)$_2$(*C*H)$_2$), 126.9 (s, 4C, Dipp-Ar-*para*), 126.7 (s, 4C, W_2(4-MeOPhC)$_2$(CH)$_2$-Ar-*meta*), 124.7 (s, 4C, Dipp-Ar-*meta*), 123.5 (s, 4C, Dipp-Ar-*meta*), 114.7 (s, 4C, W_2(4-MeOPhC)$_2$(CH)$_2$-Ar-*ortho*), 54.7 (s, 2C, W_2(4-*Me*OPhC)$_2$(CH)$_2$), 30.6 (s, 4C, Dipp-*C*H(CH$_3$)$_2$), 27.6 (s, 4C, Dipp-*C*H(CH$_3$)$_2$), 27.2 (s, 4C, Dipp-CH(*C*H$_3$)$_2$), 26.8 (s, 4C, Dipp-CH(*C*H$_3$)$_2$), 24.6 (s, 4C, Dipp-CH(*C*H$_3$)$_2$), 22.7 (s, 4C, Dipp-CH(*C*H$_3$)$_2$). **Anal. Calcd. for $C_{68}H_{86}N_4O_2W_2$:** N, 4.12; C, 60.09; H, 6.38. **Found:** N, 4.19; C, 60.02; H, 6.61.

Note: KC_8 is pyrophoric. Extreme caution must be exercised during its handling.

2.10 W_2(μ-κ^2-1,4-(4-FPh)$_2C_4H_2$)[μ-κ^2-HC(N-2,6-^{i}Pr$_2C_6H_3$)$_2$]$_2$ (10)

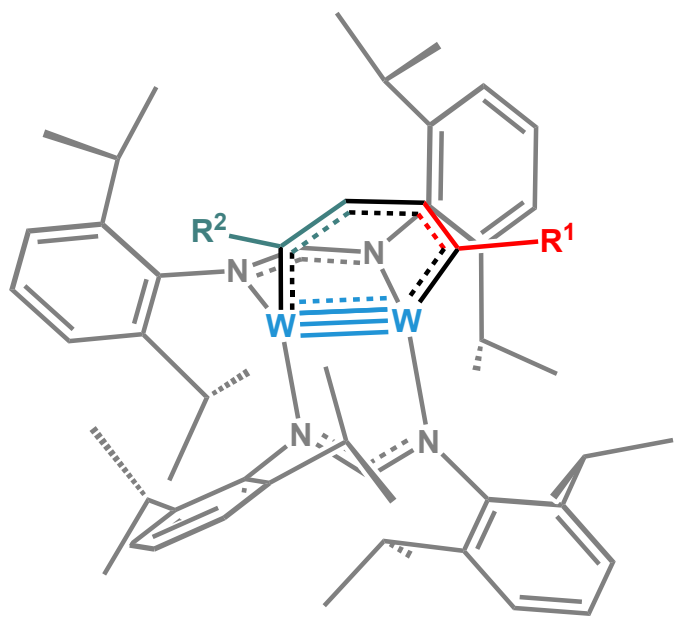

10: $R^1 = R^2 = 4\text{-}FC_6H_4$

A 20 mL vial was charged with **1** (0.0482 g, 0.0315 mmol). THF (4 mL), 4-fluorophenylacetylene (0.64 mL of a 0.1 M solution in toluene, 0.0653 mmol) and KC_8 (0.0173 g, 0.1280 mmol) were added sequentially and the mixture was stirred at room temperature for 2 h, during which the rose-red suspension turned brown. All volatiles were then removed under reduced pressure. *n*-Hexane (4 mL) was added and the brown suspension was transferred onto a pad of *Celite*; The residue was washed with *n*-hexane until the filtrate was colorless. The residue was extracted with THF and the insoluble materials were removed by filtration. The combined filtrate was concentrated under vacuum to give brown powder **10**·(0.0368 g, 0.0276 mmol, 87.6%). Single crystals suitable for X-ray diffraction were obtained by slow evaporation of THF/toluene solution at room temperature.

^{1}H NMR (500 MHz, C_6D_6, 298 K): δ 8.52 (s, 2H, NC*H*N), 8.36 (s, 2H, W_2(4-FPhC)$_2$(C*H*)$_2$), 6.99 (t, $^3J_{H–H}$ = 7.7 Hz, 4H, Dipp-Ar-*para*), 6.91 (dd, $^3J_{H–H}$ = 7.7 Hz; $^4J_{H–H}$ = 1.7 Hz, 4H, Dipp-Ar-*meta*), 6.87 (dd, $^3J_{H–H}$ = 7.6 Hz; $^4J_{H–H}$ = 1.7 Hz, 4H, Dipp-Ar-*meta*), 6.60 (dd, $^3J_{H–H}$ = 8.7 Hz; $^3J_{H–F}$ = 7.0 Hz, 4H, W_2(4-FPhC)$_2$(CH)$_2$-Ar-*meta*), 6.13 (dd, $^3J_{H–H}$ = 8.6 Hz; $^4J_{H–F}$ = 5.4 Hz, 4H, W_2(4-FPhC)$_2$(CH)$_2$-Ar-*ortho*), 3.71 (sep, $^3J_{H–H}$ = 6.8 Hz, 4H, Dipp-C*H*(CH_3)$_2$), 2.58 (sep, $^3J_{H–H}$ = 6.8 Hz, 4H, Dipp-C*H*(CH_3)$_2$), 1.27 (d, $^3J_{H–H}$ = 6.5 Hz, 12H, Dipp-CH(C*H*$_3$)$_2$), 0.93 (d, $^3J_{H–H}$ = 6.8 Hz, 12H, Dipp-CH(C*H*$_3$)$_2$), 0.84 (d, $^3J_{H–H}$ = 6.9 Hz, 12H, Dipp-CH(C*H*$_3$)$_2$), 0.70 (d, $^3J_{H–H}$ = 6.5 Hz, 12H, Dipp-CH(C*H*$_3$)$_2$). **^{13}C{^{1}H} NMR (126 MHz, C_6D_6, 298 K):** δ 230.0 (s, 2C, W_2(4-FPh*C*)$_2$(CH)$_2$), 172.2 (s, 2C, N*C*HN), 160.8 (d, 2C, $^1J_{C–F}$ = 242.2 Hz, W_2(4-FPhC)$_2$(CH)$_2$-Ar-*para*), 145.9 (s, 4C, Dipp-Ar-*C*), 142.6 (s, 4C, Dipp-Ar-*ortho*), 142.3 (d, $^4J_{C–F}$ = 3.4 Hz, 2C, W_2(4-FPhC)$_2$(CH)$_2$-Ar-*C*), 127.1 (s, 4C, Dipp-Ar-*para*), 127.0 (d, $^3J_{C–F}$ = 7.2 Hz, 4C, W_2(4-FPhC)$_2$(CH)$_2$-Ar-*ortho*), 124.7 (s, 4C, Dipp-Ar-*meta*), 123.6 (s, 4C, Dipp-Ar-*meta*), 115.7 (d, $^2J_{C–F}$ = 20.4 Hz, 4C, W_2(4-FPhC)$_2$(CH)$_2$-Ar-*meta*), 30.7 (s, 4C, Dipp-*C*H(CH_3)$_2$), 27.5 (s, 4C, Dipp-*C*H(CH_3)$_2$), 27.1 (s, 4C, Dipp-CH(*C*H$_3$)$_2$), 26.7 (s, 4C, Dipp-CH(*C*H$_3$)$_2$), 24.5 (s, 4C, Dipp-CH(*C*H$_3$)$_2$), 22.5 (s, 4C, Dipp-CH(*C*H$_3$)$_2$). **Anal. Calcd. for $C_{66}H_{80}F_2N_4W_2$:** N, 4.20; C, 59.38; H, 6.04. **Found:** N, 4.51; C, 60.09; H, 6.04.

Note: KC_8 is pyrophoric. Extreme caution must be exercised during its handling.

2.11 W_2(μ-κ²-1,4-(*n*-C_3H_7)$_2C_4H_2$)[μ-κ²-HC(N-2,6-^{i}Pr$_2C_6H_3$)$_2$]$_2$ (11)

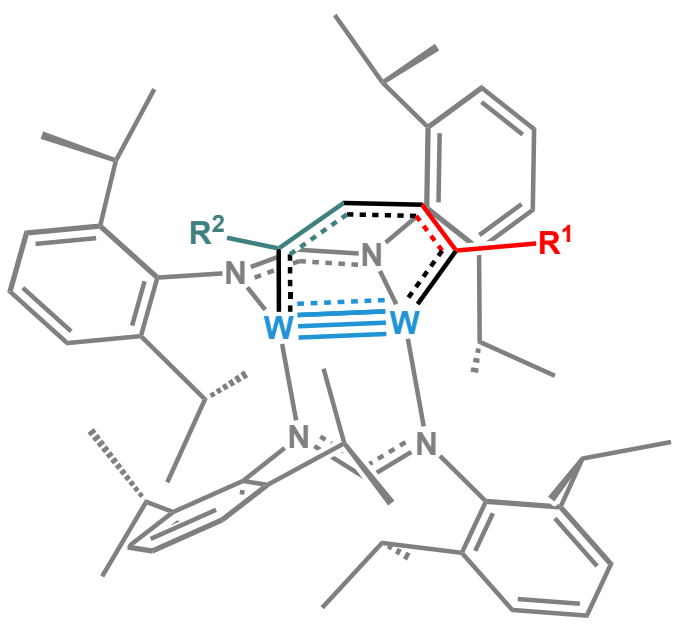

11: R^1 = R^2 = *n*-C_3H_7

A 20 mL vial was charged with **1** (0.0540 g, 0.0352 mmol). THF (4 mL), 1-pentyne (0.64 mL of a 0.1 M solution in toluene, 0.0653 mmol) and KC_8 (0.0180 g, 0.1332 mmol) were added sequentially and the mixture was stirred at room temperature for 3 h, during which the rose-red suspension turned brown. All volatiles were then removed under reduced pressure. *n*-Hexane (4 mL) was added and the brown suspension was transferred onto a pad of *Celite*; The residue was washed with Et_2O until the filtrate was colorless. The residue was extracted with THF and the insoluble materials were removed by filtration. The combined green-brown filtrate was concentrated under vacuum to give green powder **11**·(0.0283 g, 0.0230 mmol, 70.4%). Single crystals suitable for X-ray diffraction were obtained by slow evaporation of toluene solution at room temperature.

^{1}H NMR (500 MHz, C_6D_6, 298 K): δ 8.31 (s, 2H, NC*H*N), 8.22 (s, 2H, W_2(^{n}PrC)$_2$(C*H*)$_2$), 7.07–7.00 (m, 8H, Dipp-Ar-C*H*), 6.95 (dd, $^3J_{H–H}$ = 6.9 Hz; $^4J_{H–H}$ = 3.4 Hz, 4H, Dipp-Ar-*meta*), 3.69 (sep, $^3J_{H–H}$ = 6.9 Hz, 4H, Dipp-C*H*(CH_3)$_2$), 2.92 (sep, $^3J_{H–H}$ = 6.7 Hz, 4H, Dipp-C*H*(CH_3)$_2$), 2.72 (t, $^3J_{H–H}$ = 8.4 Hz, 4H, W_2(^{n}PrC)$_2$(CH)$_2$-CH_2(CH_2)(CH_3)), 1.28 (d, $^3J_{H–H}$ = 6.5 Hz, 12H, Dipp-CH(CH_3)$_2$), 1.06 (d, $^3J_{H–H}$ = 6.9 Hz, 12H, Dipp-CH(CH_3)$_2$), 1.02–0.96 (m, 16H, Dipp-CH(CH_3)$_2$ + W_2(^{n}PrC)$_2$(CH)$_2$-CH_2(CH_2)(CH_3)), 0.93 (d, $^3J_{H–H}$ = 6.5 Hz, 12H, Dipp-CH(CH_3)$_2$), 0.80 (t, $^3J_{H–H}$ = 7.1 Hz, 6H, W_2(^{n}PrC)$_2$(CH)$_2$-CH_2(CH_2)(CH_3)). **^{13}C{^{1}H} NMR (126 MHz, C_6D_6, 298 K):** δ 231.0 (s, 2C, W_2(^{n}Pr*C*)$_2$(CH)$_2$), 168.3 (s, 2C, N*C*HN), 144.7 (s, 4C, Dipp-Ar-*C*), 143.4 (s, 4C, Dipp-Ar-*ortho*), 142.5 (s, 4C, Dipp-Ar-*ortho*), 134.4 (s, 2C, W_2(^{n}Pr*C*)$_2$(*C*H)$_2$), 126.4 (s, 4C, Dipp-Ar-*C*H), 124.7 (s, 4C, Dipp-Ar-*C*H), 123.6 (s, 4C, Dipp-Ar-*meta*), 34.8 (s, 2C, W_2(^{n}PrC)$_2$(CH)$_2$-CH_2(CH_2)(CH_3)), 31.4 (s, 4C, Dipp-*C*H(CH_3)$_2$), 27.1 (s, 4C, Dipp-*C*H(CH_3)$_2$), 26.7 (s, 2C, W_2(^{n}PrC)$_2$(CH)$_2$-CH_2(*C*H_2)(CH_3)), 26.3 (s, 4C, Dipp-CH(*C*H_3)$_2$), 24.5 (s, 4C, Dipp-CH(*C*H_3)$_2$), 23.5 (s, 4C, Dipp-CH(CH_3)$_2$), 15.7 (s, 2C, W_2(^{n}PrC)$_2$(CH)$_2$-CH_2(CH_2)(*C*H_3)). **Anal. Calcd. for $C_{60}H_{86}N_4W_2$:** N, 4.55; C, 58.54; H, 7.04. **Found:** N, 4.83; C, 58.05; H, 6.76.

Note: KC_8 is pyrophoric. Extreme caution must be exercised during its handling.

2.12 W_2Cl_2[μ-η²:η²-1,4-$Ph_2C_4H_2$][μ-κ²-HC(N-2,6-^{i}Pr$_2C_6H_3$)$_2$]$_2$ (12)

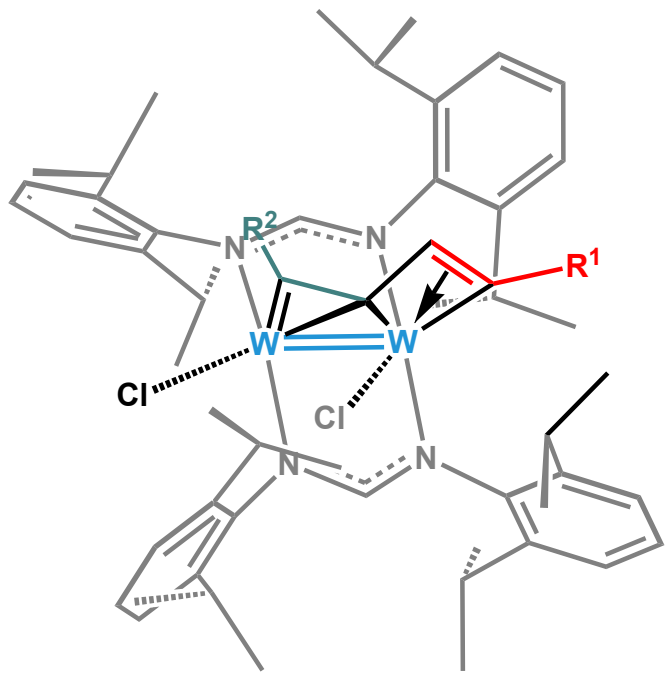

12: $R^1 = R^2 = C_6H_5$

A 20 mL vial was charged with **2** (0.0413 g, 0.0322 mmol). Et_2O (6 mL) and Phenylacetylene (0.65 mL of a 0.1 M solution in toluene, 0.0643 mmol) were added sequentially and the mixture was stirred at room temperature for 2 h, during which the orange suspension turned brown. All volatiles were then removed under reduced pressure. *n*-Hexane (2 mL) was added and the brown suspension was transferred onto a pad of *Celite*; The residue was washed with *n*-hexane until the filtrate was colorless. The residue was extracted with THF and the insoluble materials were removed by filtration. The combined dark olive-green filtrate was concentrated under vacuum to give dark olive-green powder **12**·(0.0256 g, 0.0187 mmol, 58.1%). Single crystals suitable for X-ray diffraction were obtained by slow evaporation of THF solution at room temperature.

^{1}H NMR (500 MHz, C_6D_6, 298 K): δ 8.55 (br. s, 4H, W_2(PhC(C*H*)(CH)=CPh) + NC*H*N + W_2(PhC(CH)(C*H*)=CPh), 7.04–6.80 (m, 22H, W_2(PhC(CH)(C*H*)=CPh-*Ar* + Dipp-*Ar*), 6.93 (dd, $^3J_{H–H}$ = 7.8 Hz; $^4J_{H–H}$ = 1.8 Hz, 2H, Dipp-Ar-*meta*), 4.43 (sep, $^3J_{H–H}$ = 6.5 Hz, 2H, Dipp-C*H*(CH_3)$_2$), 3.17 (sep, $^3J_{H–H}$ = 6.7 Hz, 2H, Dipp-CH(CH_3)$_2$), 3.09 (sep, $^3J_{H–H}$ = 6.8 Hz, 2H, Dipp-C*H*(CH_3)$_2$), 1.68 (d, $^3J_{H–H}$ = 6.5 Hz, 6H, Dipp-CH(CH_3)$_2$), 1.42 (d, $^3J_{H–H}$ = 6.5 Hz, 6H, Dipp-CH(CH_3)$_2$), 1.19 (d, $^3J_{H–H}$ = 8.2 Hz, 6H, Dipp-CH(CH_3)$_2$), 1.04 (d, $^3J_{H–H}$ = 6.9 Hz, 6H, Dipp-CH(CH_3)$_2$), 1.02 (d, $^3J_{H–H}$ = 7.8 Hz, 6H, Dipp-CH(CH_3)$_2$), 0.81 (d, $^3J_{H–H}$ = 7.2 Hz, 6H, Dipp-CH(CH_3)$_2$), 0.76 (d, $^3J_{H–H}$ = 6.7 Hz, 6H, Dipp-CH(CH_3)$_2$). **^{13}C{^{1}H} NMR (126 MHz, *d_8*-Toluene, 213 K):** δ 259.7 (s, 1C, W_2(Ph*C*(CH)(CH)=CPh)), 247.3 (s, 1C, W_2(PhC(CH)(CH)=*C*Ph)), 177.2 (s, 1C, N*C*HN), 150.3 (s, *Ar*), 147.4 (s, *Ar*), 146.7 (s, *Ar*), 145.9 (s, *Ar*), 144.5 (s, *Ar*), 144.0 (s, *Ar*), 143.3 (s, *Ar*), 141.8 (s, *Ar*), 141.3 (s, *Ar*), 140.8 (s, *Ar*), 133.5 (s, *Ar*), 123.2 (s, *Ar*), 123.0 (s, *Ar*), 122.4 (s, *Ar*), 119.0 (s, 1C, W_2(PhC(CH)(*C*H)=CPh)), 47.7 (s, 1C, W_2(PhC(*C*H)(CH)=CPh)), 36.1, 34.5, 29.3, 28.7, 27.8, 27.5, 26.6, 26.2, 24.5, 24.2, 23.9, 23.4, 23.1, 22.1, 14.6. **Anal. Calcd. for $C_{66}H_{82}Cl_2N_4W_2$:** N, 4.09; C, 57.86; H, 6.03. Found: N, 3.91; C, 57.70; H, 5.78.

Note: No uncommon hazards are noted.

2.13 W_2Cl_2[μ-η^2:η^2-1,4-(2-MePh)$_2C_4H_2$][μ-κ^2-HC(N-2,6-^{i}Pr$_2C_6H_3$)$_2$]$_2$ (13)

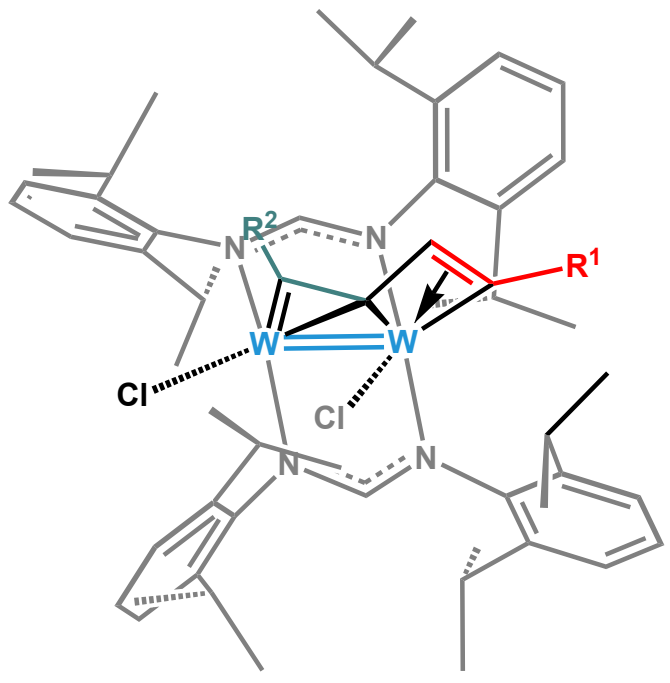

13: $R^1 = R^2 = 2\text{-}MeC_6H_4$

A 20 mL vial was charged with **2** (0.0419 g, 0.0326 mmol). Et_2O (6 mL) and 2-Methylphenylacetylene (0.57 mL of a 0.1 M solution in toluene, 0.0564 mmol) were added sequentially and the mixture was stirred at room temperature for 2 h, during which the orange suspension turned mint-green. All volatiles were then removed under reduced pressure. *n*-Hexane (2 mL) was added and the brown suspension was transferred onto a pad of *Celite*; The residue was washed with *n*-hexane until the filtrate was colorless. The residue was extracted with THF and the insoluble materials were removed by filtration. The combined chrome-green filtrate was concentrated under vacuum to give chrome-green powder **13**·(0.0200 g, 0.0143 mmol, 50.8%). Single crystals suitable for X-ray diffraction were obtained by recrystallization from THF at −35 °C.

^{1}H NMR (500 MHz, d_8-Toluene, 353 K): δ 8.74 (br. s, 2H, W_2(2-MePhC(C*H*)(CH)=C(2-MePh)) + W_2(2-MePhC(C*H*)(CH)=C(2-MePh))), 8.62 (s, 2H, NC*H*N), 7.08 (d, $^3J_{H–H}$ = 7.6 Hz, 2H, *Ar*), 7.01–6.96 (m, 4H, *Ar*), 6.90 (t, $^3J_{H–H}$ = 7.6 Hz, 2H, *Ar*), 6.80 (t, $^3J_{H–H}$ = 7.7 Hz, 2H, *Ar*), 6.76 (d, $^3J_{H–H}$ = 6.3 Hz, 4H, *Ar*), 6.72 (d, $^3J_{H–H}$ = 7.4 Hz, 2H, *Ar*), 6.65 (d, $^3J_{H–H}$ = 7.6 Hz, 2H, *Ar*), 4.27 (sep, $^3J_{H–H}$ = 6.7 Hz, 4H, Dipp-C*H*($CH_3)_2$), 3.00 (sep, $^3J_{H–H}$ = 6.7 Hz, 4H, Dipp-C*H*($CH_3)_2$), 1.62 (d, $^3J_{H–H}$ = 6.5 Hz, 6H, Dipp-CH(C$H_3)_2$), 1.55 (d, $^3J_{H–H}$ = 6.7 Hz, 6H, Dipp-CH(C$H_3)_2$), 1.26 (d, $^3J_{H–H}$ = 6.7 Hz, 6H, Dipp-CH(C$H_3)_2$), 1.12 (d, $^3J_{H–H}$ = 6.9 Hz, 6H, Dipp-CH(C$H_3)_2$), 0.78 (d, $^3J_{H–H}$ = 6.7 Hz, 6H, Dipp-CH(C$H_3)_2$), 0.77 (d, $^3J_{H–H}$ = 6.9 Hz, 6H, Dipp-CH(C$H_3)_2$), 0.71 (d, $^3J_{H–H}$ = 6.7 Hz, 6H, Dipp-CH(C$H_3)_2$), 0.68 (d, $^3J_{H–H}$ = 6.5 Hz, 6H, Dipp-CH(C$H_3)_2$). **^{13}C{^{1}H} NMR (126 MHz, d_8-Toluene, 353 K):** δ 252.1 (s, 1C, W_2(2-MePh*C*(CH)(CH)=C(2-MePh))), 176.3 (s, 2C, N*C*HN), 147.4 (s, *Ar*), 145.8 (s, *Ar*), 144.7 (s, *Ar*), 141.5 (s, *Ar*), 130.8 (s, *Ar*), 127.1 (s, *Ar*), 125.8 (s, *Ar*), 124.3 (s, *Ar*), 123.7 (s, *Ar*), 30.9, 29.8, 28.9, 28.5, 28.3, 28.2, 28.0, 25.6, 23.8, 22.4, 22.3. **Anal. Calcd. for $C_{68}H_{86}Cl_2N_4W_2$:** N, 4.01; C, 58.42; H, 6.20. **Found:** N, 3.59; C, 58.62; H, 6.07.

Note: No uncommon hazards are noted.

2.14 $W_2Cl_2[\mu\text{-}\eta^2:\eta^2\text{-}1,4\text{-}(4\text{-}OMePh)_2C_4H_2][\mu\text{-}\kappa^2\text{-}HC(N\text{-}2,6\text{-}{}^iPr_2C_6H_3)_2]_2$ (14)

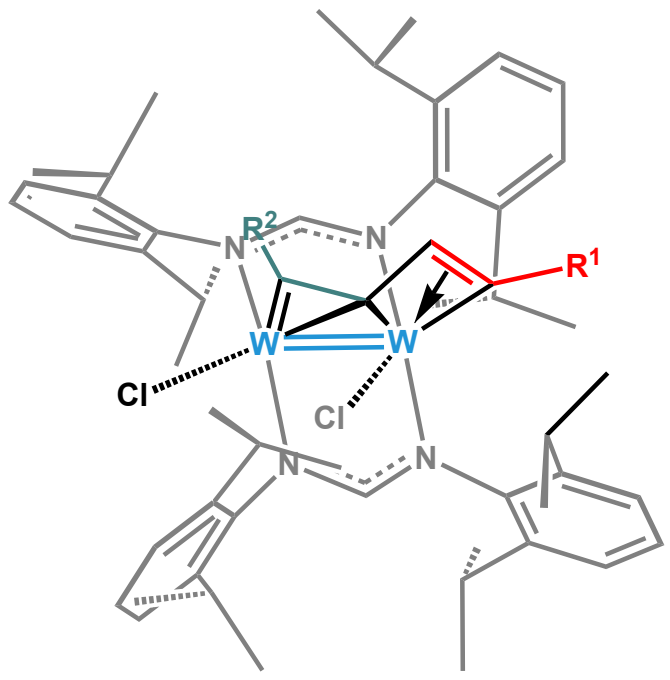

14: $R^1 = R^2 = 4\text{-}MeOC_6H_4$

A 20 mL vial was charged with **2** (0.0433 g, 0.0282 mmol). Et_2O (6 mL) and 4-methoxyphenylacetylene (0.59 mL of a 0.10 M solution in toluene, 0.0590 mmol) were added sequentially and the mixture was stirred at room temperature for 12 h, during which the suspension turned chartreuse. The mixture was passed through a pad of *Celite* and the residue was washed with Et_2O until the filtrate was light green. The residue was extracted with THF and the insoluble materials were removed by filtration. The combined filtrate was concentrated under vacuum to give brown powder **14**·(0.0291 g, 0.0203 mmol, 72.3%).·Single crystals suitable for X-ray diffraction were obtained by diffusion of *n*-hexane into THF solution at room temperature.

^{1}H NMR (d_8-THF, 500 MHz, 298K): δ 8.36 (s, 2H, NC*H*N), 8.25 (br, 2H, C(C*H*)(C*H*)C), 6.70−7.20 (m, 20H, *Ar*), 4.22 (sept, 2H, C*H*(CH_3)$_2$), 3.79 (s, 6H, OC*H*$_3$), 2.99 (sept, 2H, C*H*(CH_3)$_2$), 2.93 (sept, 2H, C*H*(CH_3)$_2$), 1.46(d, 6H, C*H*(CH_3)$_2$), 1.22(d, 6H, CH(C*H*$_3$)$_2$), 1.20 (d, 6H, CH(C*H*$_3$)$_2$), 1.09 (d, 6H, CH(C*H*$_3$)$_2$), 1.01(d, 6H, CH(C*H*$_3$)$_2$), 0.88 (d, 6H, CH(C*H*$_3$)$_2$), 0.78 (d, 6H, CH(C*H*$_3$)$_2$), 0.68 (d, 6H, CH(C*H*$_3$)$_2$). **^{13}C NMR (d_8-THF, 126 MHz, 298K):** δ 177.5 (s, 2C, N*C*N), 160.9 (s, *Ar*), 146.60 (s, *Ar*), 146.4 (s, *Ar*), 145.6 (s, *Ar*), 143.8 (s, *Ar*), 135.9 (s, *Ar*), 132.9 (s, *Ar*), 126.5 (s, *Ar*), 126.3 (s, *Ar*), 124.8 (s, *Ar*), 124.3 (s, *Ar*), 123.6 (s, *Ar*), 113.9 (s, *Ar*), 55.4 (s, 2C, O*C*H$_3$), 28.7 (s, 2C, *C*H(CH_3)$_2$), 28.4 (s, 2C, *C*H(CH_3)$_2$), 28.1 (s, 2C, *C*H(CH_3)$_2$), 27.9 (s, 2C, CH(*C*H$_3$)$_2$), 27.6 (s, 2C, CH(*C*H$_3$)$_2$), 26.4 (s, 6C, CH(*C*H$_3$)$_2$), 24.2 (s, 6C, CH(*C*H$_3$)$_2$), 23.2 (s, 6C, CH(*C*H$_3$)$_2$), 23.0 (s, 6C, CH(*C*H$_3$)$_2$). **Anal. Calcd. For $C_{68}H_{86}Cl_2N_4O_2W_2$:** N, 3.92; C, 57.11; H, 6.06. **Found:** N, 3.80; C, 57.31; H, 5.84.

Note: No uncommon hazards are noted.

2.15 W_2Cl_2[μ-η^2:η^2-1,4-(*n*-C_3H_7)$_2C_4H_2$][μ-κ^2-HC(N-2,6-^{i}Pr$_2C_6H_3$)$_2$]$_2$ (15)

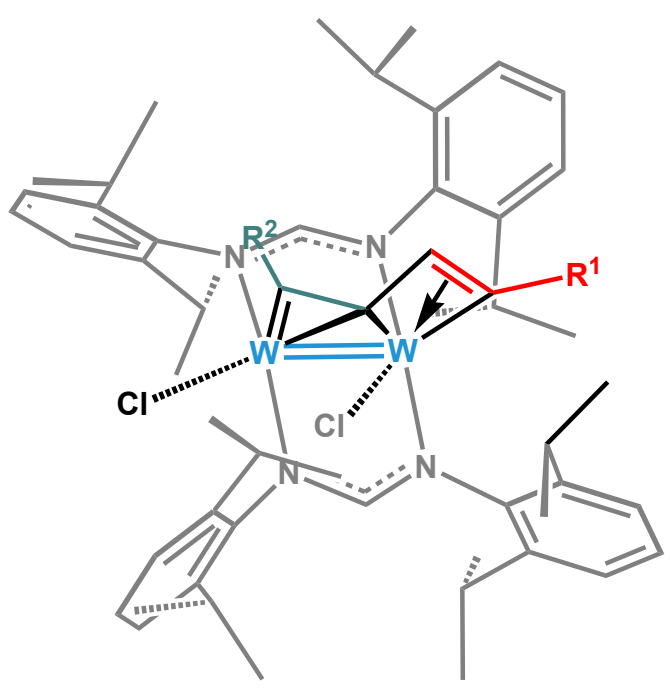

15: $R^1 = R^2 =$ *n*-C_3H_7

A 20 mL vial was charged with **2** (0.0439 g, 0.0343 mmol). Et_2O (6 mL) and 1-pentyne (0.65 mL of a 0.1 M solution in toluene, 0.0663 mmol) were added sequentially and the mixture was stirred at room temperature for 1.5 h, during which the orange suspension turned brown. All volatiles were then removed under reduced pressure. *n*-Hexane (2 mL) was added and the brown suspension was transferred onto a pad of *Celite*; The residue was extracted with *n*-hexane until the

filtrate was colorless and the insoluble materials were removed by filtration. The combined brown filtrate was concentrated under vacuum to give brown powder **15**·(0.0320 g, 0.0246 mmol, 74.1%). Single crystals suitable for X-ray diffraction were obtained by slow evaporation of THF solution at room temperature.

^{1}H NMR (500 MHz, C_6D_6, 298 K): δ 9.00 (d, $^{3}J_{H–H}$ = 2.3 Hz, 1H, W_2(^{n}PrC(C*H*)(CH)=C^{n}Pr)), 8.63 (s, 1H, NC*H*N), 8.08 (s, 1H, NC*H*N), 7.43 (d, $^{3}J_{H–H}$ = 7.3 Hz, 1H, W_2(^{n}PrC(CH)(C*H*)=C^{n}Pr)), 7.24 (d, $^{3}J_{H–H}$ = 7.3 Hz, 1H, Dipp-Ar-*meta*), 7.12 (t, $^{3}J_{H–H}$ = 7.2 Hz, 1H, Dipp-Ar-*para*), 7.07–6.85 (m, 10H, Dipp-*Ar*), 4.65 (sep, $^{3}J_{H–H}$ = 7.2 Hz, 1H, Dipp-CH(CH_3)$_2$), 4.18 (sep, $^{3}J_{H–H}$ = 6.9 Hz, 1H, Dipp-CH(CH_3)$_2$), 3.43 (sep, $^{3}J_{H–H}$ = 7.3 Hz, 1H, Dipp-C*H*(CH_3)$_2$), 3.36 (sep, $^{3}J_{H–H}$ = 6.7 Hz, 2H, Dipp-C*H*(CH_3)$_2$), 3.26 (sep, $^{3}J_{H–H}$ = 7.0 Hz, 1H, Dipp-C*H*(CH_3)$_2$), 2.84 (sep, $^{3}J_{H–H}$ = 6.7 Hz, 1H, Dipp-C*H*(CH_3)$_2$), 2.28–2.20 (m, 1H, ^{n}PrC(CH)(CH)=C(CH_2)(CH_αH$_\beta$)Et), 2.18–2.06 (m, 2H, Me(CH_2)(CH_2)C(CH)(CH)=C^{n}Pr), 2.01–1.91 (m, 1H, ^{n}PrC(CH)(CH)=C(CH$_\alpha$$H_\beta$)Et), 1.78 (d, $^{3}J_{H–H}$ = 6.6 Hz, 3H, Dipp-CH(CH_3)$_2$), 1.64 (d, $^{3}J_{H–H}$ = 6.5 Hz, 3H, Dipp-CH(CH_3)$_2$), 1.45 (d, $^{3}J_{H–H}$ = 6.8 Hz, 3H, Dipp-CH(CH_3)$_2$), 1.36 (d, $^{3}J_{H–H}$ = 6.4 Hz, 9H, Dipp-CH(CH_3)$_2$), 1.29–1.21 (m, 21H, *Me*(CH_2)(CH_2)C(CH)(CH)=C^{n}Pr + Dipp-CH(CH_3)2), 1.18 (d, $^{3}J_{H–H}$ = 7.7 Hz, 3H, Dipp-CH(CH_3)$_2$), 1.11 (d, $^{3}J_{H–H}$ = 7.1 Hz, 3H, Dipp-CH(CH_3)$_2$), 0.90 (d, $^{3}J_{H–H}$ = 6.0 Hz, 3H, Dipp-CH(CH_3)$_2$), 0.81 (d, $^{3}J_{H–H}$ = 6.8 Hz, 3H, Dipp-CH(CH_3)$_2$), 0.75–0.65 (m, 1H, ^{n}PrC(CH)(CH)=C(CH_2)(CH_αH$_\beta$)Me), 0.44 (t, $^{3}J_{H–H}$ = 7.2 Hz, 3H, ^{n}PrC(CH)(CH)=C(CH_2)(CH_2)*Me*), 0.39–0.29 (m, 1H, ^{n}PrC(CH)(CH)=C(CH_2)(CH$_\alpha$$H_\beta$)Me). **^{13}C{^{1}H} NMR (126 MHz, C_6D_6, 298 K):** δ 279.0 (s, 1C, W_2(^{n}Pr*C*(CH)(CH)=C^{n}Pr)), 249.7 (s, 1C, W_2(^{n}PrC(CH)(CH)=*C*^{n}Pr)), 177.6 (s, 1C, N*C*HN), 175.9 (s, 1C, N*C*HN), 148.6 (s, 1C, Dipp-Ar-*C*), 147.0 (s, 1C, Dipp-Ar-*C*), 146.1 (s, 1C, Dipp-Ar-*C*), 145.9 (s, 1C, Dipp-Ar-*C*), 145.3 (s, 1C, Dipp-Ar-*C*), 145.3 (s, 1C, Dipp-Ar-*C*), 144.5 (s, 1C, Dipp-Ar-*C*), 144.0 (s, 1C, Dipp-Ar-*C*), 143.9 (s, 1C, Dipp-Ar-*C*), 143.7 (s, 1C, Dipp-Ar-*C*H), 126.8 (s, 1C, Dipp-Ar-*C*H), 126.7 (s, 1C, Dipp-Ar-*C*H), 126.6 (s, 1C, Dipp-Ar-*C*H), 126.5 (s, 1C, Dipp-Ar-*C*H), 125.6 (s, 1C, Dipp-Ar-*C*H), 125.0 (s, 1C, Dipp-Ar-*C*H), 125.0 (s, 1C, Dipp-Ar-*para*), 124.9 (s, 1C, Dipp-Ar-*meta*), 124.0 (s, 1C, Dipp-Ar-*C*H), 123.8 (s, 1C, Dipp-Ar-*C*H), 122.2 (s, 1C, W_2(^{n}PrC(CH)(*C*H)=C^{n}Pr)), 45.6 (s, 2C, Et(*C*H_2)C(CH)(CH)=C(CH_2)Et), 44.9 (s, 1C, W_2(^{n}PrC(*C*H)(CH)=C^{n}Pr)), 33.7 (s, 1C, Dipp-*C*H(CH_3)$_2$), 29.2 (s, 1C, Dipp-*C*H(CH_3)$_2$), 28.7 (s, 1C, Dipp-*C*H(CH_3)$_2$), 28.3 (s, 1C, Dipp-*C*H(CH_3)$_2$), 28.1 (s, 2C, Dipp-*C*H(CH_3)$_2$ + Dipp-CH(*C*H_3)$_2$), (s, 2C, Dipp-*C*H(CH_3)$_2$), 28.0 (s, 1C, Dipp-*C*H(CH_3)$_2$), 27.9 (s, 1C, Dipp-*C*H(CH_3)2), 27.4 (s, 1C, Dipp-CH(*C*H_3)$_2$), 27.3 (s, 1C, Dipp-CH(*C*H_3)$_2$), 27.2 (s, 1C, *Me*(CH_2)(CH_2)C(CH)(CH)=C^{n}Pr), 26.6 (s, 1C, Dipp-CH(*C*H_3)$_2$), 26.5 (s, 1C, Dipp-CH(*C*H_3)$_2$), 26.4 (s,

1C, Dipp-CH(CH_3)$_2$), 26.3 (s, 1C, Dipp-CH(CH_3)$_2$), 25.7 (s, 1C, Dipp-CH(CH_3)$_2$), 25.5 (s, 1C, Dipp-CH(CH_3)$_2$), 25.4 (s, 1C, Dipp-CH(CH_3)$_2$), 25.3 (s, 1C, Dipp-CH(CH_3)$_2$), 25.2 (s, 1C, Dipp-CH(CH_3)$_2$), 25.0 (s, 1C, Dipp-CH(CH_3)$_2$), 24.0 (s, 1C, Dipp-CH(CH_3)$_2$), 22.1 (s, 1C, Dipp-CH(CH_3)$_2$), 20.9 (s, 1C, ^{n}PrC(CH)(CH)=C(CH_2)(CH_2)Me), 14.9 (s, 1C, *Me*(CH_2)(CH_2)C(CH)(CH)=C^{n}Pr) , 14.1 (s, 1C, ^{n}PrC(CH)(CH)=C(CH_2)(CH_2)*Me*). **Anal. Calcd. For $C_{60}H_{86}Cl_2N_4W_2$:** N, 4.30; C, 55.35; H, 6.66. **Found:** N, 4.10; C, 55.12; H, 6.44.

Note: No uncommon hazards are noted.

2.16 W_2Cl_2[μ-η^2:η^2-1-2-MeC_6H_4-4-*n*-$C_3H_7C_4H_2$][μ-κ^2-HC(N-2,6-^{i}Pr$_2C_6H_3$)$_2$]$_2$ (16)

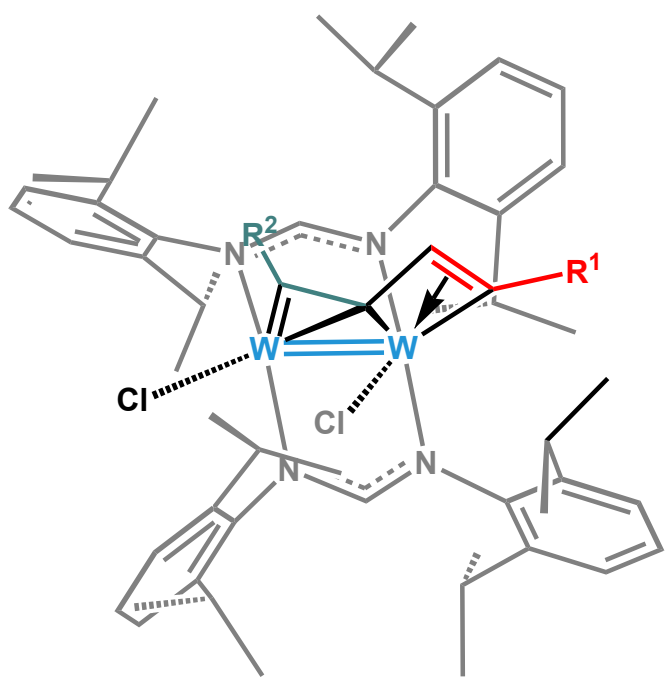

16: R^1 = 2-MeC_6H_4, R^2 = *n*-C_3H_7

A 20 mL vial was charged with **17** (0.0242 g, 0.0189 mmol). Et_2O (5 mL) and 1-pentyne (0.21 mL of a 0.1 M solution in toluene, 0.0210 mmol) were added sequentially, which the green suspension immediately turned red brown. The mixture was stirred at room temperature for 17 h. All volatiles were then removed under reduced pressure. *n*-Hexane (2 mL) was added and the red brown suspension was transferred onto a pad of *Celite*; The residue was extracted with *n*-hexane/toluene mixture (1:1) until the filtrate was colorless and the insoluble materials were removed by filtration. The combined brown filtrate was concentrated under vacuum to give brown powder **16**·(0.0219 g, 0.0162 mmol, 85.9%). Single crystals suitable for X-ray diffraction were obtained by slow evaporation of THF solution at room temperature. **^{1}H NMR (500 MHz, C_6D_6, 298 K):** δ 9.06 (br, 1H, 2-(CH_3)PhCC*H*CHC^{n}Pr), 8.71 (s, 1H, NC*H*N), 8.17 (s, 1H, NC*H*N), 7.61 (br, 1H, 2-(CH_3)PhCCHC*H*C^{n}Pr), 7.25 (d, 1H, Ar-*meta*, $^3J_{H–H}$ = 7.6 Hz), 7.12 (d, 2H, Ar-*meta*, $^3J_{H–H}$ = 7.8 Hz), 7.07-7.00 (m, 4H, Ar-C*H*), 6.92 (t, 1H, Ar-*para*, $^3J_{H–H}$ = 7.7 Hz), 6.74 (d, 1H, Ar-*meta*, $^3J_{H–H}$ = 7.7 Hz), 6.70-6.67 (m, 1H, Ar-C*H*), 6.65-6.56 (m, 4H, Ar-C*H*), 6.47 (d, 1H, Ar-*meta*, $^3J_{H–H}$ = 7.6 Hz), 6.09 (br, 1H, Ar-C*H*), 4.67-4.61(m, 2H, C*H*(CH_3)$_2$), 4.37 (br, 1H, 2-(CH_3)PhCCHCHCC$H_2$$CH_2CH_3$), 4.29-4.24 (m, 2H, C*H*Me$_2$ and 2-(CH_3)PhCCHCHCC$H_2$$CH_2CH_3$), 3.47-3.38 (m, 2H, C*H*(CH_3)$_2$), 2.95 (quint, 1H, C*H*(CH_3)$_2$, $^3J_{H–H}$ = 6.7 Hz) , 2.31-2.24 (m, 1H, 2-(CH_3)PhCCHCHCCH_2C$H_2$$CH_3$), 2.20-2.15 (m, 1H, 2-($CH_3$)PhCCHCHCC$H_2CH_2$$CH_3$), 1.95 (s, 3H, CH(C$H_3$)$_2$, $^3J_{H–H}$ = 6.5 Hz), 1.62 (d, 3H, CH(CH_3)$_2$, $^3J_{H–H}$ = 6.5 Hz), 1.42 (d, 3H, CH(CH_3)$_2$, $^3J_{H–H}$ = 6.9 Hz), 1.40 (d, 3H, CH(CH_3)$_2$, $^3J_{H-H}$ = 6.6 Hz), 1.33 (d, 3H, CH(CH_3)$_2$, $^3J_{H-H}$ = 6.4 Hz), 1.32 (d, 3H, CH(CH_3)$_2$, $^3J_{H–H}$ = 6.8 Hz), 1.30 (d, 3H, CH(CH_3)$_2$, $^3J_{H–H}$ = 6.7

Hz), 1.29 (d, 3H, CH(C*H*$_3$)$_2$, $^3J_{H–H}$ = 6.8 Hz), 1.24 (t, 9H, CH(C*H*$_3$)$_2$ and 2-(CH$_3$)PhCCHCHCCH$_2$CH$_2$C*H*$_3$, $^3J_{H–H}$ = 6.9 Hz), 1.12 (d, 3H, CH(C*H*$_3$)$_2$, $^3J_{H–H}$ = 6.8 Hz), 0.81(d, 3H, CH(C*H*$_3$)$_2$, $^3J_{H–H}$ = 6.9 Hz), 0.74 (d, 3H, CH(C*H*$_3$)$_2$, $^3J_{H–H}$ = 6.7 Hz), 0.66 (d, 3H, CH(C*H*$_3$)$_2$, $^3J_{H–H}$ = 6.8 Hz). **^{13}C NMR (126 MHz, C$_6$D$_6$, 298 K):** δ 260.5 (s, 1C, 2-(CH$_3$)PhCCHCH*C*^{n}Pr), 250.6 (s, 1C, PhC*C*HCHC^{n}Pr), 177.6 (s, 1C, N*C*HN), 175.9 (s, 1C, N*C*HN), 148.3 (s, 1C, Ar-*C*), 147.0 (s, 1C, Ar-*C*), 146.8(s, 2C, Ar-*C*), 146.0 (s, 1C, Ar-*C*), 145.9(s, 1C, Ar-*C*), 145.2 (s, 1C, Ar-*C*), 144.1 (s, 1C, Ar-*C*), 144.0 (s, 1C, Ar-*C*), 143.8 (s, 1C, Ar-*C*), 143.5 (s, 1C, Ar-*C*), 142.2 (s, 1C, Ar-*C*), 140.2 (s, 1C, Ar-*C*), 135.1 (s, 1C, Ar-*C*), 132.8 (s, 1C, Ar-*C*H), 130.3 (s, 1C, Ar-*C*H), 129.3 (s, 1C, 2-(CH$_3$)Ph*C*CHCHC^{n}Pr), 129.2 (s, 1C, Ar-*C*H), 128.5 (s, 1C, 2-(CH$_3$)PhCCH*C*HC^{n}Pr), 127.0 (s, 1C, Ar-*para*),126.7 (s, 1C, Ar-*C*H), 126.5 (s, 1C, Ar-*C*H), 126.1 (s, 1C, Ar-*C*H), 125.8 (s, 1C, Ar-*C*H), 124.9 (s, 1C, Ar-*meta*), 124.8 (s, 1C, Ar-*meta*), 124.7 (s, 2C, Ar-*C*H), 124.7 (s, 1C, Ar-*meta*), 123.8 (s, 1C, Ar-*C*H), 123.6 (s, 1C, Ar-*C*H), 122.9 (s, 1C, Ar-*meta*), 46.0 (s, 1C, 2-(CH$_3$)PhCCHCHC*C*H$_2$CH$_2$CH$_3$), 33.9 (s, 1C, *C*H(CH$_3$)$_2$), 29.1 (s, 1C, CH(*C*H$_3$)$_2$), 29.1 (s, 1C, *C*H(CH$_3$)$_2$), 28.3 (s, 1C, *C*H(CH$_3$)$_2$), 28.1 (s, 1C, *C*H(CH$_3$)$_2$), 27.9 (s, 1C, CH(*C*H$_3$)$_2$), 27.9 (s, 1C, *C*H(CH$_3$)$_2$), 27.8 (s, 1C, *C*H(CH$_3$)$_2$), 27.5 (s, 1C, *C*H(CH$_3$)$_2$), 27.5 (s, 1C, CH(*C*H$_3$)$_2$), 27.3 (s, 1C, 2-(CH$_3$)PhCCHCHCCH$_2$*C*H$_2$CH$_3$), 27.0 (s, 1C, CH(*C*H$_3$)$_2$), 26.9 (s, 1C, CH(*C*H$_3$)$_2$), 26.5 (s, 1C, CH(*C*H$_3$)$_2$), 26.2 (s, 1C, CH(*C*H$_3$)$_2$), 25.8 (s, 1C, CH(*C*H$_3$)$_2$), 25.6 (s, 1C, CH(*C*H$_3$)$_2$), 25.6 (s, 1C, CH(*C*H$_3$)$_2$), 25.3 (s, 1C, CH(*C*H$_3$)$_2$), 25.3 (s, 1C, CH(*C*H$_3$)$_2$), 25.1 (s, 1C, CH(*C*H$_3$)$_2$), 24.5 (s, 1C, CH(*C*H$_3$)$_2$), 22.2 (s, 1C, CH(*C*H$_3$)$_2$), 22.1 (s, 1C, 2-(*C*H$_3$)PhCCHCHC^{n}Pr), 20.9 (s, 1C, CH(*C*H$_3$)$_2$), 14.8 (s, 1C, 2-(CH$_3$)PhCCHCHCCH$_2$CH$_2$*C*H$_3$). **Anal. Calcd. For C$_{64}$H$_{86}$Cl$_2$N$_4$W$_2$:** N, 4.15; C, 56.94; H, 6.42. **Found:** N, 3.91; C, 56.66; H, 6.29.

Note: No uncommon hazards are noted.

2.17 W$_2$Cl$_2$[μ-η^2-2-MeC$_6$H$_4$CCH][μ-κ^2-HC(N-2,6-^{i}Pr$_2$C$_6$H$_3$)$_2$]$_2$ (17)

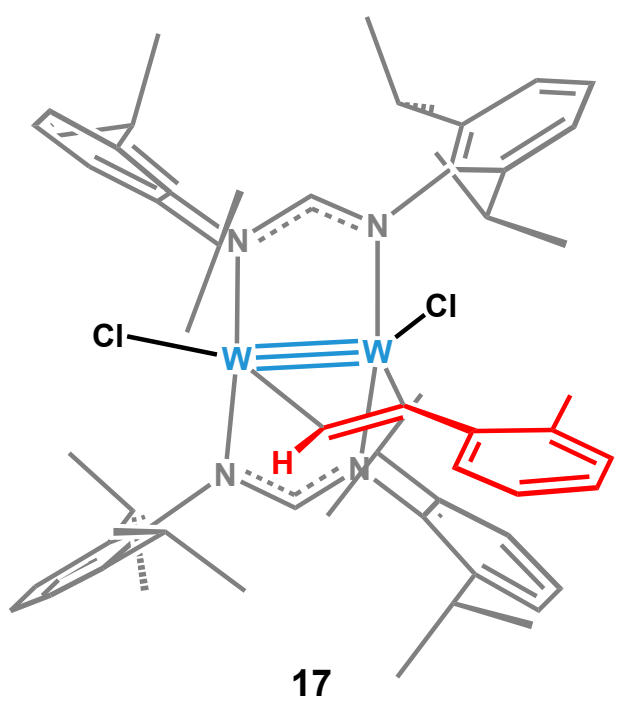

17

A 20 mL vial was charged with **2** (0.1156 g, 0.0903 mmol) and pre-cooled using a liquid nitrogen-chilled Coldwell for 1 h. Once the mixture reached a solid-liquid equilibrium, Et$_2$O (12 mL) and 2-methylphenylacetylene (0.86 mL of a 0.1 M solution in toluene, 0.0860 mmol) were added sequentially and the mixture was stirred for 0.5 h, while gradually warming to room temperature, during which the orange suspension slowly turned dark green. All volatiles were then removed under reduced pressure. *n*-Hexane (2 mL) was

added and the brown suspension was transferred onto a pad of *Celite*; The residue was washed with *n*-Hexane and Et2O sequentially until the filtrate was colorless. The remaining residue was extracted with fluorobenzene until the filtrate was colorless and the insoluble materials were removed by filtration. The combined green filtrate was concentrated under vacuum to give blue-green powder **17**·(0.0295 g, 0.0230 mmol, 25.5%). Single crystals suitable for X-ray diffraction were obtained by diffusion of *n*-hexane into toluene solution at −35 °C.

^{1}H NMR (500 MHz, C_6D_6, 298 K): δ 16.02(s, 1H, C*H*-2-$CH_3C_6H_4$), 9.18 (s, 2H, NC*H*N), 7.75 (d, 1H, Ar-C*H*, $^3J_{H–H}$ = 7.6 Hz), 7.32-7.26 (m, 3H, Ar-C*H*), 7.05 (t, 5H, Ar-C*H*, $^3J_{H–H}$ = 7.6 Hz), 6.98-6.94 (m, 4H, Ar-C*H*), 6.88 (d, 2H, Ar-C*H*, $^3J_{H–H}$ = 7.8 Hz), 6.79 (d, 1H, Ar-C*H*, $^3J_{H–H}$ = 7.6 Hz), 4.03 (quint, 3H, C*H*(CH_3)$_2$, $^3J_{H–H}$ = 6.5 Hz) ,3.83-3.77 (m, 5H, C*H*(CH_3)$_2$), 1.70 (d, 6H, CH(CH_3)$_2$, $^3J_{H–H}$ = 6.4 Hz), 1.60 (d, 6H, CH(CH_3)$_2$, $^3J_{H–H}$ = 6.2 Hz), 1.25 (d, 9H, CH(CH_3)$_2$, $^3J_{H–H}$ = 6.9 Hz), 1.18-1.15 (m, 9H, CH(CH_3)$_2$), 1.02(s, 3H, CH-2-C$H_3$$C_6H_4$), 0.77 (d, 6H, CH(C$H_3$)$_2$, $^3J_{H–H}$ = 6.6 Hz), 0.52 (d, 6H, CH(CH_3)$_2$, $^3J_{H–H}$ = 6.6Hz). **^{13}C NMR (126 MHz, C_6D_6, 298 K):** δ 217.4(s, 1C, *C*H-2-$CH_3C_6H_4$), 148.3, 145.1, 144.6, 144.6, 143.2, 141.1, 132.4, 131.4, 129.3, 127.6, 127.5, 126.2, 126.0, 125.7(s, 1C, *C*H-2-$CH_3C_6H_4$), 124.8, 123.5, 122.6, 30.2, 29.5, 29.3, 29.0, 28.6, 28.5, 28.0, 25.9, 25.2, 24.7, 23.9. **Anal. Calcd. For $C_{59}H_{78}Cl_2N_4W_2$:** N, 4.37; C, 55.28; H,6.13. **Found:** N, 3.69; C, 55.38; H, 6.26.

Note: Extreme caution must be exercised during the handling liquid nitrogen.

2.18 W_2[μ-κ^2-1-2-MeC_6H_4-4-*n*-$C_3H_7C_4H_2$][μ-κ^2-HC(N-2,6-^{i}Pr$_2C_6H_3$)$_2$]$_2$ (18)

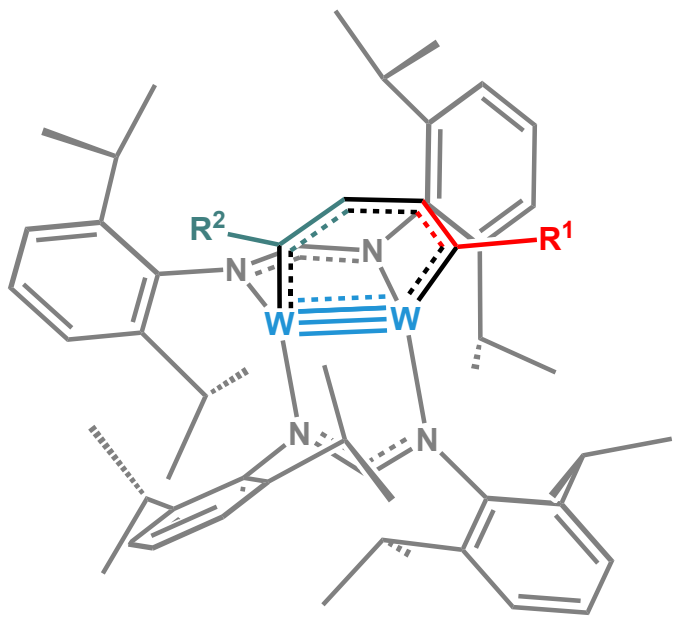

18: R^1 = 2-MeC_6H_4, R^2 = *n*-C_3H_7

A 20 mL vial was charged with **16** (0.0335 g, 0.0248 mmol). Et_2O (13 mL) and KC_8 (0.0122 g, 0.0903 mmol) were added sequentially. The mixture was stirred at room temperature for 17 h, during which the initial brown suspension turned dichroic reddish-green. All volatiles were then removed under reduced pressure. *n*-Hexane was added and the brown suspension was transferred onto a pad of *Celite*; The residue was extracted with *n*-hexane until the filtrate was green then finally colorless. The insoluble materials were removed by filtration. The combined filtrate was concentrated under vacuum to give green-brown powder **18**·(0.0261 g, 0.0204 mmol, 82.2%). Single crystals suitable for X-ray diffraction were obtained by slow evaporation of *n*-hexane solution at room temperature.

^{1}H NMR (500 MHz, C_6D_6, 298 K): δ 8.38 (s, 2H, NC*H*N), 8.22 (d, 1H, 2-(CH_3)PhCC*H*CHC^{n}Pr, $^4J_{H–H}$

= 8.7 Hz), 8.03 (d, 1H, 2-(CH_3)PhCCHC*H*C^{n}Pr, $^4J_{H\text{–}H}$ = 8.8 Hz), 7.36 (dd, 1H, 2-(CH_3)Ph-C*H*(6), $^3J_{H\text{–}H}$ = 7.7 Hz, $^4J_{H\text{–}H}$ = 1.4 Hz), 7.08-6.95 (m, 12H, dipp-Ar-C*H)*, 6.85 (ddd, 1H, 2-(CH_3)Ph-C*H*(5), $^3J_{H\text{–}H}$ = 7.4 and 7.3 Hz, $^4J_{H\text{–}H}$ = 1.4 Hz,), 6.45 (ddd, 1H, 2-(CH_3)Ph-C*H*(4), $^3J_{H\text{–}H}$ = 7.5 and 7.4 Hz, $^4J_{H\text{–}H}$ = 1.5 Hz,), 5.39 (dd, 1H, 2-(CH_3)Ph-C*H*(3), $^3J_{H\text{–}H}$ = 7.9 Hz, $^4J_{H\text{–}H}$ = 1.4 Hz,), 3.99 (br, 2H, C*H*(CH_3)$_2$), 3.87(br, 2H, C*H*(CH_3)$_2$), 3.51(br, 1H, C*H*(CH_3)$_2$),2.67(s, 1H, 2-(C*H*$_3$)PhCCHCHC^{n}Pr), 2.77 (br, 1H, C*H*(CH_3)$_2$), 2.59 (br, 4H, C*H*(CH_3)$_2$ and 2-(CH_3)PhCCHCHCC*H*$_2$$CH_2CH_3$), 1.36 (br, 2H, 2-($CH_3$)PhCCHCHCC$H_2$C*H*$_2$$CH_3$), 1.29 (t, 21H, CH(C*H*$_3$)$_2$, $^3J_{H\text{–}H}$ = 6.0 and 6.3 Hz), 1.15 (d, 6H, CH(C*H*$_3$)$_2$, $^3J_{H\text{–}H}$ = 6.6 Hz), 1.15 (d, 6H, CH(C*H*$_3$)$_2$, $^3J_{H\text{–}H}$ = 6.6 Hz), 0.94 (d, 15H, CH(C*H*$_3$)$_2$, $^3J_{H\text{–}H}$ = 6.9 Hz), 0.74 (t, 3H, 2-(CH_3)PhCCHCHCCH_2CH_2C*H*$_3$), 0.64(br,6H, CH(C*H*$_3$)$_2$). **^{13}C NMR (126 MHz, C_6D_6, 298 K):** δ 235.1(1C, 2-(CH_3)Ph*C*CHCHC^{n}Pr), 229.3 (1C, 2-(CH_3)PhCCHCH*C*^{n}Pr), 170.6 (2C, NCN), 147.7(2C,Ar-*C*), 145.9(2C,Ar-*C*), 145.3(1C,Ar-*C*), 144.0 (2C,Ar-*C*), 143.3 (1C,Ar-*C*),142.7(2C,Ar-*C*), 142.0(1C,Ar-*C*), 139.8 (1C, 2-(CH_3)PhC*C*HCHC^{n}Pr), 134.2 (1C, 2-(CH_3)PhCCH*C*HC^{n}Pr), 133.9 (2C,Ar-*C*), 133.3 (1C, 2-(CH_3)Ph-*C*H(6)), 130.9(1C, 2-(CH_3)Ph-*C*H(3)), 126.9(3C, dipp-Ar-*C*H), 126.6(1C, dipp-Ar-*C*H), 125.2(1C, 2-(CH_3)Ph-*C*H(5)),124.7(3C, dipp-Ar-*C*H), 123.9(1C, dipp-Ar-*C*H), 123.8(1C, 2-(CH_3)Ph-*C*H(4)), 123.4(3C, dipp-Ar-*C*H),123.1(1C, dipp-Ar-*C*H), 34.28(1C, 2-(CH_3)PhCCHCHC*C*$H_2$$CH_2CH_3$), 31.0 (2C, *C*H($CH_3$)$_2$), 30.8 (2C, *C*H($CH_3$)$_2$), 30.2 (2C, 2-($CH_3$)PhCCHCHCC$H_2$*C*$H_2$$CH_3$), 28.1 (1C, *C*H($CH_3$)$_2$), 27.5 (1C, *C*H($CH_3$)$_2$), 27.4(CH(*C*$H_3$)$_2$), 27.1 (2C, *C*H($CH_3$)$_2$), 27.0(5C, CH(*C*$H_3$)$_2$), 26.4(CH(*C*$H_3$)$_2$), 24.4 (CH(*C*$H_3$)$_2$), 23.9(2C, CH($CH_3$)$_2$), 22.7 (2C, CH($CH_3$)$_2$), 15.5(1C, 2-($CH_3$)PhCCHCHCC$H_2CH_2$*C*$H_3$). **Anal. Calcd. For $C_{64}H_{86}N_4W_2$:** N, 4.38; C, 60.09; H, 6.78. **Found:** N, 4.33; C, 59.43; H, 6.59.

Note: KC_8 is pyrophoric. Extreme caution must be exercised during its handling.

2.19 W_2Cl_2[μ-η^2:η^2-1-*n*-C_3H_7-4-2-MeC_6H_4(H)-C_4H][μ-κ^2-HC(N-2,6-^{i}Pr$_2$$C_6H_3$)$_2$]$_2$ (19)

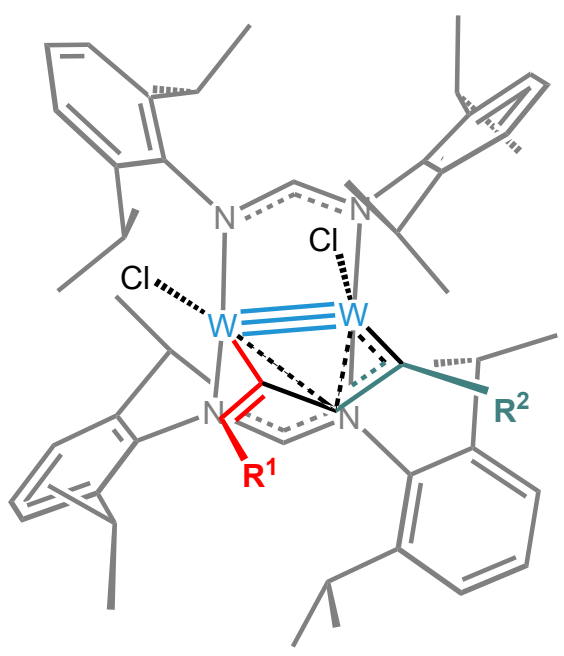

19: R^1 = 2-MeC_6H_4 R^2 = *n*-C_3H_7

A 100-mL round-flask was charged with **20** (0.0522 g, 0.0387 mmol). Toluene (30 mL) and 1-pentyne (0.43 mL of a 0.10 M solution in toluene, 0.0430 mmol) were added sequentially, which the solution immediately turned green. The resulting solution was stirred for 3 min. All volatiles were then removed under reduced pressure. The dark residue was extracted with *n*-hexane until the filtrate became colorless, and the insoluble materials were removed by filtration through a pad of Celite. The filtrate was concentrated under vacuum to afford

green solid **19** (0.0438 g, 0.0324 mmol, 83.8%). Single crystals suitable for X-ray diffraction were obtained by recrystallization from *n*-hexane at −35 °C.

^{1}H NMR (500 MHz, C_6D_6, 298 K): δ 9.38 (br, 1H, 2-(CH_3)PhCHCC*H*C^{n}Pr), 8.85(br, 1H, 2-(CH_3)PhC*H*CCHC^{n}Pr),8.75 (s, 2H, NC*H*N), 7.32 (t, 1H, 2-(CH_3)Ph-C*H*, $^3J_{H–H}$ = 7.6 Hz), 7.21 (d, 2H, Ar-C*H*, $^3J_{H–H}$ = 7.7 Hz), 7.10-7.03 (m, 6H, Ar-C*H*), 7.00-6.93 (m, 5H, Ar-C*H*), 6.88 (d, 2H, Ar-C*H*) 5.46 (br, 2H, 2-(CH_3)PhC*H*CCHCCH_2CH_2CH_3), 4.47(quint, 2H, C*H*(CH_3)$_2$, $^3J_{H–H}$ = 6.8 Hz), 4.16 (quint, 2H, C*H*(CH_3)$_2$, $^3J_{H–H}$ = 6.7 Hz), 3.02 (quint, 2H, C*H*(CH_3)$_2$, $^3J_{H–H}$ = 6.8 Hz), 2.79 (quint, 2H, C*H*(CH_3)$_2$, $^3J_{H–H}$ = 6.7 Hz), 2.39(br, 2H, 2-(CH_3)PhC*H*CCHCCH_2CH_2CH_3), 1.73(s, 3H, 2-(CH_3)PhC*H*CCHCCH_2CH_2CH_3), 1.35 (d, 18H, CH(CH_3)$_2$, $^3J_{H–H}$ = 7.2 Hz), 1.22 (d, 9H, CH(CH_3)$_2$, $^3J_{H-H}$ = 6.9 Hz), 1.18 (d, 6H, CH(CH_3)$_2$, $^3J_{H–H}$ = 6.6 Hz), 1.11 (d, 6H, CH(CH_3)$_2$, $^3J_{H–H}$ = 6.6 Hz), 1.01(d, 6H, CH(CH_3)$_2$, $^3J_{H–H}$ = 6.7 Hz),0.75(br, 6H, 2-(CH_3)PhC*H*CCHCCH_2CH_2CH_3 and CH(CH_3)$_2$). **^{13}C NMR (126 MHz, C_6D_6, 298 K):** δ 267.3 (s, 1C, 2-(CH_3)PhCHCCHC^{n}Pr), 209.5 (s, 1C, 2-(CH_3)PhCH*C*CHC^{n}Pr), 176.7(s, 1C, 2-(CH_3)PhCHC*C*HC^{n}Pr), 176.0 (s, 2C, NCHN), 176.2 (s, 3C, Dipp-Ar-C), 176.0 (s, 3C, Dipp-Ar-C), 143.9 (s, 3C, Dipp-Ar-*C*), 143.7 (s, 3C, Dipp-Ar-*C*), 138.1 (s, 1C, 2-(CH_3)Ph-*C*), 137.6 (s, 2C, 2-(CH_3)Ph-*C*), 132.5 (s, 1C, 2-(CH_3)Ph*C*HCCHC^{n}Pr), 128.7 (s, 1C, 2-(CH_3)Ph-*C*H), 127.3 (s, 3C, Dipp-Ar-*C*H), 127.2 (s, 3C, Dipp-Ar-*C*H), 127.1 (s, 1C, 2-(CH_3)Ph-*C*H), 123.7 (s, 1C, 2-(CH_3)Ph-*C*H),30.2(s, 2C, 2-(CH_3)PhCHCCHC*C*H_2*C*H_2CH_3), 29.0 (s, 3C, *C*H(CH_3)$_2$), 28.6 (s, 2C, *C*H(CH_3)$_2$), 28.3 (s, 3C, *C*H(CH_3)$_2$), 26.8 (s, 4C, CH(*C*H_3)$_2$), 26.5 (s, 4C, CH(*C*H_3)$_2$), 25.5 (s, 4C, CH(*C*H_3)$_2$), 25.1 (s, 5C, CH(*C*H_3)$_2$ and 2-(*C*H_3)Ph), 23.9 (s, 1C, 2-(CH_3)PhC*H*CCHCCH_2CH_2*C*H_3). **Anal. Calcd. for $C_{64}H_{86}Cl_2N_4W_2$:** N, 4.15; C, 56.94; H, 6.42. **Found:** N, 3.83; C, 57.05; H, 6.55.

Note: No uncommon hazards are noted.

2.20 W_2Cl_2(THF)[μ-η^1:η^2-CC(H)-2-MeC_6H_4][μ-κ^2-HC(N-2,6-^{i}Pr$_2$$C_6H_3$)$_2$]$_2$ (20)

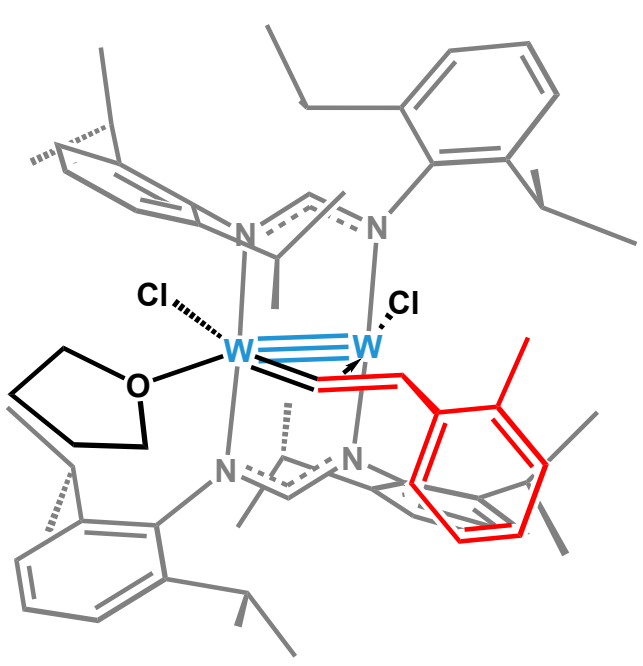

20

In a liquid-nitrogen bath, a 20 mL vial was charged with **2** (0.0438 g, 0.0341 mmol). Et_2O (6 mL) and 2-Methylphenylacetylene (0.57 mL of a 0.1 M solution in toluene, 0.0564 mmol) was added and the mixture was allowed to warm to room temperature and stirred for 3 h, during which the orange suspension turned mint-green. All volatiles were then removed under reduced pressure. *n*-Hexane (2 mL) was added and the

green suspension was transferred onto a pad of *Celite*; The residue was washed with *n*-hexane until the filtrate was colorless. The residue was extracted with THF and the insoluble materials were removed by filtration. The combined chrome-green filtrate was concentrated under vacuum to give chrome-green powder **20**·(0.0220 g, 0.0162 mmol, 65.7%). Single crystals suitable for X-ray diffraction were obtained by recrystallization from THF at −35 °C.

^{1}H NMR (500 MHz, C_6D_6, 298 K): δ 10.16 (s, ^{1}H, W_2Cl_2CC*H*(2-MePh)), 9.52 (s, 2H, NC*H*N), 8.15 (d, $^{3}J_{H–H}$ = 7.6 Hz, 1H, W_2Cl_2CC*H*(2-MePh)-Ar-*ortho*), 7.20 (t, $^{3}J_{H–H}$ = 7.2 Hz, 2H, Ar), 7.11–7.04 (m, 10H, Ar), 7.02 (d, $^{3}J_{H–H}$ = 7.6 Hz, 2H, *Ar*), 6.90 (t, $^{3}J_{H–H}$ = 7.1 Hz, 1H, *Ar*), 3.86 (br. s, 8H, Dipp-C*H*$(CH_3)_2$), 3.45 (br. s, 4H, THF-CH_2(2,5)), 2.18 (s, 3H, W_2Cl_2CCH(2-*Me*Ph)), 1.32 (br. s, 4H, THF-CH_2(3,4)), 1.31–1.01 (m, 48H, Dipp-CH(CH_3)$_2$). **^{13}C{^{1}H} NMR (126 MHz, C_6D_6, 298 K):** δ 287.5 (s, 1C, W_2Cl_2*C*CH(2-MePh)), 175.7 (s, 2C, N*C*HN), 147.1 (s, Dipp-Ar-*C*), 145.2 (br. s, Dipp-Ar-*C*), 141.6 (s, 1C, W_2Cl_2CCH(2-MePh)-Ar-*C*), 136.6 (s, 1C, W_2Cl_2CCH(2-MePh)-Ar-*C*), 131.0 (s, 1C, W_2Cl_2CCH(2-MePh)-Ar-*C*H), 127.3 (s, *Ar*), 125.8 (s, 1C, W_2Cl_2CCH(2-MePh)-Ar-*C*H), 124.1 (br. s, *Ar*), 105.1 (s, 1C, W_2Cl_2C*C*H(2-MePh)), 68.5 (s, 2C, THF-CH_2(2,5)), 28.9 (s, 16C, Dipp-CH$(CH_3)_2$), 26.4 (br. s, 8C, Dipp-*C*H$(CH_3)_2$), 25.6 (s, 2C, THF-*C*H_2(3,4)), 20.9 (s, 1C, W_2Cl_2CCH(2-*Me*Ph)). **Anal. Calcd. for $C_{63}H_{86}Cl_2N_4OW_2$:** N, 4.14; C, 55.89; H, 6.40. **Found:** N, 4.49; C, 55.64; H, 6.56.

Note: No uncommon hazards are noted.

2.21 $W_2Cl_2(C_5H_5N)[\mu\text{-}\eta^1\text{:}\eta^2\text{-}CC(H)\text{-}2\text{-}MeC_6H_4][\mu\text{-}\kappa^2\text{-}HC(N\text{-}2,6\text{-}^iPr_2C_6H_3)_2]_2$ (21)

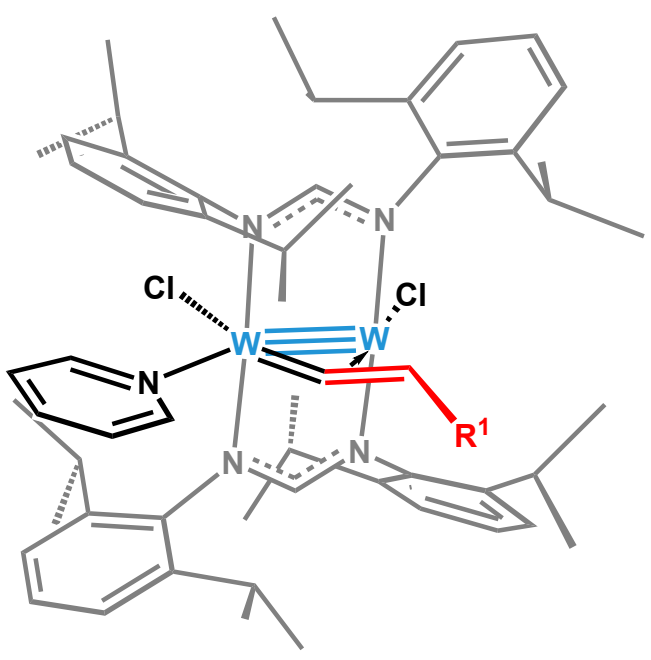

21: R^1 = 2-MeC_6H_4

A 20 mL vial was charged with **3** (0.0329 g, 0.0249 mmol). Et_2O (8 mL), pyridine (0.25 mL of a 0.1 M solution in toluene, 0.0250 mmol) and 2-methylphenylacetylene (0.28 mL of a 0.1 M solution in toluene, 0.0280 mmol) were added sequentially and the mixture was stirred at room temperature for 17 h, during which the green suspension turned brown. All volatiles were then removed under reduced pressure. The dark residue was washed with *n*-hexane until the filtrate became light green, extracted with THF, and the insoluble materials were removed by filtration through a pad of Celite. The filtrate was concentrated under vacuum to afford **21** (0.0262 g, 0.0193 mmol, 83.6%). Single crystals suitable for X-ray diffraction were obtained by diffusion of *n*-hexane into THF solution at −35 °C.

^{1}H NMR (500 MHz, C_6D_6, 298 K): δ 9.96 (s, 1H, CC*H*-2-$CH_3C_6H_4$), 9.29 (br, 2H, NC*H*N), 7.95 (d, 1H,

Ar-C*H*, ${}^3J_{H–H}$ = 7.8 Hz), 7.95 (br, 2H, (br, 2H, Py-C*H*(2,6)), 7.32 (d, 1H, Ar-C*H*, ${}^3J_{H–H}$ = 7.9 Hz), 7.06 (d, 3H, Ar-C*H*, ${}^3J_{H–H}$ = 7.3 Hz),6.85(br, 3H, Ar-C*H*), 6.75(br, 3H, Ar-C*H*), 6.60(br, 2H, Ar-C*H*), 6.33 (br, 1H, Py-C*H*(4)), 5.92 (br, 1H, Py-C*H*(3,5)), 4.38(br, 3H, C*H*(CH_3)$_2$), 4.12-4.00 (br, 5H, C*H*(CH_3)$_2$), 2.51(s, 3H, CCH-2-C$H_3$$C_6H_4$), 1.83 (br, 6H, CH(C$H_3$)$_2$), 1.56-1.99(br, 36H, CH(CH_3)$_2$), 0.74 (br, 6H, CH(CH_3)$_2$). **^{13}C NMR (126 MHz, C_6D_6, 298 K):** δ 283.0 (s, 1C, *C*CH-2-$CH_3C_6H_4$), 175.2(s, 1C, N*C*HN), 175.1(s, 1C, N*C*HN), 149.4, 146.4, 145.2, 145.0, 144.6, 143.8, 143.3, 136.2, 130.9, 129.3, 126.9, 126.6, 125.6, 125.5, 124.7, 123.9, 123.8, 123.3, 123.0, 104.18(s, 1C, C*C*H-2-$CH_3C_6H_4$), 30.2, 30.1, 29.4, 28.4, 28.3, 28.2, 27.2, 26.7, 26.5, 25.4, 25.3, 24.7, 23.2, 22.7, 21.4, 21.1. **Anal. Calcd. for $C_{64}H_{83}Cl_2N_5W_2$:** N, 5.15; C, 56.48; H, 6.15. **Found**: N, 4.64; C, 57.07; H, 6.25.

Note: No uncommon hazards are noted.

2.22 $W_2Cl_2(C_5H_5N)[\mu\text{-}\eta^1\text{:}\eta^2\text{-CC(H)-}t\text{-Bu}][\mu\text{-}\kappa^2\text{-HC(N-2,6-}^i\text{Pr}_2C_6H_3)_2]_2$ (22)

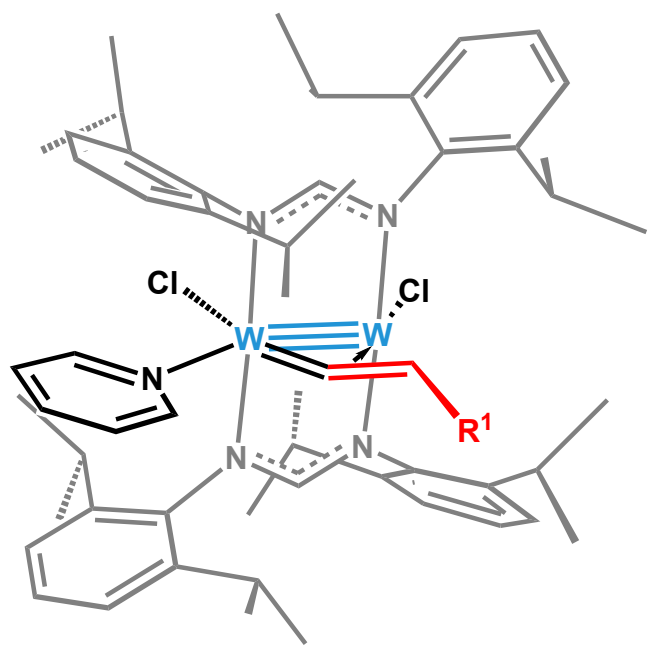

22: R[1] = ^{t}Bu

A 20 mL vial was charged with **3** (0.0965 g, 0.0722 mmol). Et_2O (8 mL) and *tert*-butylacetylene (0.87 mL of a 0.1 M solution in toluene, 0.0870 mmol) were added sequentially and the mixture was stirred at room temperature for 17 h, during which the green suspension turned brown. All volatiles were then removed under reduced pressure. The dark residue was washed with *n*-hexane until the filtrate became light green, extracted with THF, and the insoluble materials were removed by filtration through a pad of Celite. The filtrate was concentrated under vacuum to afford **22** (0.0854 g, 0.0650 mmol, 89.2%). Single crystals suitable for X-ray diffraction were obtained by recrystallization from THF at −35 °C.

^{1}H NMR (500 MHz, C_6D_6, 298 K): δ 9.38 (br, 1H, NC*H*N), 9.01 (br, 1H, NC*H*N),8.99 (br, 1H, CC*H*^{t}Bu), 7.95 (br, 2H, (br, 2H, Py-C*H*(2,6)), 7.37-7.13 (m, 2H, Ar-C*H*), 7.13-6.55 (m, 10H, Ar-C*H*), 6.31 (t, 1H, Py-C*H*(3), ${}^3J_{H–H}$ = 7.6 Hz), 5.92 (t, 1H, Py-C*H*(3,5), ${}^3J_{H–H}$ = 6.6 Hz), 4.13(br, 2H, C*H*(CH_3)$_2$), 4.19 (br, 1H, C*H*(CH_3)$_2$), 4.89 (br, 5H, C*H*(CH_3)$_2$), 1.85-1.97 (m, 9H, CH(CH_3)$_2$), 1.50(s, 9H, CCHC(CH_3)$_3$), 1.36-1.08 (m, 39H, CH(CH_3)$_2$). **^{13}C NMR (126 MHz, C_6D_6, 298 K):** δ 245.7 (s, 1C, *C*CH^{t}Bu), 175.9 (s, 1C, N*C*HN), 174.8 (s, 1C, N*C*HN), 153.7 (s, 2C, Py-*C*H(2,6)), 150.8-143.7 (br, 12C, Ar-*C*), 137.1 (s, 1C, Py-*C*H(3)), 129.3 (s, Ar-*C*H), 127.5 (s, Ar-*C*H), 126.9 (s, Ar-*C*H),124.9 (br, Ar-*C*H), 123.5(s, Ar-*C*H), 122.7 (s, 2C, Py-*C*H(3,5)), 119.3 (s, 1C, C*C*H^{t}Bu), 38.9 (s, 1C, CCH*C*(CH_3)$_3$), 29.8-26.2 (br, 16C, CH(*C*H_3)$_2$), 24.1-23.6(s, 8C, *C*H(CH_3)$_2$), 1.4 (s, 3C, CCHC(*C*H_3)$_3$). **Anal. Calcd. for $C_{61}H_{85}Cl_2N_5W_2$:**

N, 5.28; C, 55.21; H, 6.46. **Found**: N, 4.98; C, 55.10; H, 6.38.

Note: No uncommon hazards are noted.

2.23 $W_2Cl_2(C_5H_5N)[\mu\text{-}\eta^1:\eta^2\text{-}CC(H)\text{-}SiMe_3][\mu\text{-}\kappa^2\text{-}HC(N\text{-}2,6\text{-}{}^iPr_2C_6H_3)_2]_2$ (23)

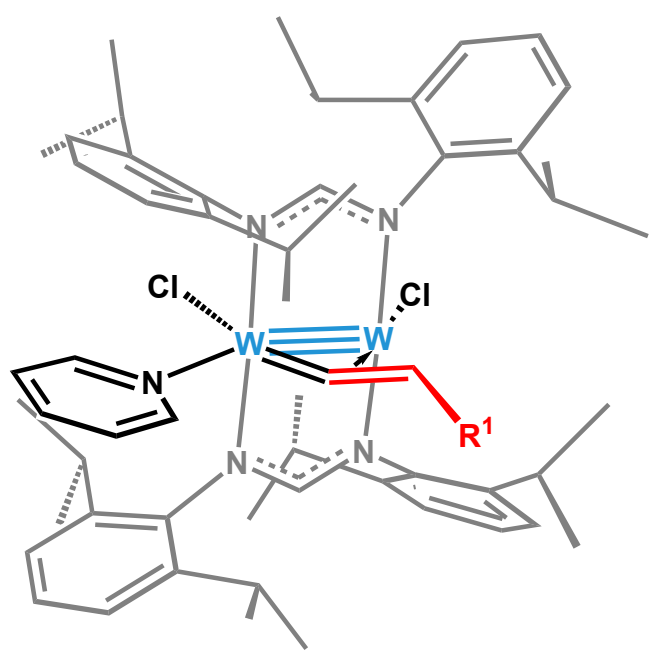

23: R[1] = SiMe3

A 20 mL vial was charged with **3** (0.1233 g, 0.0931 mmol). Et_2O (12 mL) and trimethylsilylacetylene (0.88 mL of a 0.1 M solution in toluene, 0.0880 mmol) were added sequentially and the mixture was stirred at room temperature for 17 h, during which the green suspension turned brown. All volatiles were then removed under reduced pressure. The dark residue was washed with *n*-hexane until the filtrate became light green, extracted with THF, and the insoluble materials were removed by filtration through a pad of Celite. The filtrate was concentrated under vacuum to afford **23** (0.0988 g, 0.0736 mmol, 83.6%). Single crystals suitable for X-ray diffraction were obtained by diffusion of *n*-hexane into THF solution at −35 °C.

^{1}H NMR (500 MHz, C_6D_6, 298 K): δ 9.34 (br, 1H, NC*H*N), 9.10 (br, 1H, NC*H*N),8.55 (s, 1H, CC*H*SiMe$_3$), 7.92 (br, 2H, (br, 2H, Py-C*H*(2,6)), 7.32 (br, 2H, Ar-C*H*), 7.13 (br, 2H, Ar-C*H*), 6.83 (br, 3H, Ar-C*H*), 6.70 (t, 2H, Ar-C*H*, $^3J_{H–H}$ = 7.6 Hz), 6.54 (br, 2H, Ar-C*H*), 6.30 (t, 1H, Py-C*H*(3), $^3J_{H–H}$ = 8.0 Hz), 5.90 (t, 1H, Py-C*H*(3,5), $^3J_{H–H}$ = 5.1 Hz), 4.38(br, 3H, C*H*(CH$_3$)$_2$), 4.11 (br, 2H, C*H*(CH$_3$)$_2$), 3.91 (br, 3H, C*H*(CH$_3$)$_2$), 1.79 (m, 9H, CH(C*H*$_3$)$_2$), 1.47-0.96(m, 39H, CH(C*H*$_3$)$_2$), 0.58(s, 1H, CCHSi(C*H*$_3$)$_3$). **^{13}C NMR (126 MHz, C_6D_6, 298 K):** δ 261.9 (s, 1C, *C*CHSi(CH$_3$)$_3$), 174.1 (s, 2C, N*C*HN), 153.7 (s, 2C, Py-*C*H(2,6)), 176.4 (br, 12C, Ar-*C*), 137.1 (s, 1C, Py-*C*H(3)), 125.1 (s, Ar-*C*H), 124.9 (s, Ar-*C*H), 123.4 (s, Ar-*C*H),122.6 (s, 2C, Py-*C*H(3,5)), 93.5 (s, 1C, C*C*HSi(CH$_3$)$_3$), 29.1 (br, *C*H(CH$_3$)$_2$ and CH(*C*H$_3$)$_2$), 28.0 (s, 8C, *C*H(CH$_3$)$_2$), 27.6 (s, CH(*C*H$_3$)$_2$), 3.4 (s, 3C, CCHC(*C*H$_3$)$_3$). **Anal. Calcd. for $C_{60}H_{85}Cl_2N_5SiW_2$:** N, 5.21; C, 53.66; H, 6.38. **Found:** N, 4.49; C, 54.28; H, 6.77.

Note: No uncommon hazards are noted.

2.24 $W_2Cl_2[\mu\text{-}\eta^2:\eta^2\text{-}1\text{-}C_6H_5\text{-}4\text{-}{}^tBuH\text{-}C_4H][\mu\text{-}\kappa^2\text{-}HC(N\text{-}2,6\text{-}{}^iPr_2C_6H_3)_2]_2$ (24)

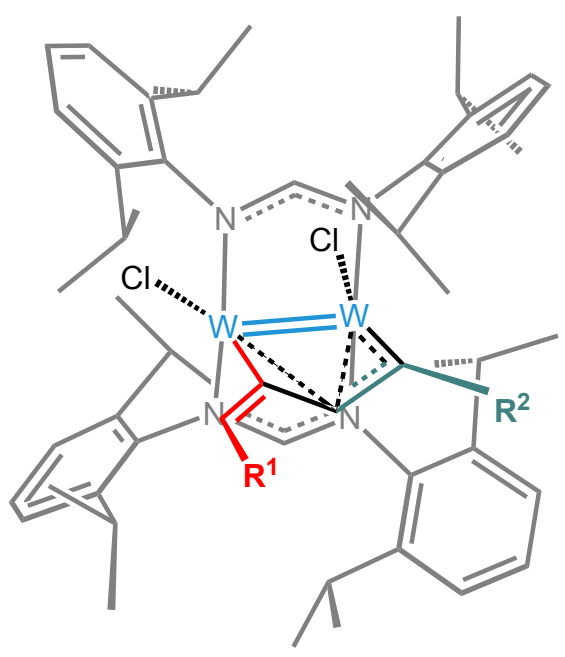

24: R^1 = ^{t}Bu, R^2 = C_6H_5

A 20-mL Schlenk tube was charged with **22** (0.0590 g, 0.0445 mmol). Toluene (15 mL) and phenylacetylene (0.49 mL of a 0.10 M solution in toluene, 0.0490 mmol) were added sequentially. The resulting solution was stirred for 17 h during which it gradually turned green. All volatiles were then removed under reduced pressure. The dark residue was washed with *n*-hexane until the filtrate became light green, extracted with Et_2O, and the insoluble materials were removed by filtration through a pad of Celite. The filtrate was concentrated under vacuum to afford **24** (0.0355 g, 0.0263 mmol, 59.1%). Single crystals suitable for X-ray diffraction were obtained by recrystallization from *n*-hexane at −35 °C.

^{1}H NMR (500 MHz, C_6D_6, 298 K): δ 8.87 (s, 2H, NC*H*N), 8.52 (d, 1H, PhCC*H*CCH^{t}Bu, $^4J_{H\text{-}H}$ = 2.1 Hz), 7.55 (d, 1H, PhCCHCC*H*^{t}Bu, $^4J_{H\text{-}H}$ = 2.1 Hz), 7.24 (dd, 2H, Dipp-Ar-*meta*, $^3J_{H–H}$ = 7.8 Hz, 1.6 Hz), 7.13 (t, 2H, Dipp-Ar-*para*, $^3J_{H–H}$ = 7.7 Hz), 6.99 (dd, 2H, Dipp-Ar-*meta*, $J_{H–H}$ = 7.8 Hz, 1.6 Hz), 6.92 (dd, 2H, Dipp-Ar-*meta*, $^3J_{H–H}$ = 7.7, 1.7 Hz), 6.91 (t, 2H, Ph-Ar-*meta*, $^3J_{H–H}$ = 7.9, 7.6 Hz), 6.86 (t, 2H, Dipp-Ar-*para*, $^3J_{H–H}$ = 7.7 Hz), 6.76 (dd, 2H, Dipp-Ar-*meta*, $J_{H–H}$ = 7.7, 1.7 Hz), 6.61 (d, 2H, Ph-Ar-*ortho*, $^3J_{H–H}$ = 7.6 Hz), 6.32 (t, 1H, Ph-Ar-para, $^3J_{H–H}$ = 7.4 Hz), 4.31(quint, 2H, C*H*(CH_3)$_2$, $^3J_{H–H}$ = 6.4 Hz), 3.74 (quint, 2H, C*H*Me_2, $^3J_{H–H}$ = 6.4 Hz), 2.82 (quint, 2H, C*H*(CH_3)$_2$, $^3J_{H–H}$ = 6.6 Hz), 2.77 (quint, 2H, C*H*(CH_3)$_2$, $^3J_{H–H}$ = 6.7 Hz), 1.76 (d, 6H, CH(C*H*$_3$)$_2$, $^3J_{H–H}$ = 6.6 Hz), 1.57 (s, 9H, PhCC*H*CCHC*Me*$_3$), 1.33 (d, 6H, CH(C*H*$_3$)$_2$, $^3J_{H\text{-}H}$ = 6.9 Hz), 1.28 (d, 6H, CH(C*H*$_3$)$_2$, $^3J_{H–H}$ = 6.6 Hz), 1.26 (d, 6H, CH(C*H*$_3$)$_2$, $^3J_{H–H}$ = 6.6 Hz), 1.16 (d, 6H, CH(C*H*$_3$)$_2$, $^3J_{H–H}$ = 6.7 Hz), 1.10 (d, 6H, CH(C*H*$_3$)$_2$, $^3J_{H–H}$ = 6.7 Hz), 1.00 (d, 6H, CH(C*H*$_3$)$_2$, $^3J_{H–H}$ = 6.6 Hz), 0.71 (d, 6H, CH(C*H*$_3$)$_2$, $^3J_{H–H}$ = 6.7 Hz). **^{13}C NMR (126 MHz, C_6D_6, 298 K):** δ 256.7 (s, 1C, Ph*C*CHCCH^{t}Bu), 199.5 (s, 1C, PhCCH*C*CH^{t}Bu), 177.0 (s, 2C, N*C*HN), 148.7 (s, 2C, Dipp-Ar-*C*), 146.3 (s, 2C, Dipp-Ar-*C*), 145.0(s, 2C, Dipp-Ar-*C*), 144.0 (s, 2C, Dipp-Ar-*C*), 143.4(s, 2C, Dipp-Ar-*C*), 143.0 (s, 2C, Dipp-Ar-*C*), 135.7 (s, 1C, Ph-*C*), 134.3 (s, 2C, Ph-Ar-*ortho*), 131.7 (s, 2C, PhCCHC*C*H^{t}Bu), 131.1 (s, 1C, Ph-Ar-*para*), 127.3 (s, 2C, Dipp-Ar-*para*), 127.0 (s, 2C, Dipp-Ar-*para*), 126.3 (s, 2C, Ph-Ar-*meta*),125.0 (s, 2C, Dipp-Ar-*meta*), 124.9 (s, 2C, Dipp-Ar-*meta*), 124.5 (s, 2C, Dipp-Ar-*meta*), 123.7 (s, 2C, Dipp-Ar-*meta*), 46.9 (s, 1C, PhC*C*HCCH^{t}Bu), 37.1 (s, 1C, PhCCHCCHC*Me*$_3$), 30.3 (s, 2C, *C*H(CH_3)$_2$), 30.3 (s, 3C, PhCCHCCHC*Me*$_3$), 28.9 (s, 4C, *C*H(CH_3)$_2$ and CH(*C*H$_3$)$_2$), 28.8 (s, 2C, *C*H(CH_3)$_2$), 27.6 (s, 2C, *C*H(CH_3)$_2$), 26.8 (s, 2C, CH(*C*H$_3$)$_2$), 26.4 (s, 2C, CH(*C*H$_3$)$_2$), 25.8 (s, 2C, CH(*C*H$_3$)$_2$), 25.7 (s, 2C, CH(*C*H$_3$)$_2$), 25.2 (s, 2C, CH(*C*H$_3$)$_2$), 25.0 (s, 2C, CH(*C*H$_3$)$_2$), 23.9 (s, 2C, CH(*C*H$_3$)$_2$). **Anal. Calcd. for $C_{64}H_{86}Cl_2N_4W_2$:** N, 4.15; C, 56.94; H, 6.42.

Found: N, 4.02; C, 57.26; H, 7.11.

Note: No uncommon hazards are noted.

2.25 W_2Cl_2[μ-η²:η²-1-*n*-C_3H_7-4-^{t}BuH-C_4H][μ-κ²-HC(N-2,6-^{i}Pr$_2$C$_6$H$_3$)$_2$]$_2$ (25)

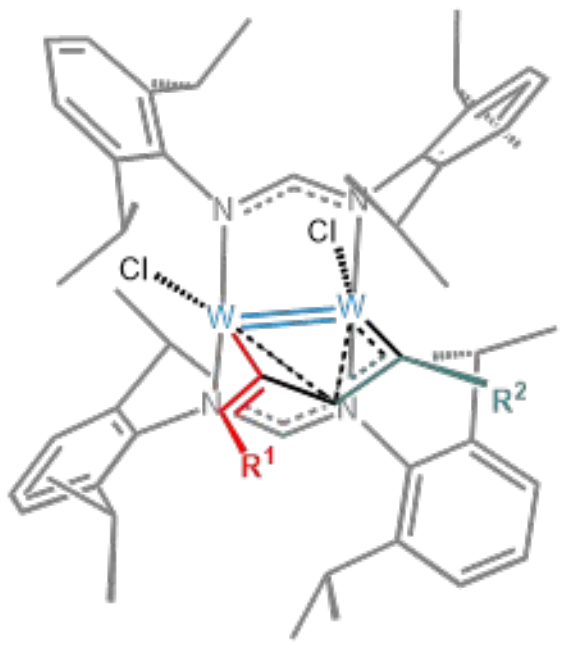

25: R¹ = ^{t}Bu, R² = *n*-C_3H_7

A 20-mL vial was charged with **22** (0.0609 g, 0.0456 mmol). Toluene (12 mL) and 1-pentyne (0.51 mL of a 0.10 M solution in toluene, 0.0510 mmol) were added sequentially. The resulting solution was stirred for 17 h during which it gradually turned dark green. All volatiles were then removed under reduced pressure. The residue was washed with TMS_2O until the filtrate turn brown to light green, extracted with *n*-pentane, and the insoluble materials were removed by filtration through a pad of Celite. The filtrate was concentrated under vacuum to afford green solid **25** (0.0206 g, 0.0157 mmol, 34.3%). Single crystals suitable for X-ray diffraction were obtained by recrystallization from *n*-pentane at −35 °C.

^{1}H NMR (500 MHz, C_6D_6, 298 K): δ 8.76 (s, 2H, NC*H*N), 8.36 (d, 1H, ^{n}PrCC*H*CCH^{t}Bu, $^4J_{H\text{-}H}$ = 2.6 Hz), 7.41 (d, 1H, ^{n}PrCCHCC*H*^{t}Bu, $^4J_{H–H}$ = 1.9 Hz), 7.22 (dd, 2H, Dipp-Ar-*meta*, $^4J_{H\text{-}H}$ = 1.7 Hz , $^3J_{H–H}$ = 7.7 Hz), 7.12(t, 2H, Dipp-Ar-*para*, $^3J_{H–H}$ = 7.7 Hz), 7.04 (dd, 2H, Dipp-Ar-*meta*, $^4J_{H\text{-}H}$ = 1.8 Hz , $^3J_{H–H}$ = 7.9 Hz), 6.98 (t, 2H, Dipp-Ar-*para*, $^3J_{H–H}$ = 7.8, 7.7 Hz), 6.98 (d, 2H, Dipp-Ar-*meta*, $^3J_{H–H}$ = 9.1 Hz), 6.92 (dd, 2H, Dipp-Ar-*meta*, $^4J_{H\text{-}H}$ = 1.8 Hz , $^3J_{H–H}$ = 7.7 Hz), 5.29(td, 2H, CH_3CH_2C*H*$_2$CC*H*CCH^{t}Bu, $^4J_{H\text{-}H}$ = 2.6 Hz , $^3J_{H–H}$ = 7.1 Hz), 4.35(quint, 2H, C*H*(CH_3)$_2$, $^3J_{H–H}$ = 6.7 Hz), 4.26 (quint, 2H, C*H*Me$_2$, $^3J_{H–H}$ = 6.7 Hz), 2.88 (quint, 2H, C*H*(CH_3)$_2$, $^3J_{H–H}$ = 6.7 Hz), 2.67 (quint, 2H, C*H*(CH_3)$_2$, $^3J_{H–H}$ = 6.7 Hz), 1.75 (d, 6H, CH(C*H*$_3$)$_2$, $^3J_{H–H}$ = 6.6 Hz), 1.51 (s, 9H, ^{n}PrCCHCCHC(C*H*$_3$)$_3$), 1.33 (d, 6H, CH(C*H*$_3$)$_2$, $^3J_{H\text{-}H}$ = 6.8 Hz), 1.32 (d, 6H, CH(C*H*$_3$)$_2$, $^3J_{H–H}$ = 6.7 Hz), 1.26 (d, 6H, CH(C*H*$_3$)$_2$, $^3J_{H–H}$ = 6.6 Hz), 1.24 (d, 6H, CH(C*H*$_3$)$_2$, $^3J_{H–H}$ = 6.6 Hz), 1.12 (d, 6H, CH(C*H*$_3$)$_2$, $^3J_{H–H}$ = 6.7 Hz), 1.07(d, 6H, CH(C*H*$_3$)$_2$, $^3J_{H–H}$ = 6.7 Hz),0.84(sext, 2H, CH_3C*H*$_2$$CH_2$CCHCCH^{t}Bu, $^3J_{H–H}$ = 7.2 Hz), 0.73 (d, 6H, CH(C*H*$_3$)$_2$, $^3J_{H–H}$ = 6.7 Hz), 0.12(t, 2H, C*H*$_3$$CH_2CH_2$CCHCCH^{t}Bu, $^3J_{H–H}$ = 7.3 Hz). **^{13}C NMR (126 MHz, C_6D_6, 298 K):** δ 266.3 (s, 1C, ^{n}Pr*C*CHCCH^{t}Bu), 199.6 (s, 1C, ^{n}PrCCH*C*CH^{t}Bu), 176.5 (s, 2C, N*C*HN), 148.7 (s, 2C, Dipp-Ar-*C*), 146.3 (s, 2C, Dipp-Ar-*C*), 146.1(s, 2C, Dipp-Ar-*C*), 144.0 (s, 2C, Dipp-Ar-*C*), 143.7(s, 2C, Dipp-Ar-*C*), 143.6 (s, 2C, Dipp-Ar-*C*), 131.7(s, 1C, ^{n}PrCCHC*C*H^{t}Bu), 127.2 (s, 2C, Dipp -Ar-*C*H), 127.2 (s, 2C, Dipp -Ar-*C*H), 124.9 (s, 2C, Dipp -Ar-*C*H), 124.8 (s, 2C, Dipp -Ar-*C*H),124.7 (s, 2C, Dipp -Ar-*C*H), 123.6(s, 2C, Dipp-Ar-*C*H), 48.7 (s, 1C, ^{n}PrC*C*HCCH^{t}Bu), 44.9 (s, 1C, CH_3CH_2*C*H$_2$CCHCCH^{t}Bu), 37.6

(s, 1C, ^{n}PrCCHCCHC(CH$_3$)$_3$),30.1 (s, 3C, ^{n}PrCCHCCHC(CH$_3$)$_3$), 29.3 (s, 2C, CH(CH$_3$)$_2$), 28.9 (s, 2C, CH(CH$_3$)$_2$), 28.8 (s, 2C, CH(CH$_3$)$_2$), 28.5 (s, 2C, CH(CH$_3$)$_2$), 27.6 (s, 2C, CH(CH$_3$)$_2$), 26.6 (s, 4C, CH(CH$_3$)$_2$), 25.7 (s, 2C, CH(CH$_3$)$_2$),25.6(s, 2C, CH(CH$_3$)$_2$), 25.5(s, 2C, CH(CH$_3$)$_2$), 25.0(s, 2C, CH(CH$_3$)$_2$), 24.9(s, 1C, CH$_3$CH$_2$CH$_2$CCHCCH^{t}Bu),24.5 (s, 2C, CH(CH$_3$)$_2$), 16.2 (s, 1C, CH$_3$CH$_2$CH$_2$CCHCCH^{t}Bu). **Anal. Calcd. for $C_{61}H_{88}Cl_2N_4W_2$:** N, 4.26; C, 55.67; H, 6.74. **Found**: N, 4.04; C, 56.49; H, 7.11.

Note: No uncommon hazards are noted.

2.26 W_2Cl_2[μ-η^2:η^2-1-*n*-C_3H_7-4-$SiMe_3$H-C_4H][μ-κ^2-HC(N-2,6-$^{i}Pr_2C_6H_3)_2]_2$ (26)

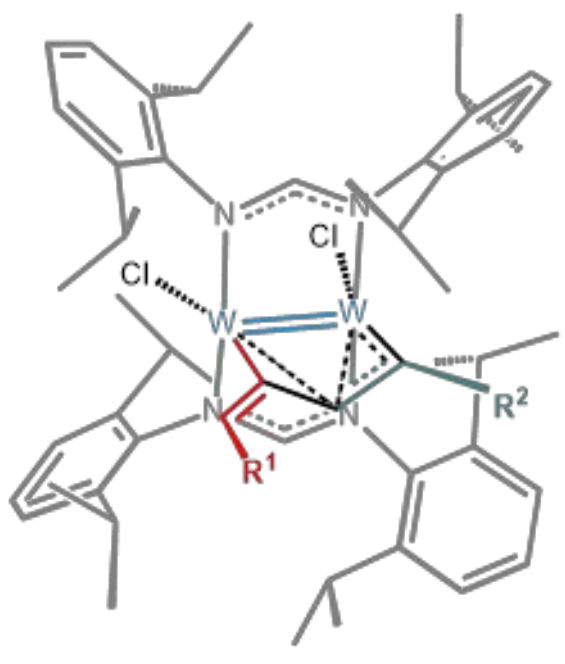

26: R^1 = SiMe$_3$, R^2 = *n*-C$_3$H$_7$

A 20-mL vial was charged with **23** (0.0581 g, 0.0433 mmol). Et$_2$O (12 mL) and 1-pentyne (0.48 mL of a 0.10 M solution in toluene, 0.0480 mmol) were added sequentially. The resulting solution was stirred for 17 h during which it gradually turned green. All volatiles were then removed under reduced pressure. The residue was washed with TMS$_2$O until the filtrate turn brown to light green, then washed with *n*-pentane/ TMS$_2$O mixture (2:1) until the filtrate become light green. The remaining residue was extracted with *n*-pentane/ TMS$_2$O mixture (2:1), and the insoluble materials were removed by filtration through a pad of Celite. The filtrate was concentrated under vacuum to afford yellow-green solid **26** (0.0216 g, 0.0162 mmol, 37.4%). Single crystals suitable for X-ray diffraction were obtained by recrystallization from Et$_2$O at −35 °C.

^{1}H NMR (500 MHz, C_6D_6, 298 K): δ 8.62 (d, 1H, ^{n}PrCC*H*CCHSi(CH$_3$)$_3$, $^4J_{H\text{-}H}$ = 2.2 Hz), 8.57 (s, 2H, NC*H*N), 8.09 (d, 1H, ^{n}PrCCHCC*H*Si(CH$_3$)$_3$, $^4J_{H–H}$ = 2.6 Hz), 7.18 (d, 1H, Dipp-Ar-*meta*, $^4J_{H\text{-}H}$ = 1.6 Hz), 7.07(t, 2H, Dipp-Ar-*para*, $^3J_{H–H}$ = 7.6 Hz), 7.01-6.93 (m, 9H, Dipp-Ar), 5.55(td, 2H, CH$_3$CH$_2$C*H$_2$*CCHCCHSi(CH$_3$)$_3$, $^4J_{H\text{-}H}$ = 2.5 Hz , $^3J_{H–H}$ = 8.2 Hz), 4.22(quint, 2H, C*H*(CH$_3$)$_2$, $^3J_{H–H}$ = 6.6 Hz), 4.05 (quint, 2H, C*H*(CH$_3$)$_2$, $^3J_{H–H}$ = 6.6 Hz), 3.31-3.22 (m, 2H, C*H*(CH$_3$)$_2$), 2.71-2.63 (m, 2H, C*H*(CH$_3$)$_2$), 2.22(sext, 2H, CH$_3$C*H$_2$*CH$_2$CCHCCHSi(CH$_3$)$_3$, $^3J_{H–H}$ = 7.4 Hz), 1.72 (d, 6H, CH(C*H$_3$*)$_2$, $^3J_{H–H}$ = 6.7 Hz), 1.38 (d, 6H, CH(C*H$_3$*)$_2$, $^3J_{H\text{-}H}$ = 6.7 Hz), 1.30 (d, 6H, CH(C*H$_3$*)$_2$, $^3J_{H–H}$ = 7.0 Hz), 1.26 (d, 6H, CH(C*H$_3$*)$_2$, $^3J_{H–H}$ = 6.9 Hz), 1.23 (d, 6H, CH(C*H$_3$*)$_2$, $^3J_{H–H}$ = 6.8 Hz), 1.22 (d, 6H, CH(C*H$_3$*)$_2$, $^3J_{H–H}$ = 6.7 Hz), 1.18 (d, 6H, CH(C*H$_3$*)$_2$, $^3J_{H–H}$ = 6.8 Hz), 0.88 (t, 3H, C*H$_3$*CH$_2$CH$_2$CCHCCHSi(CH$_3$)$_3$, $^3J_{H–H}$ = 7.1 Hz), 0.77 (d, 6H, CH(C*H$_3$*)$_2$, $^3J_{H–H}$ = 6.7 Hz) , -0.67(s, 9H, CH$_3$CH$_2$CH$_2$CCHCCHSi(C*H$_3$*)$_3$). **^{13}C NMR (126 MHz, C_6D_6, 298 K):** δ 268.8 (s, 1C, ^{n}PrCCHCCHSi(CH$_3$)$_3$), 219.1 (s, 1C,

^{n}PrCCH*C*CHSi(CH$_3$)$_3$), 177.4 (s, 2C, N*C*HN), 147.1(s, 2C, Dipp-Ar-*C*), 146.0 (s, 2C, Dipp-Ar-*C*), 146.0(s, 2C, Dipp-Ar-*C*), 145.6 (s, 2C, Dipp-Ar-*C*), 145.1 (s, 2C, Dipp-Ar-*C*), 144.6 (s, 2C, Dipp-Ar-*C*), 143.1 (s, 2C, Dipp-Ar-*C*), 130.7(s, 1C, ^{n}PrCCH*C*CHSi(CH$_3$)$_3$), 127.7 (s, 2C, Dipp -Ar-*C*H), 127.1 (s, 2C, Dipp -Ar-*C*H), 125.7 (s, 2C, Dipp -Ar-*C*H), 124.9 (s, 2C, Dipp -Ar-*C*H), 124.7(s, 2C, Dipp -Ar-*C*H), 123.5(s, 2C, Dipp-Ar-*C*H), 43.7 (s, 2C, CH$_3$*C*H$_2$*C*H$_2$CCHCCHSi(CH$_3$)$_3$), 39.4 (s, 1C, CH$_3$CH$_2$CH$_2$CCHCC*H*Si(CH$_3$)$_3$), 29.6 (s, 2C, *C*H(CH$_3$)$_2$), 28.7 (s, 2C, *C*H(CH$_3$)$_2$), 28.7 (s, 2C, *C*H(CH$_3$)$_2$), 28.2 (s, 2C, CH(*C*H$_3$)$_2$), 28.1 (s, 2C, *C*H(CH$_3$)$_2$), 27.1 (s, 2C, CH(*C*H$_3$)$_2$), 26.5 (s, 2C, CH(*C*H$_3$)$_2$),25.6(s, 2C, CH(*C*H$_3$)$_2$), 25.6(s, 2C, CH(*C*H$_3$)$_2$), 24.9(s, 2C, CH(*C*H$_3$)$_2$), 24.7(s, 2C, CH(*C*H$_3$)$_2$),23.8 (s, 2C, CH(*C*H$_3$)$_2$), 15.1(s, 1C, *C*H$_3$*C*H$_2$CH$_2$CCHCCHSi(CH$_3$)$_3$), -1.4 (s, 3C, ^{n}PrCCH*C*CHSi(*C*H$_3$)$_3$). **Anal. Calcd. for $C_{60}H_{88}Cl_2N_4W_2Si$:** N, 4.21; C, 54.10; H, 6.66. **Found**: N, 3.52; C, 54.81; H, 6.89.

Note: No uncommon hazards are noted.

2.27 $W_2Cl[\mu\text{-}\kappa^2\text{-}1\text{-}C_6H_5\text{-}4\text{-}^tBuH\text{-}C_4H][\mu\text{-}\kappa^2\text{-}HC(N\text{-}2,6\text{-}^iPr_2C_6H_3)_2]_2$ (27)

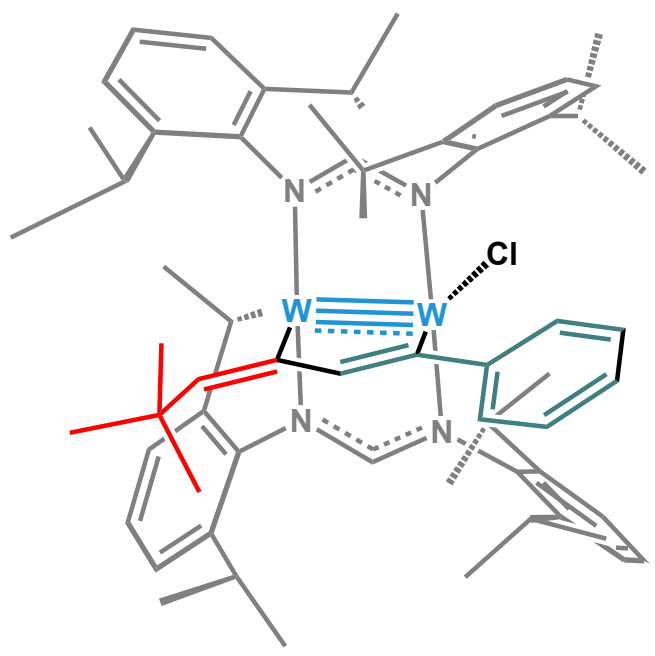

27

A 20 mL vial was charged with **24** (0.0187 g, 0.0139 mmol), and pre-cooled at −35 °C for 1 h. Pre-cooled *n*-hexane (6 mL) and KC_8 (0.0027 g, 0.0200 mmol) were added sequentially. The mixture was stirred for 18 h while gradually warming to room temperature, during which the the green suspension slowly turned dark. After the graphite was removed via filtration, all volatiles were then removed under reduced pressure. *n*-Hexane was added and the dark green suspension was transferred onto a pad of *Celite*; The residue was extracted with *n*-hexane until the filtrate was finally colorless. The insoluble materials were removed by filtration. The combined filtrate was concentrated under vacuum to give brown powder **27**·(0.0108 g, 0.00822 mmol, 59.3%). Single crystals suitable for X-ray diffraction were obtained by slow evaporation of *n*-hexane solution at room temperature.

^{1}H NMR (500 MHz, C_6D_6, 298 K): δ 8.28 (br.), 5.61 (br.), 2.41 (br.). **Evans method (500 MHz, 298 K):** μ_{eff} = 1.77 μ_B. **Anal. Calcd. for $C_{64}H_{86}ClN_4W_2$:** N, 4.26; C, 58.48; H, 6.59. **Found:** N, 4.19; C, 58.45; H, 6.62.

Note: KC_8 is pyrophoric. Extreme caution must be exercised during its handling.

2.28 W_2(μ-H)[μ-κ^2-1-C_6H_5-4-^{t}BuH-C_4H][μ-κ^3-HC(N-2,6-^{i}Pr$_2$C$_6$H$_3$)(N-2-^{i}Pr-6-(C$_3$H$_6$)C$_6$H$_3$)][μ-κ^2-HC(N-2,6-^{i}Pr$_2$C$_6$H$_3$)$_2$] (28)

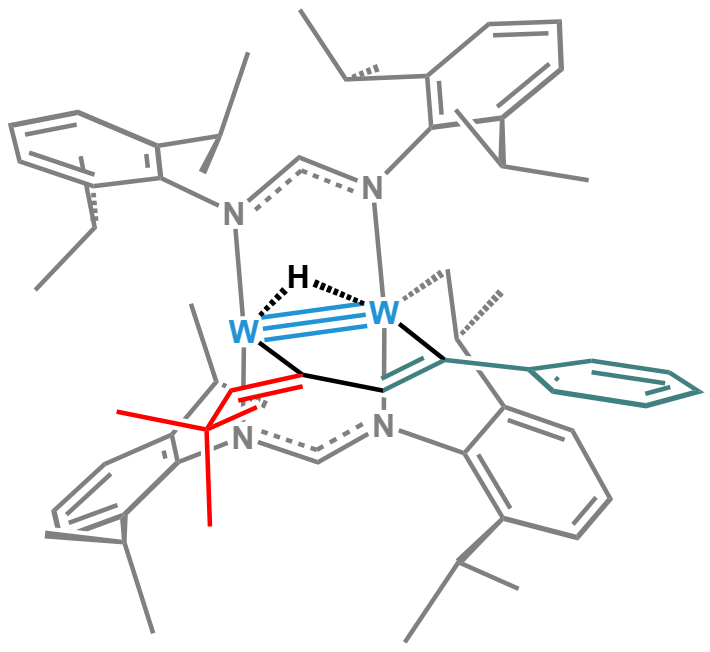

28

A 20 mL vial was charged with **24** (0.0150 g, 0.0111 mmol), and pre-cooled at −35 °C for 1 h. Pre-cooled Et_2O (6 mL) and KC_8 (0.0036 g, 0.0266 mmol) were added sequentially, resulting in an immediate color change of the green suspension to red. The mixture was stirred for 17 h while gradually warming to room temperature, during which the the red suspension turned brown. After the graphite was removed via filtration, all volatiles were then removed under reduced pressure. *n*-Hexane was added and the brown suspension was transferred onto a pad of *Celite*; The residue was extracted with *n*-hexane until the filtrate was brown then finally colorless. The insoluble materials were removed by filtration. The combined filtrate was concentrated under vacuum to give brown powder **28**·(0.0136 g, 0.0106 mmol, 95.5%). Single crystals suitable for X-ray diffraction were obtained by recrystallization from *n*-hexane at −35 °C.

^{1}H NMR (500 MHz, C_6D_6, 298 K): δ 8.15(s, 1H, PhCC*H*CCH^{t}Bu), 7.72 (s, 1H, NC*H*N), 7.27 (s, 1H, NC*H*N), 7.19 (d, 2H, dipp-Ar-*meta*, $^3J_{H\text{-}H}$ = 7.8 Hz), 7.15 (s, 1H, PhCCHCC*H*^{t}Bu), 7.12-7.00 (m, 8H, Ar-C*H*), 6.94 (d, 2H, dipp-Ar-*meta*, $^3J_{H\text{-}H}$ = 7.8 Hz), 6.90 (d, 1H, Ar-*meta*, $^3J_{H\text{-}H}$ = 7.2 Hz), 6.88 (d, 1H, Ar-*meta*, $^3J_{H\text{-}H}$ = 7.1 Hz), 6.85 (t, 2H, dipp-Ar-*para*, $^3J_{H\text{-}H}$ = 7.8 Hz), 6.70 (d, 1H, Ar-*meta*, $^3J_{H\text{-}H}$ = 7.8 Hz), 4.71(ddd, 1H, W–*H*, $^1J_{W\text{-}H}$ = 157.7, $^1J_{W\text{-}H}$ = 44.8 Hz, $^3J_{H\text{-}H}$ = 4.0 Hz), 3.76 (quint, 1H, C*H*(CH$_3$)$_2$, $^3J_{H\text{-}H}$ = 6.8 Hz), 3.91 (br, 1H, C*H*(CH$_3$)$_2$), 3.56(d, 1H, Dipp-C*H*(CH$_\alpha$H$_\beta$)(CH$_3$), $^3J_{H\text{-}H}$ = 6.2 Hz), 3.47 (quint, 1H, C*H*(CH$_3$)$_2$, $^3J_{H\text{-}H}$ = 6.7 Hz), 3.22 (quint, 1H, C*H*(CH$_3$)$_2$, $^3J_{H\text{-}H}$ = 7.2 Hz), 3.20 (quint, 1H, C*H*(CH$_3$)$_2$, $^3J_{H\text{-}H}$ = 6.6 Hz), 3.12 (quint, 1H, C*H*(CH$_3$)$_2$, $^3J_{H\text{-}H}$ = 6.7 Hz), 3.08 (dd, Dipp-CH(CH$_3$)(C*H$_\alpha$H$_\beta$*)−W, $^2J_{H\text{-}H}$ = 14.7 Hz, $^3J_{H\text{-}H}$ = 5.1 Hz), 2.89 (quint, 1H, C*H*(CH$_3$)$_2$, $^3J_{H\text{-}H}$ = 7.3 Hz), 1.60 (d, 3H, CH(C*H*$_3$)$_2$, $^3J_{H\text{-}H}$ = 6.7 Hz), 1.50 (d, 3H, CH(C*H*$_3$)$_2$, $^3J_{H\text{-}H}$ = 7.0 Hz), 1.50 (d, 3H, CH(C*H*$_3$)$_2$, $^3J_{H\text{-}H}$ = 6.8 Hz), 1.45 (s, 9H, PhCCHCCH(C*H*$_3$)$_3$), 1.24-1.17 (m, 12H, CH(C*H*$_3$)$_2$), 1.12 (d, 3H, CH(C*H*$_3$)$_2$, $^3J_{H\text{-}H}$ = 6.8 Hz), 1.11 (d, 3H, CH(C*H*$_3$)$_2$, $^3J_{H\text{-}H}$ = 6.7 Hz), 1.04 (d, 6H, CH(C*H*$_3$)$_2$, $^3J_{H\text{-}H}$ = 6.8 Hz), 0.99 (d, 3H, CH(C*H*$_3$)$_2$, $^3J_{H\text{-}H}$ = 6.7 Hz),0.61 (d, 3H, CH(C*H*$_3$)$_2$, $^3J_{H\text{-}H}$ = 6.8 Hz), 0.42 (d, 3H, CH(C*H*$_3$)$_2$, $^3J_{H\text{-}H}$ = 6.6 Hz), 0.26 (d, 3H, CH(C*H*$_3$)$_2$, $^3J_{H\text{-}H}$ = 7.0 Hz). **^{13}C NMR (126 MHz, C_6D_6, 298 K):** δ 231.9 (s, 1C, Ph*C*CHCCH^{t}Bu), 160.0 (s, 1C, N*C*HN), 156.0 (s, 1C, N*C*HN), 148.6 (s, 1C, Ar-*C*), 146.9 (s, 1C, Ar-*C*), 146.7(s, 1C, Ar-*C*), 146.4 (s, 1C, Ar-*C*), 145.2(s, 1C, Ar-*C*), 145.1 (s, 1C, Ar-*C*), 144.8 (s, 1C, Ar-*C*),

144.7 (s, 1C, Ar-*C*), 144.6 (s, 1C, Ar-*C*), 143.1 (s, 1C, Ar-*C*), 143.0 (s, 1C, Ar-*C*), 141.2 (s, 1C, Ar-*C*),140.3 (s, 1C, Ar-*C*), 139.8 (s, 1C, PhCCH*C*CH^{t}Bu), 129.3 (s, 1C, PhC*C*HCCH^{t}Bu), 126.9 (s, 1C, Ar-*C*H), 126.7 (s, 1C, Ar-*C*H), 126.4 (s, 1C, Ar-*C*H), 126.3 (s, 1C, Ar-*C*H),125.6 (s, 1C, Ar-*C*H), 125.3 (s, 1C, Ar-*C*H), 124.8 (s, 2C, Dipp-Ar-*para*), 124.7 (s, 1C, Ar-*meta*), 124.6 (s, 2C, Dipp-Ar-*meta*), 124.4 (s, 2C, Dipp-Ar-*meta*), 123.2 (s, 1C, Ar-*C*H), 123.1 (s, 1C, Ar-*C*H), 122.8 (s, 1C, Ar-*C*H), 122.2 (s, 1C, Ar-*meta*),119.1 (s, 1C, Dipp-*C*H($CH_\alpha H_\beta$)(CH_3)),106.8 (s, 1C, PhC*C*HCCH^{t}Bu), 74.6(s, 1C, Dipp-CH(*C*$H_\alpha H_\beta$)(CH_3)), 37.5 (s, 1C, *C*H(CH_3)$_2$), 33.0 (s,3C, PhCCH*C*CH (*C*H_3)$_3$), 29.0 (s, 1C, *C*H(CH_3)$_2$), 28.6(s, 1C, *C*H(CH_3)$_2$), 28.3 (s, 1C, *C*H(CH_3)$_2$), 28.2 (s, 1C, *C*HMe$_2$), 28.2 (s, 2C, *C*H(CH_3)$_2$ and CH(*C*H_3)$_2$), 28.1 (s, 1C, *C*H(CH_3)$_2$), 27.9 (s, 1C, CH(*C*H_3)$_2$), 27.6 (s, 1C, CH(*C*H_3)$_2$), 27.4 (s, 1C, CH(*C*H_3)$_2$), 27.3 (s, 1C, *C*H(CH_3)$_2$), 26.2 (s, 1C, CH(*C*H_3)$_2$), 25.5 (s, 1C, CH(*C*H_3)$_2$), 25.5 (s, 1C, CH(*C*H_3)$_2$), 25.2 (s, 1C, CH(*C*H_3)$_2$), 25.2 (s, 1C, CH(*C*H_3)$_2$), 24.9 (s, 1C, CH(*C*H_3)$_2$), 24.9 (s, 1C, CH(*C*H_3)$_2$), 24.3 (s, 1C, CH(*C*H_3)$_2$), 22.4 (s, 1C, CH(*C*H_3)$_2$), 22.1 (s, 1C, CH(*C*H_3)$_2$), 20.5 (s, 1C, CH(*C*H_3)$_2$).

Anal. Calcd. for $C_{64}H_{86}N_4W_2$: N, 4.38; C, 60.10; H, 6.78. Found: N, 4.38; C, 59.79; H, 6.46.

Note: KC_8 is pyrophoric. Extreme caution must be exercised during its handling.

3 X-ray crystallography

Single crystals data were collected on Rigaku XtaLAB HyPix-Arc 150 diffractometer with Cu-$K\alpha$ radiation (λ = 1.54178 Å) or Mo-$K\alpha$ radiation (λ = 0.71073 Å) at 100(10) K. The structure determinations and refinements were carried out using the *SHELXS*[6] and *SHELXL*[7] programs respectively on the Olex2 interface.[8] The structures were solved using direct methods, which yielded the positions of all nonhydrogen atoms. Hydrogen atoms were placed in calculated positions in the final structure refinement. Crystallographic refinement parameters are listed in Table *S*1−*S*10.

Table *S*1. Crystal and intensity collection data for **1−3**

Complex	**1**	**2**	**3**
CCDC Number	2549678	2549679	2549680
Empirical formula	$C_{66}H_{102}Cl_4LiN_4O_4W_2$	$C_{54}H_{78}Cl_3LiN_4OW_2$	$C_{64}H_{90}Cl_2N_6OW_2$
Formula weight	1531.95	1280.19	1398.01
Temperature, K	100.00(10)	100.00(10)	99.98(10)
Crystal system	triclinic	monoclinic	triclinic
Space group	P-1	$P2_1/n$	P-1
a, Å	14.4876(4)	16.9667(2)	11.9444(3)
b, Å	15.2869(5)	16.6787(2)	13.0030(3)
c, Å	22.8006(7)	23.4235(3)	21.1633(4)
α, deg	88.371(2)	90	81.926(2)
β, deg	85.313(2)	99.5740(10)	80.832(2)
γ, deg	63.022(3)	90	74.920(2)
V, Å^3	4484.9(3)	6536.12(14)	3116.26(13)
Z	2	4	2
ρ_{calc}g/cm^3	1.134	1.301	1.490
μ/mm^{-1}	6.041	7.786	7.845
F(000)	1554.0	2560.0	1412.0
Cryst dimens, mm^3	0.41 × 0.072 × 0.069	0.14 × 0.13 × 0.11	0.101 × 0.074 × 0.021
radiation wavelength: γ, Å	CuKα (λ = 1.54184)	Cu Kα (λ = 1.54184)	Cu Kα (λ = 1.54184)
2θ rang, deg	3.888 to 134.152	5.986 to 134.152	4.252 to 134.148
Limiting indices	$-17 \leq h \leq 17$<br>$-17 \leq k \leq 18$<br>$-27 \leq l \leq 26$	$-20 \leq h \leq 19$<br>$-13 \leq k \leq 19$<br>$-27 \leq l \leq 27$	$-14 \leq h \leq 14$<br>$-15 \leq k \leq 15$<br>$-23 \leq l \leq 25$
Reflections collected	65097	49180	39708
Independent reflections	15947 [R_{int} = 0.0368]	11665 [R_{int} = 0.0309]	11102 [R_{int} = 0.0265]
Data / restraints / parameters	15947/0/757	11665/6/602	11102/0/695
GOF on F^2	1.068	1.090	1.032
Final R indices [I＞2σ(I)]	R_1 = 0.0419, wR_2 = 0.1164	R_1 = 0.0413, wR_2 = 0.1122	R_1 = 0.0279, wR_2 = 0.0636
R indices (all data)	R_1 = 0.0465, wR_2 = 0.1186	R_1 = 0.0441, wR_2 = 0.1137	R_1 = 0.0322, wR_2 = 0.0602
Largest diff. peak and hole	1.80/-1.77	1.09/-2.17	2.07/-1.42

Table *S*2. Crystal and intensity collection data for **4−6**

Complex	**4**	**5**	**6**
CCDC Number	2549681	2549682	2549683
Empirical formula	$C_{54}H_{78}N_4OW_2$	$C_{132}H_{164}N_8W_4$	$C_{73}H_{98}N_4W_2$
Formula weight	1166.90	2598.10	1399.25
Temperature, K	100.00(10)	100(2)	100(1)
Crystal system	orthorhombic	triclinic	triclinic
Space group	Pbca	P-1	P-1
a, Å	19.86087(12)	18.7501(8)	12.2398(2)
b, Å	19.45343(12)	19.9150(8)	14.8728(2)
c, Å	27.50650(16)	20.5796(9)	18.9170(3)
α, deg	90	100.764(2)	85.2295(14)
β, deg	90	108.486(2)	72.7266(15)
γ, deg	90	114.659(2)	79.1005(14)
V, $Å^3$	10627.47(11)	6138.2(5)	3227.70(10)
Z	8	2	2
ρ_{calc}g/cm^3	1.459	1.406	1.440
μ/mm^{-1}	8.168	3.785	6.811
F(000)	4688.0	2616.0	1424.0
Cryst dimens, mm^3	0.115 × 0.085 × 0.054	0.06 × 0.05 × 0.01	0.17 × 0.03 × 0.03
radiation wavelength: γ, Å	Cu Kα (λ = 1.54184)	Mo Kα (λ = 0.71073)	Cu Kα (λ = 1.54184)
2θ rang, deg	6.426 to 134.156	1.123 to 26.441	4.894 to 134.156
Limiting indices	$-23 \leq h \leq 23$<br>$-23 \leq k \leq 20$<br>$-32 \leq l \leq 32$	$-23 \leq h \leq 21$<br>$-24 \leq k \leq 24$<br>$-25 \leq l \leq 25$	$-14 \leq h \leq 14$<br>$-17 \leq k \leq 17$<br>$-18 \leq l \leq 22$
Reflections collected	70620	101105	48289
Independent reflections	9490 [R_{int} = 0.0333]	25116 [R_{int} = 0.0347]	11492 [R_{int} = 0.0220]
Data / restraints / parameters	9490/0/564	25116/1224/1329	11492/0/733
GOF on F^2	1.095	1.097	1.042
Final R indices [I > 2σ(I)]	R_1 = 0.0352, wR_2 = 0.0873	R_1 = 0.0330, wR_2 = 0.0827	R_1 = 0.0180, wR_2 = 0.0451
R indices (all data)	R_1 = 0.0399, wR_2 = 0.0895	R_1 = 0.0465, wR_2 = 0.0888	R_1 = 0.0195, wR_2 = 0.0458
Largest diff. peak and hole	1.63/-1.29	2.917/-1.398	0.81/-0.71

Table *S*3. Crystal and intensity collection data for **7−9**

Complex	**7**	**8**	**9**
CCDC Number	2549684	2549685	2549686
Empirical formula	$C_{68}H_{86}N_4W_2$	$C_{68}H_{86}N_4W_2$	$C_{68}H_{86}N_4O_2W_2$
Formula weight	1327.10	1327.10	1359.11
Temperature, K	100.01(10)	99.99(10)	200(2)
Crystal system	monoclinic	triclinic	triclinic
Space group	$P2_1/n$	P-1	P-1
a, Å	15.08040(10)	11.4983(2)	11.6553(7)
b, Å	20.6737(2)	14.9460(5)	15.0472(8)
c, Å	19.0375(2)	18.8142(6)	18.8406(9)
α, deg	90	75.778(3)	98.206(2)
β, deg	94.9850(10)	72.458(2)	107.191(2)
γ, deg	90	75.642(2)	105.440(2)
V, Å^3	5912.83(9)	2935.05(16)	2990.8(3)
Z	4	2	2
ρ_{calc}g/cm^3	1.491	1.502	1.509
μ/mm^{-1}	7.405	7.459	3.891
F(000)	2680.0	1340.0	1372
Cryst dimens, mm^3	0.11 × 0.03 × 0.02	0.04 × 0.03 × 0.03	0.06 × 0.06 × 0.02
radiation wavelength: γ, Å	Cu Kα (λ = 1.54184)	Cu Kα (λ = 1.54184)	Mo Kα (λ = 0.71073)
2θ rang, deg	6.324 to 134.142	5.012 to 134.158	2.12 to 25.27
Limiting indices	$-14 \le h \le 18$<br>$-24 \le k \le 24$<br>$-20 \le l \le 22$	$-11 \le h \le 13$<br>$-17 \le k \le 17$<br>$-22 \le l \le 22$	$-14 \le h \le 13$<br>$-18 \le k \le 18$<br>$-22 \le l \le 22$
Reflections collected	46108	32892	102034
Independent reflections	10556 [R_{int} = 0.0215]	10397 [R_{int} = 0.0291]	10397 [R_{int} = 0.0587]
Data / restraints / parameters	10556/0/685	10397/67/716	10715/1/701
GOF on F^2	1.038	1.088	1.063
Final R indices [$I > 2\sigma(I)$]	R_1 = 0.0191, wR_2 = 0.0473	R_1 = 0.0322, wR_2 = 0.0815	R_1 = 0.0228, wR_2 = 0.0483
R indices (all data)	R_1 = 0.0209, wR_2 = 0.0480	R_1 = 0.0396, wR_2 = 0.0840	R_1 = 0.0343, wR_2 = 0.0532
Largest diff. peak and hole	0.58/-0.91	1.52/-2.03	0.886/-0.602

Table *S*4. Crystal and intensity collection data for **10−12**

Complex	**10**	**11**	**12**
CCDC Number	2549687	2549688	2549689
Empirical formula	$C_{66}H_{80}F_2N_4W_2$	$C_{60}H_{86}N_4W_2$	$C_{66}H_{82}Cl_2N_4W_2$
Formula weight	1335.04	1231.02	1369.95
Temperature, K	100(2)	99.99(10)	100.01(10)
Crystal system	triclinic	triclinic	monoclinic
Space group	P-1	P-1	$P2_1/n$
a, Å	11.7767	11.9904(3)	19.6372(2)
b, Å	15.0100	12.6426(2)	13.1573(2)
c, Å	18.5341	21.0374(4)	23.1572(3)
α, deg	86.362	73.799(2)	90
β, deg	86.022	83.777(2)	98.6350(10)
γ, deg	86.665	62.833(2)	90
V, $Å^3$	3176.4	2723.90(11)	5915.36(13)
Z	2	2	4
$\rho_{calc}g/cm^3$	1.396	1.501	1.538
μ/mm^{-1}	3.664	7.984	8.233
F(000)	1340	1244.0	2752.0
Cryst dimens, mm^3	0.19 × 0.17 × 0.05	0.062 × 0.062 × 0.052	0.142 × 0.101 × 0.09
radiation wavelength: γ, Å	Mo Kα (λ = 0.71073)	Cu Kα (λ = 1.54184)	Cu Kα (λ = 1.54184)
2*θ* rang, deg	1.103 to 26.419	4.376 to 150.38	5.508 to 134.138
Limiting indices	$-14 \leq h \leq 13$<br>$-18 \leq k \leq 18$<br>$-22 \leq l \leq 22$	$-15 \leq h \leq 15$<br>$-15 \leq k \leq 15$<br>$-25 \leq l \leq 25$	$-22 \leq h \leq 23$<br>$-15 \leq k \leq 15$<br>$-27 \leq l \leq 27$
Reflections collected	54235	17590	42661
Independent reflections	13007 [R_{int} = 0.0274]	17590 [R_{int} = 0.0557]	10561 [R_{int} = 0.0245]
Data / restraints / parameters	13007/0/683	17590/0/615	10561/225/738
GOF on F^2	1.053	1.093	1.288
Final R indices [I > 2*σ*(I)]	R_1 = 0.0162, wR_2 = 0.0370	R_1 = 0.0343, wR_2 = 0.0982	R_1 = 0.0421, wR_2 = 0.0901
R indices (all data)	R_1 = 0.0202, wR_2 = 0.0387	R_1 = 0.0364, wR_2 = 0.0998	R_1 = 0.0430, wR_2 = 0.0905
Largest diff. peak and hole	0.592/-0.332	1.93/-1.55	1.47/-1.51

Table *S*5. Crystal and intensity collection data for **13−15**

Complex	**13**	**14**	**15**
CCDC Number	2549690	2549691	2549692
Empirical formula	$C_{72}H_{96}Cl_2N_4OW_2$	$C_{68}H_{86}Cl_2N_4O_2W_2$	$C_{60}H_{86}Cl_2N_4W_2$
Formula weight	1472.12	1430.00	1301.92
Temperature, K	99.99(10)	100.00(10)	100.00(10)
Crystal system	triclinic	triclinic	monoclinic
Space group	P-1	P-1	$P2_1/n$
a, Å	11.74680(10)	12.9524(2)	20.1523(2)
b, Å	16.8231(2)	16.9517(3)	12.22440(10)
c, Å	17.44260(10)	17.0000(3)	23.5581(3)
α, deg	89.8650(10)	71.616(2)	90
β, deg	78.4280(10)	69.6810(10)	98.6080(10)
γ, deg	75.0640(10)	74.5510(10)	90
V, $Å^3$	3258.37(5)	3269.69(11)	5738.16(10)
Z	2	2	4
$\rho_{calc} g/cm^3$	1.500	1.452	1.507
μ/mm^{-1}	7.526	7.496	8.450
F(000)	1492.0	1440.0	2624.0
Cryst dimens, mm^3	0.08 × 0.08 × 0.07	0.05 × 0.04 × 0.02	0.06 × 0.05 × 0.04
radiation wavelength: γ, Å	Cu Kα (λ = 1.54184)	Cu Kα (λ = 1.54184)	Cu Kα (λ = 1.54184)
2θ rang, deg	5.178 to 134.16	5.582 to 134.152	5.388 to 134.156
Limiting indices	$-14 \leq h \leq 11$<br>$-20 \leq k \leq 20$<br>$-20 \leq l \leq 20$	$-10 \leq h \leq 15$<br>$-20 \leq k \leq 20$<br>$-20 \leq l \leq 20$	$-23 \leq h \leq 24$<br>$-14 \leq k \leq 14$<br>$-28 \leq l \leq 27$
Reflections collected	50157	42394	42284
Independent reflections	11576 [R_{int} = 0.0224]	11617 [R_{int} = 0.0508]	10238 [R_{int} = 0.0241]
Data / restraints / parameters	11576/0/759	11617/0/721	10238/0/635
GOF on F^2	1.056	1.054	1.052
Final R indices [I＞2σ(I)]	R_1 = 0.0220, wR_2 = 0.0532	R_1 = 0.0408, wR_2 = 0.1371	R_1 = 0.0179, wR_2 = 0.0431
R indices (all data)	R_1 = 0.0236, wR_2 = 0.0539	R_1 = 0.0682, wR_2 = 0.1977	R_1 = 0.0191, wR_2 = 0.0436
Largest diff. peak and hole	1.31/-0.90	3.01/-2.80	0.45/-0.62

Table *S*6. Crystal and intensity collection data for **16−18**

Complex	**16**	**17**	**18**
CCDC Number	2549693	2549694	2549695
Empirical formula	$C_{64}H_{85}Cl_2N_4W_2$	$C_{59}H_{78}Cl_2N_4W_2$	$C_{64}H_{86}N_4W_2$
Formula weight	1348.95	1281.85	1279.06
Temperature, K	100.00(10)	100.00(14)	99.99(10)
Crystal system	triclinic	triclinic	monoclinic
Space group	P-1	P-1	$P2_1/n$
a, Å	13.20945(17)	10.77746(14)	19.5534(2)
b, Å	13.91671(16)	12.1050(2)	13.89685(17)
c, Å	17.92536(19)	21.7408(3)	20.8335(2)
α, deg	96.0288(9)	99.5117(14)	90
β, deg	100.2585(10)	95.5813(11)	92.1085(10)
γ, deg	111.2946(11)	101.4810(13)	90
V, Å^3	2968.99(6)	2716.39(7)	5657.28(12)
Z	2	2	4
ρ_{calc}g/cm^3	1.509	1.567	1.502
μ/mm^{-1}	8.190	8.918	7.714
F(000)	1358.0	1284.0	2584.0
Cryst dimens, mm^3	0.05 × 0.03 × 0.03	0.05 × 0.04 × 0.02	0.08 × 0.03 × 0.02
radiation wavelength: γ, Å	Cu Kα (λ = 1.54184)	Cu Kα (λ = 1.54184)	Cu Kα (λ = 1.54184)
2θ rang, deg	5.098 to 149.102	7.59 to 148.856	6.088 to 146.46
Limiting indices	$-16 \le h \le 16$<br>$-17 \le k \le 17$<br>$-19 \le l \le 22$	$-12 \le h \le 13$<br>$-14 \le k \le 14$<br>$-26 \le l \le 26$	$-23 \le h \le 24$<br>$-16 \le k \le 17$<br>$-25 \le l \le 23$
Reflections collected	57251	51131	38842
Independent reflections	11521 [R_{int} = 0.0292	10492 [R_{int} = 0.0439]	10947 [R_{int} = 0.0326]
Data / restraints / parameters	11521/252/732	10492/18/631	10947/0/648
GOF on F^2	1.039	1.092	1.144
Final R indices [I>2σ(I)]	R_1 = 0.0237,<br>wR_2 = 0.0568	R_1 = 0.0423<br>wR_2 = 0.1160	R_1 = 0.0544,<br>wR_2 = 0.1138
R indices (all data)	R_1 = 0.0268,<br>wR_2 = 0.0583	R_1 = 0.0514<br>wR_2 = 0.1208	R_1 = 0.0599,<br>wR_2 = 0.1159
Largest diff. peak and hole	0.82/-0.98	4.41/-1.68	2.06/-2.07

Table *S*7. Crystal and intensity collection data for **19−21**

Complex	**19**	**20**	**21**
CCDC Number	2549700	2549699	2549696
Empirical formula	$C_{64}H_{86}Cl_2N_4W_2$	$C_{63}H_{86}Cl_2N_4OW_2$	$C_{64}H_{83}Cl_2N_5W_2$
Formula weight	1349.96	1353.95	1360.95
Temperature, K	100.00(10)	100.00(10)	100.00(12)
Crystal system	triclinic	monoclinic	monoclinic
Space group	P-1	$P2_1/n$	$P2_1/c$
a, Å	12.38780(10)	12.0630(2)	20.1954(2)
b, Å	13.1958(2)	24.0203(3)	12.6387(2)
c, Å	21.6565(2)	20.3879(3)	26.4043(3)
α, deg	72.9540(10)	90	90
β, deg	81.1580(10)	101.5550(10)	94.1730(10)
γ, deg	75.1800(10)	90	90
V, Å^3	3260.12(7)	5787.81(15)	6721.66(15)
Z	2	4	4
ρ_{calc}g/cm^3	1.375	1.554	1.345
μ/mm^{-1}	7.459	8.417	7.245
F(000)	1360.0	2728.0	2736.0
Cryst dimens, mm^3	0.07 × 0.05 × 0.04	0.06 × 0.04 × 0.03	0.11 × 0.02 × 0.02
radiation wavelength: γ	Cu Kα (λ = 1.54184)	Cu Kα (λ = 1.54184)	Cu Kα (λ = 1.54184)
2*θ* rang, deg	7.326 to 148.884	5.754 to 134.15	6.714 to 146.12
Limiting indices	$-15 \leq h \leq 14$<br>$-16 \leq k \leq 16$<br>$-26 \leq l \leq 26$	$-14 \leq h \leq 14$,<br>$-28 \leq k \leq 25$,<br>$-24 \leq l \leq 24$	$-24 \leq h \leq 25$<br>$-15 \leq k \leq 14$<br>$-31 \leq l \leq 32$
Reflections collected	60131	43203	67081
Independent reflections	12646 [R_{int} = 0.0405]	10337 [R_{int} = 0.0249]	13099 [R_{int} = 0.0472]
Data / restraints / parameters	12646/39/691	10337/20/676	13099/2236/1229
GOF on F^2	1.059	1.085	1.145
Final R indices [I > 2*σ*(I)]	R_1 = 0.0269,<br>wR_2 = 0.0674	R_1 = 0.0268,<br>wR_2 = 0.0627	R_1 = 0.0530,<br>wR_2 = 0.1155
R indices (all data)	R_1 = 0.0303,<br>wR_2 = 0.0689	R_1 = 0.0292,<br>wR_2 = 0.0637	R_1 = 0.0584,<br>wR_2 = 0.1174
Largest diff. peak and hole	1.29/-1.65	1.45/-1.07	1.66/-1.73

Table *S*8. Crystal and intensity collection data for **22−24**

Complex	**22**	**23**	**24**
CCDC Number	2549697	2549698	2549701
Empirical formula	$C_{61}H_{85}Cl_2N_5W_2$	$C_{60}H_{85.31}Cl_2N_5SiW_2$	$C_{64}H_{86}Cl_2N_4W_2$
Formula weight	1326.93	1343.32	1349.96
Temperature, K	99.99(10)	100.00(13)	99.99(10)
Crystal system	monoclinic	monoclinic	monoclinic
Space group	$P2_1/n$	$P2_1/n$	$P2_1/c$
a, Å	16.57650(10)	16.9517(2)	15.99240(10)
b, Å	16.73890(10)	16.9933(2)	18.3733(2)
c, Å	23.22410(10)	23.3629(3)	20.2907(2)
α, deg	90	90	90
β, deg	98.0610(10)	97.7661(12)	98.3570(10)
γ, deg	90	90	90
V, Å^3	6380.37(6)	6668.33(15)	5898.77(10)
Z	4	4	4
ρ_{calc}g/cm^3	1.381	1.338	1.520
μ/mm^{-1}	7.616	7.460	8.245
F(000)	2672.0	2705.0	2720.0
Cryst dimens, mm^3	0.16 × 0.12 × 0.07	0.1 × 0.05 × 0.05	0.04 × 0.04 × 0.01
radiation wavelength: γ	Cu Kα (λ = 1.54184)	Cu Kα (λ = 1.54184)	Cu Kα (λ = 1.54184)
2θ rang, deg	6.162 to 146.878	6.07 to 146.528	6.522 to 134.16
Limiting indices	$-20 \le h \le 18$<br>$-20 \le k \le 19$<br>$-27 \le l \le 28$	$-17 \le h \le 20$,<br>$-20 \le k \le 20$<br>$-28 \le l \le 28$	$-18 \le h \le 19$, -<br>$19 \le k \le 21$, -<br>$24 \le l \le 23$
Reflections collected	46136	50205	39143
Independent reflections	12451 [R_{int} = 0.0281]	12877 [R_{int} = 0.0190]	10506 [R_{int} = 0.0328]
Data / restraints / parameters	12451/96/690	12877/347/754	10506/0/673
GOF on F^2	1.057	1.161	1.037
Final R indices [I > 2σ(I)]	R_1 = 0.0324,<br>wR_2 = 0.0830	R_1 = 0.0434,<br>wR_2 = 0.1049	R_1 = 0.0264,<br>wR_2 = 0.0583
R indices (all data)	R_1 = 0.0343,<br>wR_2 = 0.0842	R_1 = 0.0445,<br>wR_2 = 0.1054	R_1 = 0.0322,<br>wR_2 = 0.0602
Largest diff. peak and hole	3.35/-1.20	2.22/-1.62	2.07/-1.42

Table *S*9. Crystal and intensity collection data for **25**−**27**

Complex	**25**	**26**	**27**
CCDC Number	2549702	2549703	2549704
Empirical formula	$C_{66}H_{100}Cl_2N_4W_2$	$C_{60}H_{87}Cl_2N_4SiW_2$	$C_{64}H_{86}ClN_4W_2$
Formula weight	1388.09	1331.02	1314.51
Temperature, K	99.99(10)	99.99(10)	100.02(13)
Crystal system	triclinic	monoclinic	monoclinic
Space group	P-1	P2$_1$/n	P2$_1$/n
a, Å	12.4063(3)	13.16067(14)	14.57540(10)
b, Å	13.6391(3)	23.6656(2)	20.5155(2)
c, Å	18.9156(5)	19.27180(19)	19.8063(2)
α, deg	93.040(2)	90	90
β, deg	96.086(2)	100.8020(10)	96.0530(10)
γ, deg	93.831(2)	90	90
V, Å^3	3169.74(14)	5895.95(11)	5889.49(9)
Z	2	4	4
ρ_{calc}g/cm^3	1.454	1.499	1.483
μ/mm^{-1}	7.683	8.426	7.834
F(000)	1412.0	2684.0	2652.0
Cryst dimens, mm^3	0.2 × 0.15 × 0.03	0.1 × 0.08 × 0.02	0.12 × 0.06 × 0.05
radiation wavelength: γ	Cu Kα (λ = 1.54184)	Cu Kα (λ = 1.54184)	Cu Kα (λ = 1.54184)
2θ rang, deg	7.184 to 148.978	5.978 to 146.438	6.22 to 151.13
Limiting indices	-15 ≤ h ≤ 15	-16 ≤ h ≤ 16	-17 ≤ h ≤ 16
	-15 ≤ k ≤ 16,	-28 ≤ k ≤ 27	-25 ≤ k ≤ 24
	-23 ≤ l ≤ 21	-23 ≤ l ≤ 23	-24 ≤ l ≤ 23
Reflections collected	39278	57720	60913
Independent reflections	12159 [R_{int} = 0.0335]	11508 [R_{int} = 0.0393]	11656 [R_{int} = 0.0227]
Data / restraints / parameters	12159/0/694	11508/0/642	11656/0/659
GOF on F^2	1.118	1.056	1.067
Final R indices	R_1 = 0.0342,	R_1 = 0.0349,	R_1 = 0.0218,
[I>2σ(I)]	wR_2 = 0.0924	wR_2 = 0.0918	wR_2 = 0.0524
R indices (all data)	R_1 = 0.0367, wR_2 = 0.0938	R_1 = 0.0415, wR_2 = 0.0955	R_1 = 0.0225, wR_2 = 0.0528
Largest diff. peak and hole	1.74/-2.32	1.07/-1.88	0.69/-1.53

Table *S*10. Crystal and intensity collection data for **28**

Complex	**28**
CCDC Number	2549705
Empirical formula	$C_{64}H_{84}N_4W_2$
Formula weight	1277.05
Temperature, K	100.01(10)
Crystal system	triclinic
Space group	P-1
a, Å	12.41280(16)
b, Å	13.50598(17)
c, Å	20.4797(3)
α, deg	95.1858(10)
β, deg	92.7166(10)
γ, deg	114.4693(12)
V, Å^3	3098.53(7)
Z	2
ρ_{calc}g/cm^3	1.369
μ/mm^{-1}	7.042
F(000)	1288.0
Cryst dimens, mm^3	0.04 × 0.03 × 0.02
radiation wavelength: γ	Cu Kα (λ = 1.54184)
2θ rang, deg	7.244 to 149.486
Limiting indices	$-15 \le h \le 14$
	$-16 \le k \le 16$
	$-25 \le l \le 23$
Reflections collected	41549
Independent reflections	11997 [R_{int} = 0.0317]
Data / restraints / parameters	11997/0/649
GOF on F^2	1.068
Final R indices	R_1 = 0.0282,
[I＞2σ(I)]	wR_2 = 0.0691
R indices (all data)	R_1 = 0.0339,
	wR_2 = 0.0711
Largest diff. peak and hole	1.68/-1.21

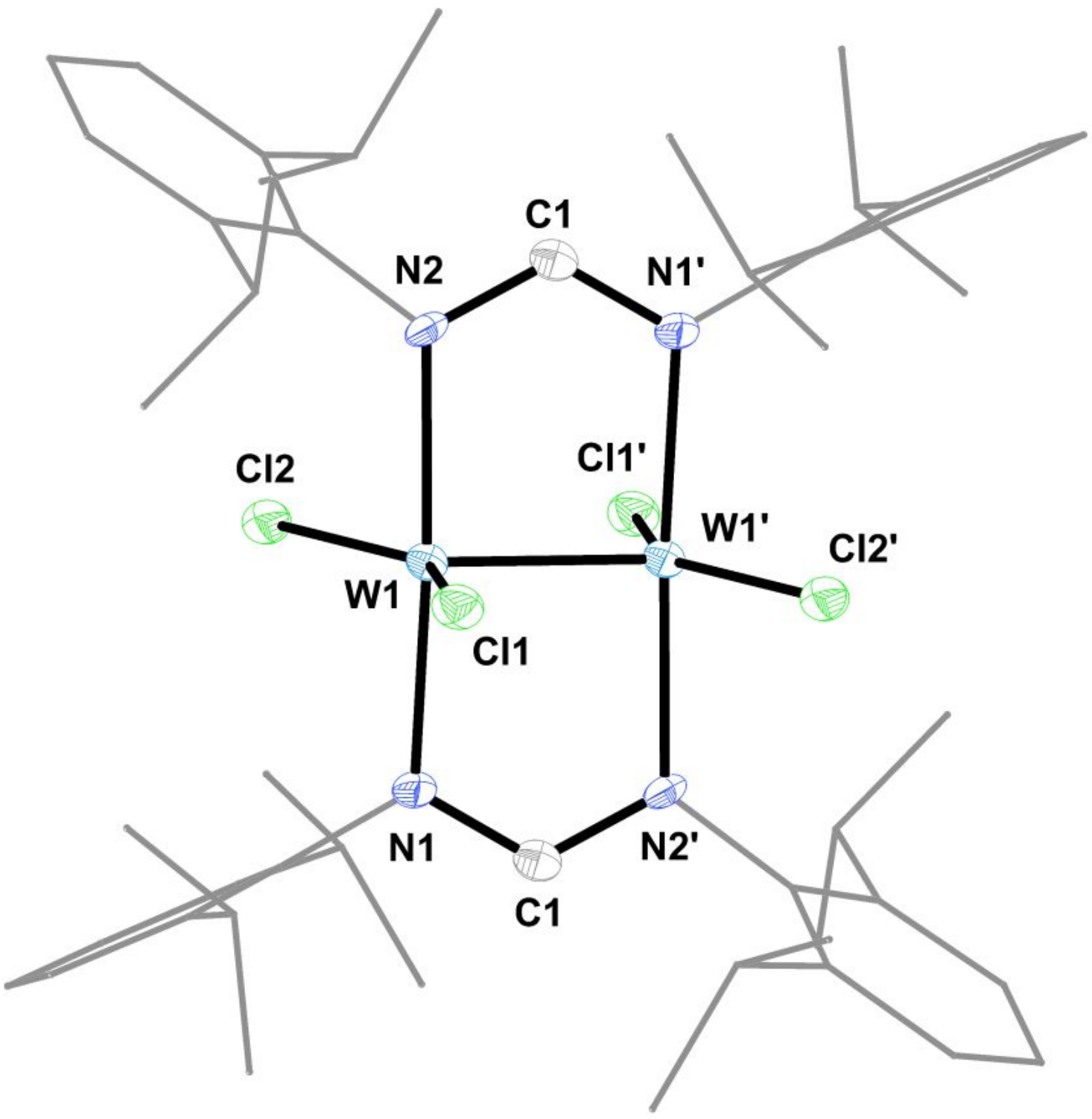

Figure. *S*1 The solid-state molecular structure of **1** drawn with 30% thermal ellipsoids probability level. Counter cation and hydrogen atoms are omitted for the sake of clarity. Selected bond lengths (Å) and angles (°): W(1)–W(1') 2.2535(4), W(1)–Cl(1) 2.3679(11), W(1)–Cl(2) 2.3748(12), W(1)–N(1) 2.134(4), W(1)–N(2') 2.133(3), N(1)–C(1) 1.322(6), N(2)–C(1) 1.325(6); W(1)–W(1')–Cl(1') 98.89(3), W(1)–W(1')–Cl(2') 112.40(3), W(1')–W(1)–N(1) 90.30(10), W(1')–W(1)–N(2) 90.81(10), Cl(1)–W(1)–Cl(2) 148.71(4), Cl(1)–W(1)–N(1) 92.93(11), N(1)–W(1)–N(2') 171.98(5); N(2)–W(1')–W(1)–N(2') 0.000.

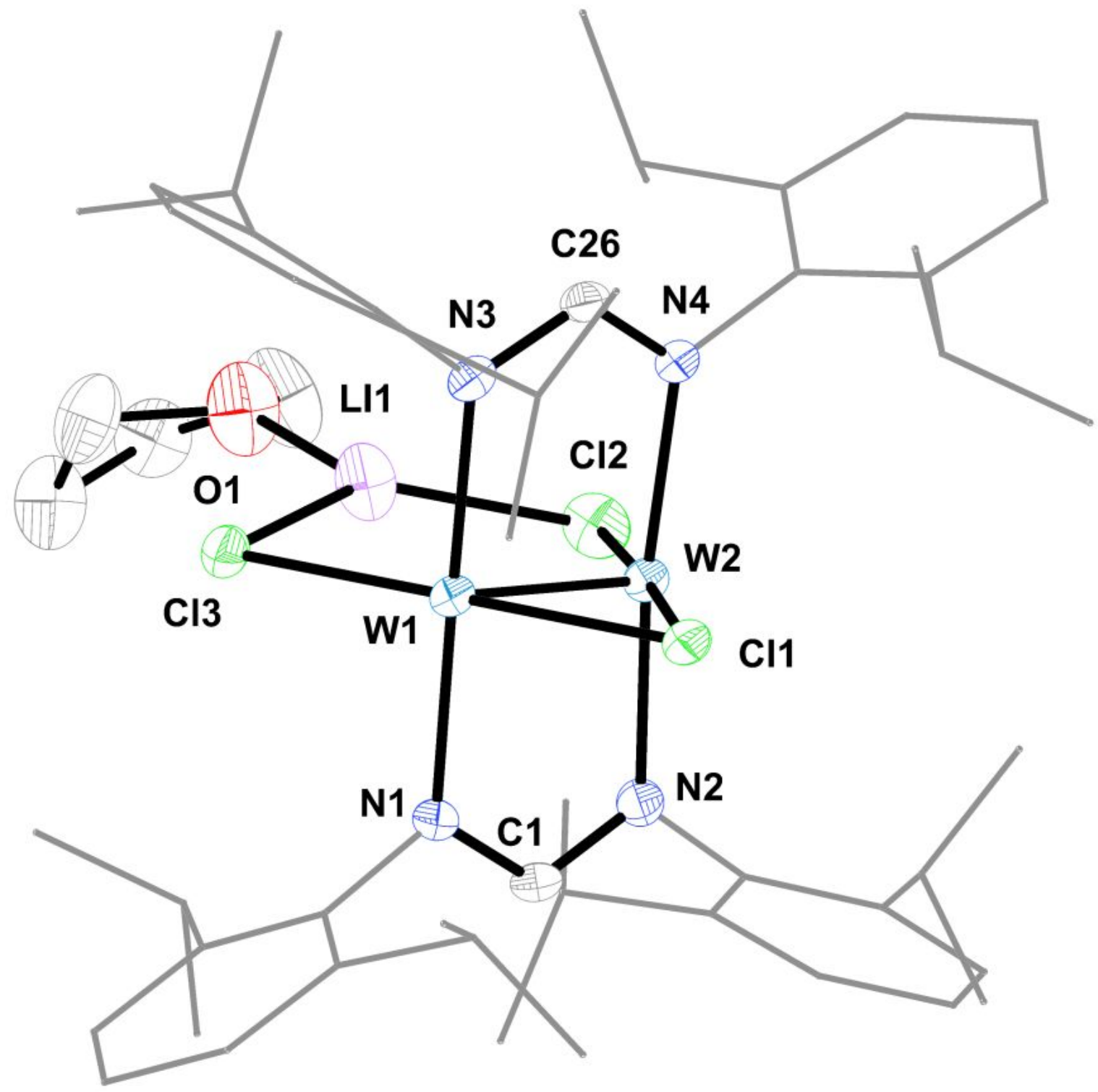

Figure. *S*2 The solid-state molecular structure of **2** drawn with 30% thermal ellipsoids probability level. Hydrogen atoms are omitted for the sake of clarity. Selected bond lengths (Å) and angles (°): W(1)–W(2) 2.2017(3), W(1)–Cl(1) 2.4876(11), W(1)–Cl(3) 2.4082(11), W(1)–N(1) 2.111(4), W(1)–N(3) 2.105(4), W(1)···Li(1) 3.169(10), W(2)–Cl(1) 2.4996(12), W(2)–Cl(2) 2.4117(14), W(2)–N(2) 2.107(4), W(2)–N(4) 2.114(4), W(2)···Li(1) 3.19(1), Cl(2)–Li(1) 2.287(11), Cl(3)–Li(1) 2.294(10), O(1)–Li(1) 1.901(11), N(1)–C(1) 1.333(6), N(2)–C(1) 1.337(6), N(3)–C(26) 1.342(6), N(4)–C(26) 1.338(6); W(1)–W(2)–Cl(1) 63.52(3), W(1)–W(2)–Cl(2) 114.21(4), W(1)–Cl(1)–W(2) 53.40(2), W(2)–W(1)–Cl(1) 64.08(3), W(2)–W(1)–Cl(3) 115.95(3), Cl(1)–W(1)–Cl(3) 179.93(5), Cl(1)–W(2)–Cl(2) 177.32(4), Cl(2)–Li(1)–Cl(3) 135.8(5), N(1)–W(1)–N(3) 173.85(15), N(2)–W(2)–N(4) 172.83(15); N(1)–W(1)–W(2)–N(4) 6.116.

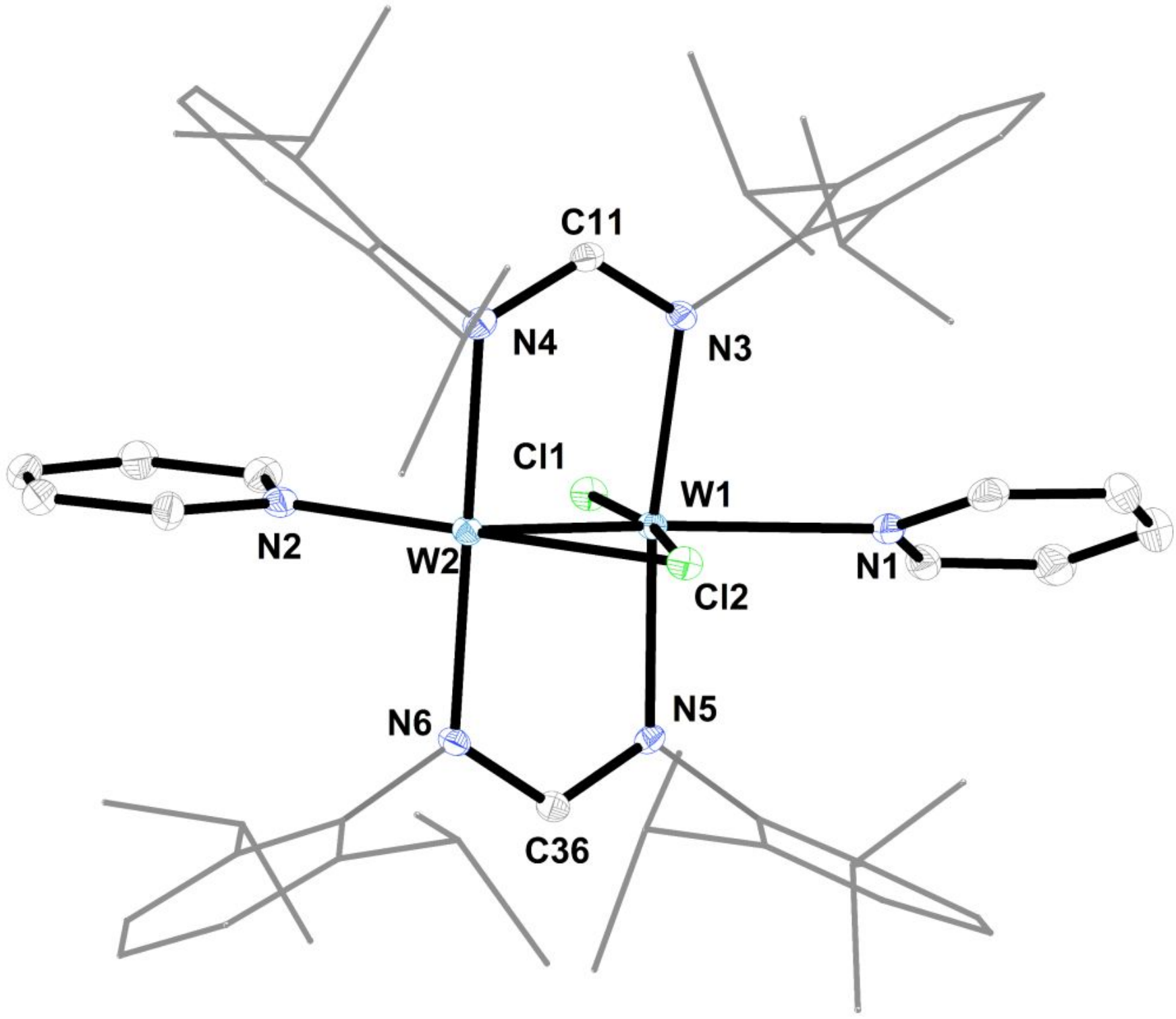

Figure. *S*3 The solid-state molecular structure of **3** drawn with 30% thermal ellipsoids probability level. Hydrogen atoms are omitted for the sake of clarity. Selected bond lengths (Å) and angles (°): W(1)–W(2) 2.2393(2), W(1)–Cl(1) 2.4086(8), W(1)–Cl(2) 2.5373(8), W(1)–N(1) 2.442(3), W(1)–N(3) 2.171(3), W(1)–N(5) 2.141(3), W(2)–Cl(2) 2.5141(8), W(2)–N(2) 2.132(3), W(2)–N(2) 2.112(3), W(2)–N(6) 2.115(3), N(3)–C(11) 1.337(5), N(4)–C(11) 1.329(5), N(5)–C(36) 1.334(5), N(6)–C(36) 1.335(5); W(1)–W(2)–Cl(2) 64.22(2), W(1)–W(2)–N(2) 117.53(8), W(1)–Cl(2)–W(2) 52.628(17), W(2)–W(1)–Cl(1) 109.05(2), W(2)–W(1)–Cl(2) 63.15(2), W(2)–W(1)–N(1) 160.43(7), Cl(1)–W(1)–Cl(2) 172.11(3), Cl(1)–W(1)–N(1) 90.51(7), Cl(2)–W(1)–N(1) 97.31(7), Cl(2)–W(1)–N(2) 178.18(5), N(3)–W(1)–N(5) 168.81(11), N(4)–W(2)–N(6) 174.02 (11); N(3)–W(1)–N(2)–N(6) 9.13(7).

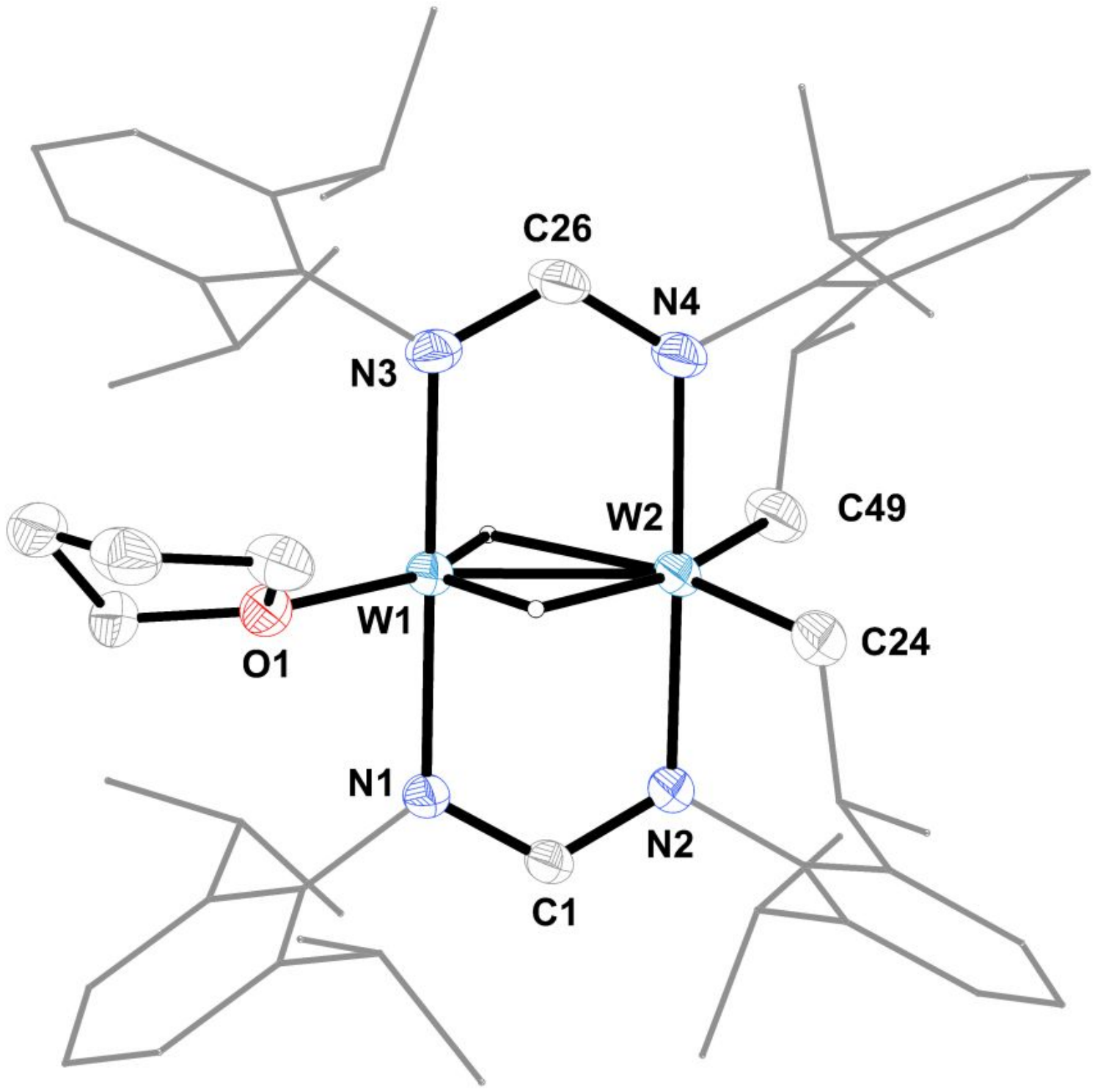

Figure. *S*4 The solid-state molecular structure of **4** drawn with 30% thermal ellipsoids probability level. Hydrogen atoms are omitted for the sake of clarity. Selected bond lengths (Å) and angles (°): W(1)–W(2) 2.3285(3), W(1)–O(1) 2.227(3), W(1)–N(1) 2.124(3), W(1)–N(3) 2.119(4), W(1)–H 1.56, W(1)–HA 1.50, W(2)–N(2) 2.069(4), W(2)–N(4) 2.091(4), W(2)–C(24) 2.183(5), W(2)–C(49) 2.168(5), W(2)–H 1.95, W(2)–HA 2.12, N(1)–C(1) 1.329(6), N(2)–C(1) 1.327(6), N(3)–C(26) 1.345(6), N(4)–C(26) 1.330(6), C(23)–C(24) 1.548(7), C(48)–C(49) 1.562(7); W(1)–W(2)–H 41.6, W(1)–W(2)–HA 38.9, W(1)–W(2)–C(24) 121.83(15), W(1)–W(2)–C(49) 120.62(16), W(1)–H–W(2) 82.3, W(1)–HA–W(2) 77.9, W(2)–W(1)–O(1) 139.08(9), W(2)–W(1)–H 56.0, W(2)–W(1)–HA 63.1, W(2)–C(24)–C(23) 116.6(3), W(2)–C(49)–C(48) 116.5(4), N(1)–W(1)–N(3) 179.88(17), N(2)–W(2)–N(4) 178.62(17); N(1)–W(1)–W(2)–N(4) 0.558.

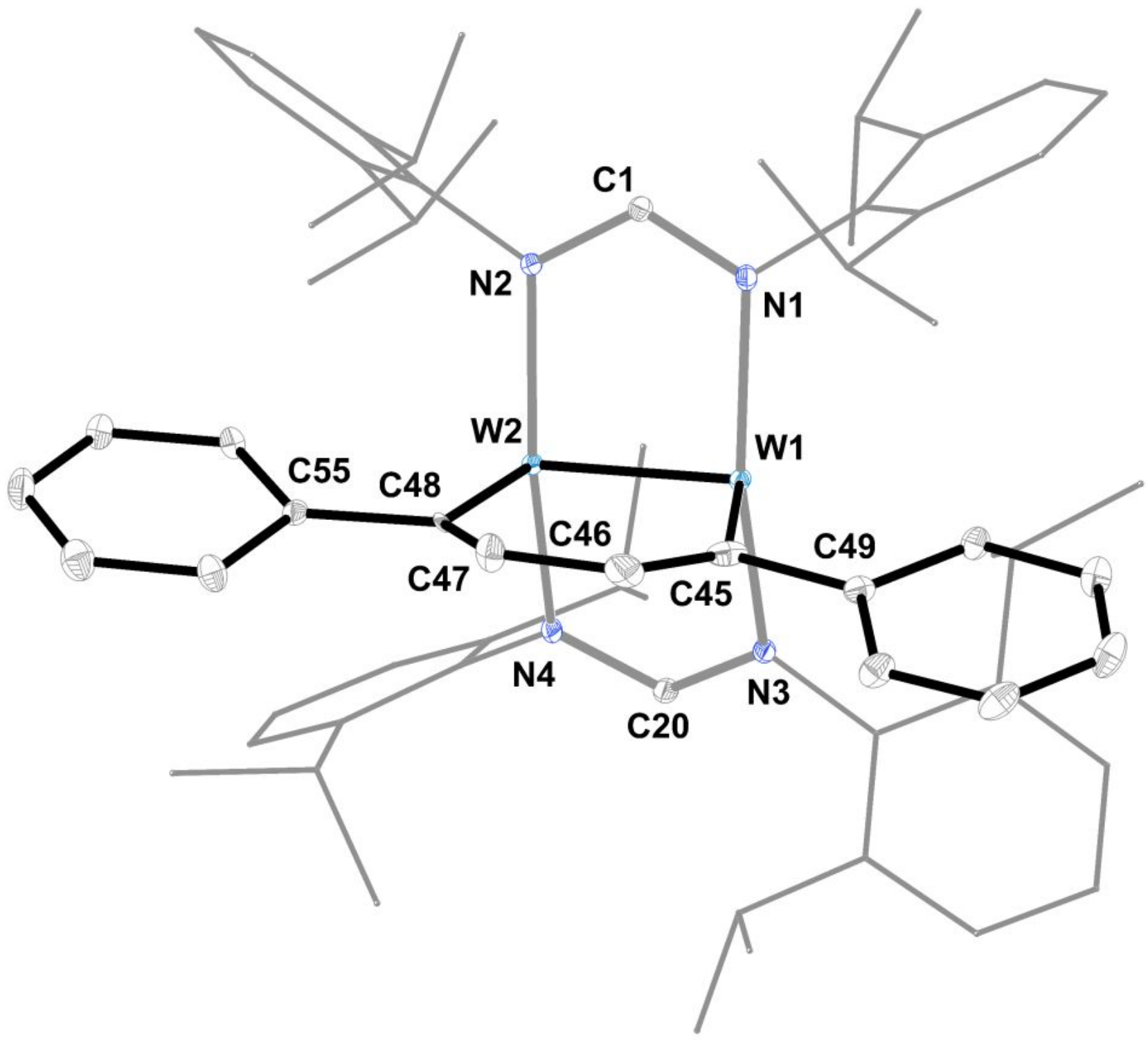

Figure. *S*5 The solid-state molecular structure of **5A** drawn with 30% thermal ellipsoids probability level. Hydrogen atoms are omitted for the sake of clarity. Selected bond lengths (Å) and angles (°): W(1)–W(2) 2.2377(3), W(1)–N(1) 2.136(4), W(1)–N(3) 2.106(4), W(1)–C(45) 1.995(5), W(2)–N(2) 2.105(4), W(2)–N(4) 2.135(4), W(2)–C(48) 2.033(4), N(1)–C(1) 1.325(6), N(2)–C(1) 1.334(6), N(3)–C(20) 1.337(6), N(4)–C(20) 1.331(6), C(45)–C(46) 1.336(8), C(45)–C(49) 1.512(8), C(46)–C(47) 1.425(8), C(47)–C(48) 1.365(7), C(48)–C(55) 1.522(6); W(1)–W(2)–C(48) 100.87(19), W(1)–W(2)–N(2) 91.20(13), W(1)–W(2)–N(4) 90.75(3),W(1)–C(45)–C(46) 130.7(5), W(2)–W(1)–C(45) 102.35(17), W(2)–W(1)–N(1), 91.02(13), W(2)–W(1)–N(3) 91.57(13), W(2)–C(48)–C(47) 131.0(4), C(45)–C(46)–C(47) 128.8(7), C(46)–C(47)–C(48) 125.5(5); C(45)–W(1)–W(2)–C(48) 3.7, W(1)–W(2)–C(48)–C(47) 5.0, W(2)–W(1)–C(45)–C(46) 10.3, C(45)–C(46)–C(47)–C(48) 4.8

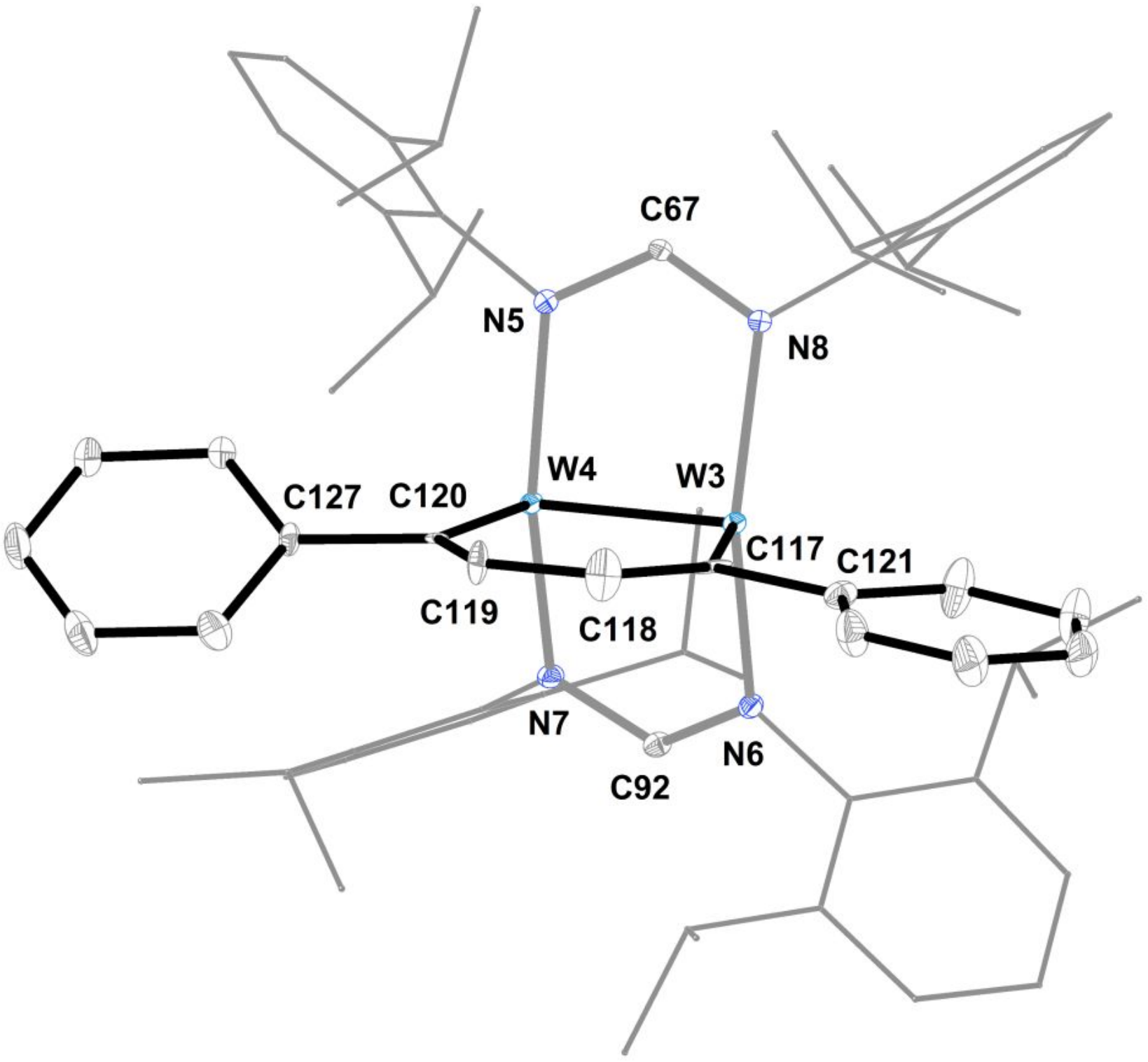

Figure. *S*6 The solid-state molecular structure of **5B** drawn with 30% thermal ellipsoids probability level. Hydrogen atoms are omitted for the sake of clarity. Selected bond lengths (Å) and angles (°): W(3)–W(4) 2.2404(3), W(3)–N(6) 2.092(4), W(3)–N(8) 2.115(4), W(3)–C(117) 2.006(5), W(4)–N(5) 2.099(4), W(4)–N(7) 2.140(4), W(4)–C(120) 1.989(4), N(5)–C(67) 1.330(6), N(6)–C(92) 1.334(6), N(7)–C(92) 1.328(6), N(8)–C(67) 1.323(6), C(117)–C(118) 1.361(8), C(117)–C(121) 1.530(8), C(118)–C(119) 1.383(8), C(119)–C(120) 1.397(7), C(120)–C(127) 1.536(6); W(3)–W(4)–C(120) 99.87(14), W(3)–C(117)–C(118) 131.9(5), W(4)–W(3)–C(117) 102.58(17), W(4)–C(120)–C(119) 132.7(4), N(5)–W(4)–N(7) 148.17(15), N(5)–W(4)–C(120) 100.75(16), N(6)–W(3)–N(8) 152.72(15), N(6)–W(3)–C(117) 102.01(17), C(117)–C(118)–C(119) 125.6(5), C(118)–C(119)–C(120) 127.2(5); W(3)–W(4)–C(120)–C(119) 2.888, W(3)–C(117)–C(118)–C(119) 5.157, W(3)–C(117)–C(121)–C(122) 2.636, W(4)–W(3)–C(117)–C(118) 4.259, W(4)–C(120)–C(119)–C(118) 3.529, W(4)–C(120)–C(127)–C(132) 39.144, N(5)–W(4)–W(3)–N(6) 24.142, N(7)–W(4)–W(3)–N(8) 34.856, C(117)–W(3)–W(4)–C(120) 0.423, C(117)–C(118)–C(119)–C(120) 0.859.

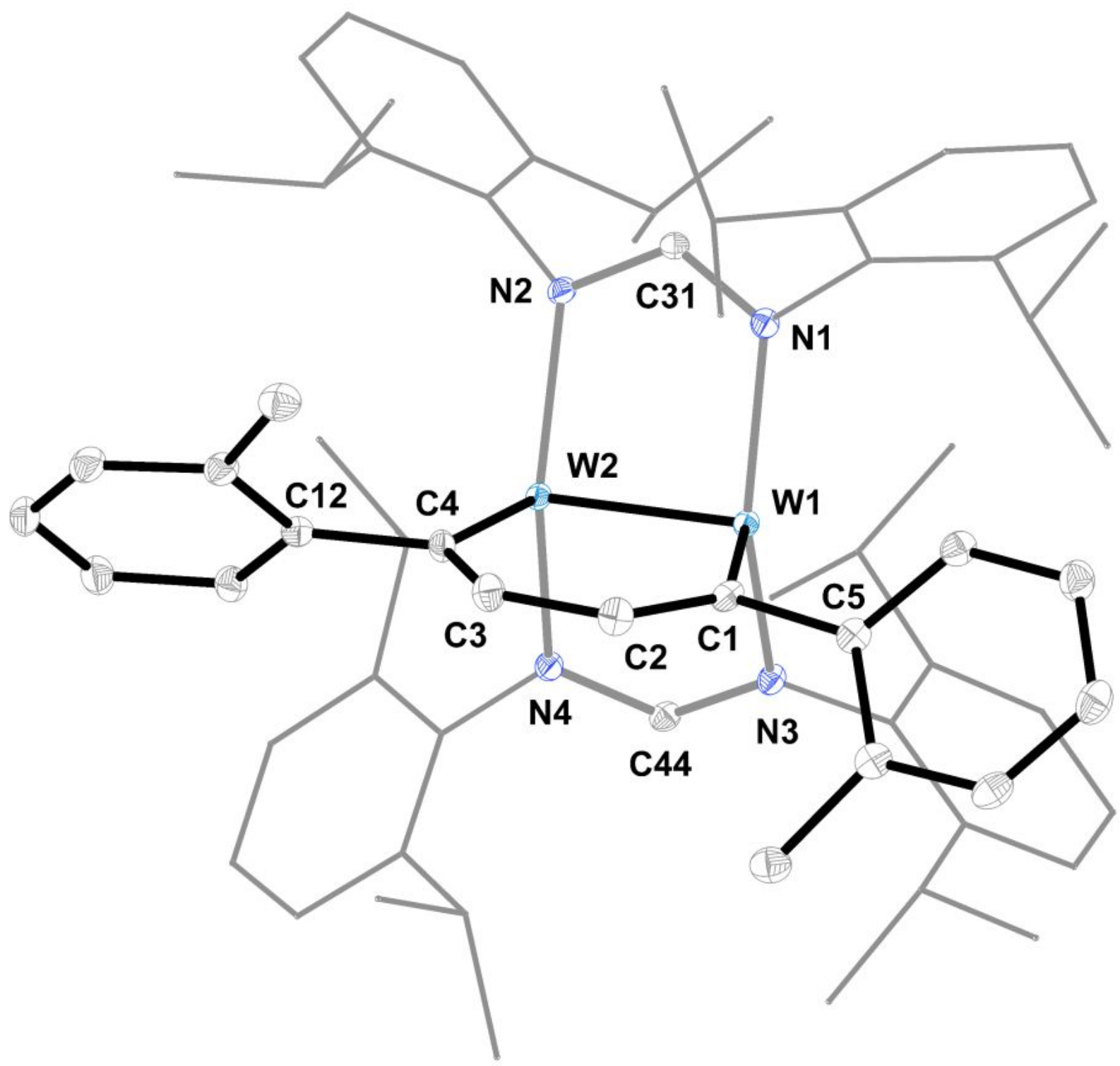

Figure. *S*7 The solid-state molecular structure of **6** drawn with 30% thermal ellipsoids probability level. Hydrogen atoms are omitted for the sake of clarity. Selected bond lengths (Å) and angles (°): W(1)–W(2) 2.25100(12), W(1)–N(1) 2.0935(17), W(1)–N(3) 2.1223(17), W(1)–C(1) 2.003(2), W(2)–N(2) 2.1400(17), W(2)–N(4) 2.091(17), W(2)–C(4) 1.999(2), N(1)–C(31) 1.333(3), N(2)–C(31) 1.332(3), N(3)–C(44) 1.338(3), N(4)–C(44) 1.332(3), C(1)–C(2) 1.408(3), C(1)–C(5) 1.496(3), C(2)–C(3) 1.382(3), C(3)–C(4) 1.413(3), C(4)–C(12) 1.501(3); W(1)–W(2)–C(4) 102.29(6), W(1)–C(1)–C(2) 130.29(15), W(2)–W(1)–C(1) 102.16(6), W(2)–C(4)–C(3) 129.99(15), N(1)–W(1)–N(3) 144.86(7), N(1)–W(1)–C(1) 105.11(7), N(2)–W(2)–N(4) 143.64(7), N(2)–W(2)–C(4) 109.91(8), C(1)–C(2)–C(3) 127.3(2), C(2)–C(3)–C(4) 127.8(2); W(1)–W(2)–C(4)–C(3) 2.795, W(1)–C(1)–C(2)–C(3) 1.288, W(1)–C(1)–C(5)–C(6) 52.906, W(2)–W(1)–C(1)–C(2) 4.461, W(2)–C(4)–C(3)–C(2) 0.772, W(2)–C(4)–C(12)–C(17) 49.269, N(1)–W(1)–W(2)–N(4) 26.997, N(2)–W(2)–W(1)–N(3) 44.431, C(1)–W(1)–W(2)–C(4) 4.043, C(1)–C(2)–C(3)–C(4) 2.190.

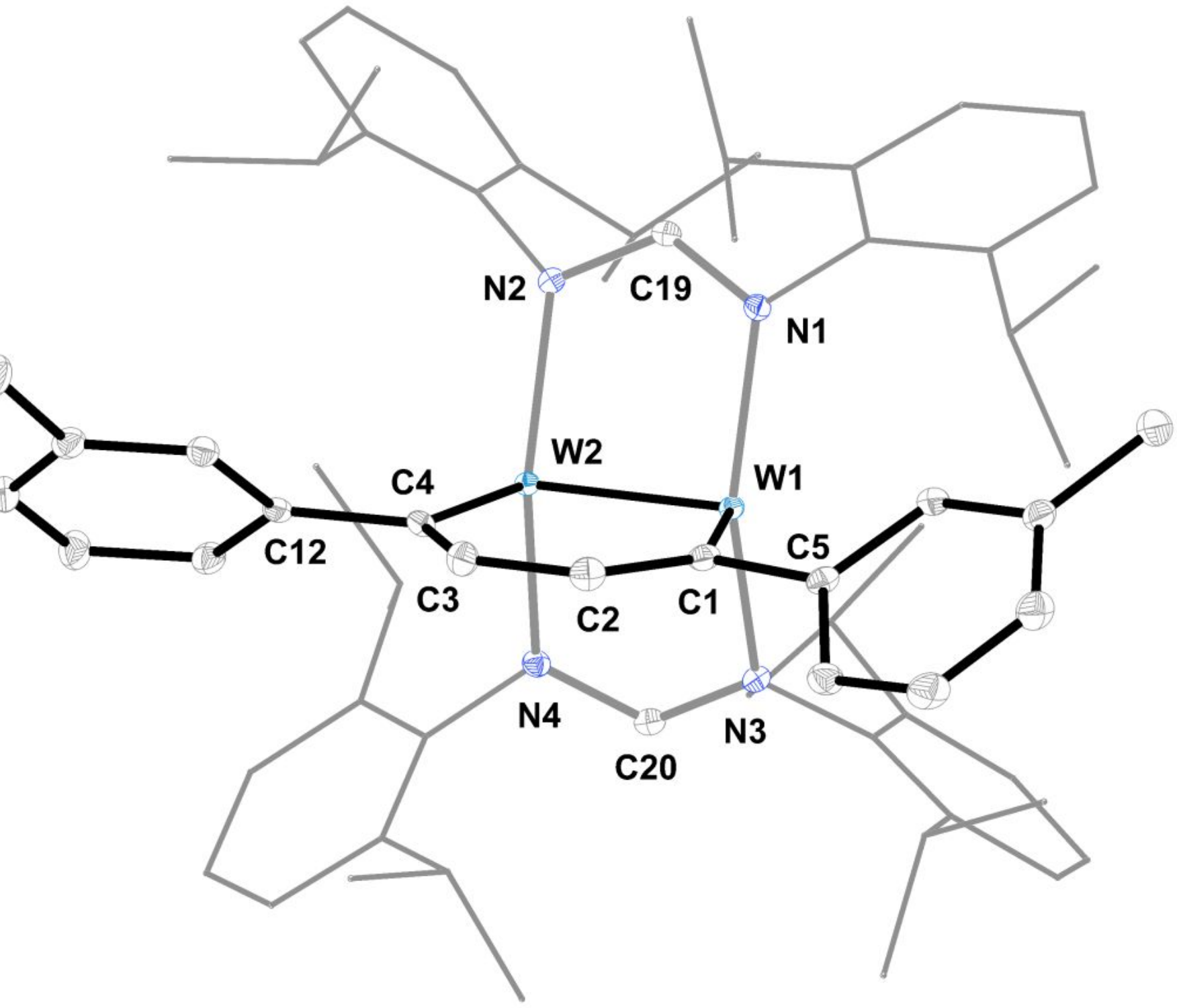

Figure. *S*8 The solid-state molecular structure of **7** drawn with 30% thermal ellipsoids probability level. Hydrogen atoms are omitted for the sake of clarity. Selected bond lengths (Å) and angles (°): W(1)–W(2) 2.24996(13), W(1)–N(1) 2.0888(18), W(1)–N(3) 2.1382(17), W(1)–C(1) 2.012(2), W(2)–N(2) 2.1216(17), W(2)–N(4) 2.0886(17), W(2)–C(4) 2.007(2), N(1)–C(19) 1.333(3), N(2)–C(19) 1.336(3), N(3)–C(20) 1.338(3), N(4)–C(20) 1.324(3), C(1)–C(2) 1.411(3), C(1)–C(5) 1.499(3), C(2)–C(3) 1.374(3), C(3)–C(4) 1.408(3), C(4)–C(12) 1.497(3); W(1)–W(2)–C(4) 103.23(6), W(2)–C(4)–C(3) 129.09(17), W(2)–W(1)–C(1) 101.77(7), W(1)–C(1)–C(2) 129.42(17), N(1)–W(1)–N(3) 144.27(7), N(1)–W(1)–C(1) 105.07(8), N(2)–W(2)–N(4) 146.64(7), N(2)–W(2)–C(4) 128.7(2), C(2)–C(3)–C(4) 127.8(2), C(1)–C(2)–C(3) 109.72(8); W(1)–C(1)–C(2)–C(3) 0.720, W(2)–C(4)–C(3)–C(2) 0.061, N(2)–W(2)–W(1)–N(3) 40.744, C(1)–W(1)–W(2)–C(4) 0.012, C(1)–C(2)–C(3)–C(4) 0.541, W(1)–Cl(1)–C(5)–C(6) 41.578, W(2)–C(4)–C(12)–C(17) 45.250, N(1)–W(1)–W(2)–N(4) 28.233, W(2)–W(1)–C(1)–C(2) 0.371.

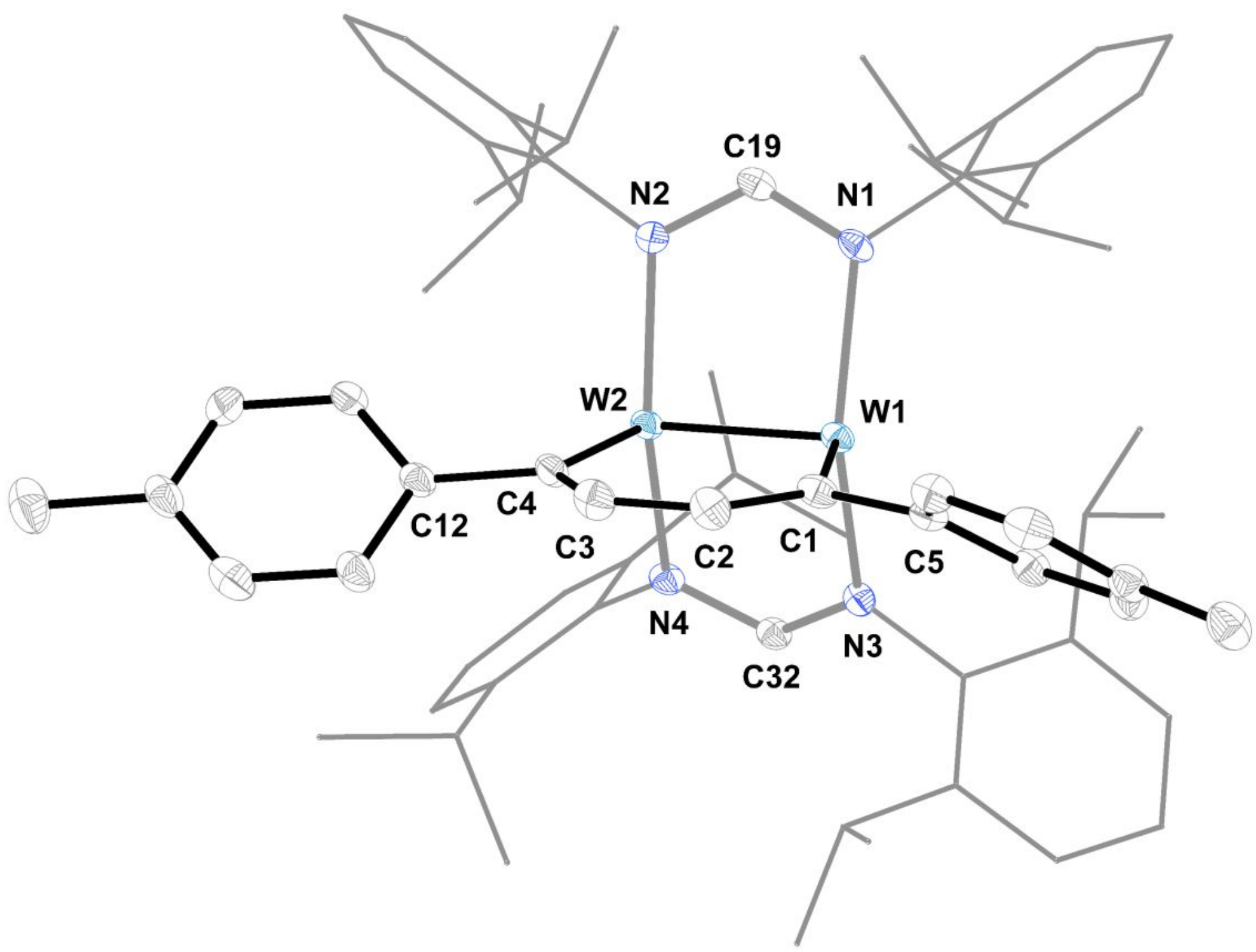

Figure. *S*9 The solid-state molecular structure of **8** drawn with 30% thermal ellipsoids probability level. Hydrogen atoms are omitted for the sake of clarity. Selected bond lengths (Å) and angles (°): W(1)–W(2) 2.2494(3), W(1)–N(1) 2.153(3), W(1)–N(3) 2.095(3), W(1)–C(1) 2.030(4), W(2)–N(2) 2.097(3), W(2)–N(4) 2.103(3), W(2)–C(4) 2.006(4), C(1)–C(2) 1.399(6), N(1)–C(19) 1.336(5), N(2)–C(19) 1.332(5), N(3)–C(32) 1.324(5), N(4)–C(32) 1.343(5), C(1)–C(5) 1.492(6), C(2)–C(3) 1.367(7), C(3)–C(4) 1.405(6), C(4)–C(12) 1.512(6); W(1)–W(2)–C(4) 103.03(13), W(1)–C(1)–C(2) 127.2(3), W(2)–W(1)–C(1) 102.69(13), W(2)–C(4)–C(3) 128.1(3), N(1)–W(1)–N(3) 146.51(12), N(1)–W(1)–C(1) 109.23(14), N(2)–W(2)–N(4) 148.24(13), N(2)–W(2)–C(4) 102.62(15), C(1)–C(2)–C(3) 130.1(4), C(2)–C(3)–C(4) 128.8(4); W(1)–C(1)–C(2)–C(3) 3.613, W(1)–C(1)–C(5)–C(6) 37.319, W(2)–W(1)–C(1)–C(2) 3.278, W(2)–C(4)–C(3)–C(2) 2.932, W(2)–C(4)–C(12)–C(13) 42.414, N(1)–W(1)–W(2)–N(4) 37.715, N(2)–W(2)–W(1)–N(3) 27.401, C(1)–W(1)–W(2)–C(4) 0.704, C(1)–C(2)–C(3)–C(4) 0.079.

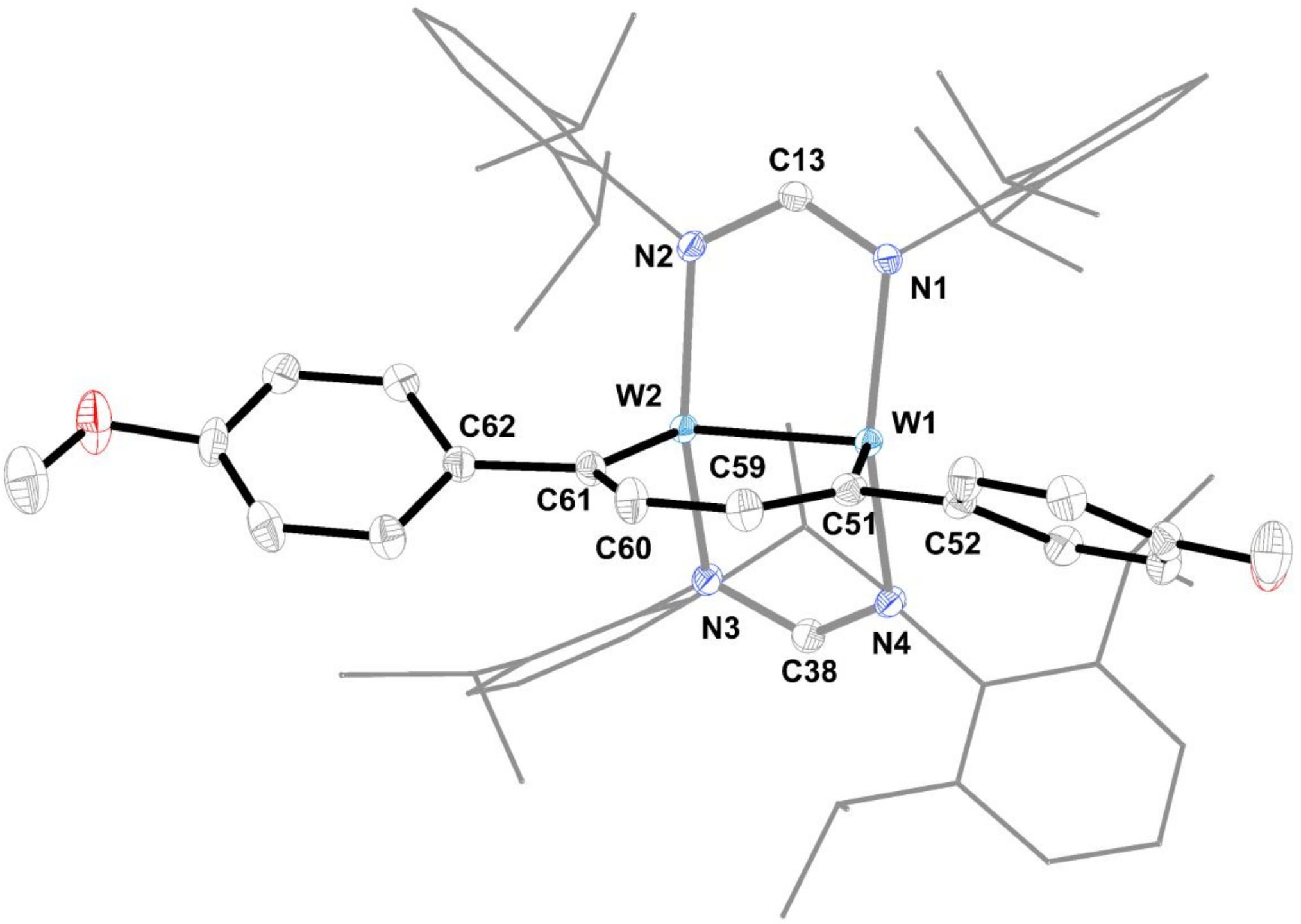

Figure. *S*10 The solid-state molecular structure of **9** drawn with 30% thermal ellipsoids probability level. Hydrogen atoms are omitted for the sake of clarity. Selected bond lengths (Å) and angles (°): W(1)−W(2) 2.2508(2), W(1)–N(1) 2.109(3), W(1)–N(4) 2.097(3), W(1) − C(51) 2.009(4), W(2)–N(2) 2.094(3), W(2)–N(3) 2.153(3), W(2)−C(61) 2.021(4), C(51)−C(59) 1.412(5), C(59)−C(60) 1.373(5), C(60)−C(61) 1.406(5); N(2)−W(2)−N(3) 145.55(12), N(4)−W(2)−N(1) 148.23(12), C(60)−C(59)−C(51) 128.8(4), C(61) − C(60) − C(59) 128.7(4); W(1)−W(2) − C(61) − C(60) 1.875, W(1)−C(51) − C(59) − C(60) 1.494, W(2)−W(1) − C(51) − C(59) 0.611, W(2)−C(51) − C(59) − C(60) 1.625, N(1)−W(1) − W(2) − N(3) 38.842, C(51)−W(1)−W(2)−C(61) 0.704, C(51)−C(59)−C(60)−C(61) 0.356.

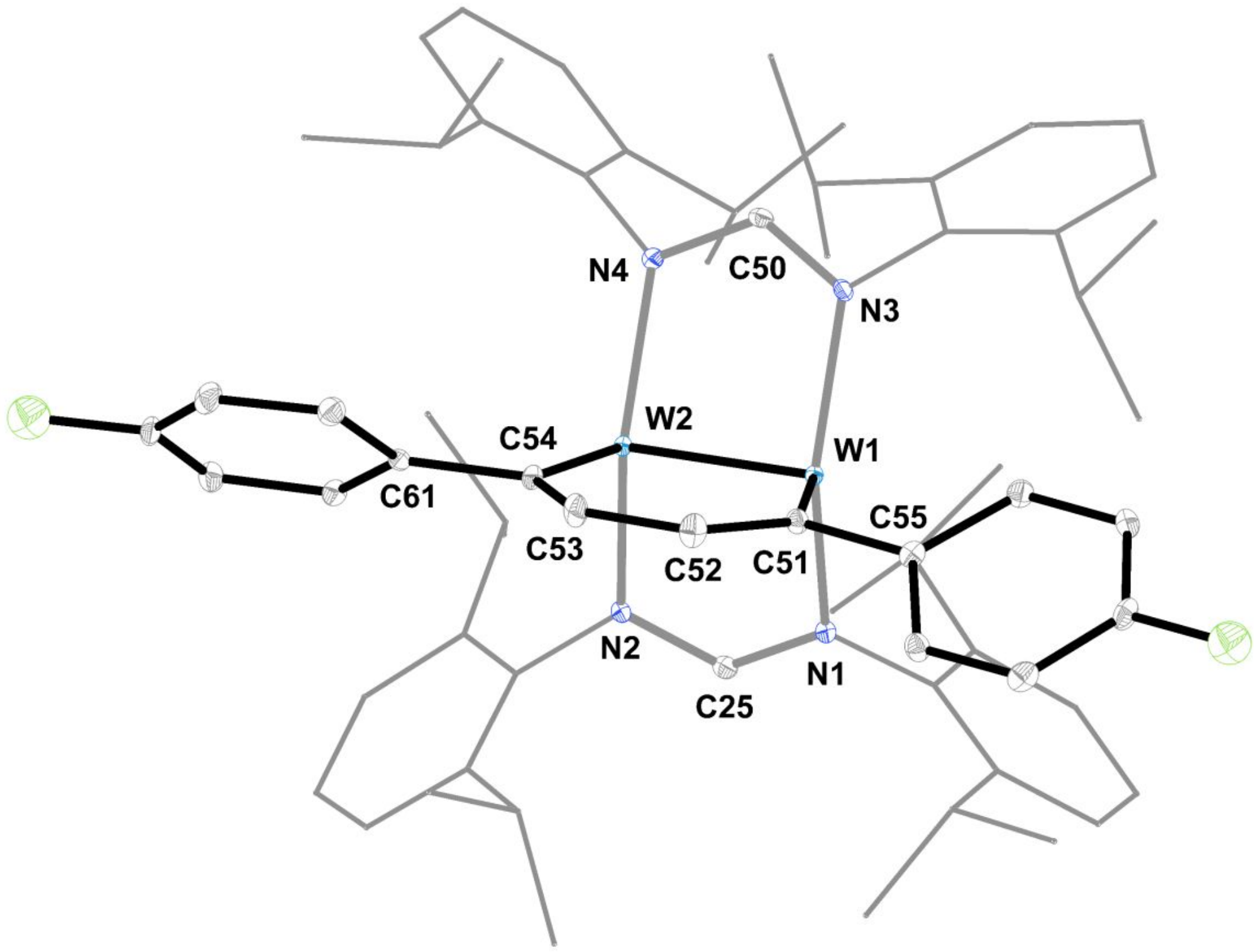

Figure. *S*11 The solid-state molecular structure of **10** drawn with 30% thermal ellipsoids probability level. Hydrogen atoms are omitted for the sake of clarity. Selected bond lengths (Å) and angles (°): W(1)−W(2) 2.24452(15), W(1)−N(1), 2.1161(15), W(1)−N(3), 2.0888(16), W(1)−C(51) 2.0026(19), W(2)−N(2), 2.0920(15), W(2)−N(4), 2.0888 (16), W(2)−C(54) 2.001(2), C(51)−C(52) 1.408(3), C(52)−C(53) 1.378(3), C(53)−C(54) 1.412(3); N(3)−W(1)−N(1) 145.76(6) N(2)−W(2)−N(4) 146.08(6) C(53)−C(52)−C(51) 128.09(19) C(54)−C(53)−C(52) 127.7(2); W(1)−W(2)−C(54)−C(53) 0.402, W(1)−C(51)−C(52)−C(53) 0.461, W(2)−W(1)−C(51)−C(52) 1.517, W(2)−C(54)−C(53)−C(52) 0.917, N(1)−W(1)−W(2)−N(4) 41.209, C(51)−W(1)−W(2)−C(54) 1.182, C(51)−C(59)−C(53)−C(54) 1.046.

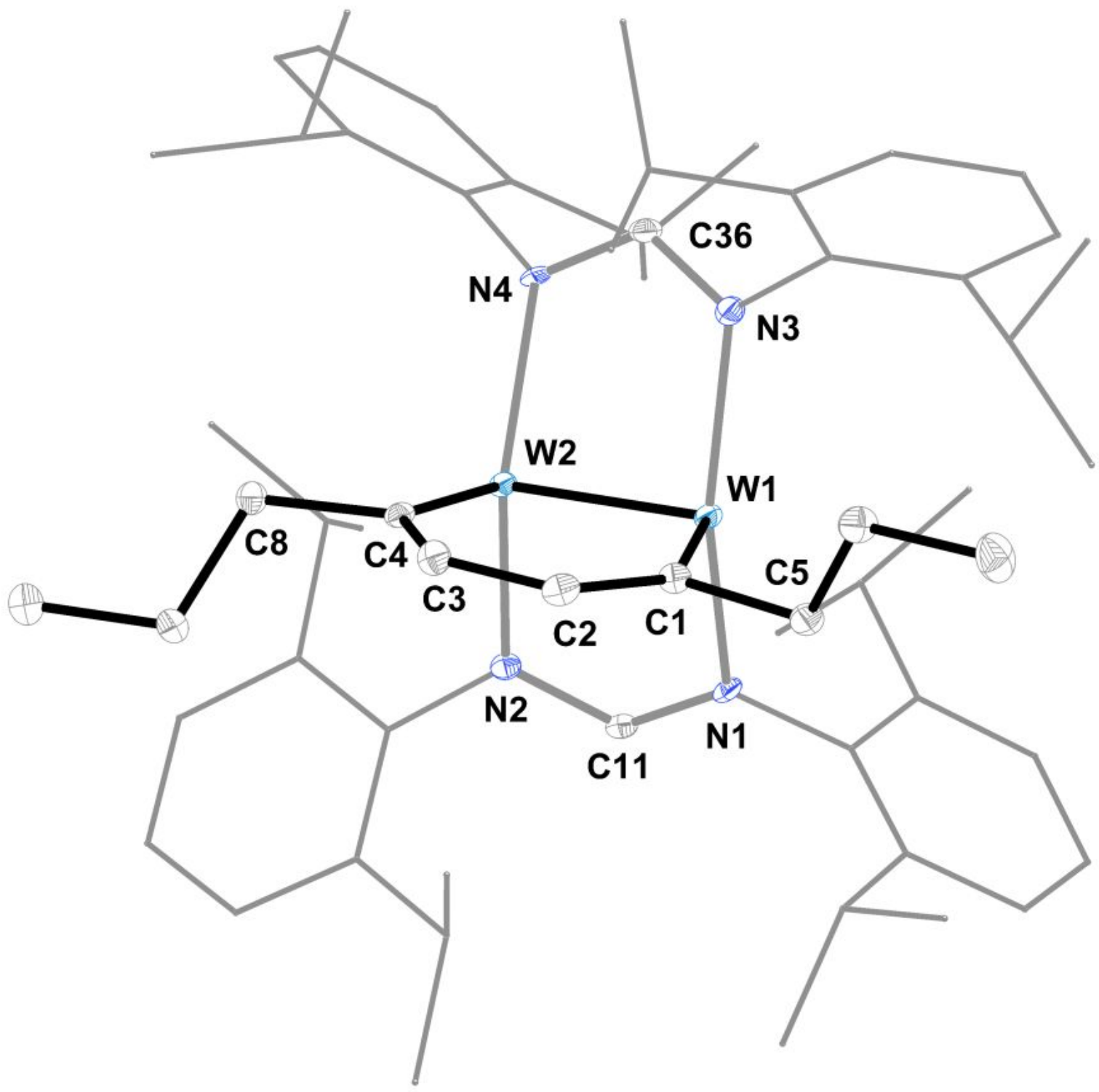

Figure. *S*12 The solid-state molecular structure of **11** drawn with 30% thermal ellipsoids probability level. Hydrogen atoms are omitted for the sake of clarity. Selected bond lengths (Å) and angles (°): W(1)–W(2) 2.2385(3), W(1)–N(1) 2.130(4), W(1)–N(3) 2.114(4), W(1)–C(1) 1.974(4), W(2)–N(2) 2.090(4), W(2)–N(4) 2.144(3), W(2)–C(4) 1.983(4), N(1)–C(11) 1.333(6), N(2)–C(11) 1.334(6), N(3)–C(36) 1.329(6), N(4)–C(36) 1.328(6), C(1)–C(2) 1.395(6), C(1)–C(5) 1.538(6), C(2)–C(3) 1.388(7), C(3)–C(4) 1.407(6), C(4)–C(8) 1.534(6); W(1)–W(2)–C(4) 100.94(13), W(1)–C(1)–C(2) 133.3(3), W(2)–W(1)–C(1) 101.37(13), W(2)–C(4)–C(3) 133.1(3), N(1)–W(1)–N(3) 150.84(15), N(1)–W(1)–C(1) 105.91(16), N(2)–W(2)–N(4) 146.48(14), N(2)–W(2)–C(4) 105.14(15), C(1)–C(2)–C(3) 125.7(4), C(2)–C(3)–C(4) 125.1(4); W(1)–W(2)–C(4)–C(3) 5.939, W(1)–C(1)–C(2)–C(3) 0.627, W(2)–W(1)–C(1)–C(2) 5.946, W(2)–C(4)–C(3)–C(2) 0.670, N(1)–W(1)–W(2)–N(4) 40.674, N(2)–W(2)–W(1)–N(3) 21.934, C(1)–W(1)–W(2)–C(4) 6.323, C(1)–C(2)–C(3)–C(4) 3.242.

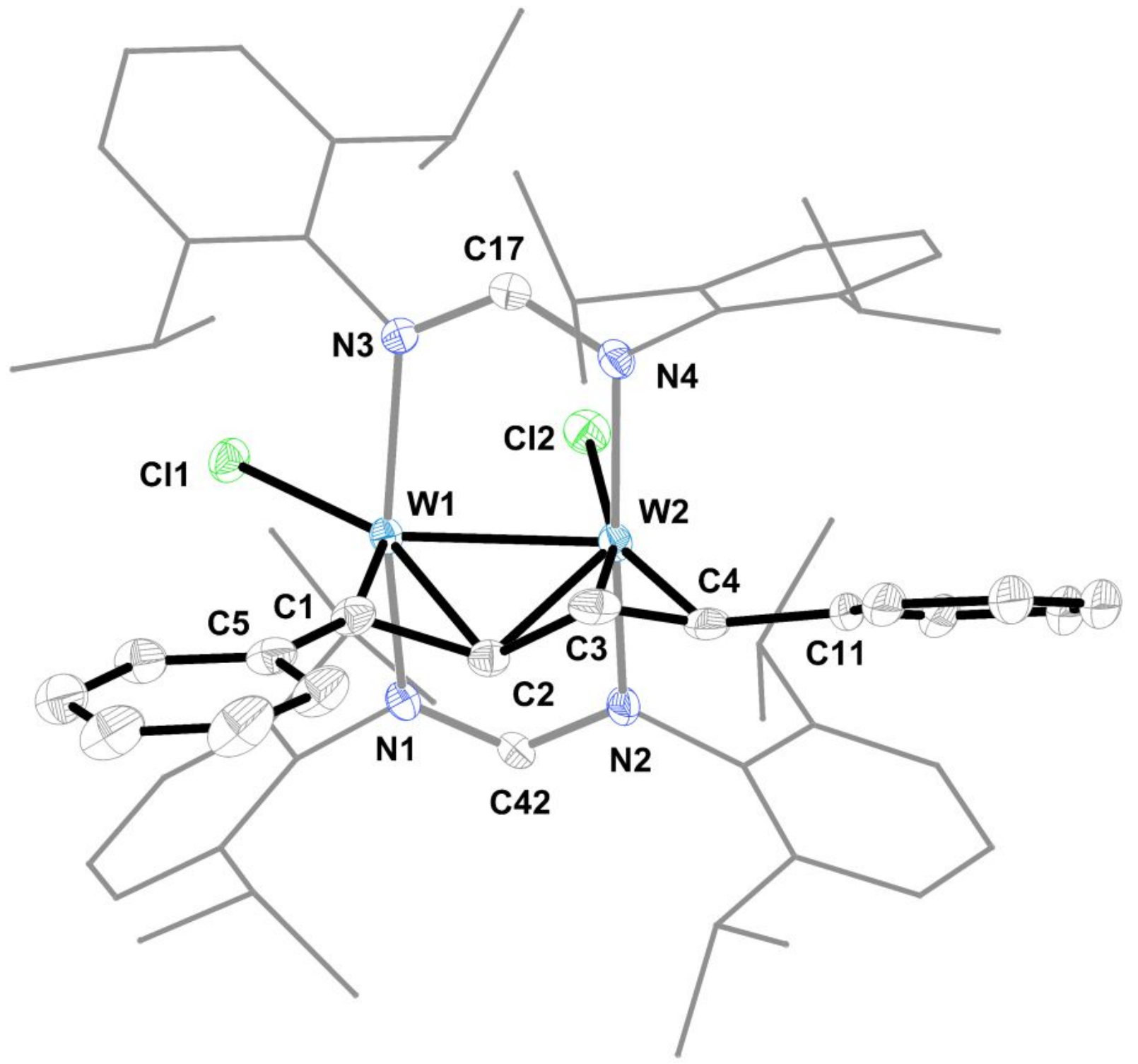

Figure. *S*13 The solid-state molecular structure of **12** drawn with 30% thermal ellipsoids probability level. Hydrogen atoms are omitted for the sake of clarity. Selected bond lengths (Å) and angles (°): W(1)–W(2) 2.4281(4), W(1)–C(1) 1.892(6), W(1)–C(2) 2.273(6), W(2)–C(2) 2.2696(6), W(2)–C(3) 2.2976(6), W(2)–C(4) 2.049(8), W(2)–N(2) 2.129(5), W(2)–N(4) 2.139(5), N(1)–C(42) 1.335(7), N(2)–C(42) 1.329(7), N(3)–C(17) 1.325(7), N(4)–C(17) 1.335(8), C(1)–C(2) 1.504(8), C(1)–C(5) 1.449(9), C(2)–C(3) 1.497(8), C(3)–C(4) 1.344(10), C(4)–C(11) 1.521(10); W(1)–W(2)–Cl(2) 78.59(4), W(1)–C(2)–C(3) 55.8(3), W(2)–C(2)–C(3) 71.9(3), W(2)–C(2)–C(1) 117.1(4), W(2)–W(1)–Cl(1) 139.16(4), W(1)–C(1)–C(2) 83.24(4), W(1)–C(1)–Cl(1) 121.2(4), W(2)–C(4)–C(3) 82.4(4), N(1)–W(1)–N(3) 158.29(17), N(1)–W(1)–Cl(1) 87.75(13), N(1)–W(1)–C(1) 108.3(2), N(2)–W(2)–N(4) 169.25(19), N(2)–W(2)–Cl(2) 84.50(14), N(2)–W(2)–C(3) 108.3(2), C(1)–C(2)–C(3) 124.05(5), C(2)–C(3)–C(4) 111.6(6); N(1)–W(1)–W(2)–N(4) 11.804, N(2)–W(2)–W(1)–N(3) 19.457.

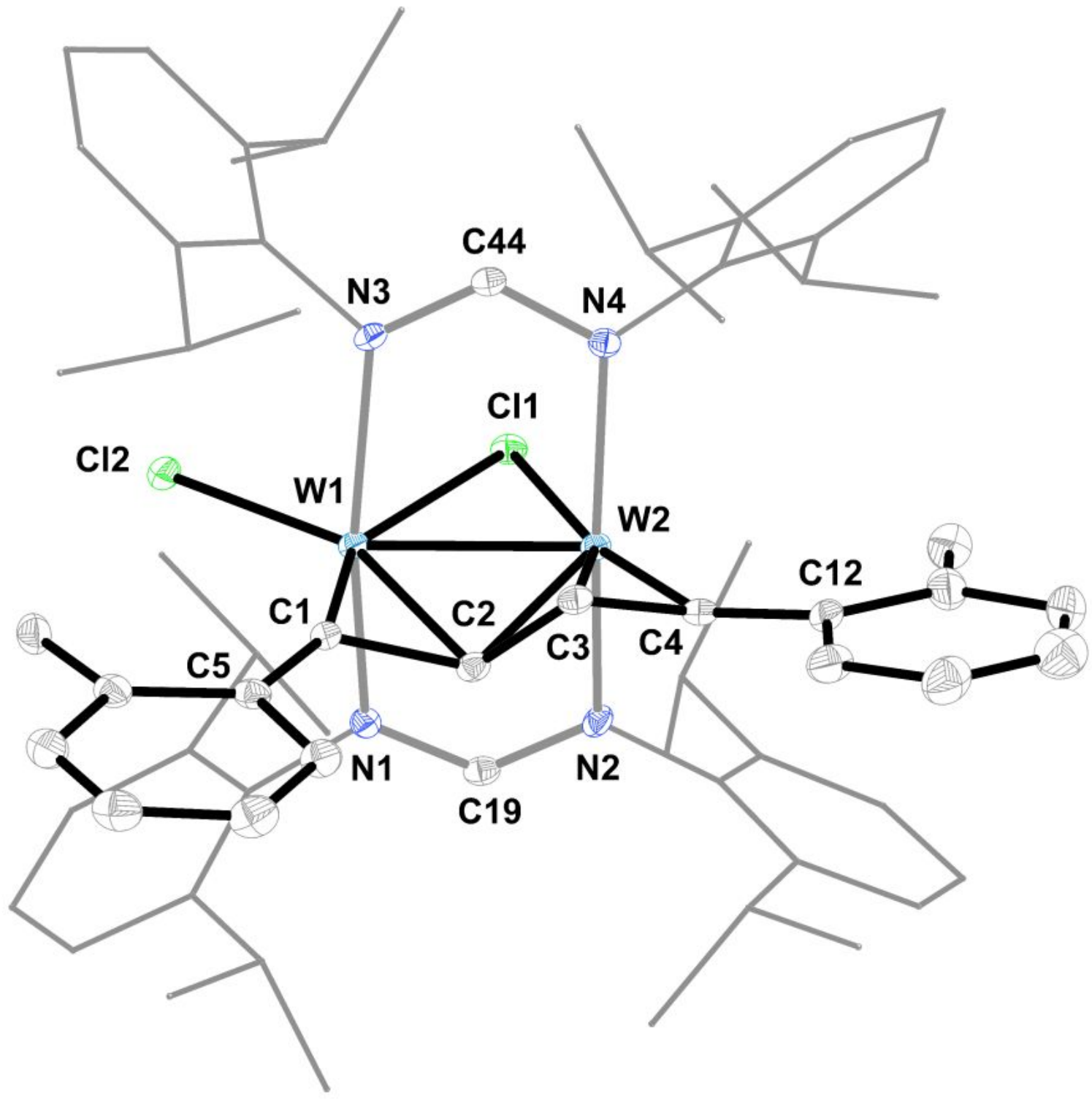

Figure. *S*14 The solid-state molecular structure of **13** drawn with 30% thermal ellipsoids probability level. Hydrogen atoms are omitted for the sake of clarity. Selected bond lengths (Å) and angles (°): W(1)–W(2) 2.44705(16), W(1)–Cl(1) 2.7742(6), W(1)–Cl(2) 2.3588(6), W(1)–N(1) 2.191(2), W(1)–N(3) 2.214(2), W(1)–C(1) 1.879(3), W(1)–C(2) 2.253(3), W(2)–Cl(1) 2.4628(6), W(2)–N(2) 2.131(2), W(2)–N(4) 2.140(2), W(2)–C(2) 2.252(3), W(2)–C(3) 2.284(6), W(2)–C(4) 2.059(3), N(1)–C(19) 1.316(3), N(2)–C(19) 1.340(3), N(3)–C(44) 1.320(3), N(4)–C(44) 1.334(3), C(1)–C(2) 1.533(4), C(1)–C(5) 1.463(4), C(2)–C(3) 1.456(4), C(3)–C(4) 1.389(4), C(4)–C(12) 1.443(4); W(1)–W(2)–Cl(1) 68.809(15), W(1)–C(1)–C(2) 81.96(15), W(1)–C(2)–W(2) 65.81(7), W(1)–C(2)–C(1) 55.69(13), W(1)–C(2)–C(3) 117.78(18), W(2)–W(1)–Cl(2) 144.477(17), W(2)–C(2)–C(1) 119.53(17), W(2)–C(2)–C(3) 72.47(15), W(2)–C(4)–C(3) 80.39(16), N(1)–W(1)–N(3) 160.95(8), N(1)–W(1)–Cl(2) 90.72(6), N(1)–W(1)–C(1) 95.82(9), N(2)–W(2)–N(4) 161.55(8), N(2)–W(2)–Cl(1) 81.94(6), N(2)–W(2)–C(3) 110.39(9), C(1)–C(2)–C(3) 122.0(2), C(2)–C(3)–C(4) 112.4(2); N(1)–W(1)–W(2)–N(4) 19.020, N(2)–W(2)–W(1)–N(3) 16.689.

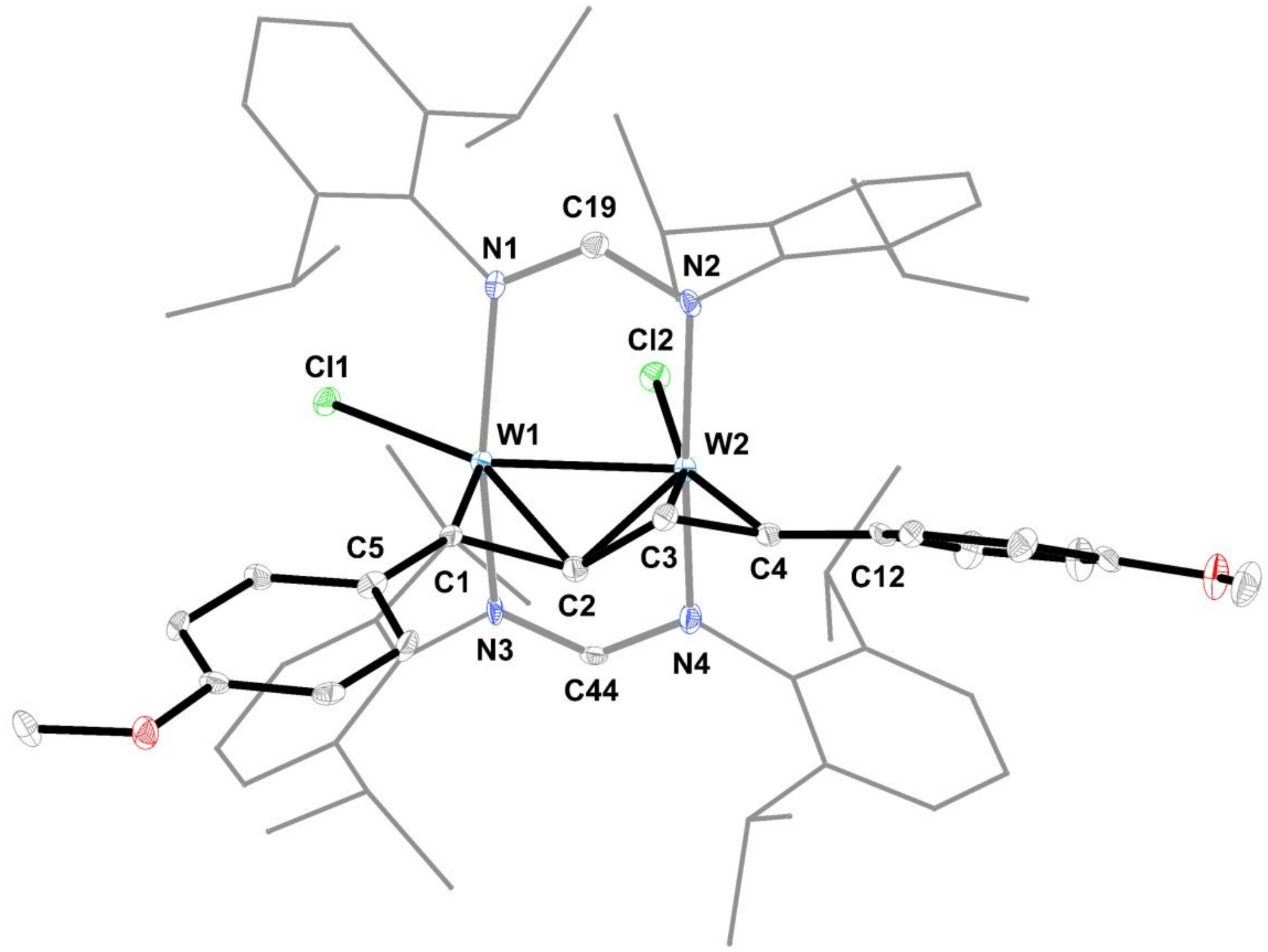

Figure. *S*15 The solid-state molecular structure of **14** drawn with 30% thermal ellipsoids probability level. Hydrogen atoms are omitted for the sake of clarity. Selected bond lengths (Å) and angles (°): W(1)−W(2) 2.4311(4), W(1)−Cl(1) 2.3726(14), W(2)−Cl(2) 2.4025(15), W(1)−C(1) 1.881(6), W(1)−C(2) 2.289(6), W(2)−C(2) 2.264(6), W(2)−C(3) 2.291(6), W(2)−C(4) 2.050(6), C(1)−C(2) 1.533(8), C(1)−C(5) 1.462(8), C(2)−C(3) 1.475(9), C(3)−C(4) 1.385(9), C(4)−C(12) 1.427(11), W(1)−N(1) 2.203(5), W(1)−N(3) 2.188(5); W(2)−N(2) 2.140(6), W(2)−N(4) 2.144(6), N(1)−C(19) 1.326(8), N(2)−C(19) 1.334(11), N(3)−C(44) 1.326(11), N(4)−C(44) 1.337(5); N(1)−C(19)−N(2) 125.3(6), N(3)−C(44)−N(4) 123.4(6), C(1)−W(1)−C(2) 41.7(3), W(1)−C(2)−W(2) 64.56(18), C(2)−W(2)−C(3) 37.8(2), C(3)−W(2)−C(4) 36.7(3); C(1)−W(1)−W(2)−C(4) 2.6(3), C(1)−W(1)−W(2)−C(3) 13.7(3), C(1)−W(1)−W(2)−C(2) 11.5(3), C(2)−W(1)−W(2)−C(3) 25.2(3), C(2)−W(1)−W(2)−C(4) 14.2(3).

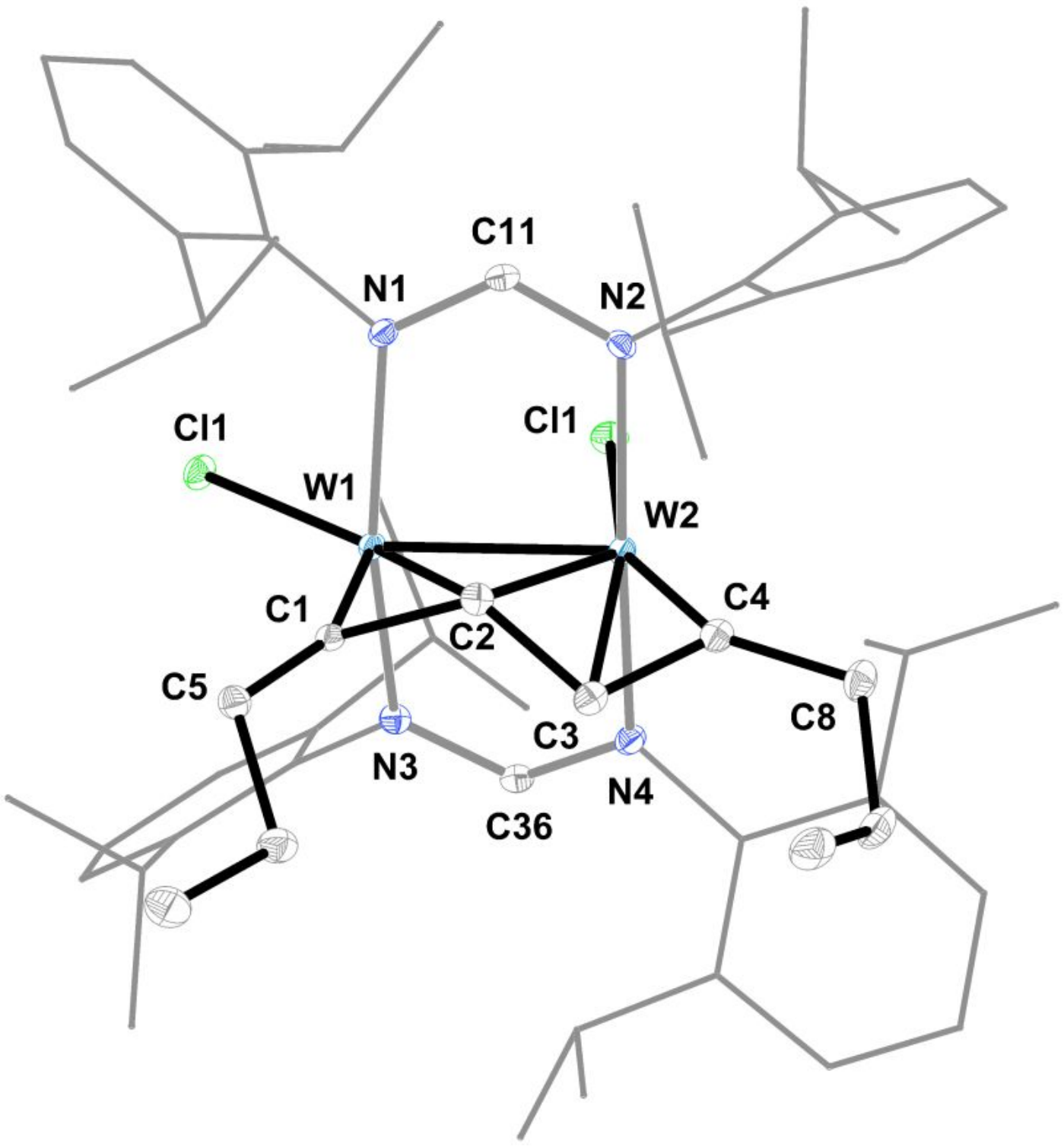

Figure. *S*16 The solid-state molecular structure of **15** drawn with 30% thermal ellipsoids probability level. Hydrogen atoms are omitted for the sake of clarity. Selected bond lengths (Å) and angles (°): W(1)–W(2) 2.43589(13), W(1)–Cl(1) 2.3497(8), W(1)–N(1) 2.1807(17), W(1)–N(3) 2.1957(17), W(1)–C(1) 1.872(2), W(1)–C(2) 2.265(2), W(2)–Cl(2) 2.3865(2), W(2)–N(2) 2.1379(17), W(2)–N(4) 2.1536(18), W(2)–C(2) 2.264(2), W(2)–C(3) 2.296(2), W(2)–C(4) 2.038(2), N(1)–C(11) 1.323(3), N(2)–C(11) 1.323(3), N(3)–C(36) 1.322(3), N(4)–C(36) 1.331(3), C(1)–C(2) 1.511(3), C(1)–C(5) 1.495(3), C(2)–C(3) 1.461(3), C(3)–C(4) 1.372(3), C(4)–C(8) 1.490(3); W(1)–W(2)–Cl(2) 81.458(13), W(1)–C(1)–C(2) 83.35(13), W(1)–C(2)–W(2) 65.06(6), W(1)–C(2)–C(1) 55.17(11), W(1)–C(2)–C(3) 121.51(15), W(2)–W(1)–Cl(1) 140.937(14), W(2)–C(2)–C(1) 117.10(14), W(2)–C(2)–C(3) 72.47(12), W(2)–C(4)–C(3) 82.10(14), N(1)–W(1)–N(3) 156.90(7), N(1)–W(1)–Cl(1) 87.16(5), N(1)–W(1)–C(1) 100.74(8), N(2)–W(2)–N(4) 169.08(7), N(2)–W(2)–Cl(2) 83.73(5), N(2)–W(2)–C(3) 107.66(7), C(1)–C(2)–C(3) 123.90(19), C(2)–C(3)–C(4) 112.2(2); N(1)–W(1)–W(2)–N(4) 13.660, N(2)–W(2)–W(1)–N(3) 19.366.

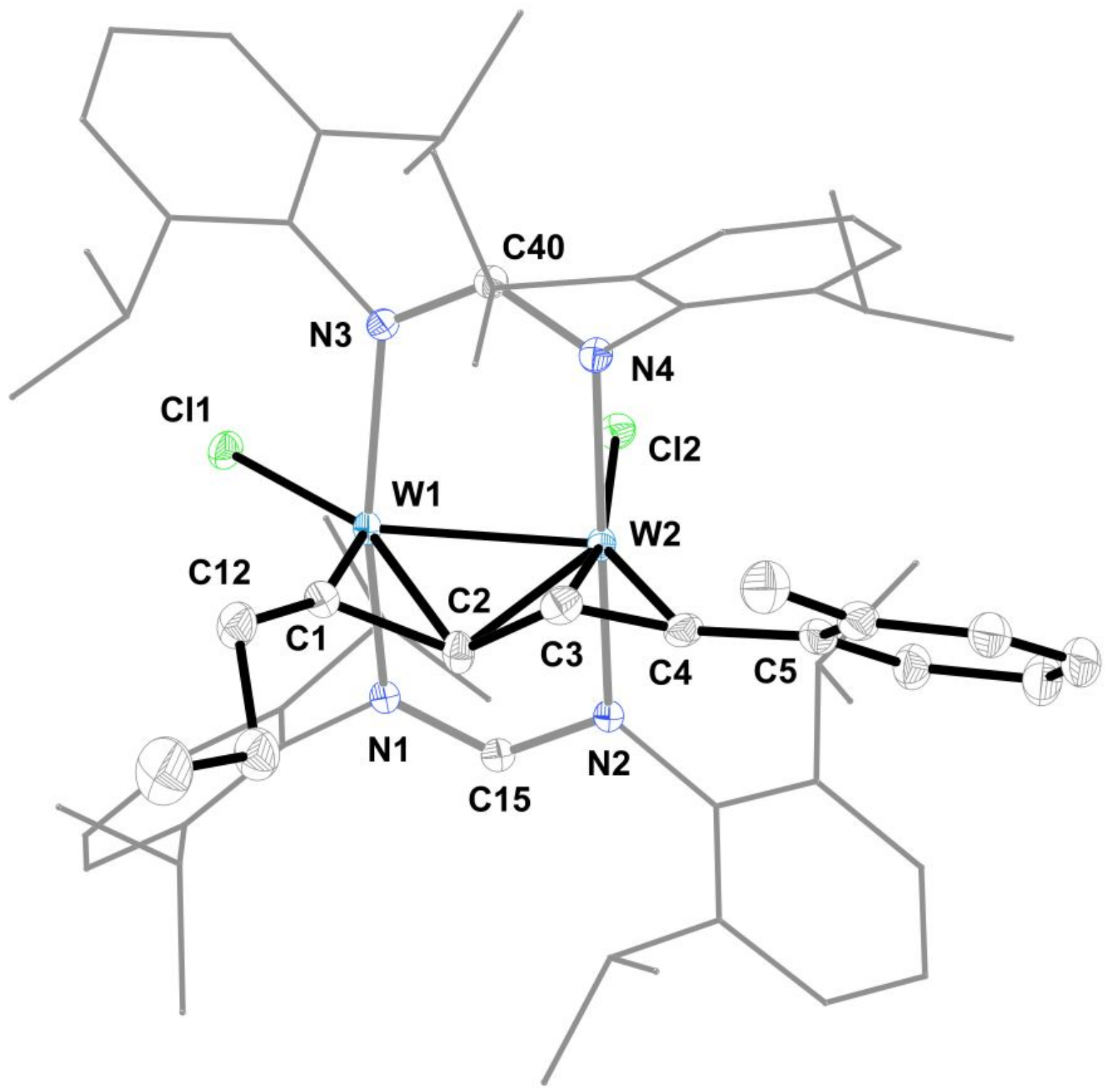

Figure. *S*17 The solid-state molecular structure of **16** drawn with 30% thermal ellipsoids probability level. Hydrogen atoms are omitted for the sake of clarity. Selected bond lengths (Å) and angles (°): W(1)–W(2) 2.4516(3), W(1)–N(1) 2.710(3), W(1)–N(3) 2.173(2), W(1)–Cl(1) 2.3433(6), W(1)–C(1) 1.882(4), W(1)–C(2) 2.270(3), W(2)–N(2) 2.169(3), W(2)–N(4) 2.158(3), W(2)–Cl(2) 2.3575(8), W2–C(2) 2.258(3), W2–C(3) 2.294(3), W2–C(4) 2.063(2), C(1)–C(2) 1.514(4), C(1)–C(12) 1.507(6), C(2)–C(3) 1.468(4), C(3)–C(4) 1.382(6), C(4)–C(5) 1.463(5); W(1)–W(2)–Cl(2) 85.07(2), W(1)–W(2)–C(2) 57.45(6), W(1)–W(2)–C(3) 88.17(9), W(1)–W(2)–C(4) 122.90(12), W(1)–C(2)–C(3) 122.8(2), W(1)–C(2)–C(1) 116.8(2), W(1)–C(1)–C(12) 156.5(2), W(2)–W(1)–Cl(1) 137.40(3), W(2)–W(1)–C(1) 126.86(10). W(2)–W(1)–C(2) 162.32(8), W(2)–C(1)–C(1) 116.8(2), W(2)–C(2)–C(3) 72.54(19), W(2)–C(3)–C(4) 62.62(17), W(2)–C(4)–C(5) 130.8(3), C(1)–C(2)–C(3) 122.5(3), C(1)–C(2)–W(2) 116.8(2), C(2)–C(3)–C(4) 112.3(3), C(2)–C(1)–C(12) 120.4(2), C(3)–C(4)–C(5) 140.0(3); C(1)–W(1)–W(2)–C(2) 15.63(13), C(1)–W(1)–W(2)–C(3) 7.47(12), C(1)–W(1)–W(2)–C(4) 4.46(14), C(2)–W(1)–W(2)–C(3) 23.09(12), C(2)–W(1)–W(2)–C(4) 11.17(14), Cl(1)–W(1)–W(2)–Cl(2) 3.65(4).

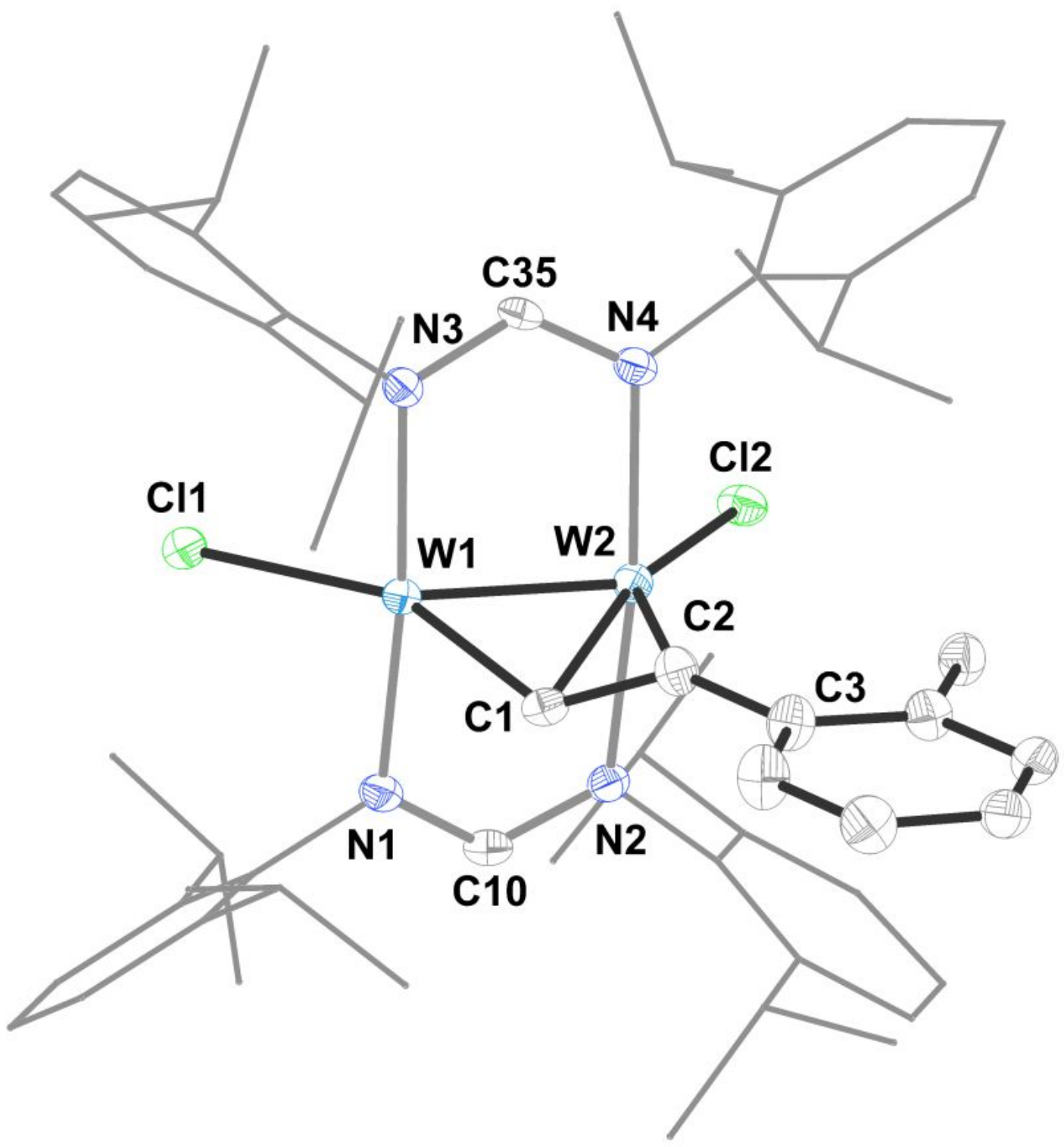

Figure. *S*18 The solid-state molecular structure of **17** drawn with 30% thermal ellipsoids probability level. Hydrogen atoms are omitted for the sake of clarity. Selected bond lengths (Å) and angles (°): W(1)–W(2) 2.3227(6), W(1)–N(1) 2.085(5), W(1)–N(3) 2.102(5), W(1)–C(1) 2.136(9), W(1)–Cl(1) 2.399(1), W(2)–N(2) 2.188(5), W(2)–N(4) 2.173(5), W(2)–C(1) 2.333(9), W(2)–C(2) 2.005(7), W(2)–Cl(2) 2.350(1), C(1)–C(2) 1.340(9), C(2)–C(3) 1.459(9), C(10)–N(1) 1.338(7), C(10)–N(2) 1.320(8), C(35)–N(3) 1.344(8), C(35)–N(4) 1.321(7); W(1)–W(2)–Cl(2) 132.75(4), W(1)–W(2)–C(1) 54.6(2), W(1) W(2)–C(2) 86.2(2), W(1)–C(1)–C(2) 115.2(6), W(2)–W(1)–C(1) 63.0(2), W(2)–W(1)–Cl(1) 134.39(4), W(2)–C(2)–C(3) 142.8(5), C(1)–W(2)–C(2) 35.0(3), C(1)–C(2)–C(3) 128.0(7); W(1)–W(2)–C(2)–C(1) 23.5(5), W(1)–W(2)–C(2)–C(3) 178.1(8), C(1)–W(1)–W(2)–C(2) 16.3(3), Cl(1)–W(1)–W(2)–Cl(2) 3.41(7).

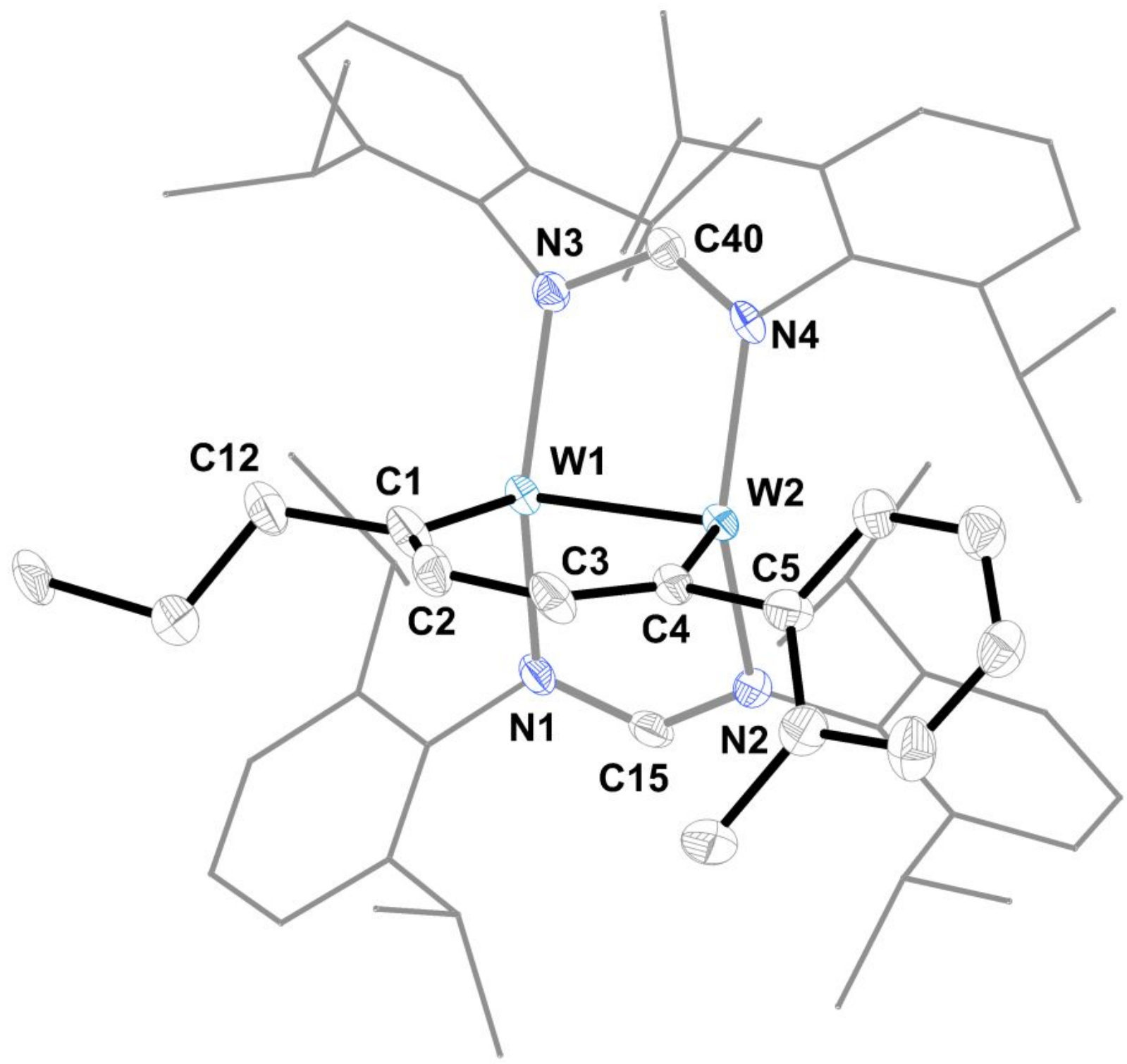

Figure. *S*19 The solid-state molecular structure of **18** drawn with 30% thermal ellipsoids probability level. Hydrogen atoms are omitted for the sake of clarity. Selected bond lengths (Å) and angles (°): W(1)–W(2) 2.2437(5), W(1)–C(1) 1.969(8), W(1)–N(1) 2.096(6), W(1)–N(3) 2.132(6), W(2)–N(2) 2.121(6), W(2)–N(4) 2.094(6), C(1)–C(2) 1.377(12), C(1)–C(12) 1.538(12), C(2)–C(3) 1.385(11), C(3)–C(4) 1.414(11), C(4)–C(5) 1.489(12), C(40)–N(3) 1.336(9), C(40)–N(4) 1.331(9), C(15)–N(1) 1.311(10), C(15)–N(2) 1.345(10); W(1)–W(2)–C(4) 100.3(2), W(1)–C(1)–C(2) 133.4(6), W(1)–C(1)–C(12) 112.5(6), W(2)–W(1)–C(1) 102.5(2), W(2)–C(4)–C(3) 131.2(6), W(2)–C(4)–C(5) 113.2(6), C(1)–C(2)–C(3) 125.5(8), C(2)–C(1)–C(12) 114.0(7), C(2)–C(3)–C(4) 126.8(8), C(3)–C(4)–W(2) 131.2(6), C(3)–C(4)–C(5) 115.4(7); W(1)–W(2)–C(4)–C(3) 4.9(8), W(1)–W(2)–C(4)–C(5) 179.5(5), W(2)–W(1)–C(1)–C(2) 1.8(9), W(2)–W(1)–C(1)–C(12) 174.6(5), C(1)–C(1)–W(2)–W(4) 4.2(3), C(1)–C(2)–C(3)–C(4) 4(1).

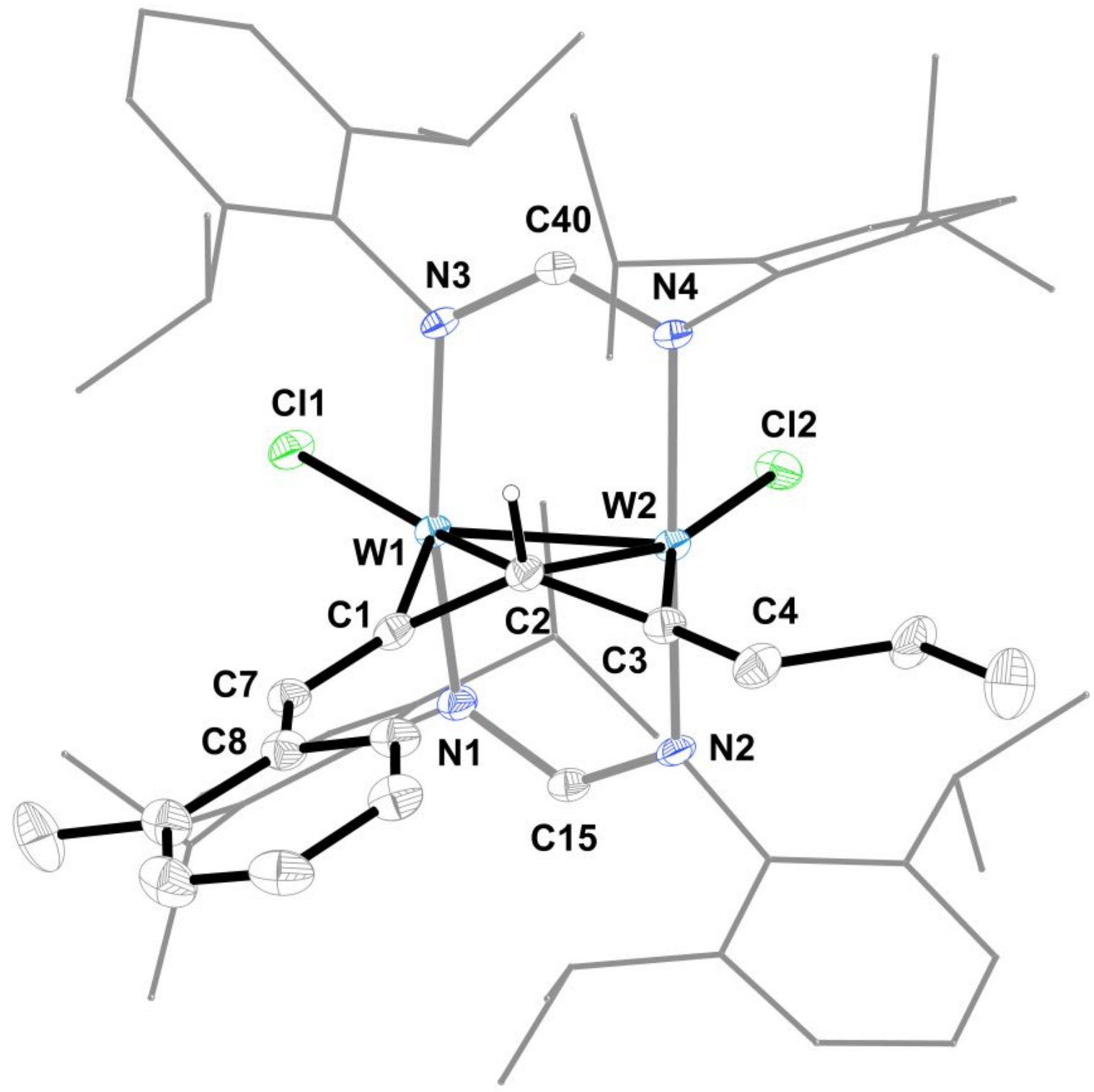

Figure. *S*20 The solid-state molecular structure of **19** drawn with 30% thermal ellipsoids probability level. Hydrogen atoms are omitted for the sake of clarity. Selected bond lengths (Å) and angles (°): W(1)–W(2) 2.3533(5), W(1)–N(1) 2.122(2), W(1)–N(3) 2.100(2), W(1)–Cl(1) 2.3871(9), W(1)–C(1) 2.059(2), W(1)–C(2) 2.426(3), W(2)–N(2) 2.170(2), W(2)–N(4) 2.188(2), W(2)–Cl(2) 2.3836(7), W(2)–C(2) 2.333(3), W(2)–C(3) 1.927(2), C(1)–C(2) 1.458(4), C(1)–C(7) 1.347(3), C(2)–C(3) 1.450(5), C(3)–C(4) 1.498(5); W(1)–W(2)–Cl(2) 105.43(2), W(1)–C(2)–W(2) 59.24(7), W(1)–C(1)–C(2) 85.4(2), W(1)–C(1)–C(7) 138.9(2), W(1)–C(2)–C(1) 57.8(1), W(1)–W(2)–C(2) 62.34(7), W(1)–W(2)–C(3) 98.09(8), W(2)–W(1)–Cl(1) 135.42(2), W(2)–W(1)–C(1) 90.53(8), W(2)–W(1)–C(2) 58.41(7), W(2)–W(1)–C(1) 90.53(8), C(1)–C(2)–C(3) 131.7(3), C(2)–C(1)–C(7) 134.7(3), C(2)–C(3)–C(4) 128.3(3), C(2)–W(1)–C(1) 36.8(1), C(2)–W(2)–C(3) 38.3(1); W(1)–W(2)–C(3)–C(2) 20.6(2), W(1)–W(2)–C(2)–C(1) 29.2(2), W(2)–W(1)–C(1)–C(2) 27.5(2), C(1)–W(1)–W(2)–C(3) 4.7(1), Cl(1)–W(1)–W(2)–Cl(2) 4.95(4).

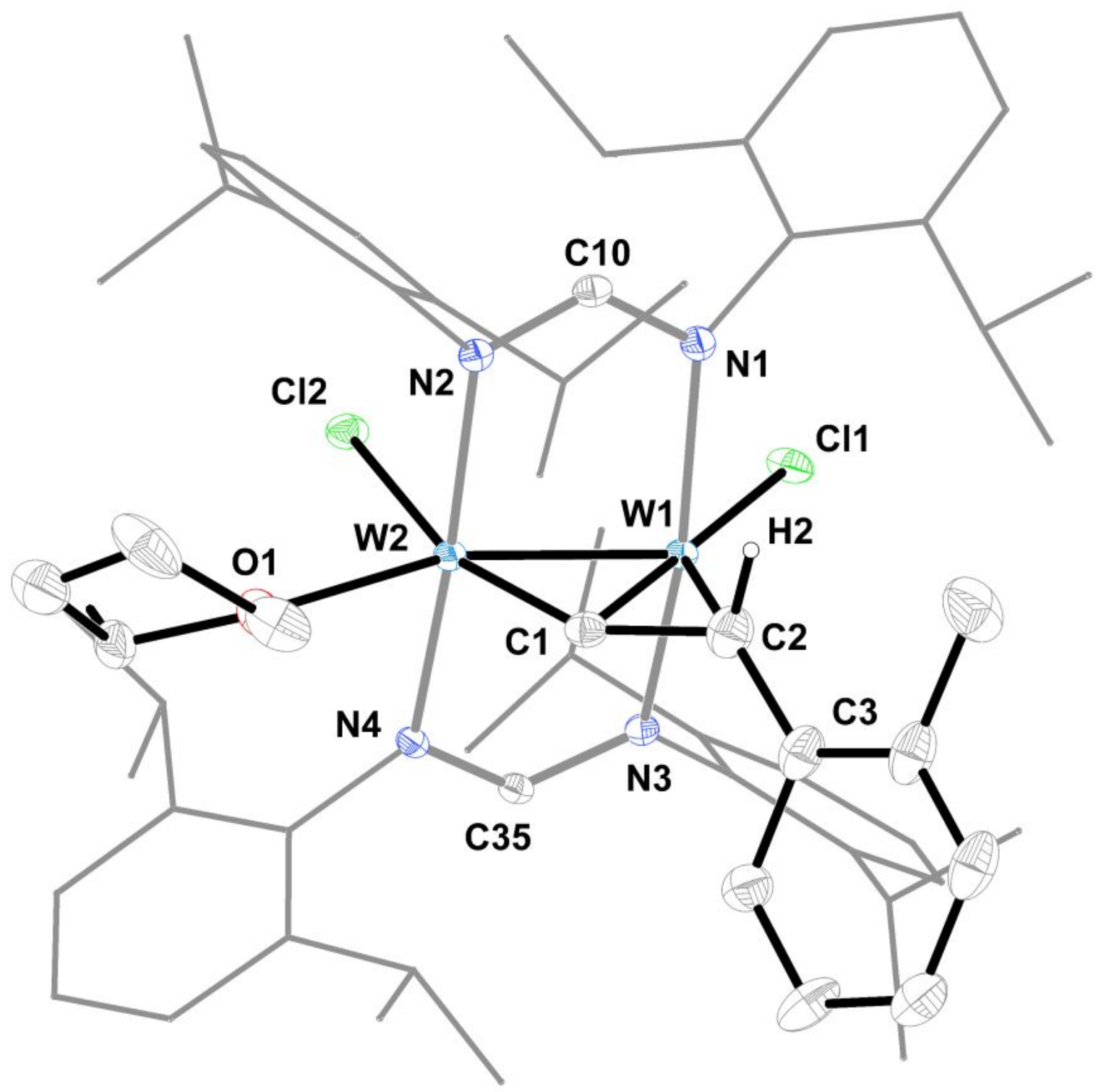

Figure. *S*21 The solid-state molecular structure of **20** drawn with 30% thermal ellipsoids probability level. Hydrogen atoms are omitted for the sake of clarity. Selected bond lengths (Å) and angles (°): W(1)–W(2) 2.3311(2), W(1)–Cl(1) 2.3717(8), W(1)–N(1) 2.143(3), W(1)–N(3) 2.130(3), W(1)–C(1) 2.163(4), W(1)–C(2) 2.316(4), W(2)–Cl(2) 2.4135(8), W(2)–O(1) 2.374(3), W(2)–N(2) 2.188(3), W(2)–N(4) 2.150(3), W(2)–C(1) 1.911(4), N(1)–C(10) 1.348(4), N(2)–C(10) 1.313(4), N(3)–C(35) 1.348(4), N(4)–C(35) 1.322(4), C(1)–C(2) 1.430(6), C(2)–C(3) 1.469(6); W(1)–W(2)–Cl(2) 100.71(2), W(1)–W(2)–O(1) 155.09(7), W(1)–W(2)–C(1) 60.35(11), W(1)–C(1)–W(2) 69.50(11), W(1)–C(1)–C(2) 77.3(2), W(1)–C(2)–C(1) 65.6(2), W(1)–C(2)–C(3) 134.8(3), W(2)–W(1)–Cl(1) 130.65(2), W(2)–W(1)–C(1) 50.15(10), W(2)–C(1)–C(2) 145.0(3), Cl(1)–W(1)–C(1) 178.11(10), Cl(1)–W(1)–C(2) 141.82(11), Cl(2)–W(2)–C(1) 160.56(11), Cl(2)–W(2)–O(1) 104.18(7), O(1)–W(2)–C(1) 94.90(13), N(1)–W(1)–N(3) 160.97(11), N(2)–W(2)–N(4) 174.62(10), C(1)–C(2)–C(3) 123.9(4).

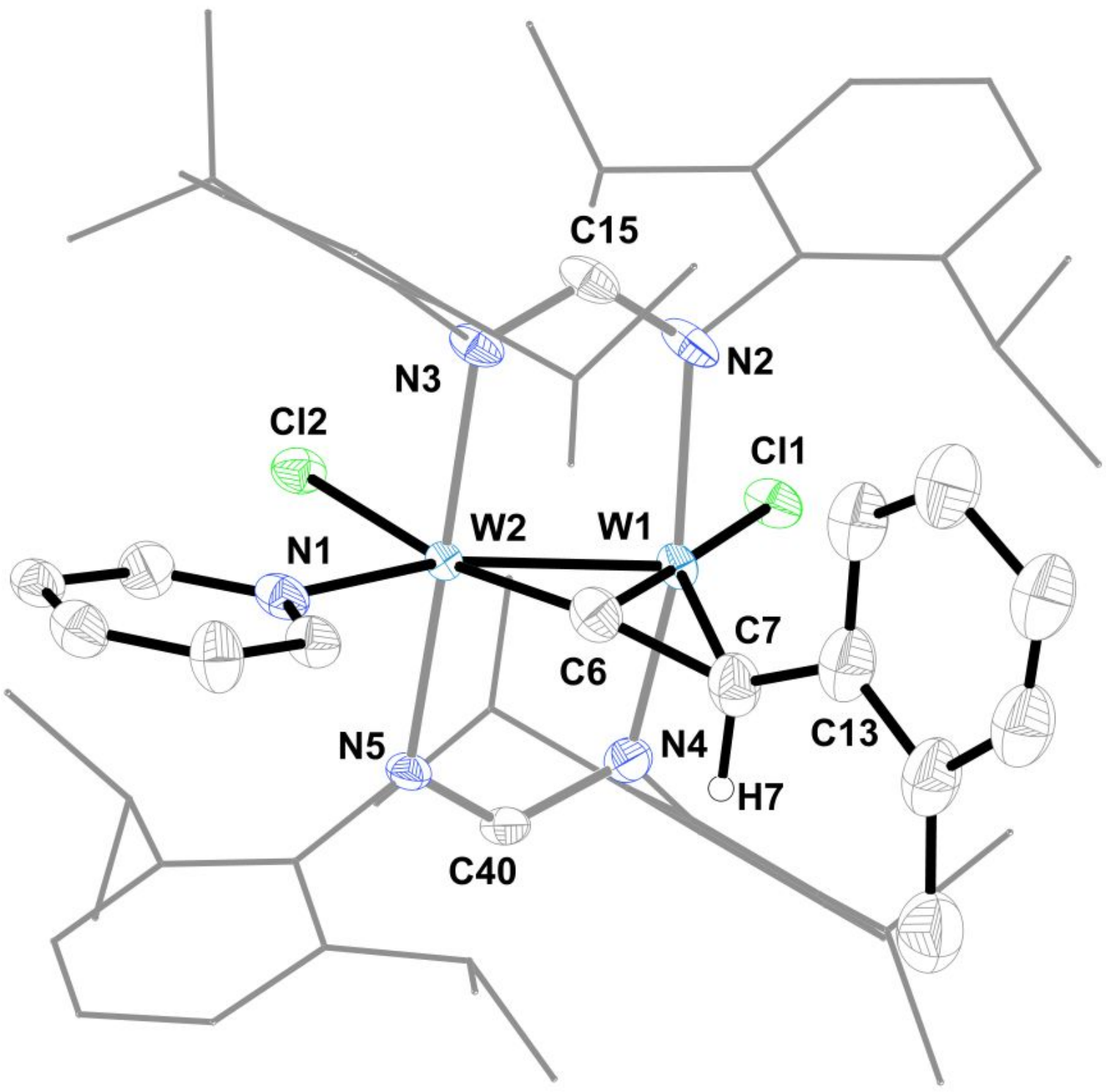

Figure. *S*22 The solid-state molecular structure of **21** drawn with 30% thermal ellipsoids probability level. Hydrogen atoms are omitted for the sake of clarity. Selected bond lengths (Å) and angles (°): W(1)−W(2) 2.3607(16), W(1)−Cl(1) 2.390(2), W(1)−C(6) 2.07(4), W(1)−C(7) 2.333(17), W(1)−N(2) 2.147(3), W(1)−N(4) 2.134(6), W(2)−Cl(2) 2.4166(19), W(2)−N(1) 2.296(6), W(2)−N(3) 2.173(6), W(2)−N(5) 2.153(6), W(2)−C(6) 1.85(3), C(6)−C(7) 1.47(3), C(7)−C(13) 1.51(3); W(1)−W(2)−C1(2) 106.15(6), W(1)−W(2)−C(6) 57.4(12), W(1)−W(2)−N(1) 151.33(15), W(1)−C(6)−C(7) 80.5(17), W(1)−C(7)−C(6) 61.1(16), W(2)−W(1)−C1(1) 131.53(8), W(2)−W(1)−C(6) 48.7(7), W(2)−W(1)−C(7) 85.9(4), W(2)−C(6)−C(7) 149(3); W(2)−W(1)−C(7)−C(6) 12.9(14), C(6)−W(2)−W(1)−C(7) 10.7(13).

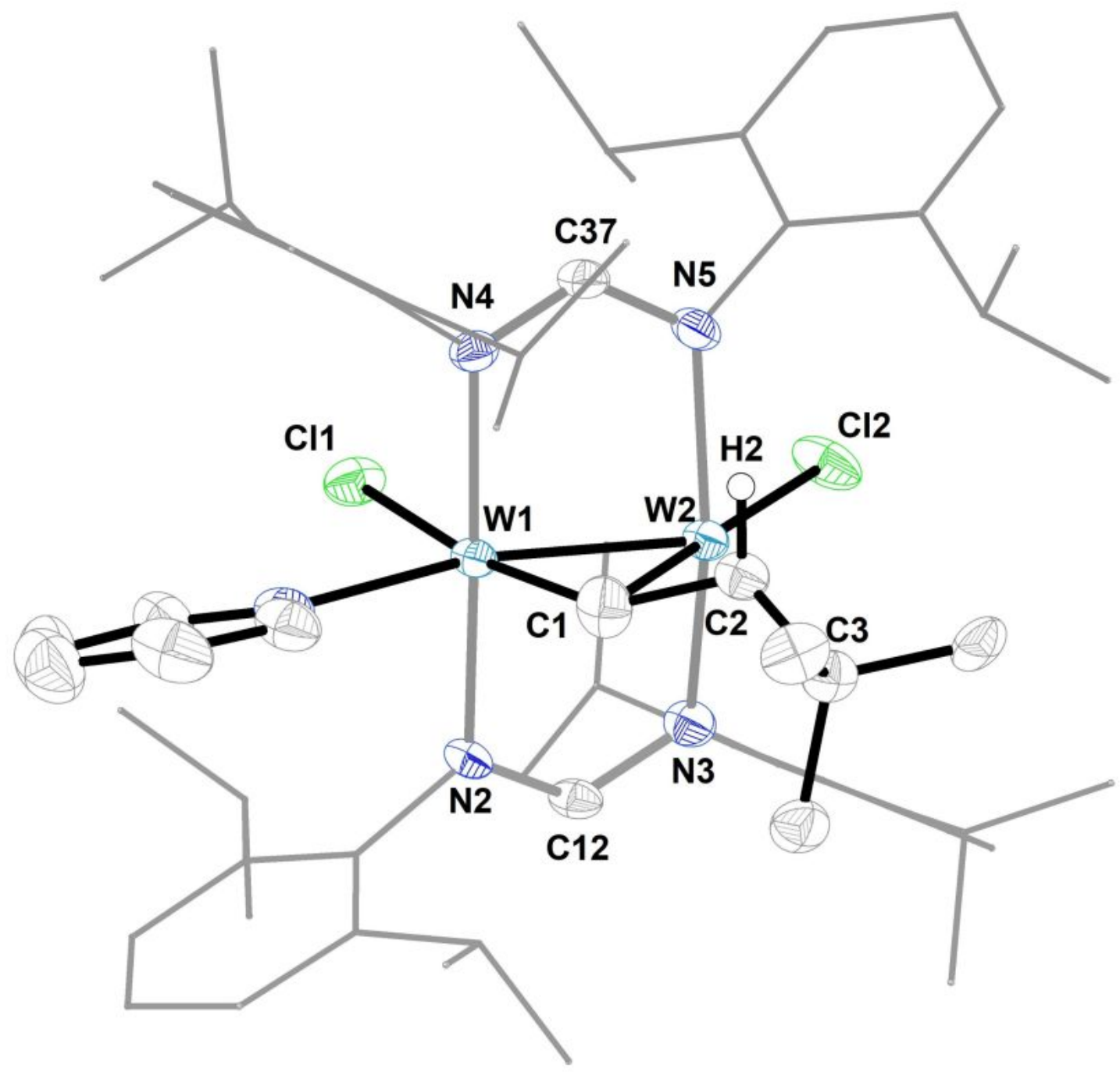

Figure. *S*23 The solid-state molecular structure of **22** drawn with 30% thermal ellipsoids probability level. Hydrogen atoms are omitted for the sake of clarity. Selected bond lengths (Å) and angles (°): W(1)−W(2) 2.3476(5), W(1)−Cl(1) 2.432(1), W(1)−C(1) 1.879(3), W(1)−N(1) 2.326(3), W(1)−N(2) 2.161(3), W(1)−N(4) 2.170(3), W(2)−Cl(2) 2.3669(10), W(2)−N(3) 2.132(3), W(2)−N(5) 2.128(3), W(2)−C(1) 2.152(3), W(2)−C(2) 2.317(5), C(1)−C(2) 1.468(6), C(2)−C(3) 1.534(6); W(1)−W(2)−C1(2) 133.56(3), W(2)−W(1)−C1(1) 105.72(3), W(1)−W(2)−C(1) 49.05(9), W(1)−W(2)−C(2) 85.84(13), W(1)−C(1)−C(2) 143.1(3), W(2)−W(1)−C(1) 60.03(10), W(2)−C(1)−C(2) 77.1(2), W(2)−C(2)−C(1) 64.7(2), N(1)−W(1)−W(2) 156.83(8); W(2)−W(1)−C(1)−C(2) 31.3(5), C(1)−W(1)−W(2)−C(2) 11.47(19), N(1)−W(1)−W(2)−C(2) 2.4(3).

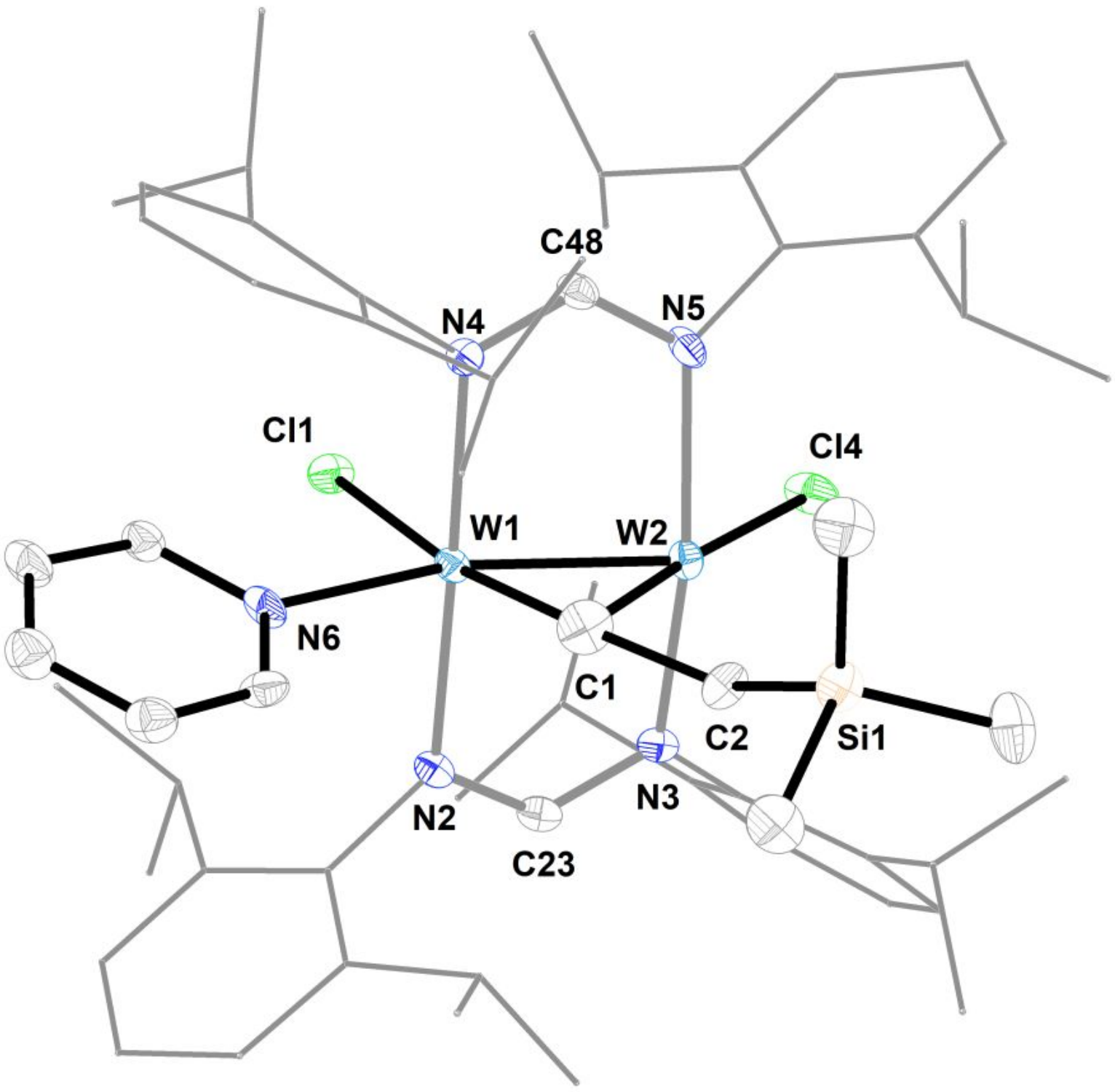

Figure. *S*24 The solid-state molecular structure of **23** drawn with 30% thermal ellipsoids probability level. Hydrogen atoms are omitted for the sake of clarity. Selected bond lengths (Å) and angles (°): W(1)–W(2) 2.35892(3), W(1)–N(2) 2.174(5), W(1)–N(4) 2.161(5), W(1)–C(1) 1.885(7), W(1)–Cl(1) 2.4309(17), W(1)–N(6) 2.29(3), W(2)–N(3) 2.125(5), W(2)–N(5) 2.126(5), W(2)–C(1) 2.163(7), W(2)–C(2) 2.289(9), W(2)–Cl(4) 2.3742(17), C(1)–C(2) 1.483(12), C(2)–Si(1) 1.872(11); W(1)–W(2)–C(1) 49.04(18), W(1)–W(2)–C(2) 86.8(2), W(1)–W(2)–Cl(4) 135.18(4), W(1)–C(1)–C(2) 142.7(6), W(1)–C(1)–W(2) 70.9(2), W(2)–W(1)–N(6) 150.7(9), W(2)–W(1)–C(1) 60.0(2), W(2)–W(1)–Cl(1) 105.56(4), W(2)–C(1)–C(2) 75.2(4), W(2)–C(2)–C(1) 66.0(4), C(1)–W(2)–C(2) 38.8(3); W(1)–W(2)–C(2)–C(1) 12.0(4), Cl(1)–W(1)–W(2)–Cl(4) 1.42(10), C(1)–W(1)–W(2)–C(2) 9.9(3).

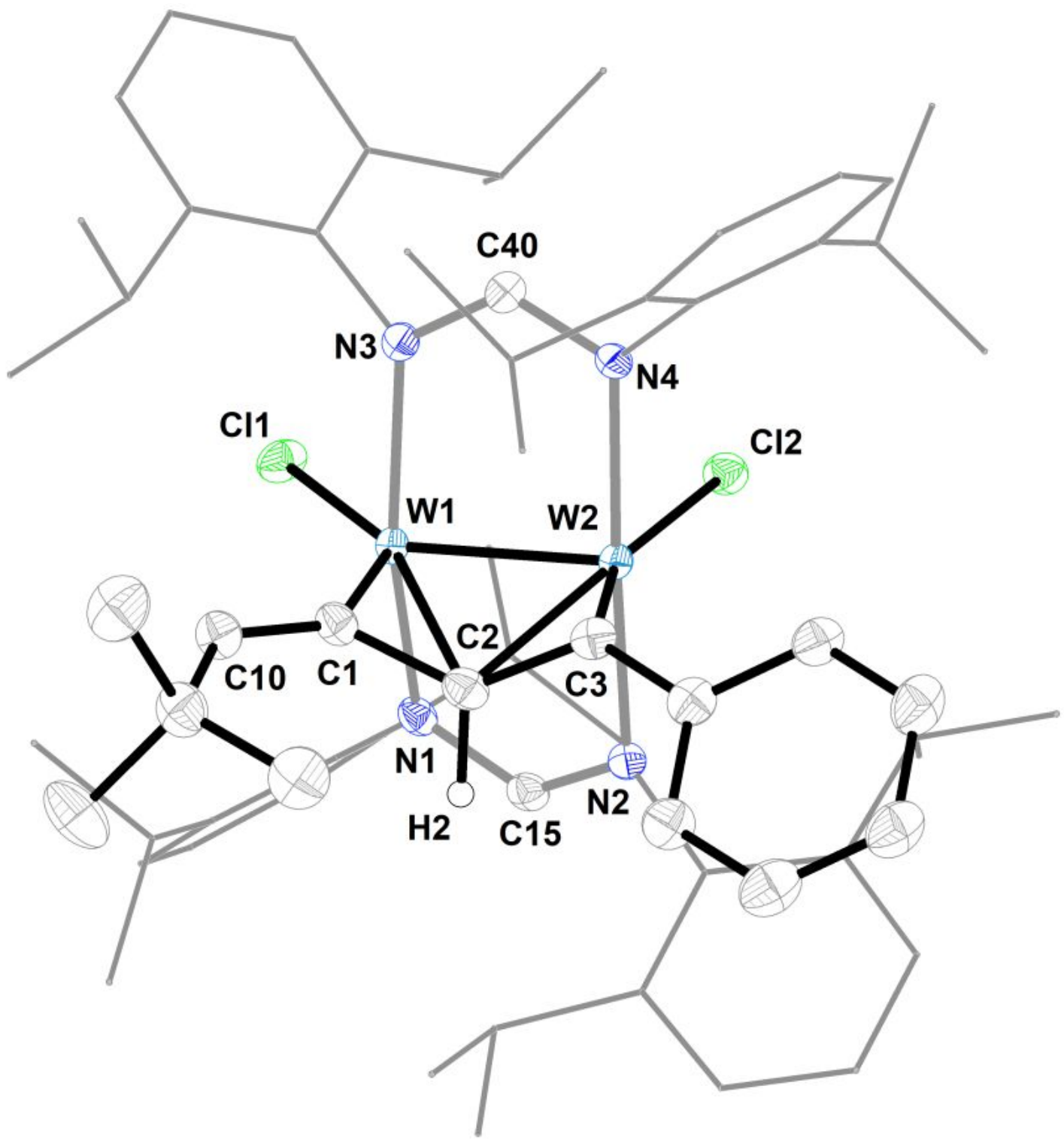

Figure. *S*25 The solid-state molecular structure of **24** drawn with 30% thermal ellipsoids probability level. Hydrogen atoms are omitted for the sake of clarity. Selected bond lengths (Å) and angles (°): W(1)−W(2) 2.3252(6), W(1)−Cl(1) 2.374(1), W(1)−N(1) 2.115(3), W(1)−N(3) 2.099(3), W(1)−C(10) 3.170(4), W(1)−C(1) 2.089(3), W(1)−C(2) 2.514(4), W(2)−Cl(2) 2.4129(9), W(2)−N(2) 2.183(3), W(2)−N(4) 2.164(3), W(2)−C(2) 2.389(4), W(2)−C(3) 1.944(3), C(10)−C(1) 1.348(5), C(1)−C(2) 1.446(5), C(2)−C(3) 1.452(5); W(1)−W(2)−C(3) 98.56(11), W(1)−C(2)−W(2) 56.54(8), W(2)−W(1)−C(1) 90.38(10), W(1)−C(1)−C(10) 133.4(3), W(1)−C(1)−C(2) 88.7(2), W(2)−C(3)−C(2) 88.2(2), C(10)−C(1)−C(2) 136.0(3), C(1)−C(2)−C(3) 130.7(3); Cl(1)−W(1)−W(2)−Cl(2) 2.20(4), C(1)−W(1)−W(2)−C(4) 0.92(13), C(1)−C(2)−C(3)−C(4) 112.0(5), W(1)−W(2)−C(3)−C(2) 23.71(19).

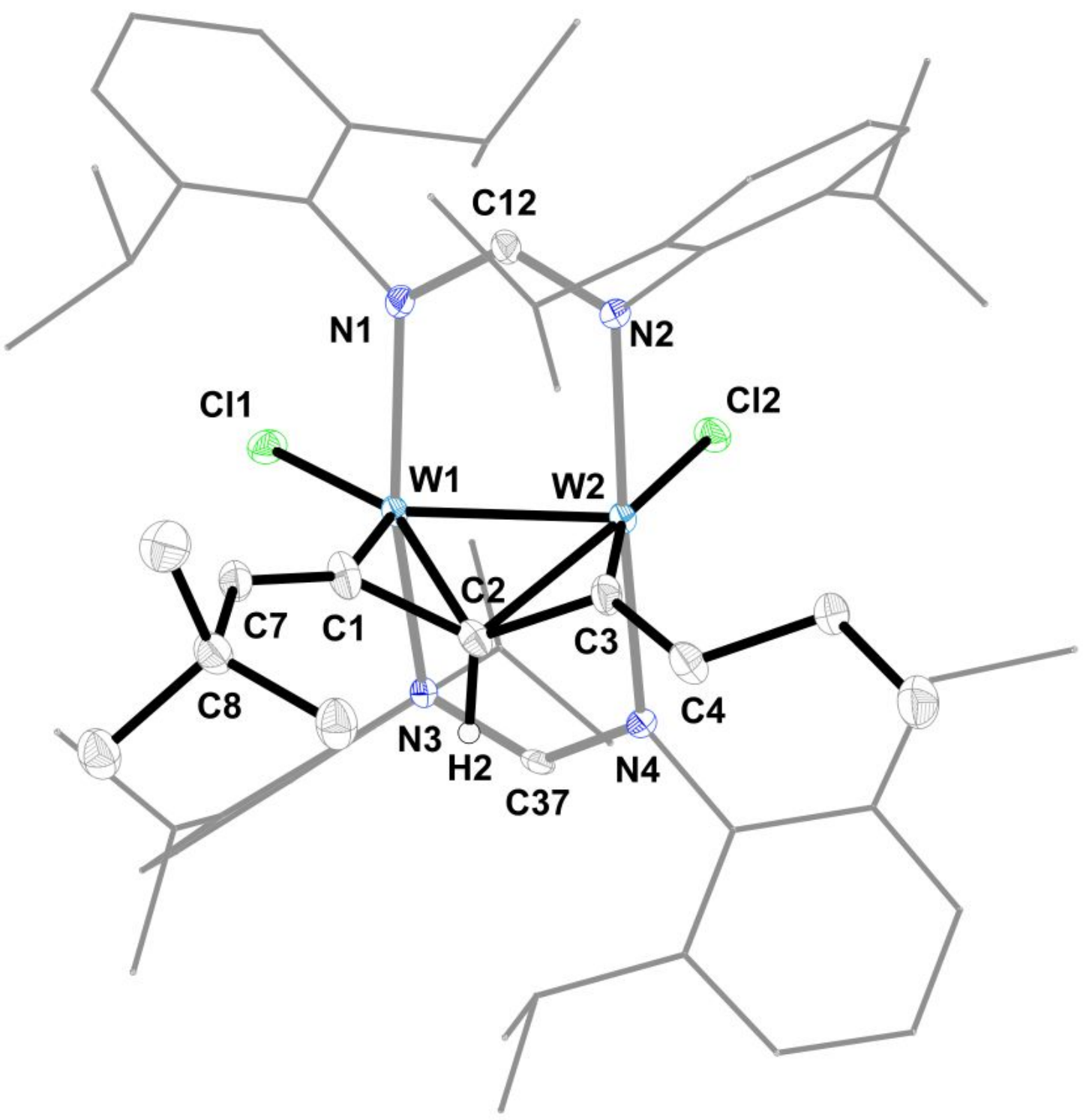

Figure. *S*26 The solid-state molecular structure of **25** drawn with 30% thermal ellipsoids probability level. Hydrogen atoms are omitted for the sake of clarity. Selected bond lengths (Å) and angles (°): W(1)−W(2) 2.3401(4), W(1)−Cl(1) 2.3861(10), W(1)−N(1) 2.118(3), W(1)−N(3) 2.119(3), W(2)−Cl(2) 2.4140(9), W(2)−N(2) 2.153(3), W(2)−N(4) 2.196(3), W(1)−C(1) 2.095(4), W(1)−C(2) 2.415(4), W(2)−C(2) 2.336(4), W(2)−C(3) 1.975(4), C(1)−C(2) 1.446(5), C(1)−C(7) 1.331(5), C(2)−C(3) 1.459(5), C(3)−C(4) 1.472(6), C(7)−C(8) 1.509(6); W(1)−W(2)−C(2) 62.19(10), W(1)−W(2)−C(3) 98.21(11), W(1)−W(2)−Cl(2) 98.77(3), W(1)−C(1)−C(2) 83.8(2), W(1)−C(1)−C(7) 137.7(3), W(1)−C(2)−C(3) 112.8(3), W(2)−W(1)−Cl(1) 135.28(3), W(2)−C(2)−C(1) 111.1(3), W(2)−C(2)−C(3) 57.28(19), W(2)−C(3)−C(2) 84.3(2), W(2)−C(3)−C(4) 140.2(3), C(1)−C(2)−C(3) 132.3(4), C(2)−C(3)−C(4) 125.5(3), C(2)−C(1)−C(7) 137.1(4); C(1)−W(1)−W(2)−C(2) 18.83(16), C(1)−W(1)−W(2)−C(3) 4.97(16), C(2)−W(1)−W(2)−C(3) 13.86(16), Cl(1)−W(1)−W(2)−Cl(2) 0.06(5),

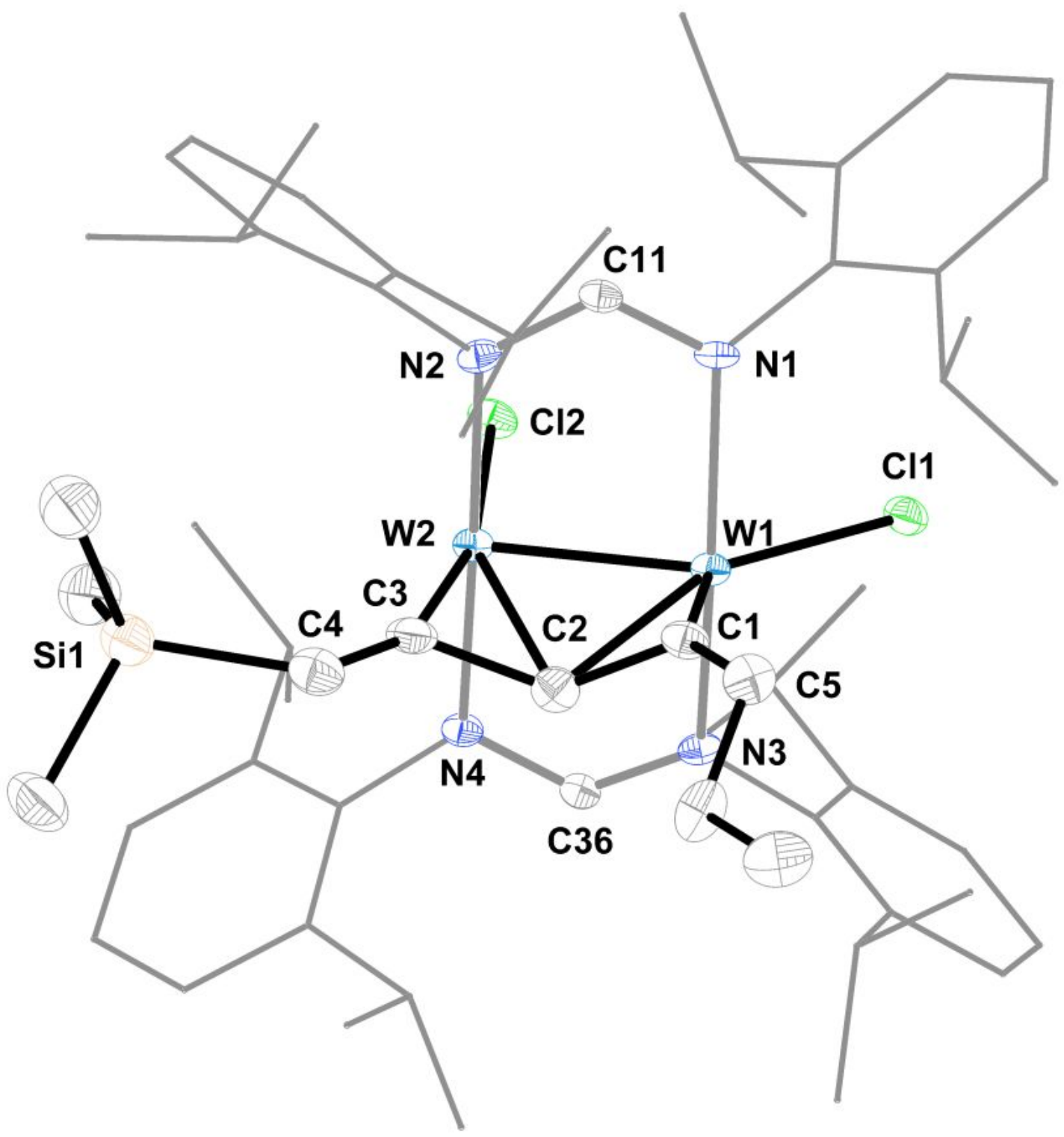

Figure. ***S*27** The solid-state molecular structure of **26** drawn with 30% thermal ellipsoids probability level. Hydrogen atoms are omitted for the sake of clarity. Selected bond lengths (Å) and angles (°): W(1)–W(2) 2.3668(5), W(1)–C(1) 1.926(4),W(1)–C(2) 2.341(4), W(1)–Cl(1) 2.353(1), W(1)–N(1) 2.167(3), W(1)–N(3) 2.175(3), W(2)–C(2) 2.322(4), W(2)–C(3) 2.077(4), W(2)–Cl(2) 2.353(1), W(2)–N(2) 2.145(3), W(2)–N(4) 2.128(3), C(1)–C(2) 1.440(7), C(1)–C(5) 1.509(7), C(2)–C(3) 1.514(7), C(3)–C(4) 1.290(7), C(4)–Si(1) 1.886(6); W(1)–W(2)–C(2) 59.9(1), W(1)–W(2)–C(3) 97.3(1), W(1)–W(2)–Cl(2) 95.11(3), W(1)–C(1)–C(2) 86.9(3), W(1)–C(2)–C(1) 55.2(2), W(1)–C(1)–C(5) 147.6(3), W(2)–W(1)–C(1) 92.0(1), W(2)–W(1)–C(2) 59.1(1), W(2)–W(1)–Cl(1) 135.12(3), W(2)–C(2)–C(3) 61.4(2), W(2)–C(3)–C(2) 78.9(2), W(2)–C(3)–C(4) 150.2(4), C(1)–C(2)–C(3) 132.9(4), C(2)–C(3)–C(4) 129.4(4), C(2)–C(1)–C(5) 123.8(4), C(1)–W(1)–C(2) 37.9(2), C(2)–W(2)–C(3) 39.8(2); W(1)–W(2)–C(3)–C(2) 19.3(2), W(1)–W(2)–C(3)–C(4) -177.2(8), W(2)–W(1)–C(1)–C(2) -28.1(2), C(1)–W(1)–W(2)–C(3) 5.6(2), Cl(1)–W(1)–W(2)–Cl(2) 6.76(5).

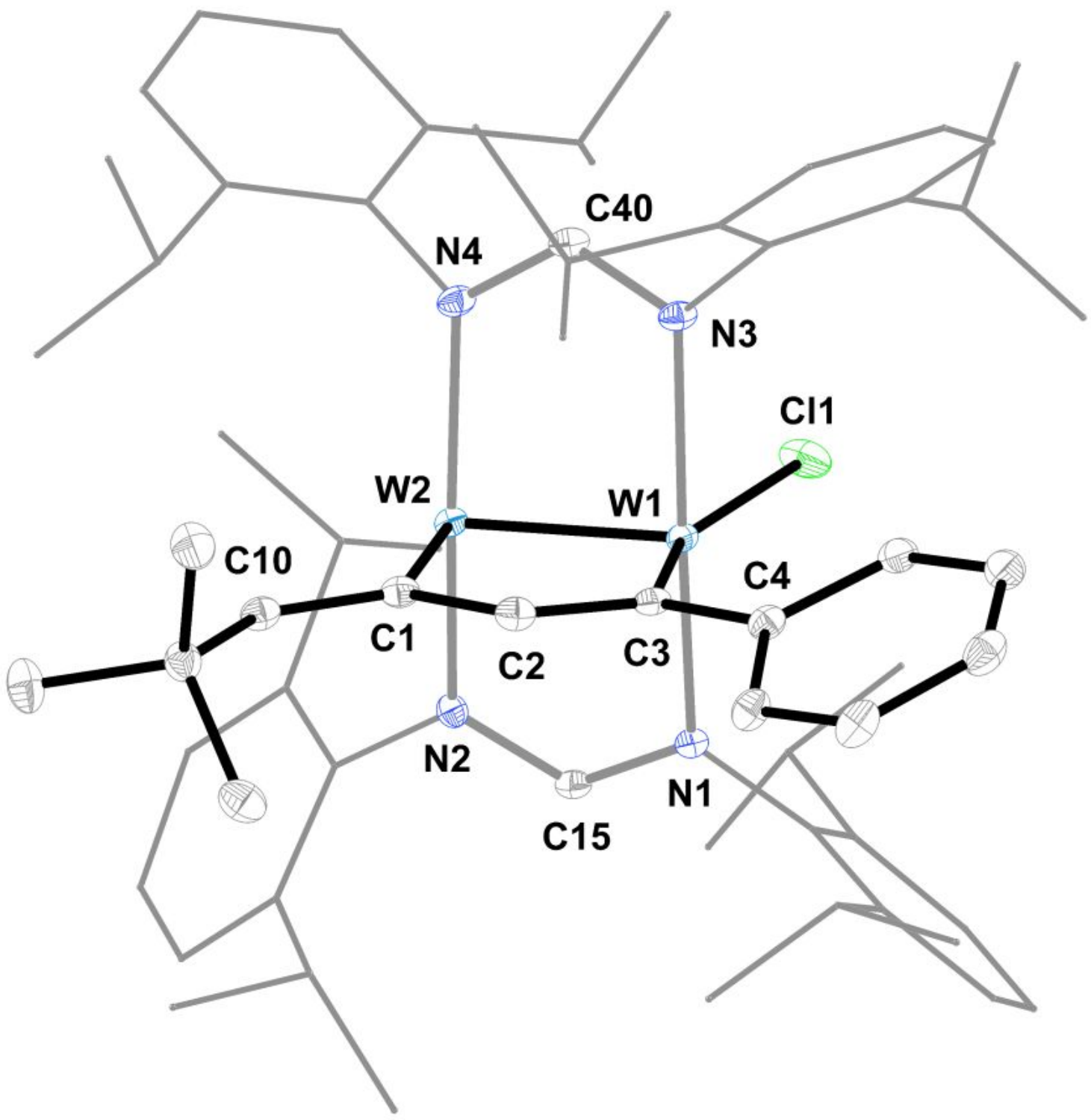

Figure. *S*28 The solid-state molecular structure of **27** drawn with 30% thermal ellipsoids probability level. Hydrogen atoms are omitted for the sake of clarity. Selected bond lengths (Å) and angles (°): W(1)−W(2) 2.2387(4), W(1)−C(3) 2.136(3), W(1)−Cl(1) 2.4357(7), W(1)−N(1) 2.1761(19), W(1)−N(3) 2.1897(19), W(2)−C(1) 2.107(3), W(2)−N(2) 2.0400(19), W(2)−N(4) 2.091(2), C(1)−C(2) 1.462(4), C(1)−C(10) 1.355(3), C(2)−C(3) 1.366(3), C(3)−C(4) 1.490(3); W(1)−W(2)−C(1) 90.97(7), W(2)−W(1)−C(3) 94.67(6), W(1)−C(1)−C(2) 118.24(17), C(1)−C(2)−C(3) 120.0(2), W(1)−C(3)−C(2) 115.87(18), W(2)−W(1)−Cl(1) 106.28(2); C(1)−W(2)−W(1)−C(3) 2.2(1), Cl(1)−W(2)−W(1)−C(1) 178.52(7).

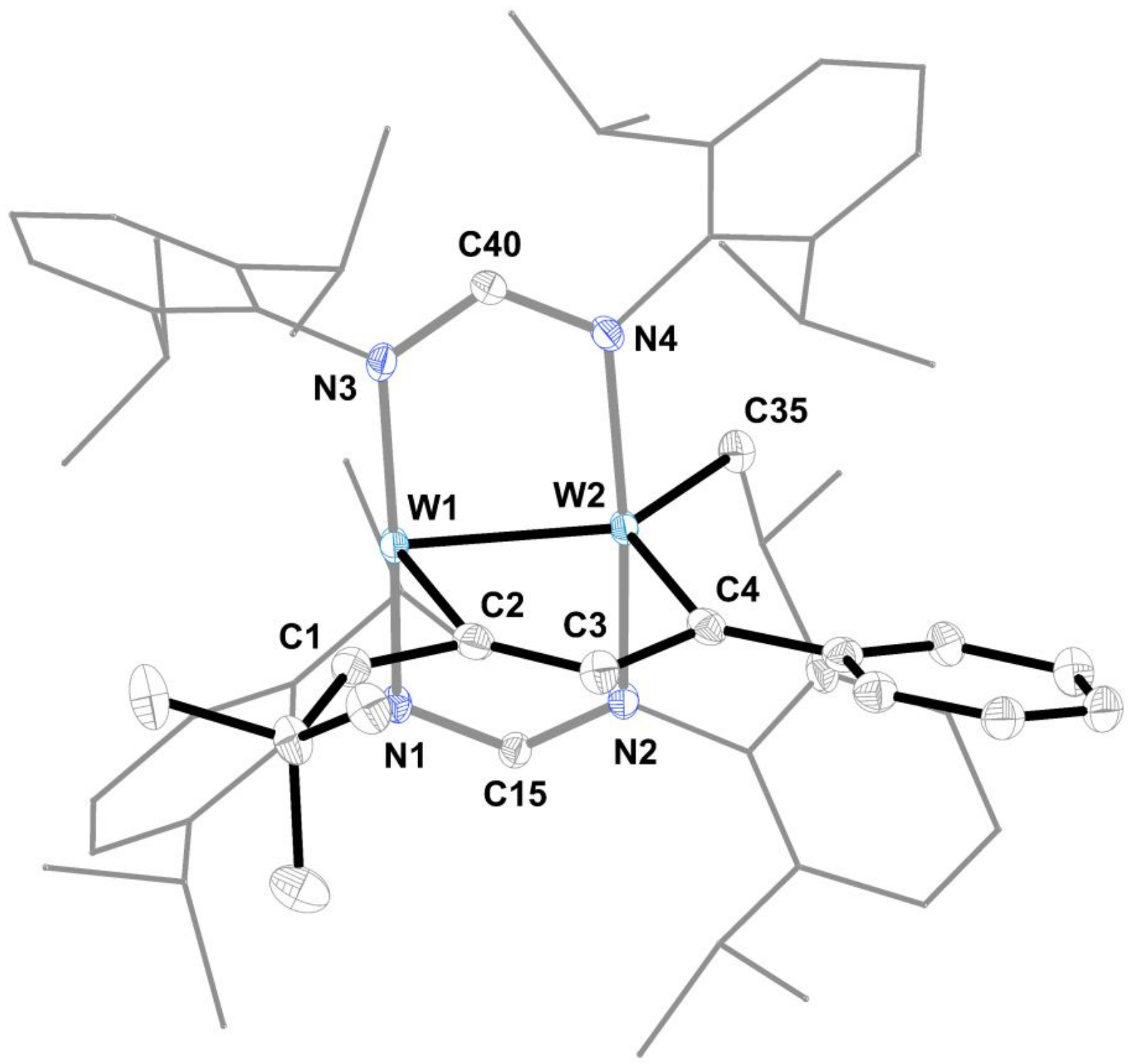

Figure. ***S*29** The solid-state molecular structure of **28** drawn with 30% thermal ellipsoids probability level. Hydrogen atoms are omitted for the sake of clarity. Selected bond lengths (Å) and angles (°): W(1)−W(2) 2.3495(3), W(1)−N(1) 2.112(3), W(1)−N(3) 2.087(3), W(1)−C(1) 2.452(4), W(1)−C(2) 2.105(4), W(2)−N(2) 2.101(3), W(2)−N(4) 2.146(3), W(2)−C(4) 2.099(4), W(2)−C(35) 2.151(4), C(1)−C(2) 1.334(5), C(2)−C(3) 1.436(5), C(3)−C(4) 1.372(6); W(1)−W(2)−C(4) 109.57(11), W(1)−W(2)−C(35) 125.82(9), W(2)−W(1)−C(1) 104.18(8),W(2)−W(1)−C(2) 71.25(11), C(1)−W(1)−C(2) 32.94(13), C(1)−C(2)−C(3) 132.3(4), C(2)−C(3)−C(4) 115.7(4), C(4)−W(2)−C(35) 123.86(13); C(1)−W(1)−W(2)−C(4) 2.10(13), C(1)−W(1)−W(2)−C(35) 172.45(15), C(1)−C(2)−C(3)−C(4) 179.5(4), C(2)−W(1)−W(2)−C(4) 1.40(14).

4 NMR Spectroscopy

4.1 $[Li(THF)_4][\{WCl_2(\mu\text{-}\kappa^2\text{-}HC(N\text{-}2,6\text{-}{}^iPr_2C_6H_3)_2)\}_2]$ (1)

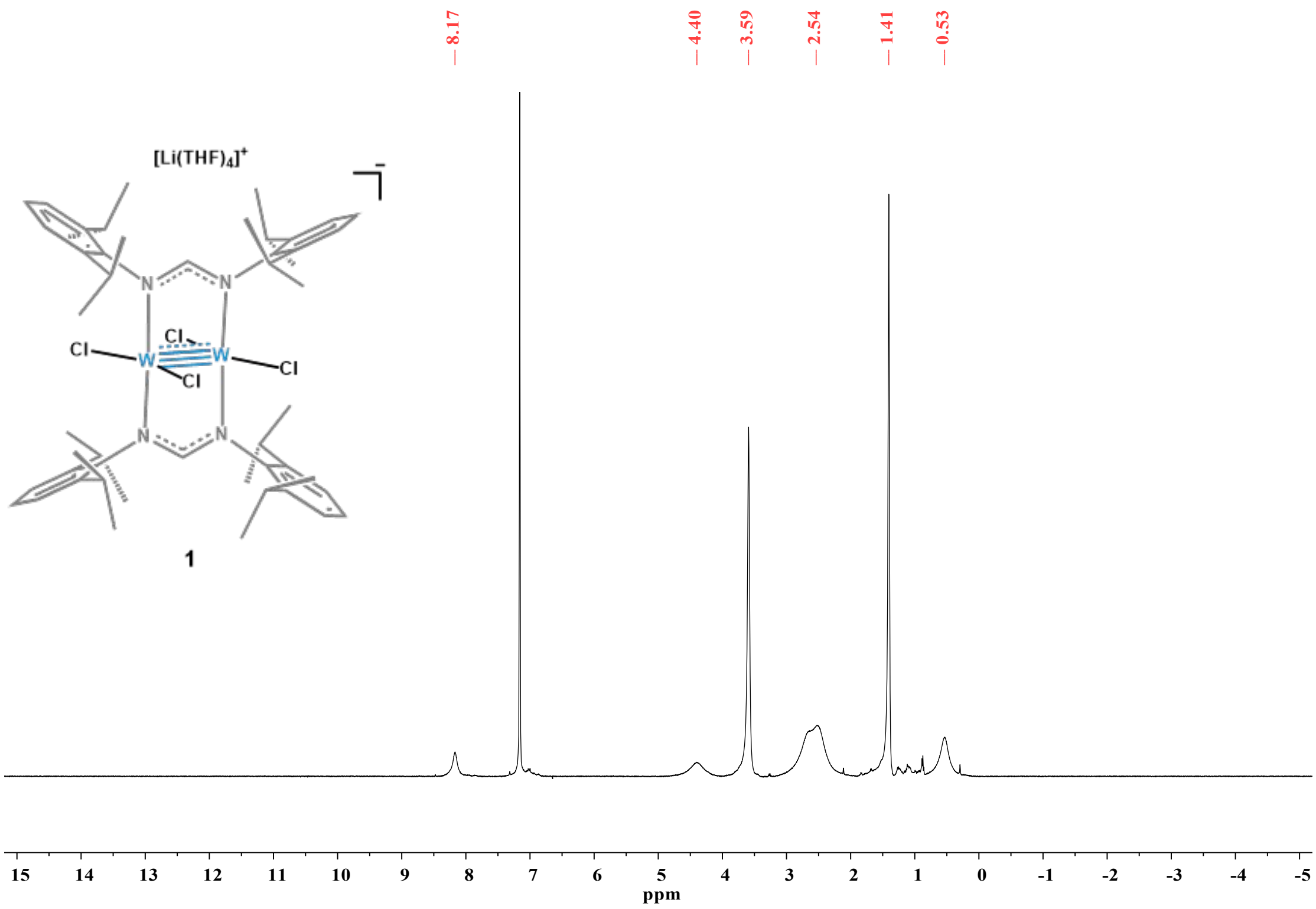

Figure. *S*30 ^1H NMR spectrum of **1** (500 MHz, C_6D_6, 298 K).

4.2 $W_2(\mu\text{-}Cl)(\mu\text{-}\kappa^2\text{-}Cl_2Li(THF))[\mu\text{-}\kappa^2\text{-}HC(N\text{-}2,6\text{-}{}^iPr_2C_6H_3)_2]_2$ (2)

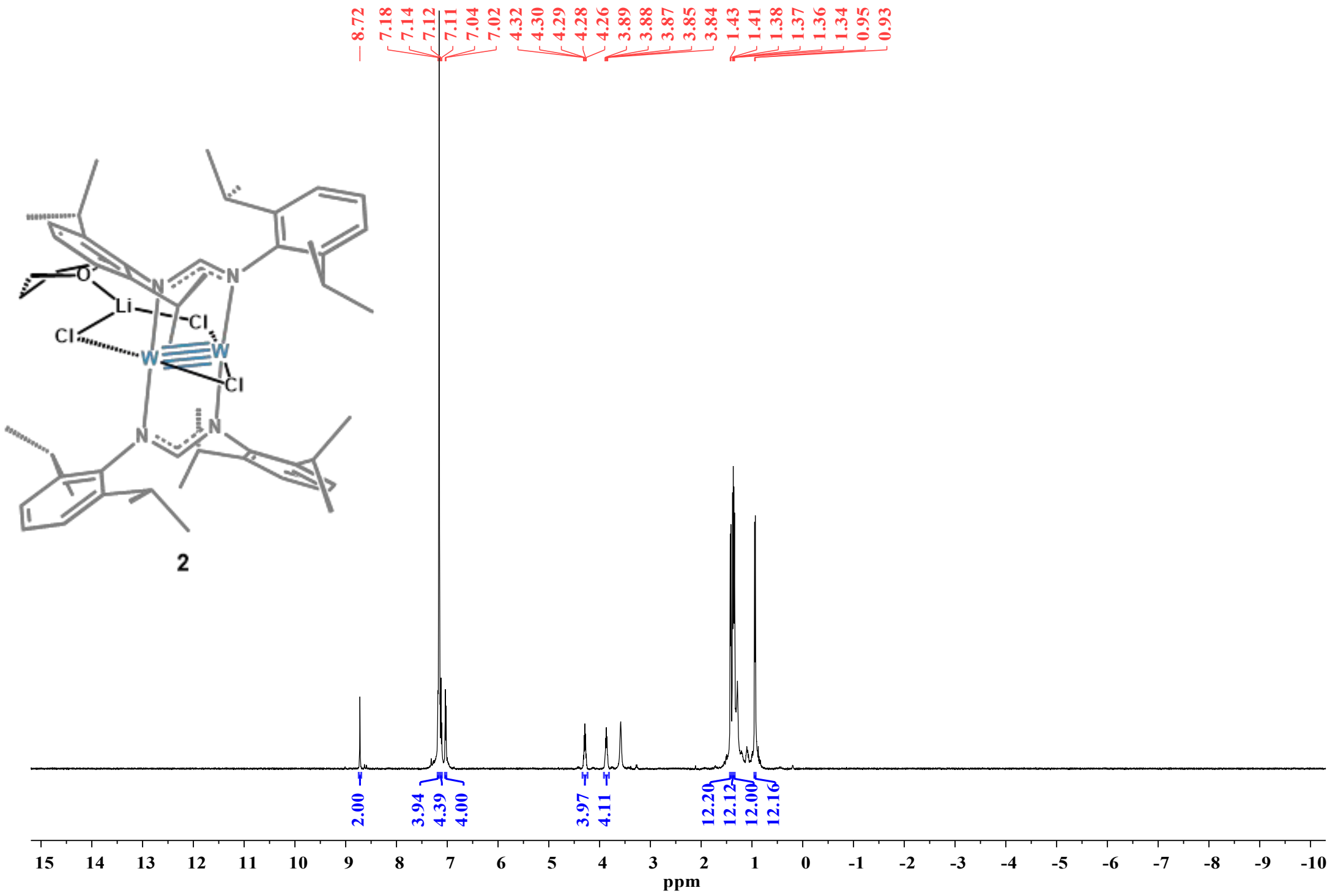

Figure. *S*31 ^{1}H NMR spectrum of **2** (500 MHz, C_6D_6, 298 K).

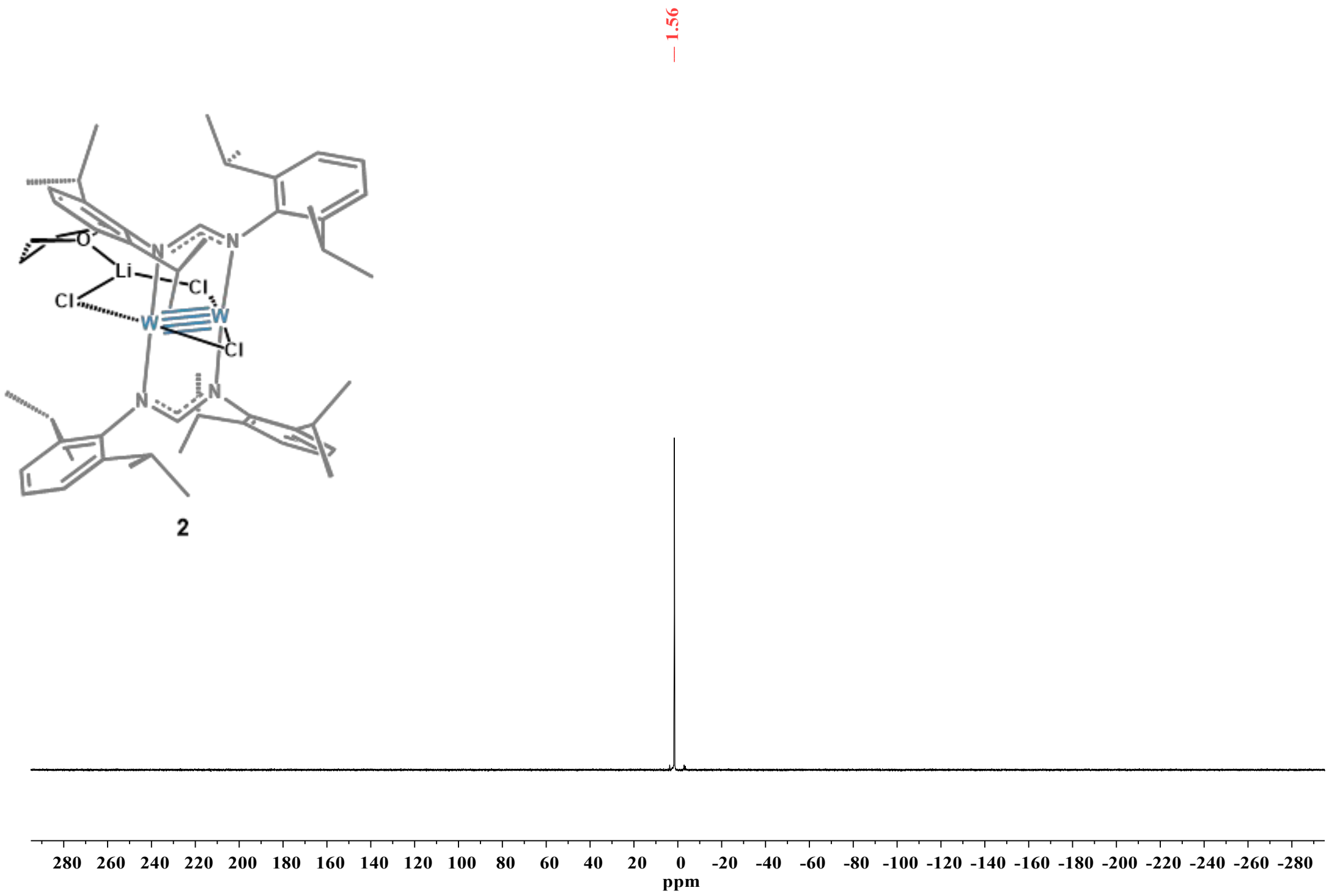

Figure. *S*32 ^{7}Li{^{1}H} NMR spectrum of **2** (194 MHz, C_6D_6, 298 K).

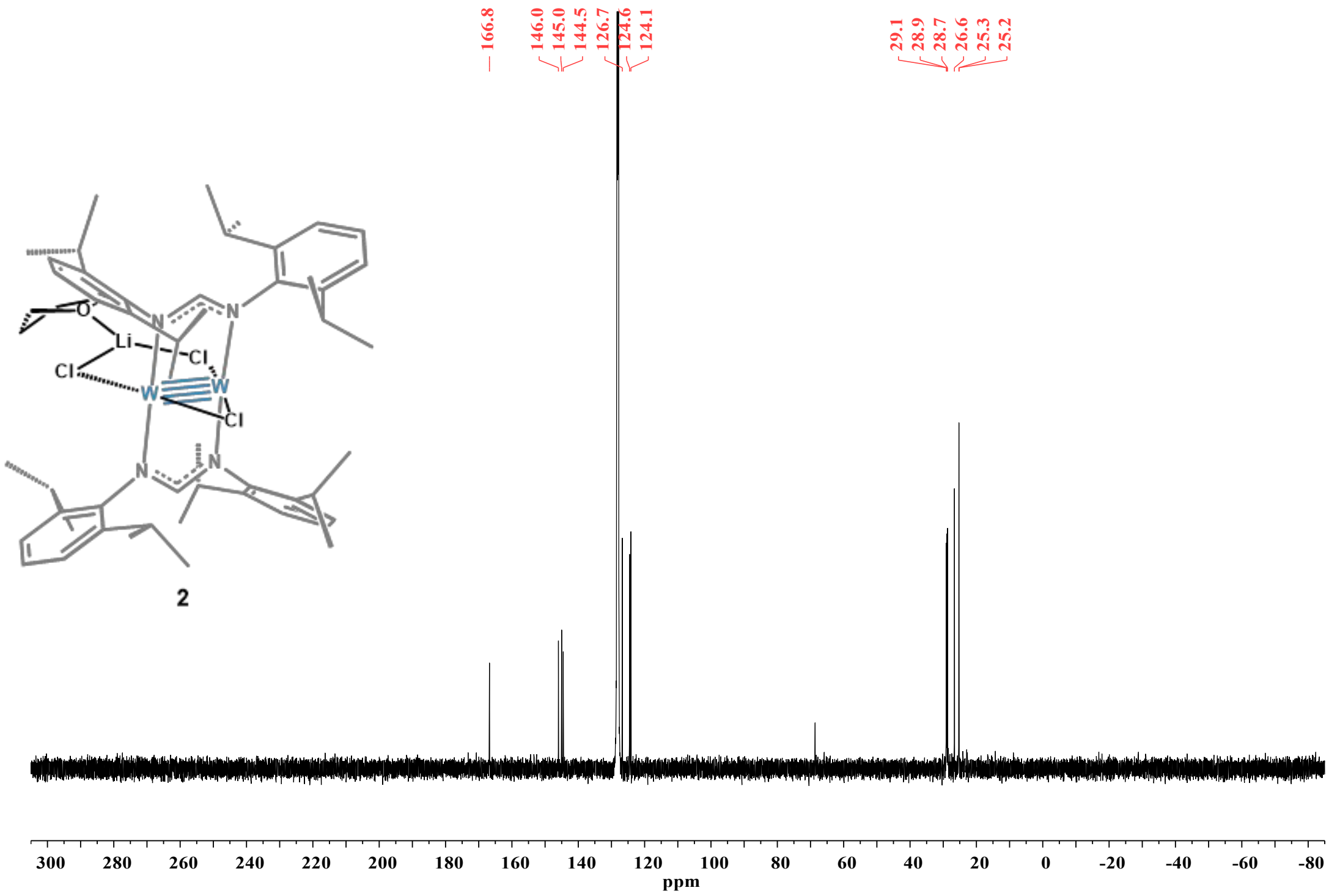

Figure. *S*33 $^{13}C\{^{1}H\}$ NMR spectrum of **2** (126 MHz, C_6D_6, 298 K).

4.3 $W_2Cl(\mu\text{-}Cl)(NC_5H_5)_2[\mu\text{-}\kappa^2\text{-}HC(N\text{-}2,6\text{-}{}^iPr_2C_6H_3)_2]_2$ (3)

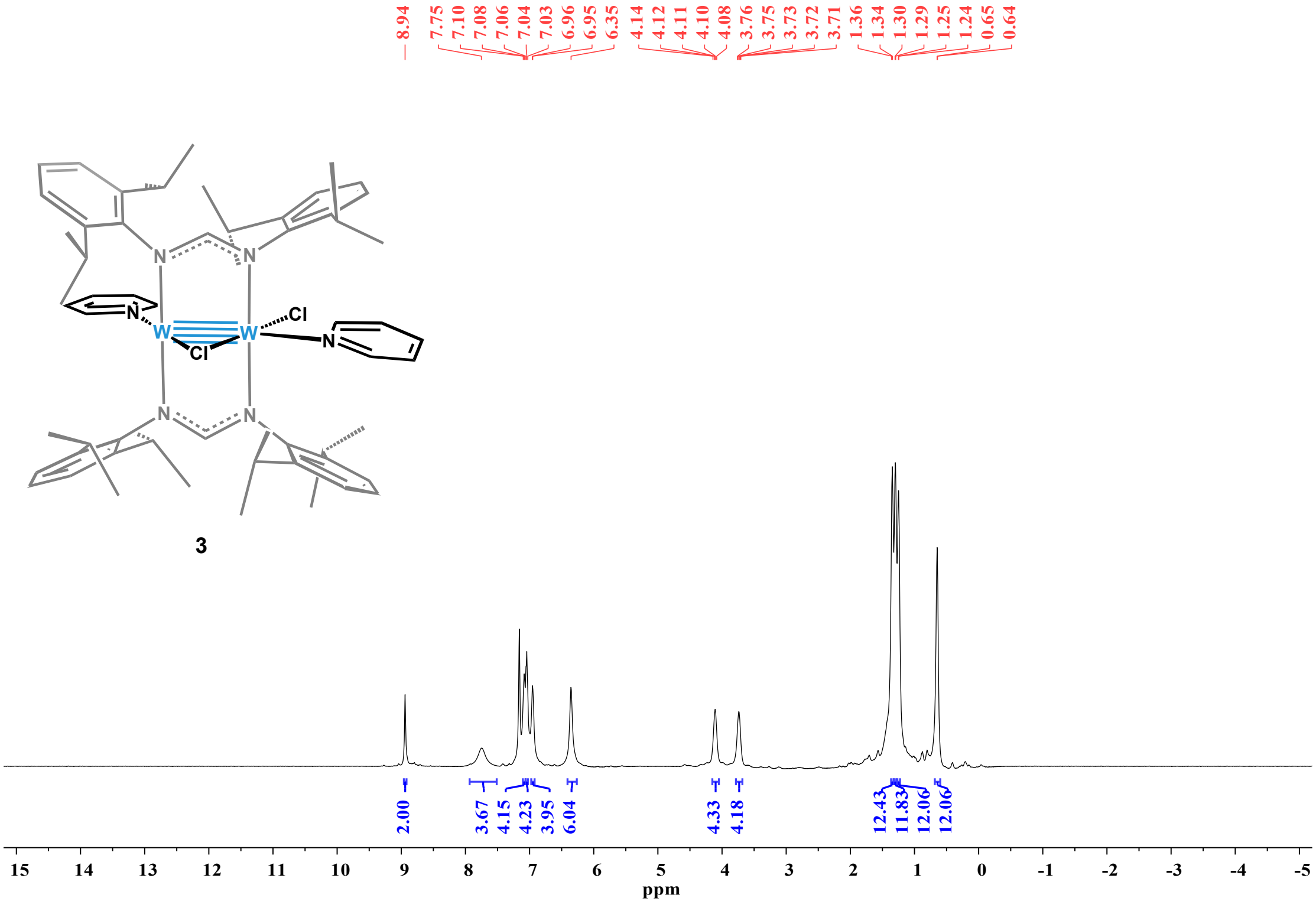

Figure. *S*34 ^{1}H NMR spectrum of **3** (500 MHz, C_6D_6, 298 K).

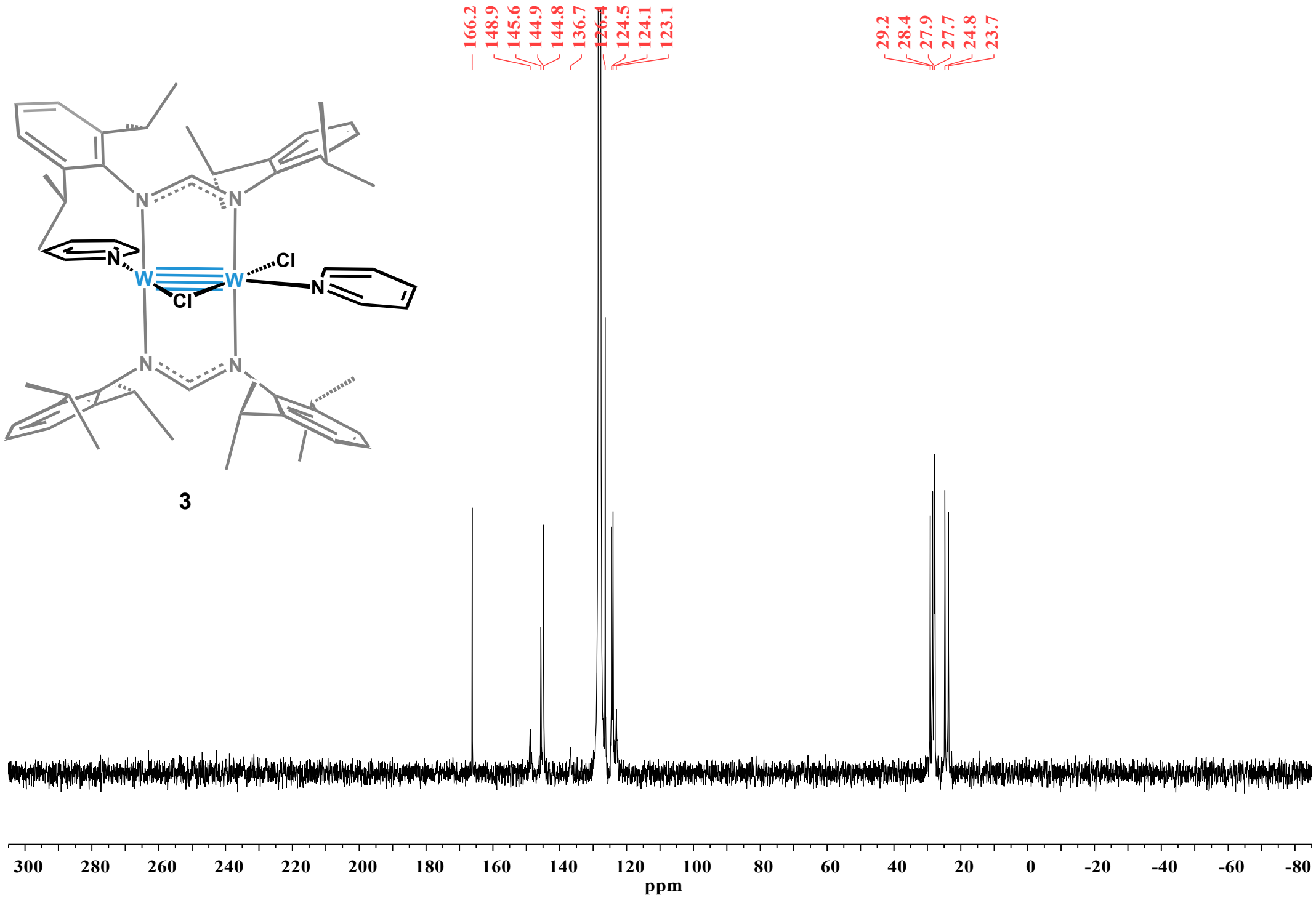

Figure. *S*35 $^{13}C\{^1H\}$ NMR spectrum of **3** (126 MHz, C_6D_6, 298 K).

4.4 $W_2(THF)(\mu\text{-}H)_2[\mu\text{-}\kappa^3\text{-}HC(N\text{-}2\text{-}CH(CH_2)(CH_3)\text{-}6\text{-}^iPrC_6H_3)(N\text{-}2,6\text{-}^iPr_2C_6H_3)]_2$ (4)

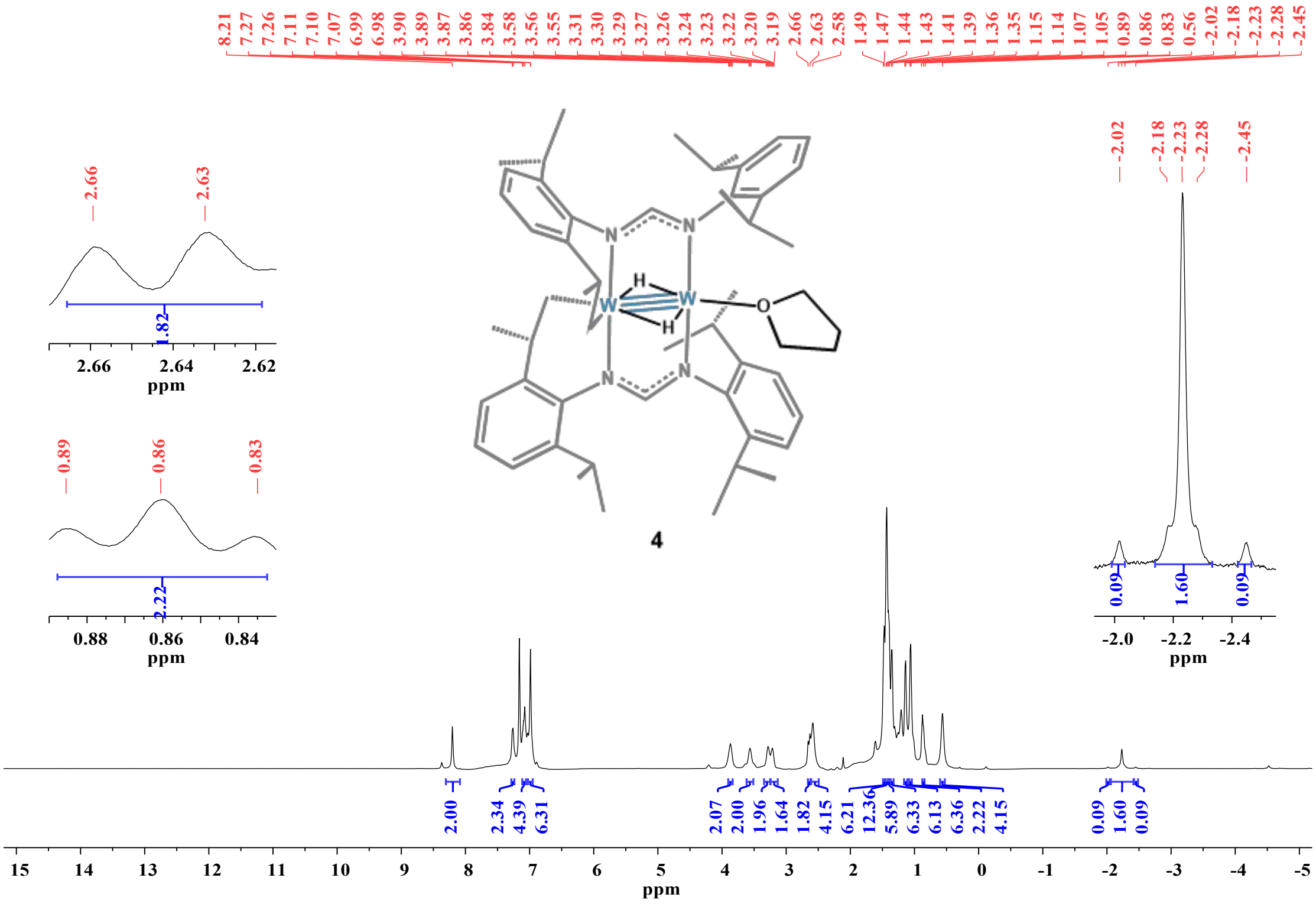

Figure. *S*36 ^1H NMR spectrum of **4** (500 MHz, C_6D_6, 298 K).

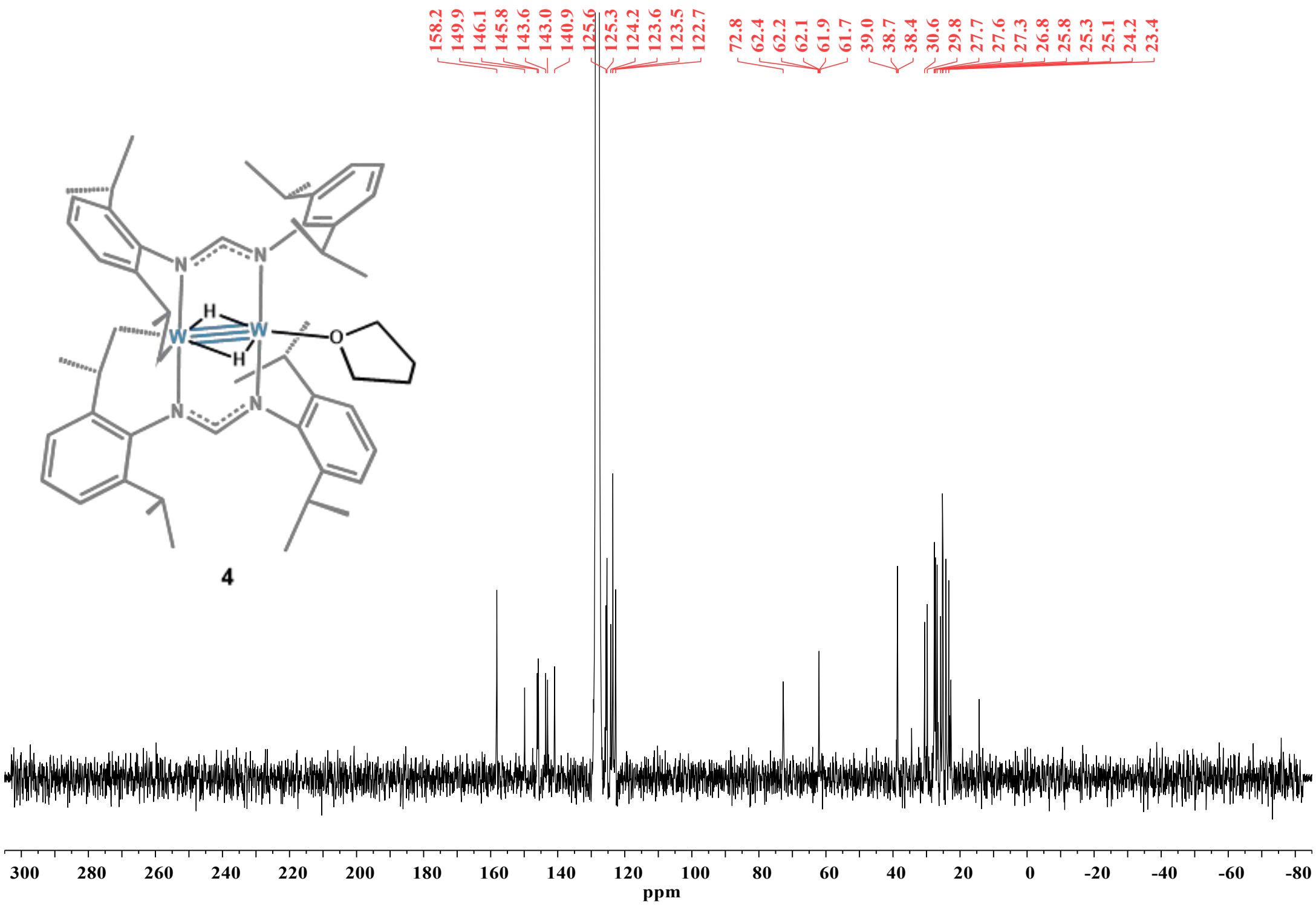

Figure. *S*37 $^{13}C\{^1H\}$ NMR spectrum of **4** (126 MHz, C_6D_6, 298 K).

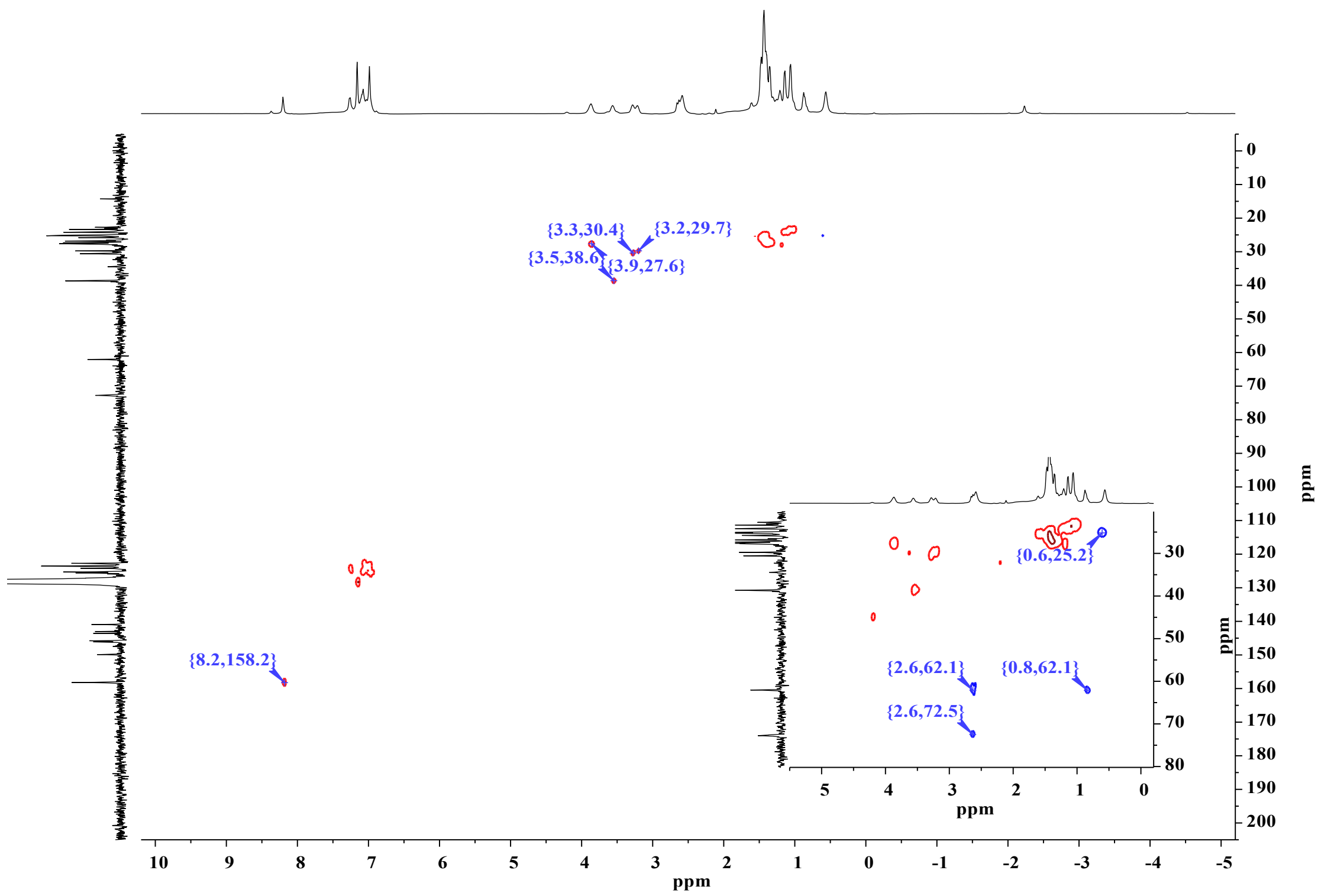

Figure. *S*38 HSQC (^1H-^{13}C) NMR spectrum of **4** (C_6D_6, 298 K).

4.5 $W_2(\mu\text{-}\kappa^2\text{-}1,4\text{-}Ph_2C_4H_2)[\mu\text{-}\kappa^2\text{-}HC(N\text{-}2,6\text{-}{}^iPr_2C_6H_3)_2]_2$ (5)

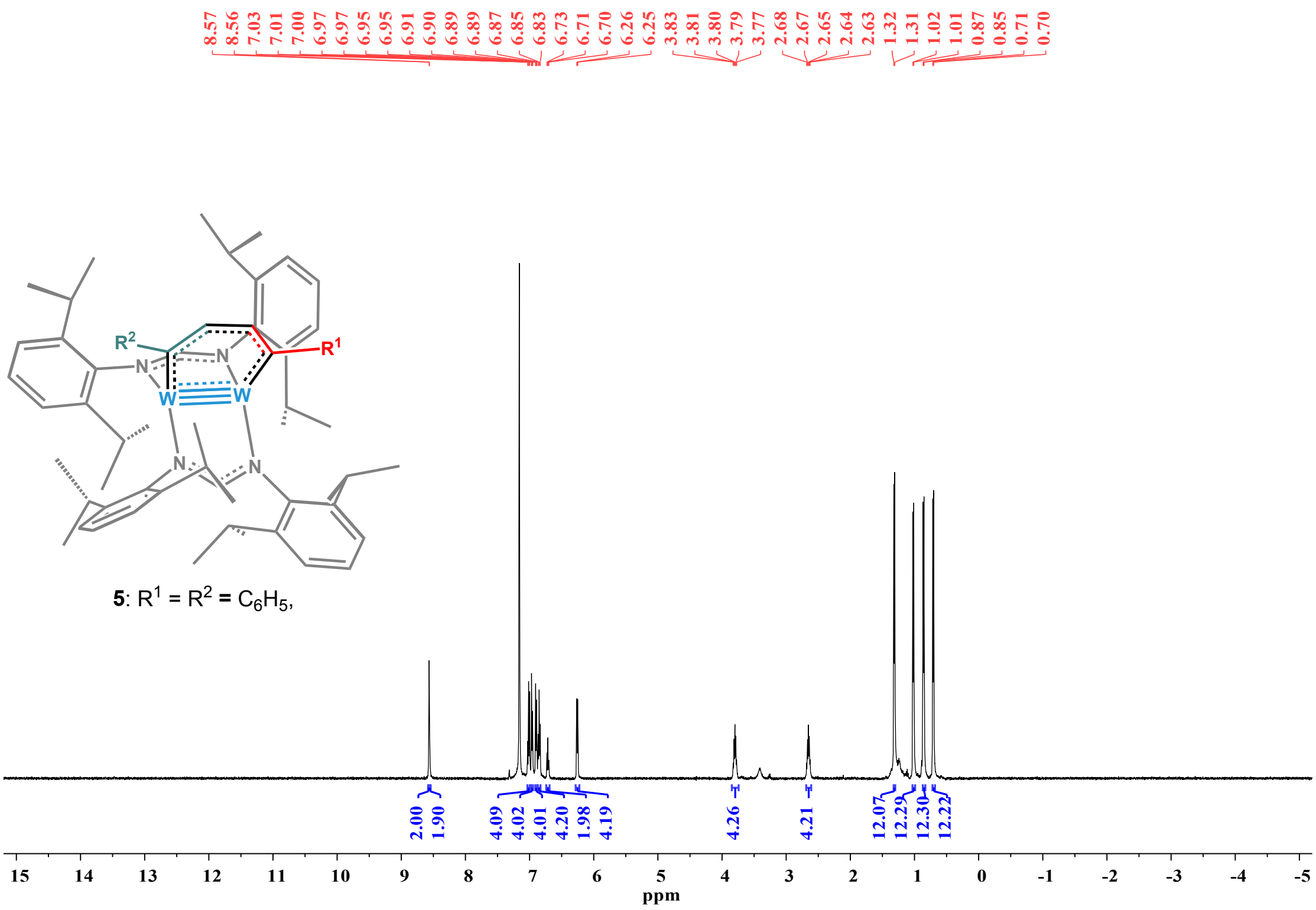

Figure. *S*39 ^{1}H NMR spectrum of **5** (500 MHz, C_6D_6, 298 K).

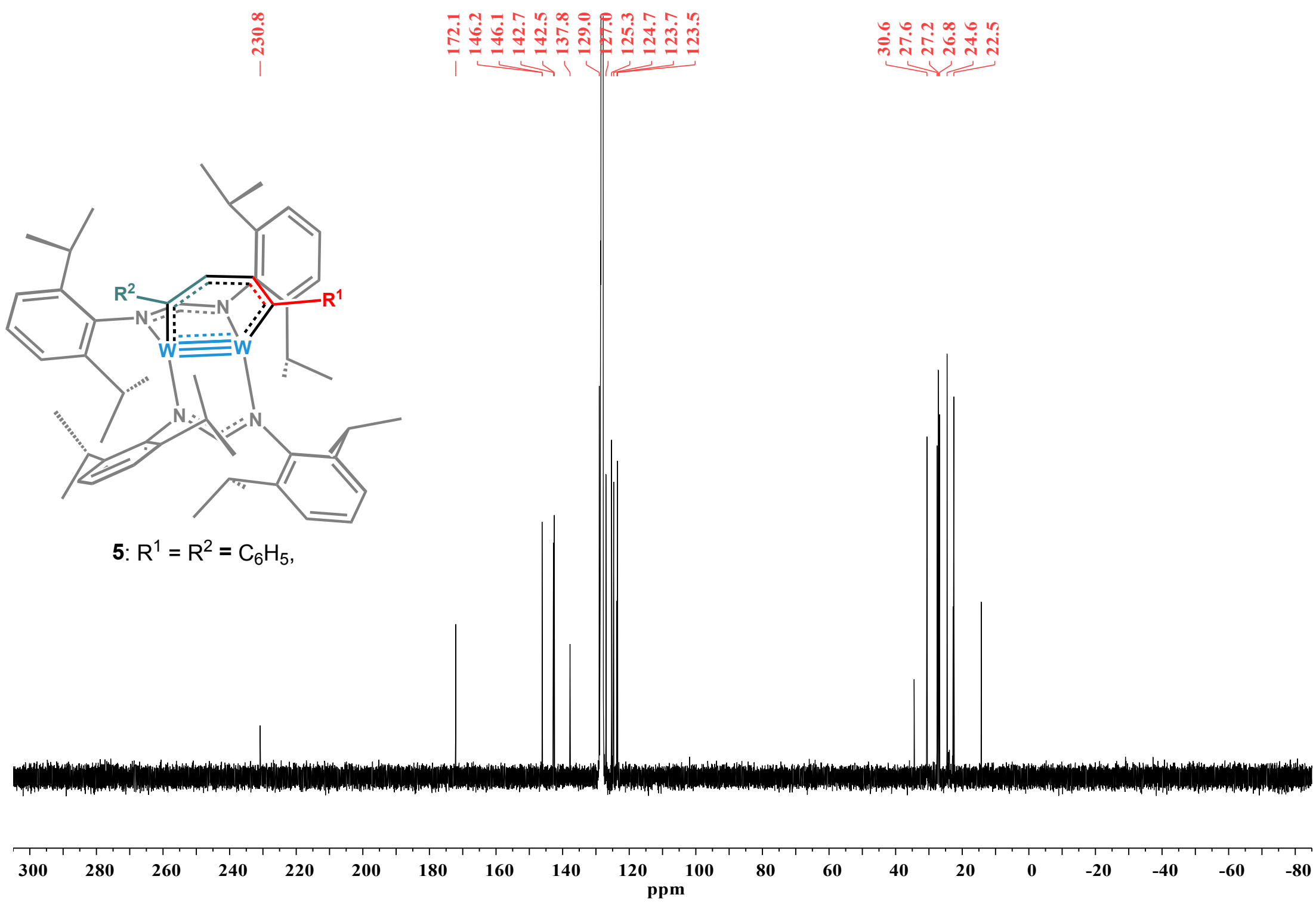

Figure. *S*40 $^{13}C\{^1H\}$ NMR spectrum of **5** (126 MHz, C_6D_6, 298 K).

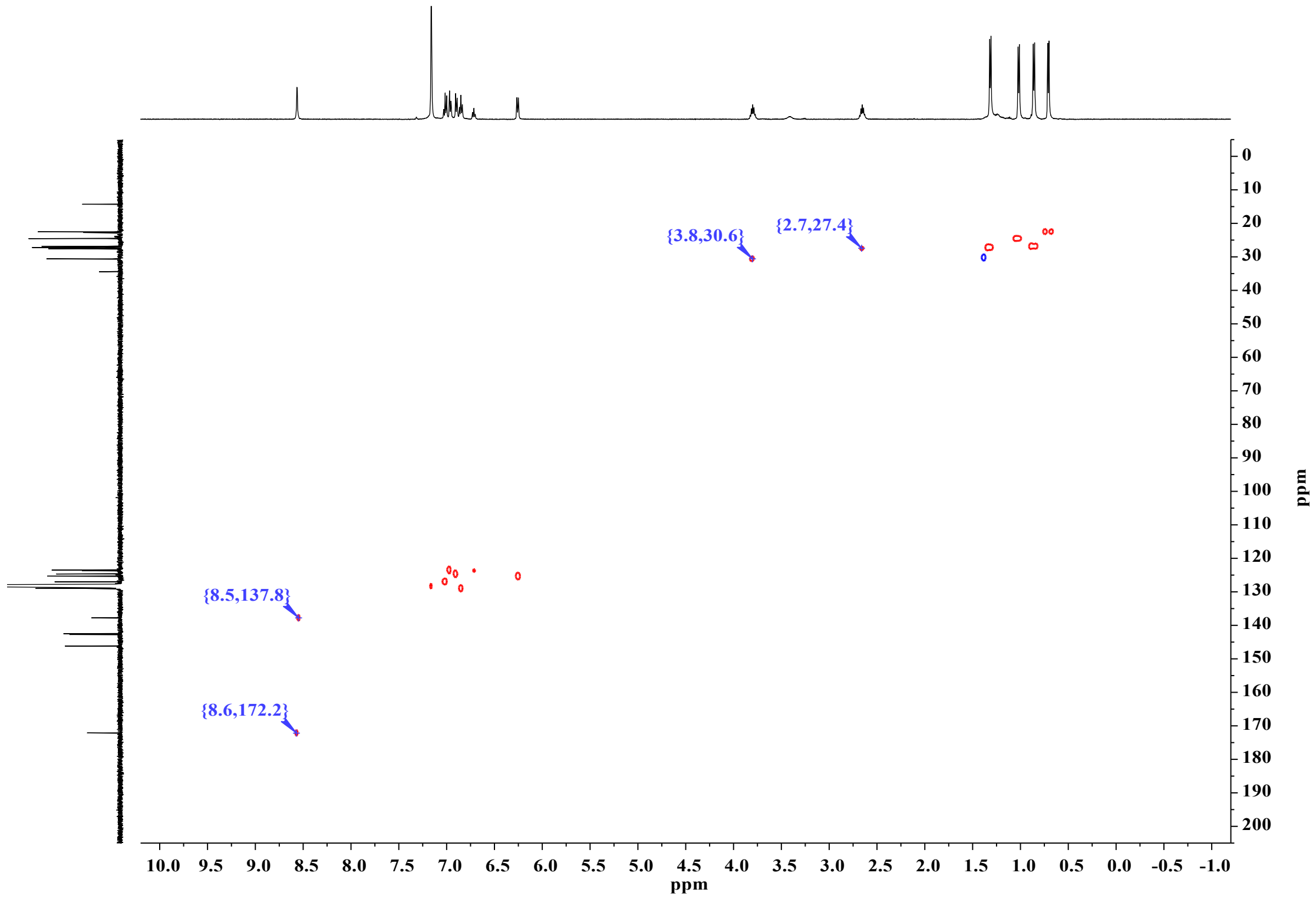

Figure. *S*41 HSQC (^1H-^{13}C) NMR spectrum of **5** (C_6D_6, 298 K).

4.6 $W_2(\mu\text{-}\kappa^2\text{-}1,4\text{-}(2\text{-MePh})_2C_4H_2)[\mu\text{-}\kappa^2\text{-HC(N-2,6-}^i\text{Pr}_2C_6H_3)_2]_2$ (6)

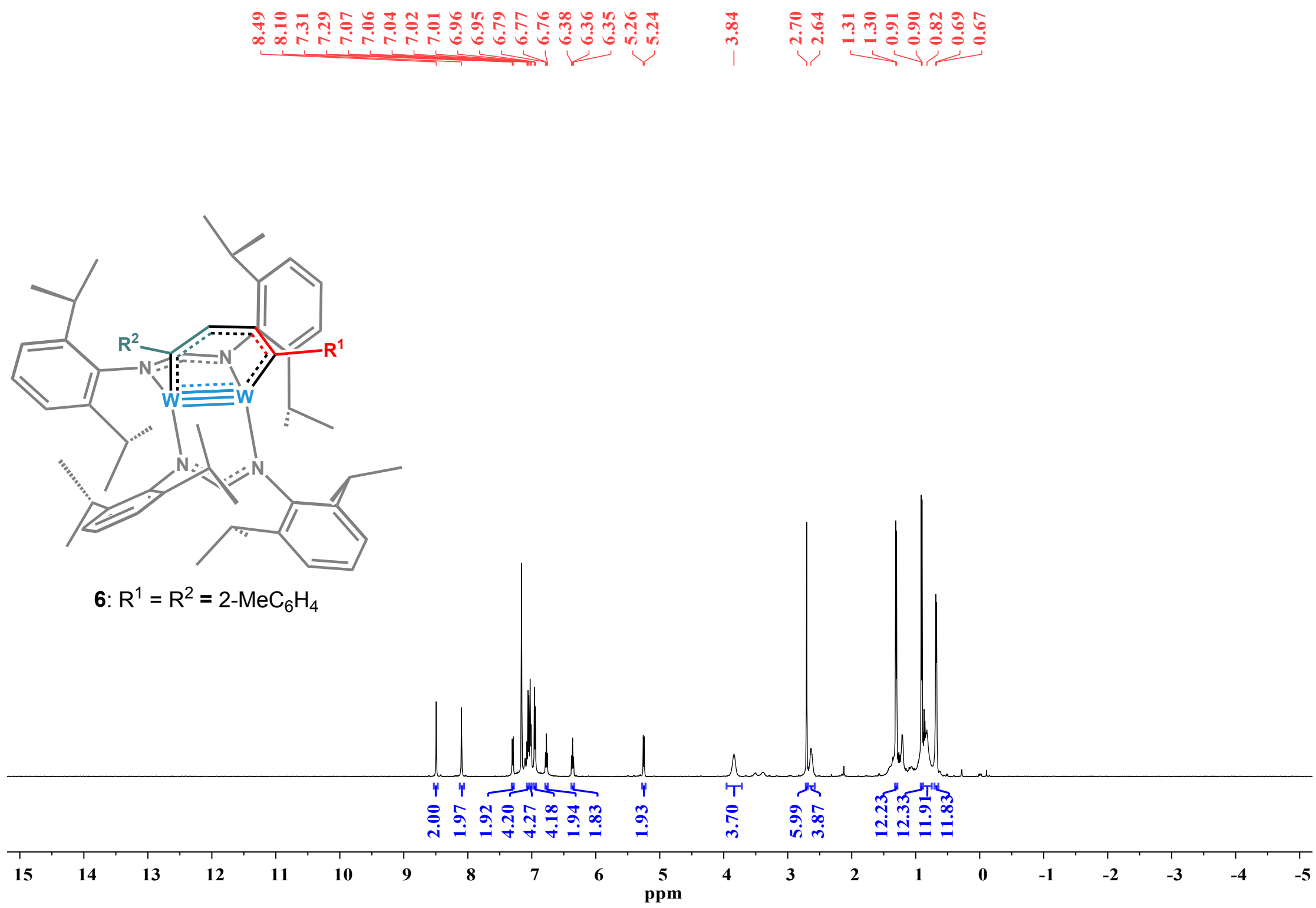

Figure. *S*42 ^{1}H NMR spectrum of **6** (500 MHz, C_6D_6, 333 K).

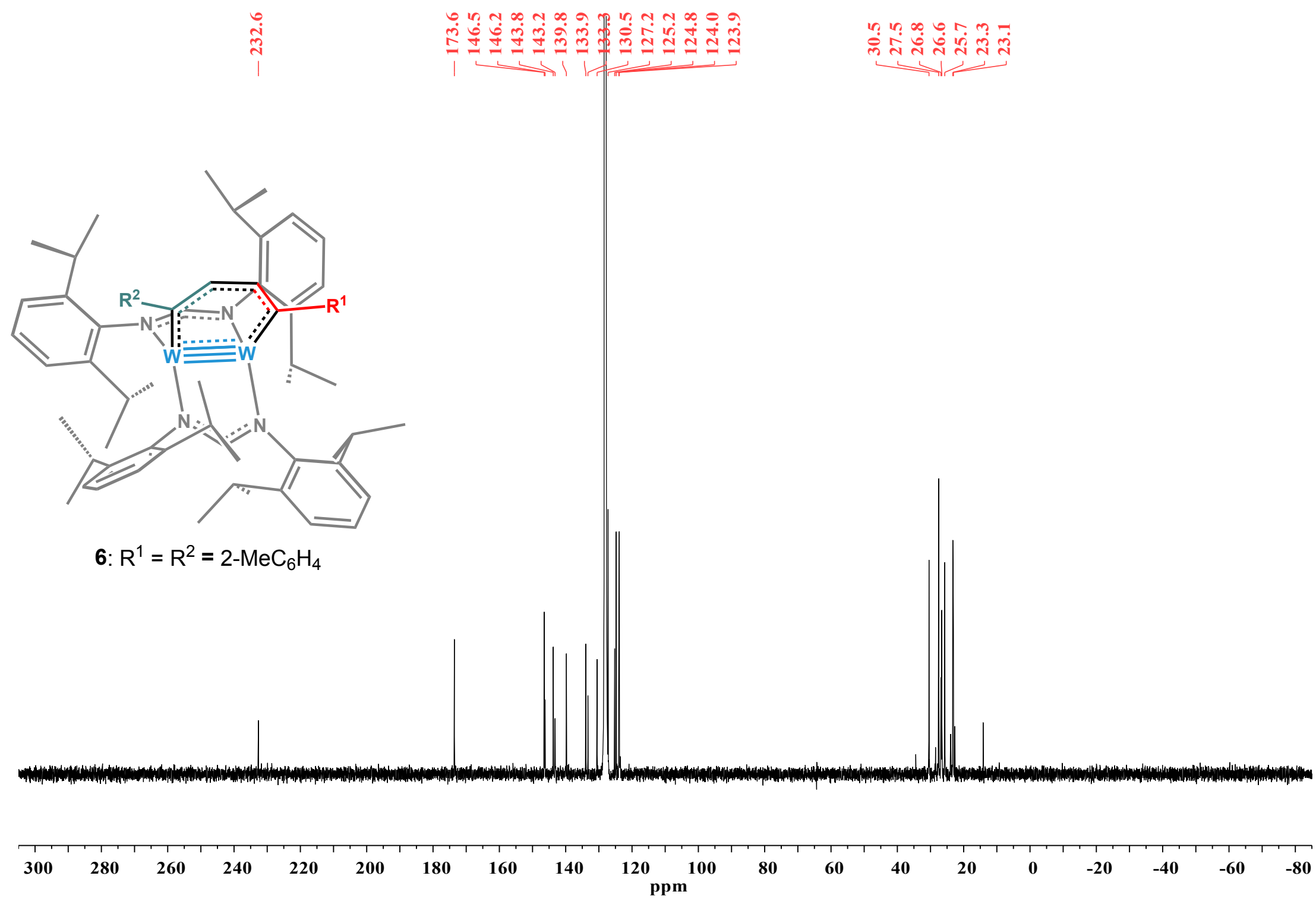

Figure. *S*43 $^{13}C\{^1H\}$ NMR spectrum of **6** (126 MHz, C_6D_6, 333 K).

4.7 $W_2(\mu\text{-}\kappa^2\text{-}1,4\text{-}(3\text{-}MePh)_2C_4H_2)[\mu\text{-}\kappa^2\text{-}HC(N\text{-}2,6\text{-}{}^iPr_2C_6H_3)_2]_2$ (7)

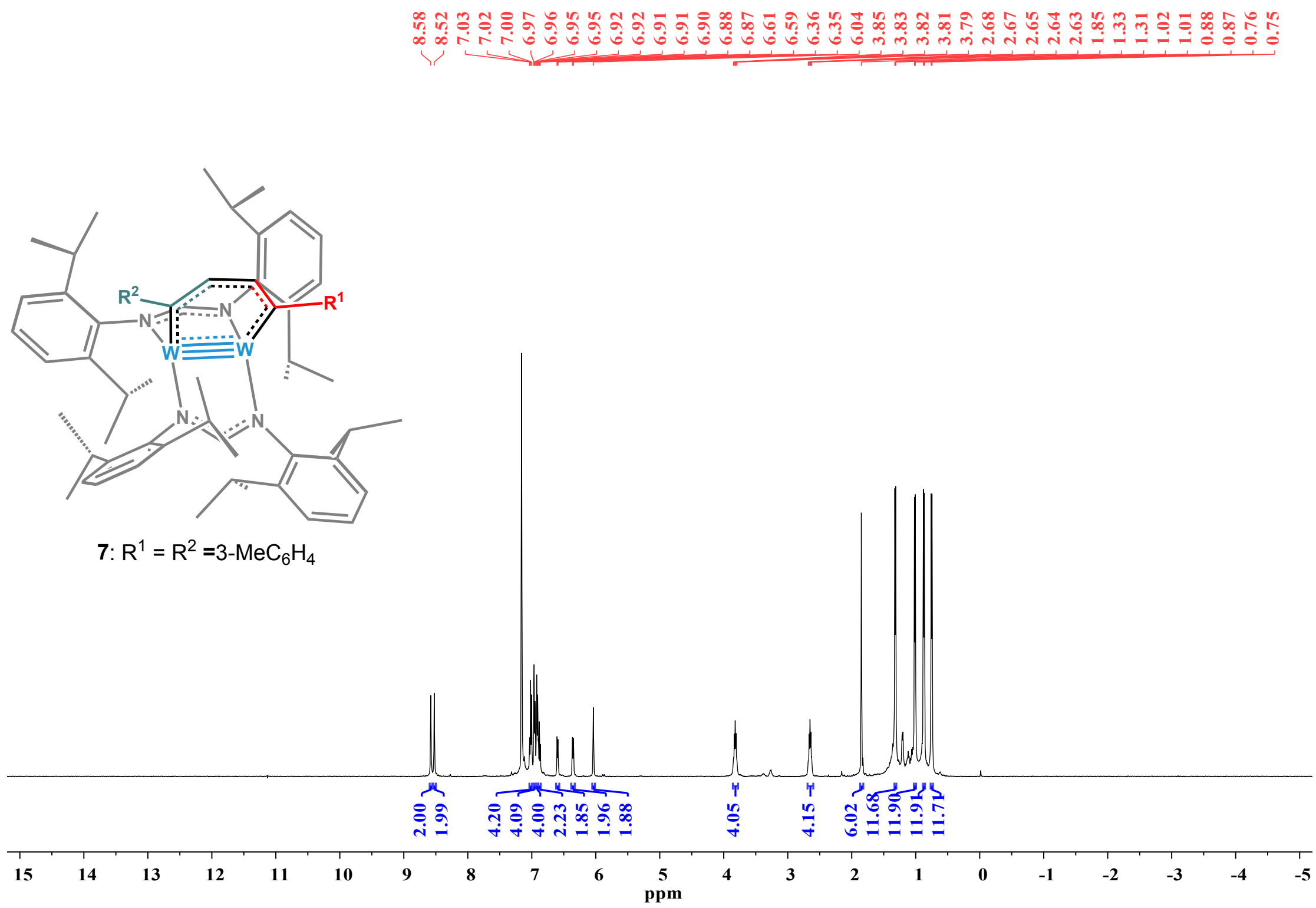

Figure. ***S*44** ^{1}H NMR spectrum of **7** (500 MHz, C_6D_6, 298 K).

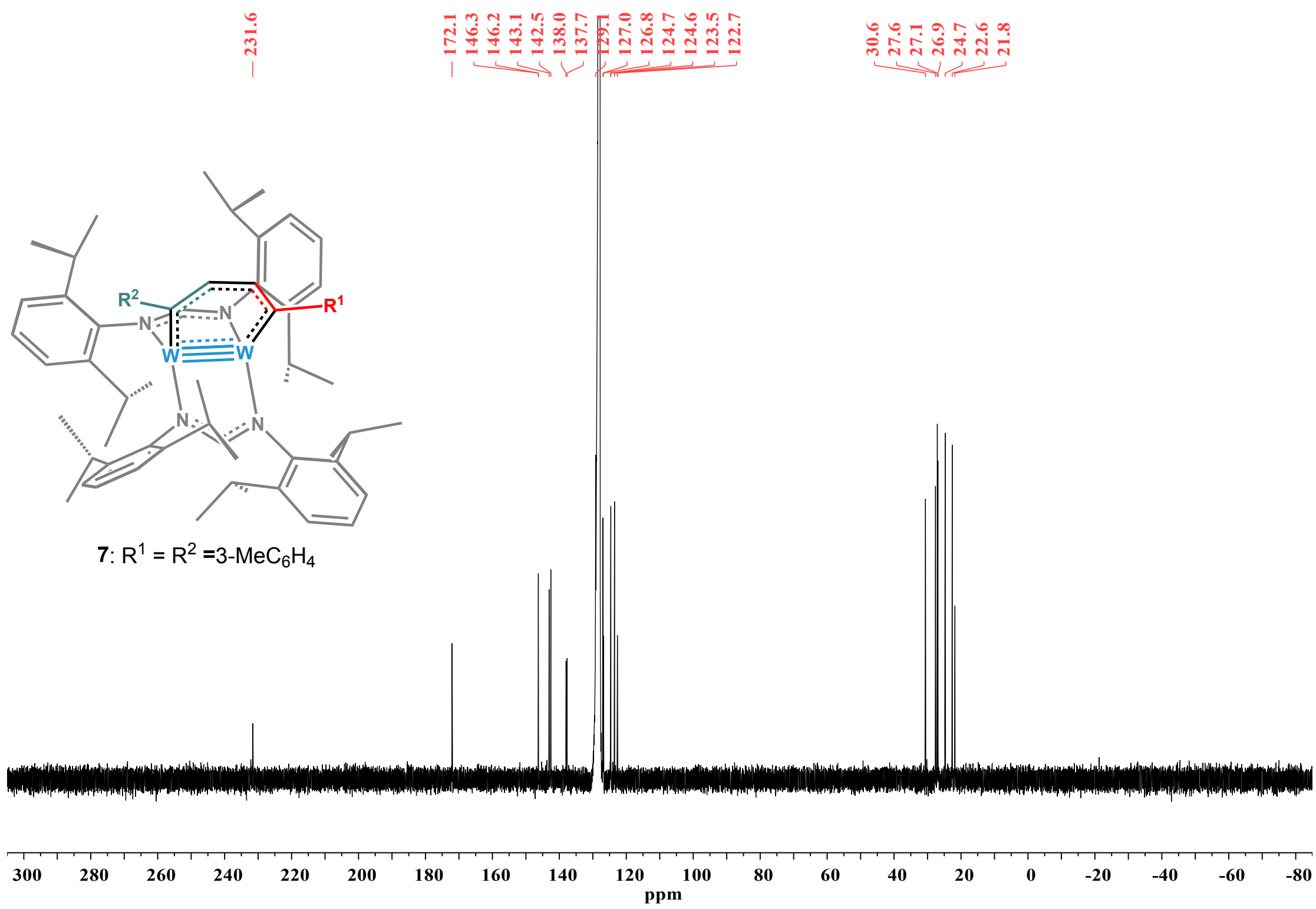

Figure. ***S*45** $^{13}C\{^1H\}$ NMR spectrum of **7** (126 MHz, C_6D_6, 298 K).

4.8 $W_2(\mu\text{-}\kappa^2\text{-}1,4\text{-}(4\text{-}MePh)_2C_4H_2)[\mu\text{-}\kappa^2\text{-}HC(N\text{-}2,6\text{-}{}^iPr_2C_6H_3)_2]_2$ (8)

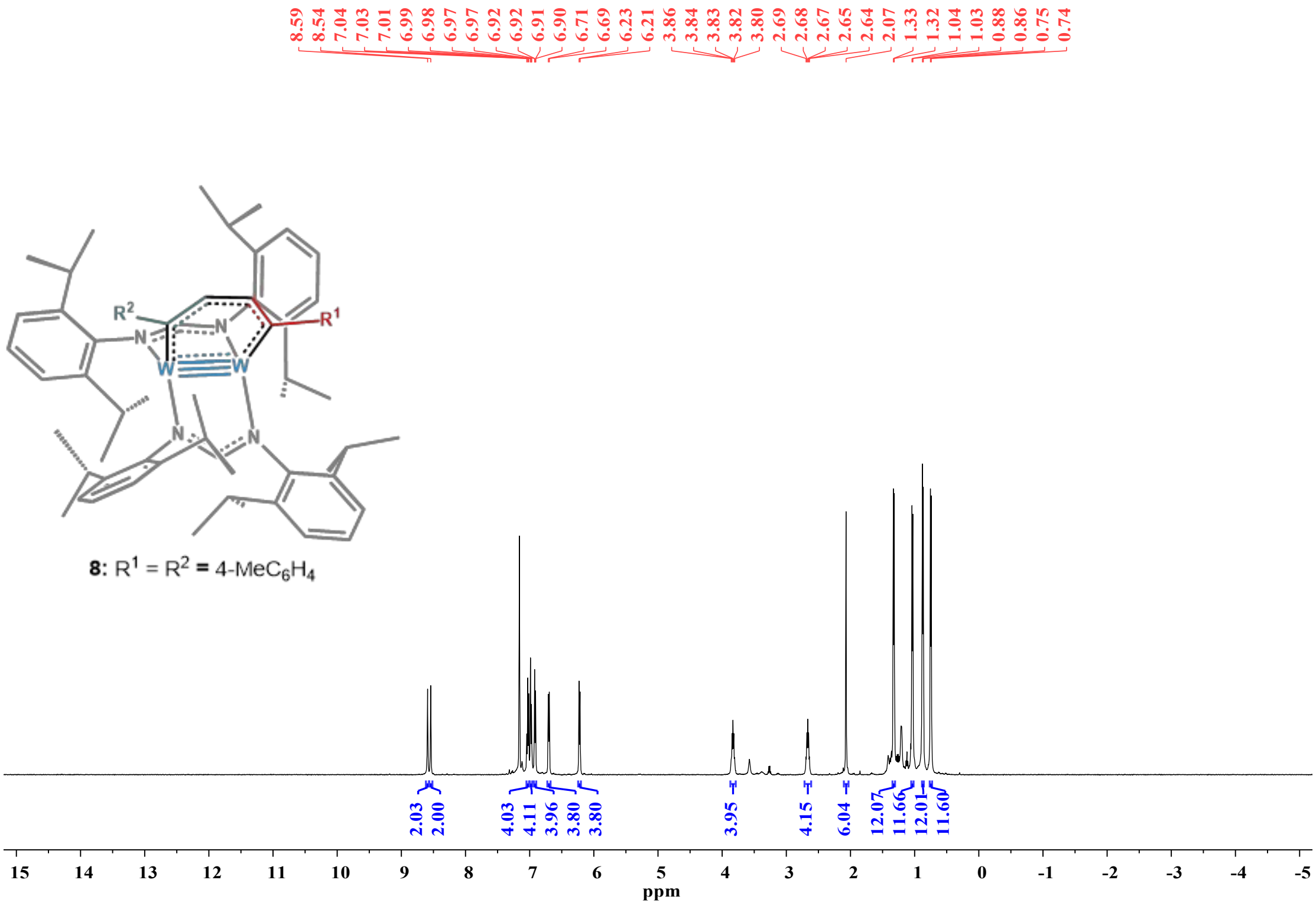

Figure. ***S*46** ^1H NMR spectrum of **8** (500 MHz, C_6D_6, 298 K).

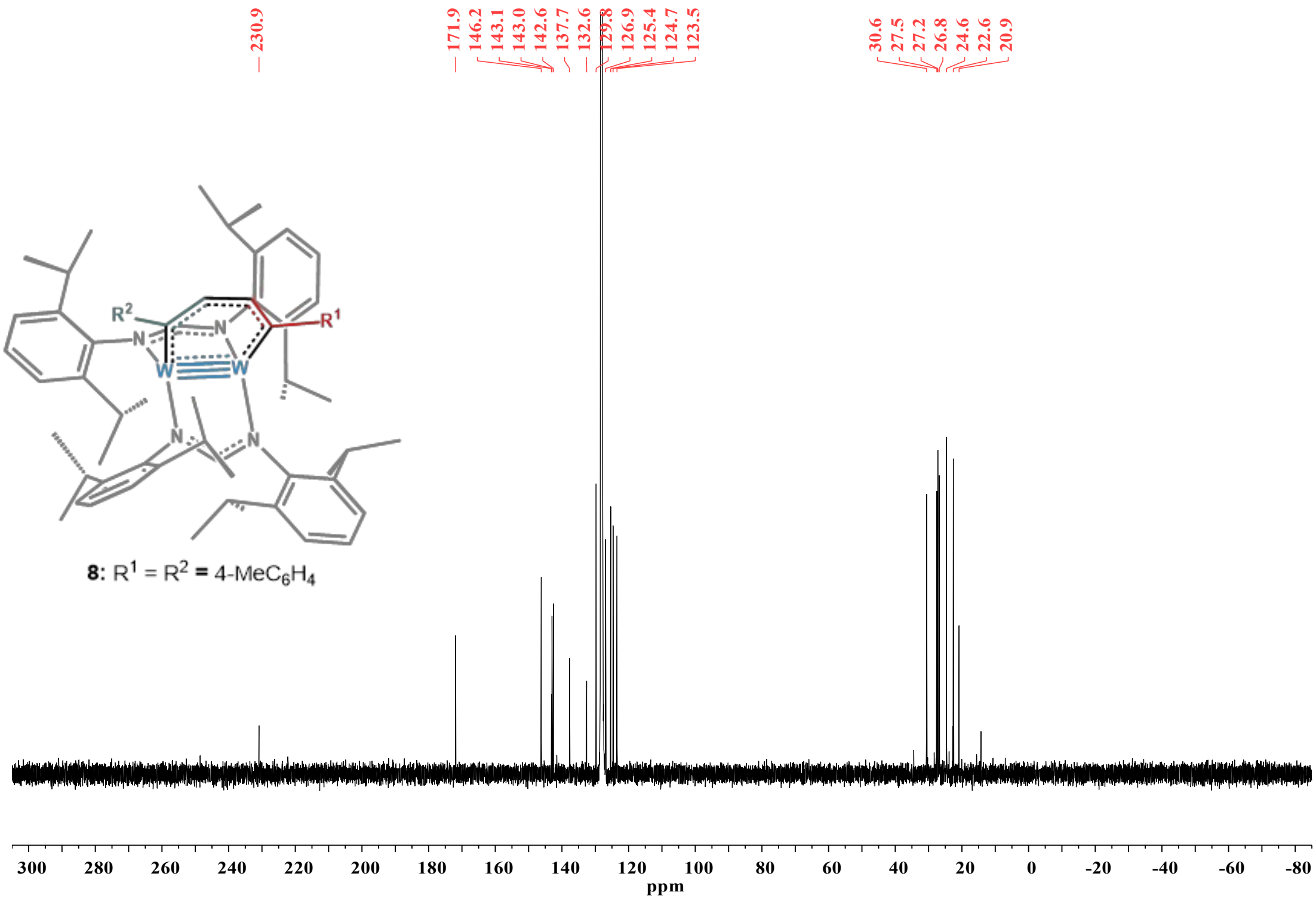

Figure. ***S*47** $^{13}C\{^1H\}$ NMR spectrum of **8** (126 MHz, C_6D_6, 298 K).

4.9 $W_2(\mu\text{-}\kappa^2\text{-}1,4\text{-}(4\text{-}OMePh)_2C_4H_2)[\mu\text{-}\kappa^2\text{-}HC(N\text{-}2,6\text{-}^iPr_2C_6H_3)_2]_2$ (9)

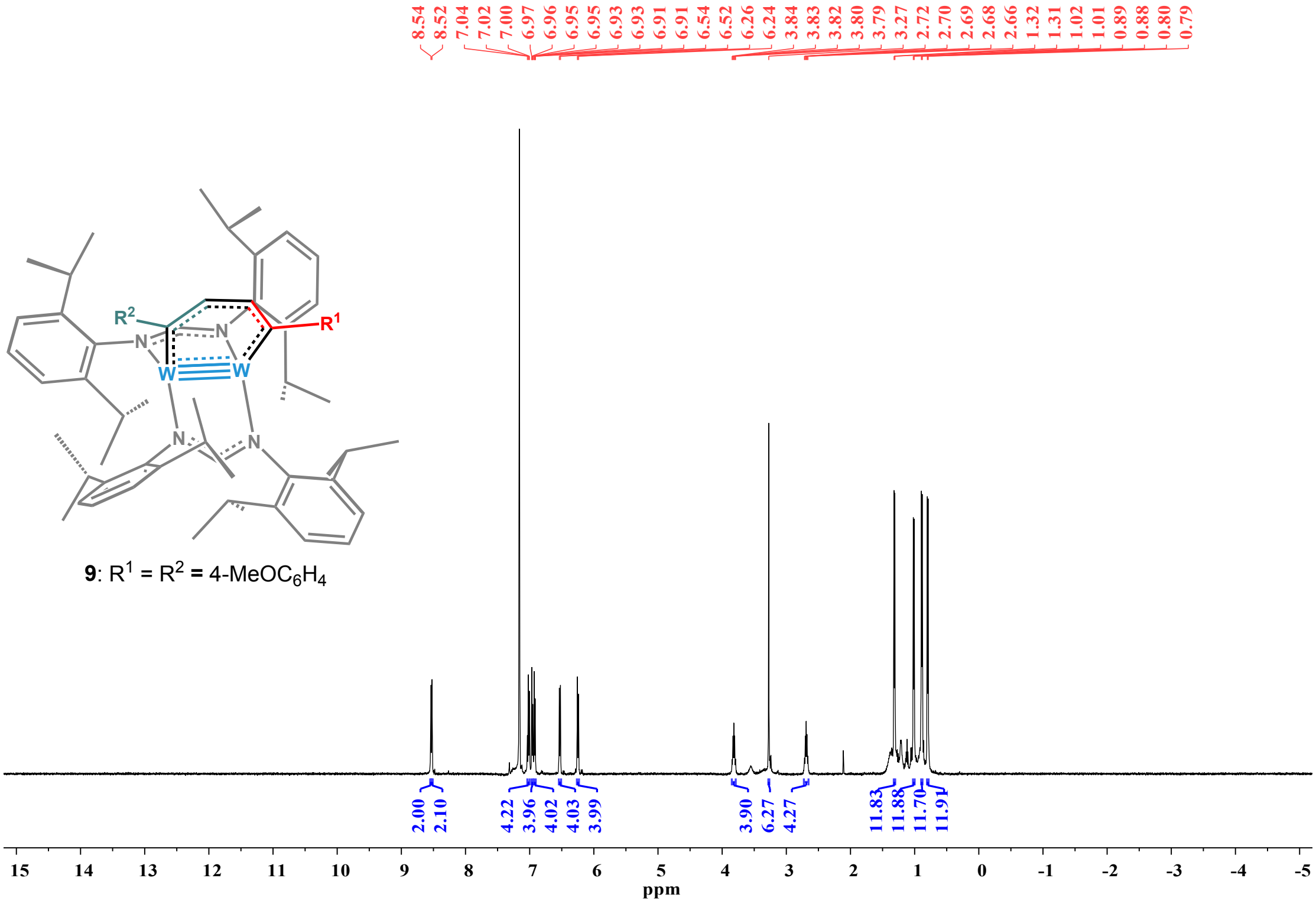

Figure. *S*48 ^1H NMR spectrum of **9** (500 MHz, C_6D_6, 298 K).

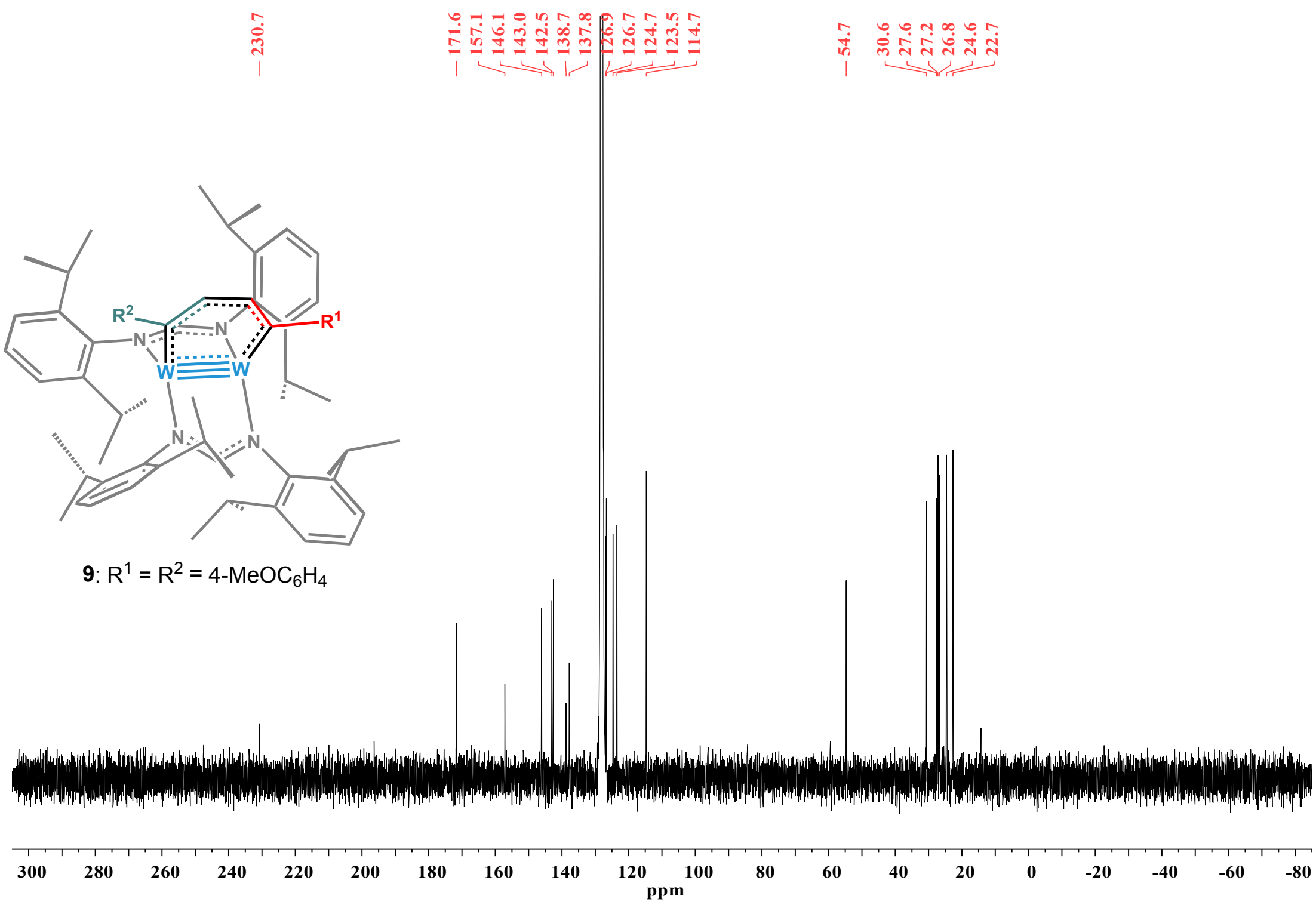

Figure. *S*49 $^{13}C\{^1H\}$ NMR spectrum of **9** (126 MHz, C_6D_6, 298 K).

4.10 $W_2(\mu\text{-}\kappa^2\text{-}1,4\text{-}(4\text{-FPh})_2C_4H_2)[\mu\text{-}\kappa^2\text{-HC(N-2,6-}^i\text{Pr}_2C_6H_3)_2]_2$ (10)

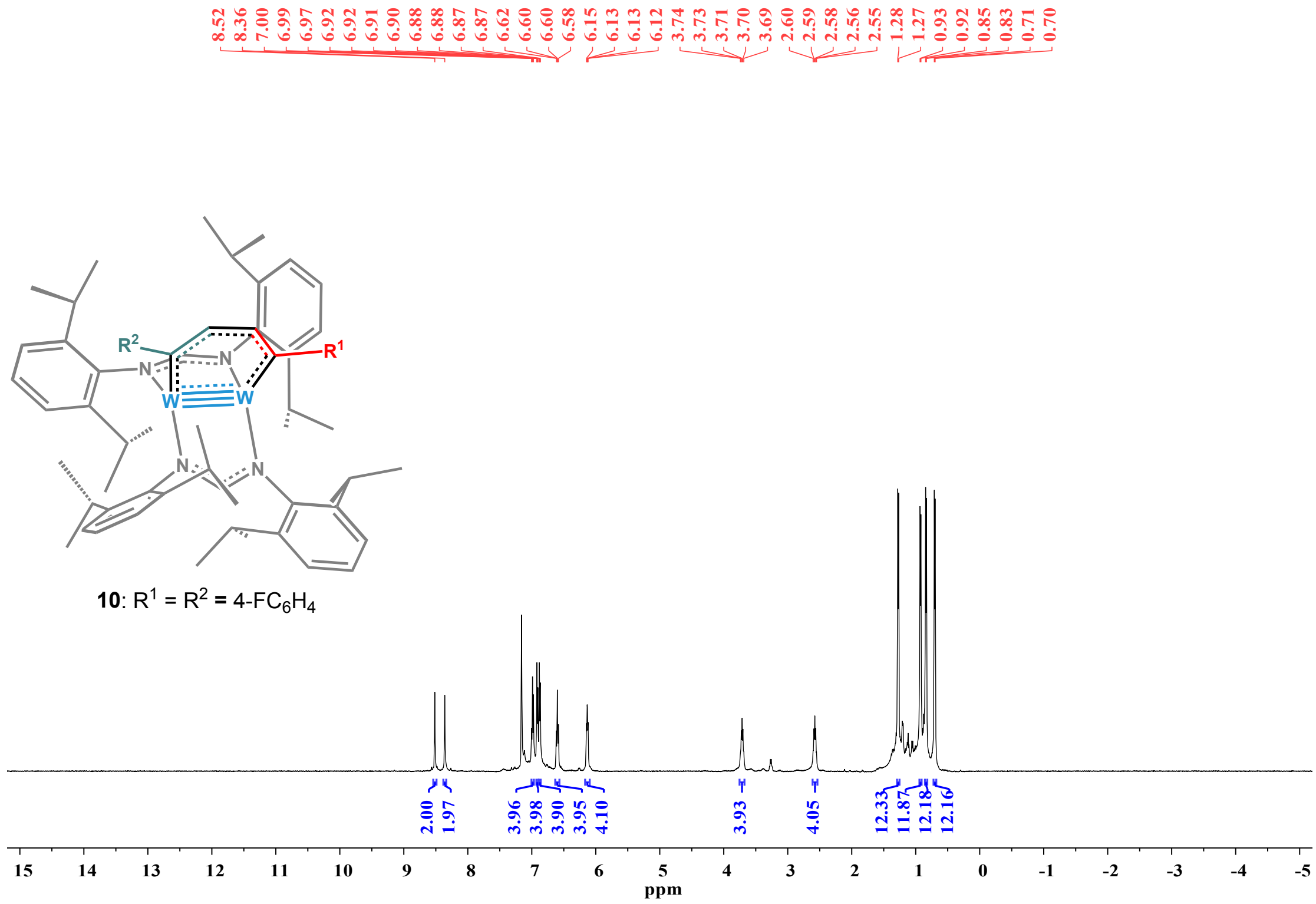

Figure. *S*50 ^{1}H NMR spectrum of **10** (500 MHz, C_6D_6, 298 K).

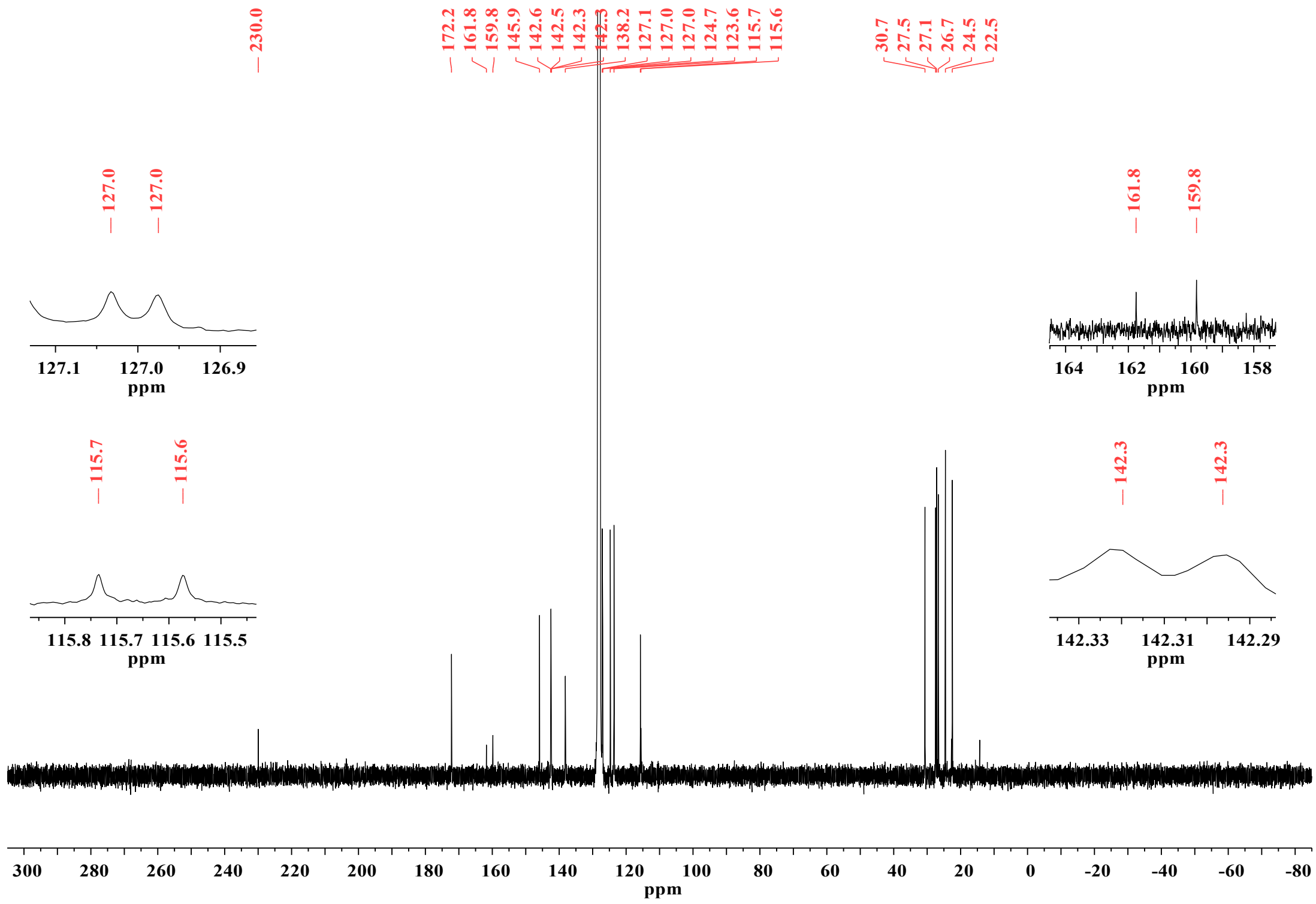

Figure. *S*51 $^{13}C\{^1H\}$ NMR spectrum of **10** (126 MHz, C_6D_6, 298 K).

4.11 $W_2(\mu\text{-}\kappa^2\text{-}1,4\text{-}(n\text{-}C_3H_7)_2C_4H_2)[\mu\text{-}\kappa^2\text{-}HC(N\text{-}2,6\text{-}{}^{i}Pr_2C_6H_3)_2]_2$ (11)

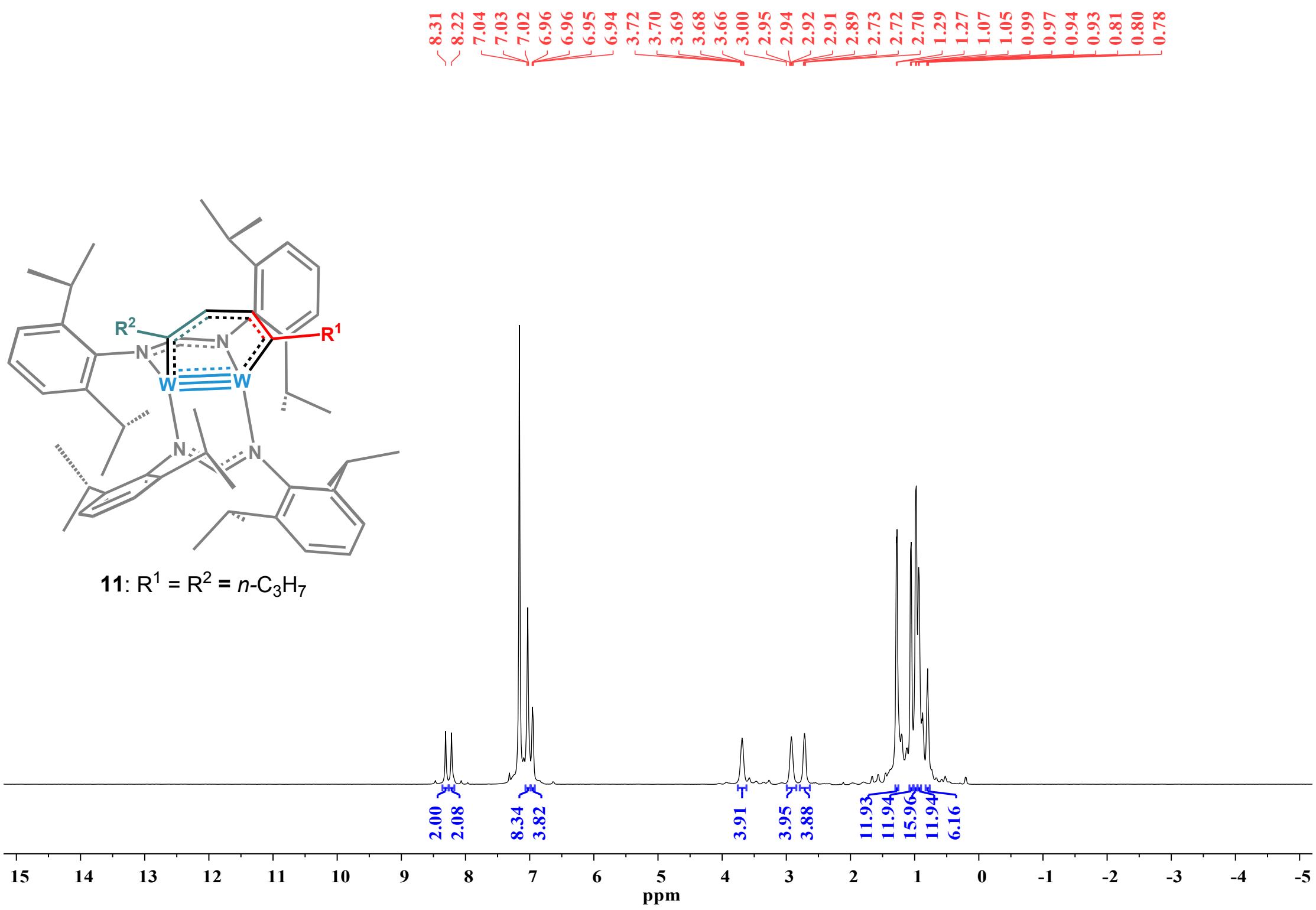

Figure. *S*52 ^{1}H NMR spectrum of **11** (500 MHz, C_6D_6, 298 K).

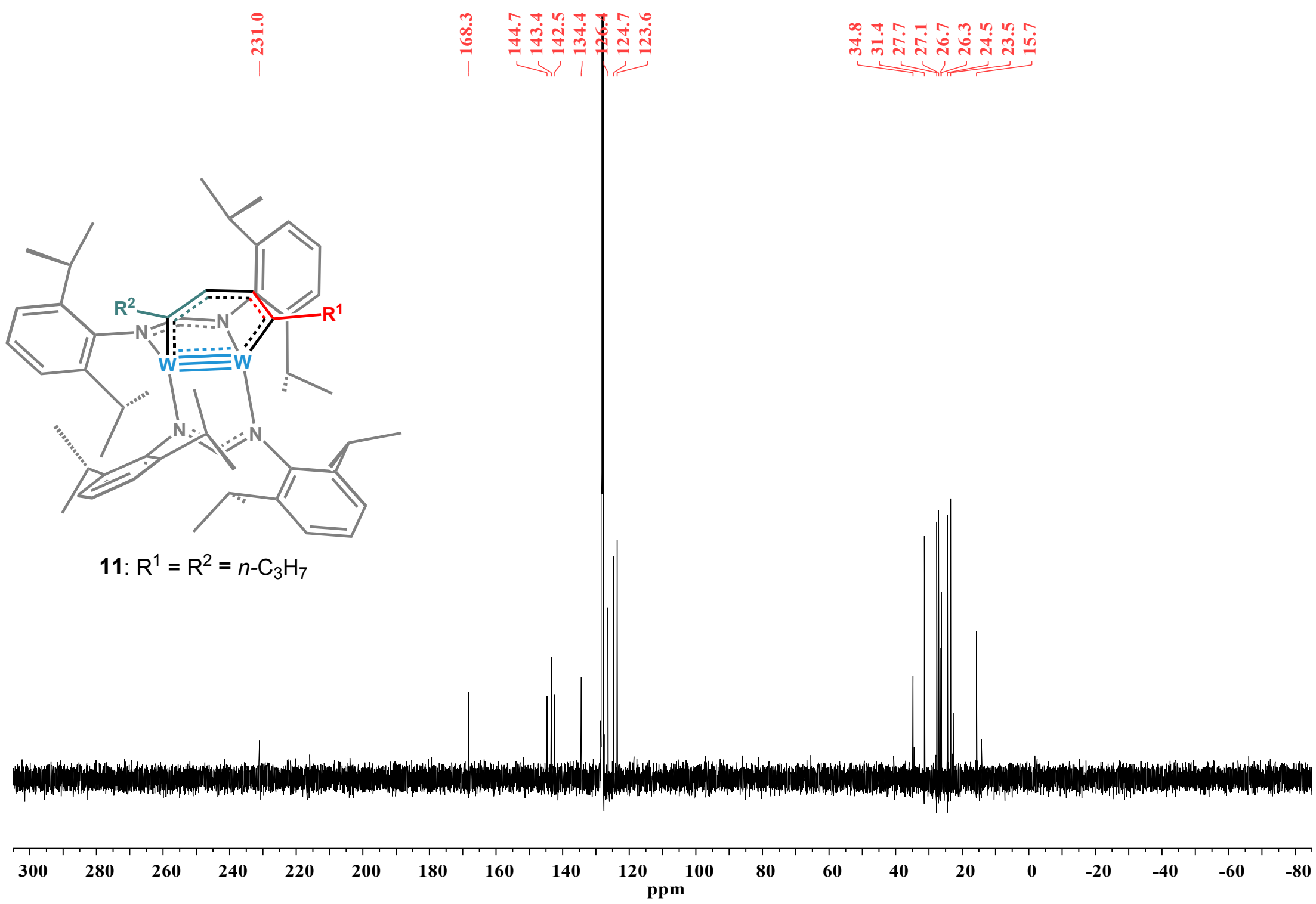

Figure. *S*53 $^{13}C\{^1H\}$ NMR spectrum of **11** (126 MHz, C_6D_6, 298 K).

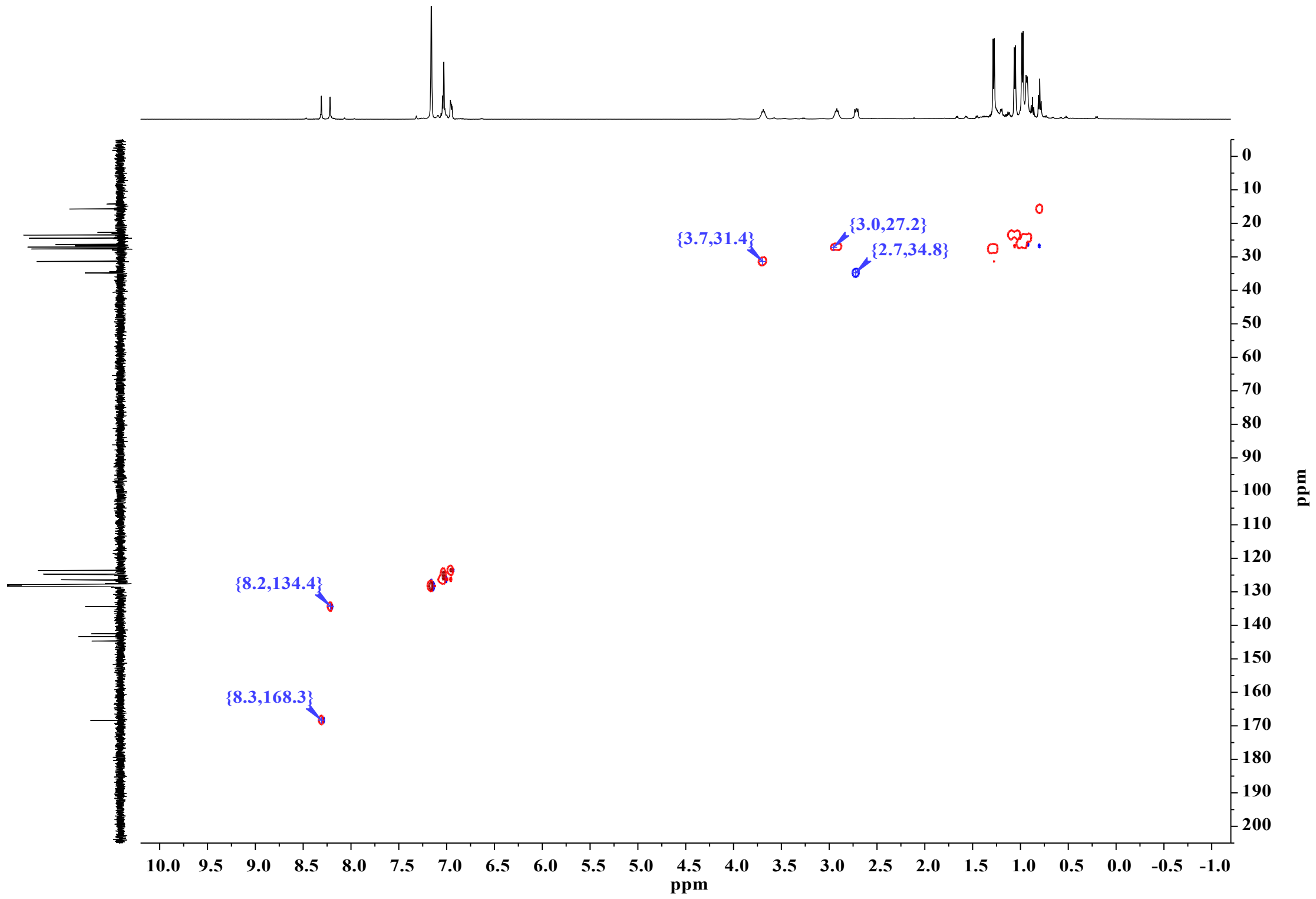

Figure. *S*54 HSQC (^1H-^{13}C) NMR spectrum of **11** (C_6D_6, 298 K).

4.12 $W_2Cl_2[\mu\text{-}\eta^2\text{:}\eta^2\text{-}1,4\text{-}Ph_2C_4H_2][\mu\text{-}\kappa^2\text{-}HC(N\text{-}2,6\text{-}{}^iPr_2C_6H_3)_2]_2$ (12)

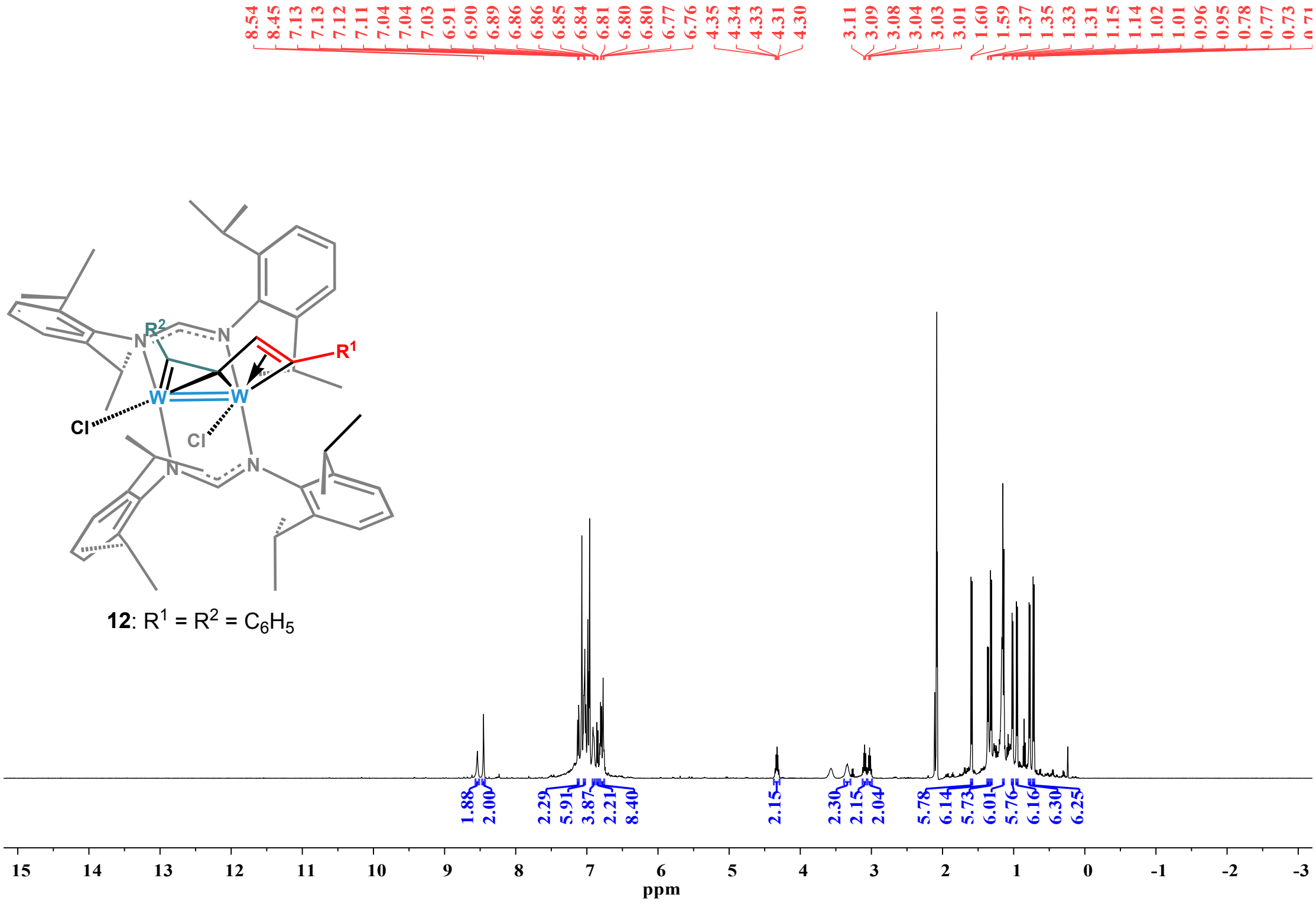

Figure. *S*55 ^{1}H NMR spectrum of **12** (500 MHz, d_8-toluene, 333 K).

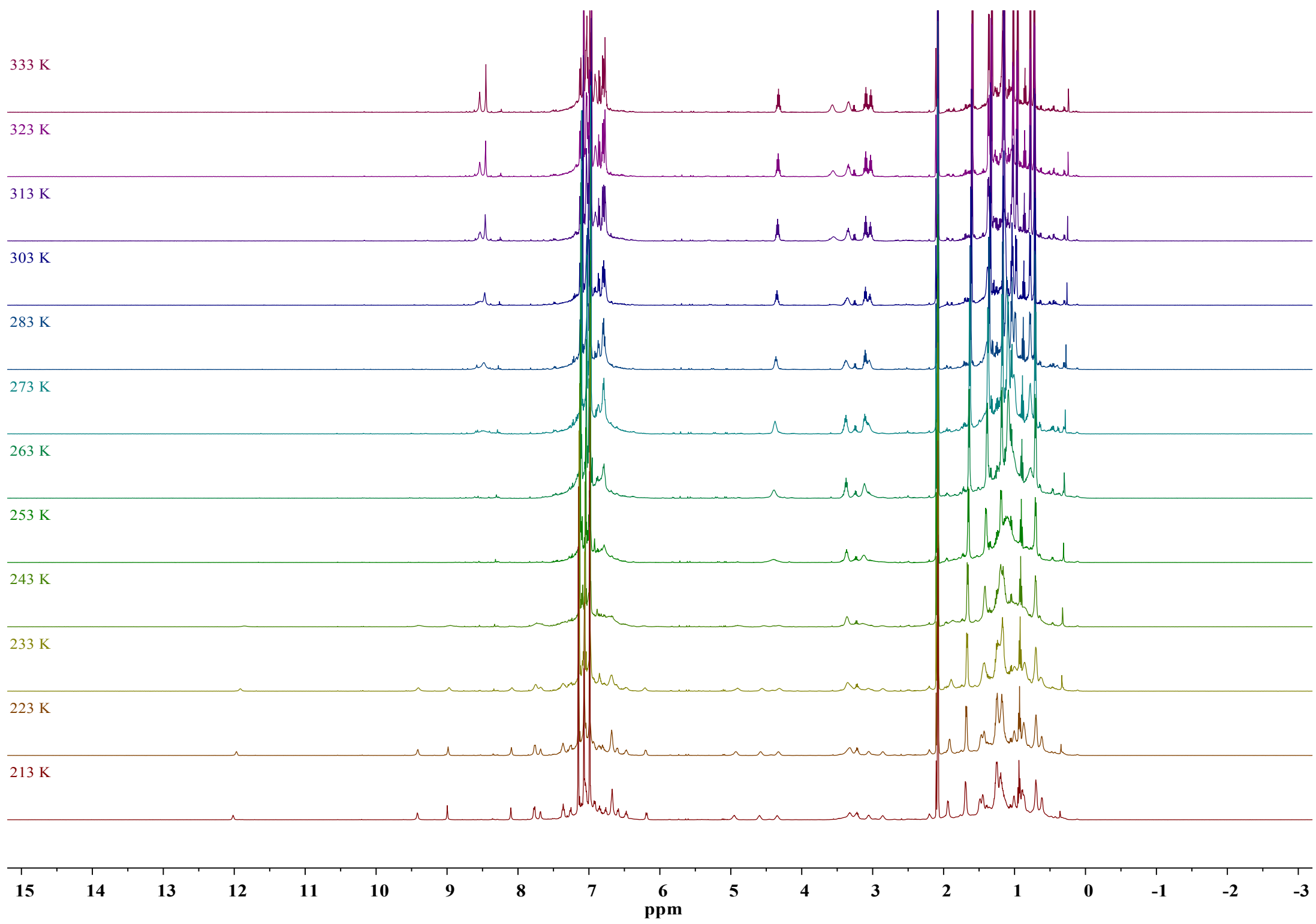

Figure. *S*56 Variable-temperature ^{1}H NMR spectrum of **12** (500 MHz, d_8-toluene).

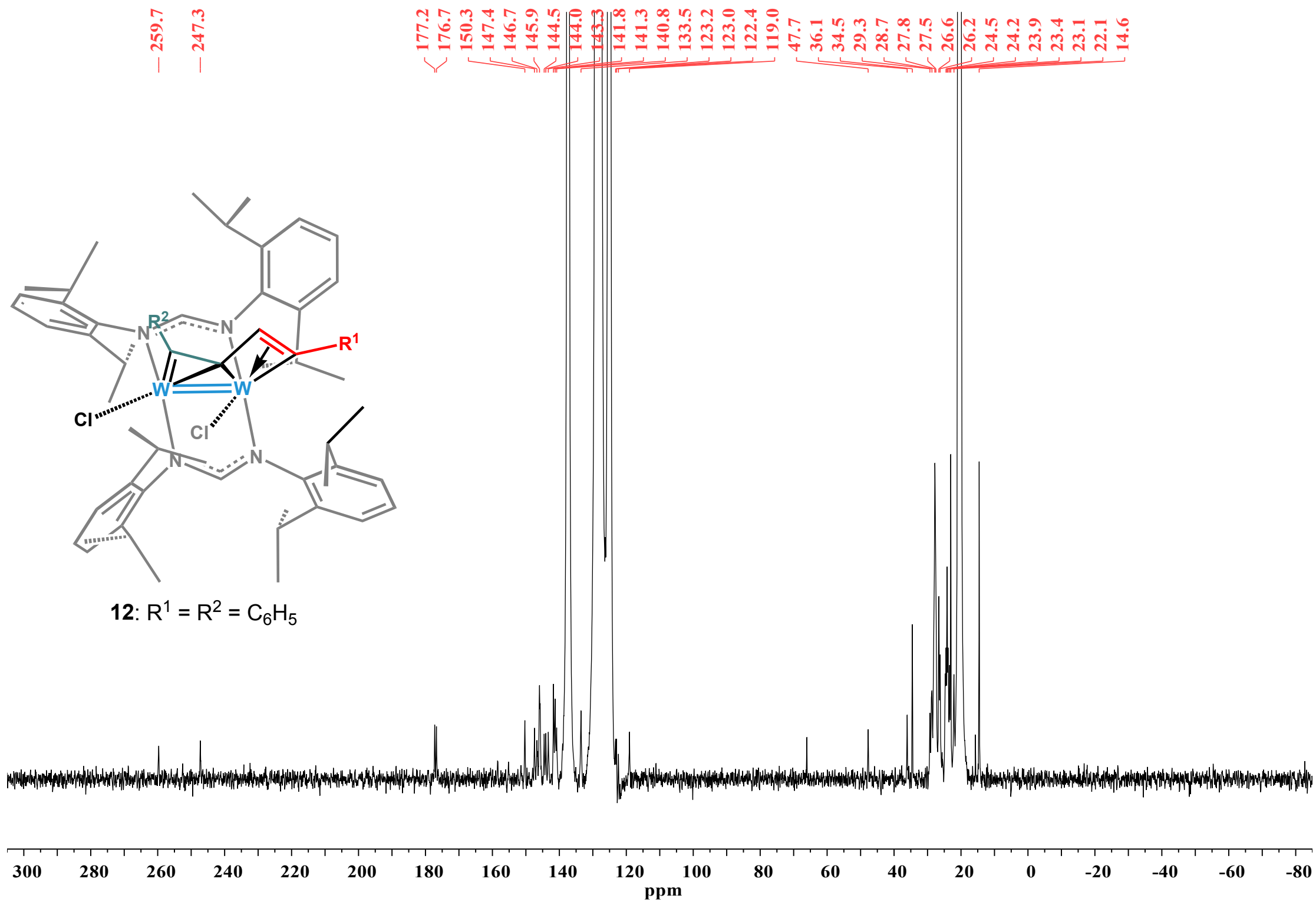

Figure. *S*57 $^{13}C\{^{1}H\}$ NMR spectrum of **12** (126 MHz, d_8-toluene, 333 K).

4.13 $W_2Cl_2[\mu$-η^2:η^2-1,4-(2-MePh)$_2C_4H_2][\mu$-κ^2-HC(N-2,6-^{i}Pr$_2C_6H_3)_2]_2$ (13)

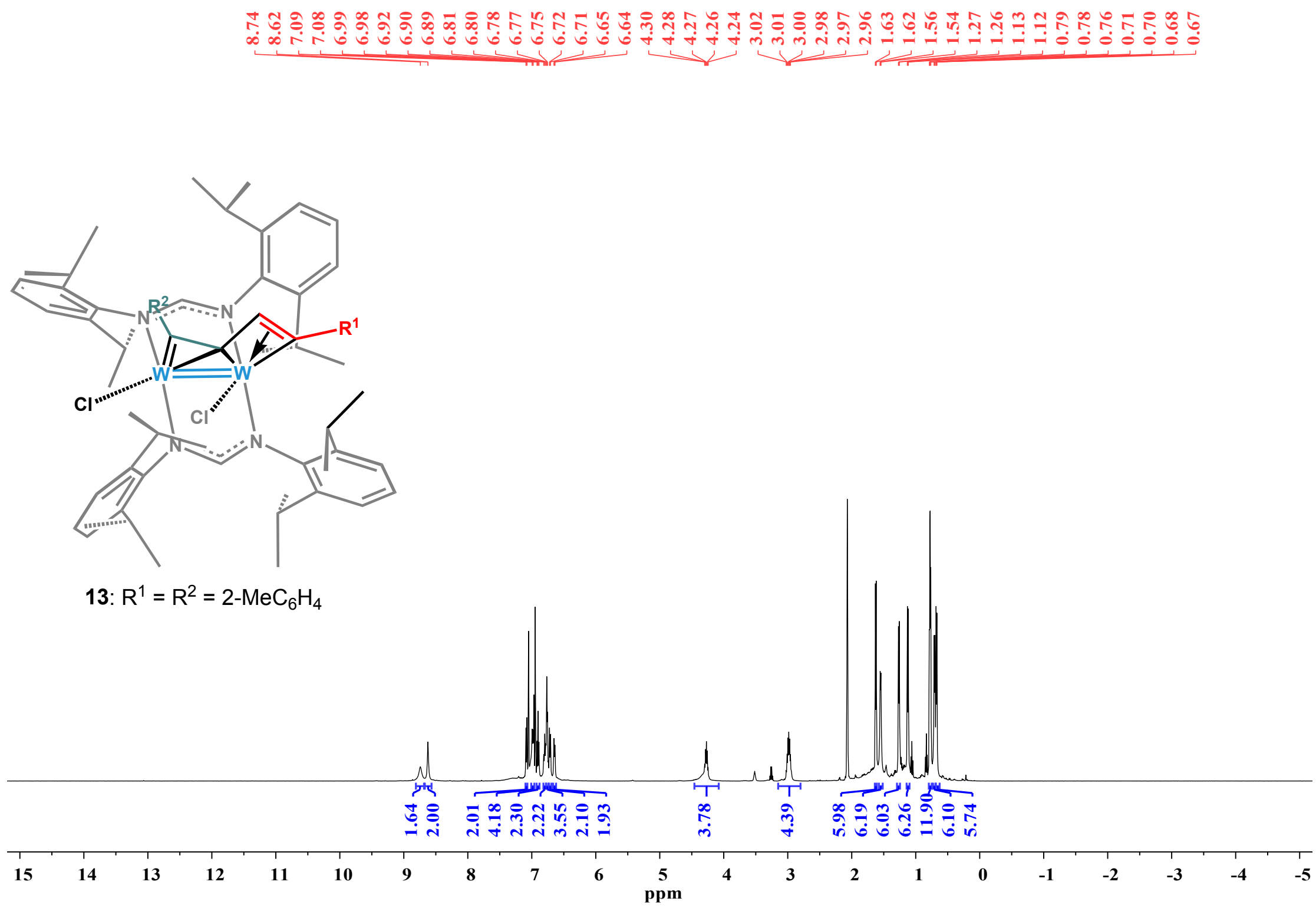

Figure. *S*58 ^{1}H NMR spectrum of **13** (500 MHz, d_8-toluene, 353 K).

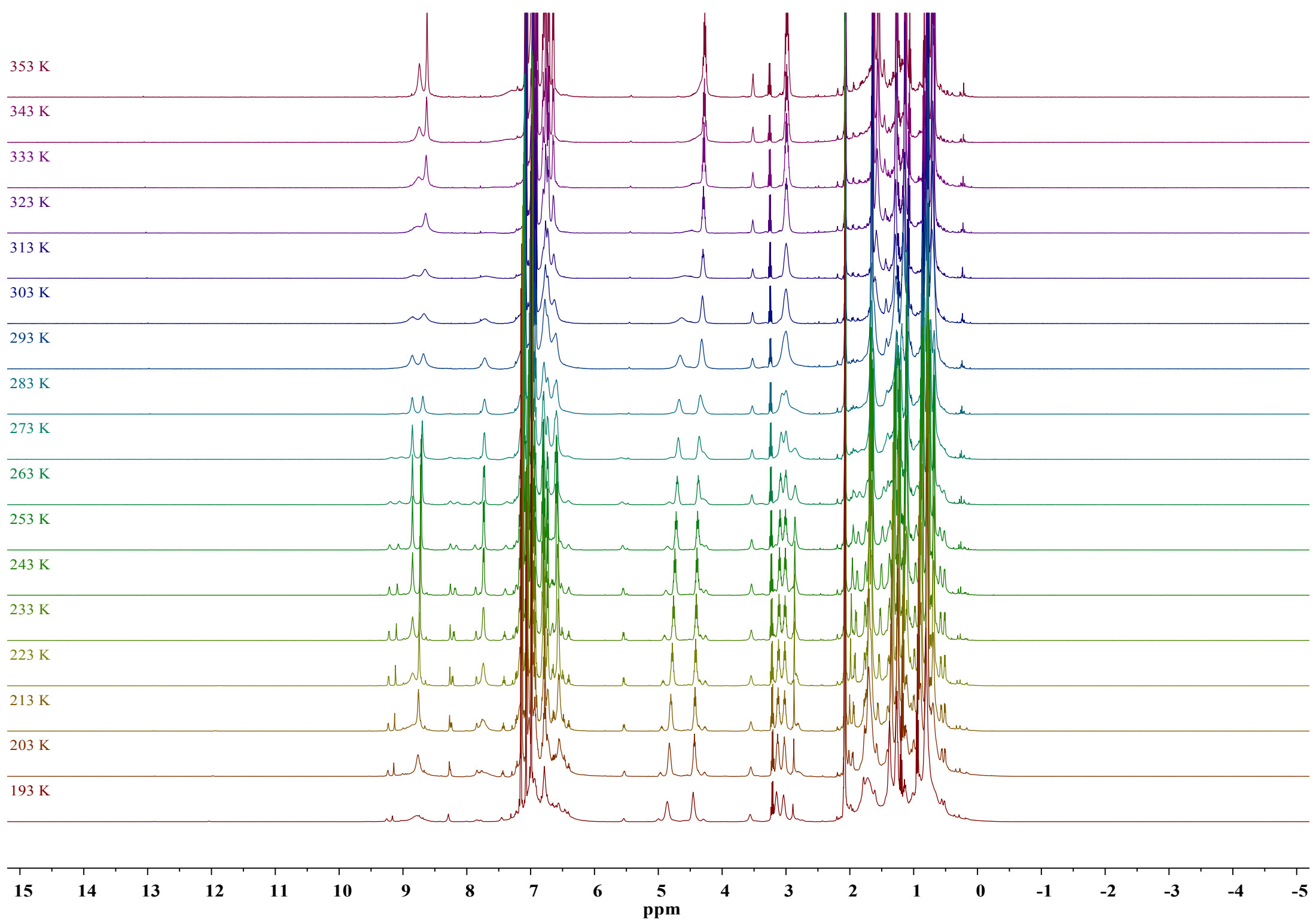

Figure. *S*59 Variable-temperature ^{1}H NMR spectrum of **13** (500 MHz, d_8-toluene).

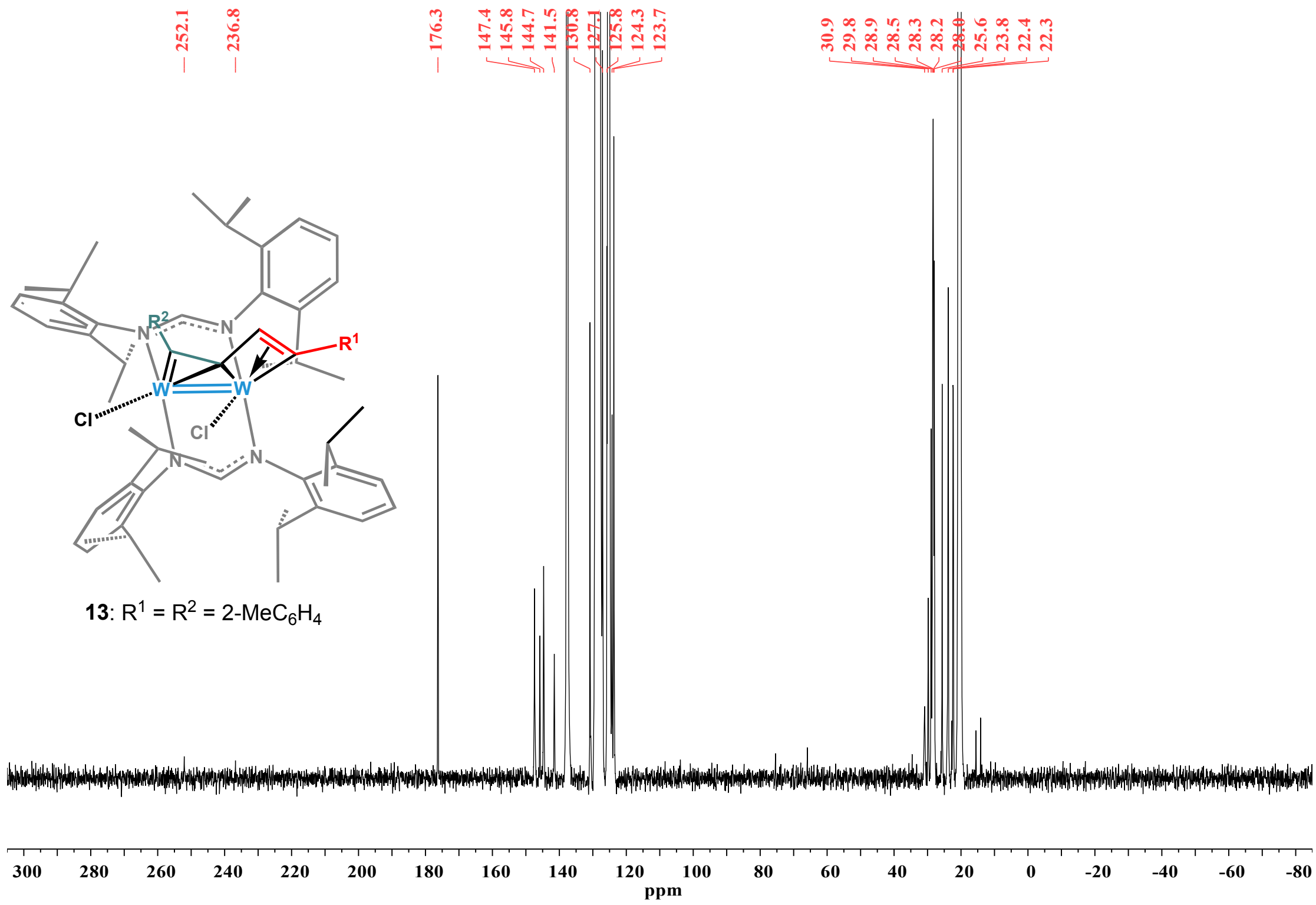

Figure. *S*60 $^{13}C\{^{1}H\}$ NMR spectrum of **13** (126 MHz, d_8-toluene, 353 K).

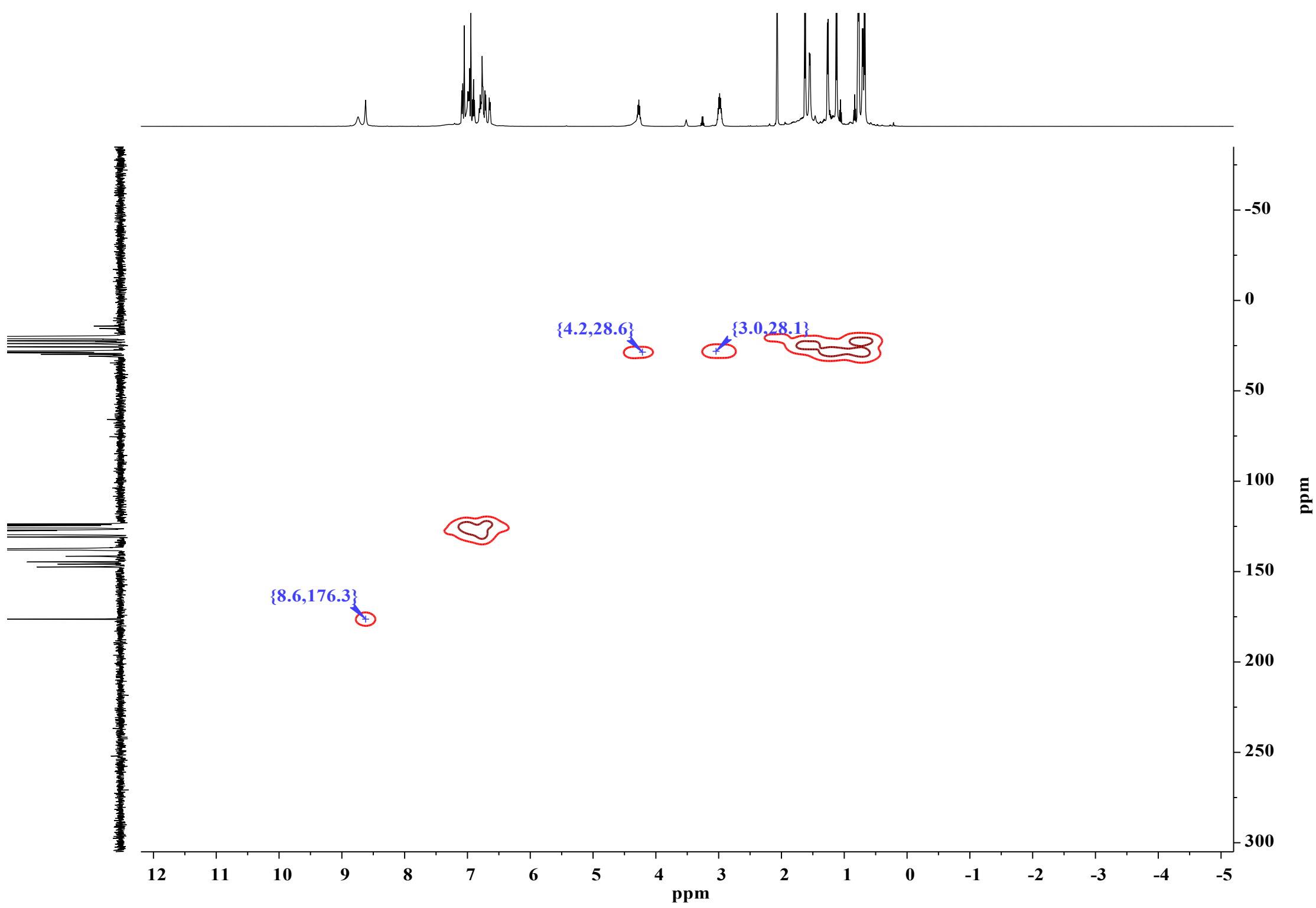

Figure. *S*61 HSQC (^{1}H-^{13}C) NMR spectrum of **13** (d_8-toluene, 353 K).

4.14 $W_2Cl_2[\mu-\eta^2:\eta^2-1,4-(4-OMePh)_2C_4H_2][\mu-\kappa^2-HC(N-2,6-{}^iPr_2C_6H_3)_2]_2$ (14)

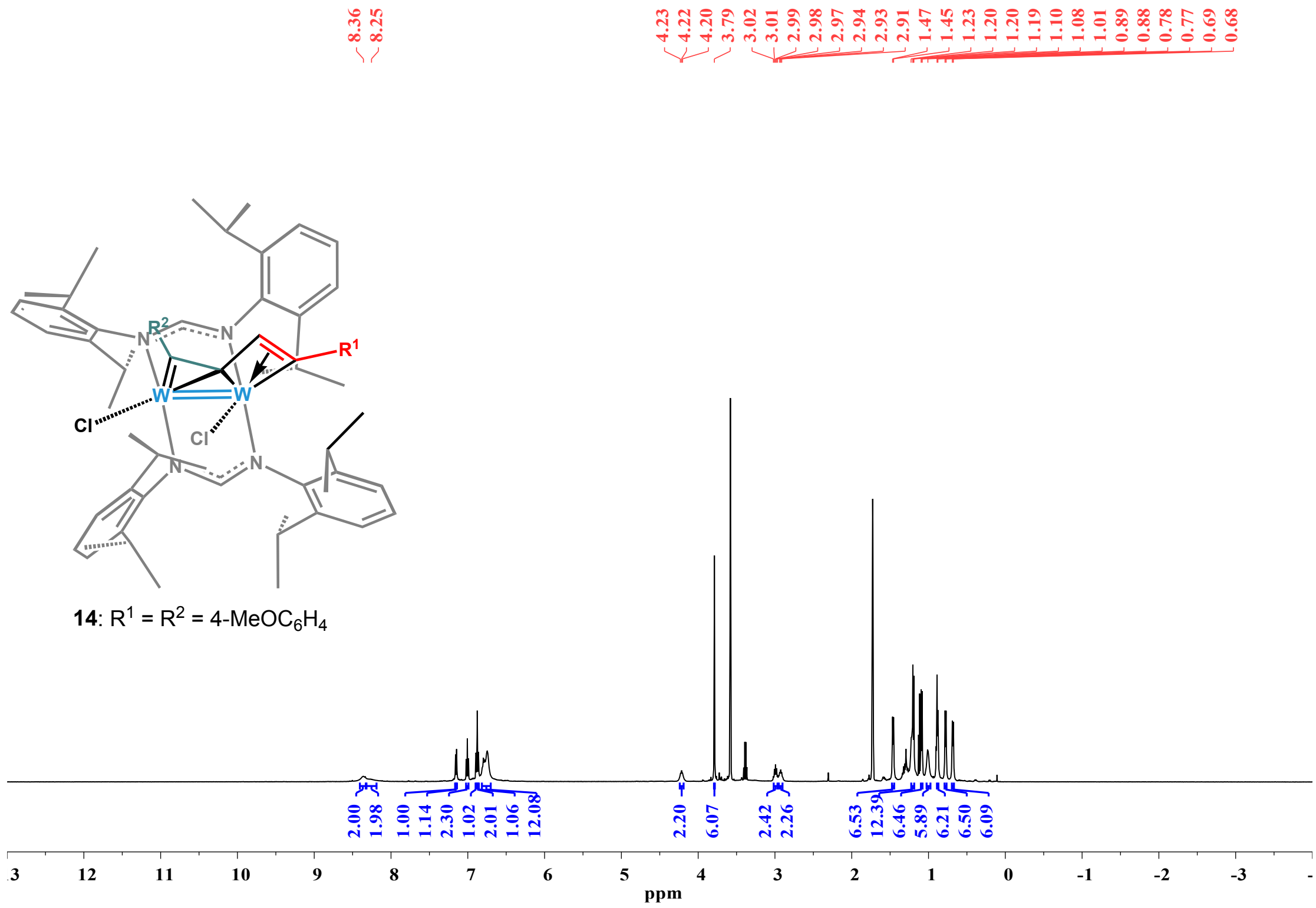

Figure. *S*62 ^1H NMR spectrum of **14** (500 MHz, d_8-THF, 298 K).

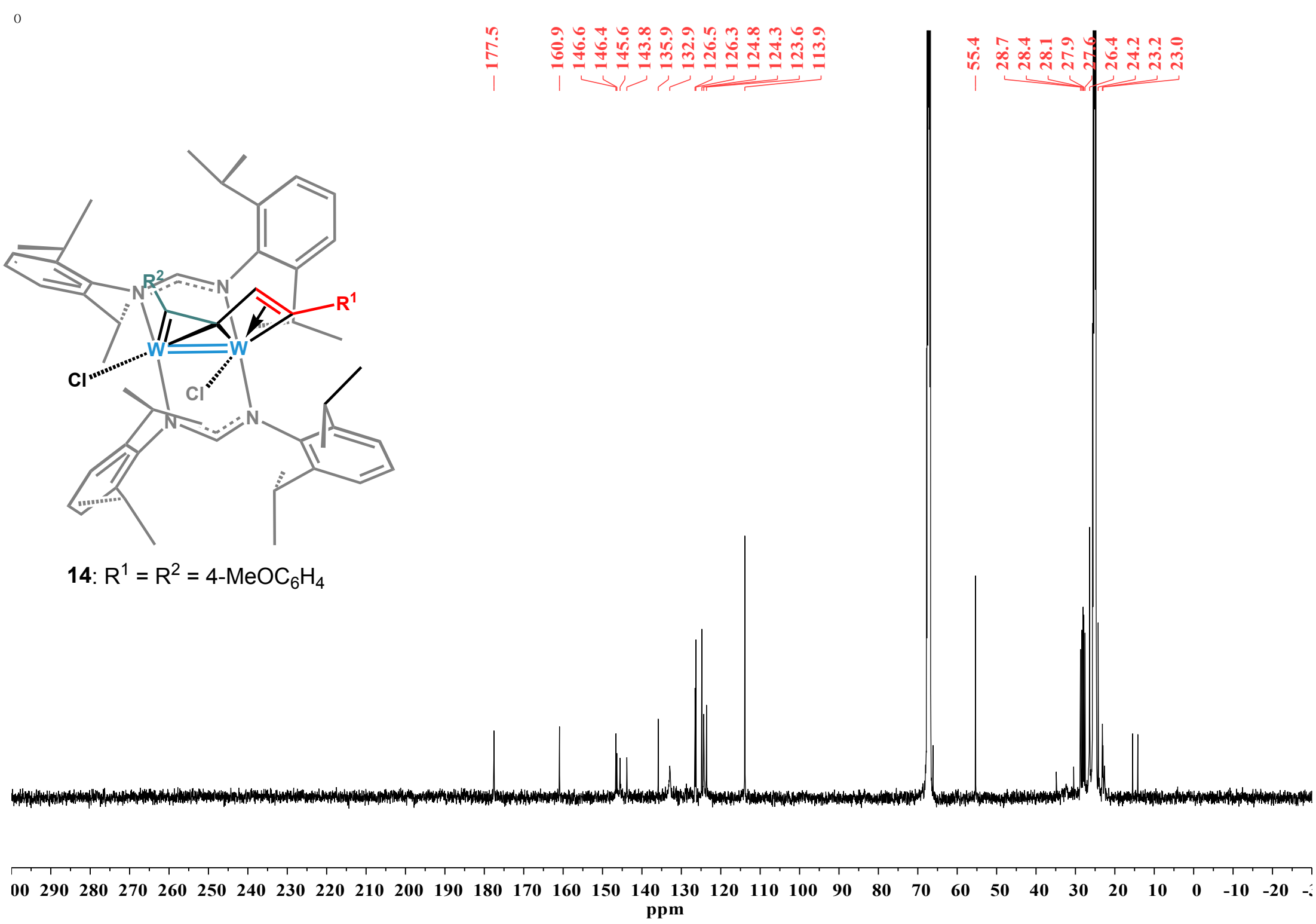

Figure. *S*63 $^{13}C\{^1H\}$ NMR spectrum of **14** (126 MHz, d_8-THF, 298 K).

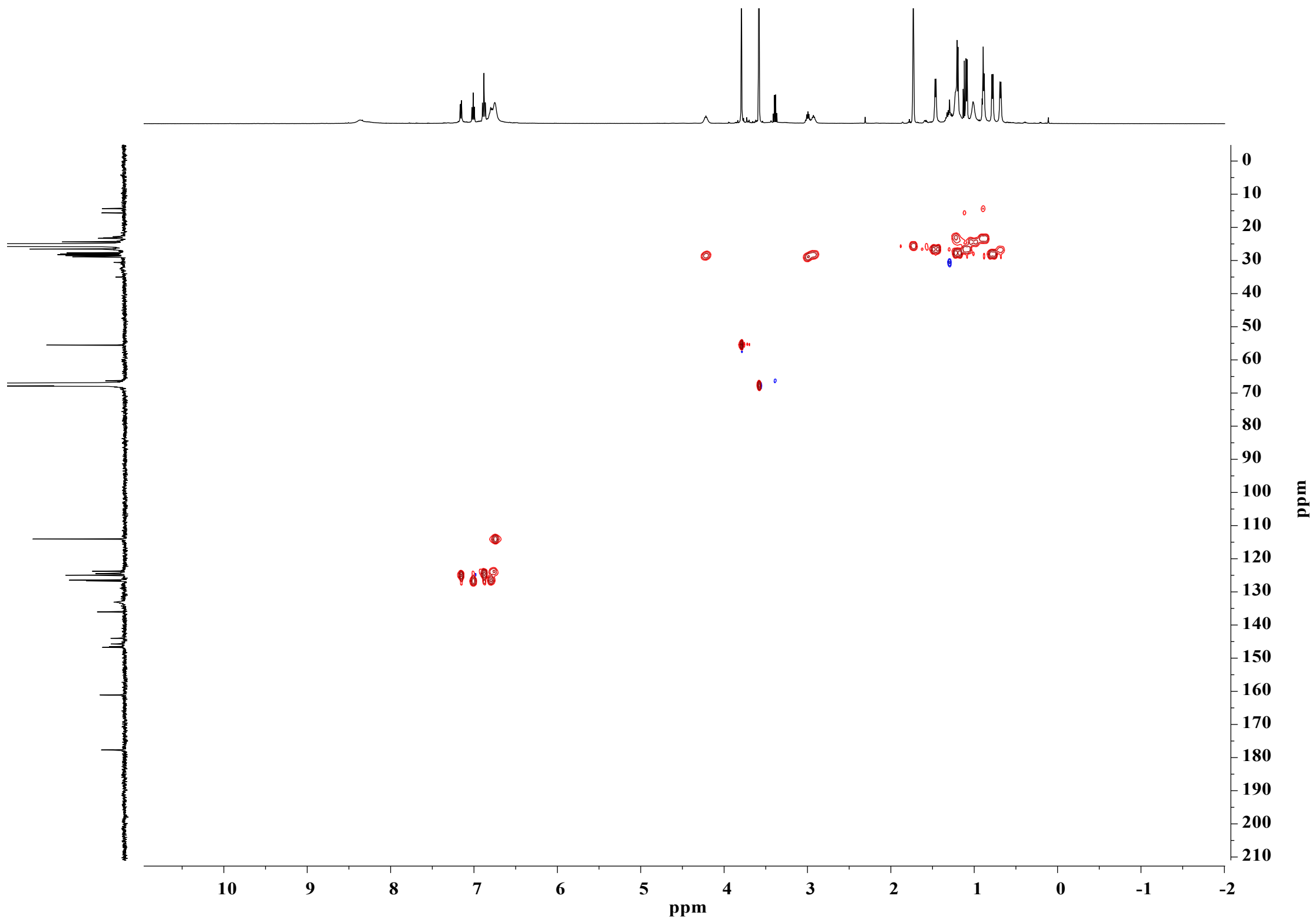

Figure. *S*64 HSQC (^{1}H-^{13}C) NMR spectrum of **14** (d_8-THF, 353 K).

4.15 $W_2Cl_2[\mu\text{-}\eta^2\text{:}\eta^2\text{-}1,4\text{-}(n\text{-}C_3H_7)_2C_4H_2][\mu\text{-}\kappa^2\text{-}HC(N\text{-}2,6\text{-}{}^iPr_2C_6H_3)_2]_2$ (15)

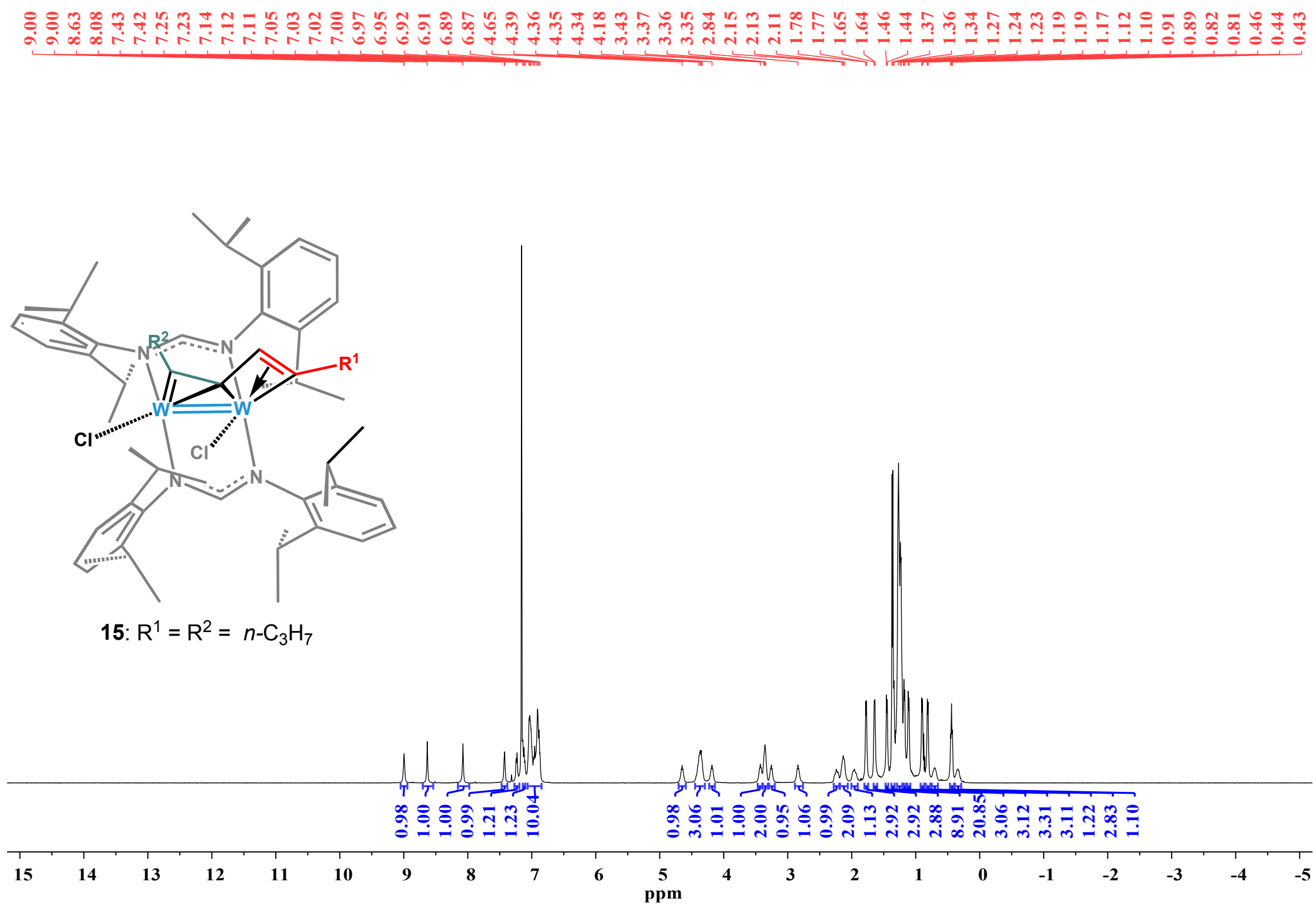

Figure. ***S*65** ^1H NMR spectrum of **15** (500 MHz, C_6D_6, 298 K).

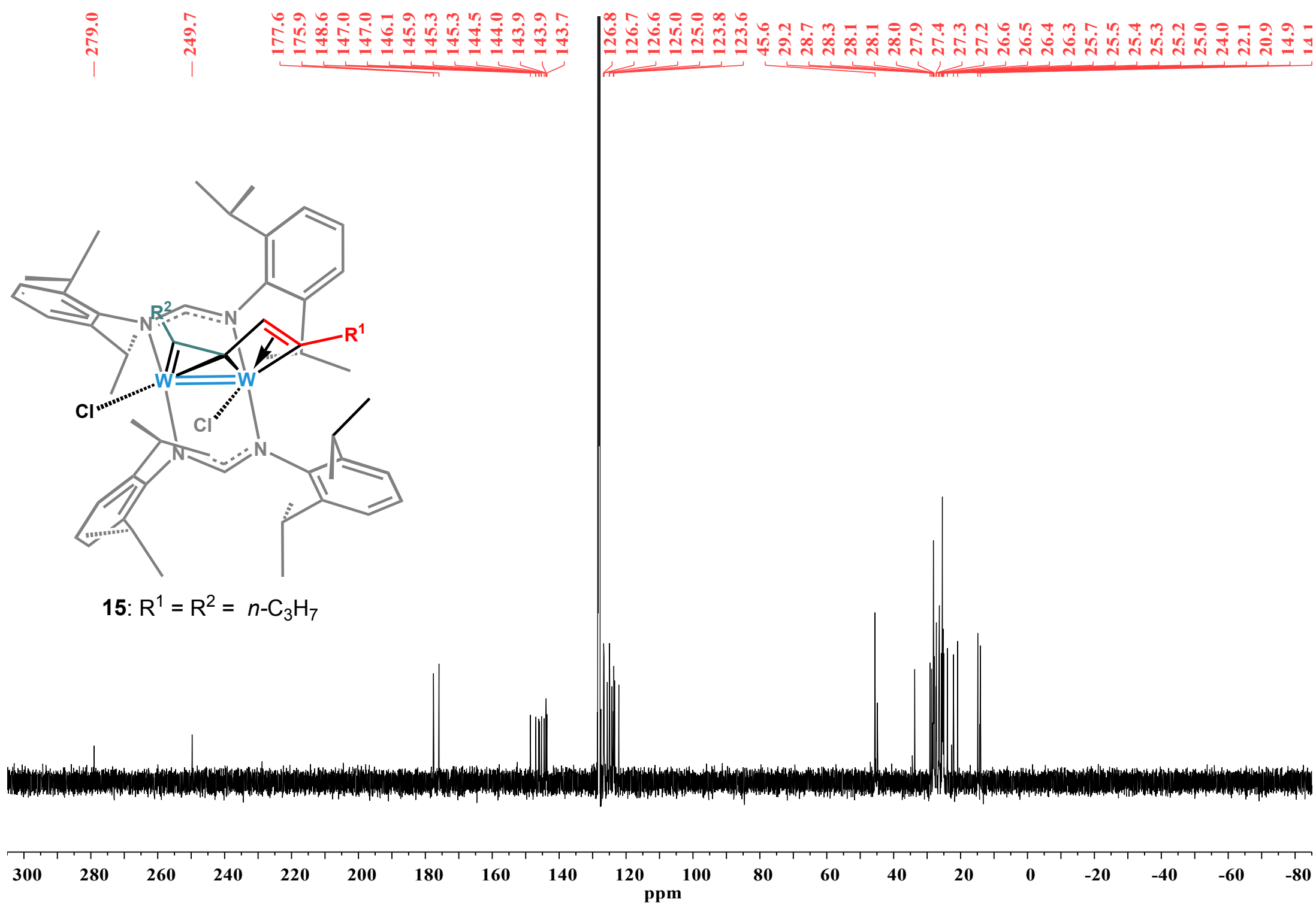

Figure. ***S*66** $^{13}C\{^1H\}$ NMR spectrum of **15** (126 MHz, C_6D_6, 298 K).

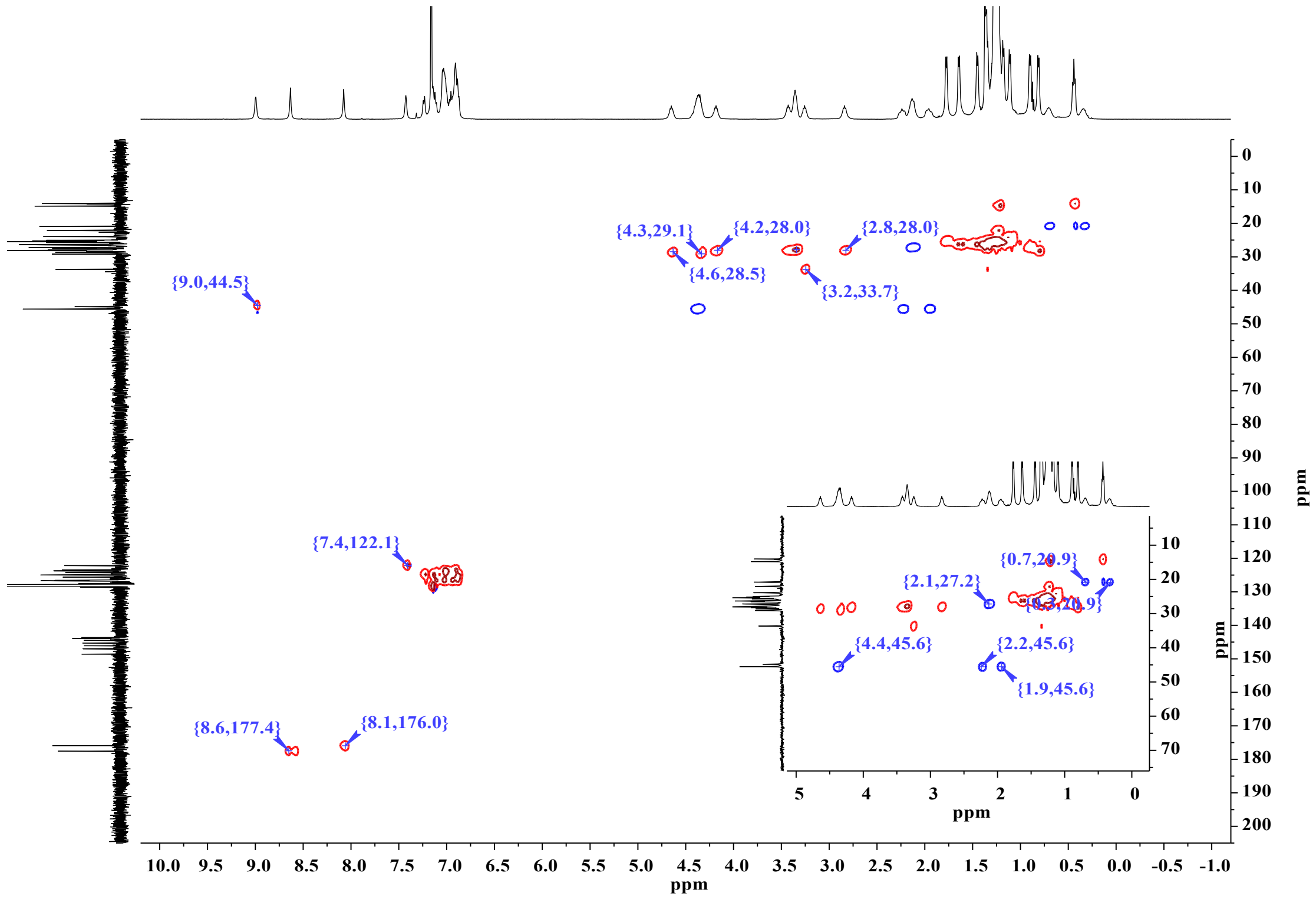

Figure. *S*67 HSQC (^1H-^{13}C) NMR spectrum of **15** (C_6D_6, 353 K).

4.16 $W_2Cl_2[\mu\text{-}\eta^2{:}\eta^2\text{-}1\text{-}2\text{-}MeC_6H_4\text{-}4\text{-}n\text{-}C_3H_7C_4H_2][\mu\text{-}\kappa^2\text{-}HC(N\text{-}2,6\text{-}{}^iPr_2C_6H_3)_2]_2$ (16)

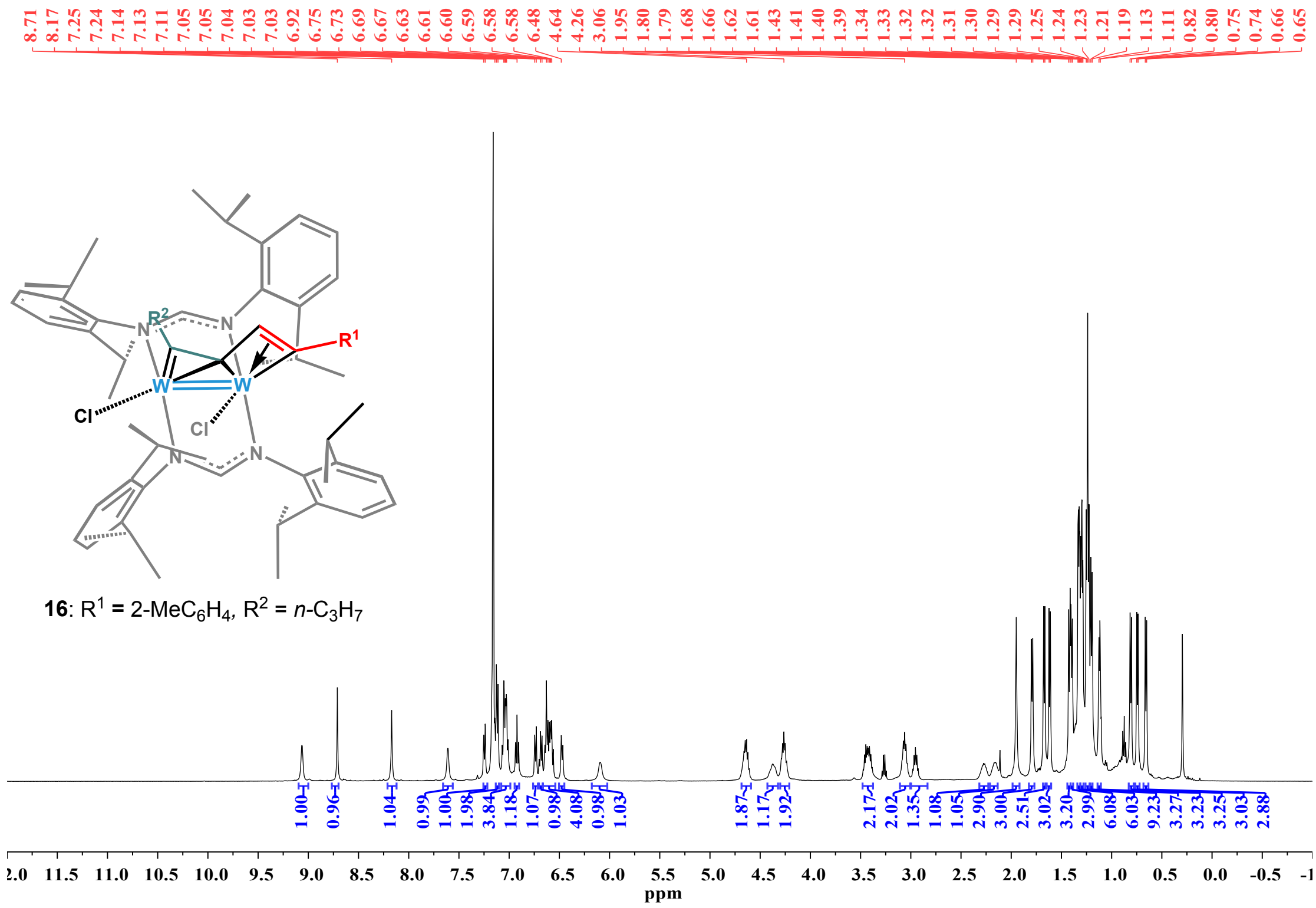

Figure. *S*68 ^{1}H NMR spectrum of **16** (500 MHz, C_6D_6, 298 K).

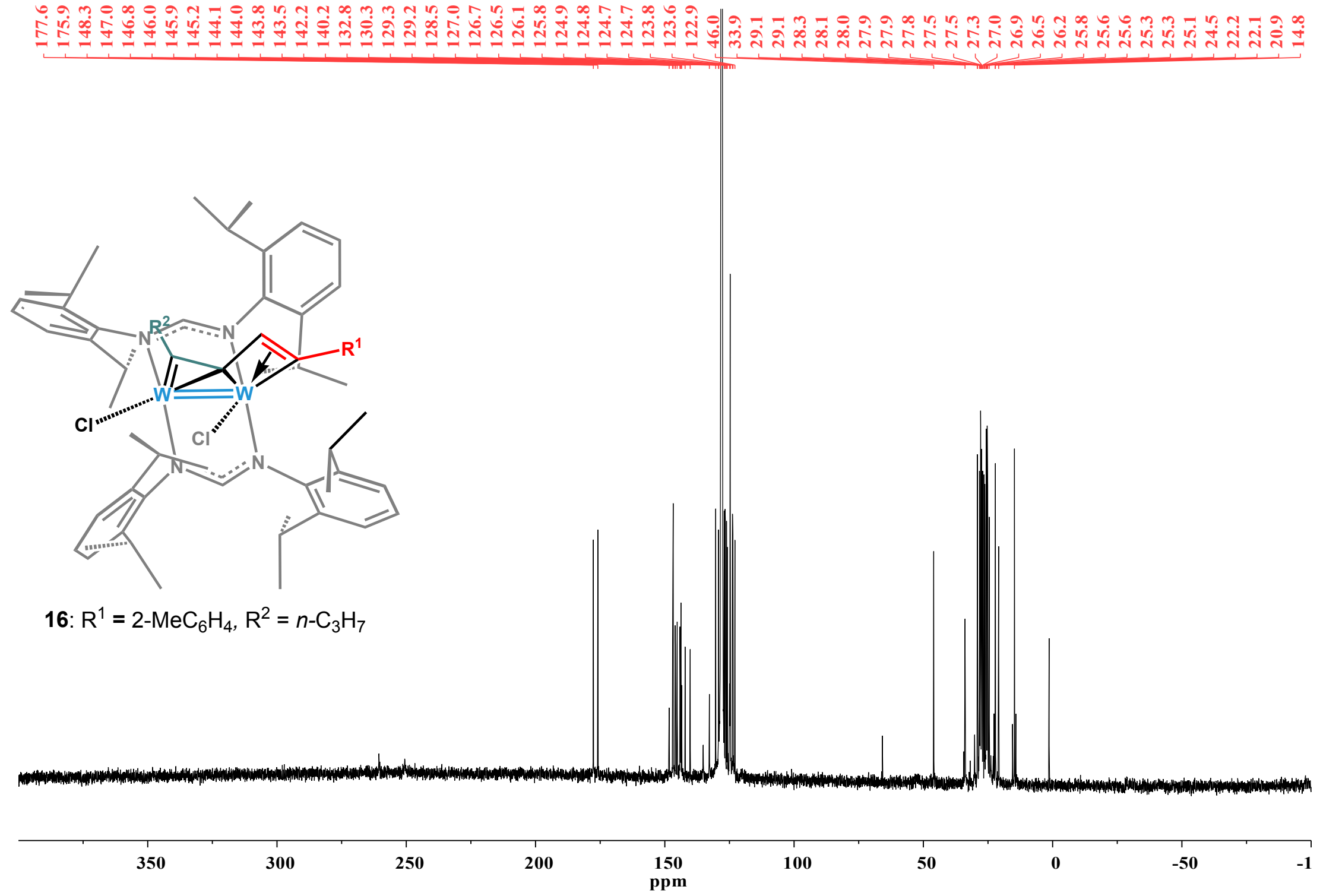

Figure. *S*69 $^{13}C\{^1H\}$ NMR spectrum of **16** (126 MHz, C_6D_6, 298 K).

4.17 W_2Cl_2[μ-η^2-2-MeC_6H_4CCH][μ-κ^2-HC(N-2,6-$^i Pr_2C_6H_3$)$_2$]$_2$ (17)

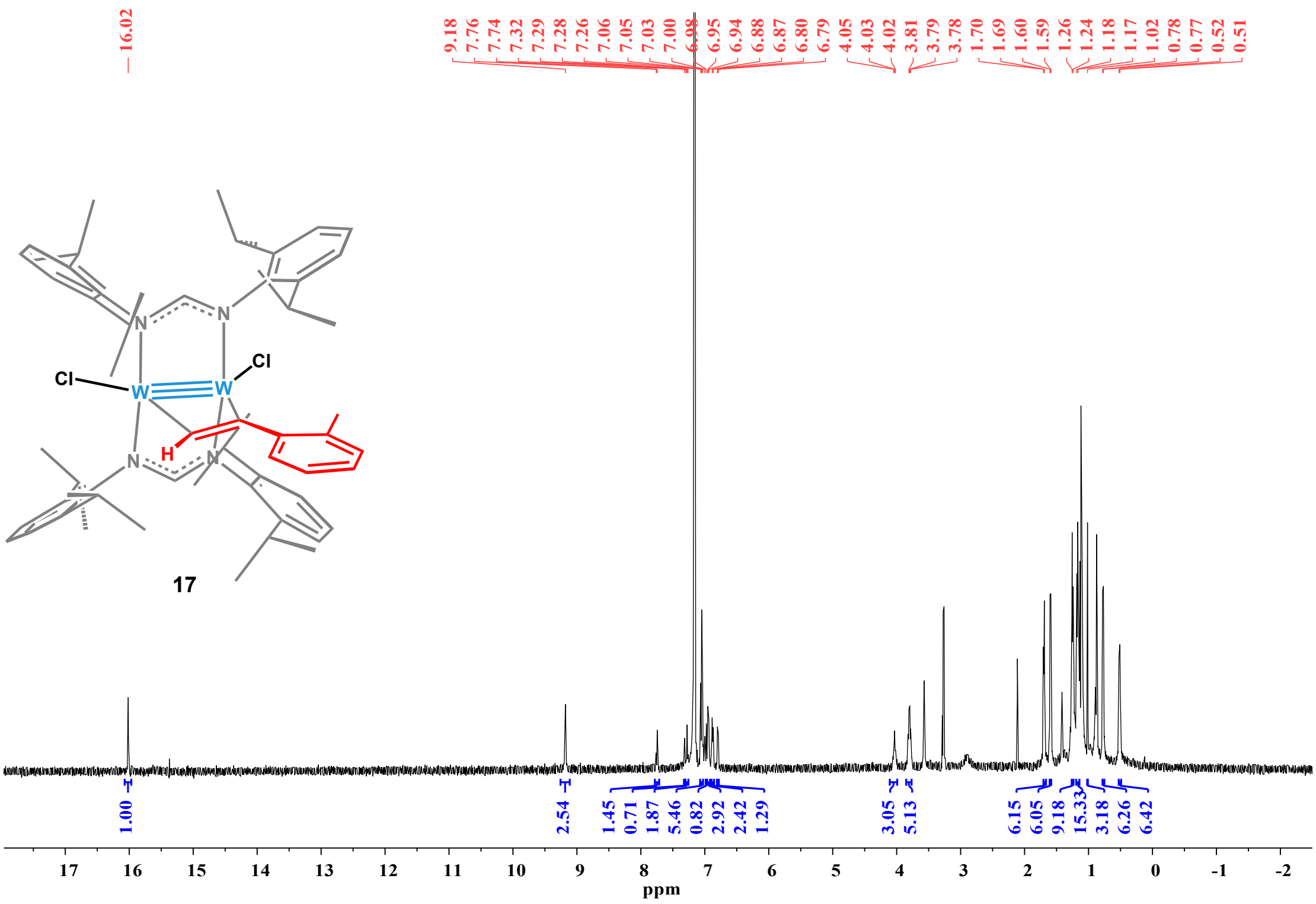

Figure. *S*70 ^{1}H NMR spectrum of **17** (500 MHz, C_6D_6, 298 K).

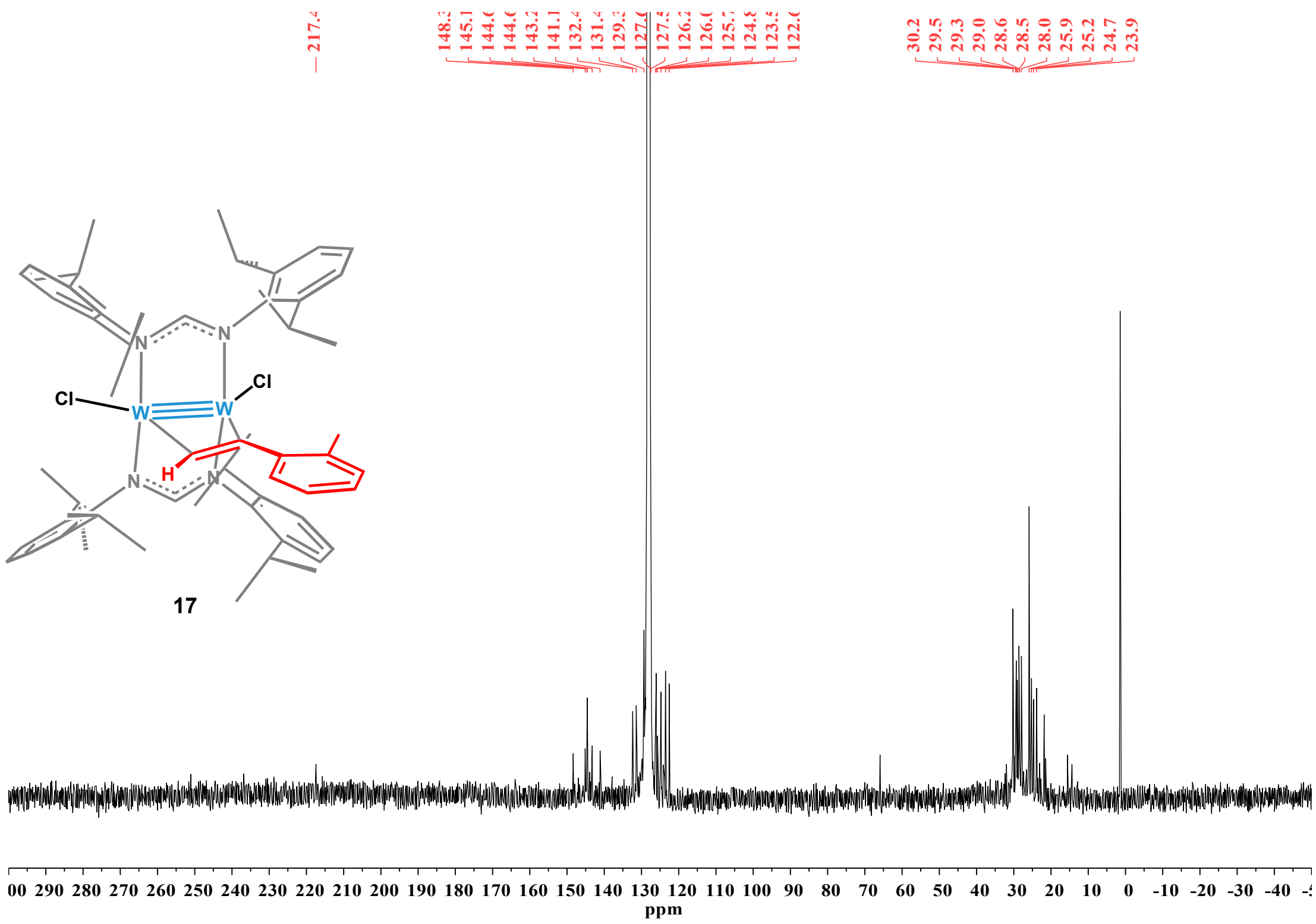

Figure. *S*71 $^{13}C\{^1H\}$ NMR spectrum of **17** (126 MHz, C_6D_6, 298 K).

4.18 $W_2[\mu\text{-}\kappa^2\text{-}1\text{-}2\text{-}MeC_6H_4\text{-}4\text{-}n\text{-}C_3H_7C_4H_2][\mu\text{-}\kappa^2\text{-}HC(N\text{-}2,6\text{-}{}^iPr_2C_6H_3)_2]_2$ (18)

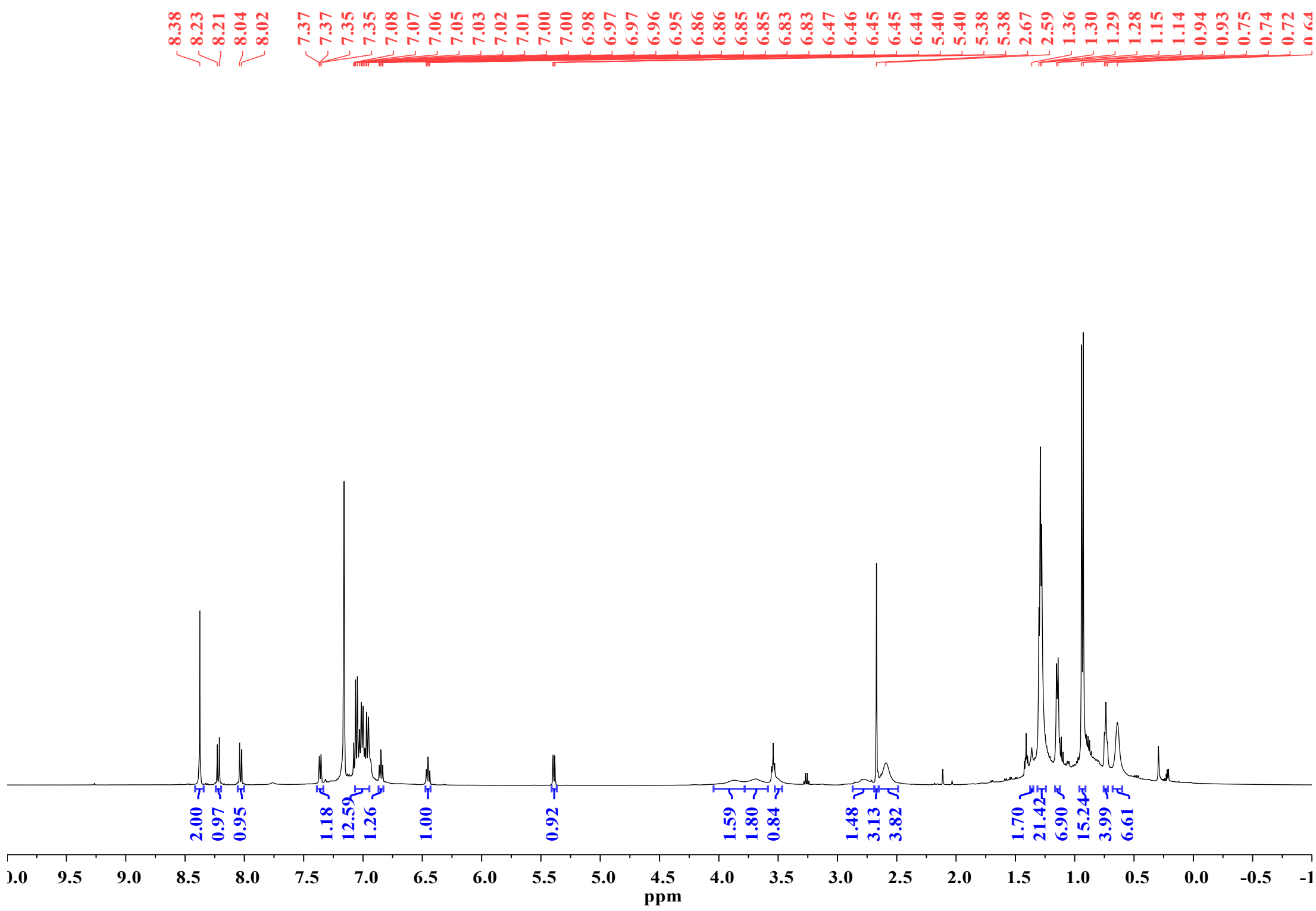

Figure. *S*72 ^1H NMR spectrum of **18** (500 MHz, C_6D_6, 298 K).

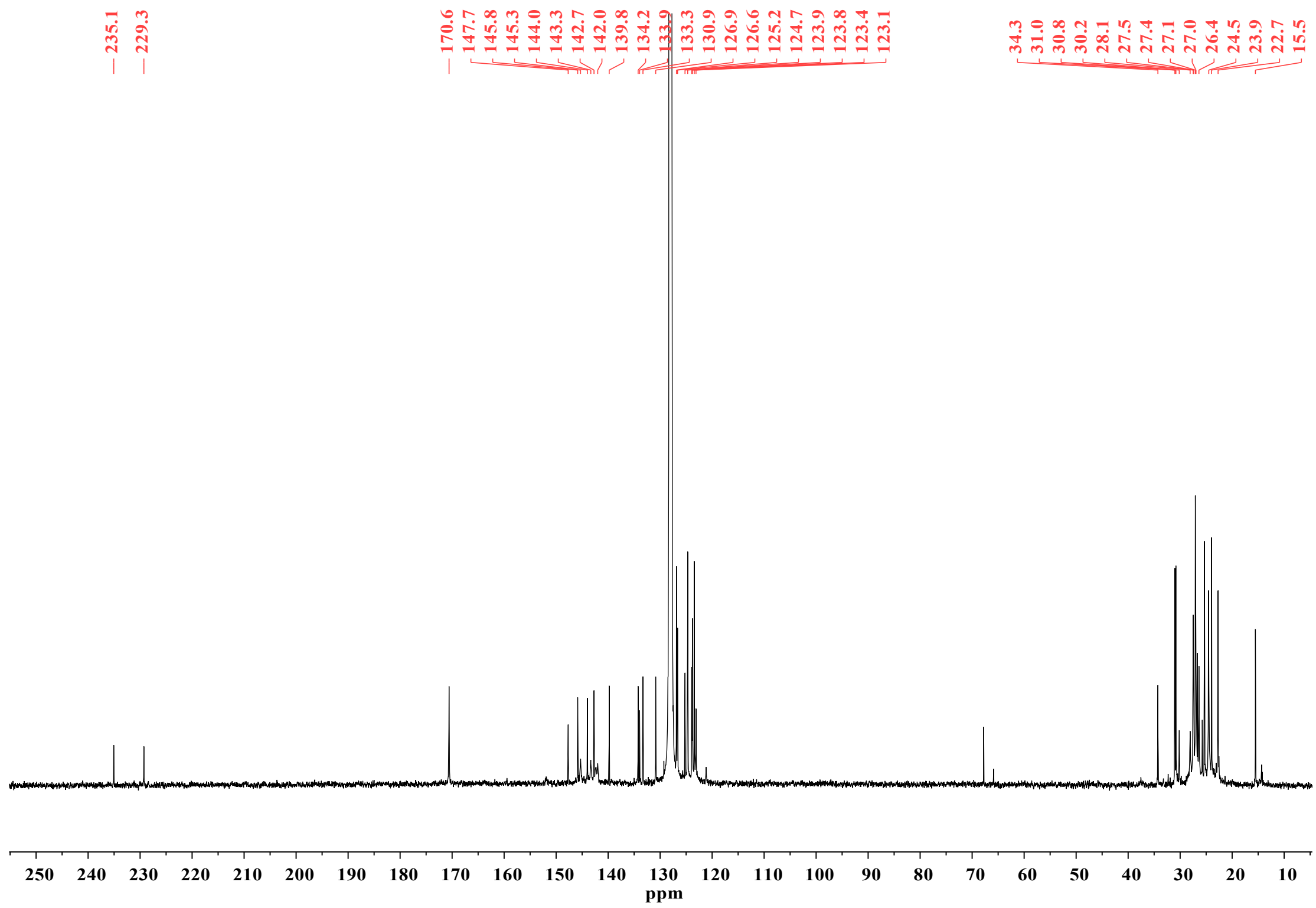

Figure. *S*73 $^{13}C\{^1H\}$ NMR spectrum of **18** (126 MHz, C_6D_6, 298 K).

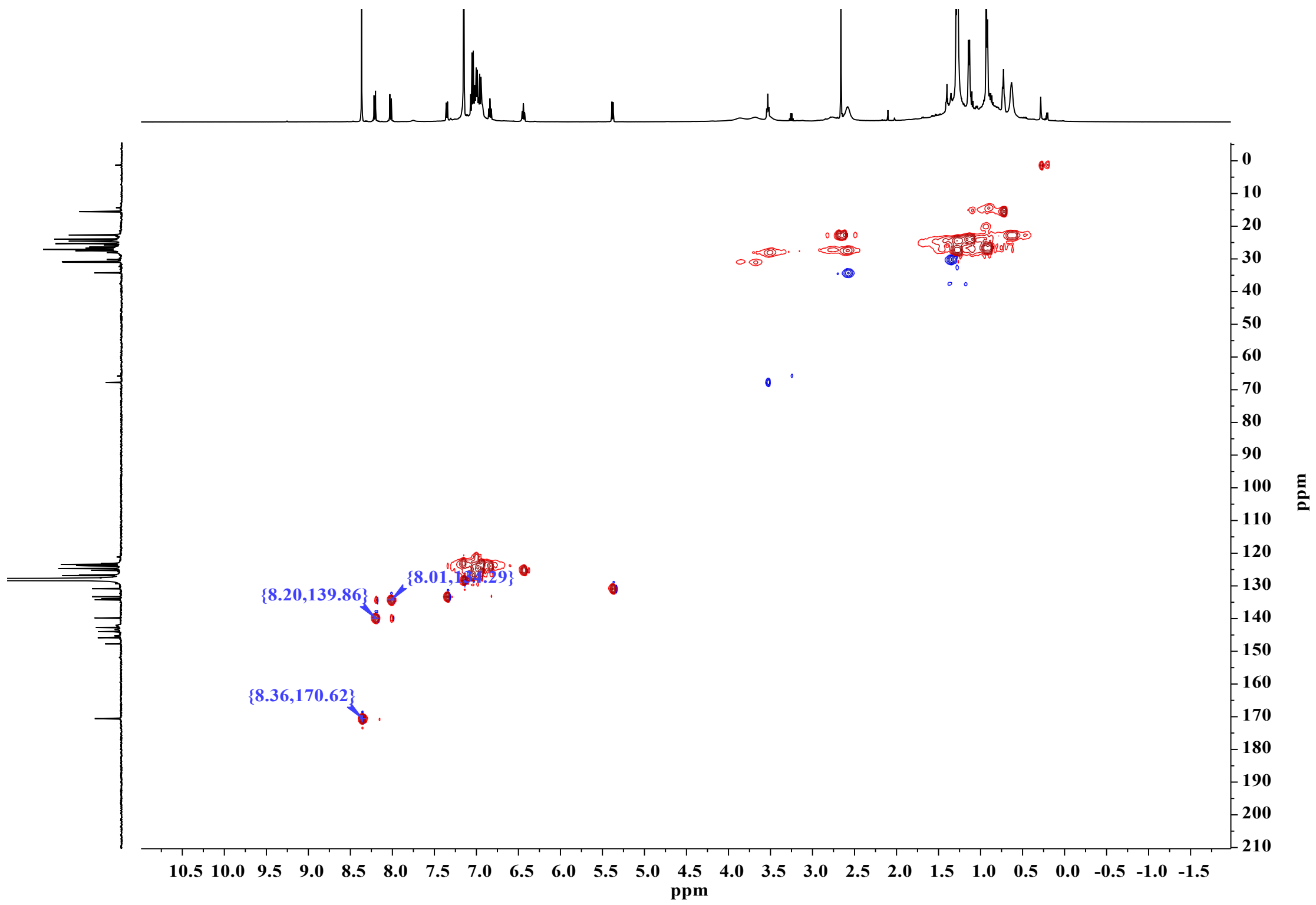

Figure. *S*74 HSQC (^1H-^{13}C) NMR spectrum of **18** (C_6D_6, 298 K).

4.19 W_2Cl_2[μ-η^2:η^2-1-*n*-C_3H_7-4-2-MeC_6H_4(H)-C_4H][μ-κ^2-HC(N-2,6-^{i}$Pr_2C_6H_3$)$_2$]$_2$ (19)

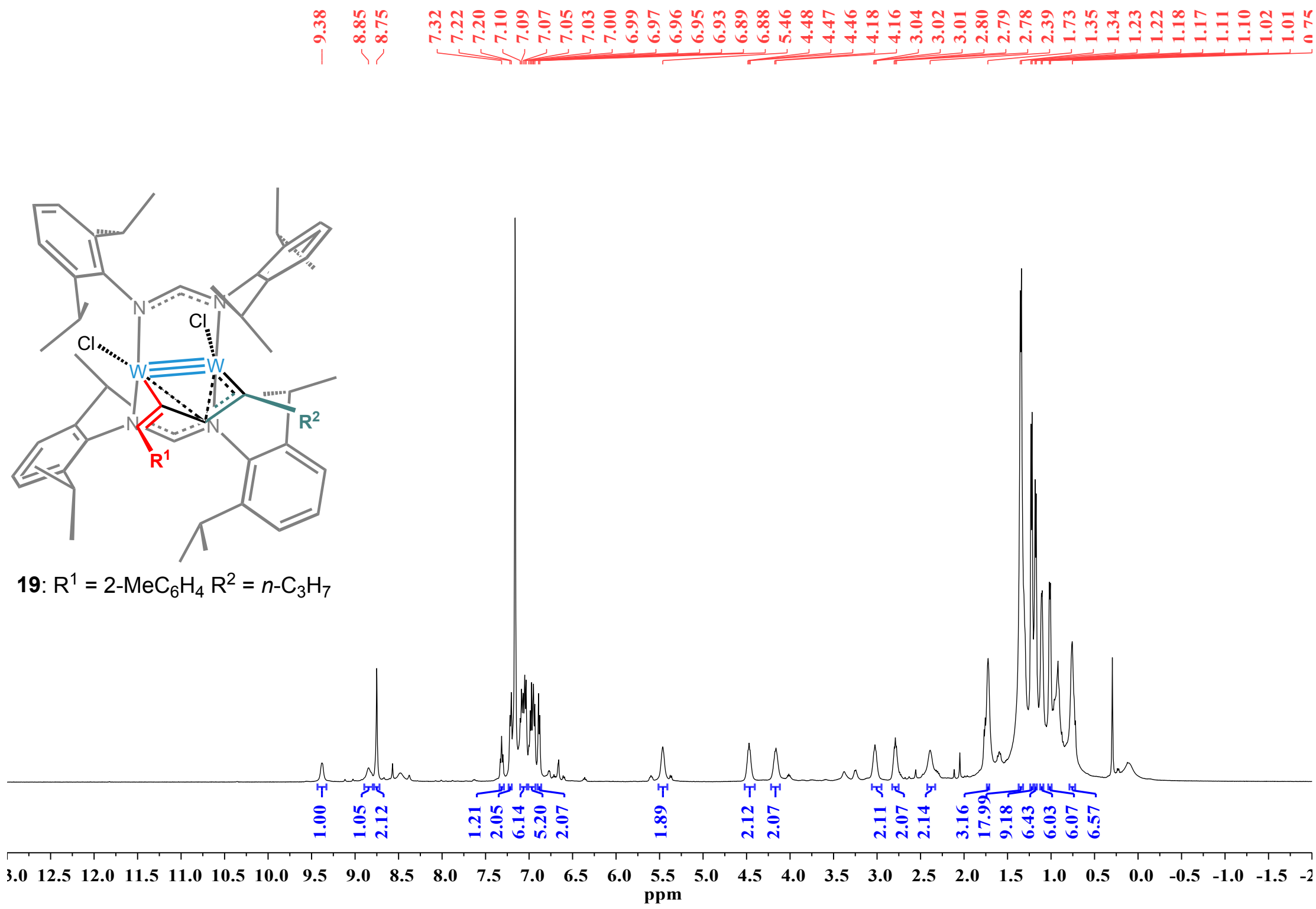

Figure. *S*75 ^{1}H NMR spectrum of **19** (500 MHz, C_6D_6, 298 K).

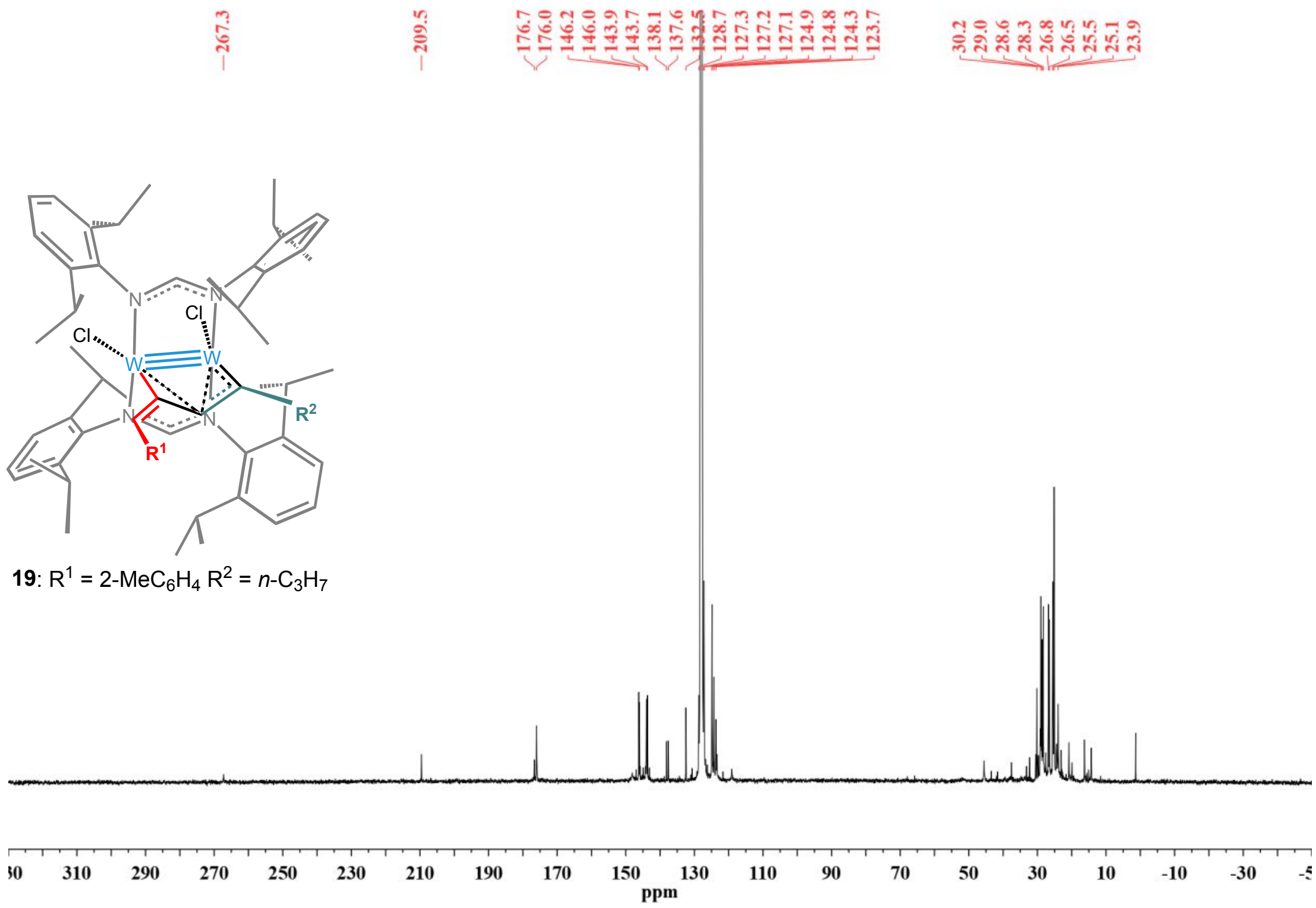

Figure. *S*76 $^{13}C\{^1H\}$ NMR spectrum of **19** (126 MHz, C_6D_6, 298 K).

4.20 $W_2Cl_2(THF)[\mu-\eta^1:\eta^2-CC(H)-2-MeC_6H_4][\mu-\kappa^2-HC(N-2,6-{}^iPr_2C_6H_3)_2]_2$ (20)

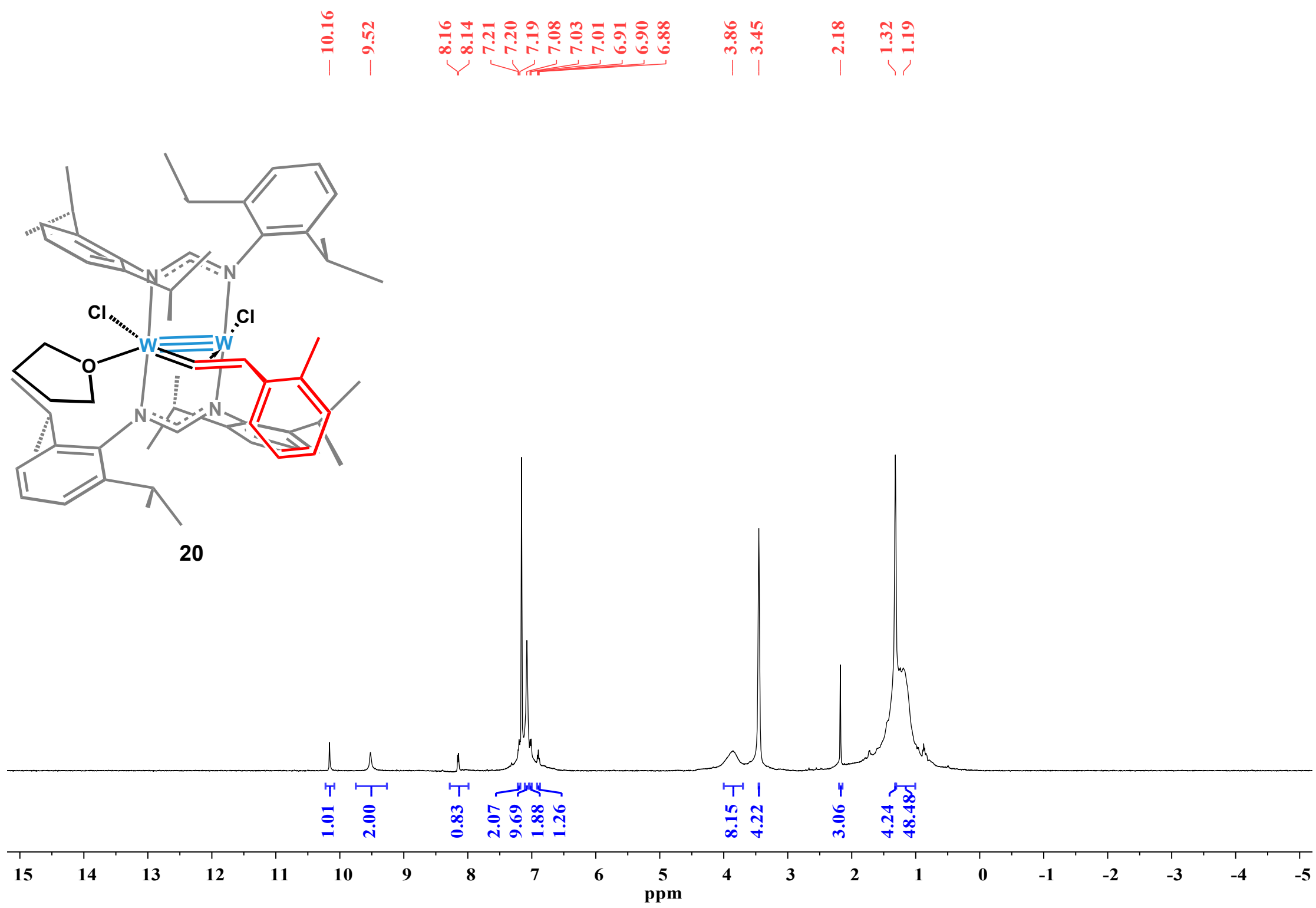

Figure. *S*77 ^{1}H NMR spectrum of **20** (500 MHz, C_6D_6, 298 K).

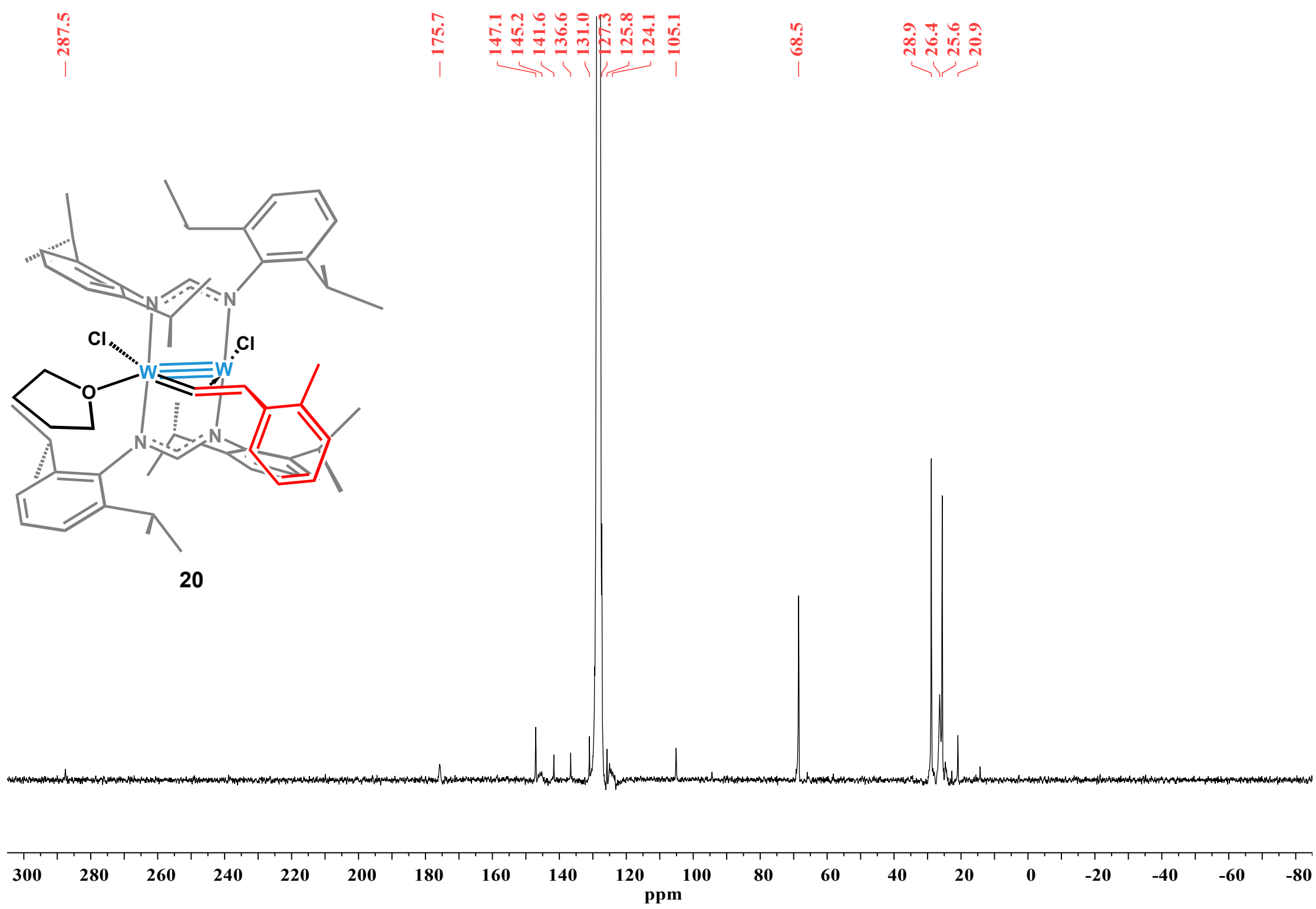

Figure. *S*78 $^{13}C\{^1H\}$ NMR spectrum of **20** (126 MHz, C_6D_6, 298 K).

4.21 $W_2Cl_2(C_5H_5N)[\mu\text{-}\eta^1\text{:}\eta^2\text{-}CC(H)\text{-}2\text{-}MeC_6H_4][\mu\text{-}\kappa^2\text{-}HC(N\text{-}2,6\text{-}{}^iPr_2C_6H_3)_2]_2$ (21)

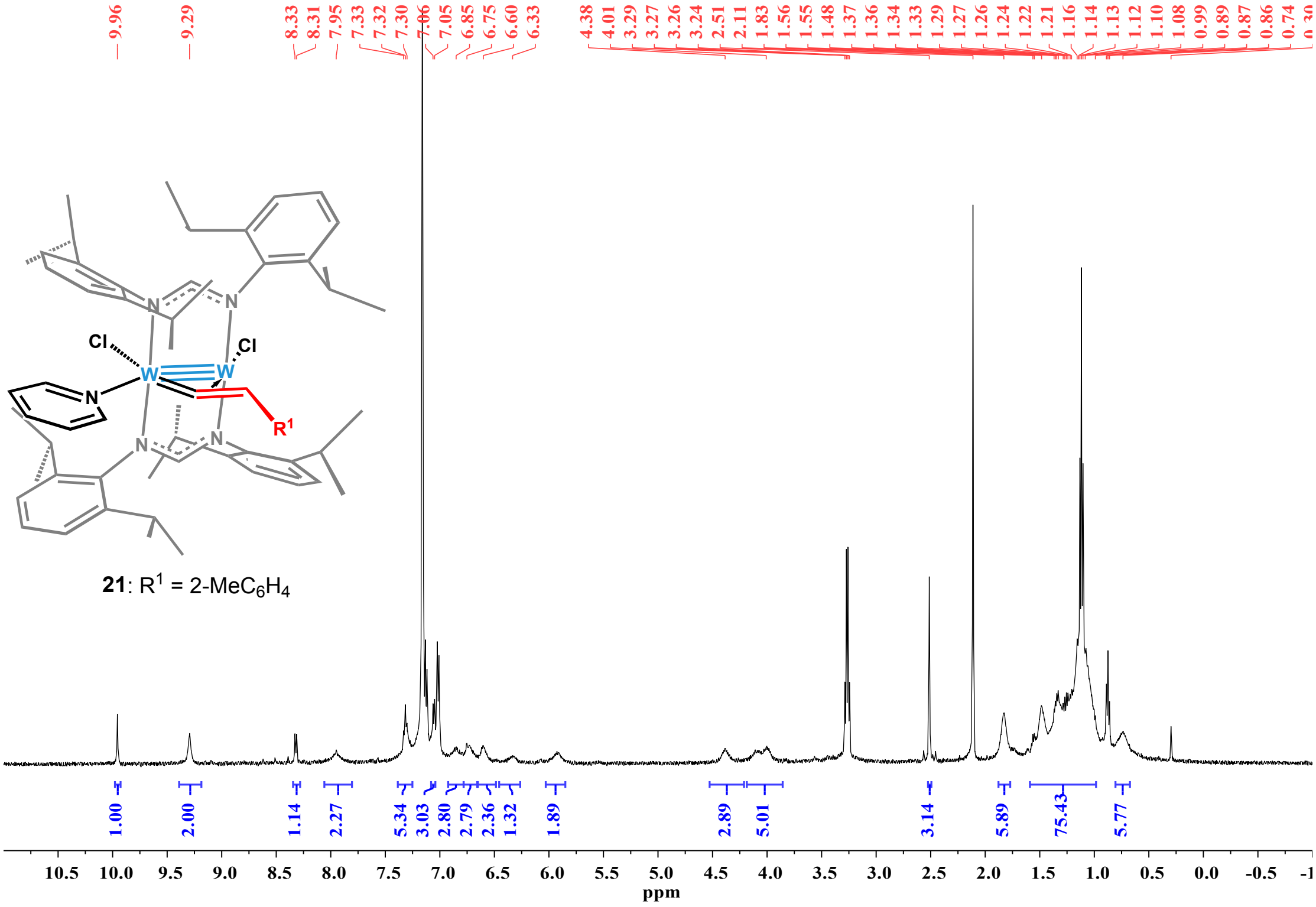

Figure. *S*79 ^{1}H NMR spectrum of **21** (500 MHz, C_6D_6, 298 K).

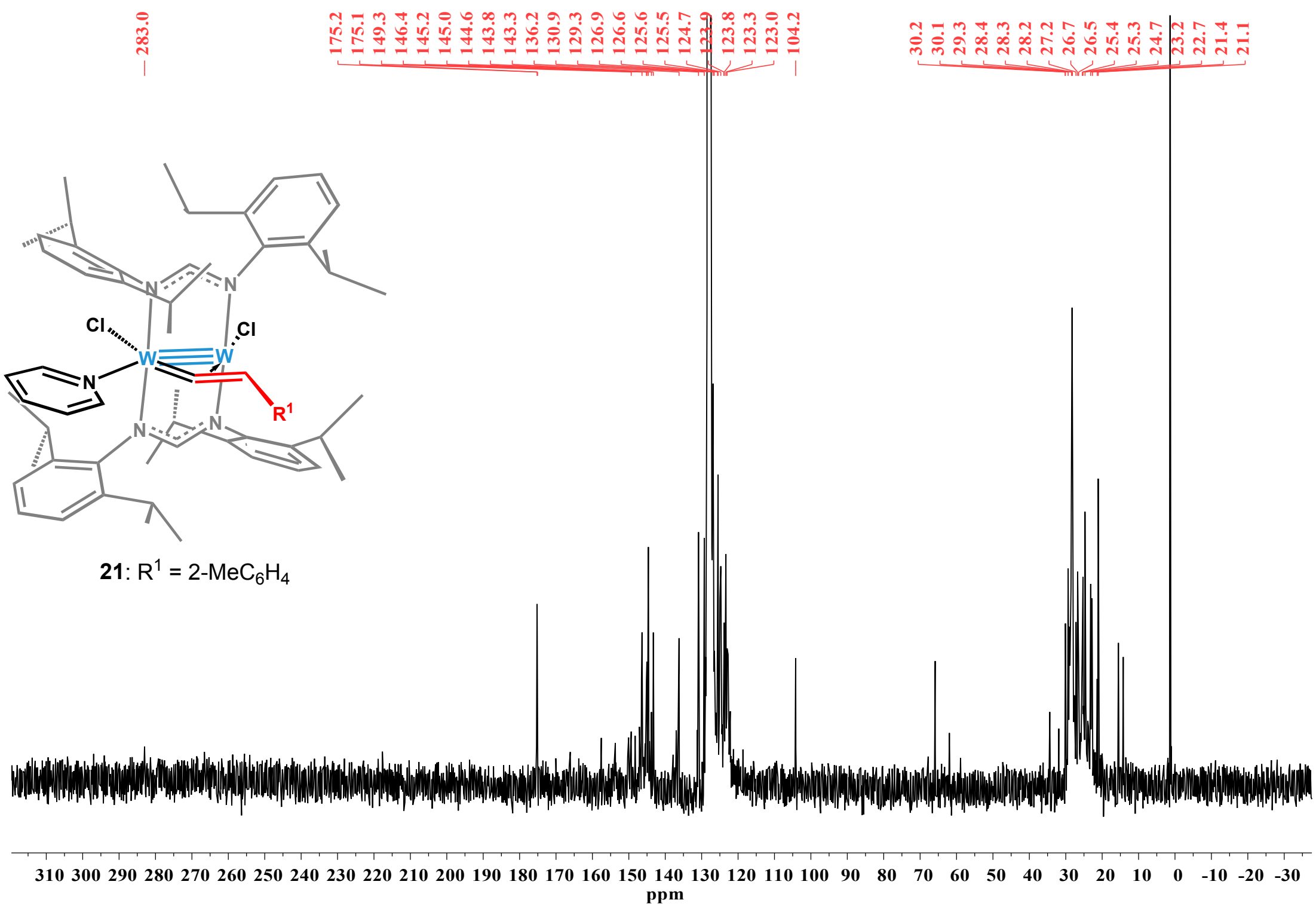

Figure. *S*80 $^{13}C\{^1H\}$ NMR spectrum of **21** (126 MHz, C_6D_6, 298 K).

4.22 $W_2Cl_2(C_5H_5N)[\mu\text{-}\eta^1\text{:}\eta^2\text{-}CC(H)\text{-}t\text{-}Bu][\mu\text{-}\kappa^2\text{-}HC(N\text{-}2,6\text{-}{}^{i}Pr_2C_6H_3)_2]_2$ (22)

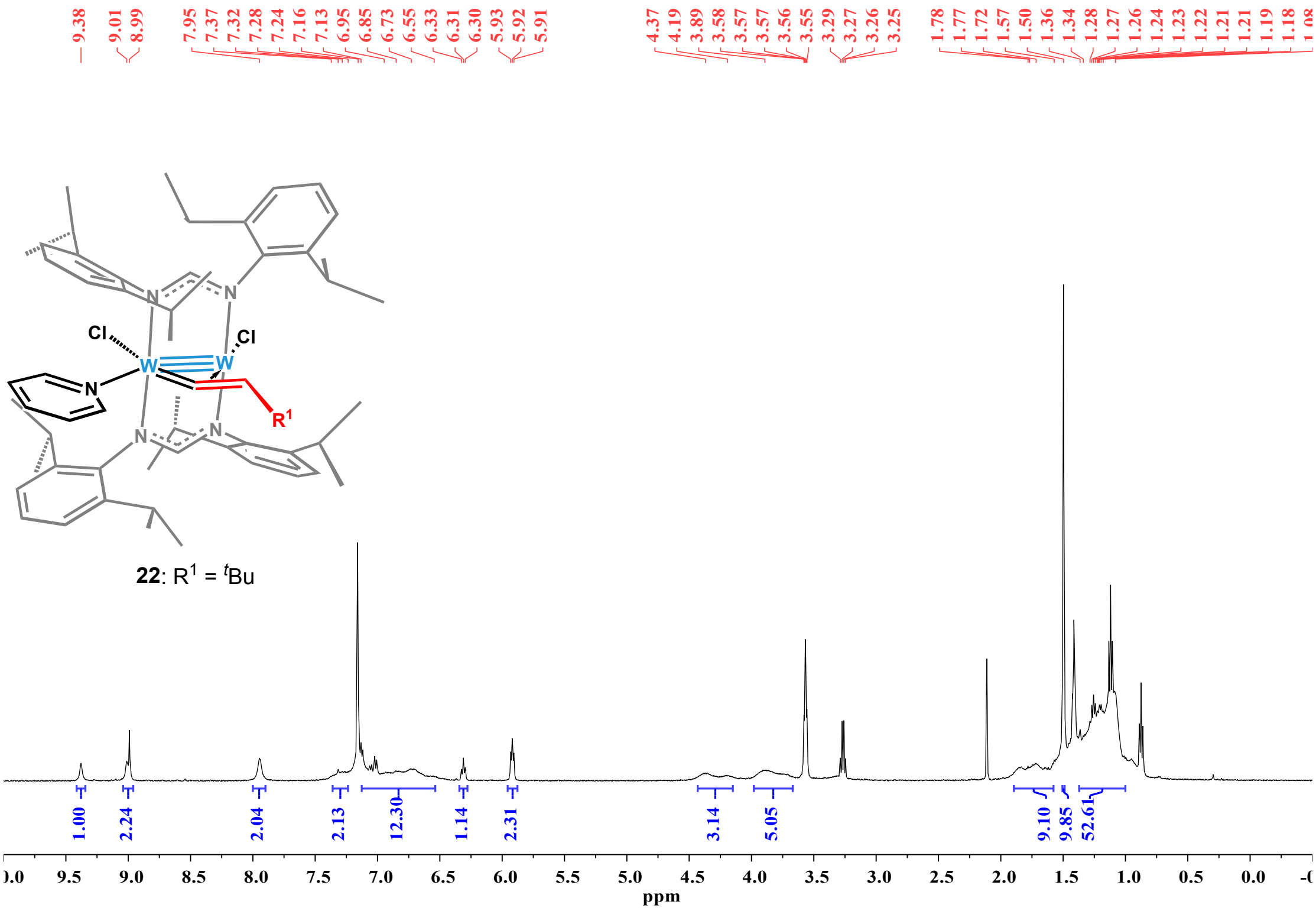

Figure. *S*81 ^{1}H NMR spectrum of **22** (500 MHz, C_6D_6, 298 K).

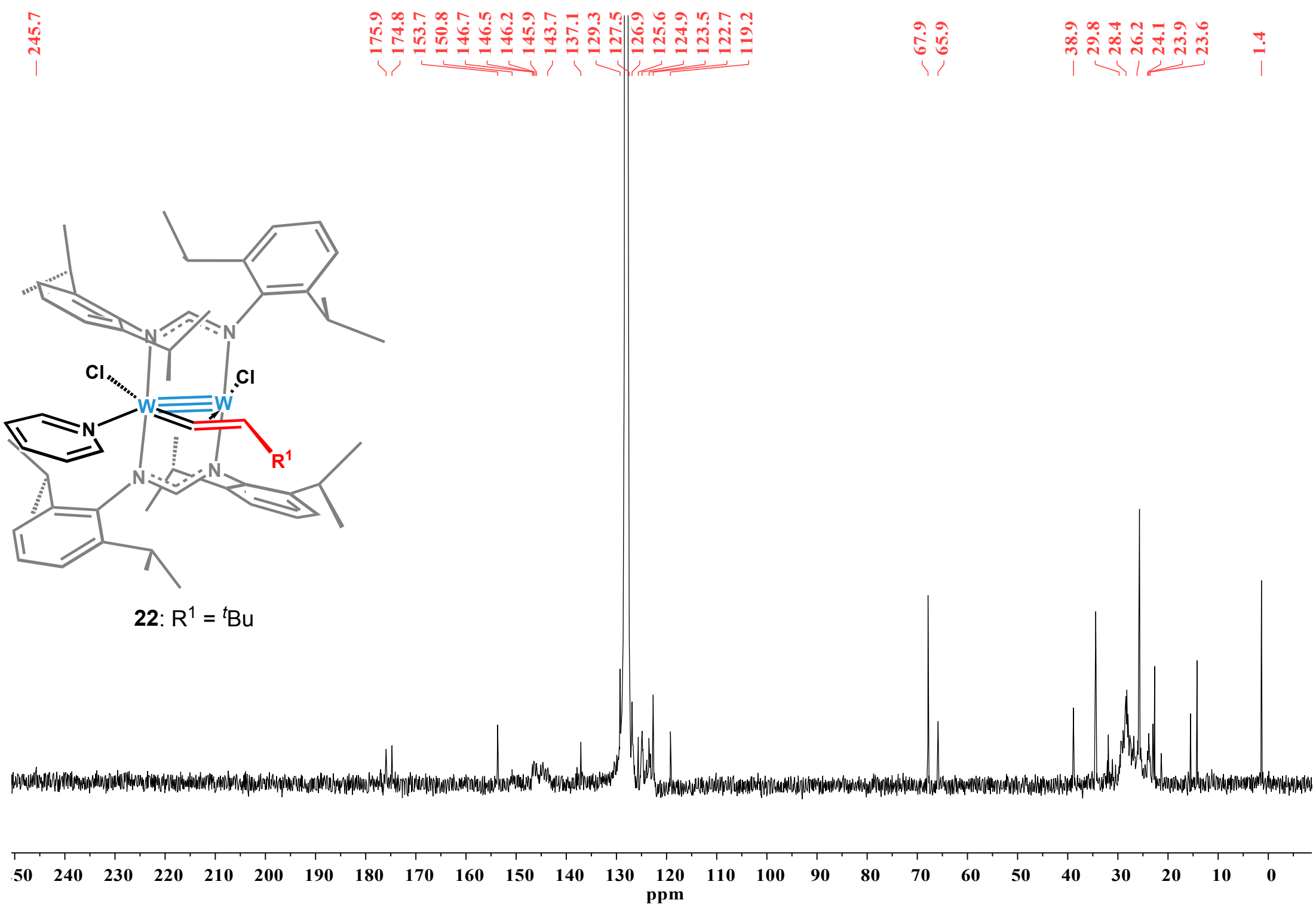

Figure. *S*82 $^{13}C\{^1H\}$ NMR spectrum of **22** (126 MHz, C_6D_6, 298 K).

4.23 $W_2Cl_2(C_5H_5N)[\mu\text{-}\eta^1:\eta^2\text{-}CC(H)\text{-}SiMe_3][\mu\text{-}\kappa^2\text{-}HC(N\text{-}2,6\text{-}^iPr_2C_6H_3)_2]_2$ (23)

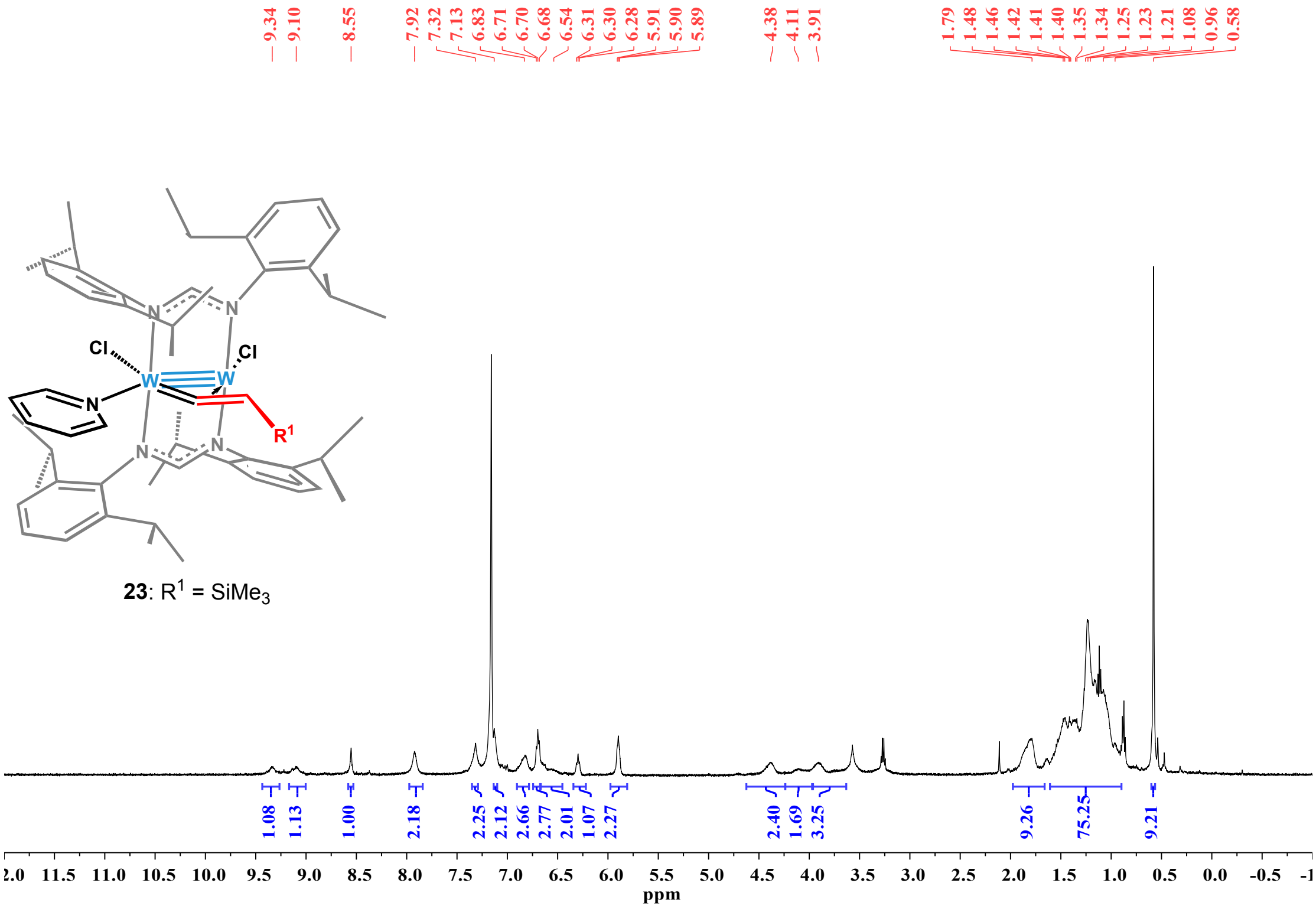

Figure. *S*83 ^1H NMR spectrum of **23** (500 MHz, C_6D_6, 298 K).

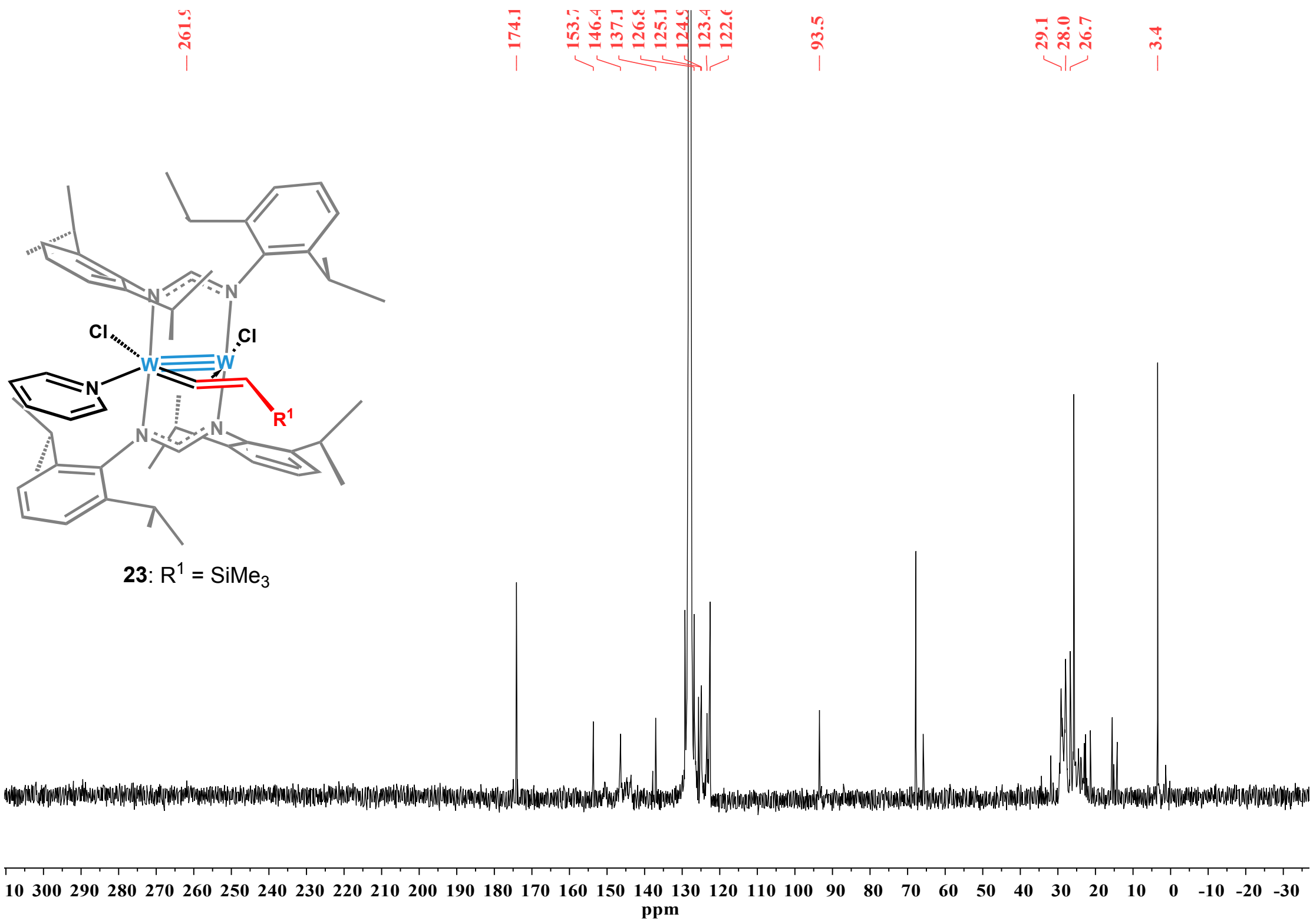

Figure. *S*84 $^{13}C\{^1H\}$ NMR spectrum of **23** (126 MHz, C_6D_6, 298 K).

4.24 W_2Cl_2[μ-η²:η²-1-C_6H_5-4-^{t}BuH-C_4H][μ-κ²-HC(N-2,6-^{i}Pr$_2C_6H_3$)$_2$]$_2$ (24)

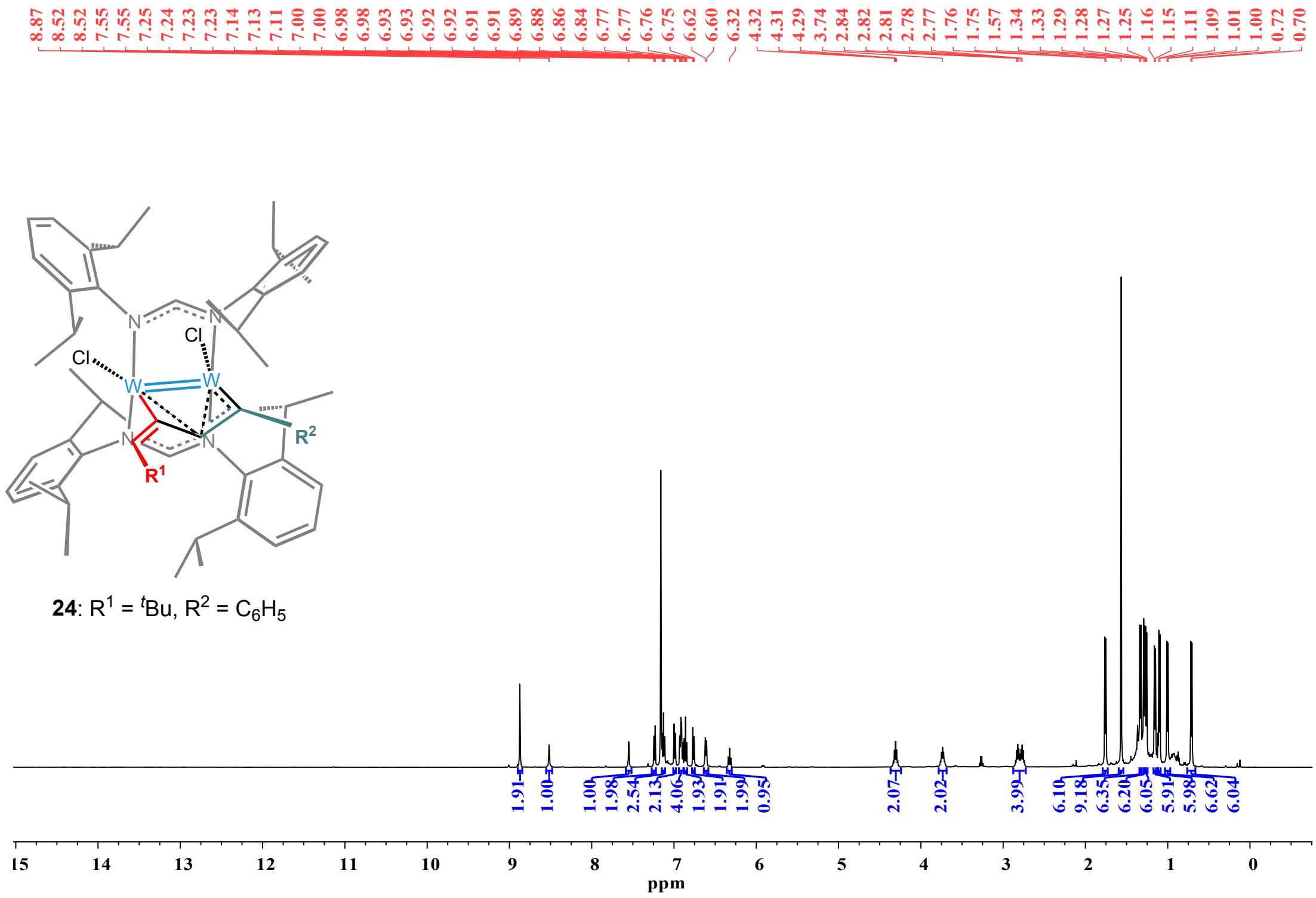

Figure. *S*85 ¹H NMR spectrum of **24** (500 MHz, C_6D_6, 298 K).

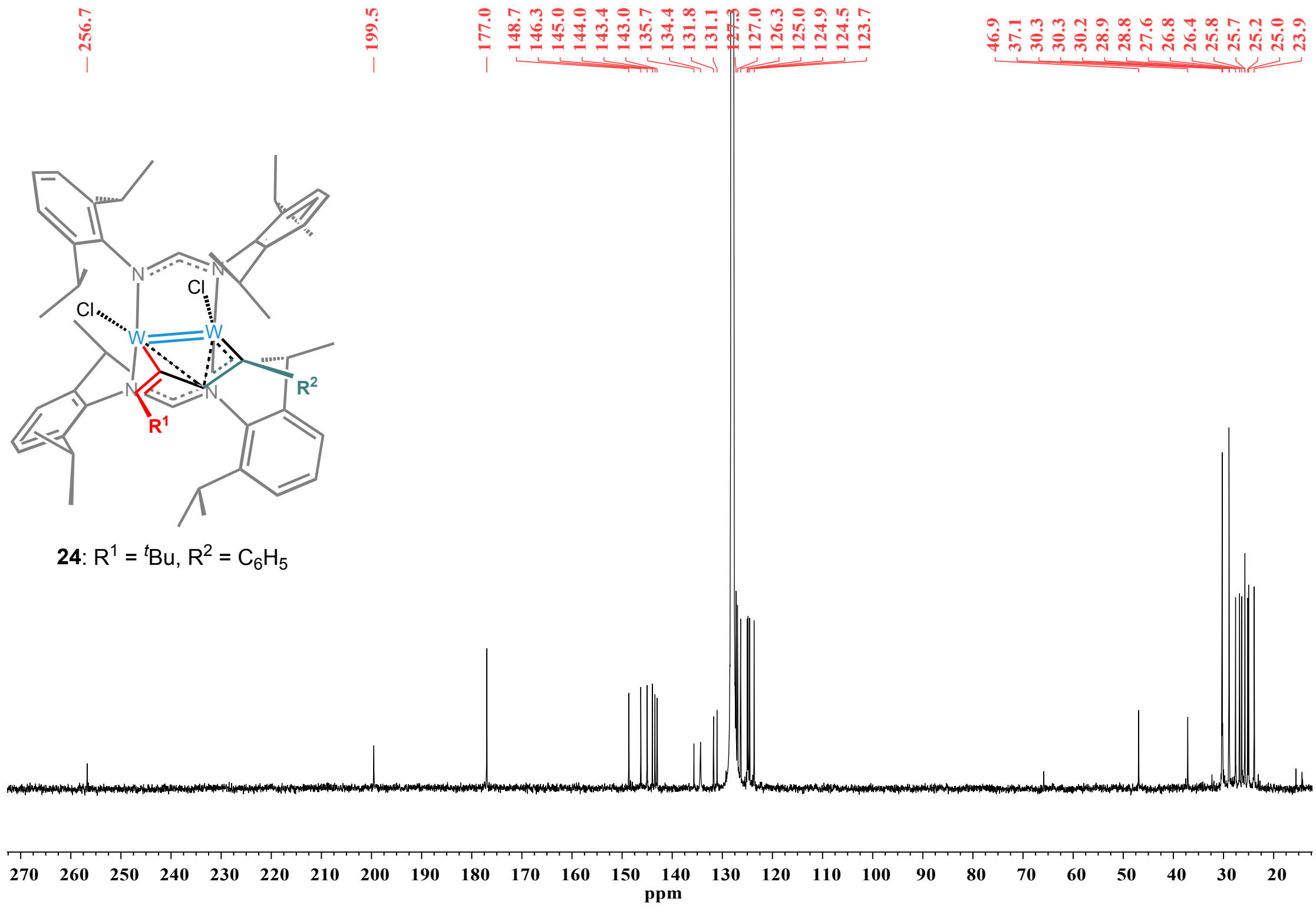

Figure. *S*86 ^{13}C{¹H} NMR spectrum of **24** (126 MHz, C_6D_6, 298 K).

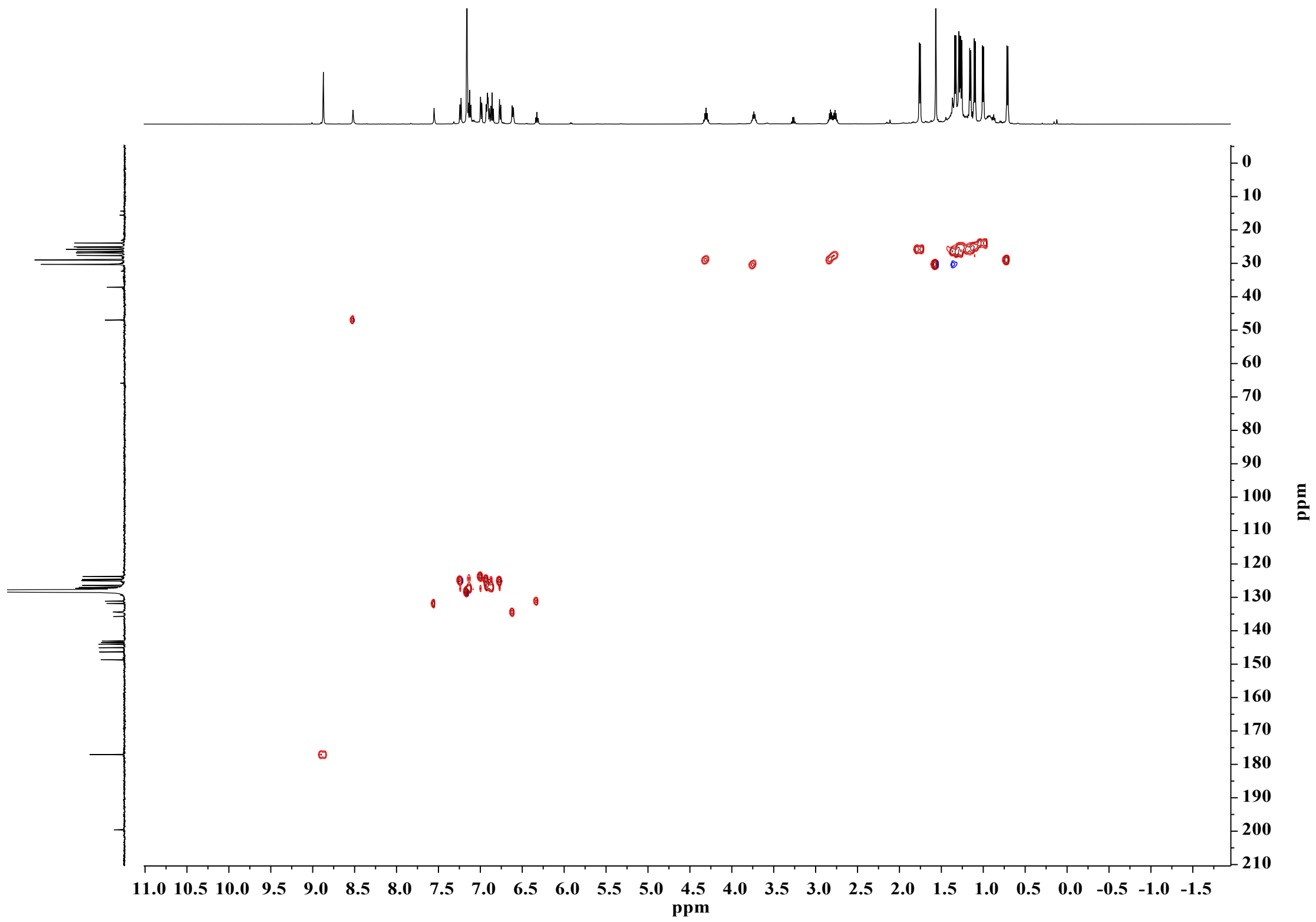

Figure. *S*87 HSQC (^{1}H-^{13}C) NMR spectrum of **24** (C_6D_6, 298 K).

4.25 $W_2Cl_2[\mu\text{-}\eta^2\text{:}\eta^2\text{-}1\text{-}n\text{-}C_3H_7\text{-}4\text{-}^tBuH\text{-}C_4H][\mu\text{-}\kappa^2\text{-}HC(N\text{-}2,6\text{-}^iPr_2C_6H_3)_2]_2$ (25)

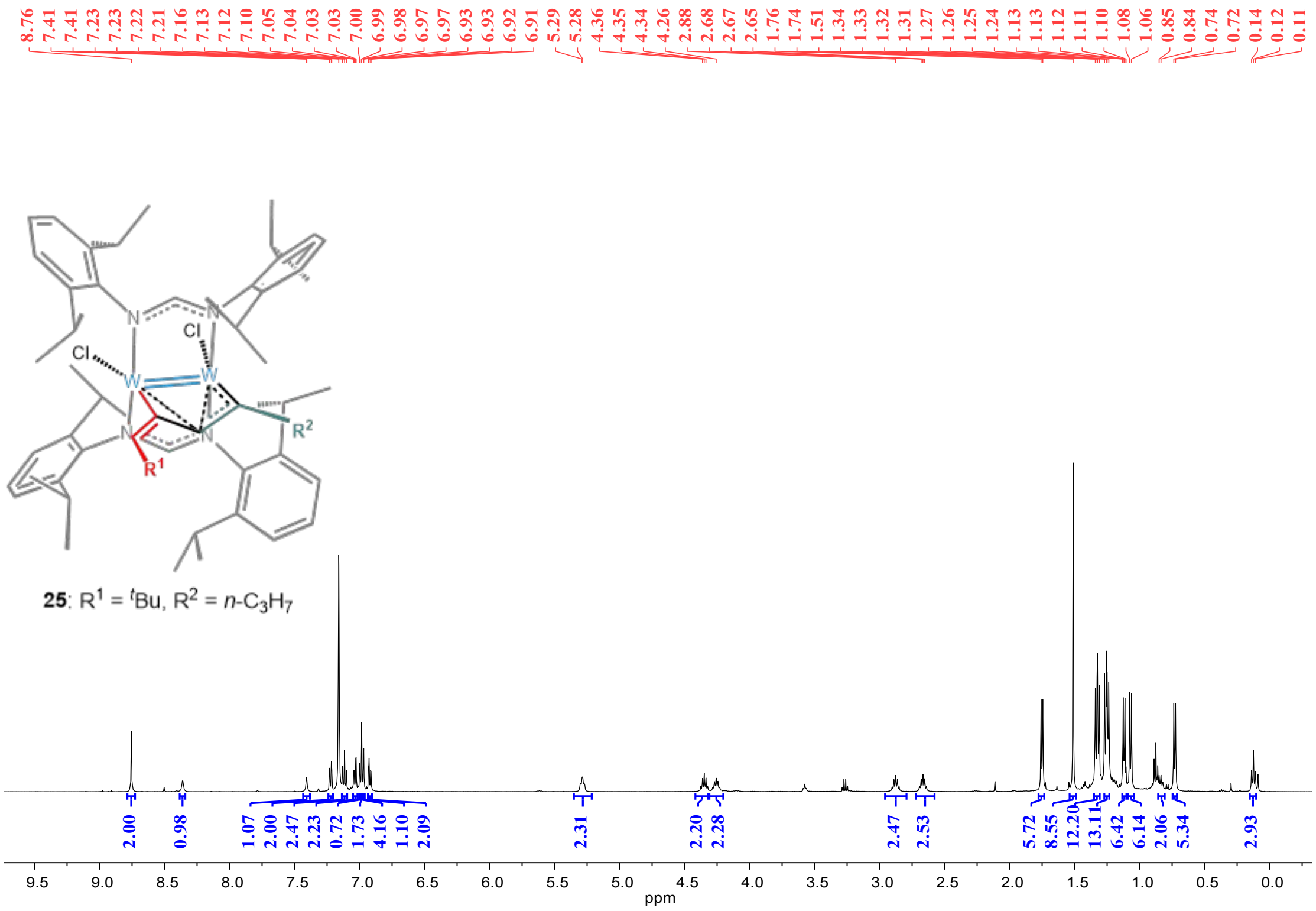

Figure. *S*88 ^{1}H NMR spectrum of **25** (500 MHz, C_6D_6, 298 K).

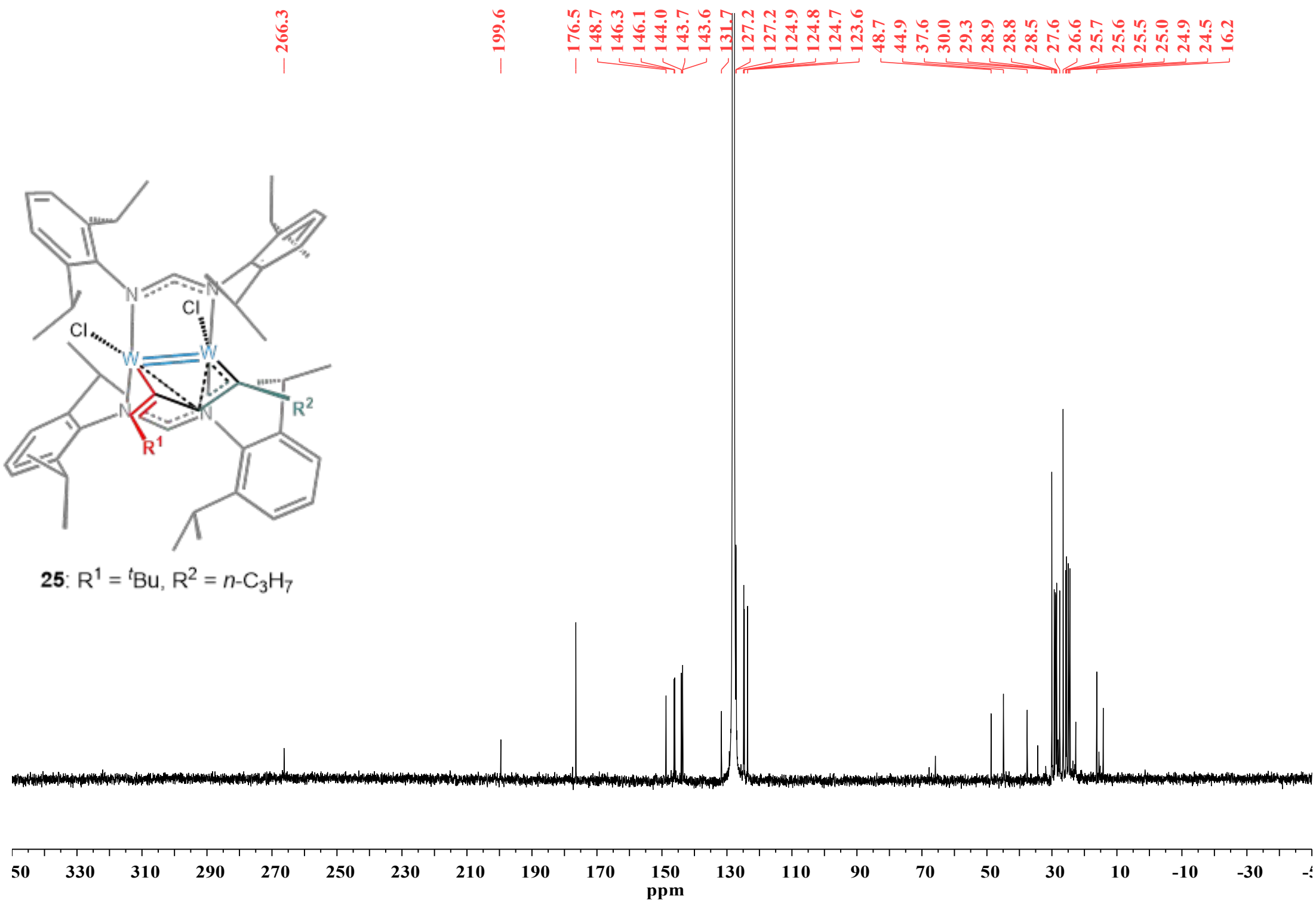

Figure. *S*89 $^{13}C\{^1H\}$ NMR spectrum of **25** (126 MHz, C_6D_6, 298 K).

4.26 $W_2Cl_2[\mu\text{-}\eta^2\text{:}\eta^2\text{-}1\text{-}n\text{-}C_3H_7\text{-}4\text{-}SiMe_3H\text{-}C_4H][\mu\text{-}\kappa^2\text{-}HC(N\text{-}2,6\text{-}{}^iPr_2C_6H_3)_2]_2$ (26)

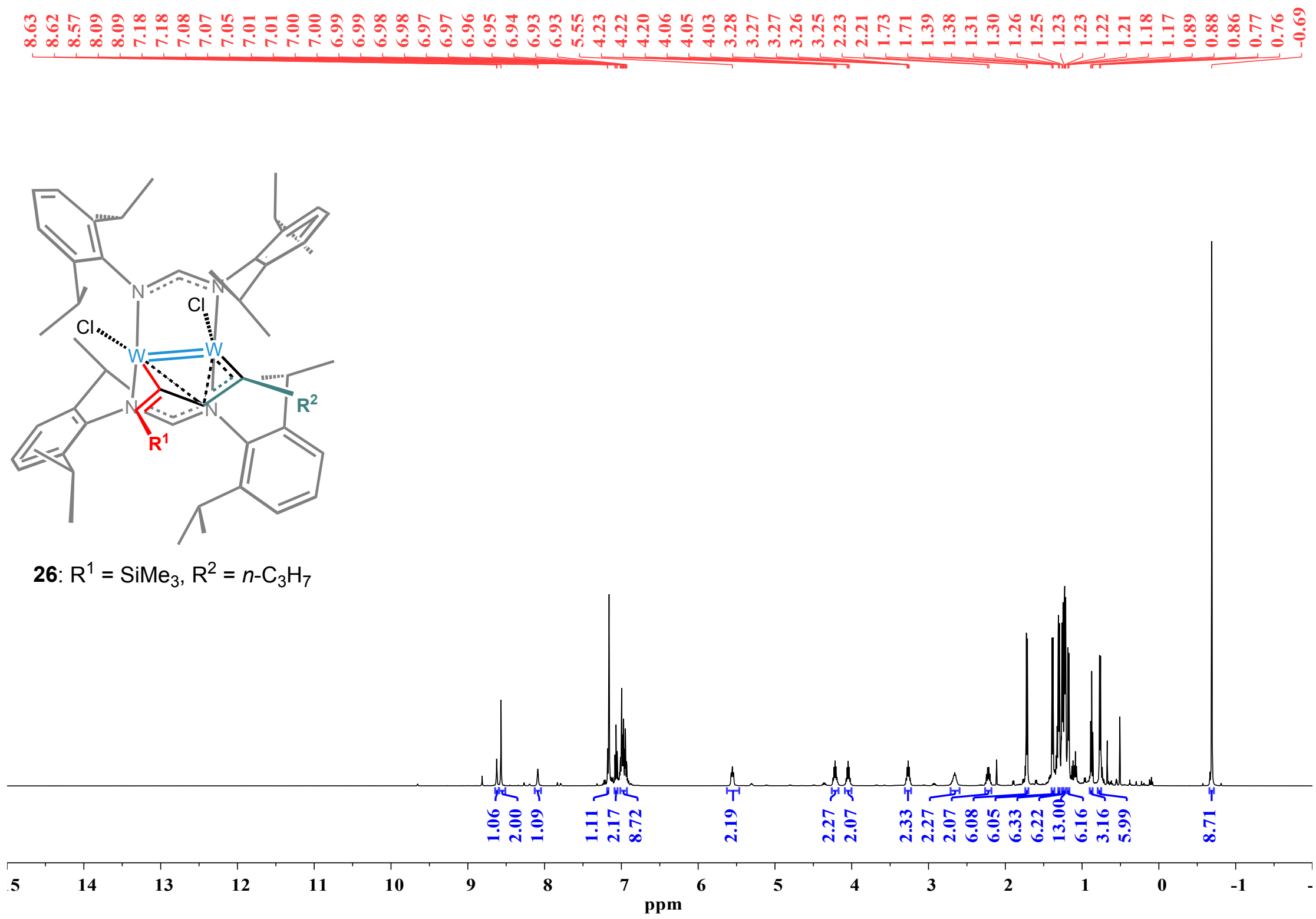

Figure. *S*90 ^{1}H NMR spectrum of **26** (500 MHz, C_6D_6, 298 K).

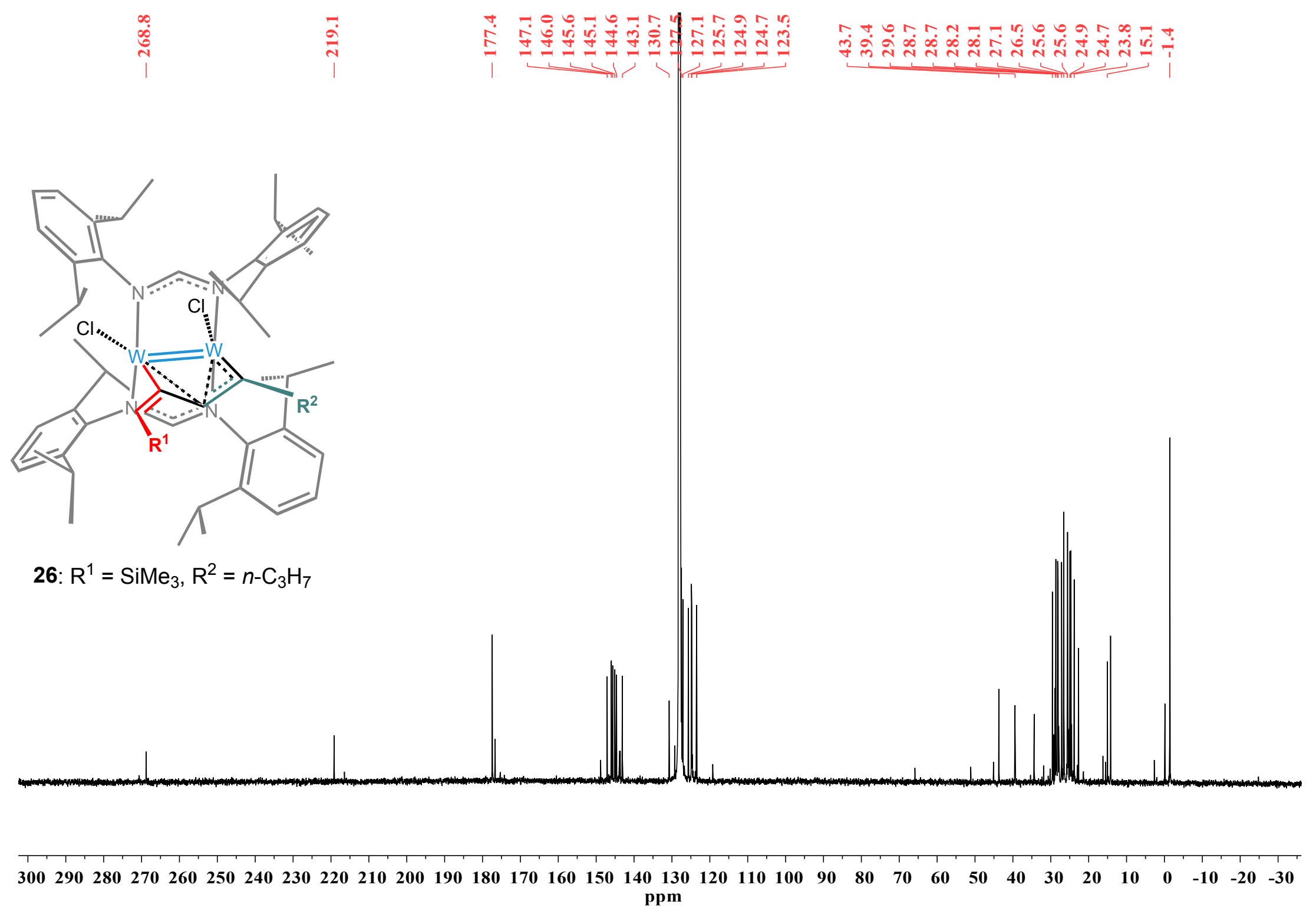

Figure. *S*91 $^{13}C\{^1H\}$ NMR spectrum of **26** (126 MHz, C_6D_6, 298 K).

4.27 $W_2Cl[\mu$-κ^2-1-C_6H_5-4-^{t}BuH-$C_4H][\mu$-κ^2-HC(N-2,6-^{i}Pr$_2C_6H_3)_2]_2$ (27)

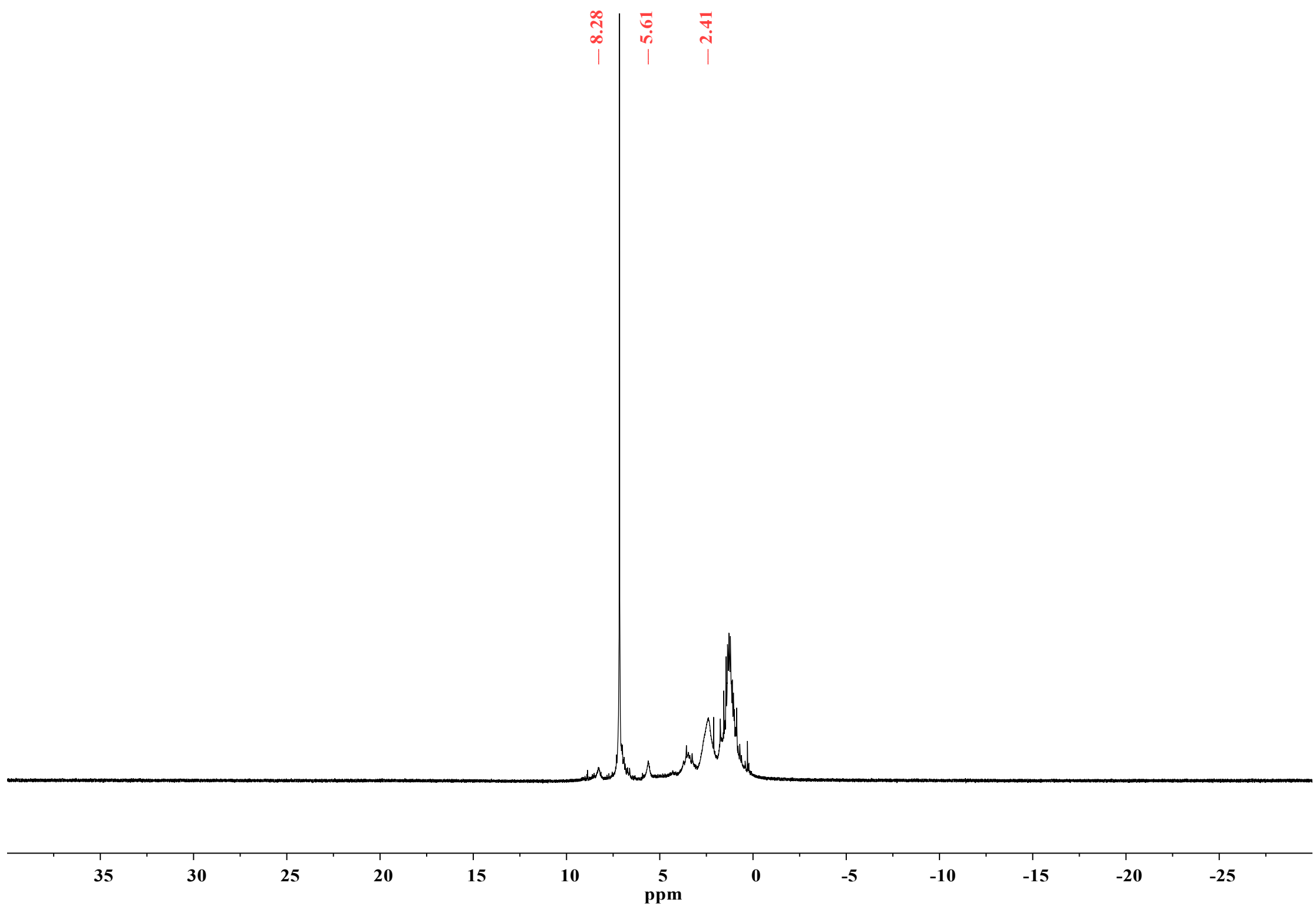

Figure. *S*92 ^{1}H NMR spectrum of **27** (500 MHz, C_6D_6, 298 K).

4.28 $W_2(\mu\text{-H})[\mu\text{-}\kappa^2\text{-1-}C_6H_5\text{-4-}^tBuH\text{-}C_4H][\mu\text{-}\kappa^3\text{-HC(N-2,6-}^iPr_2C_6H_3)(N\text{-2-}^iPr\text{-6-}(C_3H_6)C_6H_3)][\mu\text{-}\kappa^2\text{-HC(N-2,6-}^iPr_2C_6H_3)_2]$ (28)

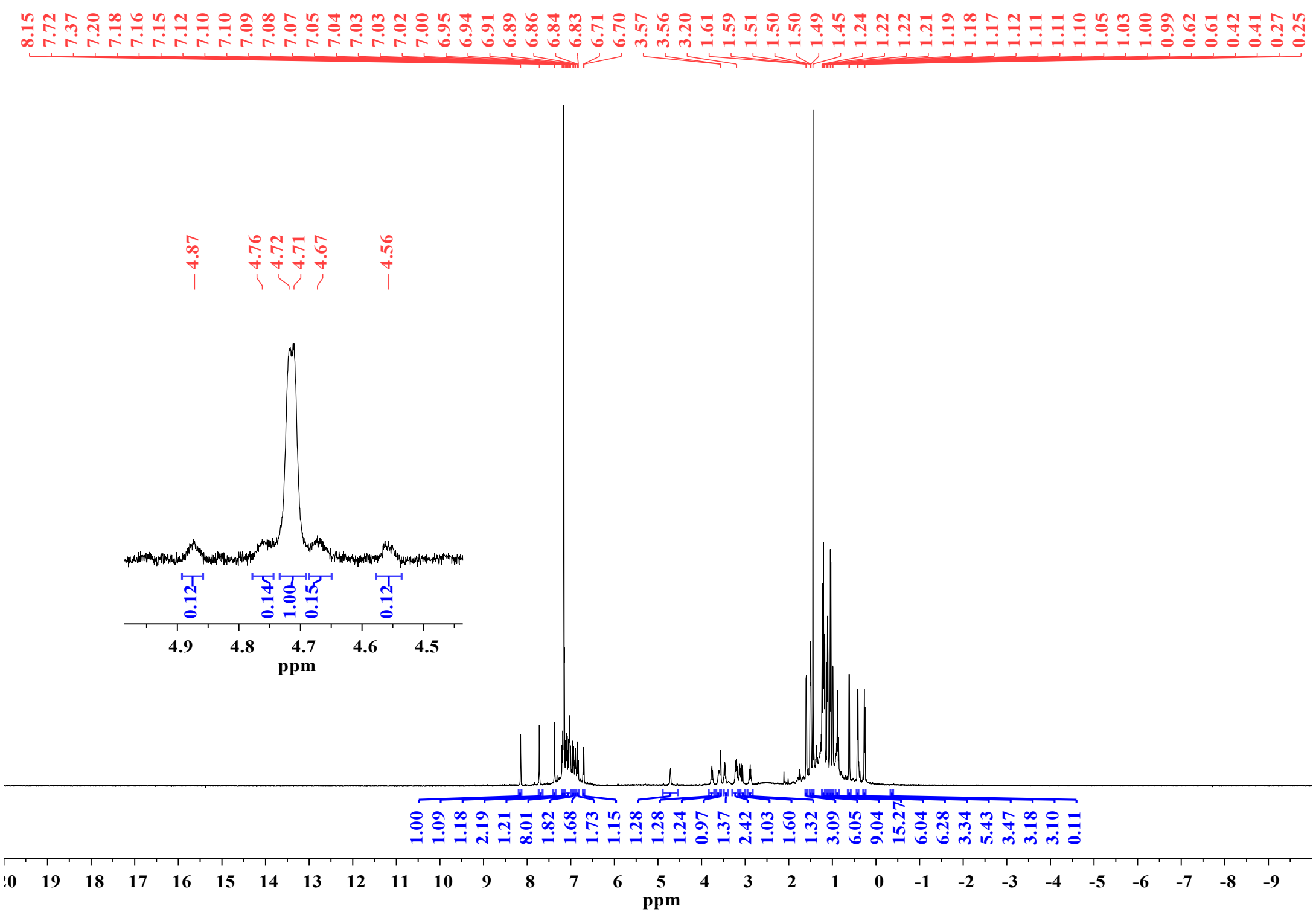

Figure. *S*93 ^{1}H NMR spectrum of **28** (500 MHz, C_6D_6, 298 K).

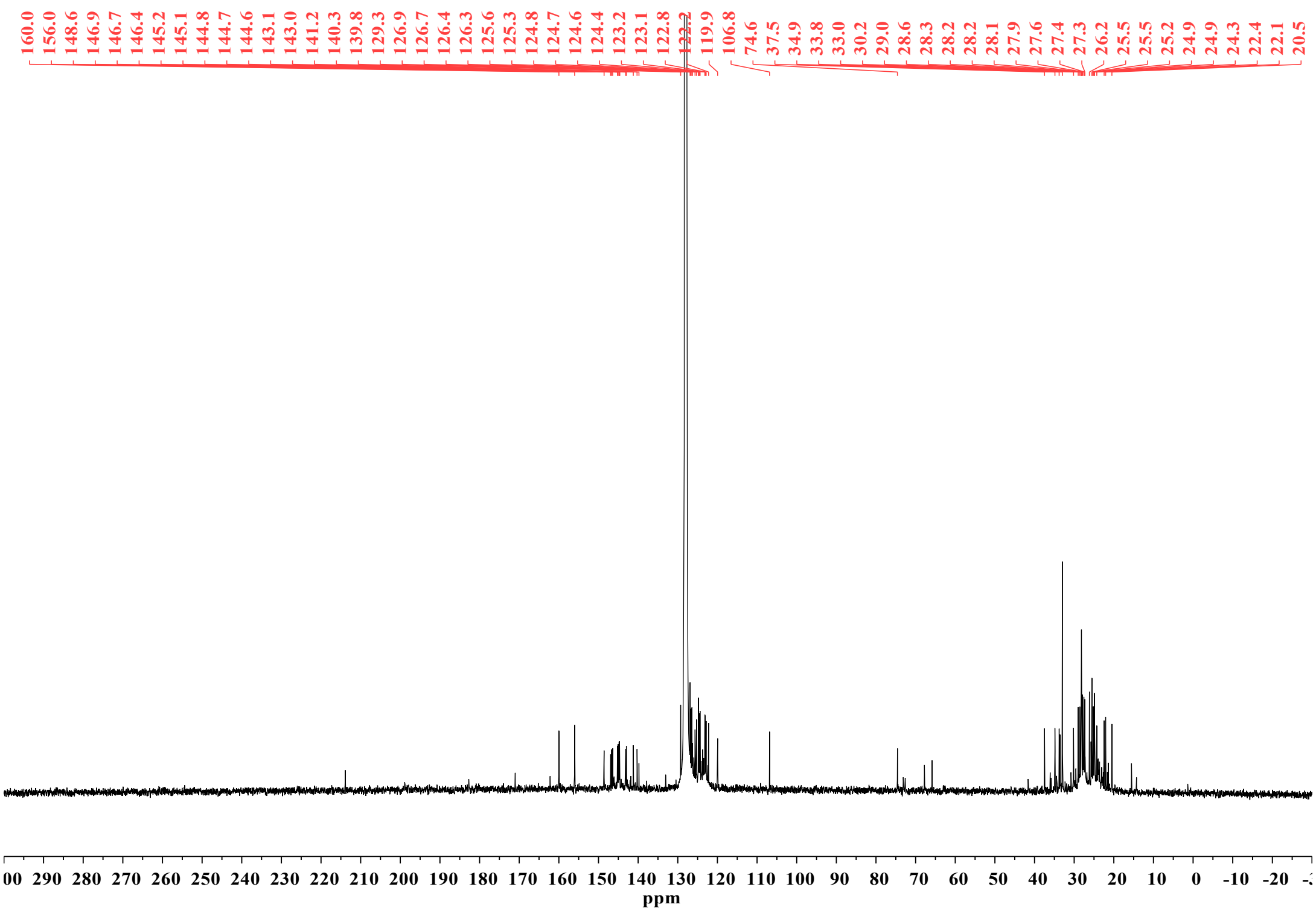

Figure. *S*94 $^{13}C\{^1H\}$ NMR spectrum of **28** (126 MHz, C_6D_6, 298 K).

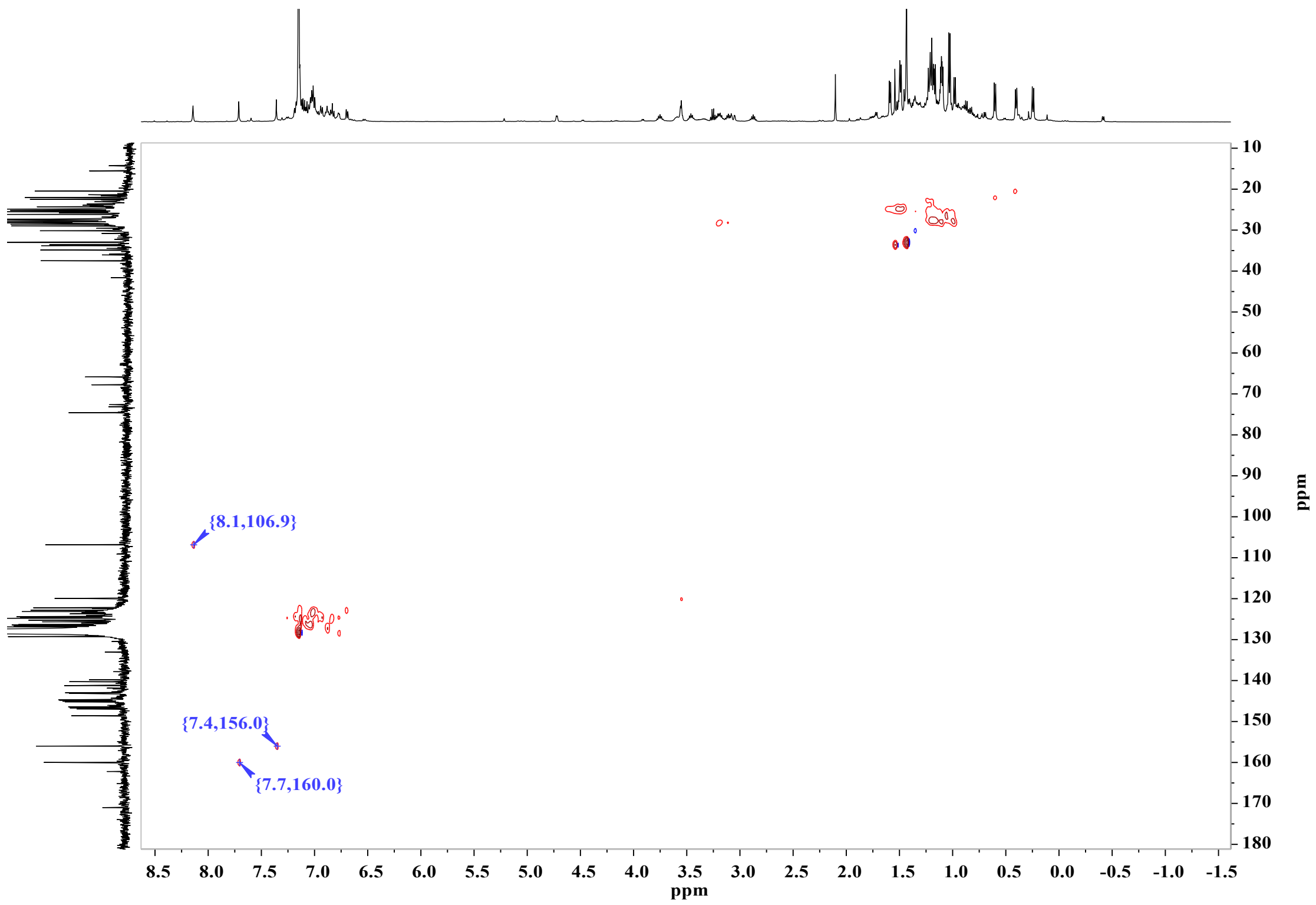

Figure. *S*95 HSQC (^{1}H-^{13}C) NMR spectrum of **28** (C_6D_6, 298 K).

5 Evans method

Effective magnetic moment was determined by Evans method.[9–11] Sample was dissolved in the mixture of C_6D_6 and TMS_2O, and ^1H NMR spectra were measured at 298 K with the capillary of the mixture of C_6D_6 and TMS_2O as internal standard. Effective magnetic moments were determined by following equation, and diamagnetic constants were calculated by Pascal's constants.[12]

$$x_g = \frac{3\Delta f}{4\pi f m} + \frac{x_0(D_{solvent} - D_{mix})}{m} + x_0$$

$$x_m = x_g(MW) - x_d$$

$$\mu_{eff} = \sqrt{8x_m T}$$

Δf: chemical shift difference; f: operating frequency of ^1H NMR; m: concentration of sample in solvent (g/mL); χ_0: mass susceptibility of solvent; χ_d: diamagnetic correction of sample.

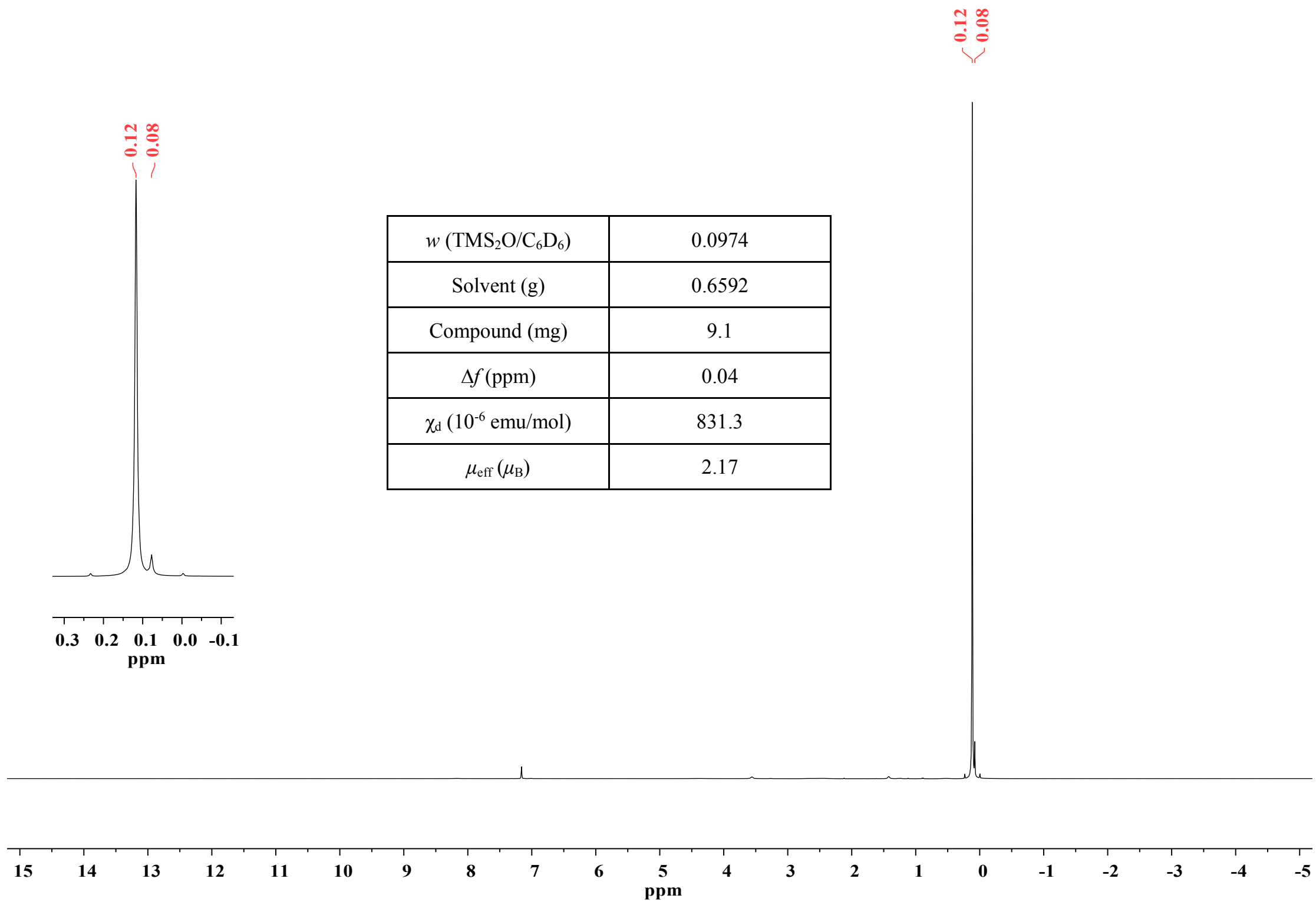

w (TMS_2O/C_6D_6)	0.0974
Solvent (g)	0.6592
Compound (mg)	9.1
Δf (ppm)	0.04
χ_d (10^{-6} emu/mol)	831.3
μ_{eff} (μ_B)	2.17

Figure. *S*96 Evans method ^{1}H NMR spectrum of **1** (500 mHz, C_6D_6, 298 K).

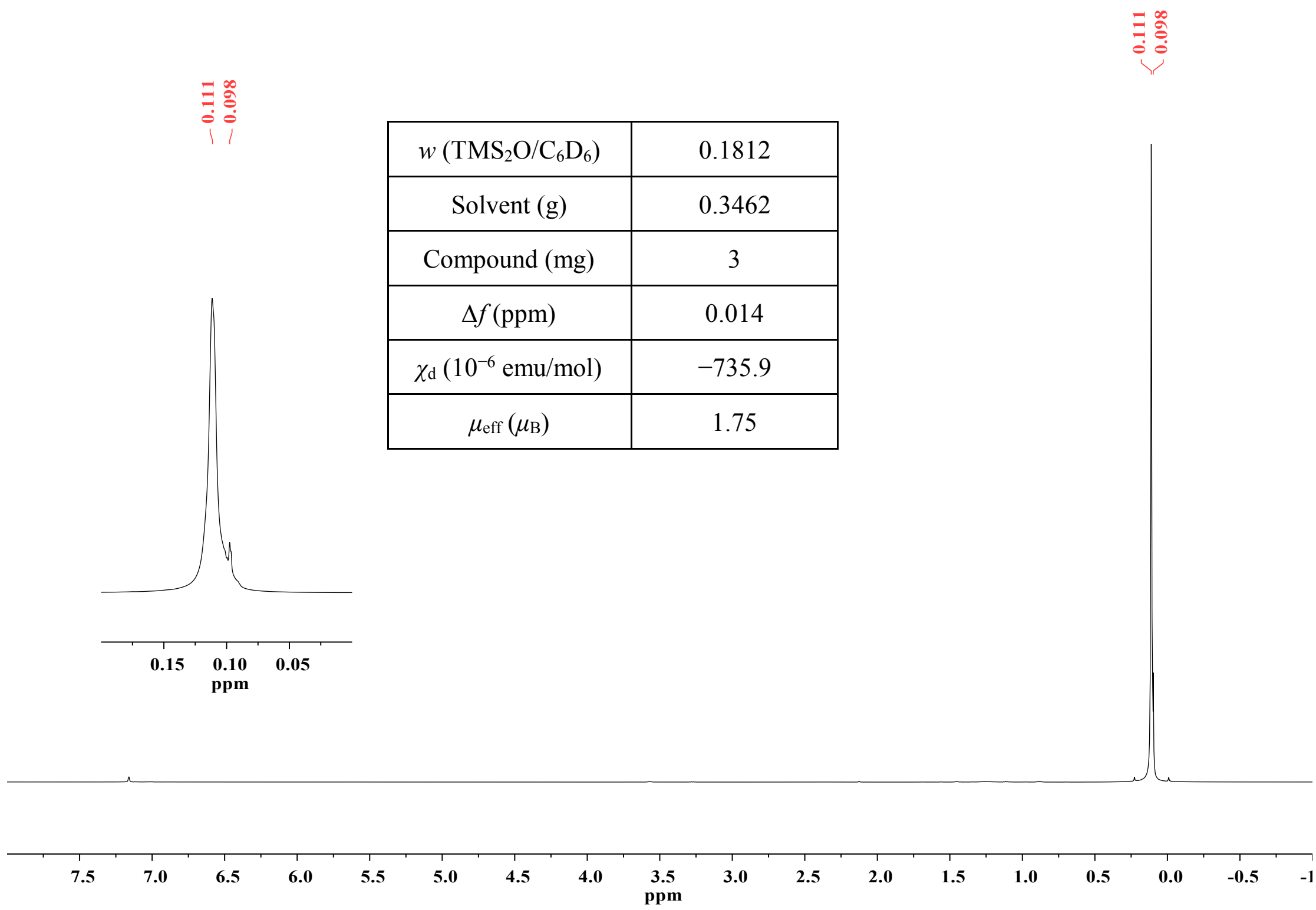

w (TMS_2O/C_6D_6)	0.1812
Solvent (g)	0.3462
Compound (mg)	3
Δf (ppm)	0.014
χ_d (10^{-6} emu/mol)	−735.9
μ_{eff} (μ_B)	1.75

Figure. *S*97 Evans method ^{1}H NMR spectrum of **27** (500 mHz, C_6D_6, 298 K).

6 Elemental analysis

National Chung Hsing University

The Office of Research and Development, Instrument Center

Report on Elemental Analysis

Instrument Director : Prof. Chen-Yu Yeh

Operator : I-Chuan Chen

Note: This result is for academic use only, not to be used for any judicial or commercial advertising purpose.

Information of Submitted Sample

Web NO :	BSEA0001002022080051		
Department :	National Tsing Hua University Department of chemistry	Date of	
Supervisor :	Prof. Yi-Chou Tsai	Receive:	2022.08.25
User name :	Cian-Wei Yang	completion:	2022.08.25

Results of Analysis

Sample code	*Weight(mg)*	*N %*	*C %*	*H %*	*O %*	*S %*	*Repeat*	*Charge*
CW-230233lt	5.188	4.09	52.43	6.54				$ 320

Instrumentation: *Elementar vario EL CUBE (CHN-OS Rapid, German), Accuracy: 0.1%, Precision: 0.2%*

	Standard sample	*N%*	*C %*	*H %*	*O %*	*S %*
★	*Acetanilide*	*10.36*	*71.09*	*6.71*		
	Benzoic acid				*26.20*	
	Sulfanilic acid	*8.09*	*41.60*	*4.07*		*18.50*
	Daily standard	*10.33*	*71.12*	*6.78*		

Figure. *S*98 Elemental analysis report of **1**

National Chung Hsing University

The Office of Research and Development, Instrument Center

Report on Elemental Analysis

Instrument Director : Prof. Chen-Yu Yeh

Operator : I-Chuan Chen

Note: This result is for academic use only, not to be used for any judicial or commercial advertising purpose.

Information of Submitted Sample

Web NO :	BSEA0001002023050024		
Department :	National Tsing Hua University Department of chemistry		Date of
Supervisor :	Prof. Yi-Chou Tsai	Receive:	2023.05.11
User name :	Cian-Wei Yang	completion:	2023.05.11

Results of Analysis

Sample code	*Weight(mg)*	*N %*	*C %*	*H %*	*O %*	*S %*	*Repeat*	*Charge*
CW-221284lt	4.618	4.34	51.00	6.41				$ 320

Instrumentation: Elementar vario EL CUBE (CHN-OS Rapid, German), Accuracy: 0.1%, Precision: 0.2%

	Standard sample	*N%*	*C%*	*H%*	*O%*	*S%*
★	*Acetanilide*	*10.36*	*71.09*	*6.71*		
	Benzoic acid				*26.20*	
	Sulfanilic acid	*8.09*	*41.60*	*4.07*		*18.50*
	Daily standard	*10.36*	*71.14*	*6.61*		

Figure. *S*99 Elemental analysis report of **2**

National Chung Hsing University

The Office of Research and Development, Instrument Center

Report on Elemental Analysis

Instrument Director : Prof. Chen-Yu Yeh

Operator : I-Chuan Chen

Note: This result is for academic use only, not to be used for any judicial or commercial advertising purpose.

Information of Submitted Sample

Web NO :	BSEA0001002023030016		
Department :	National Tsing Hua University		Date of
	Department of chemistry		
Supervisor :	Prof. Yi-Chou Tsai	Receive:	2023.03.09
User name :	Cian-Wei Yang	completion:	2023.03.09

Results of Analysis

Sample code	*Weight(mg)*	*N %*	*C %*	*H %*	*O %*	*S %*	*Repeat*	*Charge*
CW-230134lt	0.922	5.98	53.75	5.73				$ 320

Instrumentation: *Elementar vario EL CUBE (CHN-OS Rapid, German), Accuracy: 0.1%, Precision: 0.2%*

	Standard sample	*N%*	*C%*	*H%*	*O%*	*S%*
★	*Acetanilide*	*10.36*	*71.09*	*6.71*		
	Benzoic acid				*26.20*	
	Sulfanilic acid	*8.09*	*41.60*	*4.07*		*18.50*
	Daily standard	*10.35*	*71.09*	*6.66*		

Figure. *S*100 Elemental analysis report of **3**

Precision Instrumentation Center--Elemental Analysis Report

College of Science, National Taiwan University

民國 86 年申請服務標章的校徽

Remark: 1. This result is for academic use only, not to be used for any judicial or commercial advertising purpose.

2. Instrument： elementar Vario EL cube 型 (for NCSH, German)

Accuracy: ± 0.1%
Precision: ± 0.2%
Inaccuracy of instrument: ± 0.3%

3. Instrument Director: Prof. Wong, Ken-Tsung Operator: Ching-Wei Lu

Web. NO.	52021050005
User name	Chiao-Yun Chen
Supervisor	Prof. Yi-Chou Tsai
University or Institute	National Tsing Hua University Department of chemistry
Acceptance date	2021/5/12
Analysis date	2021/5/12

Sample code	Date Time	Weight(mg)	Grp	N%	C%	S%	H%	Repeat	Charge
acetanilide	12.05.2021	4.033	standard	10.360	71.090	0.000	6.710		
standard daily data	12.05.2021	3.667		10.350	71.177	0.000	6.796		
CY-Wch	12.05.2021	4.318	sensitive	4.535	55.262		7.147	1	$ 400

Figure. *S*101 Elemental analysis report of **4**

Precision Instrumentation Center--Elemental Analysis Report

College of Science, National Taiwan University

民國 86 年申請服務標章的校徽

Remark: 1. This result is for academic use only, not to be used for any judicial or commercial advertising purpose.

2. Instrument： elementar Vario EL cube 型 (for NCSH, German)

Accuracy: ± 0.1%
Precision: ± 0.2%
Inaccuracy of instrument: ± 0.3%

3. Instrument Director: Prof. Wong, Ken-Tsung   Operator: Ching-Wei Lu

Web. NO.	52020110005
User name	Chiao-Yun Chen
Supervisor	Prof. Yi-Chou Tsai
University or Institute	National Tsing Hua University Department of chemistry
Acceptance date	2020/11/25
Analysis date	2020/11/25

Sample code	Date Time	Weight(mg)	Grp	N%	C%	S%	H%	Repeat	Charge
acetanilide	25.11.2020	3.588	standard	10.360	71.090	0.000	6.710		
standard daily data	25.11.2020	3.809		10.355	71.134	0.000	6.699		
CY2-201-2PhCCH	25.11.2020	1.432	sensitive	4.289	60.965		6.934	1	$ 2,000

Figure. *S*102 Elemental analysis report of **5**

National Chung Hsing University

The Office of Research and Development, Instrument Center

Report on Elemental Analysis

Instrument Director : Prof. Chen-Yu Yeh

Operator : I-Chuan Chen

Note: This result is for academic use only, not to be used for any judicial or commercial advertising purpose.

Information of Submitted Sample

Web NO :	BSEA0001002023080009		
Department :	National Tsing Hua University Department of chemistry		Date of
Supervisor :	Prof. Yi-Chou Tsai	Receive:	2023.08.03
User name :	Cian-Wei Yang	completion:	2023.08.03

Results of Analysis

Sample code	*Weight(mg)*	*N %*	*C %*	*H %*	*O %*	*S %*	*Repeat*	*Charge*
CW-230270lt	1.172	3.72	61.04	6.28				$ 320

Instrumentation: *Elementar vario EL CUBE (CHN-OS Rapid, German), Accuracy: 0.1%, Precision: 0.2%*

	Standard sample	*N%*	*C%*	*H%*	*O%*	*S%*
★	*Acetanilide*	*10.36*	*71.09*	*6.71*		
	Benzoic acid				*26.20*	
	Sulfanilic acid	*8.09*	*41.60*	*4.07*		*18.50*
	Daily standard	*10.40*	*71.04*	*6.67*		

Figure. *S*103 Elemental analysis report of **6**

National Chung Hsing University

The Office of Research and Development, Instrument Center

Report on Elemental Analysis

Instrument Director : Prof. Chen-Yu Yeh

Operator : I-Chuan Chen

Note: This result is for academic use only, not to be used for any judicial or commercial advertising purpose.

Information of Submitted Sample

Web NO :	BSEA00010020220120030		
Department :	National Tsing Hua University Department of chemistry		Date of
Supervisor :	Prof. Yi-Chou Tsai	Receive:	2022.12.23
User name :	Cian-Wei Yang	completion:	2022.12.23

Results of Analysis

Sample code	*Weight(mg)*	*N %*	*C %*	*H %*	*O %*	*S %*	*Repeat*	*Charge*
CW-221196lt	3.222	4.14	60.96	6.54				$ 320

Instrumentation: *Elementar vario EL CUBE (CHN-OS Rapid, German), Accuracy: 0.1%, Precision: 0.2%*

	Standard sample	*N %*	*C %*	*H %*	*O %*	*S %*
★	*Acetanilide*	*10.36*	*71.09*	*6.71*		
	Benzoic acid				*26.20*	
	Sulfanilic acid	*8.09*	*41.60*	*4.07*		*18.50*
	Daily standard	*10.33*	*71.14*	*6.78*		

Figure. *S*104 Elemental analysis report of **7**

National Chung Hsing University

The Office of Research and Development, Instrument Center

Report on Elemental Analysis

Instrument Director : Prof. Chen-Yu Yeh

Operator : I-Chuan Chen

Note: This result is for academic use only, not to be used for any judicial or commercial advertising purpose.

Information of Submitted Sample

Web NO :	BSEA0001002022120030		
Department :	National Tsing Hua University<br>Department of chemistry		Date of
Supervisor :	Prof. Yi-Chou Tsai	Receive:	2022.12.23
User name :	Cian-Wei Yang	completion:	2022.12.23

Results of Analysis

Sample code	*Weight(mg)*	*N %*	*C %*	*H %*	*O %*	*S %*	*Repeat*	*Charge*
CW-221222lt	2.414	4.16	61.08	6.41				$ 320

Instrumentation: *Elementar vario EL CUBE (CHN-OS Rapid, German), Accuracy: 0.1%, Precision: 0.2%*

	Standard sample	*N%*	*C%*	*H%*	*O%*	*S%*
★	*Acetanilide*	*10.36*	*71.09*	*6.71*		
	Benzoic acid				*26.20*	
	Sulfanilic acid	*8.09*	*41.60*	*4.07*		*18.50*
	Daily standard	*10.33*	*71.14*	*6.78*		

Figure. *S*105 Elemental analysis report of **8**

Precision Instrumentation Center--Elemental Analysis Report

College of Science, National Taiwan University

民國 86 年申請服務標章的校徽

Remark: 1. This result is for academic use only, not to be used for any judicial or commercial advertising purpose.

2. Instrument： elementar Vario EL cube 型 (for NCSH, German)

Accuracy: ± 0.1%
Precision: ± 0.2%
Inaccuracy of instrument: ± 0.3%

3. Instrument Director: Prof. Wong, Ken-Tsung Operator: Ching-Wei Lu

Web. NO.	52021030012
User name	Chiao-Yun Chen
Supervisor	Prof. Yi-Chou Tsai
University or Institute	National Tsing Hua University Department of chemistry
Acceptance date	2021/3/24
Analysis date	2021/3/24

Sample code	Date Time	Weight(mg)	Grp	N%	C%	S%	H%	Repeat	Charge
acetanilide	24.03.2021	3.814	standard	10.360	71.090	0.000	6.710		
standard daily data	24.03.2021	3.728		10.377	70.987	0.000	6.692		
CY-MeOPhCCH	24.03.2021	1.807	sensitive	4.192	60.016		6.610	1	$ 400

Figure. *S*106 Elemental analysis report of **9**

Precision Instrumentation Center--Elemental Analysis Report

College of Science, National Taiwan University

Remark: 1. This result is for academic use only, not to be used for any judicial or commercial advertising purpose.

2. Instrument： elementar Vario EL cube 型 (for NCSH, German)

Accuracy: ± 0.1%
Precision: ± 0.2%
Inaccuracy of instrument: ± 0.3%

3. Instrument Director: Prof. Wong, Ken-Tsung Operator: Ching-Wei Lu

Web. NO.	52020110005
User name	Chiao-Yun Chen
Supervisor	Prof. Yi-Chou Tsai
University or Institute	National Tsing Hua University Department of chemistry
Acceptance date	2020/11/25
Analysis date	2020/11/25

Sample code	Date Time	Weight(mg)	Grp	N%	C%	S%	H%	Repeat	Charge
acetanilide	25.11.2020	3.588	standard	10.360	71.090	0.000	6.710		
standard daily data	25.11.2020	3.809		10.355	71.134	0.000	6.699		

Figure. *S*107 Elemental analysis report of **10**

National Chung Hsing University

The Office of Research and Development, Instrument Center

Report on Elemental Analysis

Instrument Director : Prof. Chen-Yu Yeh

Operator : I-Chuan Chen

Note: This result is for academic use only, not to be used for any judicial or commercial advertising purpose.

Information of Submitted Sample

Web NO :	BSEA0001002022100033		
Department :	National Tsing Hua University Department of chemistry		Date of
Supervisor :	Prof. Yi-Chou Tsai	Receive:	2022.10.27
User name :	Cian-Wei Yang	completion:	2022.10.27

Results of Analysis

Sample code	*Weight(mg)*	*N %*	*C %*	*H %*	*O %*	*S %*	*Repeat*	*Charge*
CW-221037lt2	0.466	4.83	58.05	6.76				$ 320

Instrumentation: *Elementar vario EL CUBE (CHN-OS Rapid, German), Accuracy: 0.1%, Precision: 0.2%*

	Standard sample	*N %*	*C %*	*H %*	*O %*	*S %*
★	*Acetanilide*	*10.36*	*71.09*	*6.71*		
	Benzoic acid				*26.20*	
	Sulfanilic acid	*8.09*	*41.60*	*4.07*		*18.50*
	Daily standard	*10.35*	*71.06*	*6.69*		

Figure. *S*108 Elemental analysis report of **11**

National Chung Hsing University

The Office of Research and Development, Instrument Center

Report on Elemental Analysis

Instrument Director : Prof. Chen-Yu Yeh

Operator : I-Chuan Chen

Note: This result is for academic use only, not to be used for any judicial or commercial advertising purpose.

Information of Submitted Sample

Web NO :	BSEA0001002022110003		
Department :	National Tsing Hua University<br>Department of chemistry		Date of
Supervisor :	Prof. Yi-Chou Tsai	Receive:	2022.11.03
User name :	Cian-Wei Yang	completion:	2022.11.03

Results of Analysis

Sample code	Weight(mg)	N %	C %	H %	O %	S %	Repeat	Charge
CW-230108lt	1.614	3.91	57.70	5.78				$ 320

Instrumentation: *Elementar vario EL CUBE (CHN-OS Rapid, German), Accuracy: 0.1%, Precision: 0.2%*

	Standard sample	N %	C %	H %	O %	S %
★	Acetanilide	10.36	71.09	6.71		
	Benzoic acid				26.20	
	Sulfanilic acid	8.09	41.60	4.07		18.50
	Daily standard	10.41	71.04	6.75		

Figure. *S*109 Elemental analysis report of **12**

National Chung Hsing University

The Office of Research and Development, Instrument Center

Report on Elemental Analysis

Instrument Director : Prof. Chen-Yu Yeh

Operator : I-Chuan Chen

Note: This result is for academic use only, not to be used for any judicial or commercial advertising purpose.

Information of Submitted Sample

Web NO :	BSEA0001002023030049		
Department :	National Tsing Hua University		Date of
	Department of chemistry		
Supervisor :	Prof. Yi-Chou Tsai	Receive:	2023.03.23
User name :	Cian-Wei Yang	completion:	2023.03.23

Results of Analysis

Sample code	*Weight(mg)*	*N %*	*C %*	*H %*	*O %*	*S %*	*Repeat*	*Charge*
CW-230234lt	0.703	3.59	58.62	6.07				$ 320

Instrumentation: *Elementar vario EL CUBE (CHN-OS Rapid, German), Accuracy: 0.1%, Precision: 0.2%*

	Standard sample	*N %*	*C %*	*H %*	*O %*	*S %*
★	*Acetanilide*	*10.36*	*71.09*	*6.71*		
	Benzoic acid				*26.20*	
	Sulfanilic acid	*8.09*	*41.60*	*4.07*		*18.50*
	Daily standard	*10.46*	*71.06*	*6.66*		

Figure. *S*110 Elemental analysis report of **13**

National Chung Hsing University

The Office of Research and Development, Instrument Center

Report on Elemental Analysis

Instrument Director : Prof. Chen-Yu Yeh

Operator : I-Chuan Chen

Note: This result is for academic use only, not to be used for any judicial or commercial advertising purpose.

Information of Submitted Sample

Web NO :	BSEA0001002024030010		
Department :	National Tsing Hua University Department of chemistry		Date of
Supervisor :	Prof. Yi-Chou Tsai	Receive:	2024.03.07
User name :	Wen-Chuan Hsu	completion:	2024.03.07

Results of Analysis

Sample code	*Weight(mg)*	*N %*	*C %*	*H %*	*O %*	*S %*	*Repeat*	*Charge*
VC3-W_4Py-4OMe-89	1.943	3.80	57.31	5.84				$ 320

Instrumentation: *Elementar vario EL CUBE (CHN-OS Rapid, German), Accuracy: 0.1%, Precision: 0.2%*

	Standard sample	*N %*	*C %*	*H %*	*O %*	*S %*
★	*Acetanilide*	*10.36*	*71.09*	*6.71*		
	Benzoic acid				*26.20*	
	Sulfanilic acid	*8.09*	*41.60*	*4.07*		*18.50*
	Daily standard	*10.39*	*71.14*	*6.63*		

Figure. *S*111 Elemental analysis report of **14**

National Chung Hsing University

The Office of Research and Development, Instrument Center

Report on Elemental Analysis

Instrument Director : Prof. Chen-Yu Yeh

Operator : I-Chuan Chen

Note: This result is for academic use only, not to be used for any judicial or commercial advertising purpose.

Information of Submitted Sample

Web NO :	BSEA0001002023040031		
Department :	National Tsing Hua University Department of chemistry	Date of	
Supervisor :	Prof. Yi-Chou Tsai	Receive:	2023.04.20
User name :	Cian-Wei Yang	completion:	2023.04.20

Results of Analysis

Sample code	*Weight(mg)*	*N %*	*C %*	*H %*	*O %*	*S %*	*Repeat*	*Charge*
CW-230109LT	3.306	4.10	55.12	6.44				$ 320

Instrumentation: *Elementar vario EL CUBE (CHN-OS Rapid, German), Accuracy: 0.1%, Precision: 0.2%*

	Standard sample	*N%*	*C%*	*H%*	*O%*	*S%*
★	*Acetanilide*	*10.36*	*71.09*	*6.71*		
	Benzoic acid				*26.20*	
	Sulfanilic acid	*8.09*	*41.60*	*4.07*		*18.50*
	Daily standard	*10.41*	*71.14*	*6.68*		

Figure. *S*112 Elemental analysis report of **15**

National Chung Hsing University

The Office of Research and Development, Instrument Center

Report on Elemental Analysis

Instrument Director : Prof. Bao-Tsan Ko

Operator : I-Chuan Chen

Note: This result is for academic use only, not to be used for any judicial or commercial advertising purpose.

Information of Submitted Sample

Web NO :	BSEA0001002026010007		
Department :	National Tsing Hua University Department of chemistry		Date of
Supervisor :	Prof. Yi-Chou Tsai	Receive:	2026.01.08
User name :	Wen-Chi Chen	completion:	2026.01.08

Results of Analysis

Sample code	*Weight(mg)*	*N %*	*C %*	*H %*	*O %*	*S %*	*Repeat*	*Charge*
1-WC3-Wint-1-P-78	0.906	3.91	56.66	6.29				$ 320

Instrumentation: Elementar vario EL CUBE (CHN-OS Rapid, German), Accuracy: 0.1%, Precision: 0.2%

	Standard sample	*N %*	*C %*	*H %*	*O %*	*S %*
★	*Acetanilide*	*10.36*	*71.09*	*6.71*		
	Benzoic acid				*26.20*	
	Sulfanilic acid	*8.09*	*41.60*	*4.07*		*18.50*
	Daily standard	*10.39*	*71.12*	*6.73*		

Figure. *S*113 Elemental analysis report of **16**

National Chung Hsing University

The Office of Research and Development, Instrument Center

Report on Elemental Analysis

Instrument Director : Prof. Bao-Tsan Ko

Operator : I-Chuan Chen

Note: This result is for academic use only, not to be used for any judicial or commercial advertising purpose.

Information of Submitted Sample

Web NO :	BSEA0001002026010044		
Department :	National Tsing Hua University<br>Department of chemistry		Date of
Supervisor :	Prof. Yi-Chou Tsai	Receive:	2026.01.29
User name :	Wen-Chi Chen	completion:	2026.01.29

Results of Analysis

Sample code	*Weight(mg)*	*N %*	*C %*	*H %*	*O %*	*S %*	*Repeat*	*Charge*
1-WC3- W2MePh-int-94	1.058	3.69	55.38	6.26				$ 320

Instrumentation: Elementar vario EL CUBE (CHN-OS Rapid, German), Accuracy: 0.1%, Precision: 0.2%

	Standard sample	*N %*	*C %*	*H %*	*O %*	*S %*
★	*Acetanilide*	*10.36*	*71.09*	*6.71*		
	Benzoic acid				*26.20*	
	Sulfanilic acid	*8.09*	*41.60*	*4.07*		*18.50*
	Daily standard	*10.34*	*71.08*	*6.72*		

Figure. *S*114 Elemental analysis report of **17**

National Chung Hsing University

The Office of Research and Development, Instrument Center

Report on Elemental Analysis

Instrument Director : Prof. Bao-Tsan Ko

Operator : I-Chuan Chen

Note: This result is for academic use only, not to be used for any judicial or commercial advertising purpose.

Information of Submitted Sample

Web NO :	BSEA0001002026030045		
Department :	National Tsing Hua University Department of chemistry		Date of
Supervisor :	Prof. Yi-Chou Tsai	Receive:	2026.03.19
User name :	Wen-Chi Chen	completion:	2026.03.19

Results of Analysis

Sample code	*Weight(mg)*	*N %*	*C %*	*H %*	*O %*	*S %*	*Repeat*	*Charge*
1-WC3-W2Me1-P-222-re-121	1.566	4.33	59.43	6.59				$ 320

Instrumentation: Elementar vario EL CUBE (CHN-OS Rapid, German), Accuracy: 0.1%, Precision: 0.2%

	Standard sample	*N %*	*C %*	*H %*	*O %*	*S %*
★	*Acetanilide*	*10.36*	*71.09*	*6.71*		
	Benzoic acid				*26.20*	
	Sulfanilic acid	*8.09*	*41.60*	*4.07*		*18.50*
	Daily standard	*10.38*	*71.01*	*6.63*		

Figure. *S*115 Elemental analysis report of **18**

National Chung Hsing University

The Office of Research and Development, Instrument Center

Report on Elemental Analysis

Instrument Director : Prof. Bao-Tsan Ko

Operator : I-Chuan Chen

Note: This result is for academic use only, not to be used for any judicial or commercial advertising purpose.

Information of Submitted Sample

Web NO :	BSEA0001002026010007		
Department :	National Tsing Hua University<br>Department of chemistry	Date of	
Supervisor :	Prof. Yi-Chou Tsai	Receive:	2026.01.08
User name :	Wen-Chi Chen	completion:	2026.01.08

Results of Analysis

Sample code	*Weight(mg)*	*N %*	*C %*	*H %*	*O %*	*S %*	*Repeat*	*Charge*
1-WC3-W2MePy-67-232	0.573	4.64	57.07	6.25				$ 320

Instrumentation: Elementar vario EL CUBE (CHN-OS Rapid, German), Accuracy: 0.1%, Precision: 0.2%

	Standard sample	*N %*	*C %*	*H %*	*O %*	*S %*
★	*Acetanilide*	*10.36*	*71.09*	*6.71*		
	Benzoic acid				*26.20*	
	Sulfanilic acid	*8.09*	*41.60*	*4.07*		*18.50*
	Daily standard	*10.39*	*71.12*	*6.73*		

Figure. *S*116 Elemental analysis report of **19**

National Chung Hsing University

The Office of Research and Development, Instrument Center

Report on Elemental Analysis

Instrument Director : Prof. Chen-Yu Yeh

Operator : I-Chuan Chen

Note: This result is for academic use only, not to be used for any judicial or commercial advertising purpose.

Information of Submitted Sample

Web NO :	BSEA0001002024070020		
Department :	National Tsing Hua University Department of chemistry		Date of
Supervisor :	Prof. Yi-Chou Tsai	Receive:	2024.07.18
User name :	Chen Wen-Chi	completion:	2024.07.18

Results of Analysis

Sample code	*Weight(mg)*	*N %*	*C %*	*H %*	*O %*	*S %*	*Repeat*	*Charge*
WC-9-WtBu	2.088	4.98	55.10	6.38				$ 320

Instrumentation: *Elementar vario EL CUBE (CHN-OS Rapid, German), Accuracy: 0.1%, Precision: 0.2%*

	Standard sample	*N %*	*C %*	*H %*	*O %*	*S %*
★	*Acetanilide*	*10.36*	*71.09*	*6.71*		
	Benzoic acid				*26.20*	
	Sulfanilic acid	*8.09*	*41.60*	*4.07*		*18.50*
	Daily standard	*10.36*	*71.11*	*6.76*		

Figure. *S*117 Elemental analysis report of **20**

National Chung Hsing University

The Office of Research and Development, Instrument Center

Report on Elemental Analysis

Instrument Director : Prof. Chen-Yu Yeh

Operator : I-Chuan Chen

Note: This result is for academic use only, not to be used for any judicial or commercial advertising purpose.

Information of Submitted Sample

Web NO :	BSEA0001002025080028		
Department :	National Tsing Hua University<br>Department of chemistry		Date of
Supervisor :	Prof. Yi-Chou Tsai	Receive:	2025.08.07
User name :	Wen-Chi Chen	completion:	2025.08.07

Results of Analysis

Sample code	*Weight(mg)*	*N %*	*C %*	*H %*	*O %*	*S %*	*Repeat*	*Charge*
WC-WSiMe3-149-1	1.257	4.49	54.28	6.77				$ 320

Instrumentation: Elementar vario EL CUBE (CHN-OS Rapid, German), Accuracy: 0.1%, Precision: 0.2%

	Standard sample	*N %*	*C %*	*H %*	*O %*	*S %*
★	*Acetanilide*	*10.36*	*71.09*	*6.71*		
	Benzoic acid				*26.20*	
	Sulfanilic acid	*8.09*	*41.60*	*4.07*		*18.50*
	Daily standard	*10.33*	*71.00*	*6.64*		

Figure. *S*118 Elemental analysis report of **21**

National Chung Hsing University

The Office of Research and Development, Instrument Center

Report on Elemental Analysis

Instrument Director : Prof. Chen-Yu Yeh

Operator : I-Chuan Chen

Note: This result is for academic use only, not to be used for any judicial or commercial advertising purpose.

Information of Submitted Sample

Web NO :	BSEA0001002023020044		
Department :	National Tsing Hua University<br>Department of chemistry		Date of
Supervisor :	Prof. Yi-Chou Tsai	Receive:	2023.02.23
User name :	Cian-Wei Yang	completion:	2023.02.23

Results of Analysis

Sample code	*Weight(mg)*	*N %*	*C %*	*H %*	*O %*	*S %*	*Repeat*	*Charge*
CW-230346LT	3.758	4.49	55.64	6.56				$ 320

Instrumentation: *Elementar vario EL CUBE (CHN-OS Rapid, German), Accuracy: 0.1%, Precision: 0.2%*

	Standard sample	*N%*	*C %*	*H %*	*O %*	*S %*
★	*Acetanilide*	*10.36*	*71.09*	*6.71*		
	Benzoic acid				*26.20*	
	Sulfanilic acid	*8.09*	*41.60*	*4.07*		*18.50*
	Daily standard	*10.37*	*71.17*	*6.79*		

Figure. *S*119 Elemental analysis report of **22**

National Chung Hsing University

The Office of Research and Development, Instrument Center

Report on Elemental Analysis

Instrument Director : Prof. Bao-Tsan Ko

Operator : I-Chuan Chen

Note: This result is for academic use only, not to be used for any judicial or commercial advertising purpose.

Information of Submitted Sample

Web NO :	BSEA0001002026010044		
Department :	National Tsing Hua University Department of chemistry		Date of
Supervisor :	Prof. Yi-Chou Tsai	Receive:	2026.02.26
User name :	Wen-Chi Chen	completion:	2026.02.26

Results of Analysis

Sample code	*Weight(mg)*	*N %*	*C %*	*H %*	*O %*	*S %*	*Repeat*	*Charge*
1-WC3-W2Me1-P-2+2+1-119	1.839	3.83	57.05	6.55				$ 320

Instrumentation: Elementar vario EL CUBE (CHN-OS Rapid, German), Accuracy: 0.1%, Precision: 0.2%

	Standard sample	*N %*	*C %*	*H %*	*O %*	*S %*
★	*Acetanilide*	*10.36*	*71.09*	*6.71*		
	Benzoic acid				*26.20*	
	Sulfanilic acid	*8.09*	*41.60*	*4.07*		*18.50*
	Daily standard	*10.39*	*71.01*	*6.79*		

Figure. *S*120 Elemental analysis report of **23**

National Chung Hsing University

The Office of Research and Development, Instrument Center

Report on Elemental Analysis

Instrument Director : Prof. Chen-Yu Yeh

Operator : I-Chuan Chen

Note: This result is for academic use only, not to be used for any judicial or commercial advertising purpose.

Information of Submitted Sample

Web NO :	BSEA0001002024100025		
Department :	National Tsing Hua University Department of chemistry		Date of
Supervisor :	Prof. Yi-Chou Tsai	Receive:	2024.10.17
User name :	Chen Wen-Chi	completion:	2024.10.17

Results of Analysis

Sample code	*Weight(mg)*	*N %*	*C %*	*H %*	*O %*	*S %*	*Repeat*	*Charge*
WC-WtBu+PhCCH-51	0.852	4.02	57.26	6.30				$ 320

Instrumentation: ***Elementar vario EL CUBE (CHN-OS Rapid, German), Accuracy: 0.1%, Precision: 0.2%***

	Standard sample	*N %*	*C %*	*H %*	*O %*	*S %*
★	*Acetanilide*	*10.36*	*71.09*	*6.71*		
	Benzoic acid				*26.20*	
	Sulfanilic acid	*8.09*	*41.60*	*4.07*		*18.50*
	Daily standard	*10.39*	*71.16*	*6.70*		

Figure. *S*121 Elemental analysis report of **24**

National Chung Hsing University

The Office of Research and Development, Instrument Center

Report on Elemental Analysis

Instrument Director : Prof. Chen-Yu Yeh

Operator : I-Chuan Chen

Note: This result is for academic use only, not to be used for any judicial or commercial advertising purpose.

Information of Submitted Sample

Web NO :	BSEA0001002025070039		
Department :	National Tsing Hua University		Date of
	Department of chemistry		
Supervisor :	Prof. Yi-Chou Tsai	Receive:	2025.07.24
User name :	Wen-Chi Chen	completion:	2025.07.24

Results of Analysis

Sample code	*Weight(mg)*	*N %*	*C %*	*H %*	*O %*	*S %*	*Repeat*	*Charge*
WC2-WtBu-1-P-143	1.362	4.04	56.49	7.11				$ 320

Instrumentation: Elementar vario EL CUBE (CHN-OS Rapid, German), Accuracy: 0.1%, Precision: 0.2%

	Standard sample	*N %*	*C %*	*H %*	*O %*	*S %*
★	*Acetanilide*	*10.36*	*71.09*	*6.71*		
	Benzoic acid				*26.20*	
	Sulfanilic acid	*8.09*	*41.60*	*4.07*		*18.50*
	Daily standard	*10.38*	*71.14*	*6.69*		

Figure. *S*122 Elemental analysis report of **25**

National Chung Hsing University

The Office of Research and Development, Instrument Center

Report on Elemental Analysis

Instrument Director : Prof. Chen-Yu Yeh

Operator : I-Chuan Chen

Note: This result is for academic use only, not to be used for any judicial or commercial advertising purpose.

Information of Submitted Sample

Web NO :	BSEA0001002025120017		
Department :	National Tsing Hua University<br>Department of chemistry	Date of	
Supervisor :	Prof. Yi-Chou Tsai	Receive:	2025.12.11
User name :	Wen-Chi Chen	completion:	2025.12.11

Results of Analysis

Sample code	*Weight(mg)*	*N %*	*C %*	*H %*	*O %*	*S %*	*Repeat*	*Charge*
1-WC2-WSiMe1-P-231	0.546	3.52	54.81	6.89				$ 320

Instrumentation: Elementar vario EL CUBE (CHN-OS Rapid, German), Accuracy: 0.1%, Precision: 0.2%

	Standard sample	*N %*	*C %*	*H %*	*O %*	*S %*
★	*Acetanilide*	*10.36*	*71.09*	*6.71*		
	Benzoic acid				*26.20*	
	Sulfanilic acid	*8.09*	*41.60*	*4.07*		*18.50*
	Daily standard	*10.36*	*71.09*	*6.64*		

Figure. *S*123 Elemental analysis report of **26**

National Chung Hsing University

The Office of Research and Development, Instrument Center

Report on Elemental Analysis

Instrument Director : Prof. Bao-Tsan Ko

Operator : I-Chuan Chen

Note: This result is for academic use only, not to be used for any judicial or commercial advertising purpose.

Information of Submitted Sample

Web NO :	BSEA0001002026040055		
Department :	National Tsing Hua University Department of chemistry	Date of	
Supervisor :	Prof. Yi-Chou Tsai	Receive:	2026.04.30
User name :	Wen-Chi Chen	completion:	2026.04.30

Results of Analysis

Sample code	*Weight(mg)*	*N %*	*C %*	*H %*	*O %*	*S %*	*Repeat*	*Charge*
2-WC3-WtBuPh-lere-E-45min-151	2.144	4.19	58.45	6.62				$ 320

Instrumentation: Elementar vario EL CUBE (CHN-OS Rapid, German), Accuracy: 0.1%, Precision: 0.2%

	Standard sample	*N %*	*C %*	*H %*	*O %*	*S %*
★	*Acetanilide*	*10.36*	*71.09*	*6.71*		
	Benzoic acid				*26.20*	
	Sulfanilic acid	*8.09*	*41.60*	*4.07*		*18.50*
	Daily standard	*10.39*	*71.13*	*6.65*		

Figure. *S*124 Elemental analysis report of **27**

National Chung Hsing University

The Office of Research and Development, Instrument Center

Report on Elemental Analysis

Instrument Director : Prof. Chen-Yu Yeh

Operator : I-Chuan Chen

Note: This result is for academic use only, not to be used for any judicial or commercial advertising purpose.

Information of Submitted Sample

Web NO :	BSEA0001002025120017		
Department :	National Tsing Hua University Department of chemistry		Date of
Supervisor :	Prof. Yi-Chou Tsai	Receive:	2025.12.11
User name :	Wen-Chi Chen	completion:	2025.12.11

Results of Analysis

Sample code	Weight(mg)	N %	C %	H %	O %	S %	Repeat	Charge
2-WC2-WtBuPh-P-2e-185	1.108	4.38	59.79	6.46				$ 320

Instrumentation: Elementar vario EL CUBE (CHN-OS Rapid, German), Accuracy: 0.1%, Precision: 0.2%

	Standard sample	N %	C %	H %	O %	S %
★	Acetanilide	10.36	71.09	6.71		
	Benzoic acid				26.20	
	Sulfanilic acid	8.09	41.60	4.07		18.50
	Daily standard	10.36	71.09	6.64		

Figure. *S*125 Elemental analysis report of **28**

7 Details of computational studies

7.1 Computational methods

Calculations were performed with the Gaussian 16 software package.[13] The molecular geometries were optimized without symmetry constraints at the (U)BP86 level of density functional theory (DFT),[14,15] and stability of wavefunction was checked for optimized structure. Vibrational frequency calculations at the same level of theory have also been performed at 1 atm and 298.15 K to identify all the located stationary points as minima (zero imaginary frequency) or transition states (one imaginary frequency). The SCF convergence criterion was set to 10^{-8} in all cases. Intrinsic reaction coordinates (IRC)[16,17] were calculated for the transition states to validate the reactants and products. The SDD ECP basis set was used to describe W atom[18,19] and a 6-31G(d,p) Pople basis set for H, C, N, ,O, Li and Cl atoms (named BS-I).[20–24] The Cartesian coordinates of the optimized geometries are listed in Table *S*23. Multiwfn 3.8 (dev) program[25,26] was used for the analyses of localized molecular orbitals (LMOs),[27] Mayer bond order (MBO),[28] multi-center bond order (MCBO)[29] and nucleus-independent chemical shift (NICS).[30] The LMOs were searched by Pipek-Mezey method based on Mulliken population. The MBOs are summarized in Table *S*22.

NICS were evaluated on a simplified model (**5-S**) of Complex **5** in which the diisopropylphenyl groups of the amidinate ligands were replaced by methyl groups, and the phenyl substituents on the C_4W_2 unit were replaced by hydrogen**.** To elucidate the influence of local σ-framework anisotropy within the C_4W_2 fragment and the metal–metal multiple bond anisotropy associated with the W–W bond, a hydrogenated reference (**5-H**) was constructed by adding one hydrogen atom to each carbon of the C_4 unit to enforce formal single bonds; for this reference, the C_4W_2 ring was constrained to be planar**.** All NICS were computed with the GIAO method at the BP86/BS-I level. For the C_4W_2 ring, the geometric centers served as the anchor points. Along the local ring normal, we placed ghost atoms (Bq) at 0.1 Å increments from −10.0 to +10.0 Å and recorded the shielding tensor at each position to generate the NICS line scan**.** The parent truncated model and the hydrogenated reference were processed identically, and their $NICS_{zz}$ profiles were compared (Figure *S*153).

7.2 Comparison of the experimental and calculated structural metrics

Table *S*11. Comparisons of the experimental and calculated core structural metrics of **1** (S = 0, doublet state). Bond lengths and bond angles are respectively taken in units of (Å) and in units of (°).

^{2}1

	Exp.	Cal.
W(1)–W(1')	2.2535(4)	2.3018
W(1)–Cl(1)	2.3679(11)	2.4081
W(1)–Cl(2)	2.3748(12)	2.4021
W(1)–N(1)	2.134(4)	2.1917
W(1)–N(2')	2.133(3)	2.1808
N(1)–C(1)	1.322(6)	1.3443
N(2)–C(1)	1.325(6)	1.3443
W(1)–W(1')–Cl(1')	98.89(3)	99.637
W(1)–W(1')–N(2)	90.81(10)	90.419
W(1)–W(1')–Cl(2')	112.40(3)	113.639
W(1')–W(1)–N(1)	90.30(10)	90.419
Cl(1)–W(1)–Cl(2)	148.71(4)	146.687
Cl(1)–W(1)–N(1)	92.93(11)	93.427
N(1)–W(1)–N(2')	171.98(15)	171.649
N(2)–W(1')–W(1)–N(2')	0.000	2.193

Table *S*12. Comparisons of the experimental and calculated core structural metrics of **2** (*S* = 0, singlet state). Bond lengths and bond angles are respectively taken in units of (Å) and in units of (°).

^{1}**2**

	Exp.	Cal.
W(1)–W(2)	2.2017(3)	2.2463
W(1)–Cl(1)	2.4876(11)	2.5791
W(1)–Cl(3)	2.4082(11)	2.4714
W(1)–N(1)	2.111(4)	2.1558
W(1)–N(3)	2.105(4)	2.1316
W(1)–Li(1)	3.169(10)	3.2103
W(2)–Cl(1)	2.4996(12)	2.5761
W(2)–Cl(2)	2.4117(14)	2.4716
W(2)–Li(1)	3.19(1)	3.2204
W(1)–W(2)–Cl(1)	63.52(3)	64.237
W(1)–W(2)–Cl(2)	114.21(4)	114.903
W(1)–Cl(1)–W(2)	53.40(2)	51.663
W(2)–W(1)–Cl(1)	64.08(3)	64.099
W(2)–W(1)–Cl(3)	115.95(3)	115.083
Cl(1)–W(1)–Cl(3)	179.93(5)	175.299
Cl(1)–W(2)–Cl(2)	177.32(4)	175.767
Cl(2)–Li(1)–Cl(3)	135.8(5)	140.231
N(1)–W(1)–N(3)	173.85(15)	168.729
N(1)–W(1)–W(2)–N(4)	6.116	12.600

Table *S*13. Comparisons of the experimental and calculated core structural metrics of **3** (*S* = 0, singlet state). Bond lengths and bond angles are respectively taken in units of (Å) and in units of (°).

^{1}**3**

	Exp.	Cal.
W(1)–W(2)	2.2393(2)	2.2846
W(1)–Cl(1)	2.4086(8)	2.4133
W(1)–Cl(2)	2.5373(8)	2.6031
W(1)–N(1)	2.442(3)	2.4903
W(1)–N(3)	2.171(3)	2.2021
W(1)–N(5)	2.147(6)	2.1681
W(2)–Cl(2)	2.5141(8)	2.6179
W(2)–N(2)	2.132(3)	2.1217
W(1)–W(2)–Cl(2)	64.22(2)	63.691
W(1)–W(2)–N(2)	117.53(8)	115.328
W(1)–Cl(2)–W(2)	52.628(17)	51.896
W(2)–W(1)–Cl(1)	109.05(2)	110.625
W(2)–W(1)–Cl(2)	63.15(2)	64.367
W(2)–W(1)–N(1)	160.43(7)	157.593
Cl(1)–W(1)–Cl(2)	172.11(3)	174.950
Cl(1)–W(1)–N(1)	90.51(7)	91.614
Cl(2)–W(1)–N(1)	97.31(7)	93.393
Cl(2)–W(2)–N(2)	178.18(5)	178.783
N(3)–W(1)–N(5)	168.81(11)	169.395
N(3)–W(1)–W(2)–N(6)	9.137	10.832

Table *S*14. Comparisons of the experimental and calculated core structural metrics of **4** (*S* = 0, singlet state). Bond lengths and bond angles are respectively taken in units of (Å) and in units of (°).

^{1}**4**

	Exp.	Cal.
W(1)–W(2)	2.3285(3)	2.3782
W(1)–O(1)	2.227(3)	2.3233
W(1)–N(1)	2.124(3)	2.1558
W(1)–N(3)	2.119(4)	2.1563
W(1)–H	1.56	1.7332
W(1)–HA	1.50	1.7528
W(2)–C(24)	2.183(5)	2.1936
W(2)–C(49)	2.168(5)	2.1865
W(2)–H	1.95	2.0944
W(2)–HA	2.12	2.0560
W(1)–W(2)–H	41.6	45.046
W(1)–W(2)–HA	38.9	45.856
W(1)–W(2)–C(24)	121.83(15)	120.400
W(1)–H–W(2)	82.3	76.178
W(1)–HA–W(2)	77.9	76.822
W(2)–W(1)–O(1)	139.08(9)	138.964
W(2)–C(24)–C(23)	116.6(3)	115.948
W(2)–C(49)–C(48)	116.5(4)	116.756
N(1)–W(1)–N(3)	179.88(17)	177.698
N(1)–W(1)–W(2)–N(4)	0.558	0.111

Table *S*15. Comparisons of the experimental and calculated core structural metrics of **5** (*S* = 0, singlet state). Bond lengths and bond angles are respectively taken in units of (Å) and in units of (°).

^{1}5

	Exp.	Cal.
W(3)–W(4)	2.2404(3)	2.2867
W(3)–C(117)	2.006(5)	2.0350
W(4)–C(120)	1.989(4)	2.0196
C(117)–C(118)	1.361(8)	1.4193
C(118)–C(119)	1.383(8)	1.3978
C(119)–C(120)	1.397(7)	1.4143
W(3)–W(4)–C(120)	99.87(14)	102.623
W(3)–C(117)–C(118)	131.9(5)	129.128
W(4)–W(3)–C(117)	102.58(17)	101.784
W(4)–C(120)–C(119)	132.7(4)	129.698
C(117)–C(118)–C(119)	125.6(5)	128.605
C(118)–C(119)–C(120)	127.2(5)	127.470
N(6)–W(3)–N(8)	152.72(15)	149.604
W(3)–W(4)–C(120)–C(119)	2.888	6.990
W(3)–C(117)–C(118)–C(119)	5.157	8.362
W(4)–W(3)–C(117)–C(118)	4.259	5.747
W(4)–C(120)–C(119)–C(118)	3.529	7.465
N(5)–W(4)–W(3)–N(6)	24.142	24.461
C(117)–W(3)–W(4)–C(120)	0.423	0.820
C(117)–C(118)–C(119)–C(120)	0.859	1.172

Table *S*16. Comparisons of the experimental and calculated core structural metrics of **12** (*S* = 0, singlet state). Bond lengths and bond angles are respectively taken in units of (Å) and in units of (°).

^{1}**12**		
	Exp.	Cal.
W(1)–W(2)	2.4281(4)	2.4970
W(1)–C(1)	1.892(6)	1.9162
W(1)–C(2)	2.273(6)	2.3089
W(2)–C(2)	2.269(6)	2.3170
W(2)–C(3)	2.297(6)	2.3200
W(2)–C(4)	2.049(8)	2.0692
C(1)–C(2)	1.504(8)	1.5129
C(2)–C(3)	1.497(8)	1.4681
C(3)–C(4)	1.344(10)	1.4014
W(1)–C(1)–C(2)	83.2(4)	83.766
W(1)–C(2)–W(2)	64.64(15)	65.344
W(1)–C(2)–C(1)	55.8(3)	55.589
W(1)–C(2)–C(3)	121.2(4)	119.743
W(2)–C(2)–C(1)	117.1(4)	118.182
W(2)–C(2)–C(3)	71.9(3)	71.654
W(2)–C(4)–C(3)	82.4(4)	81.442
N(1)–W(1)–N(3)	158.29(17)	154.664
C(1)–C(2)–C(3)	124.0(5)	123.443
C(2)–C(3)–C(4)	111.6(6)	111.973
N(1)–W(1)–W(2)–N(4)	11.804	14.090

Table *S*17. Comparisons of the experimental and calculated core structural metrics of **20** (*S* = 0, singlet state). Bond lengths and bond angles are respectively taken in units of (Å) and in units of (°).

^{1}20

	Exp.	Cal.
W(1)–W(2)	2.3311(2)	2.3803
W(1)–C(1)	2.163(4)	2.1902
W(1)–C(2)	2.316(4)	2.3909
W(2)–C(1)	1.911(4)	1.9292
C(1)–C(2)	1.430(6)	1.4186
C(2)–C(3)	1.469(6)	1.5001
W(1)–W(2)–C(1)	60.35(11)	60.009
W(1)–C(1)–W(2)	69.50(11)	70.67
W(1)–C(1)–C(2)	77.3(2)	79.869
W(1)–C(2)–C(1)	65.6(2)	64.391
W(1)–C(2)–C(3)	134.8(3)	133.693
W(2)–W(1)–C(1)	50.15(10)	49.724
W(2)–C(1)–C(2)	145.0(3)	148.619
N(1)–W(1)–N(3)	160.97(11)	162.052
C(1)–C(2)–C(3)	123.9(4)	123.582
W(1)–W(2)–C(1)–C(2)	19.396	18.740
W(1)–C(2)–C(1)–W(2)	18.591	17.890
W(2)–W(1)–C(2)–C(1)	8.623	7.477
C(1)–W(2)–W(1)–C(2)	6.756	5.718

Table *S*18. Comparisons of the experimental and calculated core structural metrics of **21** (S = 0, singlet state). Bond lengths and bond angles are respectively taken in units of (Å) and in units of (°).

^{1}21

	Exp.	Cal.
W(1)−W(2)	2.3607(16)	2.4086
W(1)−C(6)	2.07(4)	2.169
W(1)−C(7)	2.333(17)	2.344
W(1)−N(2)	2.147(3)	2.173
W(1)−N(4)	2.134(6)	2.171
W(2)−N(1)	2.296(6)	2.347
W(2)−N(3)	2.173(6)	2.239
W(2)−N(5)	2.153(6)	2.179
W(2)−C(6)	1.85(3)	1.937
C(6)−C(7)	1.47(3)	1.425
W(1)−W(2)−C(6)	57.4(12)	58.7
W(1)−W(2)−N(1)	151.33(15)	149.96
W(1)−C(6)−C(7)	80.5(17)	78.42
W(1)−C(7)−C(6)	61.1(16)	65.03
W(2)−W(1)−C(6)	48.7(7)	49.73
W(2)−W(1)−C(7)	85.9 (4)	85.92
W(2)−W(1)−C(7)−C(6)	12.9(14)	7.22
C(6)−W(2)−W(1)−C(7)	10.7(13)	5.64

Table *S*19. Comparisons of the experimental and calculated core structural metrics of **24** (*S* = 0, singlet state). Bond lengths and bond angles are respectively taken in units of (Å) and in units of (°).

^{1}24

	Exp.	Cal.
W(1)−W(2)	2.3252(6)	2.3780
W(1)−C(10)	3.170(4)	3.175
W(1)−C(1)	2.089(3)	2.086
W(1)−C(2)	2.514(4)	2.498
W(2)−C(3)	3.136(4)	3.183
W(2)−C(2)	2.389(4)	2.406
W(2)−C(3)	1.944(3)	1.982
C(10)−C(1)	1.348(5)	1.360
C(1)−C(2)	1.446(5)	1.467
C(2)−C(3)	1.452(5)	1.464
W(1)−W(2)−C(3)	98.56(11)	97.58
W(1)−C(2)−W(2)	56.54(8)	57.98
W(2)−W(1)−C(1)	90.38(10)	90.73
W(1)−C(1)−C(10)	133.4(3)	133.21
W(1)−C(1)−C(2)	88.7(2)	87.52
W(2)−C(3)−C(2)	88.2(2)	87.17
C(10)−C(1)−C(2)	136.0(3)	137.97
C(1)−C(2)−C(3)	130.7(3)	131.34
C(1)−W(1)−W(2)−C(4)	0.92(13)	3.20
C(1)−C(2)−C(3)−C(4)	112.0(5)	105.40

Table *S*20. Comparisons of the experimental and calculated core structural metrics of **27** (S = 1/2, doublet state). Bond lengths and bond angles are respectively taken in units of (Å) and in units of (°).

^{2}27

	Exp.	Cal.
W(1)−W(2)	2.2387(4)	2.2620
W(1)−C(3)	2.136(3)	2.1414
W(1)−Cl(1)	2.4357(7)	2.4983
W(1)−N(1)	2.1761(19)	2.2377
W(1)−N(3)	2.1897(19)	2.1841
W(2)−C(1)	2.107(3)	2.1381
W(2)−N(2)	2.0400(19)	2.1311
W(2)−N(4)	2.091(2)	2.0884
C(1)−C(2)	1.462(4)	1.4628
C(1)−C(10)	1.355(3)	1.3795
C(2)−C(3)	1.366(3)	1.3898
C(3)−C(4)	1.490(3)	1.4861
W(1)−W(2)−C(1)	90.97(7)	90.716
W(2)−W(1)−C(3)	94.67(6)	95.008
W(1)−C(1)−C(2)	118.24(17)	117.706
C(1)−C(2)−C(3)	120.0(2)	120.810
W(1)−C(3)−C(2)	115.87(18)	115.402
W(2)−W(1)−Cl(1)	106.28(2)	99.231
C(1)−W(2)−W(1)−C(3)	2.2(1)	4.210
Cl(1)−W(2)−W(1)−C(1)	178.52(7)	176.590

Table *S*21. Comparisons of the experimental and calculated core structural metrics of **28** (*S* = 0, singlet state). Bond lengths and bond angles are respectively taken in units of (Å) and in units of (°).

[1]28

	Exp.	Cal.
W(1)−W(2)	2.3495(3)	2.3985
W(1)−C(1)	2.452(4)	2.5549
W(1)−C(2)	2.105(4)	2.1385
W(2)−C(4)	2.099(4)	2.1094
W(2)−C(35)	2.151(4)	2.1673
C(1)−C(2)	1.334(5)	1.3645
C(2)−C(3)	1.436(5)	1.4362
C(3)−C(4)	1.372(6)	1.3959
W(1)−W(2)−C(4)	109.57(11)	108.019
W(1)−W(2)−C(35)	125.82(9)	126.403
W(2)−W(1)−C(1)	104.18(8)	104.800
W(2)−W(1)−C(2)	71.25(11)	72.630
C(1)−W(1)−C(2)	32.94(13)	32.276
C(1)−C(2)−C(3)	132.3(4)	131.730
C(2)−C(3)−C(4)	115.7(4)	117.046
C(4)−W(2)−C(35)	123.86(13)	125.062
C(1)−W(1)−W(2)−C(4)	2.10(13)	5.739
C(1)−W(1)−W(2)−C(35)	172.45(15)	177.800
C(1)−C(2)−C(3)−C(4)	179.5(4)	175.820
C(2)−W(1)−W(2)−C(4)	1.40(14)	3.093

Table *S*22. W−W bond lengths (Å, experimental and calculated) and Mayer bond orders (MBO) for selected complexes

	W−W (exp.)	W−W (cal.)	MBO
^{2}**1**	2.2535(4)	2.3018	2.89
^{1}**2**	2.2017(3)	2.2463	3.02
^{1}**3**	2.2393(2)	2.2846	2.82
^{1}**4**	2.3285(3)	2.3782	2.12
^{1}**5**	2.2404(3)	2.2867	2.79
^{1}**12**	2.4281(4)	2.4970	1.60
^{1}**20**	2.3311(2)	2.3803	2.13
^{1}**21**	2.3607(16)	2.4086	1.95
^{1}**22**	2.3476(5)	2.41034	1.93
^{1}**24**	2.3252(6)	2.3780	2.10
^{2}**27**	2.2387(4)	2.2620	2.79
^{1}**28**	2.3495(3)	2.3985	2.08

7.3 Thermodynamic parameters and energy profiles

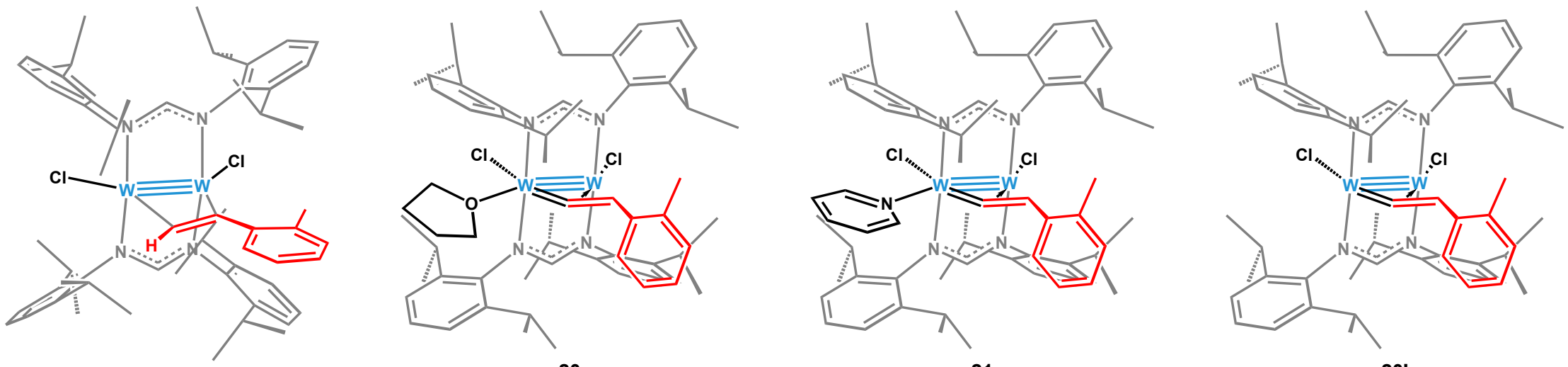

17 20 21 20'

Compound	**17**	**20**	**21**	**20'**
Relative electronic energy (ΔE)	0.00	−5.76	−6.66	2.38
Relative enthalpy (ΔH)	0.00	−2.65	−4.72	2.89

Figure. *S*126. Calculated relative electronic energies (ΔE) and enthalpy (ΔH) for complexes **17**, **20**, **21** and **20'** in kcal·mol^{-1}.

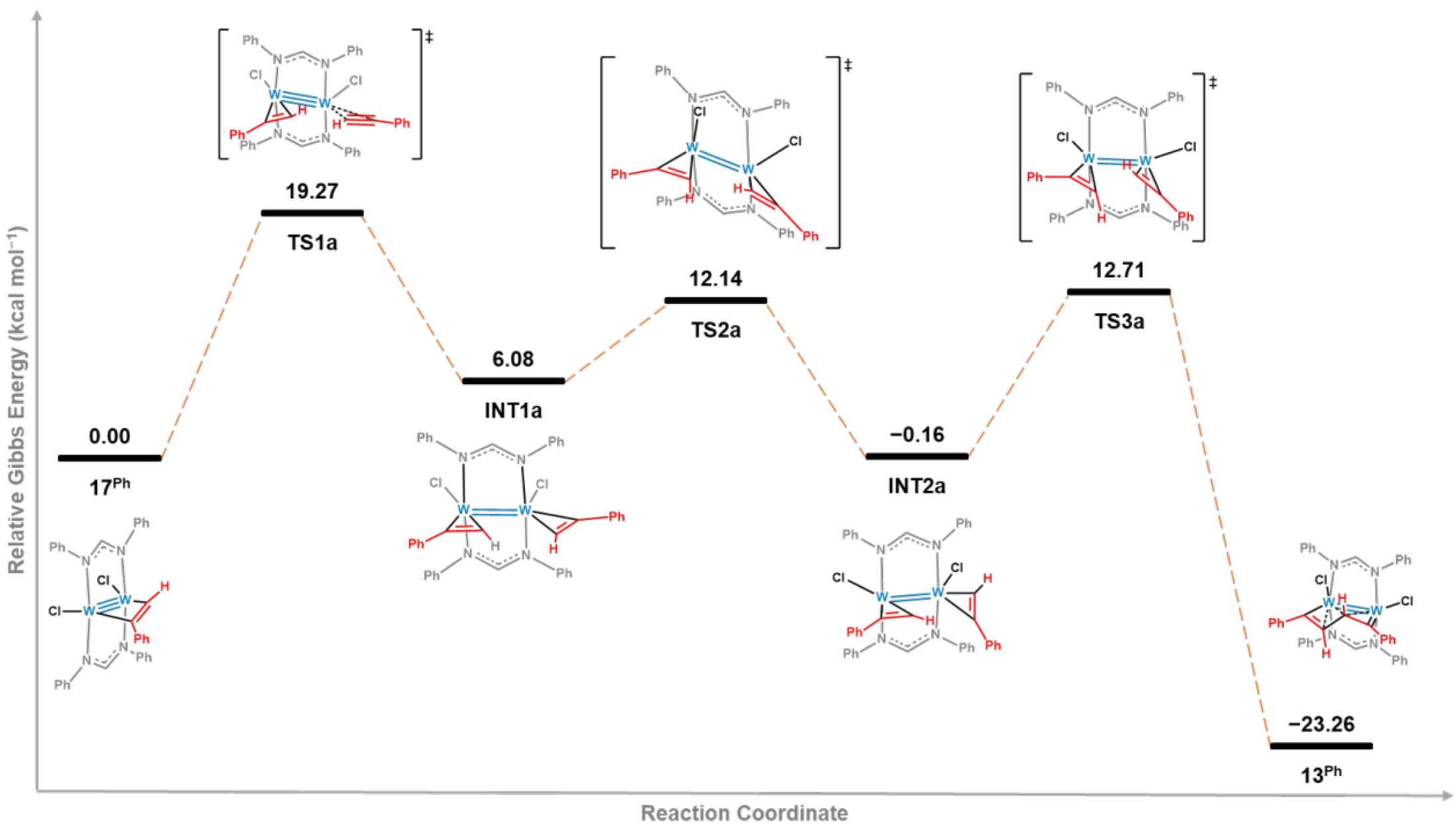

Figure. *S*127. Energy profile for the [2+2+2] cycloaddition pathway from intermediate $\mathbf{17^{Ph}}$ to $\mathbf{13^{Ph}}$. Relative Gibbs free energies (ΔG) are given in kcal·mol^{-1}

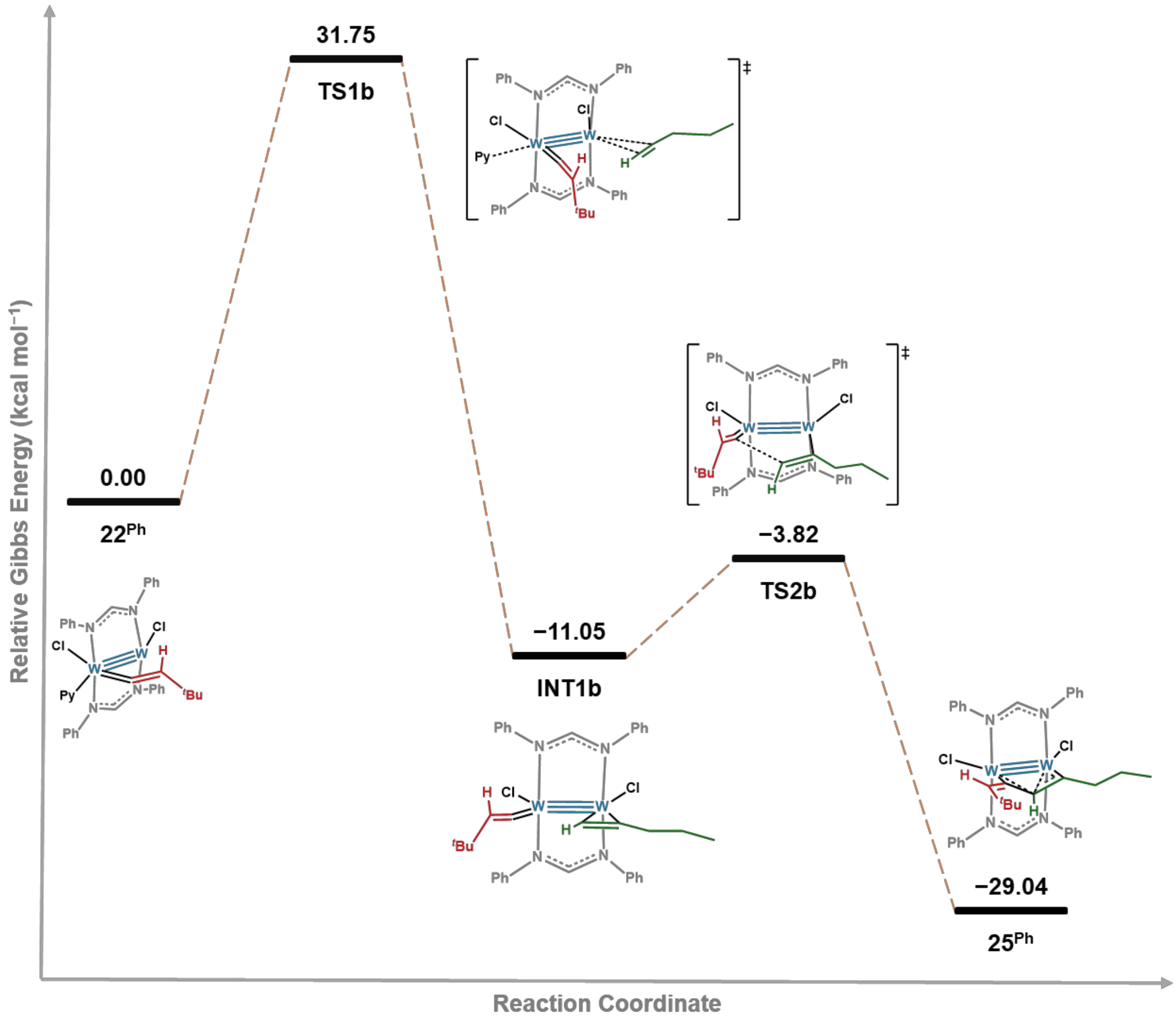

Figure. *S*128. Energy profile for the [2+2+1] cycloaddition pathway from intermediate **22**$^{\mathbf{Ph}}$ to **25**$^{\mathbf{Ph}}$ (R = Ph). Relative Gibbs free energies (ΔG) are given in kcal·mol^{-1}

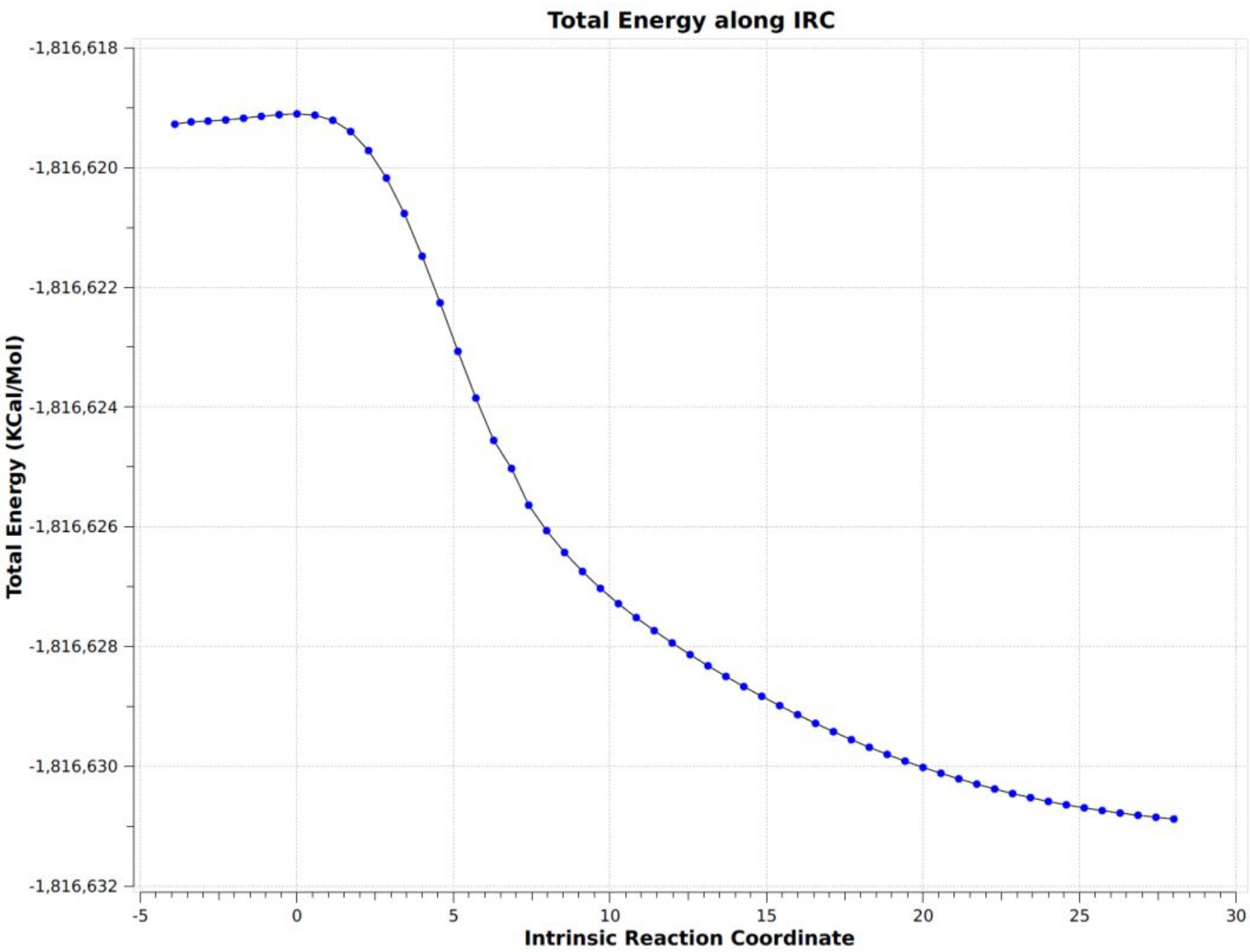

Figure. *S*129. IRC plot of transition state **TS1a**. Total energies are given in kcal·mol^{-1}

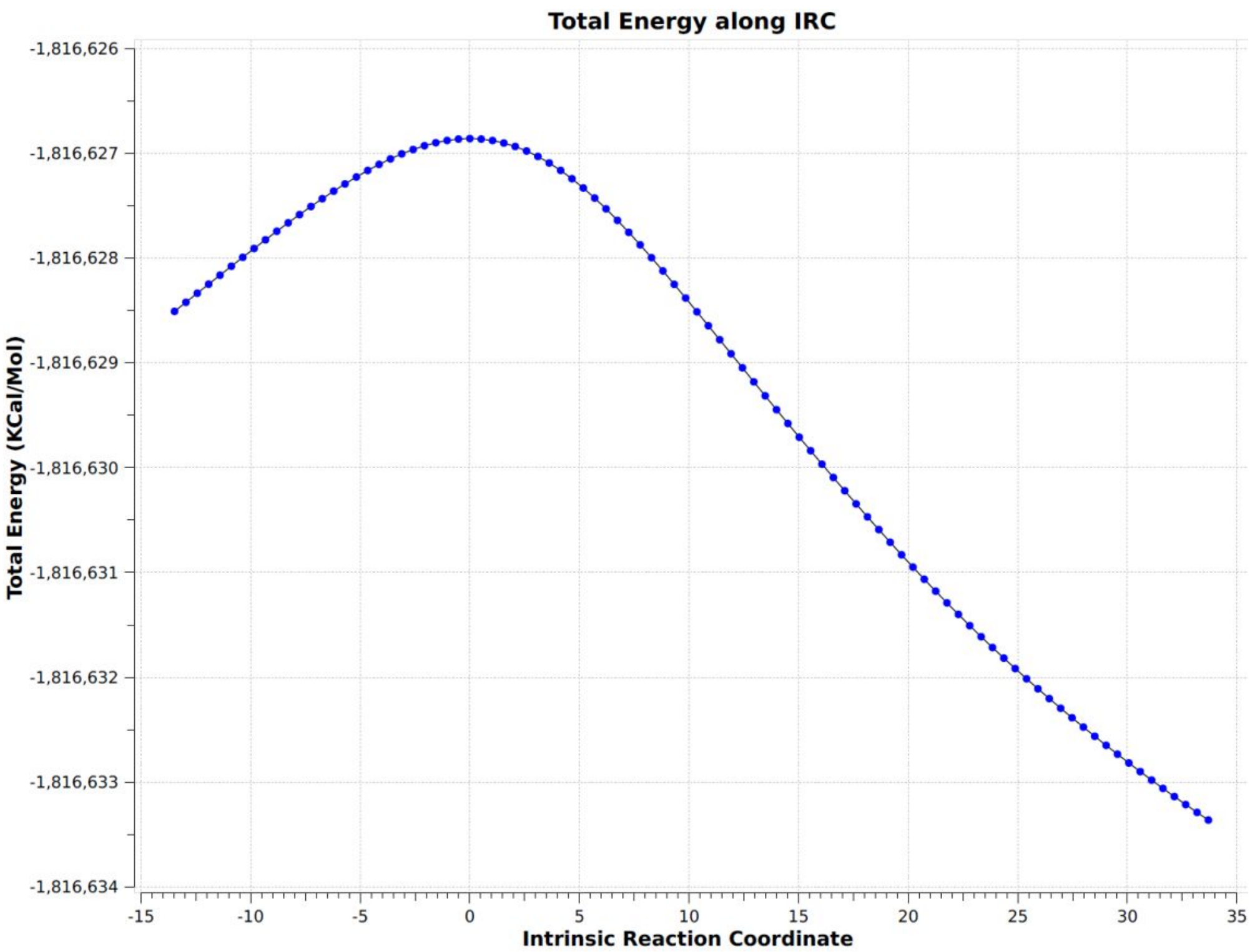

Figure. *S*130. IRC plot of transition state **TS2a**. Total energies are given in kcal·mol^{-1}

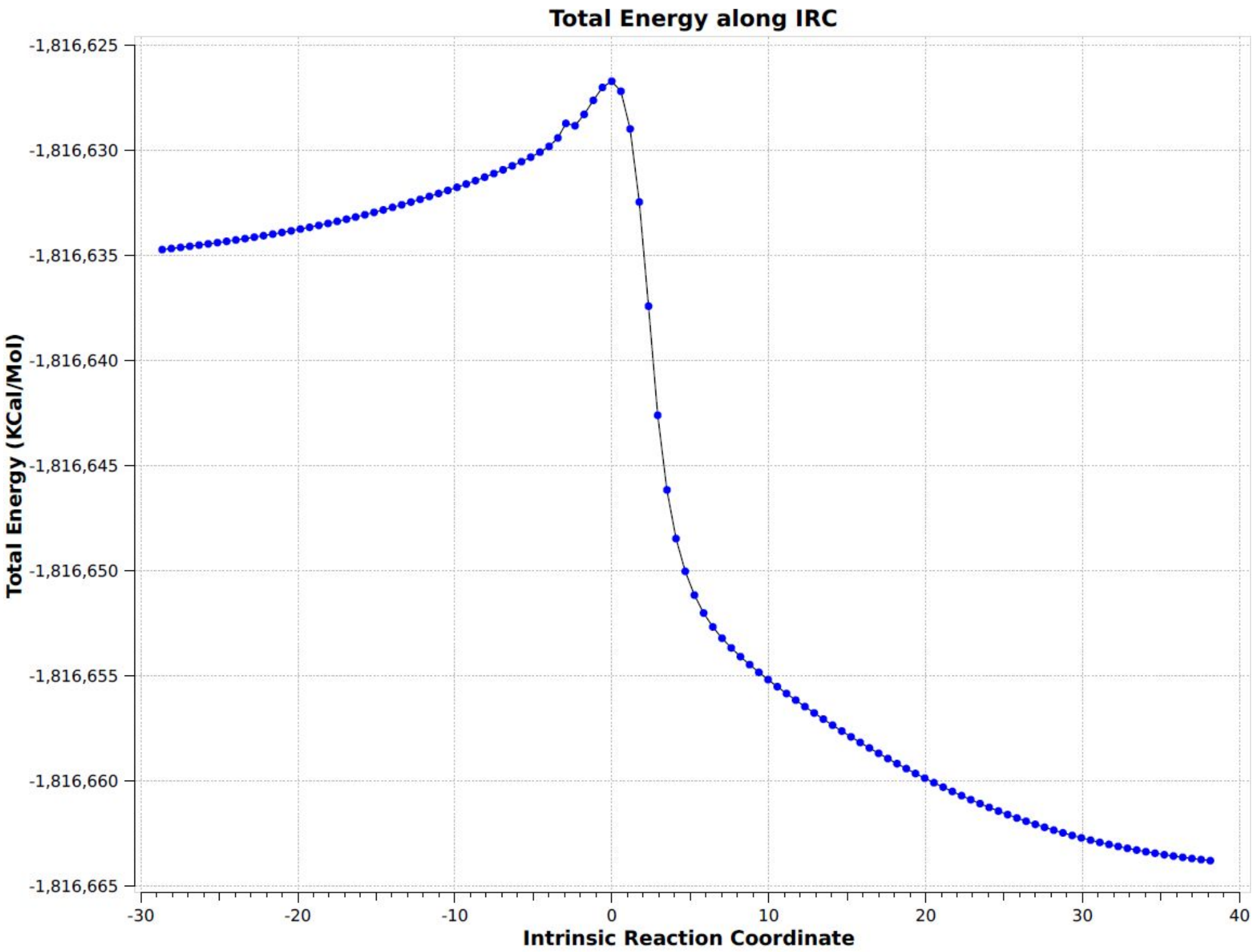

Figure. *S*131. IRC plot of transition state **TS3a**. Total energies are given in kcal·mol^{-1}

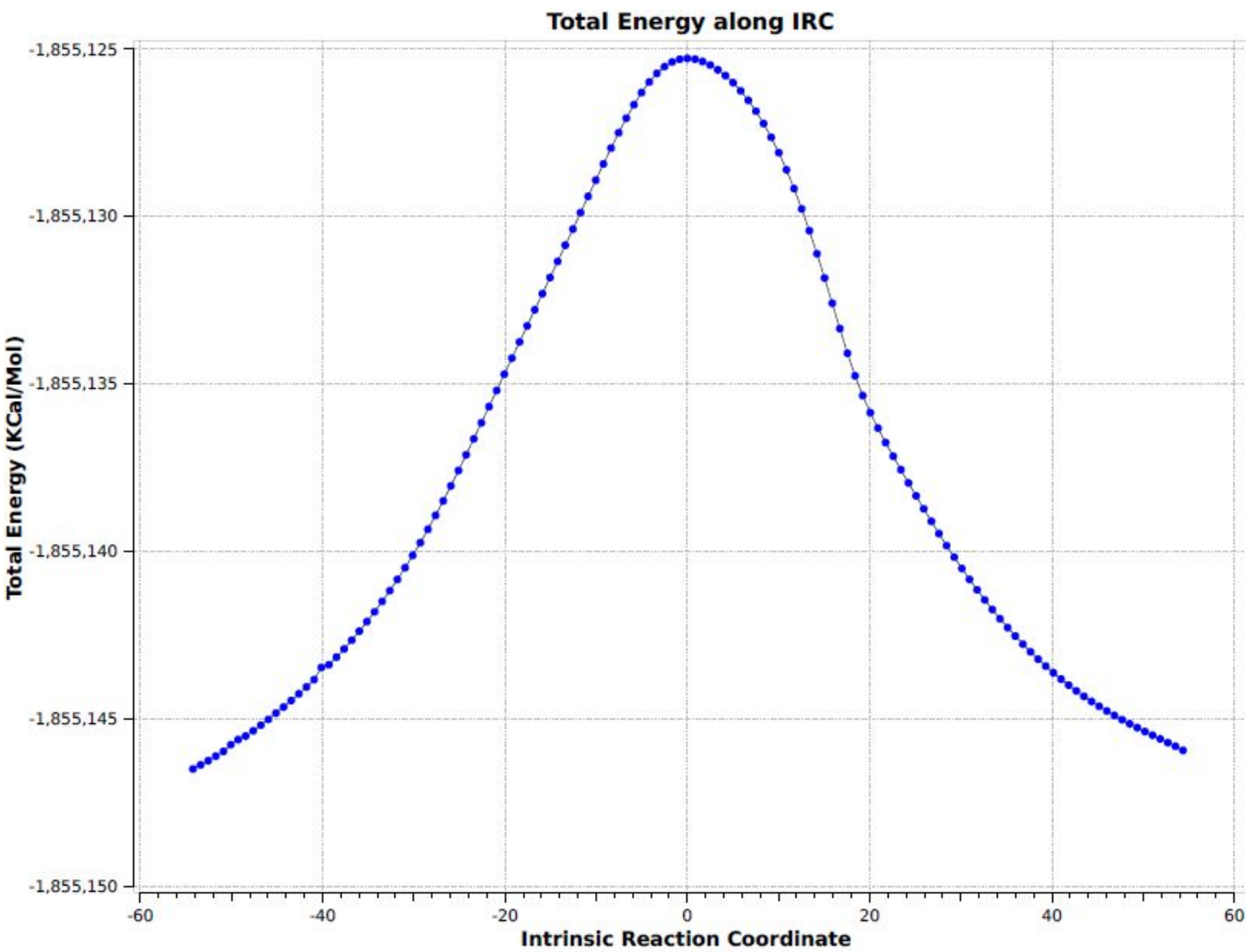

Figure. *S*132. IRC plot of transition state **TS1b**. Total energies are given in kcal·mol^{-1}

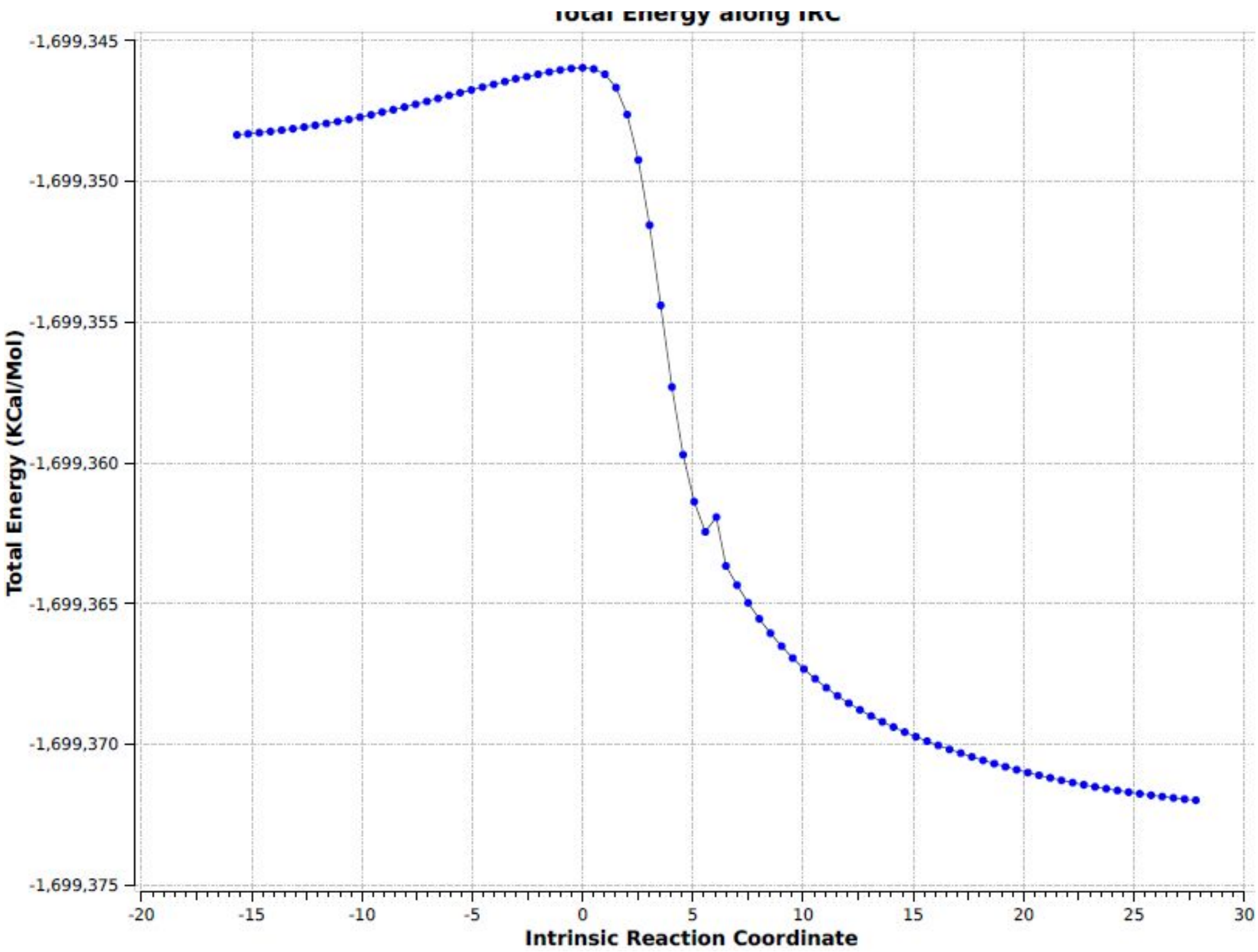

Figure. *S*133. IRC plot of transition state **TS2b**. Total energies are given in kcal·mol^{-1}

7.4 Canonical molecular orbital analysis

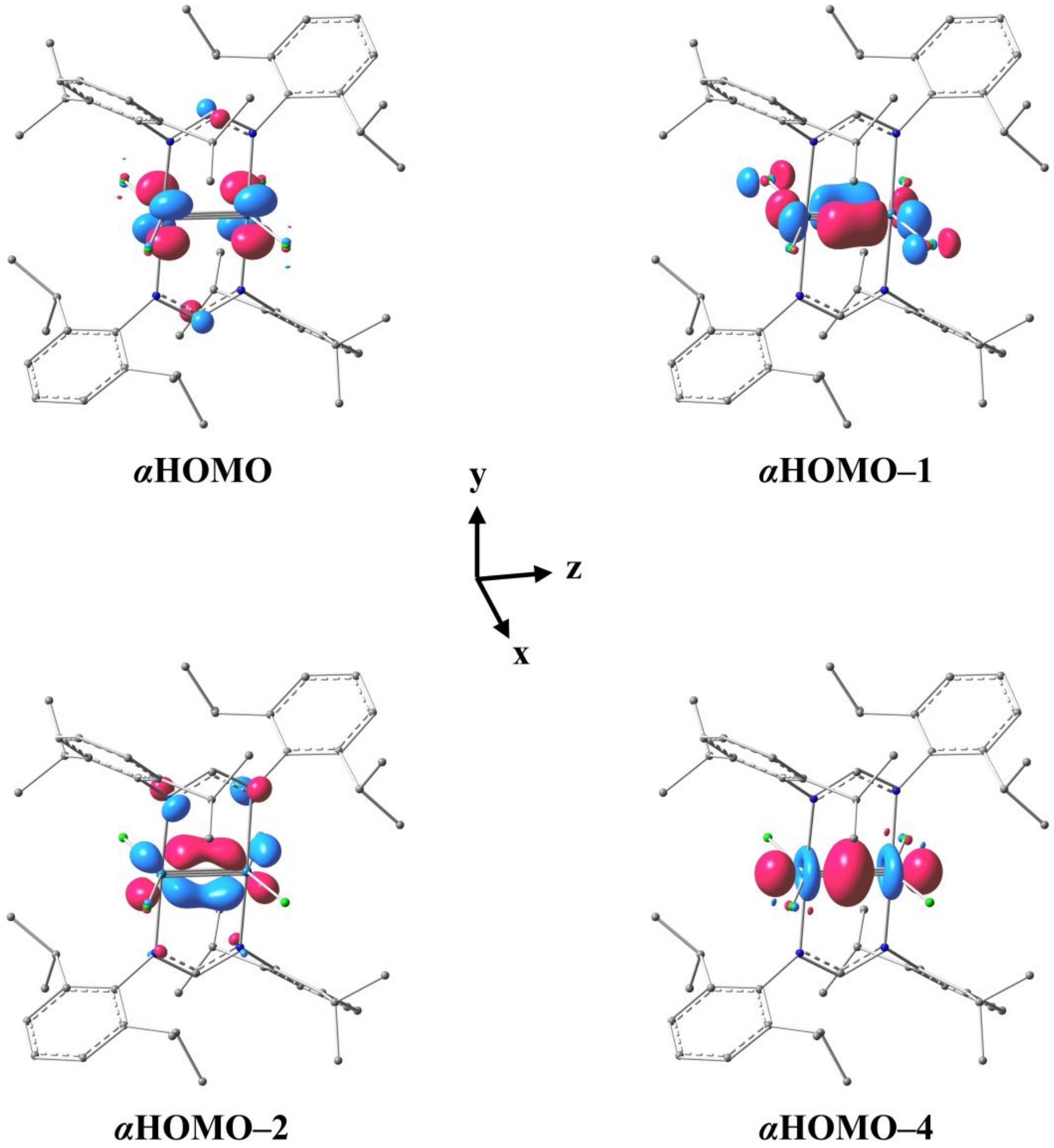

Figure. *S*134. The first half FMOs of **1** plotted with an isosurface of 0.08 a.u.

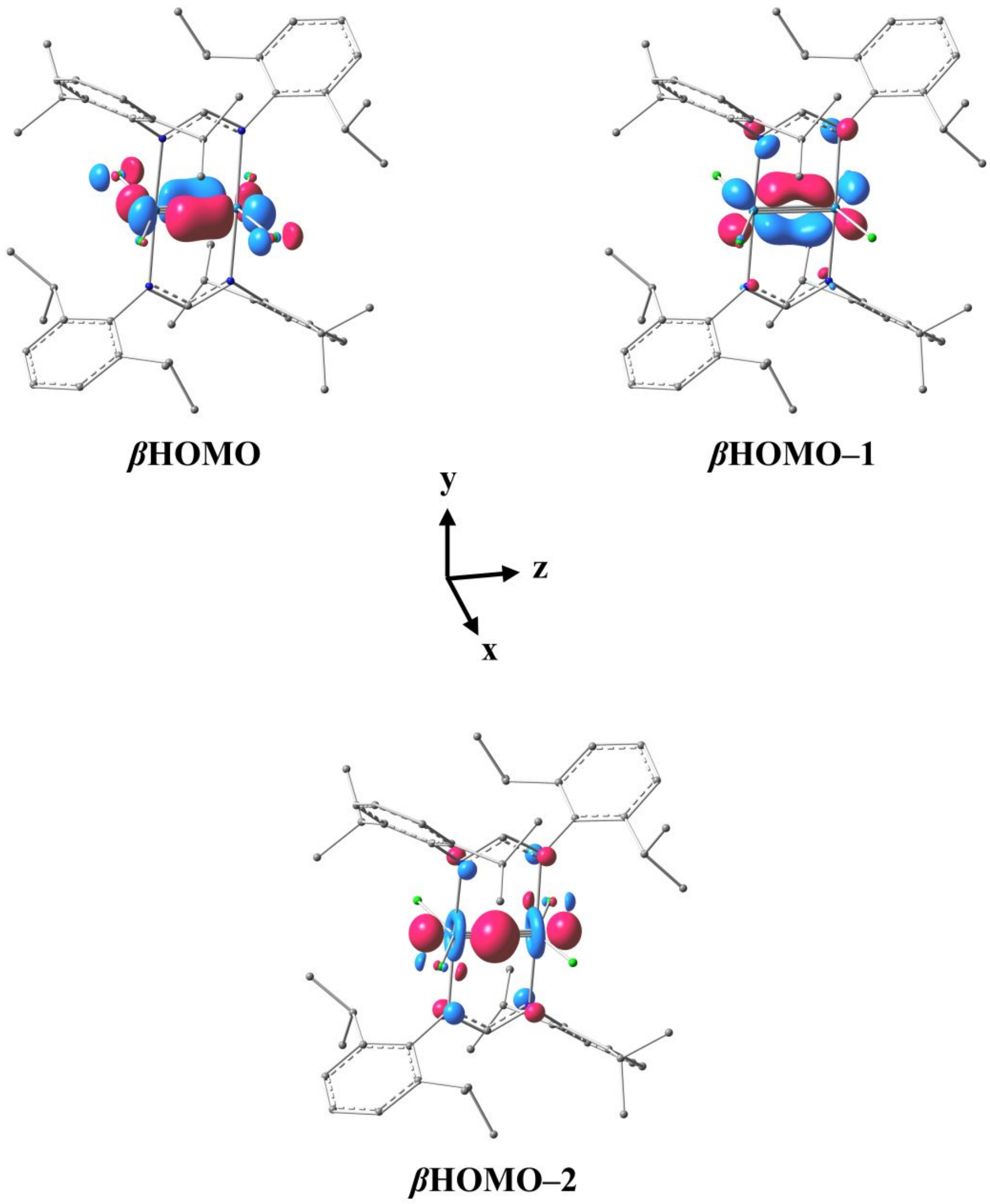

Figure. *S*135. The second half FMOs of **1** plotted with an isosurface of 0.08 a.u.

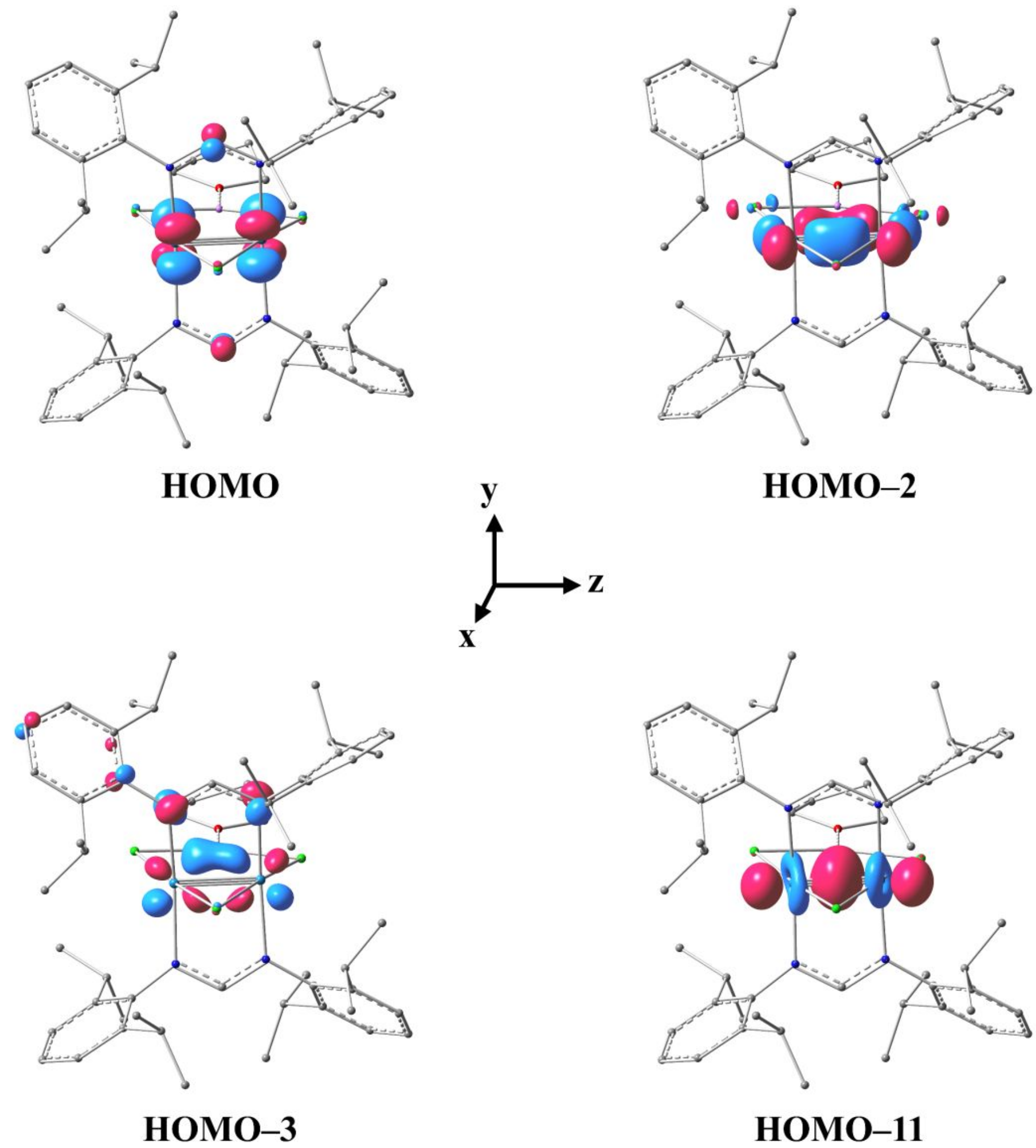

Figure. *S*136. The FMOs of **2** plotted with an isosurface of 0.08 a.u.

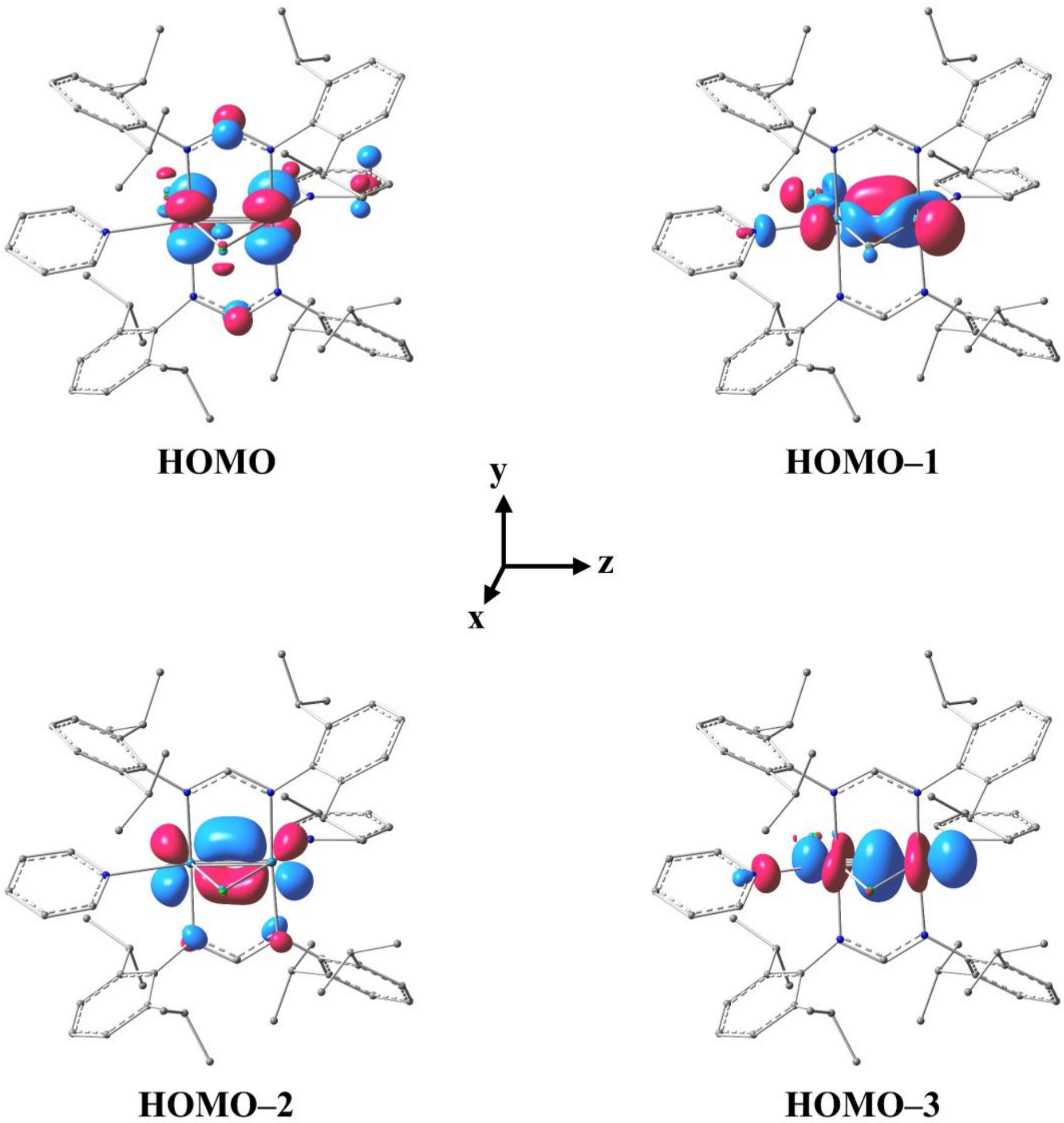

Figure. *S*137. The FMOs of **3** plotted with an isosurface of 0.06 a.u.

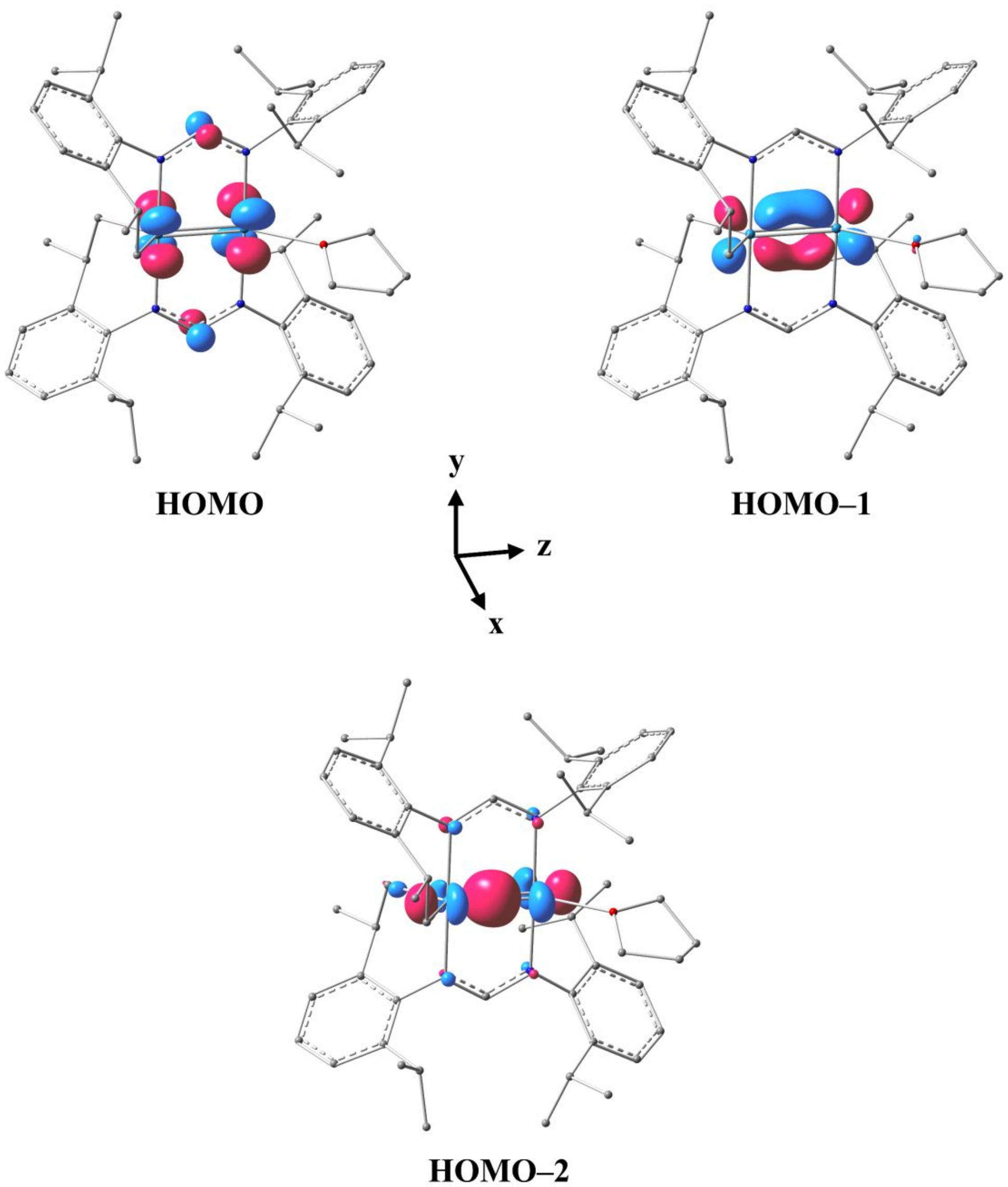

Figure. *S*138. The FMOs of **4** plotted with an isosurface of 0.08 a.u.

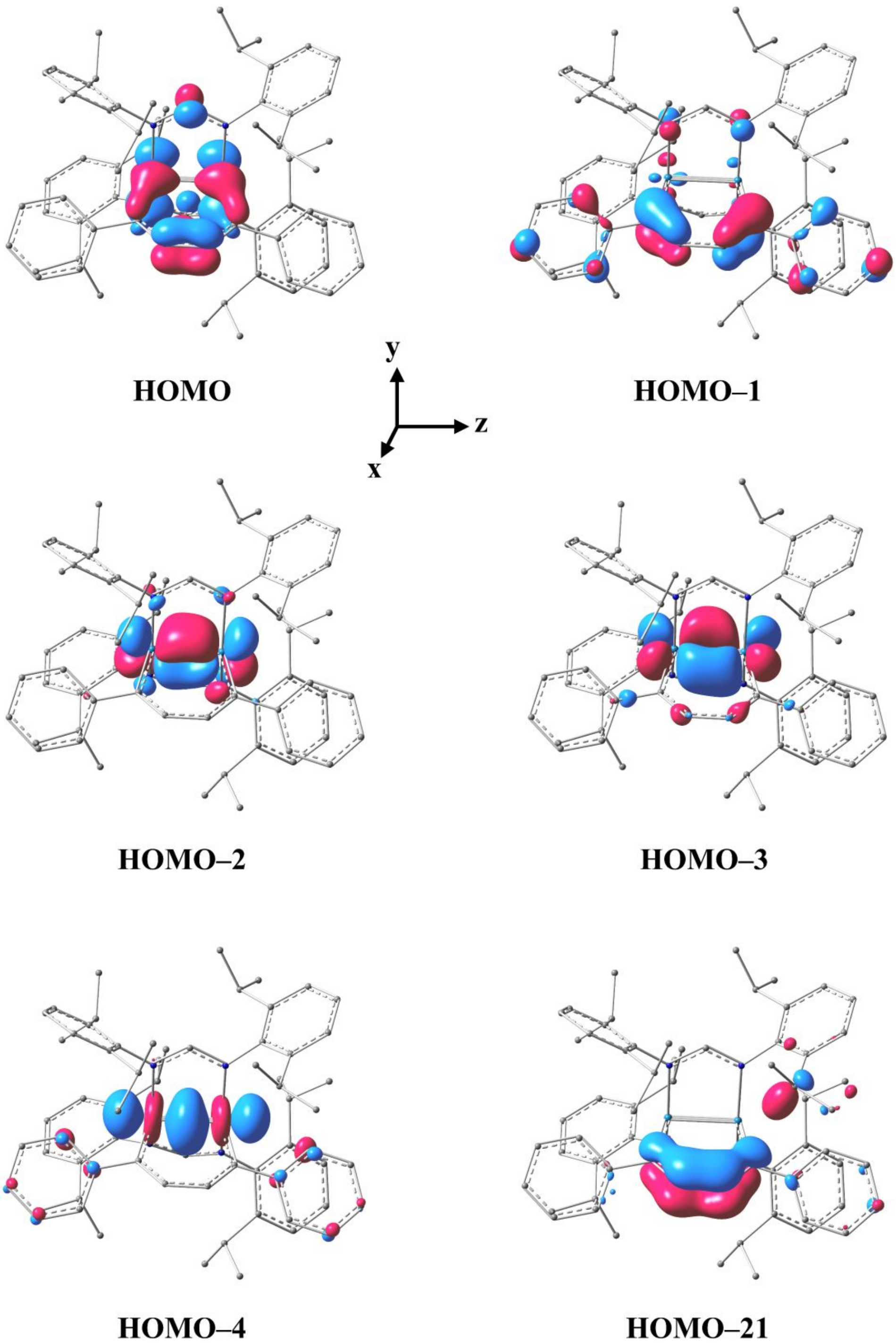

Figure. *S*139. The FMOs of **5** plotted with an isosurface of 0.05 a.u.

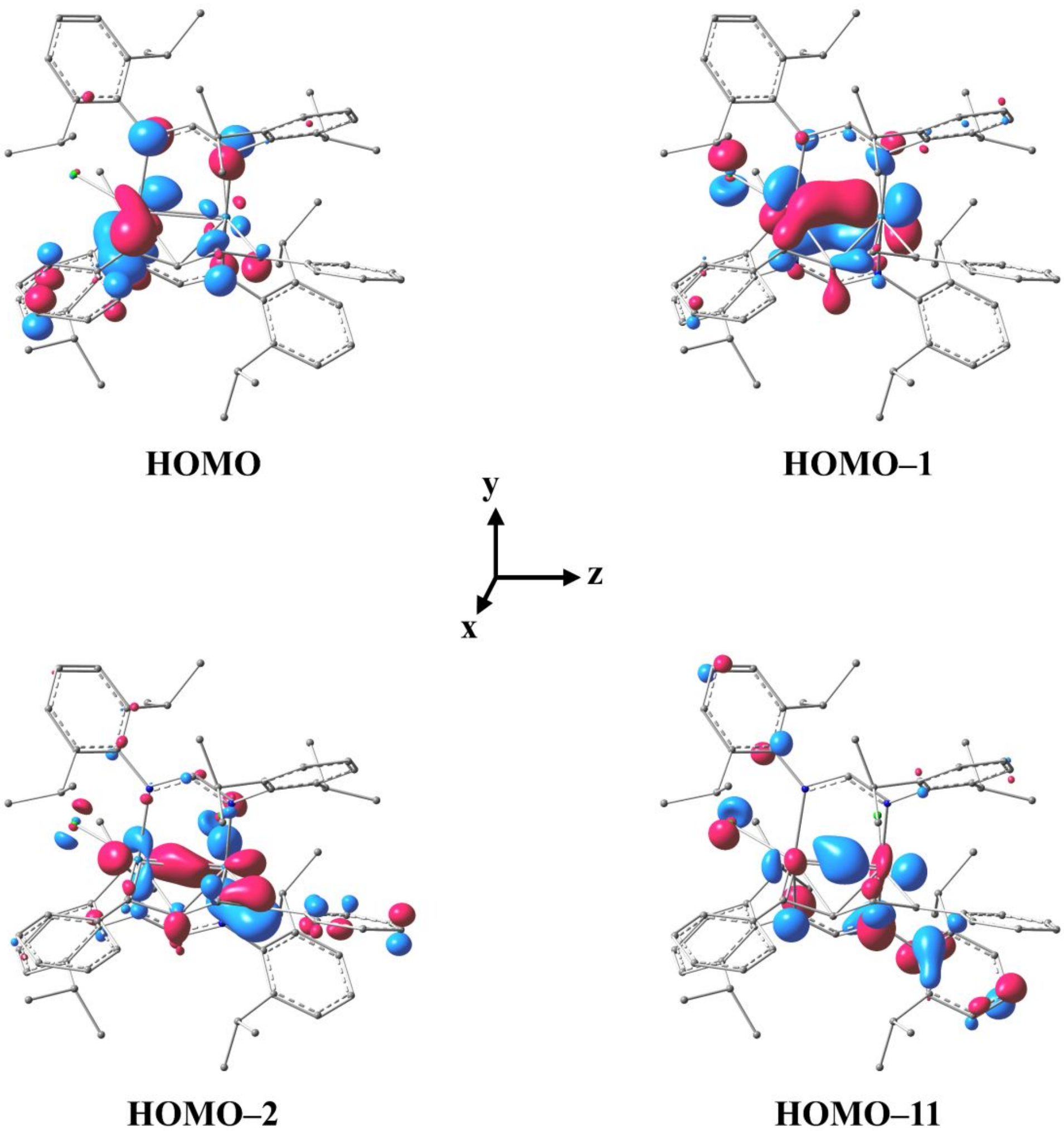

Figure. *S*140. The FMOs of **12** plotted with an isosurface of 0.05 a.u.

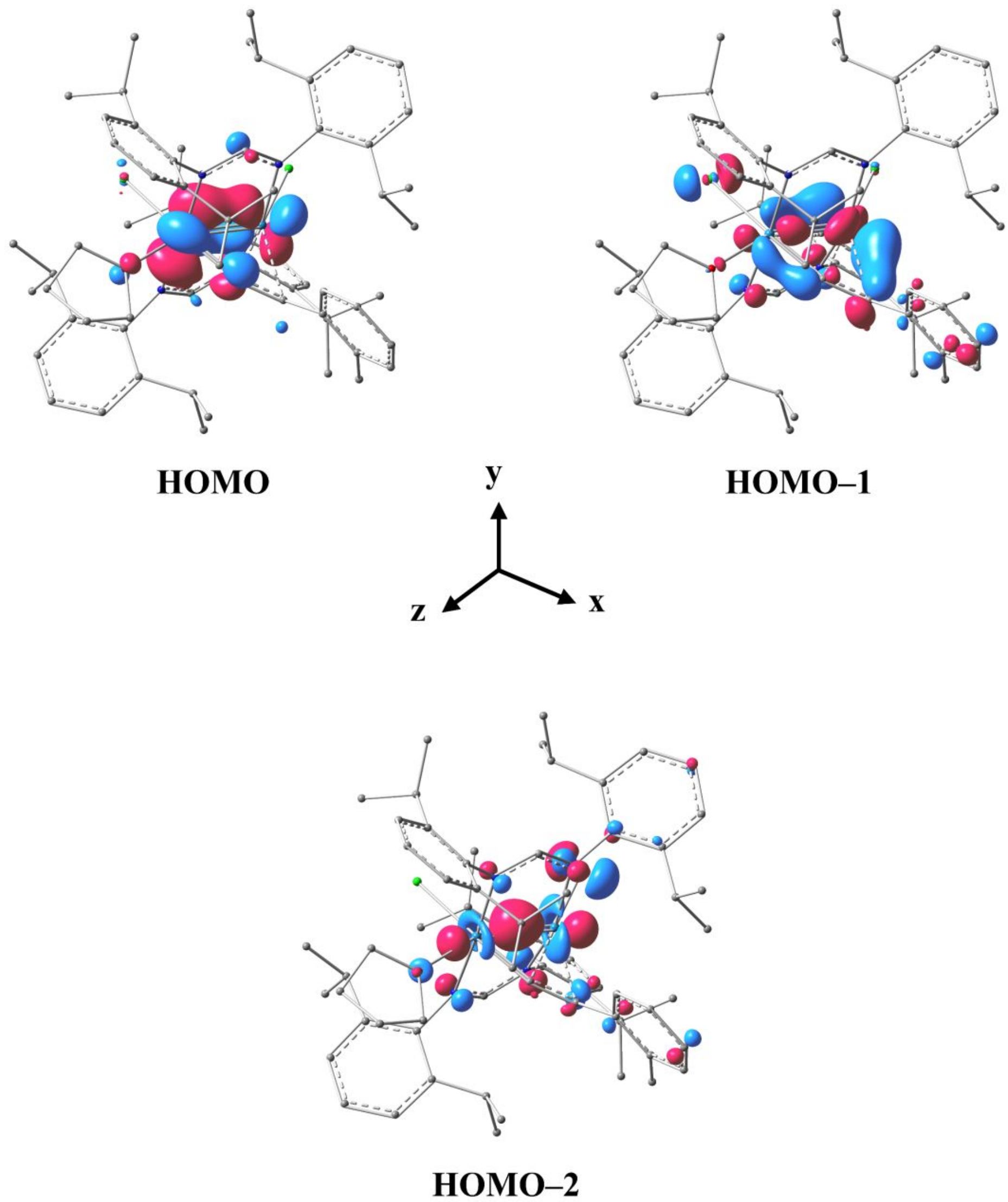

Figure. *S*141. The FMOs of **20** plotted with an isosurface of 0.06 a.u.

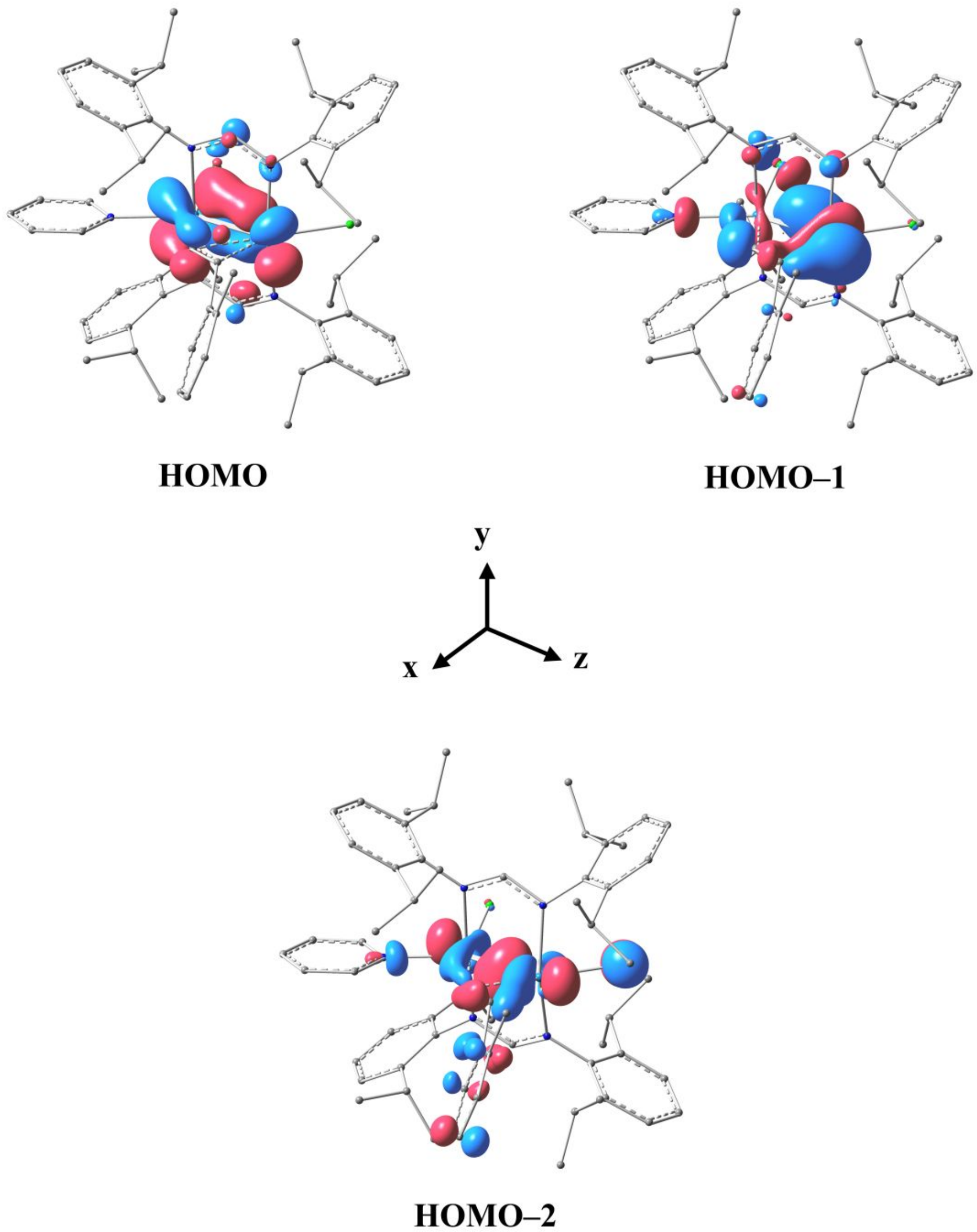

Figure. *S*142. The FMOs of **21** plotted with an isosurface of 0.05 a.u.

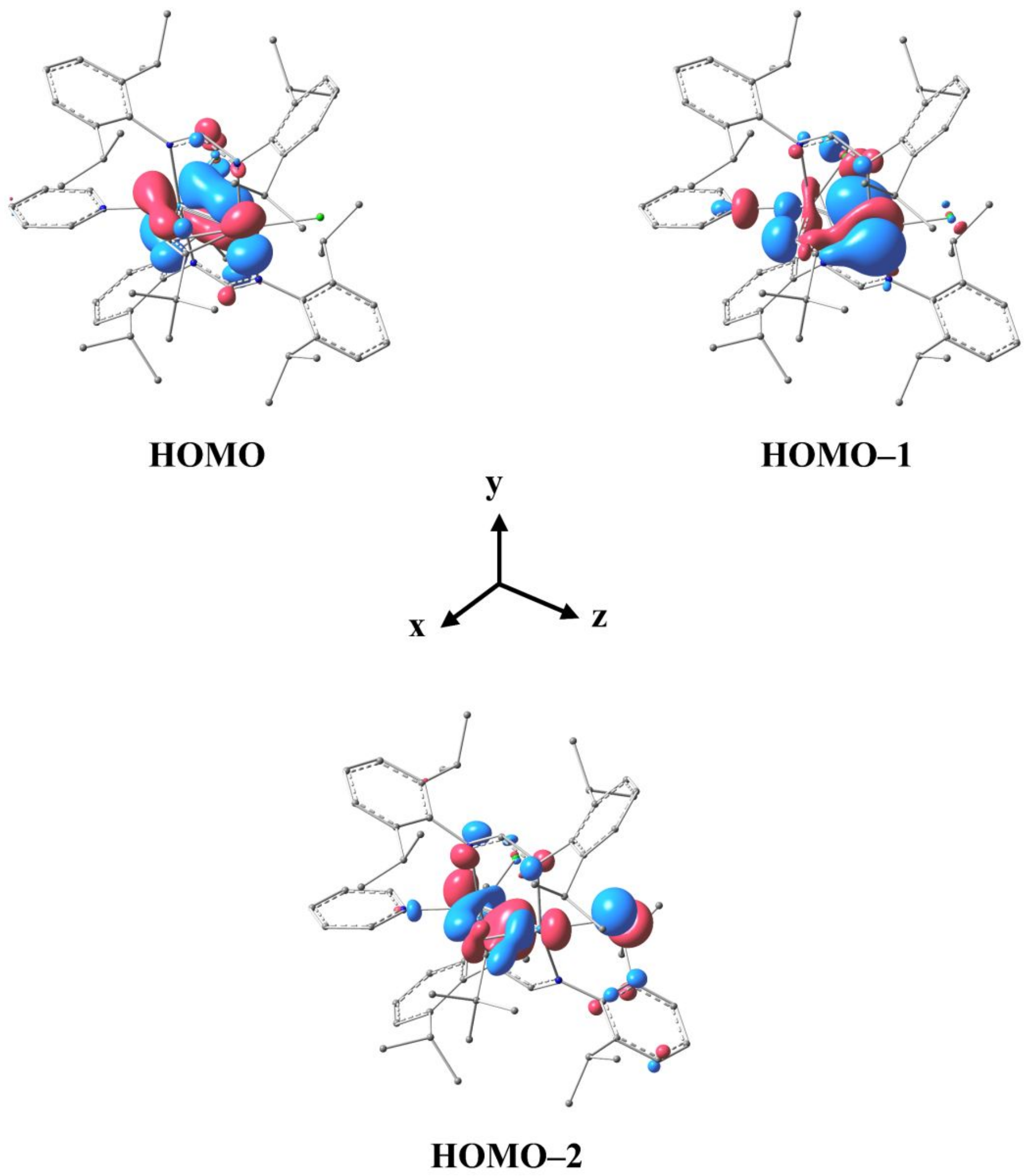

Figure. *S*143. The FMOs of **22** plotted with an isosurface of 0.05 a.u.

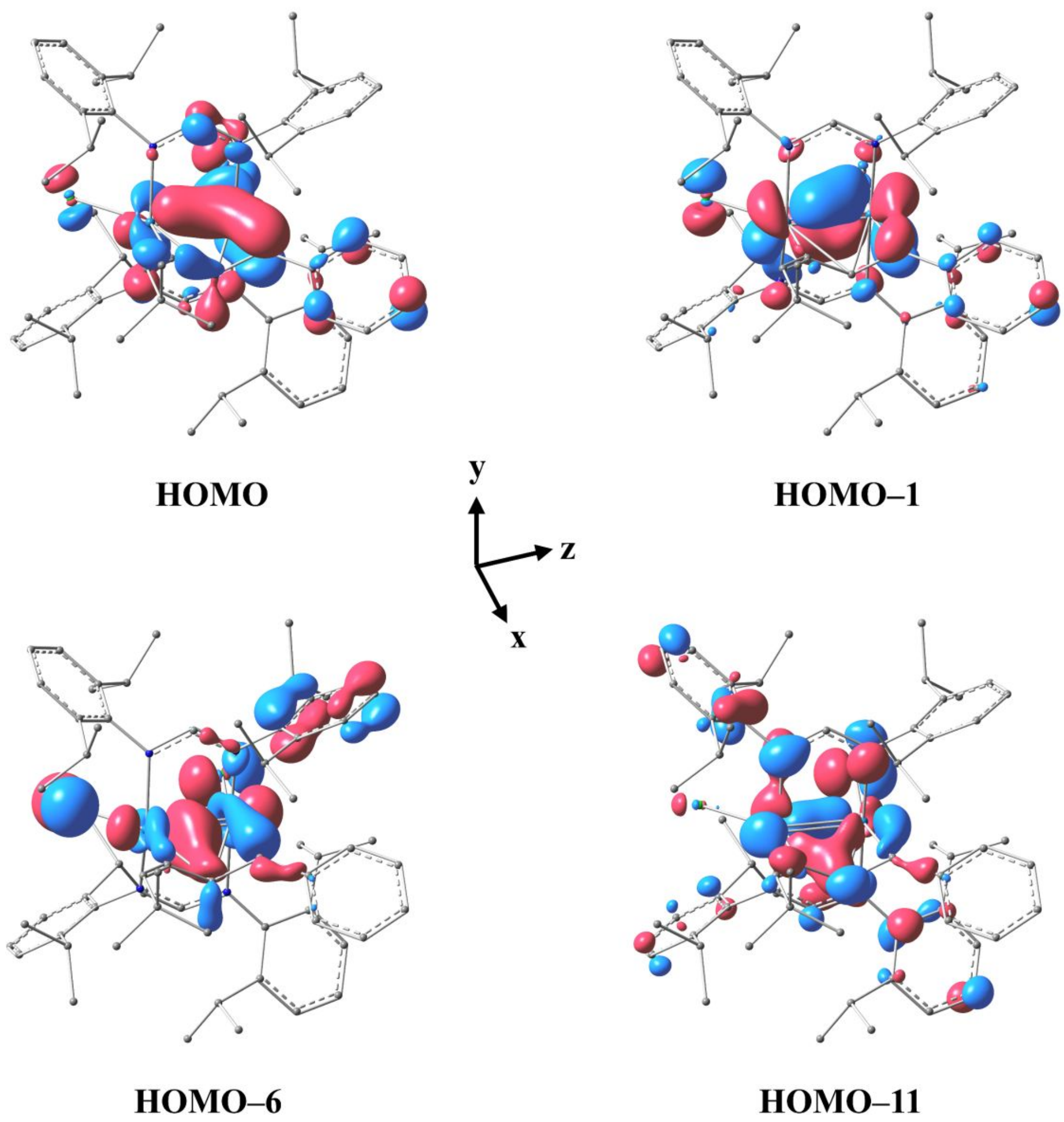

Figure. *S*144. The FMOs of **24** plotted with an isosurface of 0.04 a.u.

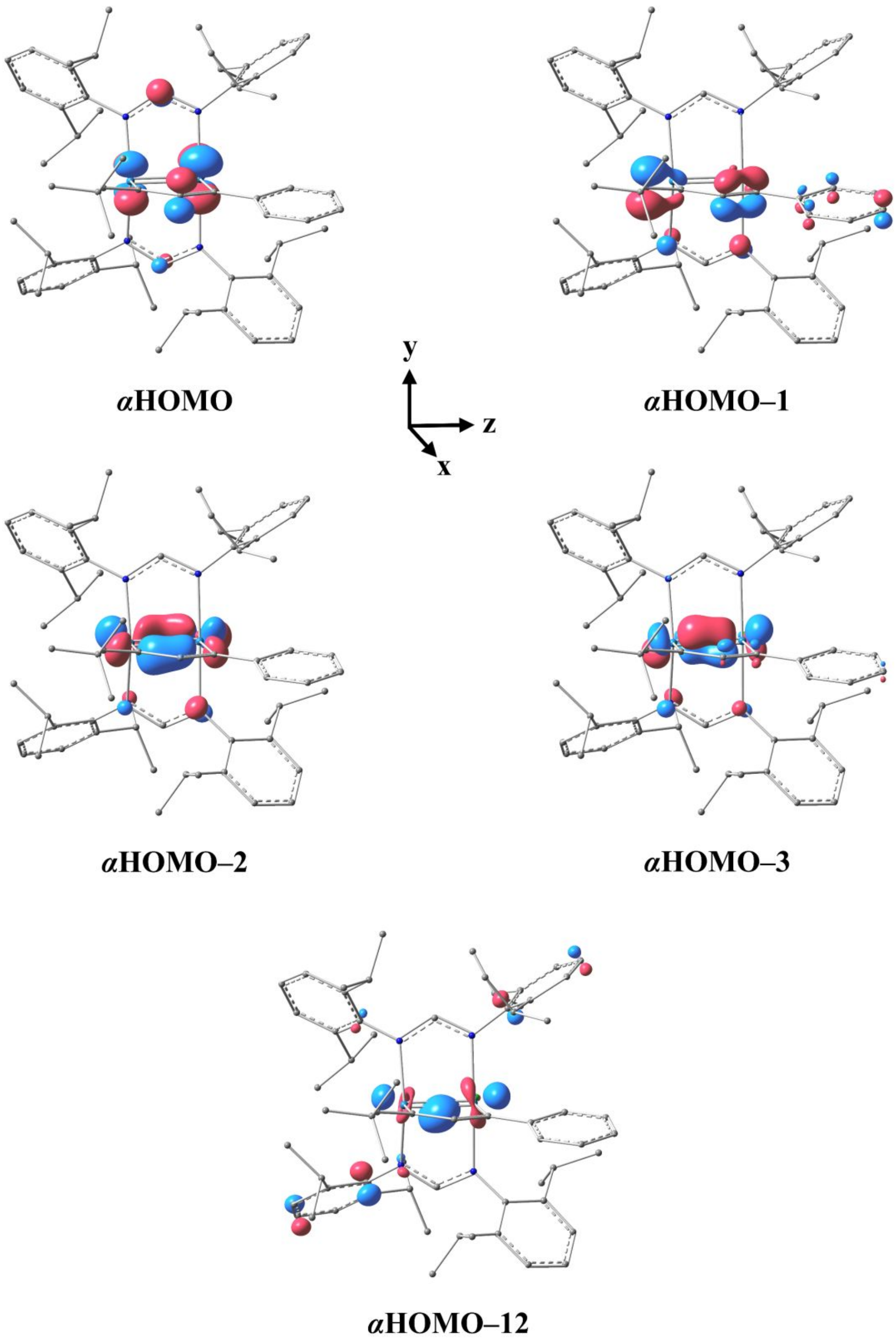

Figure. *S*145. The first half of FMOs of **27** plotted with an isosurface of 0.07 a.u.

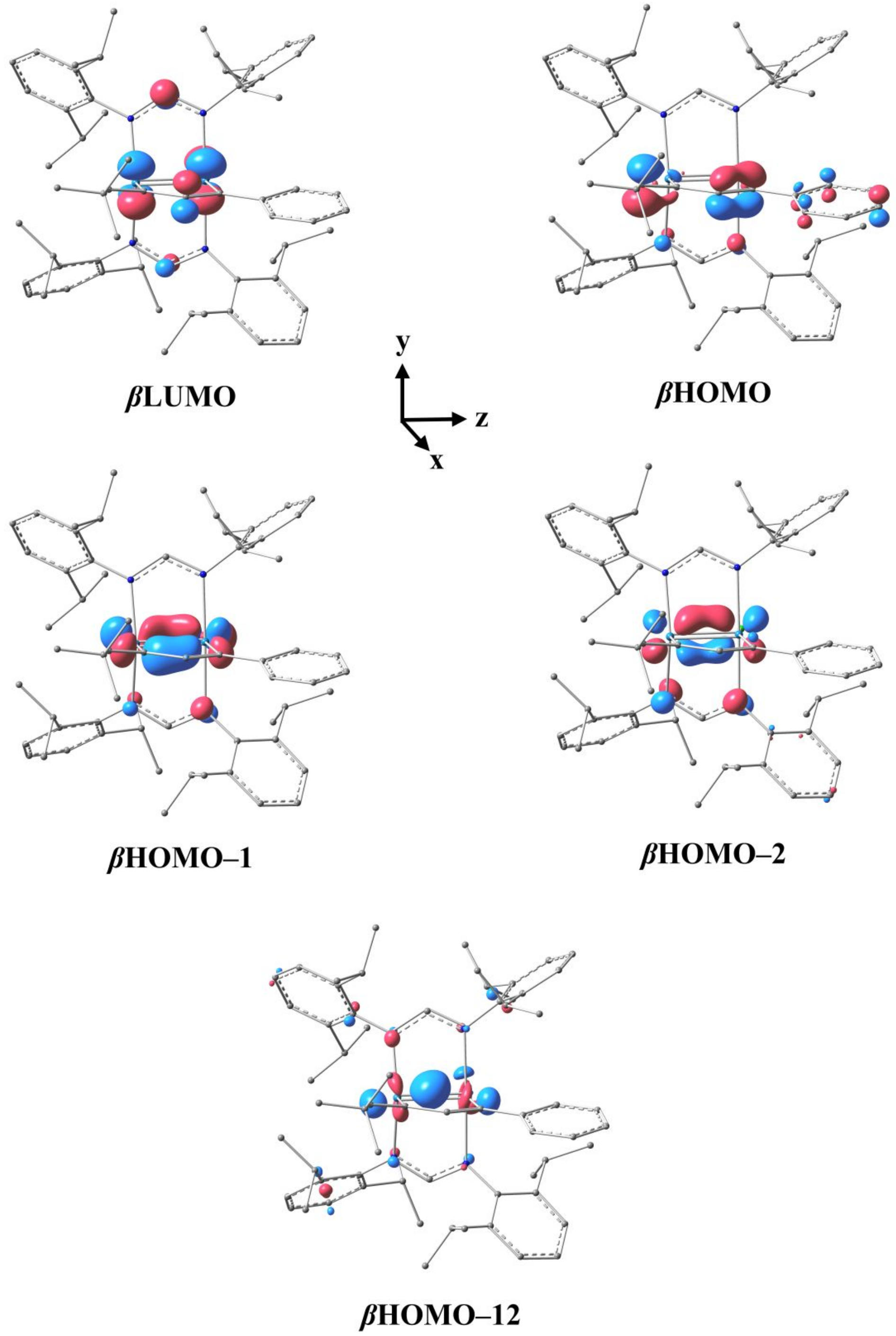

Figure. *S*146. The second half of FMOs of **27** plotted with an isosurface of 0.07 a.u.

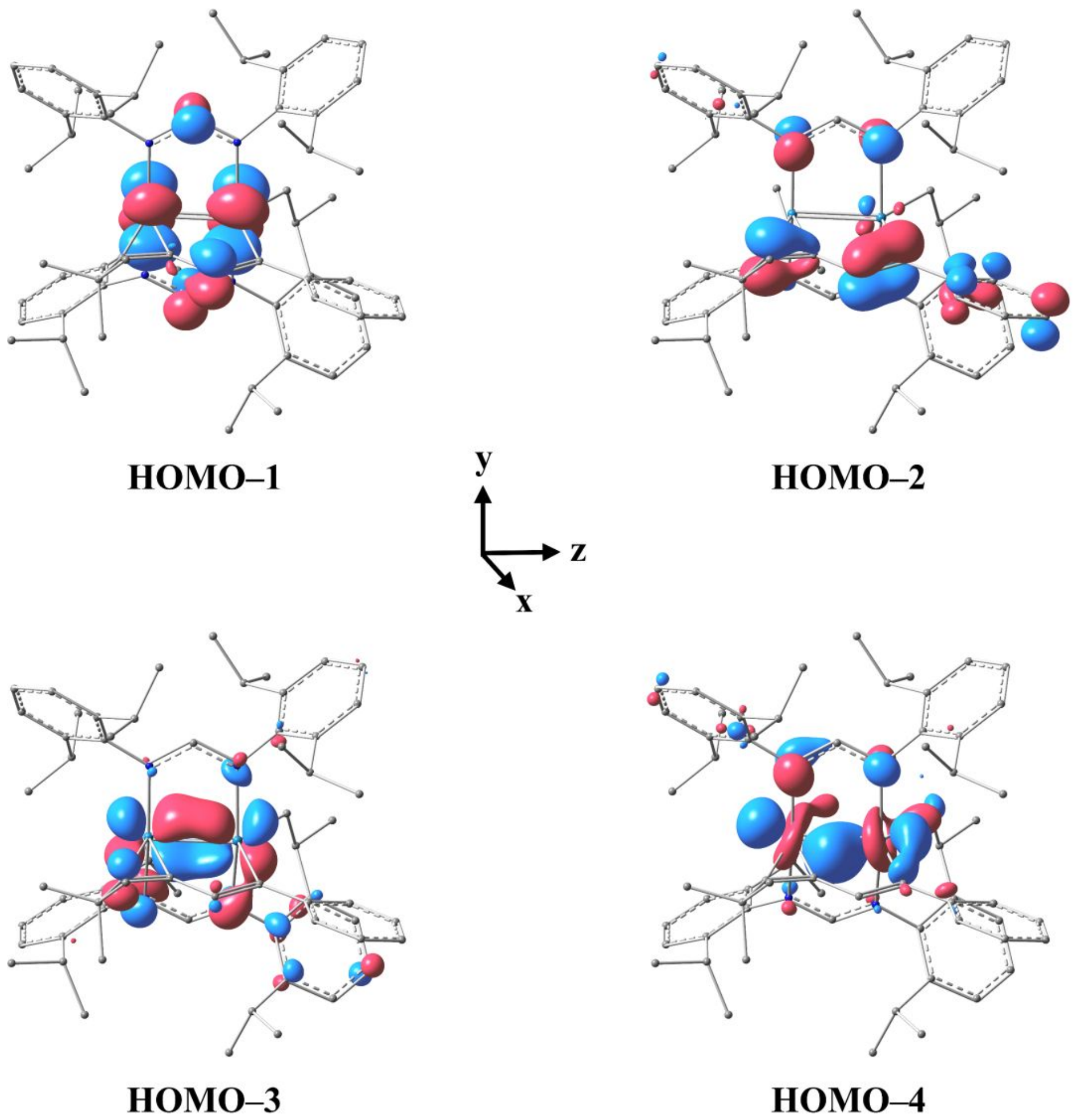

Figure. *S*147. The FMOs of **28** plotted with an isosurface of 0.05 a.u.

7.5 Localized molecular orbital analysis

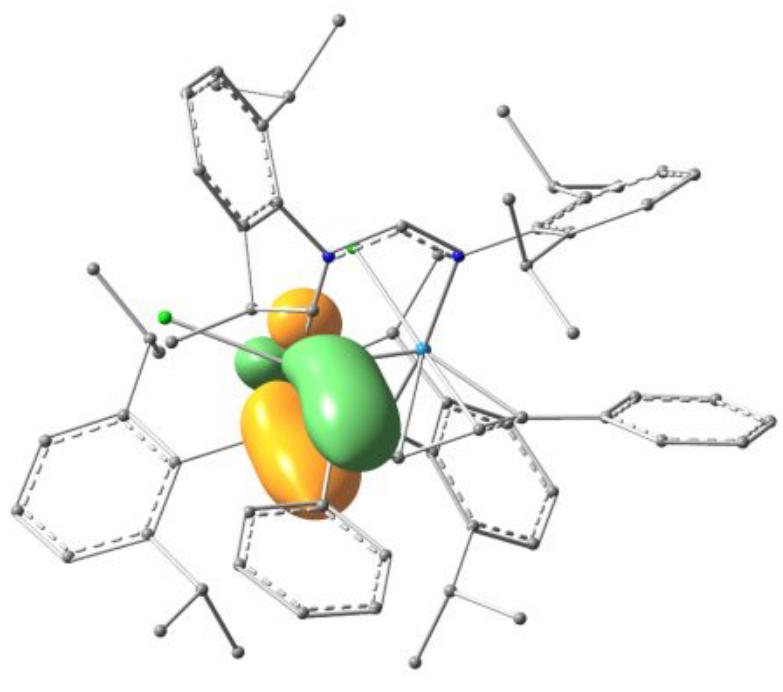

LMO 284
W1 38.4% + C1 38.4% + W2 4.8%

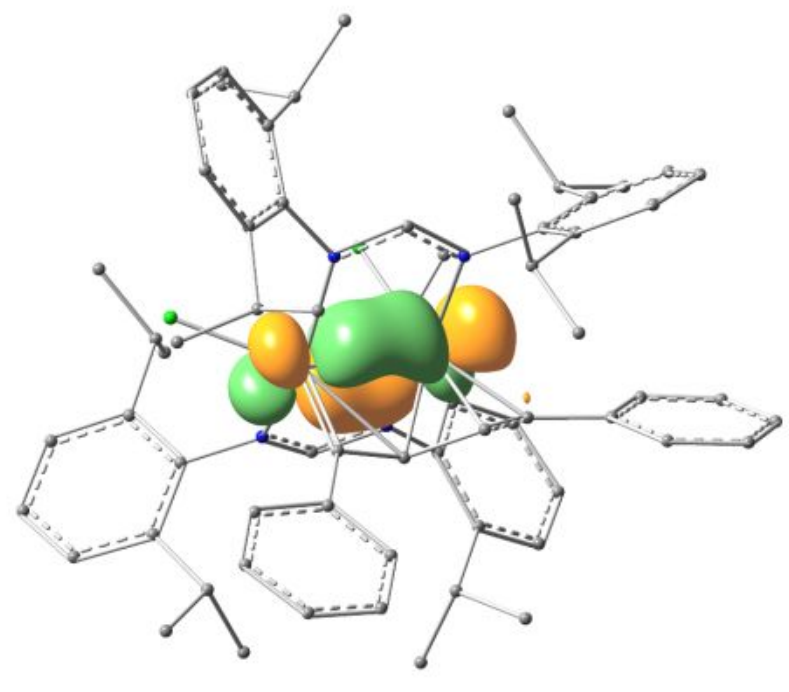

LMO 283
W1 35.7 + W2 44.2%

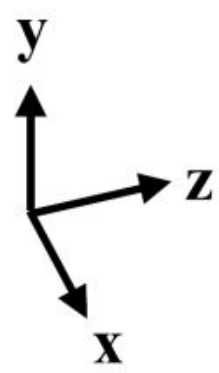

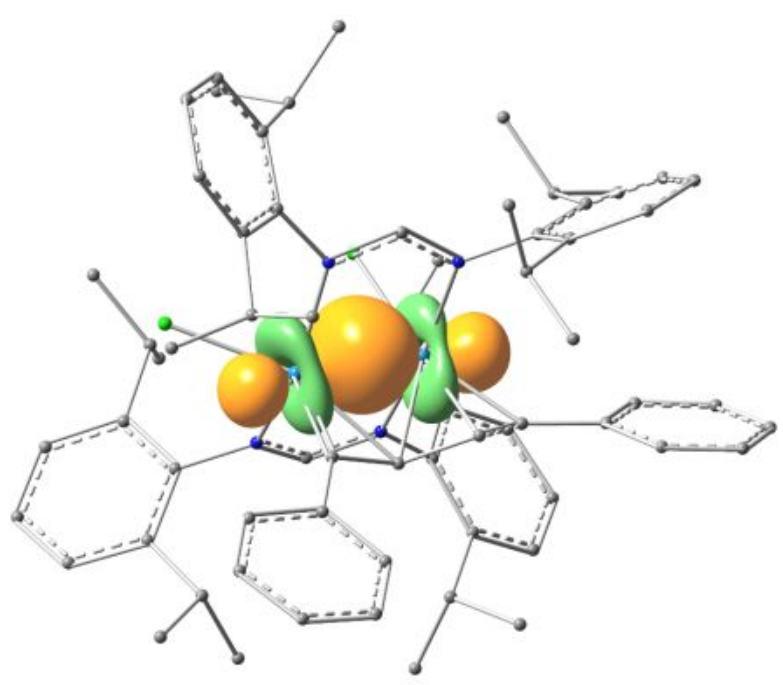

LMO 274
W1 41.1% + W2 46.6%

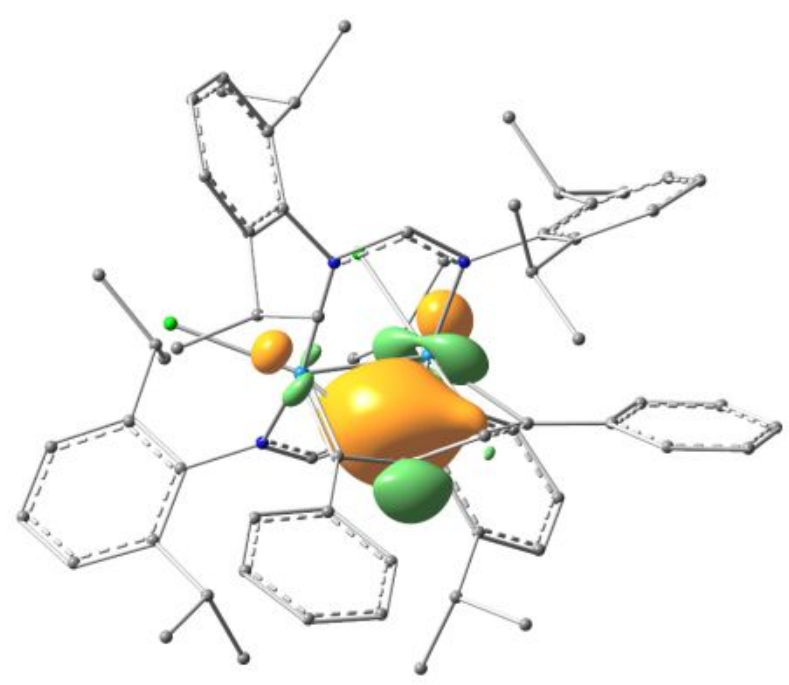

LMO 266
W1 14.0% + W2 26.2% + C2 39.5%

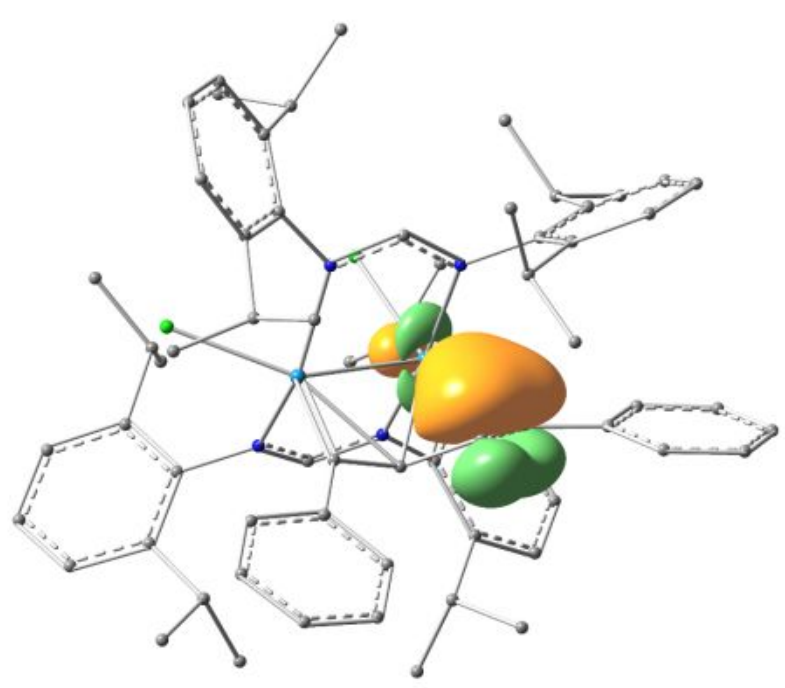

LMO 244
W2 22.6% C3 32.5% + C4 28.3%

Figure. *S*148. The LMOs of **12** plotted with an isosurface of 0.06 a.u.

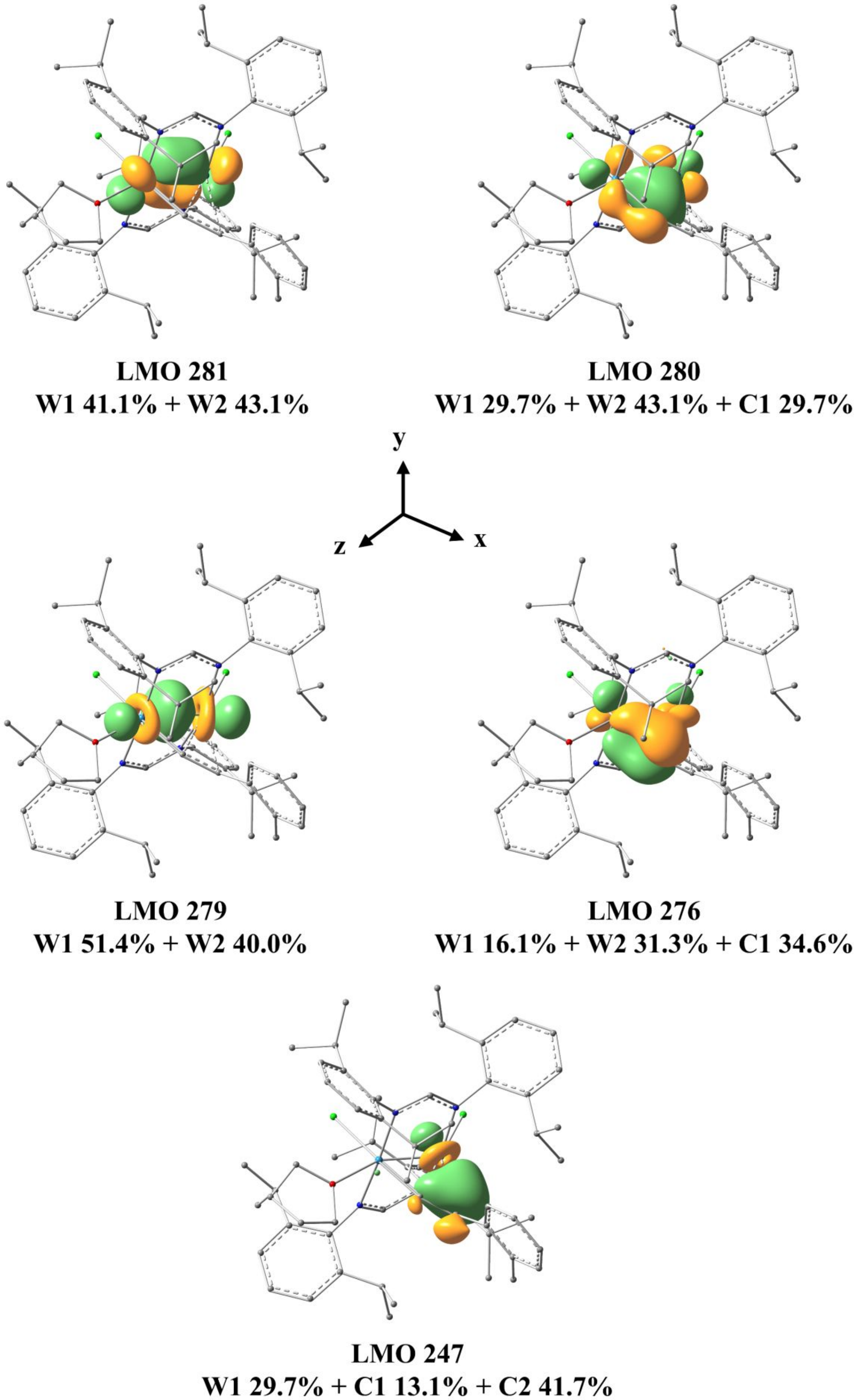

Figure. *S*149. The LMOs of **20** plotted with an isosurface of 0.06 a.u.

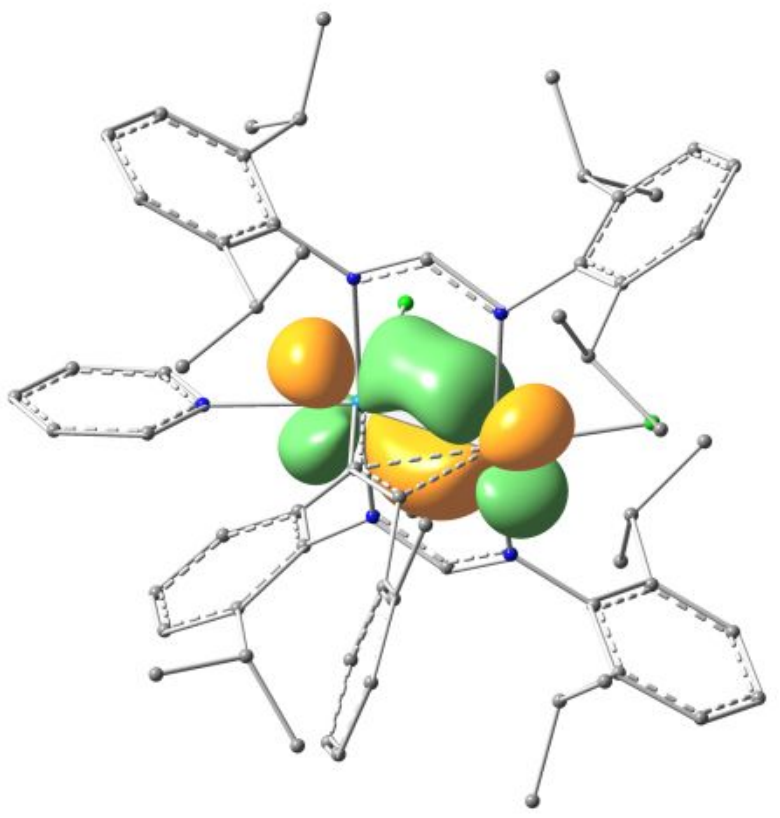

LMO 282
W1 38.2% + W2 43.8%

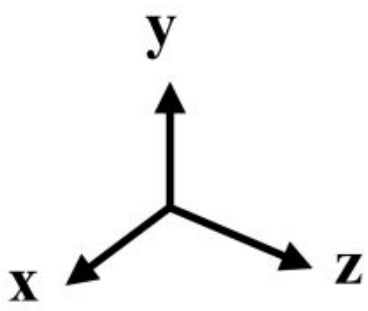

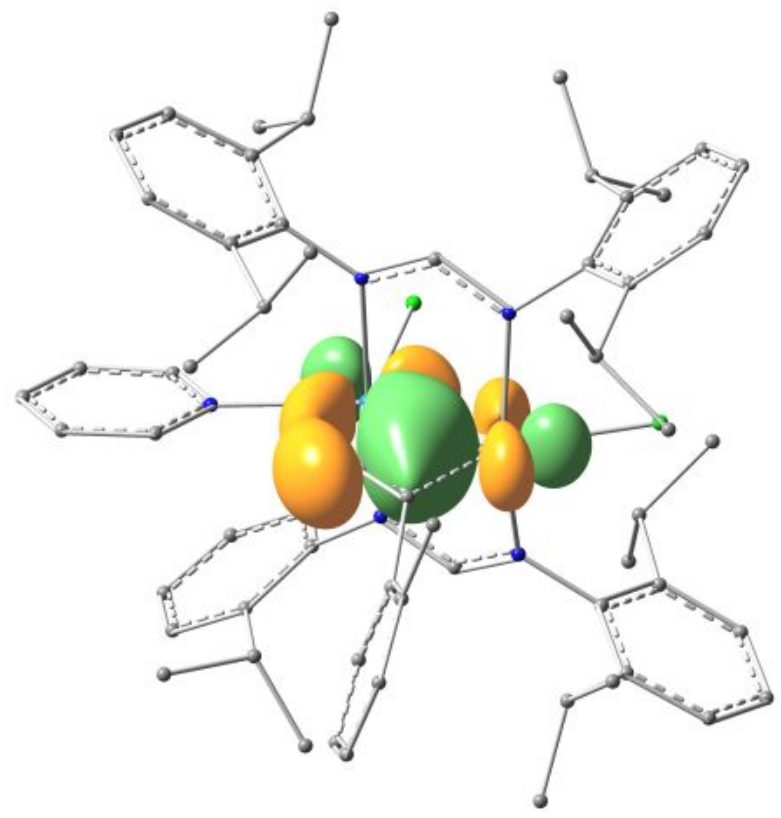

LMO 281
W1 53.5% + W2 36.9%

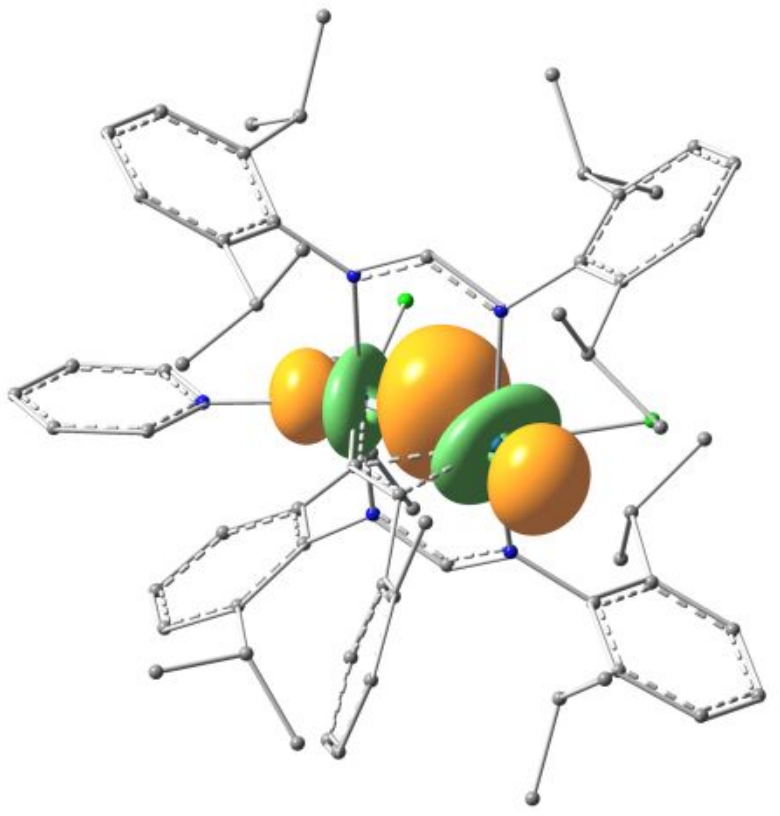

LMO 280
W1 28.6% + W2 29.2% + C6 32.2%

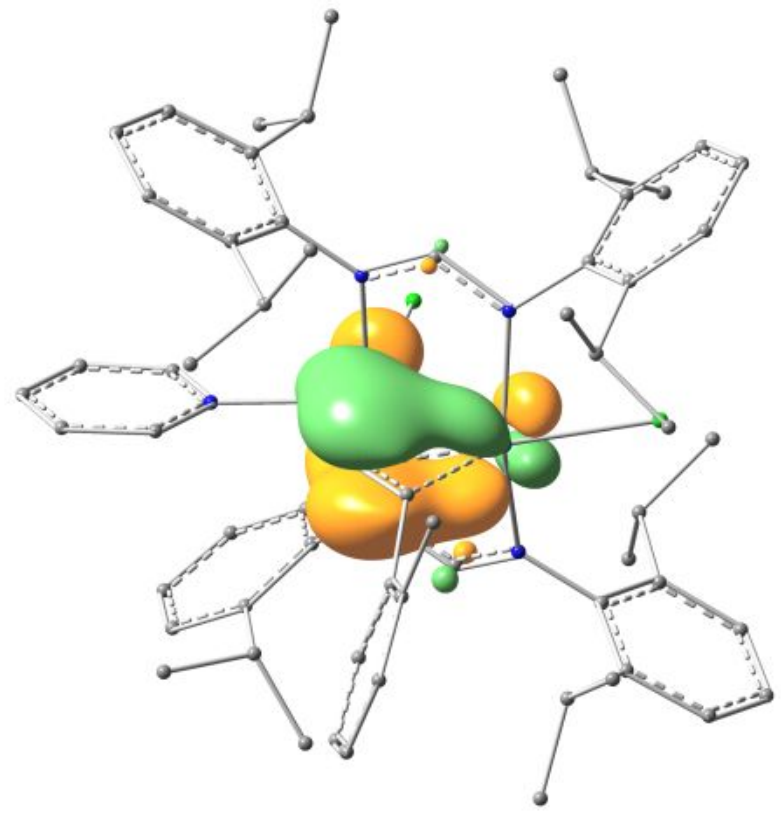

LMO 264
W1 16.4% + W2 31.0% + C6 34.1%

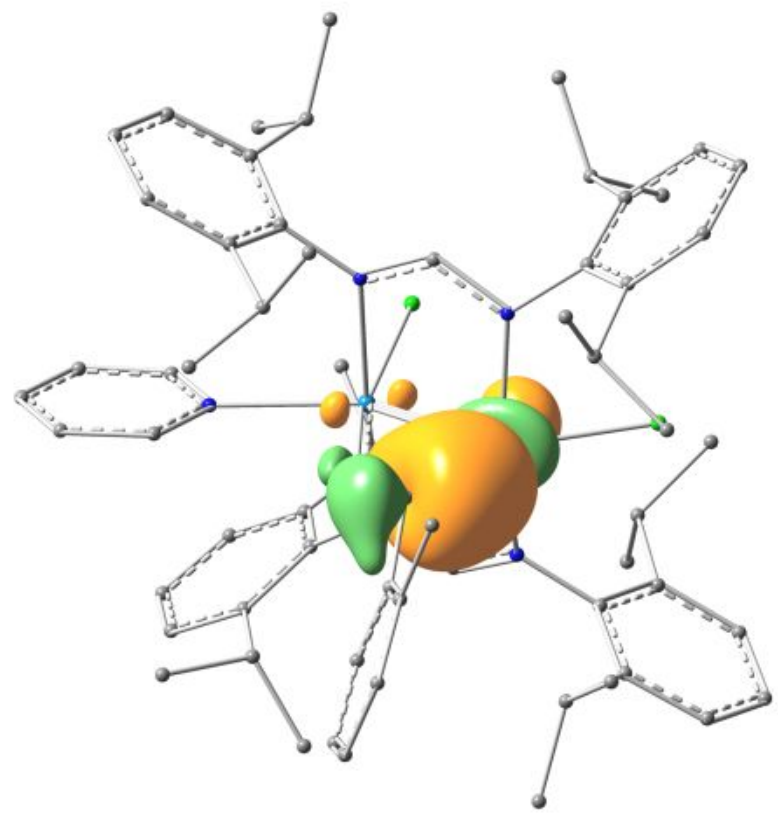

LMO 251
W1 31.0% + C6 11.2% + C7 42.3%

Figure. *S*150. The LMOs of **21** plotted with an isosurface of 0.05 a.u.

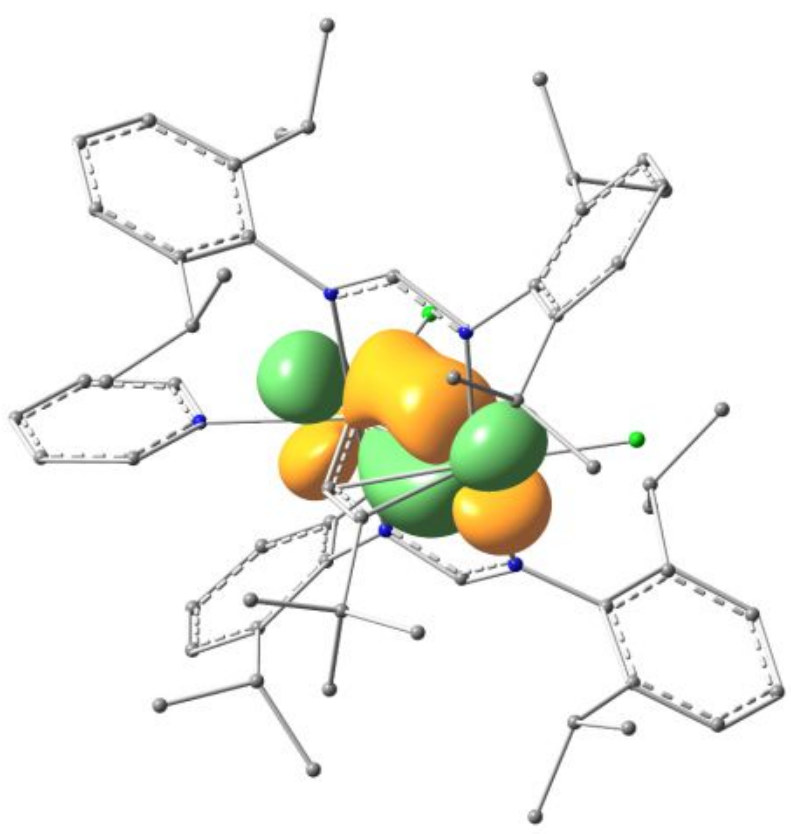

LMO 274
W1 38.0% + W2 43.7%

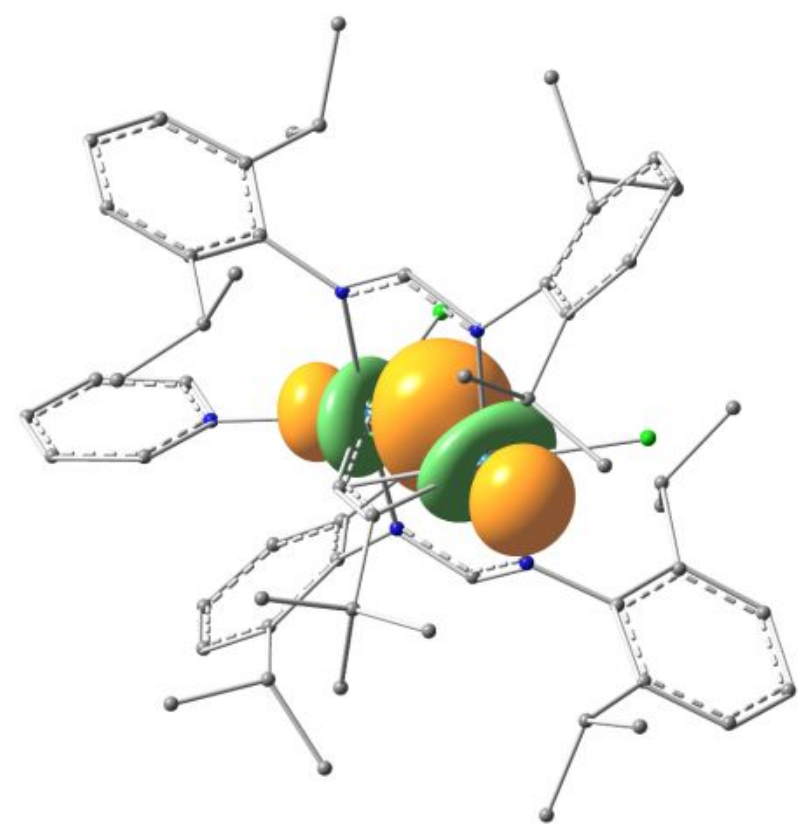

LMO 273
W1 37.9% + W2 52.3%

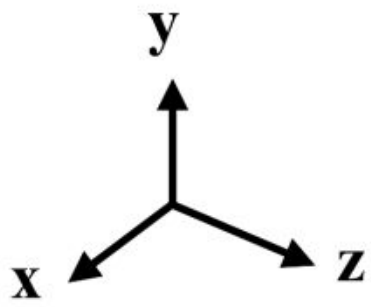

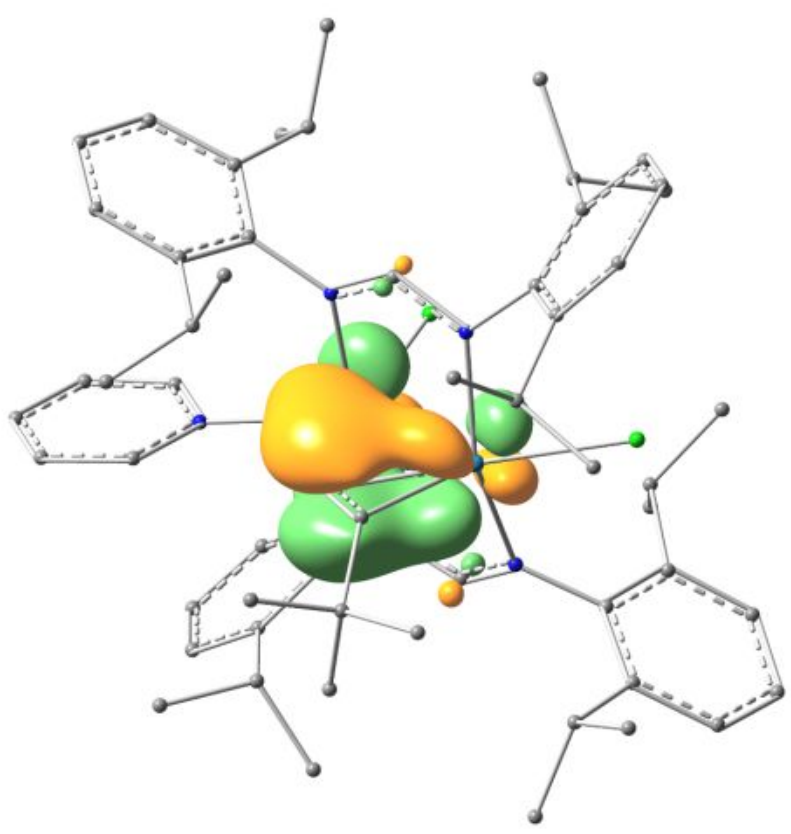

LMO 272
W1 28.7% + W2 28.3% + C1 32.5%

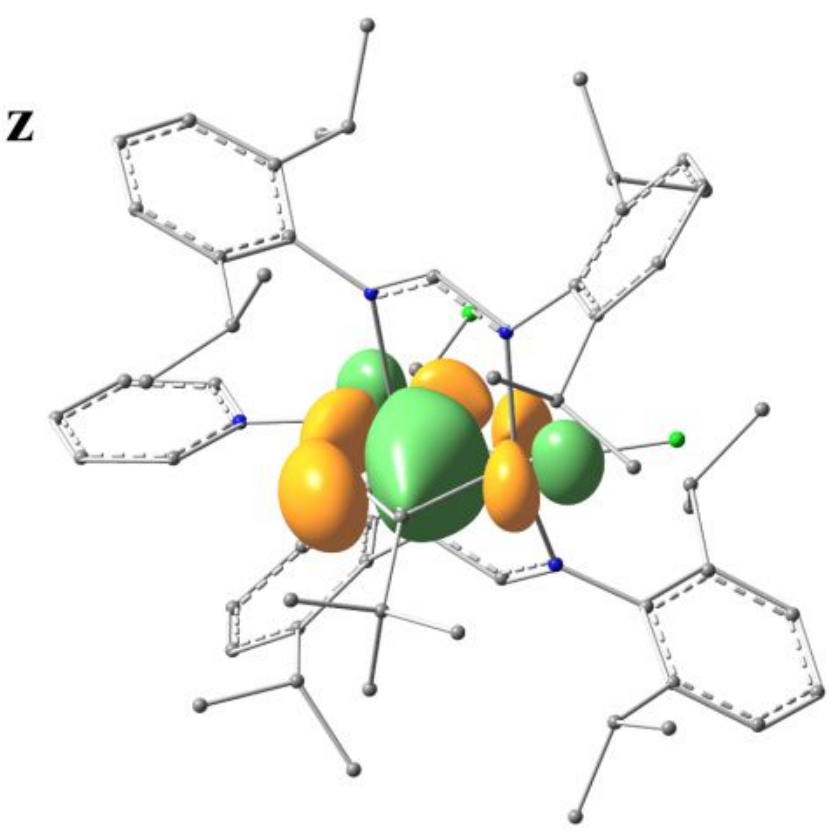

LMO 267
W1 31.6% + W2 16.1% + C1 33.5%

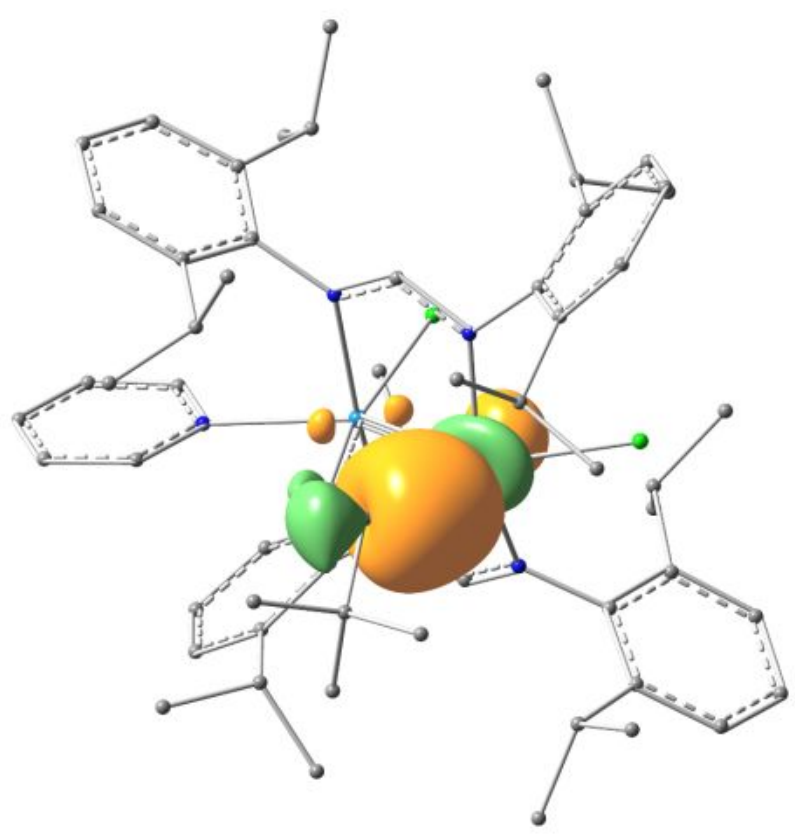

LMO 256
W2 29.7% + C1 13.1% + C2 41.7%

Figure. *S*151. The LMOs of **22** plotted with an isosurface of 0.06 a.u.

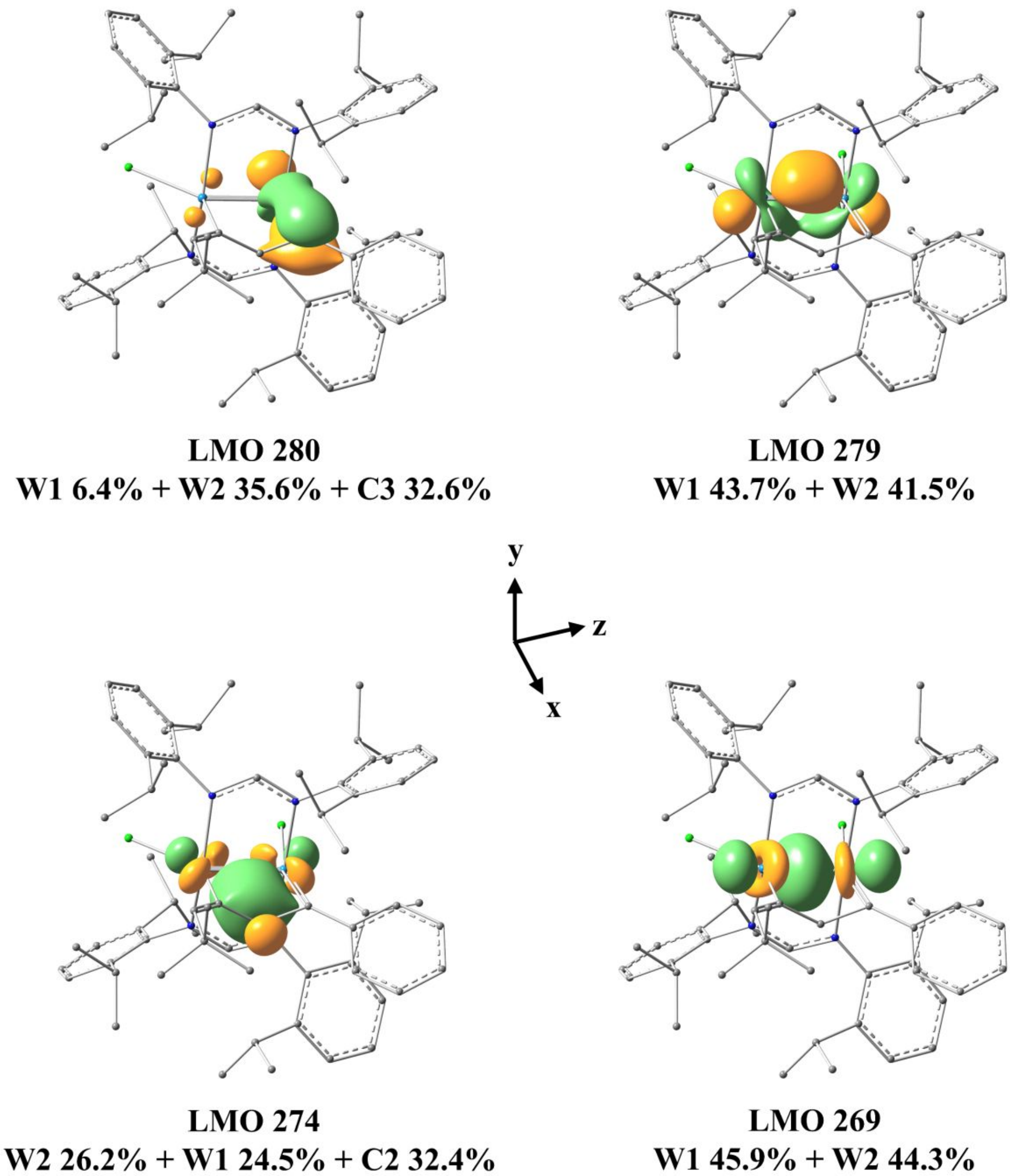

Figure. *S*152. The LMOs of **24** plotted with an isosurface of 0.06 a.u.

7.6 Nucleus-independent chemical shift (NICS) analysis

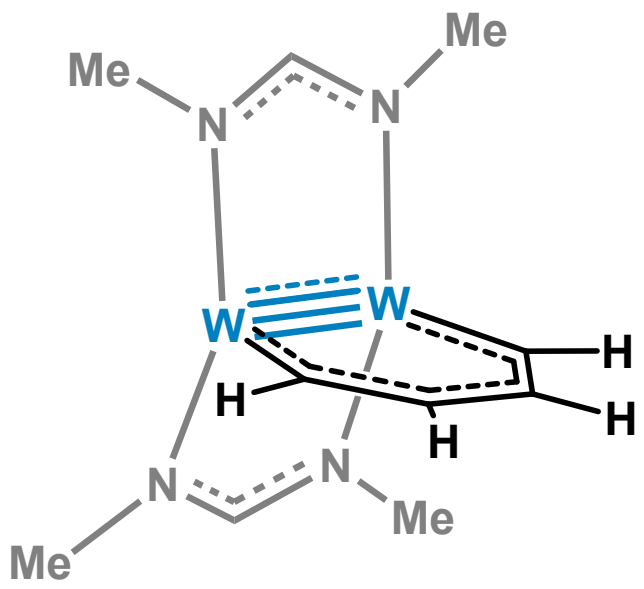

5-S

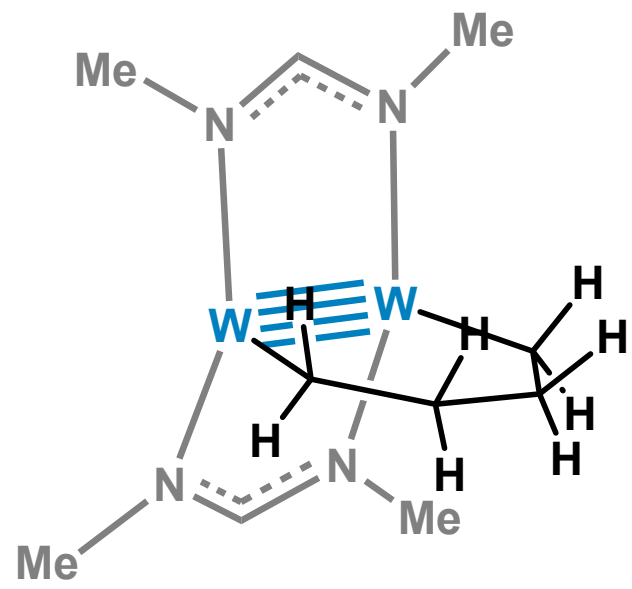

5-H

	**5** (exp.)	**5** (cal.)	**5-S** (cal.)	**5-H** (cal.)
W–W (Å)	2.2404(3)	2.2867	2.2693	2.2346
W–C_α (Å)	2.006(5)<br>1.989(4)	2.0350<br>2.0196	1.9916<br>1.9916	2.1542<br>2.1542
C_α–C_β (Å)	1.361(8)<br>1.383(8)	1.4193<br>1.3978	1.4098<br>1.4098	1.5509<br>1.5509
C_β–C_β (Å)	1.397(7)	1.4143	1.4069	1.5800

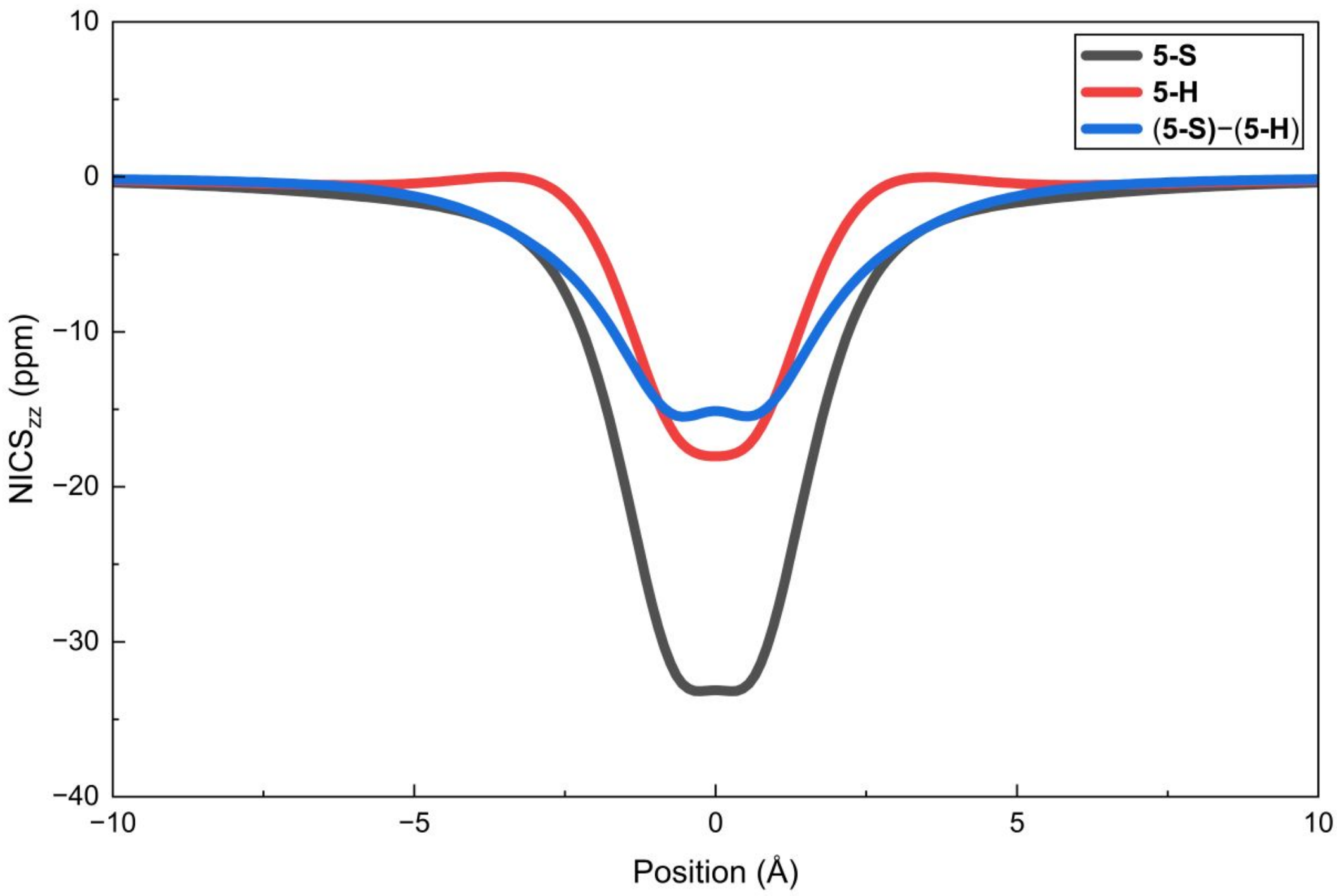

Figure. *S*153. Comparison of structural parameters and $NICS_{zz}$ line scans for **5-S** and **5-H**.

7.7 Cartesian coordinates for the optimized geometries.

Table *S*23. Cartesian coordinates data sets and thermodynamic data for DFT optimized in at 1 atm and 298.15 K.

[2]1

Electronic Energy (BP86)	−4142.266849
Zero-point correction	1.069763
Thermal correction to Energy	1.141131
Thermal correction to Enthalpy	1.142075
Thermal correction to Gibbs Free Energy	0.961312

C	2.98477200	-2.34927700	0.28840300
C	3.29349000	-3.28645700	-0.73934600
C	4.00577300	-4.45514300	-0.40146900
H	4.23745400	-5.17825600	-1.19195100
C	4.43652000	-4.69900800	0.90496600
H	4.98746000	-5.61510600	1.14715800
C	4.17325000	-3.74823900	1.89594400
H	4.53084300	-3.92181900	2.91673000
C	3.45293200	-2.56918800	1.62181400
C	2.81167400	-0.00010000	0.00003700
H	3.91416400	-0.00015500	0.00004400
C	2.98505300	2.34903100	-0.28835200
C	3.45324800	2.56883900	-1.62176400
C	4.17383100	3.74772600	-1.89590300
H	4.53146800	3.92121500	-2.91668900
C	4.43731600	4.69844200	-0.90493200
H	4.98847100	5.61441000	-1.14712900
C	4.00648900	4.45469700	0.40149900
H	4.23830500	5.17777800	1.19197200
C	3.29393900	3.28617600	0.73938300
C	3.27548500	1.55619400	-2.75670300
H	2.50102500	0.83076900	-2.45721800
C	4.60367000	0.79888800	-2.99821100
H	5.40362500	1.50003800	-3.29965300
H	4.48534100	0.05216500	-3.80240800
H	4.95108300	0.27265700	-2.09274100
C	2.80089800	2.19792500	-4.07890100
H	1.87993500	2.77941400	-3.92390500
H	2.58610000	1.40666500	-4.81829300
H	3.57035300	2.85863100	-4.51922100
C	2.95706700	3.05435800	2.21185700
H	2.40314400	2.10540700	2.29228000
C	2.05375900	4.16691600	2.78415700

H	2.55900200	5.15025700	2.76186900
H	1.79138600	3.94408200	3.83314700
H	1.11518400	4.24863600	2.21578200
C	4.24837300	2.93336600	3.05504900
H	4.91681200	2.14813300	2.66596200
H	4.00064300	2.68505900	4.10273000
H	4.81532900	3.88238000	3.06185100
C	2.95678900	-3.05450400	-2.21184600
H	2.40302300	-2.10545500	-2.29225700
C	4.24820900	-2.93366800	-3.05489100
H	4.81500500	-3.88277600	-3.06169400
H	4.91673500	-2.14856800	-2.66568600
H	4.00063000	-2.68525500	-4.10258400
C	2.05337100	-4.16688400	-2.78431600
H	1.79126100	-3.94401900	-3.83336500
H	1.11466400	-4.24839500	-2.21613300
H	2.55841400	-5.15032500	-2.76190800
C	3.27540400	-1.55651400	2.75676400
H	2.50112600	-0.83089600	2.45728400
C	4.60376500	-0.79952100	2.99829400
H	5.40355300	-1.50085600	3.29974800
H	4.48559500	-0.05277500	3.80249300
H	4.95131500	-0.27336700	2.09283200
C	2.80064800	-2.19815300	4.07894500
H	1.87954700	-2.77941800	3.92392500
H	2.58603300	-1.40685800	4.81835200
H	3.56994100	-2.85905400	4.51925600
Cl	-0.07905700	-1.30781200	-2.51583500
Cl	-0.00560300	-2.32430700	1.97711200
N	2.16843800	-1.18168200	-0.00280100
N	2.16855400	1.18154800	0.00285600
W	-0.00913100	-1.14507200	-0.11555600
C	-3.02708000	2.28023200	-0.53207700
C	-3.32689700	3.36649000	0.33750900
C	-4.13124500	4.41964000	-0.14097100
H	-4.36003200	5.25572200	0.52997200
C	-4.65075500	4.41300500	-1.43861300
H	-5.28068100	5.23855500	-1.78956000
C	-4.35668300	3.33811200	-2.28292500
H	-4.75949800	3.32773700	-3.30195300
C	-3.54254900	2.26648600	-1.86582600
C	-2.82832000	0.00013400	0.00001100
H	-3.93095000	0.00019400	0.00003200
C	-3.02730300	-2.27996000	0.53205100
C	-3.54283200	-2.26618800	1.86577700

C	-4.35703200	-3.33777700	2.28284800
H	-4.75988400	-3.32737700	3.30186100
C	-4.65113400	-4.41265200	1.43852700
H	-5.28111400	-5.23817000	1.78945200
C	-4.13159700	-4.41930000	0.14089600
H	-4.36042700	-5.25535500	-0.53006200
C	-3.32717600	-3.36619400	-0.33755400
C	-3.27071700	-1.14060600	2.86894500
H	-2.47267300	-0.49606900	2.46543300
C	-4.53733800	-0.27925100	3.08633400
H	-5.36368000	-0.88819000	3.49741200
H	-4.33125800	0.53723200	3.80033100
H	-4.89652700	0.17349400	2.14628900
C	-2.75810900	-1.67243400	4.22607200
H	-1.86551100	-2.30040300	4.08409300
H	-2.47998500	-0.82412400	4.87569700
H	-3.52702000	-2.26162600	4.75912900
C	-2.81746800	-3.43924700	-1.77406400
H	-2.29187100	-2.49798400	-1.99958300
C	-1.79854700	-4.58875100	-1.93403400
H	-2.27233300	-5.57206400	-1.75661600
H	-1.37193900	-4.58993200	-2.95196700
H	-0.96891600	-4.48262000	-1.21525000
C	-3.96780800	-3.58915300	-2.79460100
H	-4.70627500	-2.77668600	-2.69290300
H	-3.56820100	-3.56152300	-3.82405600
H	-4.50391200	-4.54830300	-2.67305600
C	-2.81720100	3.43953500	1.77402400
H	-2.29174500	2.49820500	1.99958900
C	-3.96753500	3.58962700	2.79453600
H	-4.50357900	4.54879800	2.67289700
H	-4.70605500	2.77720200	2.69290000
H	-3.56794100	3.56206000	3.82399900
C	-1.79811400	4.58889600	1.93394000
H	-1.37151000	4.59007800	2.95187300
H	-0.96849300	4.48257800	1.21517100
H	-2.27174100	5.57227100	1.75644500
C	-3.27037200	1.14089800	-2.86897000
H	-2.47225800	0.49645300	-2.46544600
C	-4.53690100	0.27940200	-3.08632300
H	-5.36330400	0.88823100	-3.49744200
H	-4.33071900	-0.53710000	-3.80026900
H	-4.89604400	-0.17333700	-2.14625800
C	-2.75782700	1.67272800	-4.22612000
H	-1.86529800	2.30080300	-4.08417200

H	-2.47960800	0.82442000	-4.87570700
H	-3.52680200	2.26181100	-4.75920500
Cl	-0.07898100	1.30780100	2.51584800
Cl	-0.00534300	2.32436500	-1.97709400
N	-2.19148300	1.18121700	-0.08186400
N	-2.19160400	-1.18101400	0.08186100
W	-0.00903000	1.14509800	0.11555900

[1]2

Electronic Energy (BP86)	−3921.953361
Zero-point correction	1.185774
Thermal correction to Energy	1.263782
Thermal correction to Enthalpy	1.264726
Thermal correction to Gibbs Free Energy	1.063279

C	2.73807900	-0.52992100	-0.53041600
H	3.79328200	-0.74923000	-0.75340900
C	2.42035500	-2.88421800	-0.30394800
C	2.01118200	-3.87463900	-1.24552400
C	2.54723000	-5.17268900	-1.14429900
H	2.23988800	-5.92978700	-1.87422900
C	3.46267500	-5.51198300	-0.14398000
H	3.87185100	-6.52610000	-0.08556800
C	3.84273900	-4.53992000	0.78555600
H	4.54835300	-4.80534100	1.58075900
C	3.33427700	-3.22663100	0.74004600
C	1.01929600	-3.59712900	-2.37467100
H	0.71521600	-2.53648000	-2.30992500
C	-0.25261200	-4.46497600	-2.23469300
H	-0.01765100	-5.54064400	-2.32042700
H	-0.98382300	-4.21733100	-3.02311800
H	-0.74350500	-4.31059300	-1.25773200
C	1.66784800	-3.78191200	-3.76531400
H	2.55695700	-3.13983700	-3.87973500
H	0.95049200	-3.52129600	-4.56299800
H	1.98397600	-4.82789700	-3.92707000
C	3.77907700	-2.26028600	1.84282100
H	3.26745900	-1.29518400	1.69065900
C	5.30276300	-2.00595400	1.78560900
H	5.87013400	-2.94327400	1.92265800
H	5.61354900	-1.30992900	2.58380000
H	5.60870600	-1.57563200	0.81645800
C	3.36689300	-2.77508200	3.24177800
H	2.27809500	-2.94069700	3.29008400
H	3.64954800	-2.03984400	4.01702600
H	3.86868500	-3.72683900	3.49027500

C	3.31762300	1.76449500	-0.79866800
C	3.73269700	1.89250000	-2.16168800
C	4.61301800	2.93977000	-2.50158300
H	4.92802400	3.04416200	-3.54576400
C	5.09630800	3.83705800	-1.54565400
H	5.77290900	4.64768600	-1.83682600
C	4.72918000	3.66639000	-0.20766800
H	5.14304600	4.33598500	0.55381100
C	3.85704000	2.63566800	0.19481000
C	3.32419200	0.92452900	-3.27575800
H	2.57982100	0.22182700	-2.87293000
C	4.54137600	0.09777800	-3.75471900
H	4.23399000	-0.62830300	-4.52795100
H	5.00466900	-0.46336800	-2.92507300
H	5.32253400	0.74432300	-4.19285400
C	2.65501800	1.64164800	-4.46833100
H	3.33851400	2.35706700	-4.95918300
H	1.75727500	2.19357400	-4.14544100
H	2.34235000	0.90461600	-5.22840700
C	3.62619900	2.43084900	1.69148400
H	2.80049300	1.71147300	1.80910900
C	3.22880400	3.73024600	2.42382900
H	4.05071200	4.46832900	2.42318600
H	2.98705500	3.51221100	3.47883400
H	2.34332500	4.19829800	1.96604400
C	4.89107600	1.81867500	2.33946500
H	5.17883600	0.87412700	1.85013600
H	4.71888300	1.61336000	3.41160500
H	5.75009000	2.50913600	2.26248300
C	-2.73017000	0.54301400	-0.55790400
H	-3.78304200	0.76360400	-0.79027200
C	-2.41279800	2.89744200	-0.29994200
C	-3.28856600	3.24910900	0.77266300
C	-3.77360200	4.57024500	0.84012100
H	-4.44982700	4.84339900	1.65788500
C	-3.40859500	5.53969300	-0.09832000
H	-3.79576200	6.56112900	-0.02020000
C	-2.54368500	5.18634400	-1.13779800
H	-2.25596200	5.93887000	-1.88029600
C	-2.03675100	3.87858100	-1.26426800
C	-1.10897700	3.57830000	-2.44074500
H	-0.87932000	2.49933700	-2.42606800
C	0.22298900	4.35300400	-2.31127600
H	0.05424000	5.44456300	-2.32102600
H	0.90314000	4.10943400	-3.14536000

H	0.74582200	4.11060800	-1.36845200
C	-1.78600800	3.86857400	-3.79951600
H	-2.72962100	3.30746400	-3.90382200
H	-1.11911400	3.57629900	-4.62935800
H	-2.01725900	4.94182300	-3.92056900
C	-3.30192600	-1.75362100	-0.84738500
C	-3.68990900	-1.89208900	-2.21687400
C	-4.56728500	-2.93884000	-2.56599900
H	-4.86185500	-3.05060700	-3.61539000
C	-5.07263700	-3.82628900	-1.61230100
H	-5.74690500	-4.63655800	-1.90979900
C	-4.72951300	-3.64720100	-0.26899300
H	-5.15918400	-4.31044400	0.48926400
C	-3.86098300	-2.61682300	0.14214400
C	-3.25109900	-0.93624700	-3.32917800
H	-2.51688400	-0.23056400	-2.91338200
C	-2.55158200	-1.66791200	-4.49538400
H	-1.66562700	-2.22091900	-4.14234200
H	-2.21433200	-0.93997500	-5.25370400
H	-3.22430400	-2.38552000	-4.99787200
C	-4.45296500	-0.11191800	-3.84886300
H	-5.22413500	-0.76073700	-4.30114000
H	-4.12300100	0.60559400	-4.62070600
H	-4.93596300	0.45861700	-3.03704200
C	-3.65353100	-2.40478300	1.64126200
H	-2.82254900	-1.69258700	1.76712700
C	-4.92357900	-1.77736300	2.26375600
H	-5.19607200	-0.83477500	1.76240400
H	-4.76784000	-1.56486700	3.33706500
H	-5.78628100	-2.46213000	2.17782700
C	-3.28018400	-3.70261700	2.38874800
H	-4.10901900	-4.43281200	2.38010800
H	-3.05413900	-3.47942900	3.44626300
H	-2.39155200	-4.18163800	1.94901000
C	-3.73303100	2.27700300	1.86966500
H	-3.22309500	1.31208700	1.70793900
C	-3.32735200	2.77780600	3.27522900
H	-3.83406200	3.72442900	3.53301200
H	-2.23967700	2.94648400	3.33085600
H	-3.61131600	2.03321900	4.04104400
C	-5.25785600	2.02904700	1.80457300
H	-5.57551600	1.32857900	2.59609100
H	-5.56192300	1.60835200	0.83065600
H	-5.81973700	2.96904700	1.94607100
C	-0.19180800	-1.24946100	5.55282300

H	0.75311700	-1.79155600	5.36709300
H	-1.03259700	-1.88251000	5.22713300
C	-0.29962900	-0.76301600	7.00346700
H	0.16644100	-1.46500000	7.71304600
H	-1.35748600	-0.63474200	7.29292000
C	0.40956000	0.60497000	6.94219700
H	0.13950200	1.27373500	7.77495200
H	1.50596100	0.47354400	6.94704100
C	-0.06158800	1.14295000	5.58819700
H	-1.04963300	1.63426800	5.66389600
H	0.64301000	1.83487600	5.10044500
Cl	0.02059400	0.03017600	-2.52327500
Cl	-0.22965200	-2.18322700	2.00476400
Cl	0.21997900	2.14453700	2.05207100
Li	-0.12037900	-0.01616300	2.80678700
N	2.33360500	0.75640400	-0.42090700
N	1.86583400	-1.54996000	-0.37799700
N	-1.85984000	1.56094200	-0.37974100
N	-2.32322500	-0.74407800	-0.45974200
O	-0.19180800	-0.03592700	4.73195000
W	0.21545000	1.10873100	-0.19197100
W	-0.21059400	-1.09665100	-0.21492100

^{1}3

Electronic Energy (BP86)	−3718.216575
Zero-point correction	1.244365
Thermal correction to Energy	1.323139
Thermal correction to Enthalpy	1.324083
Thermal correction to Gibbs Free Energy	1.128937

C	4.23058900	-0.86433400	-0.68377500
H	3.75122500	-1.20925700	-1.60424800
C	5.61524400	-0.96482200	-0.49956900
H	6.23450500	-1.40492200	-1.28612400
C	6.17615500	-0.49290800	0.69541800
H	7.25563700	-0.55156300	0.86854700
C	5.32444200	0.05645300	1.66393000
H	5.70994900	0.44377300	2.61106900
C	3.94963700	0.10829400	1.40144500
H	3.24634400	0.52152500	2.12853700
C	-2.06630800	0.15930400	-2.86099900
H	-0.98841400	0.02216700	-2.98934900
C	-2.93561300	0.29032600	-3.94280700
H	-2.52089500	0.24726100	-4.95418700
C	-4.31437900	0.47694100	-3.72657500
H	-5.00991600	0.58518100	-4.56348700

C	-4.76703600	0.51531800	-2.39640300
H	-5.82542700	0.65192700	-2.15711300
C	-3.85334800	0.37520800	-1.35272000
H	-4.17687300	0.40042500	-0.30774500
C	0.11375400	2.79632300	0.14997400
H	0.21426900	3.88489500	0.28147400
C	2.47528900	2.78971300	0.14806400
C	3.35211300	3.03218500	-0.95206800
C	4.61152700	3.61487800	-0.70613900
H	5.28385400	3.79079900	-1.55293700
C	5.00603100	4.00225500	0.57712500
H	5.99009800	4.45309300	0.74557300
C	4.10554000	3.84780000	1.63407200
H	4.38417000	4.20210900	2.63269600
C	2.83791900	3.25693500	1.45383000
C	2.94296600	2.81563500	-2.40846100
H	1.99370000	2.25869300	-2.41721400
C	3.97368300	2.00112400	-3.21786900
H	4.93254100	2.54103200	-3.32004600
H	3.59021400	1.81136000	-4.23501900
H	4.17595800	1.02615100	-2.74866600
C	2.70543900	4.18437400	-3.09098600
H	1.97394800	4.79201600	-2.53485200
H	2.32772600	4.04236800	-4.11934000
H	3.64219700	4.76716000	-3.15354700
C	1.89735600	3.24589600	2.66544800
H	1.02321500	2.62098600	2.42244700
C	2.52303200	2.64801600	3.94535100
H	2.83680200	1.60093100	3.80494300
H	1.78103900	2.65847900	4.76225100
H	3.39990400	3.22368700	4.29038300
C	1.41085100	4.68722000	2.95935500
H	2.26138600	5.34418100	3.21408900
H	0.70847200	4.69819300	3.81096200
H	0.89974100	5.13522400	2.09029400
C	-2.23797700	3.16644100	0.16570100
C	-3.19145500	3.16457300	1.23282500
C	-4.31017100	4.01369800	1.14546000
H	-5.04226800	4.01836100	1.95746700
C	-4.50250500	4.86561800	0.05187200
H	-5.37923900	5.52052700	0.00760600
C	-3.55830600	4.87524100	-0.97469300
H	-3.70020400	5.54667000	-1.82852400
C	-2.42331400	4.03735800	-0.95234400
C	-2.97770800	2.33664300	2.50111500

H	-2.53366700	1.36261000	2.19983800
C	-1.95774300	3.03124700	3.43074300
H	-1.00003300	3.19451300	2.91609800
H	-1.76344700	2.41586100	4.32641600
H	-2.34579000	4.01197500	3.76014800
C	-4.27280900	2.04025500	3.27920400
H	-4.71041100	2.95849000	3.70966200
H	-4.05353700	1.36778000	4.12304600
H	-5.04107900	1.56189400	2.64736200
C	-1.45735400	4.14711000	-2.13704100
H	-0.69039000	3.35992700	-2.04481600
C	-0.74584700	5.52125700	-2.12683900
H	-1.48022700	6.34421600	-2.18269100
H	-0.06950100	5.61707300	-2.99315100
H	-0.15238700	5.67288300	-1.20890600
C	-2.16620000	3.93581800	-3.49417400
H	-2.70795600	2.97784300	-3.52229400
H	-1.42380600	3.93535500	-4.31143300
H	-2.88957500	4.74224500	-3.70840700
C	-0.42238500	-2.79188200	0.26109300
H	-0.51231400	-3.87665400	0.42993600
C	1.90154400	-3.21149300	-0.07083900
C	2.38254900	-3.72961500	-1.31180000
C	3.55140100	-4.51842200	-1.30956400
H	3.92298600	-4.91339700	-2.26121500
C	4.23030800	-4.82453000	-0.12692200
H	5.14177000	-5.43186500	-0.15018100
C	3.70335700	-4.38288500	1.08989700
H	4.19715400	-4.66699600	2.02559200
C	2.53767500	-3.59282000	1.15016000
C	1.62123200	-3.58704200	-2.63094600
H	0.85512300	-2.80601700	-2.49877300
C	2.51145800	-3.17906000	-3.82440400
H	3.04847800	-2.23736100	-3.63254800
H	1.88709000	-3.02537600	-4.72157500
H	3.25075600	-3.96184700	-4.07192500
C	0.91476100	-4.92571200	-2.95189700
H	1.65425600	-5.72652200	-3.13265800
H	0.29205600	-4.83029400	-3.85940600
H	0.26923200	-5.25308100	-2.12215400
C	1.95788100	-3.28455600	2.53195800
H	1.15245500	-2.54154700	2.41306000
C	1.35435100	-4.57926700	3.13159100
H	0.58035000	-5.01430600	2.47657200
H	0.89673000	-4.37956300	4.11600400

H	2.13683100	-5.34668000	3.27037800
C	2.98416600	-2.69054900	3.52011000
H	3.78084400	-3.41276400	3.77338200
H	2.48106100	-2.41361200	4.46279800
H	3.46549200	-1.78873700	3.11157500
C	-2.80249300	-2.65400200	0.58900200
C	-3.43346100	-3.37421700	-0.46868100
C	-4.73897400	-3.87074400	-0.26630900
H	-5.22706900	-4.41784300	-1.08021900
C	-5.41656000	-3.68734900	0.93904800
H	-6.43296200	-4.07396500	1.07008300
C	-4.76989400	-3.02656600	1.98999800
H	-5.28572500	-2.91742300	2.94787100
C	-3.46727800	-2.51179600	1.84981500
C	-2.75717300	-3.70943800	-1.79946500
H	-1.78784300	-3.18413200	-1.84402800
C	-2.49451600	-5.23306600	-1.87538900
H	-1.85610300	-5.58121600	-1.04538100
H	-2.00243500	-5.50074500	-2.82531900
H	-3.44307000	-5.79556000	-1.81714300
C	-3.58792700	-3.26240800	-3.02186300
H	-4.53793500	-3.82115600	-3.09369600
H	-3.02583800	-3.45175300	-3.95306300
H	-3.83012100	-2.19000300	-2.97946700
C	-2.76434800	-1.91008200	3.06779200
H	-2.18609100	-1.02994700	2.72861200
C	-1.75227000	-2.92212000	3.64965800
H	-2.27432200	-3.83034900	4.00144600
H	-1.21015900	-2.48017600	4.50389600
H	-1.00894200	-3.22167700	2.89810500
C	-3.71954000	-1.45297700	4.18661300
H	-4.52819100	-0.80577100	3.81089200
H	-3.15453800	-0.89053900	4.94951800
H	-4.18435300	-2.31271700	4.70233000
Cl	1.39084000	-0.18821800	-2.63234400
Cl	0.30318600	0.01171100	2.25558600
N	3.40028800	-0.33868500	0.24923900
N	-2.50264900	0.20446700	-1.55906700
N	1.22649000	2.05937400	-0.05796300
N	-1.11837100	2.24908900	0.20714900
N	0.78405900	-2.26960000	-0.05669300
N	-1.51699700	-2.01003400	0.37881900
W	0.97184800	-0.11893800	-0.25672800
W	-1.26331900	0.10373700	0.16007400

^{1}4

Electronic Energy (BP86)	−2533.500137
Zero-point correction	1.172182
Thermal correction to Energy	1.240502
Thermal correction to Enthalpy	1.241446
Thermal correction to Gibbs Free Energy	1.069725

C	2.79440800	-0.28407600	0.25860700
H	3.86184300	-0.46867500	0.45086900
C	2.54360900	-2.63870400	0.27404900
C	2.84355300	-3.44353700	-0.86473600
C	3.35842700	-4.73813200	-0.65636200
H	3.59317600	-5.36738100	-1.52044500
C	3.59093400	-5.23201300	0.63341200
H	3.99503400	-6.24063600	0.77235400
C	3.31985000	-4.42217700	1.74176700
H	3.52318000	-4.80317600	2.74849800
C	2.79520100	-3.12206200	1.59243600
C	2.70145400	-2.89076800	-2.28441900
H	1.85924200	-2.17187500	-2.27481600
C	3.97234500	-2.10234800	-2.68350100
H	4.17546900	-1.28093000	-1.97924900
H	3.86047900	-1.66757400	-3.69258400
H	4.85357500	-2.76862700	-2.69297100
C	2.41046400	-3.97006000	-3.34585700
H	3.28376200	-4.62545900	-3.51323300
H	2.18238900	-3.49379800	-4.31512200
H	1.55706100	-4.61028000	-3.06739900
C	2.55515800	-2.26236600	2.83590300
H	1.88992900	-1.43195100	2.53900600
C	3.88086000	-1.65723700	3.35875100
H	4.58666000	-2.45574200	3.65021300
H	3.69701000	-1.02624600	4.24613600
H	4.37675100	-1.03350500	2.59798500
C	1.86104900	-3.03745800	3.97718600
H	2.52260900	-3.80337900	4.41934200
H	0.94926900	-3.54798100	3.62613300
H	1.58035800	-2.34629300	4.79106400
C	3.20816500	2.06340000	0.59297000
C	4.26446100	2.49148600	-0.25197900
C	5.03965000	3.59498700	0.16509200
H	5.86526000	3.93594500	-0.46998500
C	4.77033800	4.26321000	1.36228300
H	5.38247000	5.12009200	1.66394000
C	3.71066800	3.83551200	2.17833100
H	3.50916100	4.36432800	3.11427800

C	2.91195100	2.73754400	1.81652600
C	4.58176200	1.81441500	-1.58554300
H	3.80533900	1.05038200	-1.76536900
C	5.95390800	1.10319100	-1.54299900
H	6.76988900	1.82614400	-1.36551500
H	6.16112100	0.59154500	-2.49940800
H	5.99904700	0.35169400	-0.73592400
C	4.52230600	2.81128200	-2.76501700
H	3.53716900	3.30302700	-2.81964200
H	4.69987600	2.28694700	-3.72071900
H	5.29023200	3.59930300	-2.67157300
C	1.74795900	2.24884600	2.68382800
H	1.91769200	1.16458000	2.85342800
C	0.38883000	2.37139900	1.93599400
H	0.19991800	3.44633400	1.70710600
H	-0.42242800	2.05602400	2.62065600
C	1.69098600	2.91770300	4.07166200
H	2.63665800	2.80754100	4.63309100
H	0.88666500	2.45738700	4.67058400
H	1.46178900	3.99585000	3.99361400
C	-2.66066200	0.75704500	-0.57593000
H	-3.69257500	0.98406400	-0.88201000
C	-3.19658700	-1.57810300	-0.78178000
C	-2.87761200	-2.50747800	-1.82648700
C	-3.72819600	-3.60284900	-2.05653200
H	-3.48846300	-4.31418900	-2.85141400
C	-4.89199100	-3.79516100	-1.29922100
H	-5.54404900	-4.65277700	-1.49482800
C	-5.21648500	-2.86898000	-0.30712100
H	-6.13680200	-3.00520000	0.27144300
C	-4.39399200	-1.75668300	-0.02217600
C	-1.67890600	-2.27488700	-2.75275800
H	-0.78281600	-1.99363600	-2.13440900
C	-1.93717000	-1.08247800	-3.70102400
H	-2.79637500	-1.30734900	-4.35740400
H	-1.05483300	-0.89321100	-4.33591200
H	-2.15801800	-0.16332500	-3.13817100
C	-1.25343800	-3.51088500	-3.56492800
H	-1.07055100	-4.39267000	-2.92700500
H	-0.32700100	-3.29162400	-4.11834300
H	-2.02087100	-3.78331100	-4.31075800
C	-4.88238900	-0.78364800	1.05546300
H	-4.07466400	-0.06011200	1.26274600
C	-5.24175100	-1.47720800	2.39008700
H	-6.10940700	-2.15123000	2.28284100

H	-5.50749100	-0.72143400	3.14913600
H	-4.40420700	-2.07422400	2.78662900
C	-6.11457800	-0.00177100	0.53914700
H	-5.88719600	0.56366800	-0.38026300
H	-6.47618100	0.70972800	1.29991700
H	-6.94141500	-0.69428600	0.30168300
C	-2.21102700	3.12210800	-0.59626500
C	-3.05179700	3.84216200	0.29287600
C	-3.34026200	5.19038600	-0.01204600
H	-3.98684600	5.76107400	0.66406100
C	-2.81686800	5.81165000	-1.14818800
H	-3.05694100	6.85842400	-1.36389400
C	-1.97516800	5.09081300	-2.00912000
H	-1.56352900	5.58406000	-2.89421400
C	-1.65006300	3.74880800	-1.75050600
C	-3.62921300	3.24025300	1.57435500
H	-3.35524700	2.17064900	1.59809900
C	-5.17035900	3.34786900	1.60914600
H	-5.49610300	4.40148500	1.66553800
H	-5.57660500	2.83295700	2.49796600
H	-5.63140300	2.90676300	0.71072500
C	-3.02430500	3.90604300	2.83217000
H	-1.92737700	3.80775400	2.85076700
H	-3.42924500	3.44125400	3.74913700
H	-3.26946100	4.98246200	2.86597200
C	-0.68582000	2.96038300	-2.63608900
H	-1.19531700	2.01225200	-2.90647800
C	0.58485700	2.56980500	-1.83128800
H	1.08513500	3.49846300	-1.46969200
H	1.30678600	2.07782700	-2.51203900
C	-0.32926500	3.67126300	-3.95617400
H	-1.22471300	3.93432600	-4.54835300
H	0.30098800	3.00848300	-4.57320300
H	0.24852500	4.59596000	-3.77729600
C	-0.79416200	-4.11125100	0.76977500
H	0.15894600	-4.60894600	1.02408300
H	-0.92059900	-4.09320800	-0.32493300
C	-1.99509600	-4.70117100	1.51172300
H	-2.92865300	-4.46659300	0.97208500
H	-1.91802600	-5.79488100	1.62259400
C	-1.94485900	-3.95254800	2.85986900
H	-1.19487700	-4.40914300	3.52951500
H	-2.91335700	-3.95040900	3.38598300
C	-1.51400000	-2.54078600	2.44431800
H	-2.36803400	-1.89523700	2.18108700

H	-0.88217700	-2.02275200	3.18297900
N	1.95290000	-1.33028400	0.09354100
N	2.32749700	0.98007600	0.23675300
N	-2.24184100	-0.53558800	-0.50576300
N	-1.79505300	1.76124500	-0.34353900
O	-0.71274500	-2.72020000	1.22930100
H	-0.00536500	-0.15858000	1.36776600
H	0.20333500	0.03715400	-1.57108600
W	-0.15302300	-0.94988600	-0.16718800
W	0.27707800	1.38449400	-0.01990400

[1]5

Electronic Energy (BP86)	−2917.949505
Zero-point correction	1.284904
Thermal correction to Energy	1.362489
Thermal correction to Enthalpy	1.363433
Thermal correction to Gibbs Free Energy	1.171641

W	-1.13082200	0.10441200	-0.17673100
W	1.14362300	-0.13118600	-0.15606200
N	-0.93872400	2.19758300	0.36367200
N	1.40522200	1.94767500	0.27571500
N	-1.40655900	-1.96236100	0.29892400
N	0.93019300	-2.18034300	0.55384600
C	0.30715200	2.68707500	0.53462200
H	0.43391100	3.71930300	0.89911600
C	-2.03272500	3.04195400	0.81768700
C	-2.88544100	3.66432600	-0.14479600
C	-4.00884600	4.37661800	0.31729700
H	-4.67572600	4.84621500	-0.41206900
C	-4.27680300	4.51613400	1.68433600
H	-5.15615100	5.07621800	2.02011900
C	-3.39635700	3.96158500	2.61766200
H	-3.58432100	4.10847000	3.68679800
C	-2.26588100	3.22055000	2.21492400
C	-2.52709800	3.68748300	-1.63064300
H	-1.97764600	2.75693900	-1.86103600
C	-1.57218300	4.87607400	-1.89180900
H	-2.08184000	5.83611800	-1.69242400
H	-1.23507400	4.87839400	-2.94320600
H	-0.68092900	4.82911600	-1.24789400
C	-3.73525600	3.78675700	-2.58297500
H	-4.48472400	3.00429200	-2.38723800
H	-3.39178900	3.66957700	-3.62481600
H	-4.23099300	4.77248400	-2.51788800
C	2.68710400	2.63111500	0.32940900

C	3.59669400	2.34396600	1.38973300
C	4.86586100	2.95527400	1.38554000
H	5.56376700	2.73481100	2.20089400
C	5.24722500	3.83534800	0.36902600
H	6.23917200	4.29927600	0.37925200
C	4.34420400	4.11801600	-0.65999900
H	4.64040300	4.80571000	-1.45940200
C	3.06222700	3.53505500	-0.70990700
C	2.15864500	3.90530200	-1.88730500
H	1.19284000	3.38758000	-1.76333200
C	2.75528500	3.43832700	-3.23485300
H	3.71728900	3.93959000	-3.44345200
H	2.06365300	3.68306900	-4.05981500
H	2.92704500	2.35120100	-3.24077600
C	1.90072700	5.43025000	-1.91810500
H	1.46263000	5.79456900	-0.97297000
H	1.21082000	5.69042600	-2.73836400
H	2.83794800	5.99003900	-2.08388500
C	-0.32206800	-2.66961100	0.67128400
H	-0.46426000	-3.68155800	1.08590600
C	-2.67408100	-2.66505700	0.29451400
C	-3.70354000	-2.26891100	1.19747800
C	-4.93038900	-2.95867600	1.17104500
H	-5.72035500	-2.66768200	1.87130300
C	-5.15781700	-4.01057000	0.27829200
H	-6.12005100	-4.53322100	0.27270100
C	-4.14329200	-4.38629900	-0.60585900
H	-4.31957000	-5.20755800	-1.30900700
C	-2.89525800	-3.73257500	-0.62545800
C	1.95862000	-2.88107800	1.30543500
C	1.88588900	-2.93665300	2.73532300
C	2.96457500	-3.51631500	3.43209200
H	2.93335100	-3.56576000	4.52386900
C	4.07714800	-4.03607000	2.76092400
H	4.90738300	-4.47043800	3.32797300
C	4.10693700	-4.02539800	1.36349600
H	4.95933800	-4.46821600	0.84093000
C	3.05381100	-3.47033000	0.61001800
C	-3.51407400	-1.17794500	2.25209100
H	-2.59644500	-0.60310700	1.99362800
C	-3.28444400	-1.81403000	3.64320200
H	-4.16873700	-2.40364000	3.94314100
H	-3.11557200	-1.04234700	4.41460500
H	-2.41778500	-2.49591600	3.63814200
C	-4.68876100	-0.17724500	2.29584700

H	-4.90571900	0.22893300	1.29556400
H	-4.45856800	0.66763100	2.96611500
H	-5.60983400	-0.65380800	2.67374100
C	-1.84935700	-4.21615100	-1.63301800
H	-0.96602800	-3.55876900	-1.56366600
C	-2.36676800	-4.12286300	-3.08603200
H	-3.20989100	-4.81442000	-3.26190100
H	-1.56318000	-4.39181700	-3.79373800
H	-2.71121300	-3.10341900	-3.31901400
C	-1.41053500	-5.66496300	-1.31463400
H	-0.99289900	-5.75744500	-0.29702900
H	-0.64445400	-6.00750800	-2.03107300
H	-2.26612300	-6.35994500	-1.38224200
C	0.67839900	-2.40514100	3.52596600
H	-0.23669000	-2.73670200	3.00355600
C	0.57318800	-2.96661700	4.95902400
H	0.62293500	-4.06814700	4.97276900
H	-0.38906400	-2.66332100	5.40525900
H	1.37147500	-2.57884600	5.61628500
C	0.62101900	-0.86550200	3.56933100
H	1.51919700	-0.44753000	4.05375500
H	-0.26472000	-0.52587900	4.13348200
H	0.54632600	-0.43239800	2.55083100
C	3.04615200	-3.60358100	-0.91152300
H	2.57403200	-2.69604700	-1.32833600
C	4.44545800	-3.74233500	-1.53615700
H	5.11530000	-2.92131200	-1.23398800
H	4.36331400	-3.71285200	-2.63418900
H	4.92222000	-4.70307000	-1.26919900
C	2.16694500	-4.80899500	-1.32036800
H	2.58943000	-5.75166700	-0.92821900
H	2.11071500	-4.88957300	-2.42022100
H	1.14140400	-4.71013400	-0.93367200
C	-1.54782300	0.20404700	-2.15034500
C	-0.66895000	0.27746300	-3.25613100
H	-1.11964300	0.45371800	-4.24523800
C	0.72334300	0.15520900	-3.24536400
H	1.20699100	0.27266900	-4.22711000
C	-3.01271000	0.07096100	-2.41360200
C	1.58296300	-0.09104100	-2.14271900
C	-3.99896400	0.52264800	-1.49485200
H	-3.69071700	1.08645000	-0.60352200
C	-5.37015500	0.33051500	-1.72310400
H	-6.09577300	0.70162000	-0.99081000
C	-5.81293300	-0.30106000	-2.89612500

H	-6.88260100	-0.44249100	-3.08152200
C	-4.86103900	-0.74101100	-3.83145700
H	-5.18727000	-1.23543100	-4.75350800
C	-3.49139600	-0.56969300	-3.59062500
H	-2.76593000	-0.94542800	-4.31902100
C	3.02196800	-0.33395700	-2.49104000
C	3.37362100	-1.01772000	-3.68948100
H	2.57581100	-1.42009700	-4.32210300
C	4.71011500	-1.18782100	-4.07513000
H	4.93798800	-1.70562400	-5.01399300
C	5.75444000	-0.71238400	-3.26340100
H	6.79847300	-0.84496700	-3.56578700
C	5.43722700	-0.06758800	-2.05744200
H	6.23336500	0.31902600	-1.41199200
C	4.09747300	0.12269200	-1.68364600
H	3.88446000	0.69199700	-0.77247400
C	3.25776300	1.40047200	2.54052300
H	2.22522800	1.04222900	2.38046600
C	3.28007400	2.13443400	3.90062000
H	4.29329800	2.50202300	4.14054900
H	2.97438400	1.45443200	4.71564100
H	2.60044200	3.00265000	3.90157000
C	4.19178000	0.16963800	2.56581300
H	4.15371100	-0.39293800	1.61703700
H	3.90938900	-0.52523000	3.37481200
H	5.24098200	0.47079700	2.73248500
C	-1.30296800	2.71740900	3.29559200
H	-0.60297200	2.00177500	2.82919800
C	-0.47141800	3.89377700	3.86357600
H	0.09949800	4.41321100	3.07604300
H	0.24226900	3.53562500	4.62620100
H	-1.12975400	4.64128800	4.34052400
C	-2.01164000	1.97991400	4.45145600
H	-2.68893300	2.64599700	5.01359400
H	-1.26757900	1.59368800	5.16986500
H	-2.60723900	1.12934600	4.08335200

[1]12

Electronic Energy (BP86)	−3838.495614
Zero-point correction	1.288105
Thermal correction to Energy	1.369412
Thermal correction to Enthalpy	1.370356
Thermal correction to Gibbs Free Energy	1.169304

C	1.52453600	-0.03148300	1.91561900
C	0.06620100	0.36554200	1.84804800

H	-0.14096400	1.40915700	2.13090400
C	-1.03902200	-0.56937800	2.09243600
H	-0.85279500	-1.56776100	2.52015100
C	-2.24528400	-0.10930600	1.54722100
C	2.25322700	-0.16529000	3.16826600
C	3.67335000	-0.15607200	3.18953600
H	4.21038800	-0.02575600	2.24579900
C	4.37613900	-0.28265200	4.39308800
H	5.47141400	-0.26600000	4.38261900
C	3.68614300	-0.41419800	5.61182200
H	4.23878600	-0.51191200	6.55182600
C	2.28078200	-0.40652900	5.61248800
H	1.73216000	-0.49809900	6.55626800
C	1.57187600	-0.28039600	4.41111100
H	0.47715100	-0.27438100	4.42134100
C	0.16206500	-2.69156300	-0.71151600
H	0.16666700	-3.75116400	-1.01426800
C	2.41397700	-2.86216000	-1.26840200
C	2.30754900	-3.25002500	-2.64047200
C	3.28788600	-4.10731900	-3.17942700
H	3.20803000	-4.40290700	-4.23118200
C	4.35249400	-4.58118000	-2.41155700
H	5.09400900	-5.26034500	-2.84555000
C	4.46814700	-4.15963800	-1.08436400
H	5.30984500	-4.51606400	-0.48207700
C	3.53155900	-3.28892300	-0.49436600
C	1.22444700	-2.74666500	-3.59852600
H	0.50443400	-2.13230200	-3.03567500
C	1.83909300	-1.81974900	-4.67299000
H	2.55862300	-2.36331700	-5.31098800
H	2.36951300	-0.97519100	-4.20443200
H	1.04708400	-1.41243300	-5.32586400
C	0.44133200	-3.90549400	-4.25594100
H	-0.35082400	-3.50907000	-4.91443500
H	-0.03565400	-4.55777900	-3.50375100
H	1.09646500	-4.54175000	-4.87658400
C	3.76778000	-2.86532600	0.95381700
H	3.09289000	-2.01909100	1.17374800
C	3.41918000	-3.99541100	1.94762600
H	2.36923800	-4.31137400	1.84146000
H	3.57372900	-3.65625500	2.98672700
H	4.05580900	-4.88235600	1.77779700
C	5.22035100	-2.38715900	1.17216800
H	5.95032100	-3.19394800	0.98765100
H	5.35830200	-2.06112900	2.21603800

H	5.46860100	-1.54733800	0.50163900
C	-2.10989800	-3.07935300	-0.17752700
C	-3.35588400	-2.93654600	-0.86674500
C	-4.39201000	-3.84868700	-0.60170500
H	-5.34185700	-3.74215100	-1.13227700
C	-4.23437800	-4.89648900	0.31165200
H	-5.05316300	-5.59966500	0.49636400
C	-3.02036400	-5.02926100	0.98363300
H	-2.89317900	-5.84449200	1.70412200
C	-1.94755600	-4.13664500	0.77457700
C	-3.56765500	-1.87956800	-1.94795600
H	-3.03100800	-0.95764400	-1.63138800
C	-5.04170200	-1.48566800	-2.16805900
H	-5.09857500	-0.60934300	-2.83504800
H	-5.54740500	-1.23498000	-1.22168700
H	-5.60925900	-2.29627700	-2.65843700
C	-2.93844300	-2.33928400	-3.28132900
H	-3.44886500	-3.25075300	-3.64062200
H	-1.86822800	-2.56381000	-3.16518700
H	-3.03926800	-1.55686400	-4.05293200
C	-0.68453600	-4.38927300	1.60952500
H	0.03425300	-3.57000400	1.43459900
C	-0.98520200	-4.40517600	3.12737900
H	-1.61542200	-5.26576700	3.41080000
H	-1.51364800	-3.49154000	3.44850900
H	-0.04545400	-4.47908300	3.70144900
C	-0.00606400	-5.71774000	1.19592400
H	0.88161700	-5.91822500	1.81983000
H	0.31676200	-5.70684500	0.14133400
H	-0.70014600	-6.56711900	1.32039800
C	0.04255000	2.86554100	-0.33724100
H	0.01910500	3.96210500	-0.45361200
C	2.37748700	3.16438800	-0.52295000
C	2.71194900	3.49804300	-1.86736700
C	3.81003200	4.35181200	-2.09172400
H	4.07076000	4.61838900	-3.12150200
C	4.57246400	4.85824000	-1.03606000
H	5.42531800	5.51618400	-1.23488000
C	4.23341400	4.52037900	0.27781300
H	4.82356000	4.92475100	1.10593900
C	3.14071500	3.67821800	0.56335900
C	1.91579300	3.00982600	-3.07852900
H	1.22366500	2.21930100	-2.74332900
C	1.07480100	4.16600900	-3.66763900
H	0.39172800	4.60090300	-2.91888000

H	0.46785300	3.81188800	-4.51907000
H	1.72596700	4.98062200	-4.03220300
C	2.81103200	2.39676100	-4.17796700
H	3.47262600	3.15146500	-4.63915100
H	2.18180800	1.97819200	-4.98261200
H	3.43976800	1.58828400	-3.77397700
C	2.76603600	3.41625800	2.02066700
H	2.20164000	2.46800900	2.05435800
C	1.84506600	4.54287800	2.54249900
H	2.37385800	5.51283800	2.53054300
H	1.52659900	4.34105400	3.58086300
H	0.94182900	4.65101900	1.92123500
C	3.98632600	3.26872600	2.95290700
H	4.71707000	2.54530900	2.55738900
H	3.66187100	2.91451100	3.94542400
H	4.50689600	4.23143500	3.10343900
C	-2.37318300	2.95808100	-0.20309100
C	-3.17046700	3.13758400	-1.37483200
C	-4.48350800	3.63305500	-1.23063900
H	-5.10113900	3.75590400	-2.12707100
C	-4.99860300	3.99994900	0.01534500
H	-6.02486600	4.37137700	0.10608500
C	-4.16409300	3.94099500	1.13549200
H	-4.53522600	4.29715700	2.10264300
C	-2.84814800	3.44476600	1.05513200
C	-2.63413300	2.95747800	-2.79610600
H	-1.61049800	2.55642000	-2.72904800
C	-3.46772800	1.97257600	-3.64162800
H	-3.49395300	0.97319700	-3.18025000
H	-3.02737300	1.86565900	-4.64803200
H	-4.50751500	2.32345700	-3.76900900
C	-2.56838500	4.33465000	-3.50169100
H	-3.57714300	4.76299600	-3.63827300
H	-2.10778600	4.23432500	-4.49960200
H	-1.97485100	5.06197200	-2.92308100
C	-1.97323800	3.56902200	2.30416600
H	-0.99159000	3.11713000	2.08323300
C	-1.73699200	5.06671500	2.62105600
H	-1.29750700	5.60249000	1.76267000
H	-1.05660600	5.17976600	3.48230500
H	-2.68671500	5.57009900	2.87320400
C	-2.55364300	2.85408500	3.54337700
H	-3.51590000	3.29917200	3.85190500
H	-1.85916600	2.94874900	4.39653000
H	-2.72425300	1.78392700	3.35139300

Cl	3.39316100	0.17444900	-1.32800000
Cl	-0.69535000	0.14492500	-2.52637800
N	1.24673000	2.27220800	-0.26744800
N	-1.12548300	2.21035100	-0.27217600
N	1.34793300	-2.06293900	-0.66535600
N	-1.03323200	-2.14135100	-0.42535800
W	1.42615100	0.07481800	0.00488500
W	-1.06597300	0.03729900	-0.14672200
C	-3.62540200	-0.40527800	1.88840700
C	-3.95619800	-1.15288300	3.05155900
C	-4.68435100	0.12329000	1.10416600
C	-5.29131100	-1.39526500	3.39055100
H	-3.15030200	-1.53341800	3.68793200
C	-6.01941700	-0.11147700	1.45434400
H	-4.44059200	0.74542900	0.23642700
C	-6.32768000	-0.87707300	2.59257300
H	-5.52869000	-1.97996800	4.28555000
H	-6.82361400	0.31359600	0.84489700
H	-7.37242100	-1.05617900	2.86739400

[1]20

Electronic Energy (BP86)	−3801.820100
Zero-point correction	1.320750
Thermal correction to Energy	1.403202
Thermal correction to Enthalpy	1.404146
Thermal correction to Gibbs Free Energy	1.201195

C	-0.05991700	-0.11187700	1.54467000
C	1.04404000	-0.76975200	2.14557500
H	0.95856500	-1.86856800	2.19201100
C	1.78286100	-0.23248000	3.33618500
C	1.72795500	1.14200200	3.65559100
H	1.21607600	1.81037100	2.96091900
C	2.30120800	1.66029200	4.82326700
H	2.23252700	2.73251200	5.03611800
C	2.95893400	0.80010500	5.71531500
H	3.41120300	1.18603800	6.63466600
C	3.02346500	-0.56729500	5.41612500
H	3.52851400	-1.24913600	6.11046700
C	2.44758400	-1.10705800	4.24788900
C	2.54573000	-2.59816600	4.02022100
H	1.55366900	-3.07475900	3.91801100
H	3.06052600	-3.08677500	4.86320800
H	3.11012100	-2.83206200	3.10311000
C	-0.79489600	-2.72508500	-0.34757500
H	-1.09700700	-3.78322500	-0.41197000

C	1.18841000	-3.48665500	-1.35172300
C	2.21346900	-4.27156600	-0.75884000
C	2.81012900	-5.29816600	-1.51697600
H	3.59102000	-5.91210700	-1.05504400
C	2.43036700	-5.54777100	-2.83780300
H	2.90527400	-6.35209900	-3.40968700
C	1.44532100	-4.74793200	-3.42234500
H	1.15820400	-4.92289300	-4.46481900
C	0.81338300	-3.70827200	-2.71233100
C	-0.22057300	-2.86857600	-3.46566800
H	-0.57225100	-2.05544600	-2.80995400
C	0.38444600	-2.19789100	-4.72040000
H	1.26418800	-1.59117000	-4.45762700
H	-0.36572500	-1.53701700	-5.18828600
H	0.69093000	-2.94471900	-5.47462000
C	-1.44024500	-3.72767200	-3.86494700
H	-1.15258800	-4.52513200	-4.57337900
H	-2.20669600	-3.10395900	-4.35626400
H	-1.90238000	-4.21460000	-2.99065700
C	2.71257000	-4.06121100	0.66623100
H	2.15967100	-3.20401000	1.08775500
C	2.43033300	-5.29829200	1.54928200
H	1.35273000	-5.53133500	1.58495400
H	2.78247800	-5.13659100	2.58292900
H	2.95035400	-6.19081500	1.15946500
C	4.21699700	-3.70367400	0.67072700
H	4.82695700	-4.53679300	0.28011000
H	4.57285400	-3.49057000	1.69454200
H	4.41470300	-2.81998800	0.04198400
C	-2.97438700	-2.31399300	0.51887500
C	-3.00173500	-2.77319900	1.87572800
C	-4.24275500	-3.08816900	2.46505700
H	-4.26139200	-3.43897100	3.50268600
C	-5.44146100	-2.96989500	1.75555000
H	-6.39820400	-3.19980700	2.23658300
C	-5.39305700	-2.61108700	0.40567200
H	-6.31908600	-2.59682100	-0.17947800
C	-4.17809700	-2.31257200	-0.24766800
C	-1.73963400	-3.03545600	2.69937300
H	-0.88607900	-2.61397100	2.14961300
C	-1.50785900	-4.56017100	2.83497000
H	-2.33134700	-5.03961800	3.39316200
H	-0.56868000	-4.76542200	3.37863000
H	-1.44773600	-5.05114100	1.84882500
C	-1.75461000	-2.36683400	4.09045400

H	-1.85158100	-1.27276100	4.00991000
H	-0.81222700	-2.57826900	4.62483800
H	-2.57868300	-2.74232500	4.72217000
C	-4.21877400	-2.16839900	-1.76979600
H	-3.20514300	-1.91598600	-2.11908300
C	-5.17191700	-1.05946900	-2.26103200
H	-6.21665200	-1.25116100	-1.95699400
H	-5.15126000	-1.00462800	-3.36295400
H	-4.87167300	-0.07266600	-1.87741900
C	-4.63825300	-3.52346000	-2.39404600
H	-3.99143100	-4.34963700	-2.05517500
H	-4.58225700	-3.47474700	-3.49497900
H	-5.67669400	-3.78281100	-2.12095400
C	0.89815900	2.62737100	-0.47313600
H	1.23919100	3.66487600	-0.61733600
C	3.15084900	2.04547400	-0.98750600
C	4.27506300	1.94646600	-0.11876300
C	5.55811900	2.22937000	-0.62484900
H	6.41900100	2.15318900	0.04815600
C	5.75243000	2.60631900	-1.95511600
H	6.75874200	2.81236800	-2.33500900
C	4.64088000	2.72911400	-2.79113200
H	4.78609300	3.03455900	-3.83272200
C	3.33193400	2.46118300	-2.34243800
C	4.16221800	1.59229500	1.35899700
H	3.09886100	1.41732500	1.58572800
C	4.63916000	2.76475900	2.24644500
H	5.71189500	2.97630400	2.09109600
H	4.49251900	2.52045800	3.31202500
H	4.08208700	3.69107900	2.02425100
C	4.92881800	0.29766600	1.70415500
H	4.57381300	-0.54877600	1.09243000
H	4.78682300	0.04132600	2.76776300
H	6.01258700	0.40930000	1.52246500
C	2.20552300	2.66727300	-3.35921100
H	1.24298800	2.39343800	-2.89425400
C	2.14623900	4.15654800	-3.77404700
H	1.98764100	4.82132500	-2.90716600
H	1.33269900	4.32941700	-4.49710300
H	3.09014600	4.46776200	-4.25449800
C	2.36665800	1.78085300	-4.61547300
H	3.28381100	2.03603500	-5.17522700
H	1.50978300	1.93156900	-5.29511200
H	2.41361700	0.71618600	-4.34366300
C	-1.26296800	3.58184200	-0.08041500

C	-2.00083400	4.15370000	-1.15922100
C	-2.89645700	5.20905600	-0.87398000
H	-3.46842500	5.64470600	-1.69987300
C	-3.06382400	5.71726800	0.41461500
H	-3.76965400	6.53316600	0.60375800
C	-2.29423400	5.18716800	1.45568100
H	-2.38778300	5.60356100	2.46359000
C	-1.38922800	4.13302900	1.23384200
C	-1.84652500	3.76910100	-2.63121800
H	-1.16317400	2.90694000	-2.69753700
C	-3.18697500	3.35809400	-3.28184100
H	-3.66369600	2.52790600	-2.74033300
H	-3.01309700	3.02214000	-4.31841700
H	-3.89335000	4.20668700	-3.32196700
C	-1.24392500	4.95658700	-3.41965800
H	-1.94004600	5.81401200	-3.43650200
H	-1.05296200	4.66485900	-4.46700200
H	-0.29717600	5.30677400	-2.98032900
C	-0.51152700	3.66591300	2.38910800
H	-0.21841000	2.62693300	2.16049500
C	0.76246500	4.54344400	2.47320400
H	1.34713500	4.51892900	1.53944600
H	1.42176300	4.20851000	3.29280700
H	0.48916000	5.59582100	2.66743000
C	-1.21843200	3.65792600	3.76061400
H	-1.38907700	4.67899300	4.14548500
H	-0.59095600	3.13330000	4.50135200
H	-2.19500500	3.14815600	3.71808700
C	-4.17774200	1.76825700	0.48235300
H	-4.28363000	1.38654500	-0.54405100
H	-3.92176600	2.84159400	0.45029600
C	-5.37156600	1.49143200	1.39517800
H	-5.81433200	0.50666800	1.16508700
H	-6.15383500	2.26211300	1.30209200
C	-3.39162400	0.72634400	2.49532300
H	-2.54021900	1.04036300	3.11986400
H	-3.51730300	-0.36621100	2.56710600
Cl	2.60076600	-0.83182800	-2.11163300
Cl	-1.98260000	0.37559800	-2.31788000
N	0.51105200	-2.43277000	-0.59459800
N	-1.71826700	-1.81697200	-0.03103800
N	1.83005600	1.64557200	-0.50103100
N	-0.40743600	2.40467500	-0.26219900
O	-3.05891400	1.04360900	1.10106800
W	1.21262600	-0.41217300	-0.21241200

W	-1.04511700	0.32511200	-0.05551200
C	-4.70085800	1.46849700	2.78333400
H	-4.50284400	2.49904400	3.12815100
H	-5.30338800	0.95641600	3.55077300

[1]20'

Electronic Energy (BP86)	−3569.361226
Zero-point correction	1.205030
Thermal correction to Energy	1.281286
Thermal correction to Enthalpy	1.282231
Thermal correction to Gibbs Free Energy	1.091053

C	0.22692000	0.05564800	1.62972100
C	0.18237700	-1.25902800	2.15262600
H	1.16333600	-1.74564500	2.28270000
C	-0.80026200	-1.67756800	3.20351000
C	-1.97368700	-0.92107000	3.42664000
H	-2.19283800	-0.08694800	2.75348300
C	-2.84538900	-1.20618000	4.48453100
H	-3.73948100	-0.59285000	4.63421200
C	-2.56087900	-2.27528900	5.34845800
H	-3.23004200	-2.51086400	6.18239800
C	-1.40340200	-3.03764200	5.13784300
H	-1.17311900	-3.86973300	5.81338100
C	-0.50970700	-2.76133100	4.08350900
C	0.73810000	-3.60473700	3.94272900
H	1.65929000	-3.00982000	4.08422300
H	0.74950200	-4.41030000	4.69432400
H	0.80628700	-4.07473200	2.94759700
C	2.84508500	-0.16543400	-0.31131300
H	3.93550400	-0.31123300	-0.36443700
C	2.75756700	-2.25992400	-1.34693500
C	2.98722000	-3.55418300	-0.80669500
C	3.73126200	-4.47908900	-1.56432500
H	3.92452400	-5.47278200	-1.14578400
C	4.22632500	-4.15903000	-2.83164600
H	4.80710100	-4.89241700	-3.40098200
C	3.95615500	-2.89810400	-3.36972000
H	4.31729000	-2.65191200	-4.37406500
C	3.21677400	-1.93275200	-2.65856500
C	2.91572400	-0.60601500	-3.36261000
H	2.25908300	0.00238700	-2.71895500
C	2.14819100	-0.83359900	-4.68512000
H	1.23070400	-1.41749600	-4.51246900
H	1.86260800	0.13663500	-5.12678100

H	2.76313300	-1.37261400	-5.42742600
C	4.20879100	0.19883200	-3.62237600
H	4.89457800	-0.35197600	-4.29049100
H	3.97376000	1.16323000	-4.10357500
H	4.75627700	0.40786400	-2.68741400
C	2.46788300	-4.00115200	0.55651500
H	1.88082500	-3.16685000	0.97871600
C	3.62796500	-4.30751400	1.53097700
H	4.28604700	-3.43250600	1.66388200
H	3.24174400	-4.60363100	2.52200000
H	4.25119100	-5.13873800	1.15723000
C	1.52280600	-5.21686800	0.41407200
H	2.05542100	-6.09135500	0.00165600
H	1.11578300	-5.51750400	1.39597100
H	0.68052600	-4.98572500	-0.25814800
C	3.32575200	1.96161400	0.64487600
C	3.83417100	1.64927100	1.94530800
C	4.68542500	2.57668900	2.57669000
H	5.07962500	2.34041200	3.57107800
C	5.03590900	3.78442500	1.96664300
H	5.69313600	4.49451500	2.47958100
C	4.55078900	4.06535200	0.68706800
H	4.84246900	4.99834600	0.19325800
C	3.70618800	3.17234400	-0.00354800
C	3.52735900	0.34869100	2.69111600
H	2.78610500	-0.21728800	2.10486800
C	4.79577800	-0.52703000	2.82066600
H	5.57588000	-0.01542400	3.41133000
H	4.56171000	-1.47848600	3.32985600
H	5.23097200	-0.76333000	1.83460000
C	2.89708200	0.60621400	4.07731800
H	1.97447600	1.20141900	3.98627400
H	2.63988300	-0.34986400	4.56642700
H	3.58843900	1.14461000	4.74871900
C	3.29103400	3.54235100	-1.42582900
H	2.66959000	2.72639300	-1.82750300
C	2.44037000	4.83256900	-1.44972700
H	3.01796600	5.70079500	-1.08443400
H	2.11076600	5.05282400	-2.47929000
H	1.53701400	4.73258600	-0.82680400
C	4.52640000	3.70116800	-2.34332900
H	5.15903000	2.79856500	-2.33781700
H	4.20491400	3.88784300	-3.38281800
H	5.15627000	4.55400400	-2.03356500
C	-2.70475900	0.54725500	-0.20058600

H	-3.79352900	0.69667700	-0.27037700
C	-3.15008100	-1.61310800	-1.12848900
C	-3.78185000	-2.63284700	-0.36504300
C	-4.61466700	-3.56053200	-1.02092600
H	-5.10366700	-4.34182100	-0.42889000
C	-4.82548200	-3.50304600	-2.39991800
H	-5.46876900	-4.23770000	-2.89586400
C	-4.21003800	-2.48904400	-3.13785500
H	-4.37801900	-2.43521500	-4.21864300
C	-3.37136800	-1.52867500	-2.53748000
C	-3.62165100	-2.76023300	1.14404400
H	-2.94686800	-1.95917200	1.48107200
C	-4.97124100	-2.56391700	1.87088700
H	-5.69341100	-3.35518700	1.60267900
H	-4.82453400	-2.59664100	2.96389300
H	-5.43150100	-1.59423100	1.61342100
C	-2.97212800	-4.10433600	1.54019200
H	-1.99819900	-4.23760000	1.03915000
H	-2.81044900	-4.14407400	2.63069500
H	-3.61097300	-4.96021100	1.25846200
C	-2.79562800	-0.44425600	-3.45297600
H	-2.14206800	0.21799200	-2.85747100
C	-3.94898800	0.39631300	-4.05068200
H	-4.64610900	0.74858200	-3.27593600
H	-3.55244200	1.27179000	-4.59343300
H	-4.53513700	-0.20241100	-4.77012900
C	-1.94570300	-1.01052200	-4.61334900
H	-2.55588700	-1.63894400	-5.28635900
H	-1.53705700	-0.17987700	-5.21454000
H	-1.10908800	-1.61838600	-4.24238600
C	-2.47393000	2.88894000	0.45520500
C	-3.16851300	3.64980500	-0.53680100
C	-3.55614900	4.96899400	-0.22450300
H	-4.08323100	5.55118300	-0.98735000
C	-3.28379700	5.55575800	1.01160800
H	-3.58921800	6.58665100	1.21868500
C	-2.62613800	4.79850500	1.98208400
H	-2.42524000	5.23699200	2.96535700
C	-2.21822100	3.47314700	1.73821700
C	-3.56174200	3.14433400	-1.92466100
H	-3.13075700	2.14165900	-2.07410600
C	-3.00186400	4.04200800	-3.05141700
H	-1.90506800	4.11301700	-2.98670800
H	-3.26295300	3.61701600	-4.03613400
H	-3.42270400	5.06194500	-3.01206000

C	-5.10439400	3.04495900	-2.02262900
H	-5.57520400	4.02220300	-1.81839700
H	-5.41705700	2.73201100	-3.03241000
H	-5.51867000	2.32524600	-1.29438400
C	-1.55819000	2.73661100	2.90395500
H	-1.34133800	1.70705300	2.58284000
C	-2.50005400	2.65406300	4.12704400
H	-3.47352500	2.21191900	3.85422700
H	-2.04796100	2.02826000	4.91557900
H	-2.69815500	3.65001700	4.56061800
C	-0.20723400	3.37719900	3.29264700
H	-0.32908600	4.42994600	3.60309600
H	0.25421100	2.82824100	4.13157200
H	0.50789100	3.36351700	2.44929600
Cl	-0.26571900	-2.62882000	-2.00772900
Cl	0.14037800	2.34459200	-2.07116100
N	2.05083000	-1.23591300	-0.58130300
N	2.37916300	1.04214300	0.02239400
N	-2.21005900	-0.68941200	-0.47668700
N	-1.92336400	1.57133900	0.16980100
W	-0.09024000	-1.07075100	-0.20648200
W	0.21042500	1.24493800	0.11520900

¹21

Electronic Energy (BP86)	−3817.662934
Zero-point correction	1.293185
Thermal correction to Energy	1.375108
Thermal correction to Enthalpy	1.376052
Thermal correction to Gibbs Free Energy	1.173780

C	-0.07025500	0.08093400	1.53150300
C	1.11664000	0.53832000	2.17340000
H	1.18935700	1.63544100	2.27092100
C	1.73421400	-0.14800300	3.35782700
C	1.47887300	-1.51320400	3.61254700
H	0.89594300	-2.07239600	2.87852500
C	1.94390900	-2.15769500	4.76577600
H	1.71961900	-3.21760600	4.92684200
C	2.69343500	-1.43899500	5.70888800
H	3.06539000	-1.92495800	6.61675900
C	2.95320000	-0.08172600	5.47652800
H	3.52874700	0.49203900	6.21253500
C	2.48651500	0.58464100	4.32511300
C	2.78522100	2.05916200	4.17705800
H	1.86578300	2.66693200	4.09038200
H	3.34497800	2.43155400	5.05022500

H	3.39064400	2.26374200	3.27961700
C	-0.36244100	2.82034100	-0.30681200
H	-0.50342400	3.91133800	-0.36831600
C	1.75150800	3.31058200	-1.20392000
C	2.86008400	3.91567300	-0.55186400
C	3.63119600	4.86101100	-1.25609300
H	4.47983400	5.33386100	-0.74989200
C	3.34170500	5.20423300	-2.57874400
H	3.95345000	5.94273000	-3.10781300
C	2.26805800	4.58200900	-3.21984500
H	2.04791000	4.82902800	-4.26402400
C	1.46141600	3.63020800	-2.56547900
C	0.34635600	2.97615700	-3.38399200
H	-0.17897300	2.23834900	-2.75657700
C	0.91158400	2.20335600	-4.59759400
H	1.65092400	1.45433100	-4.27602800
H	0.09381700	1.68119600	-5.12400200
H	1.39917100	2.88237900	-5.31992100
C	-0.68467000	4.02430900	-3.85585000
H	-0.22808700	4.75467600	-4.54746700
H	-1.51516800	3.53305600	-4.39107300
H	-1.10840800	4.59098600	-3.01041300
C	3.27842900	3.58733600	0.87704600
H	2.56291700	2.84504700	1.27009600
C	3.22323100	4.83637900	1.78608700
H	2.21460200	5.28225400	1.79952200
H	3.50115100	4.58188500	2.82372100
H	3.92632600	5.61373800	1.43943500
C	4.68601200	2.94649600	0.89462200
H	5.44803700	3.65210700	0.51991400
H	4.98109200	2.65936500	1.91977700
H	4.71700400	2.04787200	0.25704900
C	-2.61623800	2.73750200	0.45605000
C	-2.64800500	3.15686400	1.82603100
C	-3.84178400	3.68920000	2.34930600
H	-3.86424500	4.00996700	3.39587500
C	-4.99098600	3.82265800	1.56451500
H	-5.91455100	4.22518100	1.99426100
C	-4.92965700	3.47256600	0.21479600
H	-5.80850800	3.62696300	-0.42060800
C	-3.75631700	2.95275500	-0.37291500
C	-1.41506300	3.13571700	2.72977000
H	-0.70672900	2.40838500	2.30542700
C	-0.73833600	4.52880800	2.74524900
H	-1.42620600	5.28848600	3.15738600

H	0.16937000	4.51748400	3.37470700
H	-0.44742600	4.85956400	1.73486900
C	-1.70625500	2.69716800	4.18188100
H	-2.27970300	1.75732700	4.22295100
H	-0.75915100	2.54076500	4.72631900
H	-2.27624200	3.46131800	4.73920300
C	-3.76285200	2.76855800	-1.89083900
H	-2.78169100	2.37133200	-2.19526000
C	-4.83868400	1.76931400	-2.36758000
H	-5.85673700	2.11984700	-2.11928700
H	-4.78417900	1.64581700	-3.46277800
H	-4.69444900	0.77747200	-1.91304700
C	-3.98164000	4.13559900	-2.58463200
H	-3.24503600	4.88617700	-2.25401600
H	-3.89432800	4.03018700	-3.67962600
H	-4.98653200	4.53909400	-2.36701400
C	0.50244800	-2.70518300	-0.56315300
H	0.68622400	-3.77539100	-0.74791100
C	2.82110900	-2.47493500	-1.02183400
C	3.91266600	-2.65940800	-0.12725900
C	5.12698300	-3.16687400	-0.62835700
H	5.96442000	-3.30849100	0.06338600
C	5.28220000	-3.49366600	-1.97708500
H	6.23613200	-3.88159800	-2.34999300
C	4.19971200	-3.32404100	-2.84306300
H	4.31452800	-3.58105000	-3.90147000
C	2.96132700	-2.81737500	-2.40096200
C	3.83036000	-2.36393700	1.36505000
H	2.80971000	-2.01341900	1.58395900
C	4.07412400	-3.63977700	2.20238000
H	5.09730900	-4.02899600	2.05636100
H	3.94690100	-3.42029700	3.27591800
H	3.37012800	-4.44396000	1.92764400
C	4.80337800	-1.23989700	1.78207200
H	4.61499600	-0.31931000	1.20466400
H	4.68317600	-1.00883300	2.85429300
H	5.85454000	-1.53351400	1.61147400
C	1.85550200	-2.68526300	-3.45093600
H	0.96602800	-2.23087400	-2.98213700
C	1.46866200	-4.08201200	-3.98866400
H	1.14847300	-4.76142700	-3.18063700
H	0.64842300	-4.00512500	-4.72141200
H	2.32555900	-4.55867500	-4.49628800
C	2.26517600	-1.76952000	-4.62712600
H	3.10744600	-2.19852400	-5.19837600

H	1.41782000	-1.64919900	-5.32437200
H	2.56544300	-0.77457900	-4.26686100
C	-1.79825800	-3.32018700	-0.23353900
C	-2.60607500	-3.73127900	-1.33540800
C	-3.68866400	-4.60407100	-1.08310500
H	-4.31561900	-4.91329700	-1.92609300
C	-3.96762700	-5.09816400	0.19107600
H	-4.81573200	-5.77206800	0.35328700
C	-3.12600100	-4.74655400	1.25100500
H	-3.30811800	-5.16124000	2.24727800
C	-2.04178900	-3.87042700	1.06496300
C	-2.33636700	-3.38860800	-2.80215400
H	-1.50008100	-2.67205600	-2.84687200
C	-3.55239900	-2.73970800	-3.50121800
H	-3.87658300	-1.82145300	-2.98874800
H	-3.28723500	-2.46246500	-4.53592800
H	-4.40826400	-3.43622400	-3.55379200
C	-1.94276000	-4.67627600	-3.56704500
H	-2.78365200	-5.39181500	-3.59363400
H	-1.67617900	-4.43860400	-4.61125900
H	-1.08616300	-5.18854400	-3.10142700
C	-1.10760600	-3.59942700	2.23840400
H	-0.63993000	-2.61857500	2.04641000
C	-0.00182800	-4.68317100	2.29269300
H	0.58338100	-4.72833900	1.35998600
H	0.70028000	-4.49216300	3.12291100
H	-0.45139000	-5.67938800	2.45220100
C	-1.81208500	-3.51715900	3.60845700
H	-2.15865500	-4.50678500	3.95570200
H	-1.10627700	-3.13701600	4.36684200
H	-2.68293100	-2.84243300	3.58159600
Cl	2.91765200	0.53059200	-1.88305300
Cl	-1.85959300	-0.03540600	-2.42055000
N	0.89846900	2.34530300	-0.50588500
N	-1.42351600	2.05667400	-0.04535400
N	1.57600700	-1.87761300	-0.53159700
N	-0.75832500	-2.29441900	-0.36840900
W	1.29749800	0.24015800	-0.14429500
W	-1.07813400	-0.15513700	-0.10552200
N	-3.05510500	-0.53224500	1.10093500
C	-3.16465800	-0.34593400	2.43923500
C	-4.16512800	-0.91633400	0.41751100
C	-4.36871000	-0.51693600	3.12991700
H	-2.23514900	-0.05951000	2.93967500
C	-5.40391900	-1.11487900	1.03622300

H	-4.03308500	-1.06855900	-0.65821500
C	-5.51363500	-0.90736400	2.41840100
H	-4.40007700	-0.34635000	4.20981000
H	-6.26231400	-1.42660700	0.43518800
H	-6.46960600	-1.05038500	2.93168700

[1]22

Electronic Energy (BP86)	−3704.542922
Zero-point correction	1.297066
Thermal correction to Energy	1.377989
Thermal correction to Enthalpy	1.378934
Thermal correction to Gibbs Free Energy	1.179870

W	-1.00624400	-0.08446600	-0.09513000
W	1.37249800	0.10370000	0.24532400
Cl	-1.27027400	-0.06853600	-2.53545700
Cl	3.35845700	0.19359900	-1.10730200
N	-3.16752300	-0.21709000	0.80868800
N	-0.86026200	-2.25477900	-0.18253100
N	1.51243300	-2.04960200	-0.10631500
N	-1.14047500	2.14050000	-0.21603200
N	1.24656300	2.20686600	-0.21919500
C	-0.26140000	0.16069700	1.68000800
C	-3.45659700	-0.00482500	2.11609100
H	-2.58750500	0.19893100	2.74862900
C	-4.76063000	-0.04852700	2.61900600
H	-4.93534900	0.13469400	3.68319800
C	-5.82105500	-0.32893700	1.74321800
H	-6.85208500	-0.37062900	2.10781200
C	-5.52844900	-0.55749400	0.39159000
H	-6.31486900	-0.78496600	-0.33299600
C	-4.19721300	-0.49235100	-0.03359200
H	-3.92441600	-0.66887800	-1.07861700
C	0.37028400	-2.77998000	-0.21919900
H	0.47306700	-3.86888400	-0.35112400
C	-2.00631800	-3.16588900	-0.09987900
C	-2.43731600	-3.60005300	1.19474600
C	-3.63193500	-4.33394000	1.31095900
H	-3.95869300	-4.66272900	2.30191500
C	-4.40659600	-4.65284100	0.19165900
H	-5.34287900	-5.21033000	0.30257200
C	-3.94588400	-4.28296600	-1.07104900
H	-4.51926400	-4.57528100	-1.95710200
C	-2.74346500	-3.56370300	-1.25547100
C	-2.28277800	-3.38049700	-2.70335300

H	-1.34215800	-2.80614300	-2.70364000
C	-3.30393900	-2.61136700	-3.57085900
H	-4.26569100	-3.15058200	-3.63955100
H	-2.91473200	-2.49475400	-4.59697800
H	-3.49212900	-1.60061600	-3.17796400
C	-2.02851000	-4.77332100	-3.33398700
H	-1.32571500	-5.37587100	-2.73506700
H	-1.61309500	-4.66990200	-4.35040200
H	-2.96815100	-5.34764000	-3.41459600
C	-1.59939200	-3.36972000	2.44922600
H	-0.99158900	-2.46501900	2.27780200
C	-0.64313800	-4.56725400	2.67299000
H	-0.00791900	-4.40014100	3.56018400
H	0.01772100	-4.73448100	1.80669300
H	-1.21923500	-5.49590800	2.83485100
C	-2.44012100	-3.14647800	3.72476800
H	-2.95583100	-4.06865600	4.04580100
H	-3.20289000	-2.36353700	3.58315700
H	-1.78715400	-2.84569700	4.56203800
C	2.69321000	-2.79925300	-0.56162700
C	2.82548200	-3.12261400	-1.94695900
C	3.92236600	-3.90727800	-2.35554300
H	4.02385500	-4.15614100	-3.41722900
C	4.88091100	-4.36528400	-1.44995500
H	5.71792000	-4.98448100	-1.78977600
C	4.76609200	-4.00214200	-0.10672500
H	5.52418700	-4.33863600	0.60843400
C	3.70067800	-3.20597200	0.35772200
C	3.69758000	-2.83156900	1.83533600
H	2.89788400	-2.08571500	1.98219500
C	5.03076500	-2.17240500	2.25542700
H	5.88126700	-2.86706900	2.14587000
H	4.99428500	-1.86925400	3.31603000
H	5.24357500	-1.28071500	1.64276100
C	3.38964000	-4.05489100	2.72784000
H	2.41984300	-4.51231500	2.47044200
H	3.36162300	-3.76721200	3.79363400
H	4.16608500	-4.83138700	2.60922800
C	1.87381500	-2.64372900	-3.04547700
H	1.07945600	-2.02628000	-2.59317900
C	1.21375600	-3.84025200	-3.76264200
H	1.96431100	-4.44531100	-4.30161700
H	0.47869200	-3.48637600	-4.50551600
H	0.69763200	-4.50914700	-3.05612500
C	2.60398700	-1.75938100	-4.08256300

H	3.11024900	-0.91402200	-3.59424900
H	1.87893900	-1.35919900	-4.81226500
H	3.35964800	-2.33846700	-4.64294500
C	0.01841800	2.79641800	-0.28635300
H	-0.00457100	3.89100600	-0.40649400
C	-2.34581200	2.94882400	-0.02730200
C	-2.63796000	3.46141300	1.27880800
C	-3.85878600	4.12995300	1.49223200
H	-4.07961200	4.52371200	2.48920400
C	-4.79046300	4.30459800	0.46528800
H	-5.74130800	4.81339100	0.65680800
C	-4.47205400	3.85190400	-0.81515200
H	-5.17397700	4.02891000	-1.63713000
C	-3.25447500	3.19658700	-1.09947300
C	-2.96627900	2.89663000	-2.57149900
H	-1.97340700	2.42505200	-2.64313600
C	-2.95324100	4.21182800	-3.38907700
H	-3.95641600	4.67258200	-3.42425800
H	-2.64316100	4.01367100	-4.42948700
H	-2.26184300	4.95589600	-2.96189500
C	-3.99301100	1.92290900	-3.18977900
H	-4.00973800	0.96262500	-2.65295900
H	-3.73187100	1.71098200	-4.24072900
H	-5.01271300	2.34831600	-3.17441700
C	-1.65144900	3.38890100	2.44556000
H	-0.96335200	2.54998100	2.25206100
C	-0.82358500	4.69493300	2.53477500
H	-0.25570400	4.89297300	1.61130800
H	-0.10610900	4.64510100	3.37293100
H	-1.48712900	5.56095500	2.70796100
C	-2.32587100	3.13084000	3.81115100
H	-2.89970600	4.00693400	4.16109400
H	-1.55901400	2.92412000	4.57620500
H	-3.01696900	2.27312200	3.77428000
C	2.31145100	3.09665000	-0.68940200
C	3.32798000	3.55786500	0.19395100
C	4.26157200	4.49941200	-0.28097000
H	5.02881400	4.87348900	0.40559300
C	4.23815300	4.95758100	-1.60064900
H	4.97296600	5.69208400	-1.94718800
C	3.28047100	4.44370600	-2.47754400
H	3.28188500	4.76424200	-3.52491900
C	2.31316600	3.50980500	-2.05593100
C	1.36528000	2.95344400	-3.12083200
H	0.67155300	2.23723400	-2.65018500

C	2.14952600	2.17226300	-4.20042600
H	2.82254000	2.83784900	-4.77006300
H	1.44890500	1.70730700	-4.91566300
H	2.75864000	1.37733200	-3.74419000
C	0.53089000	4.07408200	-3.77600600
H	-0.03614200	4.65236800	-3.02791700
H	-0.18672700	3.64874700	-4.49773100
H	1.17261500	4.78512600	-4.32604300
C	3.48739300	3.07140800	1.63110200
H	2.74335700	2.27572700	1.79742500
C	3.22279100	4.19864300	2.65411900
H	3.95517600	5.01782400	2.54217400
H	3.30848400	3.81814800	3.68812500
H	2.21686600	4.63279900	2.52920600
C	4.88349200	2.44348800	1.84760900
H	5.05626800	1.62122700	1.13418100
H	4.97408400	2.04303500	2.87213600
H	5.68790900	3.18728400	1.71222000
C	0.86905300	0.57561300	2.43506600
H	0.98000800	1.67857000	2.48539100
C	1.31996000	-0.00774400	3.80299700
C	0.97710700	-1.49571200	3.96794500
H	-0.11421600	-1.63260600	4.01466200
H	1.35628000	-2.10712500	3.13592600
H	1.40765300	-1.88647200	4.90698000
C	2.83052100	0.23565800	3.99707700
H	3.05835000	1.31510100	3.98086000
H	3.16537200	-0.16230600	4.97209600
H	3.42341900	-0.24206900	3.20269700
C	0.56189000	0.78152100	4.90383500
H	0.86124200	0.42577300	5.90643100
H	0.78536900	1.86189800	4.84983000
H	-0.52976300	0.65093700	4.80759100

[1]24

Electronic Energy (BP86)	−3764.693334
Zero-point correction	1.318523
Thermal correction to Energy	1.401071
Thermal correction to Enthalpy	1.402015
Thermal correction to Gibbs Free Energy	1.199930

W	-0.48309300	-1.21303100	0.13993200
Cl	-1.37826900	-3.17783200	-0.94150300
Cl	-0.05245600	0.87092400	-2.91762900
N	-2.50373000	-0.48824000	-0.14204100

N	-1.75402600	1.74226900	-0.35941700
N	1.42223500	-1.98671900	-0.50156600
N	2.37233500	0.19211500	-0.76764800
C	-0.18475100	-0.75520800	2.15294900
C	0.75706700	1.59945300	1.31461300
C	-0.60176000	-1.33304800	3.31085000
H	-1.35705100	-2.12535600	3.21152100
C	-0.23898900	-1.07171100	4.76851800
C	0.26705100	-2.40797000	5.37683800
H	-0.47758400	-3.21379100	5.25453400
H	0.45887800	-2.28596000	6.45789900
H	1.20533600	-2.73349400	4.89673700
C	-1.53992700	-0.65851800	5.51016700
H	-1.93951500	0.29016700	5.11408200
H	-1.33964100	-0.52511600	6.58841700
H	-2.32380600	-1.42880700	5.40466100
C	0.82890900	0.01605800	4.96024600
H	1.77516600	-0.26351600	4.46587300
H	1.04093100	0.15686100	6.03477800
H	0.49505300	0.98296100	4.55118700
C	-2.72854200	0.84912800	-0.25618100
H	-3.77437100	1.19473100	-0.25930500
C	-3.71511200	-1.27163200	-0.41442900
C	-4.42813900	-1.89241900	0.64692400
C	-5.60818700	-2.60087300	0.34436000
H	-6.16406100	-3.07613700	1.16001000
C	-6.08524000	-2.70514200	-0.96450400
H	-7.00661900	-3.25799600	-1.17654900
C	-5.36924700	-2.09778100	-1.99972100
H	-5.73210800	-2.18378700	-3.02960300
C	-4.18201000	-1.38036200	-1.75754700
C	-3.45672900	-0.77319200	-2.95822200
H	-2.50624700	-0.33267300	-2.61593000
C	-3.09828800	-1.84303600	-4.01391700
H	-2.51840400	-2.66176300	-3.56033500
H	-2.49162500	-1.38926800	-4.81645600
H	-4.00091300	-2.27624300	-4.48097300
C	-4.29903900	0.35087300	-3.60162900
H	-5.24806800	-0.04270400	-4.00801600
H	-3.74508000	0.81891600	-4.43356300
H	-4.55327700	1.13986400	-2.87429600
C	-3.98350600	-1.83829700	2.10611000
H	-3.08333500	-1.20272300	2.16201500
C	-3.61617500	-3.25372800	2.61023400
H	-4.48637900	-3.93097700	2.55497700

H	-3.29046300	-3.22580100	3.66605200
H	-2.81129300	-3.69990000	2.00221200
C	-5.06161400	-1.20666600	3.01602700
H	-5.35553100	-0.20508000	2.66272400
H	-4.68519100	-1.10877800	4.04979400
H	-5.97305700	-1.82880800	3.05443400
C	-2.08881200	3.14716100	-0.16495100
C	-2.18777900	4.03349900	-1.27575700
C	-2.35681200	5.41081800	-1.01990900
H	-2.42290200	6.09898700	-1.86960100
C	-2.47235400	5.90878400	0.27977600
H	-2.60269500	6.98239100	0.45302400
C	-2.45711400	5.01618600	1.35686100
H	-2.59305000	5.39914000	2.37293200
C	-2.27085000	3.63337100	1.16791300
C	-2.36137100	2.70915100	2.38396500
H	-1.86318800	1.75651300	2.12941200
C	-3.84751200	2.41401700	2.70597400
H	-4.37966400	3.34685600	2.96460200
H	-3.93099400	1.72506700	3.56437100
H	-4.37526700	1.95927600	1.85137000
C	-1.67218300	3.27571400	3.64420200
H	-0.62958100	3.56872200	3.44755400
H	-1.67112000	2.51419500	4.44336200
H	-2.20691900	4.15442800	4.04600900
C	-2.23404700	3.56818000	-2.73107900
H	-2.10791700	2.47379200	-2.74793300
C	-3.61020900	3.90924900	-3.35291800
H	-4.44220400	3.49853000	-2.75672100
H	-3.68324200	3.49418200	-4.37279200
H	-3.76060300	5.00113000	-3.42422200
C	-1.10192600	4.17689300	-3.58464200
H	-1.16299800	5.27965000	-3.61538500
H	-1.16564200	3.80697400	-4.62237100
H	-0.11348900	3.89335000	-3.19121000
C	2.44418900	-1.14026300	-0.79755500
H	3.40198800	-1.60479400	-1.07454700
C	1.73002700	-3.36295100	-0.87826700
C	1.80838400	-4.37393000	0.11866200
C	2.17590600	-5.67511500	-0.27426900
H	2.24642000	-6.45876000	0.48811900
C	2.45493600	-5.98586100	-1.60879200
H	2.74433100	-7.00379000	-1.89079500
C	2.34806600	-4.98679700	-2.58101300
H	2.54458100	-5.23235000	-3.63033100

C	1.97768100	-3.66896000	-2.24928900
C	1.81896700	-2.65575100	-3.38775400
H	1.43682700	-1.70718900	-2.97476400
C	3.16608700	-2.35987500	-4.08701800
H	3.92147100	-1.96541100	-3.38546700
H	3.02656800	-1.61417100	-4.88882200
H	3.58746000	-3.27126900	-4.54746600
C	0.76951600	-3.13554700	-4.41654800
H	1.10050700	-4.04855700	-4.94270200
H	0.59990100	-2.35265700	-5.17566800
H	-0.19097900	-3.35260200	-3.92346000
C	1.51364200	-4.12094800	1.59583800
H	1.23085600	-3.05964400	1.71260000
C	2.75691800	-4.36880800	2.47911200
H	3.09052900	-5.41968000	2.41723800
H	2.52976500	-4.15574400	3.53879700
H	3.60382100	-3.73234300	2.17375400
C	0.32093500	-4.97545300	2.08172200
H	-0.57041300	-4.79715500	1.45862200
H	0.07528200	-4.73360800	3.13051500
H	0.55512300	-6.05362200	2.03515500
C	3.65728500	0.86719100	-0.70514600
C	4.71050800	0.36841300	0.13878100
C	5.96196300	1.01810200	0.11452600
H	6.76581500	0.62840600	0.74806400
C	6.20242800	2.14345200	-0.67196400
H	7.18617700	2.62411400	-0.67353900
C	5.15715800	2.65613900	-1.44306200
H	5.32849200	3.54834000	-2.05221400
C	3.88706600	2.05252600	-1.47328900
C	2.84076200	2.67077400	-2.39303400
H	1.84275300	2.34337000	-2.04669900
C	3.00503900	2.14412300	-3.83629500
H	3.98980500	2.44080700	-4.24081600
H	2.21884800	2.55492800	-4.49185000
H	2.93146100	1.04579000	-3.87125300
C	2.84104500	4.21413900	-2.36781400
H	2.79216100	4.60340600	-1.33716800
H	1.96958300	4.59683500	-2.92530600
H	3.73798400	4.63913200	-2.85154400
C	4.61067100	-0.80390200	1.13057200
H	3.58023000	-1.19686600	1.13512500
C	5.54778300	-1.96826100	0.73152400
H	5.30409900	-2.37570900	-0.26440700
H	5.47919900	-2.79402100	1.46053000

H	6.60028900	-1.63728200	0.70269300
C	4.90892500	-0.34692600	2.57821100
H	5.95821700	-0.02758500	2.69948800
H	4.73228400	-1.17738100	3.28436100
H	4.26887300	0.50250900	2.86818000
C	0.84530800	0.19469200	1.71852000
H	1.87774000	-0.19468600	1.82624600
C	1.42752200	2.75585400	1.87939900
C	2.25878200	2.68224600	3.03211200
C	1.28148500	4.02777500	1.25148800
C	2.92395500	3.81519700	3.51720700
H	2.37736300	1.72210100	3.53944800
C	1.93913600	5.15785100	1.74643400
H	0.62207100	4.11360400	0.38155000
C	2.76741700	5.05808900	2.88014100
H	3.56429500	3.72974400	4.40176800
H	1.80234500	6.12430500	1.25014500
H	3.28057000	5.94431700	3.26748300

^{2}27

Electronic Energy (UBP86)	−3304.410159
Zero-point correction	1.315109
Thermal correction to Energy	1.396592
Thermal correction to Enthalpy	1.397536
Thermal correction to Gibbs Free Energy	1.195883

W	-1.13352700	-0.29444600	0.21863100
W	0.94971100	0.30075600	-0.43146400
Cl	0.71247700	-0.03703800	-2.89541400
N	-1.90143800	1.58053900	-0.28747700
N	0.27619400	2.36010500	-0.70681100
N	-0.76024600	-2.33235400	-0.28050900
N	1.56443700	-1.84955900	-0.35597200
C	-0.59495100	-0.03840700	2.27187000
C	1.72727200	0.46188500	1.55726700
C	-1.61287800	-0.12497400	3.19881900
H	-2.62006000	-0.34362200	2.80898500
C	-1.58663300	0.03092500	4.73114300
C	-3.03139200	-0.12311000	5.26580500
H	-3.70147500	0.64579300	4.84246800
H	-3.05457600	-0.02170700	6.36489400
H	-3.44752400	-1.11424400	5.01093500
C	-1.05668000	1.42527700	5.15812000
H	-0.02357600	1.59822000	4.81907200
H	-1.07475100	1.51583900	6.25952600

H	-1.68596600	2.22840900	4.74011700
C	-0.71673800	-1.08299700	5.37471600
H	-1.09875400	-2.08325400	5.10676400
H	-0.73839600	-0.99353900	6.47627400
H	0.33354200	-1.02741600	5.04813300
C	-1.04360600	2.56527400	-0.67300100
H	-1.45636700	3.54228800	-0.96900600
C	-3.32838500	1.81977700	-0.36074200
C	-3.96311400	2.76793700	0.49207600
C	-5.35811100	2.93928300	0.38019800
H	-5.85402100	3.67253600	1.02546700
C	-6.11984400	2.19299500	-0.52226900
H	-7.20300200	2.34030900	-0.58647500
C	-5.48617500	1.25382800	-1.34422200
H	-6.08241000	0.67344600	-2.05457200
C	-4.09513500	1.04833700	-1.28668200
C	-3.44810400	0.07747000	-2.27732400
H	-2.47362900	-0.26979600	-1.85755100
C	-4.29224200	-1.18342400	-2.54581500
H	-4.58248300	-1.69145000	-1.61159300
H	-3.71840000	-1.90128500	-3.15285700
H	-5.21159600	-0.94371500	-3.10806800
C	-3.10317100	0.80738500	-3.59503800
H	-4.02653600	1.17569000	-4.07681900
H	-2.58818000	0.12866400	-4.29533700
H	-2.44215700	1.66999900	-3.41506600
C	-3.21069300	3.61539500	1.51770700
H	-2.16242100	3.27261400	1.54543100
C	-3.78392700	3.43499700	2.94092000
H	-4.82366800	3.79900500	3.01464000
H	-3.18241600	4.00574500	3.66962300
H	-3.77450600	2.37435000	3.24089800
C	-3.22729400	5.11024500	1.12002300
H	-2.77964500	5.27782000	0.12525500
H	-2.66677100	5.71614900	1.85230700
H	-4.26109100	5.49670700	1.08377800
C	1.11556600	3.48567300	-1.10746600
C	1.20490100	3.87402500	-2.47639600
C	2.11029400	4.90016800	-2.81921300
H	2.19105500	5.20227800	-3.86871500
C	2.89702600	5.53839100	-1.85756600
H	3.60309400	6.32240500	-2.15199100
C	2.75048900	5.19020800	-0.51008200
H	3.33139000	5.72147000	0.24972100
C	1.85506200	4.18281700	-0.10338400

C	1.59494800	3.96873400	1.38828500
H	1.26967000	2.92411700	1.53117800
C	0.43596800	4.89196600	1.83666800
H	0.72219300	5.95399600	1.73099800
H	0.17941500	4.70966400	2.89544100
H	-0.46973400	4.73095000	1.23125800
C	2.82061700	4.21469200	2.29107300
H	3.70195300	3.64538100	1.95738900
H	2.58800400	3.90356000	3.32361000
H	3.09179900	5.28524900	2.33242200
C	0.31392000	3.30047200	-3.57956400
H	-0.17463100	2.39330800	-3.18907500
C	-0.78172300	4.32318900	-3.96846300
H	-1.40296300	4.61482400	-3.10359500
H	-1.44948700	3.90134900	-4.74036900
H	-0.33552800	5.24738400	-4.37720100
C	1.10873400	2.86853500	-4.83049700
H	1.58009100	3.72773600	-5.34011700
H	0.43206500	2.38566000	-5.55641700
H	1.89289200	2.14188700	-4.56763000
C	0.54828100	-2.70757000	-0.39165800
H	0.75932200	-3.78495600	-0.48056000
C	-1.73585300	-3.31893600	-0.69760100
C	-2.80281300	-3.67190200	0.18219200
C	-3.75817500	-4.61216200	-0.24790200
H	-4.57161800	-4.88919800	0.43176900
C	-3.67857500	-5.21358700	-1.50755800
H	-4.42679700	-5.94958300	-1.81997400
C	-2.62741000	-4.86638300	-2.36044400
H	-2.56088400	-5.33599500	-3.34785600
C	-1.64878700	-3.91957400	-1.99551500
C	-0.55735600	-3.60701800	-3.02709100
H	0.06886000	-2.77928400	-2.65386000
C	0.35064200	-4.83801800	-3.26077800
H	0.83306300	-5.18060800	-2.32922200
H	1.14640200	-4.59989600	-3.98731300
H	-0.22692800	-5.68880300	-3.66383600
C	-1.14422000	-3.11554300	-4.36940900
H	-1.77740900	-3.88063500	-4.85250800
H	-0.32644400	-2.86558500	-5.06636200
H	-1.74861900	-2.20492900	-4.22931700
C	-2.93840000	-3.10953400	1.59547000
H	-2.11454400	-2.39139000	1.76094600
C	-2.77744500	-4.21901300	2.66000100
H	-3.58606000	-4.96781800	2.58742000

H	-2.81139400	-3.78619200	3.67542200
H	-1.81757700	-4.74871500	2.54357000
C	-4.27590400	-2.35809500	1.78378100
H	-4.37559900	-1.51715100	1.07439600
H	-4.35660200	-1.95275100	2.80738800
H	-5.13976200	-3.02739700	1.62631700
C	2.85881600	-2.45819800	-0.10395700
C	3.02950700	-3.28459700	1.05183200
C	4.27091300	-3.92457900	1.24353400
H	4.40217400	-4.56936500	2.11939600
C	5.33708400	-3.74197700	0.36097600
H	6.29228500	-4.24992800	0.53130400
C	5.17413000	-2.88806300	-0.73452100
H	6.01092600	-2.73002900	-1.42195100
C	3.95489600	-2.23353500	-0.98968800
C	3.83666300	-1.37161900	-2.24242500
H	2.99514500	-0.67198900	-2.10068400
C	3.47465000	-2.23838000	-3.46962800
H	4.27436400	-2.97291600	-3.67586900
H	3.34014900	-1.60582200	-4.36365900
H	2.53541500	-2.78879500	-3.30272700
C	5.09346800	-0.52513700	-2.53280800
H	5.42010700	0.03624800	-1.64186900
H	4.87676000	0.19669100	-3.33897200
H	5.94380400	-1.14265200	-2.87325200
C	1.96327800	-3.48873300	2.13675800
H	1.10939400	-2.82467300	1.92312400
C	1.44634000	-4.94588400	2.17324400
H	0.99221400	-5.25297500	1.21563300
H	0.68653100	-5.06809700	2.96535200
H	2.26750200	-5.65243900	2.38819300
C	2.49767500	-3.08306100	3.52994200
H	3.30267300	-3.75673200	3.87243700
H	1.68617000	-3.13089100	4.27706100
H	2.89966600	-2.05774200	3.51440900
C	0.80605200	0.25168800	2.57641100
H	1.10723900	0.31874900	3.63557300
C	3.15683300	0.73543800	1.85738100
C	3.73942600	0.59480900	3.14531000
C	4.01803600	1.16394000	0.81398200
C	5.09339400	0.87182300	3.37273300
H	3.12135400	0.26096800	3.98507400
C	5.37369600	1.44558500	1.03517100
H	3.60444400	1.30174700	-0.19451400
C	5.92107900	1.30011000	2.32012000

H	5.50823200	0.75033100	4.37960600
H	6.00037600	1.78619500	0.20396000
H	6.97931600	1.51618400	2.49997200

[1]28

Electronic Energy (BP86)	−2844.122589
Zero-point correction	1.309682
Thermal correction to Energy	1.388170
Thermal correction to Enthalpy	1.389114
Thermal correction to Gibbs Free Energy	1.195488

N	1.35420500	-2.17190100	0.25102500
W	-1.07645100	-0.02387100	0.28949100
N	-0.98591000	-2.14047700	0.47611700
C	0.32607700	-0.09052100	-2.01092100
N	1.39620200	2.06256500	0.04383500
C	-1.01567400	-0.17715000	-2.51562900
H	-1.18611700	-0.30864700	-3.59599900
N	-0.91560400	2.16120300	0.54846600
C	-2.04888500	-0.16439000	-1.57714700
C	-3.46578700	-0.29630500	-1.97671500
C	-4.44623200	-0.70463400	-1.03671500
H	-4.13934700	-0.93240000	-0.01031700
C	-5.79103400	-0.85845100	-1.40298900
H	-6.51973500	-1.18731400	-0.65456300
C	-6.20012800	-0.59895200	-2.72108100
H	-7.25042800	-0.71580100	-3.00834300
C	-5.24818200	-0.18155200	-3.66804100
H	-5.55709500	0.03467700	-4.69692500
C	-3.90479600	-0.02935600	-3.30123600
H	-3.18251300	0.32172300	-4.04566900
C	1.89147900	0.10844200	-4.13865200
C	3.39425900	0.44070500	-4.28062700
H	3.63668400	1.40163200	-3.79350500
H	3.67391900	0.51949500	-5.34550600
H	4.02455600	-0.34222300	-3.82433600
C	1.58527000	-1.23583200	-4.85101000
H	2.17285400	-2.06207100	-4.41876700
H	1.83527700	-1.16213000	-5.92517500
H	0.51866600	-1.49934300	-4.76269000
C	1.06502700	1.23366100	-4.81137000
H	-0.01532700	1.02577900	-4.77479000
H	1.35960700	1.33164900	-5.87190000
H	1.23544300	2.20179300	-4.31519100
C	0.17752200	-2.80486900	0.49728200
H	0.18871000	-3.86787700	0.77981100

C	2.53194500	-2.91463400	0.66183100
C	3.45224000	-3.38947700	-0.31680500
C	4.61077600	-4.06414500	0.11492300
H	5.32265000	-4.43447900	-0.62924100
C	4.86066200	-4.29143500	1.47290400
H	5.76569500	-4.82263600	1.78644800
C	3.93579700	-3.85003300	2.42422200
H	4.12115900	-4.04647100	3.48593900
C	2.76530600	-3.15834500	2.05115200
C	1.78820000	-2.73703200	3.15389500
H	1.03907000	-2.06104600	2.70655400
C	2.47824400	-1.95676200	4.29387200
H	3.19662700	-2.58377500	4.85049300
H	1.72683000	-1.59706200	5.01797200
H	3.02438300	-1.08162400	3.90703500
C	1.04674400	-3.96454100	3.73650600
H	0.48901700	-4.51604600	2.96191000
H	0.32813800	-3.65114900	4.51404600
H	1.75880400	-4.67029600	4.20024400
C	3.15456500	-3.27714900	-1.81080700
H	2.47051800	-2.42080700	-1.95157400
C	4.41594900	-3.03700900	-2.66553200
H	5.09047900	-3.91124300	-2.66259600
H	4.99418600	-2.16658700	-2.31014000
H	4.13805400	-2.85797100	-3.71822700
C	2.40784200	-4.54238900	-2.29798400
H	2.15312400	-4.45822600	-3.36948500
H	1.47171400	-4.69539300	-1.73699100
H	3.03495500	-5.44206800	-2.16511800
C	-2.18995900	-2.87455700	0.76989600
C	-2.71007600	-3.84620900	-0.12500600
C	-3.92437800	-4.47549500	0.22273300
H	-4.34120600	-5.23307700	-0.45012600
C	-4.61063300	-4.14454900	1.39451600
H	-5.55575600	-4.64199000	1.63763900
C	-4.08338500	-3.17590100	2.26331500
H	-4.62085600	-2.93128400	3.18387600
C	-2.86905800	-2.52974900	1.97448500
C	-2.24265900	-1.49440400	2.90833500
H	-1.22088000	-1.86288400	3.13747400
C	-2.06249400	-0.11742500	2.21718200
H	-1.62233700	0.58660500	2.94358900
H	-3.06530800	0.30830600	1.96678200
C	-2.98138900	-1.35043800	4.25353300
H	-3.99530300	-0.93313400	4.11725200

H	-2.42882100	-0.65718400	4.90985000
H	-3.07470400	-2.31435200	4.78527000
C	-2.01130100	-4.23191400	-1.42995100
H	-1.17450000	-3.52888900	-1.58362300
C	-2.94571600	-4.10248000	-2.65365000
H	-3.79098100	-4.81118600	-2.59964400
H	-2.38870000	-4.32593800	-3.58068000
H	-3.36003100	-3.08508300	-2.73554500
C	-1.42941300	-5.66256700	-1.34449800
H	-0.71778200	-5.76774400	-0.50734100
H	-0.90196600	-5.92685800	-2.27797500
H	-2.23088700	-6.40691700	-1.18979300
C	0.28810800	2.74893100	0.40920900
H	0.38871100	3.81234900	0.66439800
C	2.62853000	2.79070300	-0.11008100
C	3.79213700	2.30241800	0.56263700
C	5.01480600	2.98231400	0.40988600
H	5.89959300	2.61074800	0.93786600
C	5.11855500	4.12998800	-0.38148300
H	6.07626000	4.65060200	-0.48342800
C	3.98063300	4.60014100	-1.04139400
H	4.05857200	5.49509200	-1.66792300
C	2.73296100	3.95134600	-0.94308600
C	1.57328100	4.53749600	-1.75508200
H	0.74155000	3.81211900	-1.73981100
C	1.95693500	4.76394700	-3.23633300
H	1.06109000	5.02784900	-3.82452600
H	2.41037100	3.86386600	-3.68192200
H	2.67827400	5.59179400	-3.35221300
C	1.07405500	5.87112500	-1.14832800
H	1.88526700	6.62060800	-1.14119100
H	0.72465700	5.75678600	-0.10835000
H	0.23949700	6.28285200	-1.74187700
C	3.77205800	1.11741000	1.53221600
H	2.76616100	0.62057600	1.48740800
C	4.84435100	0.05331200	1.21783200
H	5.86098900	0.45240000	1.37755400
H	4.78024500	-0.29109400	0.17186400
H	4.72340200	-0.82682800	1.87083500
C	3.89632500	1.62685100	2.98767300
H	3.87119000	0.79174300	3.70763800
H	3.08114500	2.32591300	3.23552700
H	4.85308400	2.16144600	3.12145600
C	-1.90363400	3.01672500	1.19011800
C	-1.73428300	3.40610700	2.55589200

C	-2.73012000	4.20511700	3.15379200
H	-2.60919200	4.50989000	4.19893900
C	-3.86438200	4.61510200	2.44802200
H	-4.62842700	5.22889000	2.93721100
C	-4.00604100	4.24724600	1.10585100
H	-4.88076900	4.58997900	0.54488400
C	-3.03856500	3.46125300	0.45022900
C	-3.17142000	3.20442800	-1.05051100
H	-2.61932200	2.27708200	-1.28048700
C	-4.62771800	3.02326200	-1.52142900
H	-5.15268900	2.24708000	-0.94142800
H	-4.64265600	2.71426000	-2.57938100
H	-5.20635200	3.96188500	-1.44815900
C	-2.49979500	4.35188700	-1.84270100
H	-3.00872000	5.31320000	-1.64724600
H	-2.54752500	4.15410700	-2.92835500
H	-1.44074600	4.46805500	-1.56183700
C	-0.50734700	3.04004400	3.40292500
H	0.05065200	2.24618100	2.87532300
C	0.43283100	4.25887100	3.57590600
H	0.79564100	4.64976400	2.61105100
H	1.31362000	3.98910000	4.18584500
H	-0.09120200	5.08358400	4.09077300
C	-0.88137900	2.49549100	4.80193800
H	-1.32391000	3.27943300	5.44112700
H	0.02185900	2.12671900	5.31868100
H	-1.60580300	1.66718000	4.74056300
W	1.28965600	-0.05723700	-0.10216100
H	0.59649300	0.02809100	1.50794600
C	1.53109300	-0.00333200	-2.64504200
H	2.45686400	0.01648900	-1.99496900

^{1}17^{Ph}

Electronic Energy (BP86)	−2586.583369
Zero-point correction	0.519893
Thermal correction to Energy	0.559657
Thermal correction to Enthalpy	0.560601
Thermal correction to Gibbs Free Energy	0.442186

W	-1.22136600	-0.61902200	-0.01211400
W	0.64277500	0.68836500	-0.40367700
Cl	-2.83314200	-1.10052200	-1.71909500
Cl	1.06473200	1.47851100	-2.63164000
N	1.79095000	-1.17101300	-0.94936000
N	-2.41105300	1.18386300	0.32536600

N	-0.48751800	2.47149800	-0.04313000
C	1.15241900	-2.32354000	-0.79196900
H	1.68554000	-3.27087000	-0.96868100
C	-1.79687900	2.37164200	0.24786600
H	-2.36104500	3.28956900	0.47512600
C	1.09692900	0.07166400	1.53175400
N	-0.14071900	-2.38137800	-0.38361600
C	-0.27904000	0.11894300	1.73530900
H	-0.74068100	0.67239600	2.57438100
C	2.21047200	-0.09100700	2.45397400
C	3.52782900	0.28587200	2.08291200
C	1.99783900	-0.60869100	3.75995400
C	4.58502600	0.17881100	2.99427600
H	3.70728200	0.66568300	1.07167000
C	3.06015700	-0.72874600	4.66281800
H	0.98691600	-0.91676300	4.04728400
C	4.35525600	-0.33053700	4.28478000
H	5.59327400	0.48378500	2.69593900
H	2.88141900	-1.13171000	5.66530100
H	5.18540800	-0.42362900	4.99282600
C	0.07680100	3.77850700	-0.16812800
C	-0.55438300	4.78310500	-0.93136900
C	1.31274200	4.05258700	0.45454900
C	0.03848700	6.04839900	-1.05072900
H	-1.48572400	4.55451100	-1.45890700
C	1.90422600	5.31682400	0.31989200
H	1.78836600	3.27308000	1.06061000
C	1.26784800	6.32078600	-0.42852500
H	-0.45563200	6.81930000	-1.65118200
H	2.86206300	5.51912200	0.81024800
H	1.73042800	7.30730900	-0.53215700
C	-3.80943700	1.17392700	0.60733500
C	-4.31226100	0.23049900	1.52805300
C	-4.70420000	2.05590800	-0.03545800
C	-5.68412100	0.17924000	1.81082600
H	-3.61289300	-0.44925600	2.02877100
C	-6.07422000	2.00466300	0.25951600
H	-4.32812400	2.74980300	-0.79382200

C	-6.57033200	1.06969800	1.18243900
H	-6.06028600	-0.55728200	2.52834700
H	-6.75999100	2.68815900	-0.25194900
H	-7.64162500	1.02816400	1.40265500
C	-0.76116400	-3.67247600	-0.35183600
C	-0.66410400	-4.56266100	-1.44130400
C	-1.51789400	-4.03740900	0.78092400
C	-1.30767500	-5.80769100	-1.38537700
H	-0.11746600	-4.25934800	-2.33979700
C	-2.16825600	-5.27915500	0.82302300
H	-1.56907800	-3.34759000	1.63139900
C	-2.06187200	-6.17031600	-0.25741000
H	-1.23302400	-6.49009100	-2.23836600
H	-2.75187400	-5.55351400	1.70782800
H	-2.56845400	-7.13988900	-0.22291300
C	3.20533100	-1.26881500	-1.18431900
C	3.81836600	-0.49800900	-2.19487500
C	4.00009200	-2.13289700	-0.39543100
C	5.19918800	-0.60731800	-2.41593500
H	3.20106900	0.16788700	-2.80218600
C	5.37862200	-2.24025400	-0.63200200
H	3.53731800	-2.69510000	0.42176900
C	5.98516300	-1.47820700	-1.64278200
H	5.66034100	-0.01011600	-3.20958000
H	5.97988600	-2.91319900	-0.01157300
H	7.06195800	-1.55840700	-1.82335900

[1]TS1a

Electronic Energy (BP86)	−2894.966698
Zero-point correction	0.626375
Thermal correction to Energy	0.673670
Thermal correction to Enthalpy	0.674614
Thermal correction to Gibbs Free Energy	0.540580

W	0.93785000	0.01379500	-0.00582300
W	-1.37165600	0.19066900	-0.63150400
Cl	2.29854200	0.39716500	-1.96197800
Cl	-1.78227400	0.27468600	-2.97571000
N	0.85321300	-2.07382600	-0.25873400
N	-1.43973400	-2.02263200	-0.79124600

N	0.94251700	2.22057600	0.18558800
N	-1.32735400	2.32229600	-0.46025000
C	-1.96542200	-0.21005800	1.28913200
C	3.22262900	-0.15962700	1.36748200
C	-0.32423100	-2.69138200	-0.53703300
H	-0.33728800	-3.79285600	-0.55266600
C	-0.16952300	2.90709500	-0.09937200
H	-0.14359100	4.00686600	-0.03174700
C	-0.67038400	0.18111000	1.53908100
H	-0.40555400	0.74897100	2.45432100
C	-3.11679200	-0.45051100	2.14654100
C	-2.95273300	-0.70012200	3.53429800
C	-4.42935400	-0.44053300	1.60989300
C	-4.06473800	-0.91589900	4.35621500
H	-1.94087900	-0.72741300	3.95283900
C	-5.53981800	-0.64720700	2.43685800
H	-4.55829800	-0.26277900	0.53715200
C	-5.36104400	-0.88610500	3.81087800
H	-3.92420700	-1.10906000	5.42508600
H	-6.54780700	-0.62839100	2.00986800
H	-6.23003300	-1.05557100	4.45542100
C	4.53870000	-0.28972000	0.86406900
C	5.15884300	0.76940300	0.13937900
C	5.28003100	-1.48268900	1.10913300
C	6.48064600	0.63718300	-0.29721600
H	4.59110600	1.67920900	-0.06725000
C	6.59775200	-1.59623100	0.65911000
H	4.80153300	-2.30538500	1.64790500
C	7.20759500	-0.53882100	-0.04127900
H	6.94639300	1.46020900	-0.84927400
H	7.15595200	-2.51742300	0.85716300
H	8.24144200	-0.63364800	-0.38913600
C	2.08262800	-0.05460800	1.92550000
H	1.66825400	0.11489900	2.92262600
C	2.10740100	3.04625300	0.38364900
C	2.64780100	3.22421700	1.67230800
C	2.70177100	3.69270700	-0.71925500
C	3.77256900	4.04338600	1.85387800

H	2.17881200	2.72264800	2.52299800
C	3.82708800	4.51108700	-0.52902300
H	2.28472400	3.53067600	-1.71740600
C	4.36668000	4.68739900	0.75554300
H	4.18336100	4.18061500	2.85956600
H	4.28241100	5.00987400	-1.39103000
H	5.24507300	5.32435400	0.90125700
C	-2.49224200	3.16061900	-0.47194200
C	-2.74344000	4.05422900	0.59360600
C	-3.41274400	3.08061800	-1.53882500
C	-3.88660200	4.86729300	0.57968900
H	-2.05405000	4.08798900	1.44379000
C	-4.55672200	3.89199900	-1.53775000
H	-3.20974600	2.39444100	-2.36536000
C	-4.79896900	4.78960800	-0.48437300
H	-4.06953200	5.55325200	1.41357800
H	-5.25917200	3.82613400	-2.37534100
H	-5.69400700	5.41984100	-0.49033400
C	-2.64312400	-2.79724800	-0.89387900
C	-3.55566300	-2.55150400	-1.94159200
C	-2.93772700	-3.79503700	0.06225500
C	-4.73210100	-3.30967600	-2.03528400
H	-3.32018300	-1.78192500	-2.68086100
C	-4.11447200	-4.55066900	-0.04452100
H	-2.25594800	-3.95289300	0.90417900
C	-5.01642700	-4.31265700	-1.09368000
H	-5.42737900	-3.11706400	-2.85914100
H	-4.33160800	-5.31811900	0.70586400
H	-5.93694500	-4.89983700	-1.17291900
C	1.96507000	-2.99061200	-0.11629000
C	2.94675400	-3.06128100	-1.12163800
C	2.03353700	-3.84725100	1.00055100
C	3.98101600	-4.00419700	-1.01468200
H	2.88689300	-2.37642400	-1.97210300
C	3.07391600	-4.78473200	1.10024300
H	1.27022200	-3.77078800	1.78205300
C	4.04578400	-4.87042600	0.08921000
H	4.73884400	-4.06049000	-1.80257200

H	3.12001600	-5.45179000	1.96765900
H	4.85216600	-5.60725700	0.16396200

[1]INT1a

Electronic Energy (BP86)	−2894.985847
Zero-point correction	0.627005
Thermal correction to Energy	0.674751
Thermal correction to Enthalpy	0.675695
Thermal correction to Gibbs Free Energy	0.538708

W	-1.50177500	0.00010500	-0.33815800
Cl	1.66345200	0.00014300	-2.09084700
Cl	-2.43022500	0.00023900	-2.51370300
N	-1.35542500	2.14812000	-0.54131200
N	0.95479700	-2.13633700	-0.03672400
N	-1.35563600	-2.14792700	-0.54155200
C	-0.16977800	2.75374500	-0.42388600
H	-0.11894700	3.83736800	-0.61791200
C	-0.17003700	-2.75364500	-0.42417500
H	-0.11929100	-3.83726700	-0.61823500
C	-2.14545100	-0.00000900	1.51394500
N	0.95499100	2.13634000	-0.03641100
C	-0.73396100	-0.00001900	1.67222000
H	-0.32129100	-0.00011600	2.68772200
C	-3.22406800	-0.00016300	2.49502600
C	-4.57306300	-0.00007400	2.05062600
C	-2.97692500	-0.00045200	3.89216200
C	-5.63009400	-0.00022900	2.96919500
H	-4.77034700	0.00010500	0.97316500
C	-4.03704100	-0.00061300	4.80613500
H	-1.94447000	-0.00055900	4.25765200
C	-5.36735400	-0.00049400	4.35015800
H	-6.66362700	-0.00015300	2.60708600
H	-3.82648300	-0.00083600	5.88087600
H	-6.19401200	-0.00061900	5.06801500
W	1.00153900	-0.00003900	0.30051700
C	2.13999600	-0.00020600	2.00530400
H	2.14282400	-0.00028300	3.10432500
C	3.04370100	-0.00017700	1.00451700
C	4.48831200	-0.00021400	0.85165700

C	5.36010600	-0.00015600	1.97338900
C	5.05247700	-0.00030500	-0.44986700
C	6.74755600	-0.00019100	1.79806600
H	4.92858400	-0.00008300	2.98047000
C	6.44290700	-0.00035200	-0.62128700
H	4.37081600	-0.00033300	-1.30611500
C	7.29131500	-0.00029300	0.49925800
H	7.41144100	-0.00014200	2.66952100
H	6.86895600	-0.00042900	-1.63019400
H	8.37840400	-0.00032600	0.36337400
C	-2.46633900	-2.98540300	-0.90171200
C	-2.41430500	-3.78455400	-2.06224800
C	-3.62602900	-2.98975800	-0.10176200
C	-3.50760100	-4.59437600	-2.40470400
H	-1.52917300	-3.74265700	-2.70487400
C	-4.71536300	-3.79984200	-0.45474200
H	-3.65835600	-2.36655500	0.79659300
C	-4.66076900	-4.60595900	-1.60406100
H	-3.46033700	-5.20755100	-3.31070000
H	-5.60972400	-3.80328700	0.17713900
H	-5.51437200	-5.23440700	-1.87730200
C	2.13860900	-2.96130700	-0.09697600
C	2.78861300	-3.34725900	1.09079500
C	2.62773100	-3.39902700	-1.34260400
C	3.91915500	-4.17450600	1.02872700
H	2.39818800	-2.99725200	2.05091100
C	3.76149000	-4.22591100	-1.39503400
H	2.12813300	-3.06405400	-2.25651700
C	4.40885300	-4.61608600	-0.21252900
H	4.41706900	-4.47869900	1.95530900
H	4.13956000	-4.56170200	-2.36636700
H	5.29329100	-5.25983900	-0.25607400
C	2.13886400	2.96122100	-0.09660500
C	2.78892900	3.34699100	1.09119200
C	2.62796900	3.39906900	-1.34219500
C	3.91951600	4.17418000	1.02918700
H	2.39850600	2.99690000	2.05127700
C	3.76177700	4.22589200	-1.39456100

H	2.12832900	3.06423400	-2.25613500
C	4.40920200	4.61588200	-0.21203100
H	4.41747600	4.47823700	1.95579000
H	4.13983900	4.56177300	-2.36586600
H	5.29367900	5.25958400	-0.25552700
C	-2.46603400	2.98573200	-0.90148900
C	-3.62571000	2.99027800	-0.10152900
C	-2.41387600	3.78485000	-2.06204200
C	-4.71492800	3.80051300	-0.45453200
H	-3.65812000	2.36712500	0.79685500
C	-3.50705600	4.59481800	-2.40452300
H	-1.52874000	3.74280700	-2.70465200
C	-4.66022000	4.60658300	-1.60387800
H	-5.60928100	3.80412500	0.17735800
H	-3.45971200	5.20796000	-3.31053700
H	-5.51373400	5.23514500	-1.87713700

[1]TS2a

Electronic Energy (BP86)	−2894.979053
Zero-point correction	0.627152
Thermal correction to Energy	0.673988
Thermal correction to Enthalpy	0.674932
Thermal correction to Gibbs Free Energy	0.541578

W	-1.47062600	-0.08542400	-0.31833000
Cl	1.92087500	0.89202900	-2.06378800
Cl	-2.53379300	0.09918400	-2.42600000
N	-1.83034900	2.03279600	-0.15242000
N	1.36446500	-1.69235600	-0.55198800
N	-0.94015400	-2.06992100	-0.94038500
C	-0.79503200	2.87899200	-0.15105000
H	-1.00620400	3.95879300	-0.21587600
C	0.34681400	-2.43878000	-0.98512000
H	0.57435800	-3.45007100	-1.35925800
C	-1.90362800	-0.54929400	1.54621400
N	0.48326600	2.50224400	-0.03171200
C	-0.52281800	-0.28604500	1.58459800
H	0.03268900	-0.44616400	2.51260900
C	-2.85431900	-0.98019300	2.56302400
C	-4.21250200	-1.20030900	2.21091300

C	-2.46940200	-1.18734900	3.91320200
C	-5.14390500	-1.61529900	3.17100500
H	-4.51933600	-1.03858500	1.17195300
C	-3.40426800	-1.60256900	4.86863000
H	-1.42874500	-1.01685700	4.20956600
C	-4.74491100	-1.81921400	4.50330600
H	-6.18651000	-1.77995600	2.87898300
H	-3.08730100	-1.75712200	5.90553700
H	-5.47360700	-2.14308400	5.25345000
W	1.04434000	0.38246600	0.12289800
C	2.01127400	0.83030800	1.88657500
H	1.88070500	1.28894300	2.87496800
C	2.95262300	0.20609900	1.17953800
C	4.34072400	-0.19087900	1.38581500
C	4.83602000	-0.50424700	2.67809800
C	5.23957500	-0.20757600	0.29146200
C	6.18437300	-0.83015400	2.86599700
H	4.14628300	-0.49227500	3.52947100
C	6.59412400	-0.50638700	0.48863700
H	4.85445400	0.02682600	-0.70503000
C	7.06885500	-0.82550400	1.77211400
H	6.55209800	-1.07802900	3.86778000
H	7.28109300	-0.50171500	-0.36410900
H	8.12560000	-1.07188500	1.92143700
C	-1.89396200	-3.03591100	-1.40713600
C	-1.77613100	-3.59591000	-2.69609800
C	-2.96888400	-3.41472200	-0.57826100
C	-2.71572000	-4.53851500	-3.14024000
H	-0.96392400	-3.26743500	-3.35243900
C	-3.90658900	-4.35336000	-1.03338200
H	-3.05202900	-2.97964900	0.42187600
C	-3.78385200	-4.92027700	-2.31303800
H	-2.61775100	-4.96447100	-4.14429400
H	-4.73400500	-4.64735800	-0.37901600
H	-4.51892800	-5.65117200	-2.66481500
C	2.64134000	-2.35739400	-0.69506200
C	3.16390000	-3.10211600	0.37885800
C	3.32435400	-2.32339000	-1.92525500

C	4.36562100	-3.80865200	0.22212500
H	2.61828700	-3.12564500	1.32743400
C	4.52865500	-3.03079400	-2.07307800
H	2.90552100	-1.73775400	-2.74832000
C	5.05156700	-3.77421500	-1.00295500
H	4.76517100	-4.38836600	1.06053200
H	5.05458100	-3.00451500	-3.03345400
H	5.98837300	-4.32788200	-1.12337800
C	1.43675700	3.56153200	-0.24626300
C	2.41615500	3.86203100	0.71986400
C	1.38551200	4.31561700	-1.43781700
C	3.31983100	4.91184900	0.50071400
H	2.46280900	3.27395600	1.63889700
C	2.29147100	5.36532100	-1.64977800
H	0.65217600	4.04940500	-2.20544600
C	3.26200600	5.66778100	-0.68198400
H	4.07231300	5.14028600	1.26266100
H	2.24447500	5.93825100	-2.58173400
H	3.97339400	6.48266100	-0.85017400
C	-3.14081500	2.61540400	-0.22190300
C	-4.11524600	2.24848500	0.72769800
C	-3.47204200	3.53075700	-1.24281800
C	-5.40118700	2.80546100	0.66271800
H	-3.85118400	1.53807900	1.51635100
C	-4.75902000	4.08625000	-1.29663300
H	-2.72755400	3.77907100	-2.00579900
C	-5.72788600	3.72698300	-0.34590900
H	-6.14894300	2.51987400	1.41001600
H	-5.00780000	4.79206600	-2.09603500
H	-6.73299500	4.15787300	-0.39442600

[1]INT2a

Electronic Energy (BP86)	−2894.998388
Zero-point correction	0.628421
Thermal correction to Energy	0.675936
Thermal correction to Enthalpy	0.676880
Thermal correction to Gibbs Free Energy	0.541306

W	1.17807800	0.77983600	-0.06322700
W	-1.07233200	-0.27757500	-0.40291800

Cl	2.05910500	1.64867200	-2.06977200
Cl	-1.78254000	-0.58144100	-2.66146000
N	1.96180800	-1.12125600	-0.98054800
N	-0.12715400	-2.17420500	-0.68189400
N	0.14652400	2.74663900	0.02132300
N	-1.87815300	1.72561700	-0.65709900
C	-1.77621300	-0.46073400	1.47967900
C	2.36328500	0.15789300	1.59159000
C	1.16229400	-2.17921400	-1.07714900
H	1.57726300	-3.11926900	-1.47406900
C	-1.12437600	2.80182800	-0.38094200
H	-1.60015000	3.79208300	-0.46570200
C	-0.56610300	0.13320100	1.62226500
H	-0.04821400	0.48108700	2.51247700
C	-2.87211700	-0.90458900	2.33640900
C	-2.85609700	-0.64762200	3.73107000
C	-3.97899200	-1.60010200	1.78803500
C	-3.91288200	-1.07115400	4.54560700
H	-2.00701700	-0.11005800	4.16625300
C	-5.03401100	-2.02291200	2.60805800
H	-3.99411600	-1.80905000	0.71287500
C	-5.00534200	-1.75962900	3.98794900
H	-3.88652300	-0.86378800	5.62061200
H	-5.88140500	-2.55967600	2.16877500
H	-5.83061200	-2.08891200	4.62775300
C	3.01182100	-0.93075700	2.31190200
C	4.13271700	-0.67385800	3.14300100
C	2.52324200	-2.25674800	2.22083700
C	4.74567000	-1.71346700	3.85266200
H	4.51737700	0.34926300	3.21525700
C	3.12972500	-3.29013500	2.94861100
H	1.65106200	-2.45475500	1.59095900
C	4.24532900	-3.02468800	3.76012100
H	5.61513300	-1.50158300	4.48424800
H	2.73373200	-4.30872800	2.87709600
H	4.72435300	-3.83555900	4.31872800
C	2.09320800	1.44957900	1.69027900
H	2.16879200	2.29086300	2.38471800

C	-3.19359100	1.99508500	-1.15713800
C	-3.38431500	2.86369200	-2.25249100
C	-4.31086300	1.37503900	-0.56094500
C	-4.67840700	3.12181700	-2.72884700
H	-2.51218800	3.30660100	-2.74422400
C	-5.60006800	1.63315000	-1.04850000
H	-4.16221900	0.70962500	0.29474100
C	-5.79068500	2.50839000	-2.13094300
H	-4.81362000	3.79460400	-3.58225400
H	-6.46096900	1.15190800	-0.57256400
H	-6.79873700	2.70601600	-2.50916700
C	0.69683700	3.97305200	0.51460500
C	1.99260400	4.38893700	0.13769700
C	-0.04289100	4.77282200	1.41612300
C	2.52010400	5.58755600	0.63836800
H	2.56570900	3.77679000	-0.56237400
C	0.49097100	5.97493200	1.90589100
H	-1.02788000	4.43283500	1.75353300
C	1.77480900	6.38935900	1.51980200
H	3.52295200	5.89979200	0.32803700
H	-0.09711200	6.57932800	2.60477300
H	2.19393600	7.32363200	1.90674200
C	-0.87070300	-3.38542300	-0.86642300
C	-0.85815200	-4.07667900	-2.09760500
C	-1.64842800	-3.88214800	0.20024100
C	-1.59921600	-5.25802500	-2.24668100
H	-0.29497800	-3.66538200	-2.94079400
C	-2.39089100	-5.06174900	0.03995300
H	-1.65254700	-3.34278300	1.15193200
C	-2.36709600	-5.75571300	-1.18107000
H	-1.58751000	-5.78284900	-3.20768800
H	-2.98517100	-5.44115600	0.87776100
H	-2.94741200	-6.67577200	-1.30359400
C	3.25583700	-1.26372100	-1.58348600
C	4.41043300	-0.78336800	-0.93189500
C	3.38720200	-1.86000200	-2.85778500
C	5.67015000	-0.93416200	-1.52700400
H	4.31642800	-0.30485000	0.04514300

C	4.65216300	-2.01174800	-3.44422800
H	2.48967700	-2.16491700	-3.40518100
C	5.80078900	-1.55371500	-2.78111400
H	6.55707000	-0.56340100	-1.00254100
H	4.73345600	-2.47135100	-4.43488000
H	6.78693500	-1.66404100	-3.24322400

[1]TS3a

Electronic Energy (BP86)	−2894.978805
Zero-point correction	0.628098
Thermal correction to Energy	0.674797
Thermal correction to Enthalpy	0.675741
Thermal correction to Gibbs Free Energy	0.542235

W	1.05523100	0.52977100	-0.28519400
W	-1.31155800	-0.19813300	-0.33130000
Cl	1.70110400	1.36833000	-2.42220800
Cl	-2.75944300	-0.12913900	-2.22756800
N	1.59982100	-1.46419500	-1.09917100
N	-0.67747600	-2.10464600	-1.14012300
N	0.36621200	2.63460000	-0.15386700
N	-1.86780700	1.88300700	-0.11752700
C	-1.47019700	-0.65521300	1.62194800
C	2.33842700	0.19286400	1.40717000
C	0.62316800	-2.33575800	-1.36865900
H	0.90352600	-3.31562000	-1.78800000
C	-0.94268000	2.86020700	-0.11090500
H	-1.29627200	3.90152600	-0.04460400
C	-0.11729400	-0.76946900	1.38160300
H	0.56222900	-1.56313400	1.70118200
C	-2.35039000	-1.04489100	2.71074600
C	-1.85098100	-1.17422200	4.03403300
C	-3.72596900	-1.31589800	2.48170000
C	-2.69516200	-1.55483600	5.08359100
H	-0.79340600	-0.96148100	4.22344400
C	-4.56259900	-1.70623400	3.53512200
H	-4.12199000	-1.22761400	1.46412400
C	-4.05380100	-1.82464500	4.84018300
H	-2.29225900	-1.64371300	6.09827600
H	-5.61850000	-1.91914200	3.33691700

H	-4.71099500	-2.12528900	5.66254900
C	3.52222800	-0.33717000	2.05488700
C	4.71623600	-0.60374700	1.33660100
C	3.50988700	-0.56500100	3.46011100
C	5.85763700	-1.06560200	2.00290600
H	4.73568500	-0.43736400	0.25623300
C	4.64697800	-1.04545200	4.11627500
H	2.59163600	-0.35986300	4.02079800
C	5.82664700	-1.29276500	3.38957400
H	6.77328700	-1.25878900	1.43512500
H	4.61982700	-1.22365500	5.19648800
H	6.71899900	-1.66332100	3.90503600
C	1.20785100	0.82161900	1.78878800
H	0.82467800	1.39906100	2.63605100
C	-3.23331600	2.33109600	-0.11535000
C	-4.10718600	1.90544400	0.90430200
C	-3.71065800	3.18876300	-1.12851600
C	-5.44243500	2.33738000	0.90955800
H	-3.72704900	1.25319900	1.69530700
C	-5.04476000	3.62075200	-1.11254600
H	-3.03891400	3.48407500	-1.94076000
C	-5.91579900	3.19624600	-0.09563400
H	-6.11197300	2.00552700	1.71011100
H	-5.40769000	4.28065700	-1.90753500
H	-6.95811600	3.53090800	-0.08878200
C	1.22971700	3.76733400	-0.24901400
C	2.42589100	3.80743900	0.50033600
C	0.92514600	4.84628000	-1.10981100
C	3.28281200	4.91350900	0.40582500
H	2.67070400	2.97378200	1.16390300
C	1.78149600	5.95329000	-1.18832200
H	0.03513700	4.79424600	-1.74459100
C	2.96368900	5.99467100	-0.43146000
H	4.20441900	4.92921700	0.99717400
H	1.53192900	6.77822200	-1.86399700
H	3.63530400	6.85600000	-0.50282900
C	-1.58975700	-3.13159500	-1.54687700
C	-2.57304200	-3.57933300	-0.64019600

C	-1.53117700	-3.69272400	-2.83968200
C	-3.47487100	-4.58350300	-1.02069500
H	-2.61292500	-3.14474900	0.36399100
C	-2.43184500	-4.70299200	-3.20880500
H	-0.80059800	-3.31015000	-3.55962100
C	-3.40648500	-5.15224000	-2.30353900
H	-4.23078400	-4.92677300	-0.30657600
H	-2.38033600	-5.12770100	-4.21688600
H	-4.11290000	-5.93489200	-2.59793700
C	2.92741900	-1.98340000	-1.27611100
C	3.30637700	-3.19094100	-0.64822000
C	3.86532300	-1.30559000	-2.08419800
C	4.59415000	-3.71603300	-0.83420000
H	2.59085300	-3.70359800	0.00351200
C	5.15361400	-1.83485900	-2.25859000
H	3.56617000	-0.37948200	-2.58024500
C	5.52507500	-3.04014900	-1.63870700
H	4.87116300	-4.65200800	-0.33763800
H	5.86757700	-1.30314500	-2.89674600
H	6.53011700	-3.44952200	-1.78324400

[1]13^Ph

Electronic Energy (BP86)	−2895.039886
Zero-point correction	0.631001
Thermal correction to Energy	0.675936
Thermal correction to Enthalpy	0.678507
Thermal correction to Gibbs Free Energy	0.545988

W	-1.14577500	0.16115900	-0.17714600
W	1.34444100	-0.06352100	-0.31608000
Cl	-2.22082400	0.38807800	-2.27699300
Cl	2.62898500	-0.14267300	-2.31691700
N	-0.91814500	2.30343100	-0.38333400
N	1.42383300	2.10612200	-0.57116200
N	-1.27170700	-1.95725800	-0.65970800
N	1.07729900	-2.19838900	-0.64149300
C	1.76865300	-0.08130100	1.56529500
C	-1.99228600	0.08163400	1.68311900
C	0.29832300	2.82743100	-0.59049800
H	0.37725600	3.91567300	-0.74103500

C	-0.14882800	-2.67882000	-0.83698200
H	-0.25756200	-3.73651900	-1.12595000
C	0.37528700	0.43350700	1.73264800
H	0.25076500	1.50600500	1.95670800
C	2.79803300	-0.16056100	2.58334500
C	2.49422800	0.05425300	3.95642300
C	4.14966800	-0.44813000	2.23629300
C	3.49332600	-0.02294500	4.93389100
H	1.46141700	0.28086000	4.24129800
C	5.14147900	-0.52344100	3.21990700
H	4.40570100	-0.60306600	1.18250700
C	4.82080400	-0.31340900	4.57370500
H	3.23528300	0.14537200	5.98506800
H	6.17438700	-0.74337300	2.92921800
H	5.59990700	-0.37267700	5.34048600
C	-3.32847600	-0.18435200	2.18706400
C	-4.48248100	0.10741000	1.40945500
C	-3.51164300	-0.70023200	3.49977500
C	-5.76393200	-0.12956700	1.91894300
H	-4.35638800	0.51461300	0.40048500
C	-4.79685600	-0.93564400	4.00211900
H	-2.63157800	-0.90030500	4.12043600
C	-5.92733500	-0.65251400	3.21466300
H	-6.64109300	0.09647600	1.30393000
H	-4.91955700	-1.33367600	5.01503800
H	-6.93168600	-0.82951900	3.61316700
C	-0.74200700	-0.42878000	2.08242600
H	-0.56875600	-1.44167000	2.48602100
C	-2.48158200	-2.63796100	-1.04114000
C	-2.64750200	-3.10207200	-2.36358200
C	-3.50462500	-2.85320100	-0.09751300
C	-3.82016000	-3.77761200	-2.73149200
H	-1.86314500	-2.90409300	-3.10076200
C	-4.67757700	-3.52581000	-0.47465000
H	-3.37060500	-2.50694100	0.93006000
C	-4.84021900	-3.99023300	-1.78968000
H	-3.93997200	-4.12881800	-3.76177100
H	-5.46363700	-3.69115700	0.26932700

H	-5.75670500	-4.51417100	-2.07964500
C	2.16556000	-3.11398500	-0.82706900
C	3.11234900	-3.27790900	0.20405700
C	2.30843300	-3.84064100	-2.02744400
C	4.18363000	-4.16849800	0.03625200
H	2.98890100	-2.71666000	1.13483100
C	3.37904000	-4.73319700	-2.18344000
H	1.59651400	-3.67730400	-2.84256500
C	4.32051700	-4.90032100	-1.15467200
H	4.91033700	-4.29382100	0.84581400
H	3.48500800	-5.28853600	-3.12128000
H	5.15863400	-5.59272200	-1.28296900
C	2.64240200	2.82645100	-0.80793500
C	2.77641000	3.67559900	-1.92628700
C	3.72547700	2.67378700	0.08145800
C	3.97171300	4.37937500	-2.13620300
H	1.95315600	3.75497300	-2.64348300
C	4.91857000	3.37706000	-0.13959000
H	3.61895900	2.01377800	0.94748400
C	5.04685700	4.23426900	-1.24520800
H	4.06589000	5.03166900	-3.01079500
H	5.75056000	3.25589300	0.56200600
H	5.98090200	4.77911800	-1.41537700
C	-2.02332400	3.21082300	-0.49209200
C	-2.96811500	3.29190000	0.55125200
C	-2.18056900	4.02239900	-1.63584100
C	-4.04367100	4.18755100	0.45613600
H	-2.83967700	2.65753100	1.43275000
C	-3.25785000	4.91684700	-1.72158800
H	-1.47061000	3.92549300	-2.46332400
C	-4.19292800	5.00376600	-0.67762500
H	-4.76590900	4.24908900	1.27700200
H	-3.37260700	5.53805200	-2.61610600
H	-5.03588400	5.69847000	-0.74968600

^{1}22^{Ph}

Electronic Energy (BP86)	−2956.367855
Zero-point correction	0.749536

Thermal correction to Energy	0.804954
Thermal correction to Enthalpy	0.805898
Thermal correction to Gibbs Free Energy	0.647111

W	-0.54352000	0.31495900	-0.99179500
Cl	3.51939100	0.09793700	-1.74404100
Cl	-0.57305900	0.68352400	-3.36696000
N	1.40745500	-2.20328300	-0.67376100
N	-0.80684300	-1.78979100	-1.36535000
N	1.85736700	2.06902300	0.10837700
N	-0.34345600	2.42615700	-0.65350200
C	0.07715200	-0.23321700	0.99928600
C	-7.12587200	-0.92775200	0.98916700
C	-8.03861600	-0.25844900	1.91810000
H	-8.89962800	0.15444800	1.35558200
H	-7.52873400	0.61483100	2.37092400
C	-8.56242700	-1.18489400	3.04464400
H	-9.06912200	-2.05242100	2.58499700
H	-7.69884200	-1.58976200	3.60228400
C	-9.51795700	-0.45603700	3.99971000
H	-10.40142800	-0.06604500	3.46372800
H	-9.02118800	0.40073300	4.48872300
H	-9.88009900	-1.12995900	4.79405800
C	-1.31805200	-0.25225300	1.16234100
H	-1.80532700	-1.23733100	1.02859200
C	-2.10502000	0.61060600	2.18261900
C	-1.26032400	1.78308900	2.71747300
H	-0.34255100	1.41935800	3.21223400
H	-1.83935700	2.36712700	3.45462600
H	-0.96041000	2.46089200	1.90479700
C	-2.47716800	-0.31786100	3.36821300
H	-3.10304900	-1.16395400	3.03398500
H	-3.04677200	0.24154800	4.13219000
H	-1.57363400	-0.73021200	3.85189500
C	-3.40407400	1.13624700	1.53119000
H	-3.18122800	1.81630200	0.69216000
H	-4.00619800	1.69533800	2.27100400
H	-4.02678100	0.30938800	1.14715600
C	0.22163500	-2.62667300	-1.11990100

H	0.07819600	-3.70347500	-1.29923800
C	0.85055300	2.88312200	-0.21094600
H	0.99626200	3.96810800	-0.10093900
C	-6.36731600	-1.50378100	0.22512300
H	-5.71237500	-2.01091300	-0.46159800
W	1.63672900	-0.07505600	-0.17188100
C	4.40235300	-0.76526100	1.50441000
C	2.53697400	-1.06515400	2.86438900
C	5.26455700	-1.17054200	2.53117100
H	4.77271900	-0.47042800	0.51709300
C	3.33117500	-1.47718200	3.93933300
H	1.44552700	-1.00343700	2.93205800
C	4.72407300	-1.53278100	3.77249600
H	6.34253600	-1.19742800	2.34883700
H	2.85968000	-1.75068300	4.88779400
H	5.37302600	-1.85319800	4.59385700
N	3.05471200	-0.71016500	1.66250400
C	2.36215300	-3.24568400	-0.40238300
C	2.06155800	-4.24282000	0.55079600
C	3.59494600	-3.28551500	-1.08438100
C	2.97985300	-5.27154100	0.81209100
H	1.10932500	-4.19611900	1.09011000
C	4.50776500	-4.31664000	-0.81355100
H	3.81798200	-2.50703500	-1.81857700
C	4.20711700	-5.31211200	0.13124000
H	2.73412900	-6.04041800	1.55251600
H	5.45882700	-4.34492000	-1.35606900
H	4.92350200	-6.11478600	0.33443900
C	-2.06038600	-2.40545700	-1.69028900
C	-2.86085400	-1.88590100	-2.73112400
C	-2.52754300	-3.52125900	-0.95619200
C	-4.09011000	-2.48668600	-3.03890500
H	-2.49391600	-1.03056200	-3.30444300
C	-3.75511500	-4.12022600	-1.27726800
H	-1.93375600	-3.90719300	-0.12108800
C	-4.54277000	-3.60820600	-2.32168100
H	-4.69380300	-2.07849600	-3.85624300
H	-4.10009000	-4.98427000	-0.69953700

H	-5.49989400	-4.07660300	-2.57245900
C	-1.37470600	3.41616000	-0.81730700
C	-2.30184900	3.30008900	-1.87614000
C	-1.50527500	4.49882500	0.08504400
C	-3.32241200	4.24965300	-2.02999800
H	-2.19433900	2.48098900	-2.59147900
C	-2.52387800	5.44802200	-0.08383300
H	-0.82780000	4.58542400	0.94015600
C	-3.43961900	5.32966200	-1.14061100
H	-4.02594400	4.14380000	-2.86245000
H	-2.60765500	6.27568400	0.62851700
H	-4.23839800	6.06750700	-1.26585900
C	3.08670800	2.72766100	0.46492300
C	3.78676100	3.48763400	-0.49304700
C	3.59081200	2.61511100	1.77521400
C	4.97919200	4.13544300	-0.13577600
H	3.40156300	3.53916900	-1.51567800
C	4.78412300	3.26650300	2.12393800
H	3.03124100	2.03138200	2.51195400
C	5.48197200	4.02728400	1.17098300
H	5.52023400	4.72053900	-0.88670900
H	5.16409900	3.18528400	3.14791200
H	6.41313900	4.53318900	1.44594700

[1]TS1b

Electronic Energy (BP86)	−2956.330186
Zero-point correction	0.747511
Thermal correction to Energy	0.802499
Thermal correction to Enthalpy	0.803443
Thermal correction to Gibbs Free Energy	0.650739

W	1.16226100	0.10417700	-0.60403700
Cl	-2.22338600	-0.16070800	-2.60663900
Cl	1.50867800	0.15532800	-2.91502000
N	-1.20153300	2.22167800	-0.44967900
N	1.14847600	2.21895100	-0.44439900
N	-1.01717200	-2.19580800	-0.43856600
N	1.32212000	-2.01406800	-0.51624800
C	-0.64063900	0.08351200	1.53224500
C	3.99318400	0.15421000	1.11230500

C	5.37069600	0.14303200	0.63602000
H	5.52990700	1.02077600	-0.01990500
H	5.53000900	-0.76259200	0.01959700
C	6.40332300	0.16921800	1.79762900
H	6.22634700	1.06837600	2.41400400
H	6.22800100	-0.70352300	2.45132000
C	7.84610100	0.15951600	1.27367300
H	8.04730200	1.03765100	0.63548700
H	8.04907200	-0.74494400	0.67407200
H	8.56778100	0.17844600	2.10710900
C	-0.43058500	0.23176300	2.85443600
H	-0.38978200	1.26505500	3.25175600
C	-0.29056300	-0.85372300	3.93526300
C	-0.06914100	-2.24414600	3.30662700
H	-0.89593500	-2.50886300	2.62709800
H	-0.00123200	-3.01816900	4.09173300
H	0.86475800	-2.26584200	2.71902800
C	-1.58866600	-0.86756600	4.78535000
H	-1.78117400	0.12308800	5.23442300
H	-1.50985500	-1.60192400	5.60839500
H	-2.46098200	-1.13628300	4.16563000
C	0.90215700	-0.50358500	4.86049200
H	1.85958000	-0.51502600	4.31079900
H	0.98232100	-1.23491700	5.68505400
H	0.77943400	0.49843400	5.30893800
C	-0.04376600	2.86916000	-0.41734600
H	-0.03973600	3.97035600	-0.38927600
C	0.18381000	-2.75560500	-0.48870000
H	0.26873000	-3.85406800	-0.50630900
C	2.80255800	0.16864300	1.44077200
H	1.91907700	0.20880700	2.06217300
W	-1.13705600	-0.00039200	-0.37171100
C	-4.63954300	-0.25537900	-0.29929900
C	-3.88347500	0.57754000	1.72922200
C	-5.97380300	-0.20531000	0.12841000
H	-4.36239700	-0.59485500	-1.30364900
C	-5.18772600	0.66349800	2.23480200
H	-3.01497400	0.86492200	2.33061900

C	-6.25637100	0.26306400	1.41906300
H	-6.77092700	-0.52854000	-0.54769300
H	-5.35486600	1.04058200	3.24827400
H	-7.28816300	0.31765300	1.78220200
N	-3.60337200	0.12631600	0.48450400
C	-2.36975700	3.05508100	-0.38600500
C	-2.52247900	3.99464200	0.65642200
C	-3.36731300	2.94409400	-1.37572200
C	-3.65372700	4.82476100	0.69897200
H	-1.75779700	4.05767700	1.43821500
C	-4.49396500	3.77745800	-1.32281800
H	-3.23543000	2.20506100	-2.17210900
C	-4.64321400	4.72055700	-0.29114400
H	-3.76193900	5.54964600	1.51308500
H	-5.25920500	3.69217200	-2.10179100
H	-5.52585200	5.36767000	-0.25800100
C	2.31709800	3.04737400	-0.37814900
C	3.36299700	2.85870000	-1.30685600
C	2.44854100	4.04213700	0.61563700
C	4.51424300	3.65882300	-1.24102400
H	3.24444200	2.10344500	-2.08981800
C	3.60182200	4.83910300	0.67194600
H	1.65307800	4.17032900	1.35730400
C	4.64061500	4.65128900	-0.25410500
H	5.31068900	3.51425900	-1.97929500
H	3.69086200	5.60409200	1.45072300
H	5.53951200	5.27451700	-0.20857500
C	2.54918100	-2.75584900	-0.49577400
C	3.56377700	-2.45252800	-1.42913200
C	2.76574600	-3.78461300	0.44767300
C	4.76801200	-3.17374100	-1.41810100
H	3.38014400	-1.67290200	-2.17475400
C	3.97223500	-4.50078800	0.45021800
H	1.99322300	-4.00336200	1.19222100
C	4.97960500	-4.19921200	-0.48059200
H	5.53913800	-2.94082600	-2.16065200
H	4.12657100	-5.29319800	1.19039300
H	5.91951800	-4.76051800	-0.47766000

C	-2.12793200	-3.09710200	-0.54831300
C	-2.26445600	-3.92053700	-1.68501300
C	-3.08896700	-3.16376000	0.47992900
C	-3.34288500	-4.81326600	-1.77926000
H	-1.53845100	-3.82779200	-2.49835000
C	-4.16300800	-4.06050900	0.37839200
H	-2.97776800	-2.51416300	1.35267200
C	-4.29376200	-4.88920200	-0.74877900
H	-3.44350400	-5.44428700	-2.66859500
H	-4.89912700	-4.11313600	1.18761800
H	-5.13485900	-5.58590200	-0.82546000

^{1}INT1b

Electronic Energy (BP86)	−2708.085080
Zero-point correction	0.662441
Thermal correction to Energy	0.710727
Thermal correction to Enthalpy	0.711671
Thermal correction to Gibbs Free Energy	0.574542

W	-1.05231100	0.08497200	-0.40829100
W	1.41899100	-0.02327800	0.01688800
Cl	-1.36964300	0.03490100	-2.75685800
Cl	2.95368300	-0.05393400	-1.79912300
N	-0.79862000	2.27436400	-0.67929200
N	1.51415800	2.11293300	-0.22668200
N	-1.08159800	-2.11193400	-0.54039000
N	1.28015600	-2.17087600	-0.36969400
C	-2.44785800	0.37123200	0.87645100
C	1.43164600	-0.07511800	2.00691500
C	2.11463700	-0.01259400	3.34460500
H	1.45877500	-0.47100300	4.11167900
H	2.18852300	1.06009600	3.62356300
C	3.51975500	-0.64029400	3.42654600
H	3.43938900	-1.72747100	3.24299600
H	4.15177700	-0.23142000	2.61606200
C	4.18883600	-0.39829000	4.78801200
H	3.58477600	-0.81780200	5.61195700
H	4.31636500	0.68085200	4.98589400
H	5.18583300	-0.86705200	4.83326600
C	-3.50319900	0.78907300	1.58435600

H	-3.64810200	1.88438000	1.64381100
C	-4.58298800	-0.02218600	2.30564900
C	-5.91400000	0.14654100	1.52740500
H	-6.18412400	1.21173400	1.42048900
H	-6.73854100	-0.36206100	2.05950400
H	-5.83325500	-0.28927800	0.51771400
C	-4.74832200	0.56973800	3.72899400
H	-3.81894800	0.46500600	4.31547100
H	-5.55484800	0.04508500	4.27173100
H	-5.00756200	1.64267200	3.69073800
C	-4.21796800	-1.51498600	2.40148300
H	-4.09752400	-1.96393600	1.40304500
H	-5.01081400	-2.06885000	2.93468200
H	-3.27309300	-1.65799400	2.95408700
C	0.40682000	2.81492800	-0.54349100
H	0.50879300	3.90586300	-0.66012200
C	0.08508400	-2.75694000	-0.56109400
H	0.07220800	-3.84639800	-0.72152400
C	0.09896100	-0.04004900	1.66072200
H	-0.72941100	-0.05943700	2.36671000
C	-1.89661200	3.19411200	-0.74438800
C	-2.90017700	3.04531900	-1.72484300
C	-1.99843700	4.24763000	0.19228600
C	-3.97434000	3.94559600	-1.77084700
H	-2.81740800	2.23393400	-2.45180200
C	-3.07440100	5.14683100	0.13401500
H	-1.24425500	4.33987100	0.98100800
C	-4.06754200	5.00031000	-0.84711800
H	-4.74155900	3.82327900	-2.54268800
H	-3.13942100	5.95725600	0.86790400
H	-4.90918800	5.69902500	-0.88911600
C	2.74663500	2.84131500	-0.22751600
C	3.08174800	3.71191000	-1.28679800
C	3.65588500	2.67055800	0.83701200
C	4.29501800	4.41591800	-1.26347400
H	2.40429500	3.80662700	-2.14143500
C	4.87017800	3.37068700	0.84890700
H	3.39152000	1.99377100	1.65499900

C	5.19395900	4.24991300	-0.19819900
H	4.54455600	5.08417400	-2.09431800
H	5.56449600	3.23243200	1.68443800
H	6.14336200	4.79445200	-0.18747000
C	-2.25703000	-2.92569800	-0.55448800
C	-2.33169300	-4.10377500	0.22435700
C	-3.37519200	-2.54650700	-1.32897800
C	-3.48801500	-4.89749800	0.20303100
H	-1.49427800	-4.37613000	0.87508400
C	-4.52985400	-3.34271400	-1.33681400
H	-3.31947600	-1.64295800	-1.94074200
C	-4.59300000	-4.52314700	-0.57767100
H	-3.52862000	-5.80383100	0.81650500
H	-5.38417500	-3.03850000	-1.95070900
H	-5.49723300	-5.13985400	-0.58751100
C	2.41972800	-3.01734100	-0.56424900
C	3.50043500	-2.95172000	0.33773400
C	2.49495200	-3.90147900	-1.66280600
C	4.62111200	-3.77440100	0.16187900
H	3.44785700	-2.25271400	1.17654600
C	3.61645900	-4.72780200	-1.82851300
H	1.69005500	-3.91224400	-2.40479400
C	4.68287100	-4.67155000	-0.91767000
H	5.45038900	-3.71406800	0.87464000
H	3.66208100	-5.40496500	-2.68791900
H	5.56061600	-5.31091900	-1.05490800

[1]TS2b

Electronic Energy (BP86)	−2708.080055
Zero-point correction	0.662139
Thermal correction to Energy	0.709527
Thermal correction to Enthalpy	0.710471
Thermal correction to Gibbs Free Energy	0.576848

W	1.18802900	0.05837300	-0.21179500
W	-1.23922300	0.12228500	-0.28525800
Cl	2.55040200	0.53253500	-2.10785800
Cl	-2.24820000	0.39173800	-2.43610300
N	1.03119300	-1.94664700	-1.15091700
N	-1.27227300	-1.98412200	-0.68477800

N	1.22080200	2.23684000	0.04987100
N	-1.11645300	2.32306200	-0.30590600
C	1.60479200	-0.86914800	1.43323400
C	-1.53451300	-0.06119900	1.70384900
C	-2.52336600	-0.14183000	2.82972200
H	-2.46526400	0.78986400	3.43175900
H	-2.16977800	-0.94604200	3.50828600
C	-3.98328800	-0.42841700	2.43236500
H	-4.34540800	0.37003900	1.75762600
H	-4.01897100	-1.36239200	1.84370500
C	-4.91140800	-0.53463200	3.65127000
H	-4.91509800	0.40066200	4.23929400
H	-4.59319800	-1.34954100	4.32575400
H	-5.95060400	-0.74207300	3.34652200
C	2.03616500	-1.62576400	2.45443700
H	2.17739800	-2.69697500	2.21204500
C	2.44209600	-1.27465100	3.88731900
C	3.98190700	-1.44936500	3.99074000
H	4.29099800	-2.46660500	3.69372300
H	4.31703200	-1.28277800	5.03044800
H	4.50607300	-0.73041900	3.33879300
C	1.75444000	-2.28094500	4.84555900
H	0.65589800	-2.19230000	4.79324500
H	2.06541100	-2.09261600	5.88838900
H	2.02419100	-3.32177000	4.59449800
C	2.05446300	0.16188500	4.28564500
H	2.47330100	0.90492000	3.58551400
H	2.43864500	0.39446900	5.29412400
H	0.95908200	0.28632300	4.30746900
C	-0.14418500	-2.57096300	-1.13105700
H	-0.20052200	-3.61057700	-1.48894600
C	0.07217500	2.91198300	-0.11723800
H	0.10121400	4.01154600	-0.06151700
C	-0.24447000	0.38760800	1.64741800
H	0.22530500	1.10455900	2.32605200
C	-2.22927800	3.18648400	-0.54760900
C	-2.15174700	4.23170300	-1.49346300
C	-3.44019600	2.97460400	0.14330900

C	-3.25934600	5.06180500	-1.72017500
H	-1.23380100	4.36356400	-2.07514000
C	-4.54641100	3.80215600	-0.09470000
H	-3.49738500	2.16399300	0.87676900
C	-4.46022100	4.85283100	-1.02323300
H	-3.18697700	5.86447400	-2.46159100
H	-5.47862700	3.62716300	0.45267400
H	-5.32546200	5.49672000	-1.20962100
C	2.42658900	2.99471500	0.15684400
C	2.64338300	4.18072200	-0.57982000
C	3.45163100	2.52051200	1.00303500
C	3.84416200	4.89040300	-0.43728200
H	1.89004400	4.52418500	-1.29534600
C	4.65386800	3.22921900	1.13186900
H	3.28887400	1.58805200	1.55438700
C	4.85365100	4.42311000	0.41977800
H	3.99907000	5.80407800	-1.02077100
H	5.43722500	2.84570800	1.79402300
H	5.79295000	4.97598300	0.51938500
C	2.11373100	-2.65942500	-1.75807400
C	1.95196500	-3.29075100	-3.00975600
C	3.36158000	-2.73245700	-1.10418000
C	3.01390700	-4.00973400	-3.57935700
H	1.00428600	-3.18391200	-3.54722700
C	4.41844700	-3.44703700	-1.68317100
H	3.48785700	-2.22973200	-0.14034900
C	4.24971400	-4.09341800	-2.91980400
H	2.87600100	-4.49050500	-4.55356400
H	5.38126900	-3.49782400	-1.16381900
H	5.07956300	-4.64687800	-3.37067600
C	-2.40408300	-2.83732400	-0.48968700
C	-3.69481300	-2.42646500	-0.89065800
C	-2.24980200	-4.09186800	0.14514500
C	-4.79931600	-3.26296600	-0.66921400
H	-3.81460700	-1.46892400	-1.40463200
C	-3.35972200	-4.92408700	0.35245700
H	-1.25998200	-4.39678500	0.50055600
C	-4.64063000	-4.51465000	-0.05164100

H	-5.79151100	-2.93407000	-0.99610200
H	-3.22112100	-5.89179900	0.84641500
H	-5.50655400	-5.16292400	0.11600800

[1]25[Ph]

Electronic Energy (BP86)	−2708.121992
Zero-point correction	0.664662
Thermal correction to Energy	0.711970
Thermal correction to Enthalpy	0.712914
Thermal correction to Gibbs Free Energy	0.579356

W	1.18891700	-0.02634800	-0.32154100
W	-1.17491000	0.25485700	-0.38735000
Cl	2.44260100	0.19172500	-2.34655000
Cl	-2.25996900	0.71293200	-2.48595000
N	0.95011100	-2.07327800	-0.90175500
N	-1.39577800	-1.89236500	-0.85342600
N	1.45187300	2.08065300	-0.09098300
N	-0.87590400	2.41686100	-0.21250700
C	1.18574500	-0.46911100	1.72768900
C	-1.42689800	-0.09504600	1.53068800
C	-2.44986100	-0.28980500	2.60540200
H	-2.39824800	0.57303000	3.30796100
H	-2.12946700	-1.15986100	3.22135600
C	-3.90112900	-0.50649800	2.13739500
H	-4.21858000	0.35619600	1.52157200
H	-3.93695200	-1.38693400	1.47184100
C	-4.87535800	-0.69404300	3.30994600
H	-4.87923900	0.18627200	3.97771800
H	-4.60097700	-1.57234800	3.92123800
H	-5.90712000	-0.84718400	2.95232700
C	2.00367400	-1.03729200	2.64699200
H	2.89087000	-1.57020600	2.26653100
C	1.89560500	-1.11098600	4.16748600
C	3.27141300	-0.69920000	4.75676800
H	4.08453600	-1.33181500	4.35891000
H	3.26693600	-0.80734800	5.85589400
H	3.50839700	0.35199700	4.51846500
C	1.61062800	-2.58959300	4.54956900
H	0.63021600	-2.91580100	4.16320300

H	1.60701000	-2.70868600	5.64815700
H	2.37987100	-3.26647200	4.13831500
C	0.80040800	-0.20187000	4.75489100
H	0.97986400	0.85738500	4.50459100
H	0.78481800	-0.29146700	5.85535900
H	-0.19920900	-0.47633000	4.38124400
C	-0.28762500	-2.60629600	-1.02043900
H	-0.37483900	-3.68005800	-1.24819100
C	0.36665300	2.88829900	-0.09999700
H	0.51750000	3.97312700	0.01683900
C	-0.04147900	0.32946600	1.74314400
H	0.08130000	1.36160200	2.13433800
C	-1.93310900	3.37955600	-0.28175200
C	-3.07724000	3.21435700	0.52495700
C	-1.86275200	4.47419900	-1.16914700
C	-4.12767500	4.14022500	0.45585700
H	-3.12243000	2.36261300	1.21058400
C	-2.91548700	5.39901800	-1.22839400
H	-1.00098600	4.57121100	-1.83692200
C	-4.05029000	5.23798700	-0.41734100
H	-5.00926600	4.00394200	1.09087600
H	-2.85411500	6.23980300	-1.92715700
H	-4.87328400	5.95745000	-0.47233500
C	2.73443200	2.71410600	-0.07929400
C	3.74258500	2.19773200	0.76163300
C	3.02455400	3.81257900	-0.91683800
C	5.01766200	2.78090800	0.77212700
H	3.50587000	1.34983900	1.41327900
C	4.30017400	4.39431000	-0.89379700
H	2.26318800	4.17994800	-1.61203800
C	5.30063600	3.88345900	-0.05077900
H	5.79156100	2.37368700	1.43115100
H	4.51760300	5.24022300	-1.55419000
H	6.29702400	4.33641400	-0.04215600
C	-2.61695500	-2.64040900	-0.76502000
C	-3.76137400	-2.23779100	-1.48587800
C	-2.68987900	-3.77959400	0.06955900
C	-4.94952000	-2.97557900	-1.37825700

H	-3.69885400	-1.35831300	-2.13164600
C	-3.88189000	-4.51366000	0.16374800
H	-1.81540200	-4.06797900	0.66240500
C	-5.01768300	-4.11506400	-0.55895300
H	-5.82806300	-2.65747500	-1.94942500
H	-3.92308600	-5.39313300	0.81519600
H	-5.94922100	-4.68470100	-0.48060000
C	2.03394600	-2.91262300	-1.30980200
C	1.94959000	-3.71895400	-2.46639700
C	3.22952100	-2.91063000	-0.56126700
C	3.03289800	-4.52566500	-2.84371200
H	1.05125900	-3.67890800	-3.09040200
C	4.31078500	-3.71295900	-0.94842000
H	3.29636200	-2.27934300	0.33006100
C	4.21579600	-4.52969200	-2.08751900
H	2.95613700	-5.13943400	-3.74728000
H	5.23106300	-3.70075500	-0.35518100
H	5.06159900	-5.15518500	-2.38983400

8 References

1. Fulmer, G. R.; Miller, A. J. M.; Sherden, N. H.; Gottlieb, H. E.; Nudelman, A.; Stoltz, B. M.; Bercaw, J. E.; Goldberg, K. I. NMR Chemical Shifts of Trace Impurities: Common Laboratory Solvents, Organics, and Gases in Deuterated Solvents Relevant to the Organometallic Chemist. *Organometallics* **2010**, *29*, 2176−2179.
2. Yang, C.-W.; Mallick, S.; Tsai, Y.-C. Facile Synthesis of “WCl_3(DME)” and Structural and Electronic Characterizations. *J. Chin. Chem. Soc.* **2022**, *69*, 1431–1437.
3. Ottmers, D. M.; Rase, H. F. Potassium Graphites Prepared by a Mixed-Reaction Technique. *Carbon* **1966**, *4*, 125–127.
4. Hsu, C.-W.; Yu, J.-S. K.; Yen, C.-H.; Lee, G.-H.; Wang, Y.; Tsai, Y.-C. Quintuply-Bonded Dichromium(I) Complexes Featuring Metal–Metal Bond Lengths of 1.74 Å. *Angew. Chem. Int. Ed.* **2008**, *47*, 9933–9936.
5. Nijhuis, C. A.; Jellema, E.; Sciarone, T. J. J.; Meetsma, A.; Budzelaar, P. H. M.; Hessen, B. First-Row Transition Metal Bis(Amidinate) Complexes; Planar Four-Coordination of Fe^{II} Enforced by Sterically Demanding Aryl Substituents. *Eur. J. Inorg. Chem*. **2005**, *2005*, 2089–2099.
6. Sheldrick, G. M. SHELXT – Integrated Space-Group and Crystal-Structure Determination. *Acta Crystallogr. A* **2015**, *71*, 3–8.
7. Sheldrick, G. M. Crystal Structure Refinement with SHELXL. *Acta Crystallogr. C* **2015**, *71*, 3–8.
8. Dolomanov, O. V.; Bourhis, L. J.; Gildea, R. J.; Howard, J. A. K.; Puschmann, H. OLEX2: A Complete Structure Solution, Refinement and Analysis Program. *J. Appl. Crystallogr.* **2009**, *42*, 339–341.
9. Evans, D. F. The Determination of the Paramagnetic Susceptibility of Substances in Solution by Nuclear Magnetic Resonance. *J. Chem. Soc.* **1959**, 2003–2005.
10. Sur, S. K. Measurement of Magnetic Susceptibility and Magnetic Moment of Paramagnetic Molecules in Solution by High-Field Fourier Transform NMR Spectroscopy. *J. Magn. Reson.* **1989**,

82, 169–173.

11. Schubert, E. M. Utilizing the Evans Method with a Superconducting NMR Spectrometer in the Undergraduate Laboratory. *J. Chem. Educ.* **1992**, *69*, 62–64.

12. Bain, G. A.; Berry, J. F. Diamagnetic Corrections and Pascal's Constants. *J. Chem. Educ.* **2008**, *85*, 532–536.

13. Frisch, M. J.; Trucks, G. W.; Schlegel, H. B.; Scuseria, G. E.; Robb, M. A.; Cheeseman, J. R.; Scalmani, G.; Barone, V.; Petersson, G. A.; Nakatsuji, H.; Li, X.; Caricato, M.; Marenich, A. V.; Bloino, J.; Janesko, B. G.; Gomperts, R.; Mennucci, B.; Hratchian, H. P.; Ortiz, J. V.; Izmaylov, A. F.; Sonnenberg, J. L.; Williams-Young, D.; Ding, F.; Lipparini, F.; Egidi, F.; Goings, J.; Peng, B.; Petrone, A.; Henderson, T.; Ranasinghe, D.; Zakrzewski, V. G.; Gao, J.; Rega, N.; Zheng, G.; Liang, W.; Hada, M.; Ehara, M.; Toyota, K.; Fukuda, R.; Hasegawa, J.; Ishida, M.; Nakajima, T.; Honda, Y.; Kitao, O.; Nakai, H.; Vreven, T.; Throssell, K.; Montgomery, J. A., Jr.; Peralta, J. E.; Ogliaro, F.; Bearpark, M. J.; Heyd, J. J.; Brothers, E. N.; Kudin, K. N.; Staroverov, V. N.; Keith, T. A.; Kobayashi, R.; Normand, J.; Raghavachari, K.; Rendell, A. P.; Burant, J. C.; Iyengar, S. S.; Tomasi, J.; Cossi, M.; Millam, J. M.; Klene, M.; Adamo, C.; Cammi, R.; Ochterski, J. W.; Martin, R. L.; Morokuma, K.; Farkas, O.; Foresman, J. B.; Fox, D. J. Gaussian 16, Revision C.01; Gaussian, Inc.: Wallingford, CT, **2016**.

14. Perdew, J. P. Density-Functional Approximation for the Correlation Energy of the Inhomogeneous Electron Gas. *Phys. Rev. B* **1986**, *33*, 8822–8824.

15. Becke, A. D. Density-Functional Exchange-Energy Approximation with Correct Asymptotic Behavior. *Phys. Rev. A* **1988**, *38*, 3098–3100.

16. Fukui, K. Formulation of the reaction coordinate. *J. Phys. Chem.* **1970,** *74*, 4161−4163.

17. Fukui, K. The path of chemical reactions-the IRC approach. *Acc. Chem. Res.* **1981,** *14*, 363−368.

18. Martin, J. M. L.; Sundermann, A. Correlation Consistent Valence Basis Sets for Use with the Stuttgart–Dresden–Bonn Relativistic effective core potentials: The Atoms Ga–Kr and In–Xe. *J.*

Chem. Phys. **2001**, *114*, 3408–3420.

19. Andrae, D.; Häußermann, U.; Dolg, M.; Stoll, H.; Preuß, H. Energy-adjusted *ab initio* pseudopotentials for the second and third row transition elements. *Theoret. Chim. Acta* **1990**, *77*, 123–141.

20. Ditchfield, R.; Hehre, W. J.; Pople, J. A. Self-Consistent Molecular-Orbital Methods. IX. An Extended Gaussian-Type Basis for Molecular-Orbital Studies of Organic Molecules. *J. Chem. Phys.* **1971**, *54*, 724–728.

21. Hehre, W. J.; Ditchfield, R.; Pople, J. A. Self-Consistent Molecular-Orbital Methods. XII. Further Extensions of Gaussian-Type Basis Sets for Use in Molecular Orbital Studies of Organic Molecules. *J. Chem. Phys.* **1972**, *56*, 2257–2261.

22. Hariharan, P. C.; Pople, J. A. The Influence of Polarization Functions on Molecular Orbital Hydrogenation Energies. *Theor. Chim. Acta* **1973**, *28*, 213–222.

23. Francl, M. M.; Pietro, W. J.; Hehre, W. J.; Binkley, J. S.; Gordon, M. S.; DeFrees, D. J.; Pople, J. A. Self-Consistent Molecular Orbital Methods. XXIII. A Polarization-Type Basis Set for Second-Row Elements. *J. Chem. Phys.* **1982**, *77*, 3654–3665.

24. Gordon, M. S.; Binkley, J. S.; Pople, J. A.; Pietro, W. J.; Hehre, W. J. Self-Consistent Molecular-Orbital Methods. 22. Small Split-Valence Basis Sets for Second-Row Elements. *J. Am. Chem. Soc.* **1982**, *104*, 2797–2803.

25. Lu, T.; Chen, F. Multiwfn: A Multifunctional Wavefunction Analyzer. *J. Comput. Chem.* **2012**, *33*, 580–592.

26. Lu, T. A Comprehensive Electron Wavefunction Analysis Toolbox for Chemists, Multiwfn. *J. Chem. Phys.* **2024**, *161*, 082503.

27. Pipek, J.; Mezey, P. G. A Fast Intrinsic Localization Procedure Applicable for Ab Initio and Semiempirical Linear Combination of Atomic Orbital Wave Functions. *J. Chem. Phys.* **1989**, *90*, 4916–4926.

28. Mayer, I. Charge, Bond Order and Valence in the AB Initio SCF Theory. *Chem. Phys. Lett.* **1983**,

97 (3), 270–274.

29. Giambiagi, M.; de Giambiagi, M. S.; Mundim, K. C. Definition of a Multicenter Bond Index. *Struct. Chem.* **1990**, *1*, 423–427.

30. Chen, Z.; Wannere, C. S.; Corminboeuf, C.; Puchta, R.; Schleyer, P. von R. Nucleus-Independent Chemical Shifts (NICS) as an Aromaticity Criterion. *Chem. Rev.* **2005**, *105*, 3842–3888.
